# Transcription factor *CsESE3* positively modulates both jasmonic acid and wax biosynthesis in citrus

**DOI:** 10.1007/s42994-022-00085-2

**Published:** 2022-11-22

**Authors:** Haoliang Wan, Haiji Qiu, Zhuoran Li, Xiaoliang Zhang, Jingyu Zhang, Deyuan Jiang, Alisdair R. Fernie, Yi Lyu, Yunjiang Cheng, Weiwei Wen

**Affiliations:** 1 Key Laboratory of Horticultural Plant Biology (MOE), College of Horticulture and Forestry Sciences, Huazhong Agricultural University, Wuhan, 430070 China; 2 Shenzhen Institute of Nutrition and Health, Huazhong Agricultural University, Wuhan, 430070 China; 3 Shenzhen Branch, Guangdong Laboratory for Lingnan Modern Agriculture, Genome Analysis Laboratory of the Ministry of Agriculture, Agricultural Genomics Institute at Shenzhen, Chinese Academy of Agricultural Sciences, Shenzhen, 518000 China; 4 Max-Planck-Institute of Molecular Plant Physiology, Am Muehlenberg 1, 14476 Potsdam, Germany; 5 Key Laboratory for Space Bioscience and Biotechnology, School of Life Sciences, Northwestern Polytechnical University, Xi’an, 710072 China

**Keywords:** Citrus, Cuticular wax, Jasmonic acid, SHINE transcription factor, Lipase

## Abstract

PLIP lipases can initiate jasmonic acid (JA) biosynthesis. However, little is known about the transcriptional regulation of this process. In this study, an ERF transcription factor (*CsESE3*) was found to be co-expressed with all necessary genes for JA biosynthesis and several key genes for wax biosynthesis in transcriptomes of ‘Newhall’ navel orange. *CsESE3* shows partial sequence similarity to the well-known wax regulator SHINEs (SHNs), but lacks a complete MM protein domain. Ectopic overexpression of *CsESE3* in tomato (OE) resulted in reduction of fruit surface brightness and dwarf phenotype compared to the wild type. The OE tomato lines also showed significant increases in the content of wax and JA and the expression of key genes related to their biosynthesis. Overexpression of *CsESE3* in citrus callus and fruit enhanced the JA content and the expression of JA biosynthetic genes. Furthermore, *CsESE3* could bind to and activate the promoters of two phospholipases from the PLIP gene family to initiate JA biosynthesis. Overall, this study indicated that *CsESE3* could mediate JA biosynthesis by activating *PLIP* genes and positively modulate wax biosynthesis. The findings provide important insights into the coordinated control of two defense strategies of plants represented by wax and JA biosynthesis.

**Supplementary Information:**

The online version contains supplementary material available at 10.1007/s42994-022-00085-2.

## Introduction

Physiological barriers and signaling networks are critical to the defense of plants against various external environmental stresses, which are typically represented by cuticular wax and jasmonic acid (JA), respectively (Wasternack and Hause 2013; Yeats and Rose 2013). Citrus fruit is rich in wax and JA. The cuticular wax of citrus fruit constitutes the outmost barrier, which can maintain fruit morphology and reduce non-stomatal water loss, UV damage and pathogen invasion (Fan et al. 2014; Johann et al. 2007; Sala 2000). In addition, as signaling molecules in response to various stresses, JA and its analogs are widely involved in the stimulation of protective compounds and resistance to drought and green mold disease (Baswal et al. 2020; He et al. 2018; Jain et al. 2019; Long et al. 2019; Moosa et al. 2019; Raza et al. 2020).

**Fig. 1:**
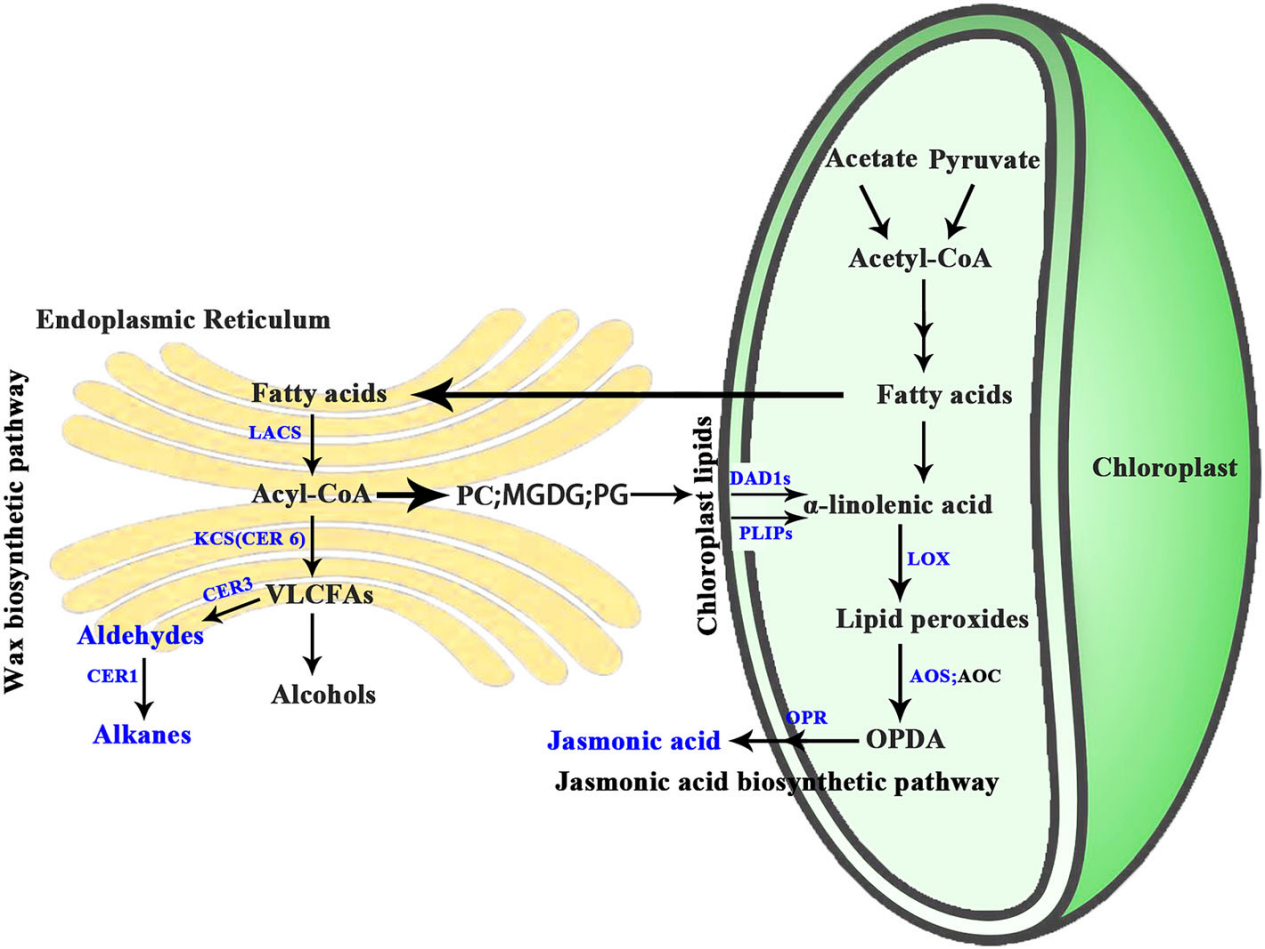
Overview of fatty acid, wax, and JA metabolism in plants. Cell compartments represented include chloroplast (*green*) and endoplasmic reticulum (*yellow*). Enzymes in the pathway proposed in this work are highlighted in *blue*. *LACS* Long-Chain Acyl-CoA Synthetase, *CER 1/3/6* Eceriferum 1/3/6, *LOX* Lipoxygenase, *AOS* Allene Oxide Synthase, *AOC* Allene Oxide Cyclase, *OPR* Oxo-Phytodienoic Acid Reductase, *VLCFA* very long-chain fatty acid, *PC* Phosphatidylcholine, *PG* Phosphatidylglycerol, *MGDG* Monogalactosyl Diacylglycerol

The biosynthesis of both wax and JA involves fatty acids as the precursor, and is strictly regulated by relevant enzymes (Fig. 1). In ‘Newhall’ navel orange, long-chain fatty aldehydes are dominant wax components, followed by alkenes (Wang et al. 2014). Fatty acids are first elongated into very long-chain fatty acids (VLCFAs), which are subsequently reduced to form aldehydes by eceriferum 3 (CER3), and then decarbonylated to form alkanes by eceriferum 1 (CER1), respectively (Kunst and Samuels 2009). For JA, its biosynthesis is initiated by chloroplast-localized phospholipase A1 (PLA1) to hydrolyze glycerolipids to release free polyunsaturated fatty acids (PUFAs) (Fig. 1). These PUFAs are sequentially metabolized by lipoxygenases (LOX), allene oxide synthase (AOS) and OPDA reductase (OPR) to produce JA (Porta and Rocha-Sosa 2002; Wasternack and Hause 2013). The lipase-catalyzed reaction is regarded as the rate-limiting step in JA biosynthesis (Christeller and Galis 2014). Two PLA1 family genes, namely the DAD1-LIKE LIPASE (DALL) and PLASTID LIPASE (PLIP) family, have been thoroughly reported to provide PUFAs for JA (Hyun et al. 2008; Park et al. 2002; Rudus et al. 2014; Ryu 2004; Seo et al. 2009; Stintzi 2000; Wang et al. 2017, 2018). However, there has been little information about the relationship between these two lipase gene families, as well as the regulatory mechanism of them to date.

Transcription factors (TFs) are important in regulating both JA and wax biosynthesis. For JA, members from ethylene response factor (ERF) gene family have been proved to regulate JA. For example, *OsEIL1* can bind to the promoters of *OsLOX9* and *OsAOS2* to activate its biosynthesis in rice (*Oryza sativa*) (Wang et al. 2012). In addition, *AtORA47* (homologue of *OsEIL1*) induces the expression of DAD1, LOX2, AOS, AOC and OPR3 under drought and wound conditions (Ma et al. 2020). Furthermore, in tomato, ERF15 and ERF16 bind to the GC-rich regions (CCG(A/T)CC) of *TomLOXD*, *AOC* and *OPR3* to upregulate their expression and increase the level of JA (Hu et al. 2021). TCP transcription factors in *Arabidopsis* and a single GRAS-domain transcription factor PAT1 (Phytochrome A signal transduction) in grapevine which regulate JA were also reported (Danisman et al. 2012; Wang et al. 2021). For wax, TF members from Myb family, including *Myb96*, *Myb94*, *Myb16*, *Myb106* in *Arabidopsis*, TF members from HD-Zip IV family, including *ZmOCL1* in Maize, *HDG1* in *Arabidopsis*, and *CD2* in tomato, TF members from ERF family, including *WRINKLED 3/4*, SHINE transcriptional factors (SHNs), and *DEWAX* are involved in the regulation of wax biosynthesis (Lee and Suh 2015; Shaheenuzzamn et al. 2021). Among all these wax-regulating TFs, SHNs are the first reported and most extensively studied ones. The SHN gene family comprises three members*.* Overexpression of SHNs resulted in dwarf plants, glossy leaf surface and a higher wax content (by 4.5-fold) (Aharoni et al. 2004). Regulation of wax by SHNs is highly conserved in plants and has been widely reported in many species such as rice, barley (*Hordeum vulgare *L.), wheat (*Triticum aestivum* L.), tomato (*Solanum lycopersicum*) and *Brassica napus* (*B. napus*) (Al-Abdallat et al. 2014; Jäger et al. 2014; Liu et al. 2019; Taketa et al. 2008; Wang et al. 2012).

The SHN gene family can be classified into the ERF-Va1 group (SHN group) based on three characteristic conserved domains, including the AP2 domain, MM domain and CM domain*.* Genes in ERF-Va2 group (SHN-Like group) showed high sequence similarity with SHNs, but lack a complete MM domain (Nakano et al. 2014). The genes in the SHN sub-group have been confirmed with conserved functions in cuticle (mainly wax and cutin) regulation. By contrast, genes in the SHN-Like sub-group are involved in diverse processes. *VviERF045* in grape berries and *PeERF1* in *Phalaenopsis* flowers are involved in cuticle formation (Lai et al. 2019; Leida et al. 2016), while *AtESE3* (At5g25190) in *Arabidopsis, SlERF52* in tomato and *PtaERF003* in *Populus* play essential roles in salt stress, flower pedicel abscission and adventitious and lateral root formation, respectively (Aharoni et al. 2004; Nakano et al. 2014; Trupiano et al. 2013; Zhang et al. 2011). ERF-Va2 genes have similar sequence structures to SHN TFs. However, in contrast to the well-characterized SHN TFs, the ERF-Va2 genes have been poorly understood for their regulatory properties.

Co-regulation of wax and JA biosynthesis has been reported in response to abiotic stress (Gutiérrez et al. 2021; Zhao et al. 2020), and SHNs might play a role in this co-regulation. Silencing *GhSHN2* in cotton not only decreased cuticle load, but also suppressed expression of jasmonic acid (JA) biosynthetic genes (Li et al. 2019a). Previously, we performed transcriptomic and metabolomic analysis on the peels of ‘Newhall’ navel orange fruits at four developmental stages and found that the total wax and JA content are mainly accumulated when fruits are close to the mature stage (Wan et al. 2020, 2021). A SHN-Like transcription factor *CsESE3* was found to be expressed in a coordinated manner with the content of wax and JA as well as several related key genes. Furthermore, *CsESE3* was significantly down-regulated in a wax-deficient mutant of ‘Newhall’ navel orange (gl-Mutant) compared with that in the WT across all developmental stages (Wan et al. 2020). *CsESE3* is therefore regarded as an important candidate associated with both two pathways.

In this study, we attempted to determine whether and how *CsESE3* modulates both wax and JA biosynthesis. The results showed that *CsESE3* could induce the biosynthesis of JA in citrus and both wax and JA biosynthesis in transgenic tomato lines. Further, *CsESE3* was found to promote JA biosynthesis by activating PLIP lipases rather than DAD1 lipases. These findings contribute to a better understanding of the coordinated regulation of wax and JA biosynthesis in citrus.

## Results

### *CsESE3* is a potential key regulatory gene of wax and jasmonic acid biosynthesis in ‘Newhall’ navel orange

**Fig. 2:**
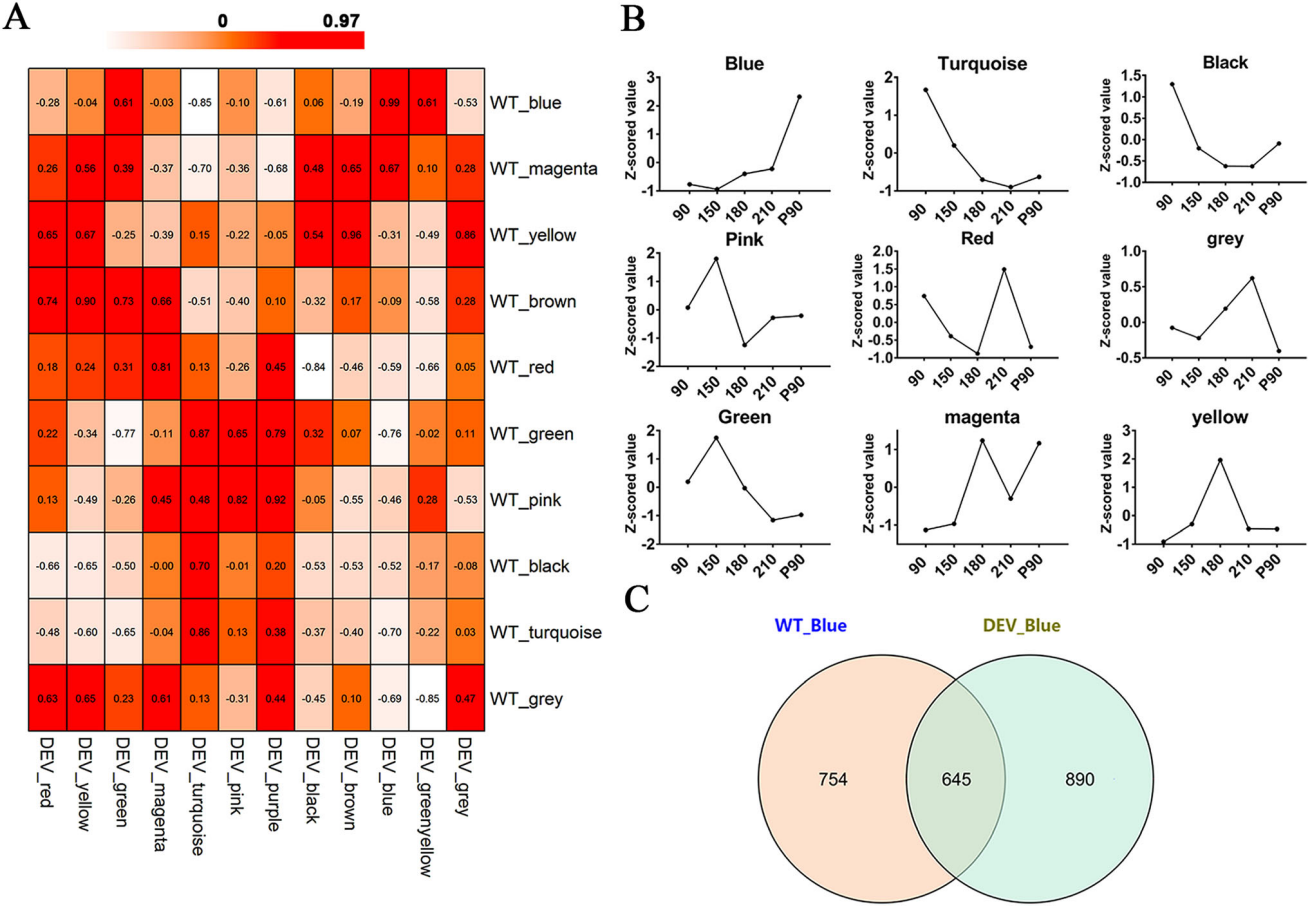
Module correlation analysis between “WT” and “DEV” transcriptomes. **A** Correlation analysis of modules in “WT” and DEV”. Numbers in the heatmap represent the PCC values. Red scales indicate high PCC values. **B** Expression patterns of hub genes in each “WT” module. Values of the characteristic vector (calculated by WGCNA) in each module were subjected to *Z*-score standardization and used to draw the line graph. **C** Overlapped genes in WT_blue and DEV_blue modules

WGCNA (weighted gene correlation network analysis) was performed on two transcriptome datasets of developmental ‘Newhall’ navel orange (covering five time points), namely “WT” and “DEV”, respectively. The “WT” transcriptome dataset was reported by our group while the “DEV” transcriptome dataset was reported by Huang et al. (2019). Samples from both datasets could be obviously separated by time points (Fig. S1). According to the algorithm of the WGCNA, genes with similar expression patterns were integrated into one module. The “WT” and “DEV” datasets were divided into 10 and 12 modules according to WGCNA analysis, respectively. The expression patterns of genes from most ‘WT’ modules could also be found within the “DEV” dataset, which could be verified by the high (over 0.80) PCC (Pearson correlation coefficient) values pairwisely calculated between the modules in “WT” and “DEV” datasets. The expression patterns of genes in the blue module showed the highest similarity between the “WT” and “DEV” datasets (PCC value > 0.99) (Fig. 2A). Besides, the hub genes in the blue module were consistently up-regulated along with fruit maturation (Fig. 2B), which is highly similar to the accumulation pattern of some functional lipids or lipid derivatives, such as wax and the senescence-related hormone JA. A total of 645 genes were characterized in both WT_blue and DEV_blue module, which were thus regarded as candidate genes with similar variation trend to the main lipid-related products in ‘Newhall’ navel orange (Fig. 2C).

Among these 645 genes, a number of key genes for lipid metabolism were characterized, especially in the wax- and JA-related pathways (Table1; Table S1). A total of 23 transcriptional factors (TFs) were identified. To identify the key TF regulating the lipid-related pathways, we compared the expression of these 23 TFs between a glossy mutant of ‘Newhall navel’ orange and its corresponding WT (Fig. S3A and B). gl-Mutant is a naturally occurring glossy mutant of ‘Newhall’ navel orange with evident changes in lipid-related metabolic pathways compared to the relative WT. For example, gl-Mutant showed remarkable decreases in the expression of genes related to wax biosynthesis and wax content (He et al. 2018) (Fig. S3A). In addition to wax, other lipid compounds or lipid derivatives, including fatty acids, phospholipids, galactolipids and JA were also evidently changed in gl-Mutant compared to WT (Wan et al. 2020). Among these 23 TFs, the expression of the TF *CsERF003* (*CsESE3*) significantly decreased in gl-Mutant at almost all developmental stages, which is consistent with the variation of key genes related to wax biosynthesis, particularly *CsCER3* (Fig. S3B). To further investigate the relationship between this ERF and lipid genes, we calculated the PCC values between *CsERF003* and the lipid-related genes in the blue module. As a result, *CsERF003* also showed high positive correlations with *CER1* and *CER3*, two key genes related to wax biosynthesis, in both transcriptome datasets. In addition, significant positive correlations were observed between *CsESE3* and almost all necessary genes for JA biosynthesis (two *PLIPs* (*PLIP1* and *PLIP3*), *LOX3*, *AOS* and three *OPRs*), with average PCC values above 0.65 in both transcriptome datasets (Table1). Therefore, it could be hypothesized that *CsERF003* might play an essential role in lipid metabolism in ‘Newhall’ navel orange.

**Table 1:** Representative lipid-related genes positively correlated with CsESE3

Gene ID	Gene Name	Cor.ESE3	KO	Annotation
Cs7g06120	*ERF003*	1.00	–	Ethylene-responsive transcription factor 3
Wax biosynthesis				
Cs1g02760	*CER1*	0.92	K15404	Protein ECERIFERUM1 /Protein WAX2
Cs4g02580	*CER3*	0.93	–	Protein ECERIFERUM3
Cs4g09520	*AT5*	0.95	–	Long-chain-alcohol O-fatty-acyltransferase family protein
Cs5g27720	*FATA*	0.83	K10782	Oleoyl-acyl carrier protein thioesterase
Cs6g12890	*FAR*	0.86	K13356	Fatty acyl-CoA reductase 2
Jasminic acid related genes				
Cs1g17210	*JAZ1(B)*	0.80	K13464	Protein TIFY 10A
Cs1g17220	*JAZ1(A)*	0.74	K13464	Protein TIFY 10A
Cs2g03240	*JAZ8*	0.80	–	Protein TIFY 5A
Cs7g02820	*JAZ10*	0.67	K13464	Protein TIFY 9
Cs2g28830	*PLIP1*	0.77	–	Alpha/beta-hydrolase domain-containing protein
orange1.1t01654	*PLIP3*	0.68	–	Alpha/beta-hydrolase domain-containing protein
Cs1g17380	*LOX3*	0.67	K00454	Linoleate 13S-lipoxygenase 3
Cs3g24230	*AOS*	0.72	K01723	Allene oxide synthase
Cs5g17900	*OPR*	0.66	K05894	Putative 12-oxophytodienoate reductase 11
Cs5g17920	*OPR*	0.67	K05894	12-oxophytodienoate reductase 2
Cs3g25140	*JMT*	0.71	–	Jasmonate O-methyltransferase
Phospholipid metabolism				
Cs3g14580	*Lipase*	0.60	–	Alpha/beta-hydrolase domain-containing protein
Cs3g23440	*Lipase*	0.80	–	Lipase
Cs7g09100	*Lipase*	0.76	–	Alpha/beta-hydrolase domain-containing protein
Cs1g12430	*FAD5*	0.77	K20416	Palmitoyl-monogalactosyldiacylglycerol delta-7 desaturase
Cs2g13110	*LPP3*	0.93	K18693	Lipid phosphate phosphatase 3
Cs2g13440	*PSS1*	0.62	K08730	Phosphatidylserine synthase 2
Cs5g11570	*DGK*	0.62	K00901	Diacylglycerol kinase

### *CsESE3* had similar sequence structure but different expression pattern compared with SHN genes

**Fig. 3:**
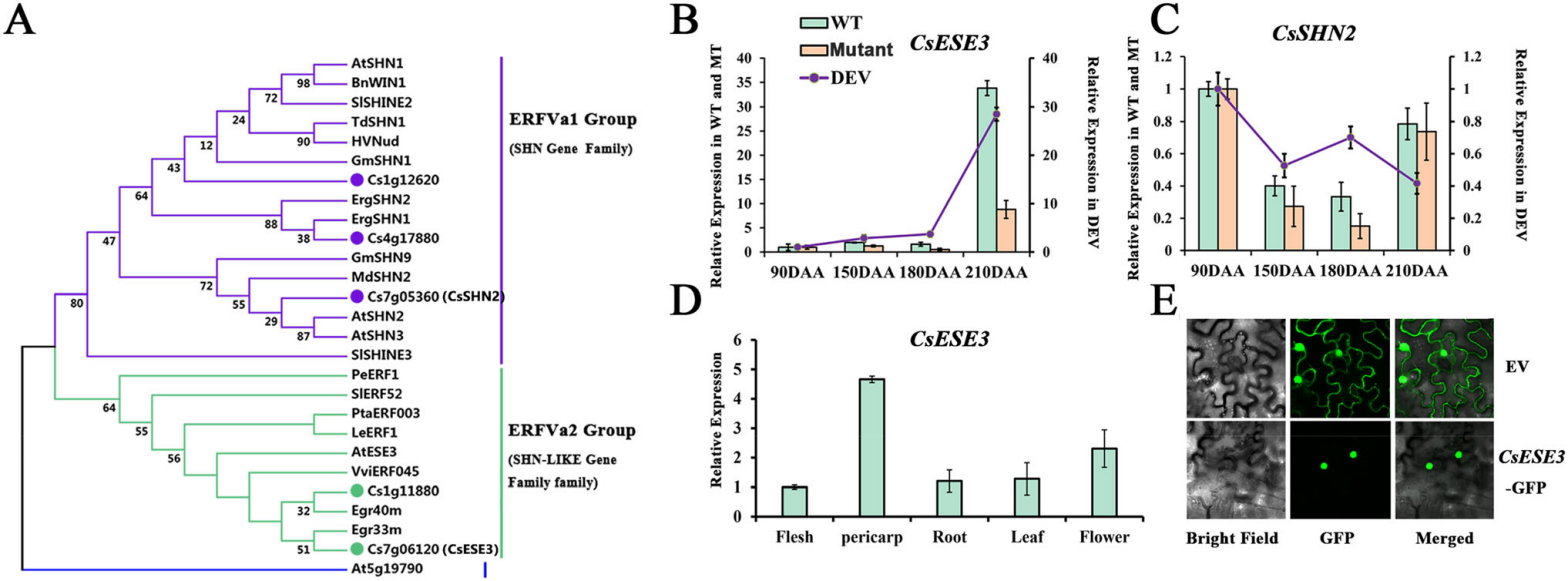
Phylogenetic analysis of ERF-V group genes and expression patterns of SHN genes in citrus. **A** Phylogenetic analysis of genes in ERF-V group of *Arabidopsis* and citrus. The green, red and blue branches represent the SHN family, the SHN-Like family, and an ERFV-b member, respectively. The genes identified in sweet orange are marked with corresponding colored circles. **B** Relative FPKM values of *CsESE3* in the three transcriptomes of “WT”, “gl-Mutant” and “DEV”. **C** Relative FPKM values of CsSHN2 in the three transcriptomes of “WT”, “gl-Mutant” and “DEV”. The left axis represents the normalized FPKM values of “WT” and “gl-Mutant”, and the right axis presents the normalized FPKM value of “DEV”. The values were normalized by FPKM values at 90 DAA. **D** Relative expression of *CsESE3* in different tissues by qRT-PCR. (E) Subcellular location of *CsESE3* and the empty vector (EV). EV was used as control

*CsERF003* (Cs7g06120; hereafter named as *CsESE3*) had a CDS length of 579 bp and showed the highest sequence similarity (64.6%) to *AtESE3* (AT5G25190) in *Arabidopsis*. *AtESE3* belongs to the ERF-Va2 sub-group, a branch of the ERF-Va. The other sub-group of ERF-Va was composed of well-known wax-regulating SHN genes. The protein sequences of ERF-Va genes from other species were extracted and used for a comprehensive identification of ERF-Va members in ‘Newhall’ navel orange. The phylogenetic analysis clearly separated the ERF-Va1 and ERF-Va2 sub-groups, which were then named as SHN Gene Family and SHN-Like Gene Family, respectively (Fig. 3A). Besides *CsESE3*, another homolog (Cs1g11880) of *AtESE3* in citrus was identified; however, the *Cs1g11880* gene was not found among the 645 genes. Thus, our further analysis would be focused only on *CsESE3.* Three SHN genes were characterized in orange, which were designated as *CsSHN1a* (Cs1212620), *CsSHN1b* (Cs4g17880) and *CsSHN2* (Cs7g05360), respectively. All members from both SHN Gene Family and SHN-Like Gene Family had the AP2 domain and CM domain. However, the SHN-Like Gene Family harbored an incomplete MM domain relative to the SHN Gene Family, which was an important feature to distinguish these two families (Fig. S2).

As mentioned above, both *SHN* genes (ERF-Va1 sub-group) and *ESE3* homologs (ERF-Va2 sub-group) have been reported to be involved in wax synthesis. To identify which sub-group genes were the main wax-regulators in citrus, we studied expression patterns of SHN homologs and *ESE3* homologs in citrus, respectively. Among the three *SHN*s, two *SHN*1 homologs, Cs1212620 and Cs4g17880, were not present in the three published transcriptomes (“WT”, “gl-Mutant” and “DEV”), and had no or low expression value (RPKM) in fruit as demonstrated in the website of orange genome (http://citrus.hzau.edu.cn/orange/index.php). Thus, we only focused on the variations of *CsSHN2* in the following analysis. As mentioned above, the expression of *CsESE3* was consistently up-regulated along with fruit maturation, and was significantly lower in gl-Mutant than in WT (Fig. 3B). However, none of these variation trends was observed for *CsSHN2* in the three transcriptomes. *CsSHN2* exhibited the highest expression level at 90 DAA (days after anthesis) and its FPKM value was comparable in gl-Mutant and WT, particularly at 210 DAA (Fig. 3C). The highest expression of *CsESE3* was found in pericarp in mature fruits, which was the main tissue for wax biosynthesis. The second highest expression was observed in flower, followed by leaf, root and flesh (Fig. 3D). Like the subcellular localization of SHN TFs*, CsESE3* was also located in cell nucleus (Fig. 3E). Therefore, it could be concluded that the expression pattern of *CsESE3* instead of *CsSHNs* is consistent with the variation of wax accumulation in ‘Newhall’ navel orange fruit.

### Overexpression of *CsESE3* activated the pathways of JA biosynthesis in citrus callus and fruit

**Fig. 4:**
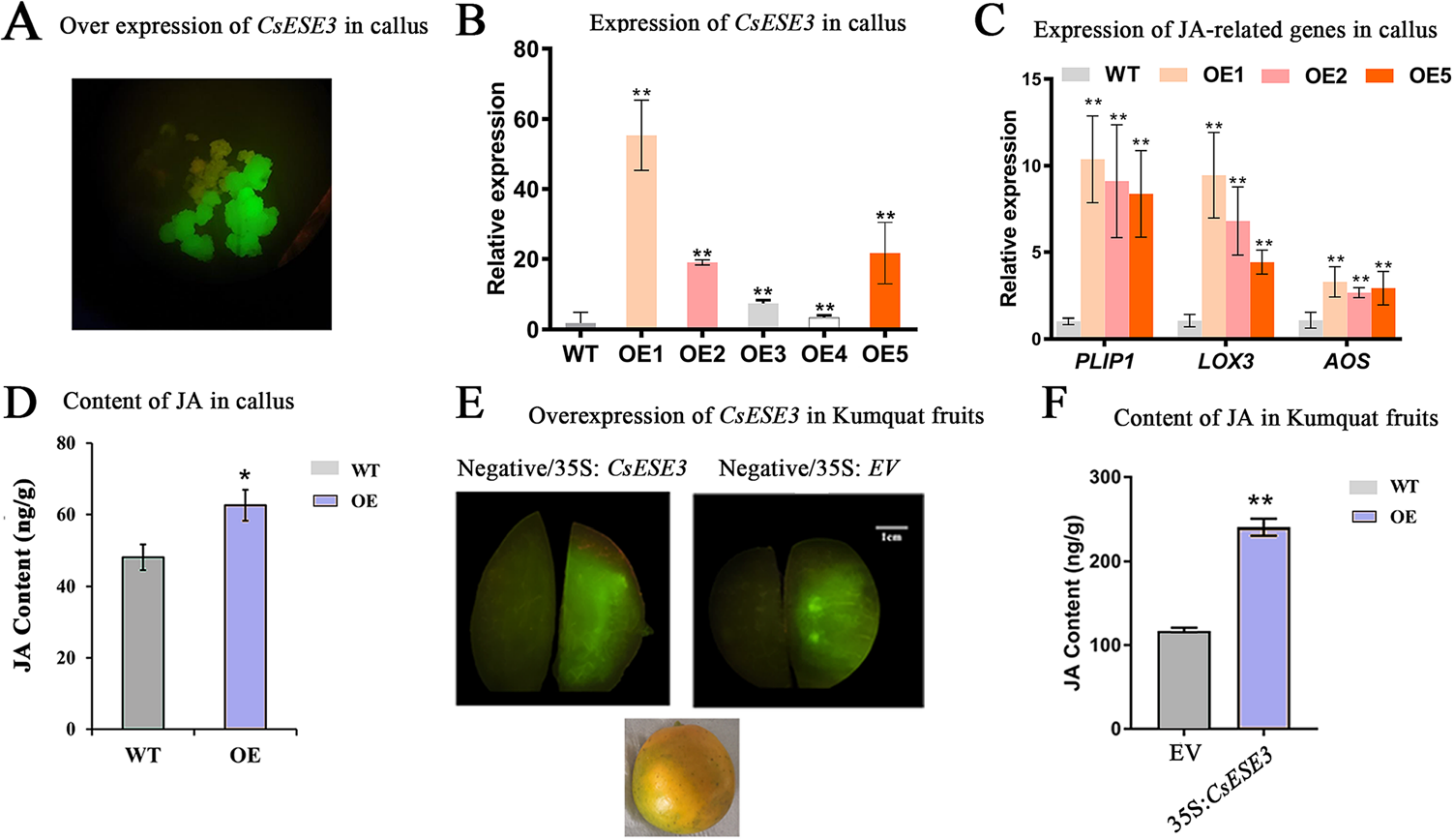
Overexpression of *CsESE3* in citrus callus and fruit. **A** GFP fluorescence in callus with *CsESE3* overexpression. **B** Relative expression level of *CsESE3* in three transgenic lines compared with that in WT. **C** Significant variations of JA biosynthesis genes in *CsESE3* overexpression callus. **D** JA content in *CsESE3* overexpressing callus and WT callus. Samples from OE1, OE2 and OE5 were mixed for JA analysis. **E** Comparison of GFP fluorescence in *CsESE3* and EV *Agrobacterium* injection areas and non-injection areas. The left part in each figure is non-injection areas (Negative) while the right part is injection areas. **F** Comparison of JA content between injection area of *CsESE3* and EV. * and * * mean that the *t* test *P* value is lower than 0.05 and 0.01, respectively, *n* = 3

The co-expression of *CsESE3* with JA-related genes suggested a potential regulatory role of this gene in JA biosynthesis. To investigate the potential roles of *CsESE3* in JA biosynthesis, we stably transformed it into citrus callus. Five transgenic lines with strong GFP fluorescence were selected to verify the expression of *CsESE3*, all of which displayed higher expression levels of *CsESE3* than the WT (Fig. 4A, B). Samples from the three lines with the highest expression, including OE1 (55.34-fold increase), OE2 (19.02-fold increase) and OE5 (21.67-fold increase), were used for further analysis. We firstly focused on the variation of members from the PLIP and DAD1 families, two JA-initiating lipase gene families. The expression of *CsPLIP1* showed a strong positive correlation with that of *CsESE3*, which was increased by up to 4.0 folds in the three OE lines. However, no significant differences were observed in the expression levels of *CsDAD1* and *CsDAll2,* both of which have been functionally characterized to initiate JA biosynthesis previously (Wan et al. 2020). These results indicated that *CsESE3* might initiate JA biosynthesis through lipases independent of the DAD1 gene family. In the downstream JA biosynthesis genes, we also observed significant increases in the expression levels of *CsLOX3* and *CsAOS*, respectively (Fig. 4C). At the metabolite level, we found that JA was highly accumulated in OE lines and the JA content was (48.16 ± 3.60) and (62.59 ± 4.33) ng/g in the WT and OE lines, respectively (Fig. 4D). To further verify the regulation of JA biosynthesis in citrus fruit, we transiently overexpressed *CsESE3* in the fruit of Kumquat (*Fortunella crassifolia* Swingle). After five days, strong GFP fluorescence was detected at the inner side of the peel, suggesting the overexpression of EV and *CsESE3* (Fig. 4E). The expression of several key wax-related genes, including *CsLACS1*, *CsCER3* and *CsCER1-3* were significantly increased in flesh with *CsESE3* overexpression compared to the control (Supplementary Fig. S4). For JA, the content of JA in the OE lines (240.18 ng/g) was more than twice that of the control with the empty vector (117.08 ng/g) (Fig. 4F). Taken together, *CsESE3* could positively modulate JA accumulation in citrus fruit.

### *CsESE3* affected the morphological and physiological traits of transgenic tomato lines

**Fig. 5:**
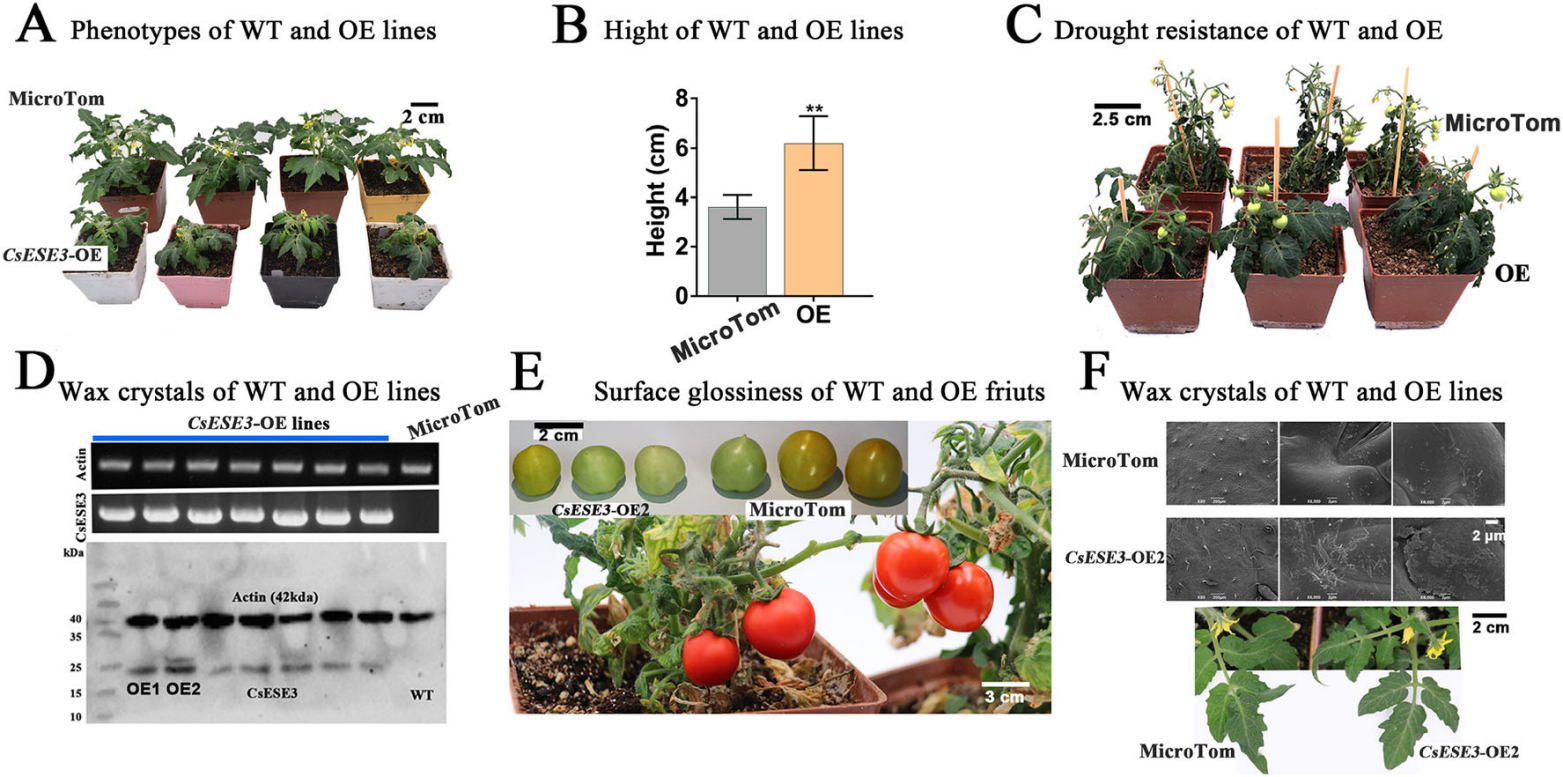
Morphological and physiological traits of *CsESE3* transgenic tomato. **A** Phenotypes of MicroTom and OE lines. **B** Height of MicroTom and *CsESE3* transgenic lines. * * mean that the *t* test *P* value is lower than 0.01. **C** Comparison of drought tolerance between *CsESE3* transgenic tomato and MicroTom. **D** Western-blot verification of transgenic tomato lines overexpressing *CsESE3*. **E** Comparison of fruit surface glossiness between MicroTom and *CsESE3* transgenic line (OE2, T1 generation). **F** Leaf morphology and SEM observation of wax crystals on MicroTom and *CsESE3* transgenic line (OE2, T1 generation)

To verify co-regulation of wax and JA biosynthesis by *CsESE3*, we ectopically overexpressed *CsESE3* in ‘Micro-Tom’ tomato, which has been widely used for functional characterization of wax- and JA-related genes (Leide et al. 2007; Li et al. 2003; Martin et al. 2017; Yu et al. 2013). Overexpression of *CsESE3* resulted in an evident dwarf phenotype and significantly longer trichrome of OE lines compared with the MicroTom (Fig. 5A, B, Fig. S5). The variation in wax content might also affect the drought resistance of plants. Watering was stopped on both MicroTom and OE lines for five days. As a result, the leaves of MicroTom all wilted and the whole plant could hardly maintain a normal morphology; while in the OE lines, the leaves were still extending and a normal morphology of the plant could be maintained (Fig. 5C). Seven transgenic lines with the sequence of *CsESE3* were selected to verify the expression of CsESE3 protein. CsESE3 protein was detected in all the seven lines. However, OE1 and OE2 lines had evidently higher protein levels than other lines, which were therefore used as the T1 generation for further analysis (Fig. 5D). Alteration of wax content often changes fruit glossiness in tomato (Petit et al. 2014). We observed that the OE2 line had rougher fruit surface and redder fruit skin as well as thicker and darker green leaves compared with the MicroTom (Fig. 5E). Scanning electron microscopy (SEM) observation on the leaves revealed that the OE2 line had longer trichomes on the fruit surface than the MicroTom. In addition, the leaf surface of the OE line had a deposition of more wax crystals (Fig. 5F).

### Contents of wax, JA were altered in *CsESE3* tomato OE lines

**Fig. 6:**
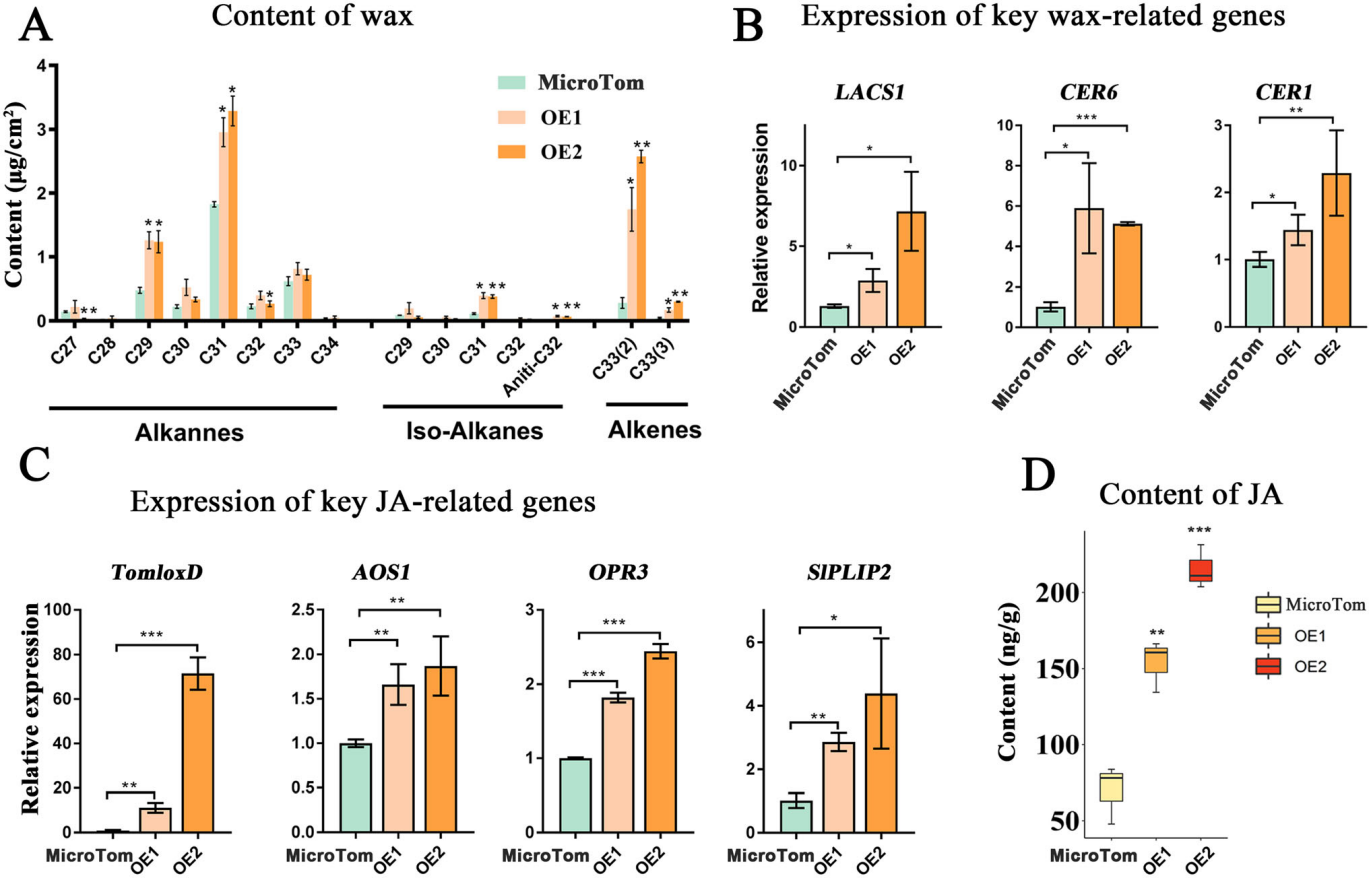
Variations of wax and JA content in *CsESE3* transgenic tomato lines relative to the MicroTom. **A** Comparison of fruit wax between *CsESE3* transgenic tomato and MicroTom. **B** Expression of key wax genes in transgenic lines compared with the MicroTom. **C** Expression of representative JA genes in transgenic lines compared with the MicroTom. **D** Content of JA in transgenic lines compared with the MicroTom. *, ** and *** mean that the *t* *t*est *P* value is lower than 0.05, 0.01, and 0.001, respectively. Primers used for qRT-PCR are listed in Supplementary Table S4

Alkanes are dominant aliphatic components in tomato wax, particularly C31 alkane, followed by the C29 alkane. Different from that of other plants, the composition of tomato wax is characterized by the presence of branched alkanes and alkenes. Among all aliphatic components of wax, we identified eight straight chain alkanes, five branched alkanes (iso-alkanes) and two alkenes (Fig. S6). The C27 alkane was decreased by 77.8% in the OE2 line, but the two dominant C29 and C31 alkanes were both highly accumulated in the two OE lines. The content of C29 alkane in MicroTom, OE1 and OE2 lines was 0.48, 1.26 and 1.24 μg/cm^2^, and that of C31 alkane was 1.83, 3.47 and 4.73 μg/cm^2^, respectively. Consistently, evidently higher levels of Iso-alkane (C31) were observed in the two OE lines. The two alkenes, 33 alkane (2) and 33 alkene (3), were at lower levels in MicroTom (0.28 and 0.047 ug/cm^2^, respectively), but were significantly induced in the two OE lines, with the former being increased by 6.16 and 9.08 folds and the latter by 3.72 and 6.53 folds in OE1 and OE2 lines, respectively (Fig. 6A). At the transcriptional level, the expression of the *SlLACS1* gene, whose product provides most of the long-chain acyl-CoAs for wax biosynthesis, was increased by 2.79 and 5.90 folds in OE1 and OE2, respectively. Similar increases were also observed in *SlCER6* and *SlCER1*, other two indispensable genes for wax biosynthesis (Fig. 6B). These results revealed that *CsESE3* promoted wax accumulation in OE lines. As a key gene associated with the biosynthesis of JA, *TomloxD* was induced by 9.45 and 60.50 folds in OE1 and OE2 lines, respectively. Among the genes encoding the subsequent steps of JA biosynthesis in tomato, we also observed significant up-regulation of *SlAOS* and *SlOPR3* (Fig. 6C). In addition, we measured the expression of two PLIP homologs in tomato, namely *SlPLIP1* (Solyc01g095720, 51.85% similarity to *AtPLIP1*) and *SlPLIP2* (Solyc01g079600, 53.71% similarity to *AtPLIP2*). We found *SlPLIP2* were significantly induced by 2.80 and 4.30-fold in OE1 and OE2, respectively (Fig. 6C). Next, we measured the JA content and observed 1.54- and 5.83-fold increases of JA in the OE1 and OE2 lines, respectively (Fig. 6D).

Previous findings have indicated that both growth conditions and trichome length might be closely associated with JA content (Chen et al. 2016; Qi et al. 2011). The increase in JA content might result in longer trichomes and further change the metabolite composition of the OE lines, since a number of metabolites such as flavonoids are specifically synthesized in surface trichomes (Chen et al. 2016; Qi et al. 2011; Zager and Lange 2018). Samples used for non-targeted metabolic analysis could be distinctly separated into the MicroTom and OE lines (Fig. S7A). Most of the flavonoid species, including the precursor naringeninchalcone, naringenin, hesperetin, eriodictyol and modified flavonoids, had high levels of accumulation in the two OE lines (Fig. S7B). 12-oxo phytodienoic acid (OPDA), the precursor for JA biosynthesis, and three compounds annotated as OPDA were evidently increased in the two OE lines, especially in OE2, whose accumulation pattern of OPDA was consistent with that of JA (Fig. S7C).

### *CsESE3* directly activated the expression of *CsPLIP1*

**Fig. 7:**
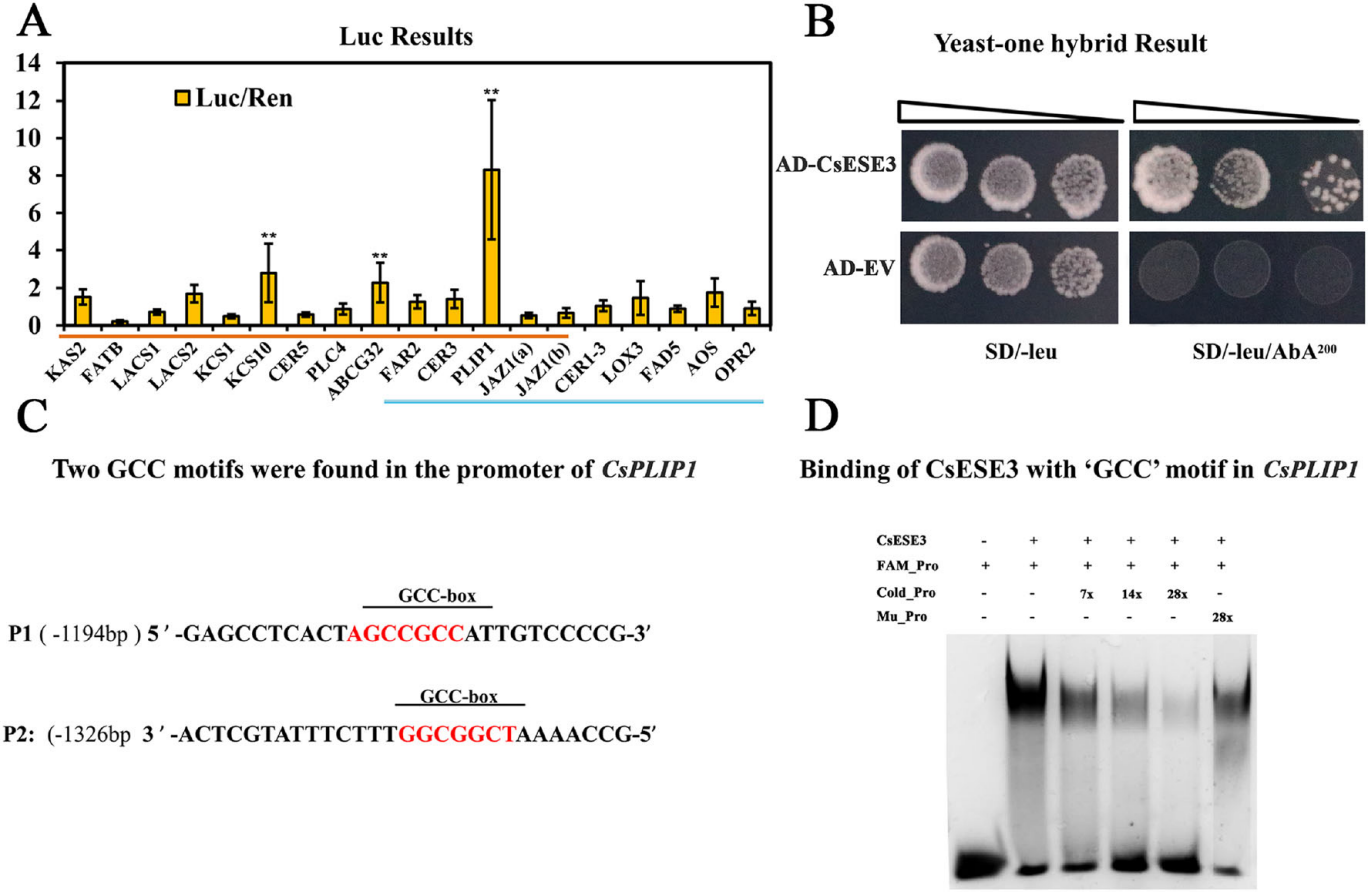
Activation of the CsPLIP1 promoter by *CsESE3*. **A** Dual-luciferase assay to verify the binding activity of *CsESE3* with lipid-related gene promoters. Genes with *red* underlines were selected from the comparison of WT and gl-Mutant, all of which were significantly down-regulated in gl-Mutant. Genes with *blue* underlines were selected from the WT_Blue module, all of which were significantly positively correlated with *CsESE3*. The bar value is expressed as the Luc/Ren ratio. ** means that the *t t*est *P* value is lower than 0.01, *n* = 6. **B** Yeast one-hybrid assay to verify the binding activity of *CsESE3* with CsPLIP1 promoter. AD_EV (empty vector) was used as the negative control. Transformed yeast cells were dotted at 10^–1^, 10^–2^ and 10^–3^ dilutions on the selective medium. SD-Leu/AbA^200^, SD medium without Leu supplemented with 200 ng/mL AbA. **C** Identification of GCC-box sites in the promoter of CsPLIP1. One site was located at about 1.1 kb away from CsPLIP in the sense strand, and the other was located at about 1.3 kb away from CsPLIP in the anti-sense strand. **D** EMSA assay to reveal the direct binding of *CsESE3* to GCC-box. ‘+’ or ‘−’ indicates the presence or absence of corresponding probes or proteins. ‘7×’, ‘14×’ or ‘28×’ means 7-fold, 14-fold or 28-fold excess of unlabeled or mutated probes, respectively

The obvious increase in both wax and JA content in *CsESE3* transgenic plants indicated that *CsESE3* might directly modulate genes in both wax and JA biosynthetic pathways. The promoters of 19 important genes from these two pathways were selected to verify their interactions with *CsESE3* by Dual Luciferase (Luc) experiment. These 19 genes for test were selected according to either of the following two criteria: showing a similarly decreasing trend to *CsESE3* in gl-Mutant relative to the WT, and being co-expressed with *CsESE3* in the developmental stages of ‘Newhall’ navel orange (present in the 645 overlapping genes). A *35S:CsESE3* construct and an empty vector were used as effectors to be fused with the promoters of these 19 genes for Luc experiments, respectively. The relative Luc activity of the JA-initiating lipase *CsPLILP1* was strongly stimulated by *CsESE3* (more than 7.30 fold), and two wax-related genes, *CsKCS10* and *CsABCG32*, were also evidently induced by 2.55 and 2.28 folds, respectively (Fig. 7A). However, Yeast one-hybrid assay (Y1H) confirmed that *CsESE3* could only bind to the promoter of *CsPLIP1* (Fig. 7B). Two ‘GCC’ (AGCCGCC) motifs were identified in the region about 1.19 and 1.33 kb away from *CsPLIP1* (Fig. 7C). We then purified the CsESE3 protein by His Ni–NTA agarose and performed electrophoretic mobility shift assay (EMSA). A band shift was observed when the purified CsESE3 protein was mixed with the labeled probe from *PLIP1*, and the binding of the CsESE3 protein to the labeled probe was attenuated by supplement of an excessive amount of cold probes (unlabeled probes) rather than by mutant probes (Fig. 7D). These results suggested that CsESE3 protein could bind to the promoter of *CsPLIP1* and activate its activity.

### *CsESE3* could not activate the promoters of three DAD1 family genes

Members in both PLIP and DAD1 gene families are involved in the hydrolysis of chloroplast membrane lipids to initiate JA biosynthesis (Ryu 2004; Stintzi 2000; Wang et al. 2017). We totally characterized seven DAD1 family genes, two PLIP genes and four other lipases in citrus genome (Fig. S8A). Similar to *AtPLIP1*, *CsPLIP1* was also localized at the chloroplasts and could significantly increase the JA content in citrus fruit (Fig. S8B–D). We identified one GCC-box (1.0 kb away from ATG) in the promoters of *CsPLIP_Like*, but not in the promoters of *CsDAD1* and *CsDALL2*. Luc experiment results showed that *CsESE3* could evidently increase the activity of the promoter of *CsPLIP_Like* by over two folds, but not the activity of the two DAD1 genes (Fig. S8E). EMSA assay further revealed that *CsESE3* activated *CsPLIP_Like* by binding to the GCC-box in the promoter (Fig. S8F and G).

## Discussion

Both wax and JA are lipid derivatives playing essential roles in the response of plants to external environmental stresses. They have similar accumulation patterns in citrus according to previous reports, both of which were highly accumulated at fruit mature stage (Wan et al. 2020, 2021; Wang et al. 2016). The key genes in these two biosynthetic pathways were also found to have the same expression pattern in the two fruit transcriptomes of ‘Newhall’ navel orange (“WT” and “DEV”) (Fig. 2A). These results suggest that biosynthesis of wax and JA are tightly connected. Simultaneous induction of wax and JA biosynthesis has also been reported in other circumstances, including cherry under ABA treatments and tea under abiotic attack, etc. (Gutiérrez et al. 2021; Zhao et al. 2020). In this study, we found *CsESE3* was co-expressed with the key genes in wax and JA biosynthetic pathways (Table 1), and was differently expressed in the glossy mutant of ‘Newhall’ navel orange with altered lipid metabolic pathways compared to the relative WT (Fig.S2). These results suggest *CsESE3* might be involved in the co-regulation of both wax and JA biosynthesis. *CsESE3* showed high sequence similarity with SHN TFs and both of them could be classified into the ERF-Va family (ERF-Va1 for *SHN*s and ERF-Va2 for *ESE3* homologs, respectively) (Fig. 3A). As mentioned in the introduction part, SHN TFs are widely reported in wax formation in plants. However, in this study, only *CsESE3* showed consistent variations with wax either in different materials (WT *vs* gl-Mutant) or across developmental stages in ‘Newhall’ navel orange (Fig. 2B, C). Therefore, it can be speculated that *CsESE3*, rather than the three SHN homologs play more important roles in wax formation in citrus. Similar results were found in phalaenopsis and grapes, where *ESE3* homologs, rather than *SHN*s were reported to regulate wax biosynthesis (Lai et al. 2019; Leida et al. 2016). Thus, we inferred that some members from the ERF-Va2 group could also regulate wax biosynthesis, which may possess overlapping functions with *SHN*s. Recently, a study reported that inhibition of *GhSHN2* in cotton resulted in decreases in content of both wax and JA at the same time (Li et al. 2019a). Thus, it’s reasonable to infer that *CsESE3* might also play important roles in the modulation of both wax and JA.

### Modulation of wax biosynthesis by *CsESE3*

Here we verified the induction effect of *CsESE3* on wax biosynthesis in tomato. Content of wax was highly accumulated and expression of key wax-related genes, including *CER1* and *CER6* were significantly induced, in *CsESE3*-OE lines (Fig. 6A, B). Similarly, activation of wax biosynthesis by *PeERF1* (the homolog of *AtESE3*) has also been reported. The time point for the high expression of *PeERF1* in Phalaenopsis was highly coincident with the time point for the active biosynthesis of cuticle wax in flowers, and heterologous overexpression of *PeERF1* in *Arabidopsis* led to higher amount of wax compared to the WT (Lai et al. 2019). Typically, alteration of wax content would evidently change plant surface glossiness. *CsESE3* was down-regulated in the wax-deficient gl-Mutant in our previous study. The significant down-regulation of *CsESE3* in gl-Mutant was associated with a significant decrease in wax load and glossier fruit surface than WT (Fig. S3A), while overexpression of *CsESE3* in tomato resulted in a higher wax content and rougher fruit surface (Fig. 5E). The rough surface is similar to the phenotype of the ‘Dull mutants’ in tomato. These ‘Dull mutants’ were characterized by a significant reduction of fruit brightness and most of them showed increases in wax content relative to WT (Petit et al. 2014). In addition, the higher wax load and rougher fruit surface of *CsESE3*-OE lines, are in sharp contrast with the lower wax load and glossier fruit surface of the *SlSHN3*-silenced tomato lines (Shi et al. 2013). As a result, alteration of fruit surface glossiness is another key proof for the induction of wax biosynthesis mediated by *CsESE3*.

Although *CsCER3* and *CsCER1-3* were significantly co-expressed with *CsESE3*, Luc experiments showed that *CsESE3* could not activate their promoters. Similarly, in the *Arabidopsis* lines with SHN overexpression or silencing, *AtSHN1* was co-expressed with *AtCER1* and *AtCER3*, but could not interact with their promoters (Aharoni et al. 2004; Kannangara et al. 2007). Thus, *CsESE3* may regulate the biosynthesis of wax aldehydes and alkanes independent of these two key genes in citrus.

### Modulation of JA biosynthesis by *CsESE3*

*CsESE3* was co-expressed with almost all necessary JA biosynthetic genes in WGCNA analysis (Table 1), and the induction effect of *CsESE3* on JA biosynthesis was observed in tomato, transgenic citrus callus and citrus fruit, respectively. Similar results were reported for other ERF members. For example, *SlERF15/16*, *OsEIL1* and *AtORA47* all could increase contents of JA as well as expression levels of JA-related genes in tomato, rice, and *Arabidopsis,* respectively (Chen et al. 2016; Hu et al. 2021; Ma et al. 2020). JA could help to allocate carbon source for plant growth and plant resistance, and JA treatment could lead to lower division and elongation rate of the leaf cells compared with the control (Attaran et al. 2014), which could well explain the dwarf phenotype of the *CsESE3*-OE tomato lines (Fig. 5A, B). Similar results were obtained in *Arabidopsis* lines overexpressing *ORA47*, *PLIP2* and *PLIP3*: the severely retarded growth was accompanied by elevated levels of JA (Chen et al. 2016; Wang et al. 2018). In addition, the JA signaling pathway has been reported to have great impacts on the development of tomato trichomes (Qi et al. 2011), which could well explain the longer length of trichomes and higher amount of flavonoids (mainly biosynthesized in trichomes) in OE lines (Fig. S7). In summary, these results clearly show that *CsESE3* is able to activate JA biosynthesis.

Chloroplast-located lipase is the link between chloroplast lipids and JA. Till now, members from PLIP and DAD1 lipase gene family are reported to initiate JA biosynthesis through hydrolyzation of chloroplast lipids (Hyun et al. 2008; Rudus et al. 2014; Seo et al. 2009; Wang et al. 2017, 2018). Similarly, *CsDAD1*, *CsDALL2* (two DAD1 family members) and *CsPLIP1* all could initiate JA biosynthesis in citrus (Fig. S8D) (Wan et al. 2020). However, only *CsPLIP1* was co-expressed with those key JA biosynthesis genes and *CsESE3* in ‘WT’ and ‘DEV’ transcriptomes, and no *DAD1* genes were found in this study (Table 1). Similar results were found in transgenic callus lines: *CsESE3* could evidently induce expression of *CsPLIP1* and several key JA-related genes (Fig. 6C), but little variations were observed for expression of *CsDAD1* and *CsDALL2*. Furthermore, we verified that *CsESE3* could active promoters of two PLIP genes but had no effect on promoters of DAD1 genes (Fig. S8E). These results demonstrate that *CsESE3* is more closely associated with the PLIP-initiated JA biosynthesis routes rather than the DAD1-initiated routes. In the Luc experiments, *CsPLIP1* was much more significantly induced than other lipid genes (Fig. 7A). The EMSA results showed that *CsESE3* binds to the GCC-box (one of the ERF binding motifs) in the promoters of *CsPLIP1* and *CsPLIP-Like* (Fig. 7D and Fig. S8G). Similar results were reported recently in tomato: two ERF TFs, *SlERF15* and *SlERF16*, could bind to the ERF binding motifs (CCG(A/T)CC) in the promoters of *TomLOXD, AOC* and *OPR3* to activate JA biosynthesis (Hu et al. 2021).There results indicated that *CsESE3* could regulate JA biosynthesis in ‘Newhall’ navel orange through the GCC-box binding site.

In summary, *CsESE3* affects both wax and JA biosynthesis, and thus may be a node connecting wax and JA biosynthesis at the transcriptional level. We proposed a working model of *CsESE3* in activation of wax and JA biosynthesis (Fig. S9). Both wax and JA are critical to the defense of plants against various external environmental stresses. Therefore, our findings would lay a foundation for developing efficient strategies for plant defense and breeding stress-resistant cultivars.

## Experimental procedures

### RNA extraction and bioinformatics analysis

#### RNA extraction and qRT-PCR validation

RNA extraction from the flavedo was performed according to the previously reported method (Wan et al. 2020). The qRT-PCR experiment was performed according to Lu et al. (2018) with minor modifications. First-strand cDNA was synthesized using the HiScript II First Strand cDNA Synthesis Kit (+ gDNA wiper; Vazyme). gDNA wiper is included in the Kit to wipe DNA fragments. The qRT-PCR was performed with the Roche LightCycler 480 system using the 23 LightCycler 480 SYBR Green master mix (Roche) and a three-step program: preincubation at 95 °C for 10 min; 40 cycles of amplification at 95 °C for 10 s, 60 °C for 10 s, and 72 °C for 20 s; followed by a melting curve at 95 °C for 5 s, 65 °C for 1 min, and then ramping at 0.11 °C/s to 97 °C with continuous fluorescence measurement. Gene-specific primers used for qRT-PCR were designed using Primer Express software. The citrus actin gene was chosen as the endogenous control, which was the same as previously reported (Huang et al. 2018). Fluorescence was measured at each extension step. Each run contained a negative control (water in place of cDNA), and each reaction was performed in triplicate. The reaction specificity was confirmed by the negative control and a melting temperature calling analysis. The data were analyzed using LightCycler 480 software release 1.5.0 (Roche). Three replicates were set for each gene quantification and the results were calculated with the 2^−ΔΔCt^ method. The primers are listed in Table S4.

#### WGCNA (weighted gene correlation network analysis) and differential gene expression analysis

Two sets of transcriptomic data on five time points of ‘Newhall’ navel orange, namely “WT” and “DEV”, were used for WGCNA analysis. The transcriptome “WT” contained five time points, and the data of four time points (90 DAA and 210 DAA) have been published in our previous study (Wan et al. 2020, 2021). The datasets are presented in Table S2. Transcriptome ‘DEV’ was downloaded from the published data by Huang et al. (2019). Genes with high correlation values (value > 0.8) were grouped into the same module according to the algorithm of WGCNA R package. WGCNA algorithm was firstly performed on ‘WT’ and ‘DEV’ datasets separately to obtain the feature vector values of each module. The module correlations between ‘WT’ and ‘DEV’ were obtained by calculating the correlation values of the module feature vectors.

#### Gene sequence analysis and construction of phylogenetic tree

The protein sequences of reported ERF-V family members were downloaded from NCBI or Uniprot website. Multiple sequence alignment was performed using the MUSC program of MEGA (version 7.0.14) software, and JTT + G was found to be the best model by model prediction. A phylogenetic tree was constructed using the maximum likelihood method and 1000 bootstrap resampling. Gene IDs used to generate the phylogenetic tree were provided in the “Accession Number” part.

Sequences of DAD1 and PLIP phospholipase family genes were downloaded from TAIR website (https://www.arabidopsis.org/). The IDs of reported phospholipase genes and construction of phylogenetic tree could refer to our previously published paper (Wan et al. 2020).

### Plant transformation and overexpression

#### Transformation of citrus callus and ‘Micro-Tom’ tomato

Embryogenic callus of sweet orange (*Citrus sinensis Osb. cv. Valencia*) was selected for transformation according the previous method (Cao et al. 2012). Briefly, the *Agrobacterium* strain GV3101 containing the overexpression vector pH7WG2d-CsESE3 (*35S:CsESE3* with a non-fused GFP tag, hygromycin resistance) was transformed into the citrus callus. Vigorous calli (previously cultured for 20 days) were dispersed in liquid culture medium for 4 d and then used for infiltration. The resulting calli were cultured on Murashige and Skoog medium in a growth room with 16 h/8 h light/dark conditions and sub-cultured every two weeks until the emergence of new transformed callus. The positive transgenic calli were selected by UV-light scanning and transformed into new growth medium. Both wild type and positive calli were harvested for compartment analysis.

The MicroTom tomato transformation was performed according to the protocol of Sun et al. (2006) and Gong et al. (2021a) with minor modifications by Bee Lynn Chew and Yu Pan (University of Nottingham). *Agrobacterium* strains with the overexpression vector pK7WG2d-CsESE3 (*35S:CsESE3* with a non-fused GFP tag; Kana resistance, Invitrogen) were used for transformation. Seedlings rooted on the selection medium were transplanted into soil with WT lines. Two independent lines with higher protein levels of *CsESE3*, OE1 and OE2, were selected and the subsequent measurements were carried out in the T1 generation.

#### Citrus fruit transient transformation and subcellular localization

Transient overexpression of genes in citrus fruit was performed as described by Gong et al. (2021a) with modifications. The full-length sequences of *CsESE3* and *CsPLIP1* without the stop codon were fused to the vectors pH7LIC6.0 (35S promoter with C terminal fusion of GFP), respectively (Li et al. 2019b). GFP was used as a reporter for the selection of positive (gene expression) area. GV3101 containing fused vectors were shaken to saturation and then transferred to fresh induction medium (to 0.4% final concentration) to shake for 14–16 h (28 °C; 220 rpm). GV3101 containing target genes were suspended in the infiltration buffer (0.05 M MES, 2 mM Na_3_PO_4_, 0.5% (m/v) D-glucose, and 0.1 mM acetylsyringone). For better transformation efficiency of the genes, 0.1% Silwet L-77 was added into the infiltration buffer according to Zhang et al. (2020). The final OD_600_ of GV3101 strains containing the constructed vector or p19 was adjusted to 0.8 and then the strains were mixed at equal volumes. Fresh mature fruit of Kumquat (*Fortunella crassifolia* Swingle) were used for infiltration. The positive (containing gene construct) and negative (containing the empty vector) control *Agrobacterium* strains were separately injected on two opposite sides of the same fruit. The injected fruit were then placed in dark for 24 h (Zhang et al. 2020). Five days later, the injection part with GFP fluoresce was harvested and frozen in liquid nitrogen for later analysis.

Subcellular localization assay was performed on *Nicotiana benthamiana* rosette leaves according to the method previously reported (Wan et al. 2020). The CDS of *CsPLIP1* without stop codon was fused into the vector pH7LIC6.0. The infiltration process was almost the same as mentioned above, except that the final OD_600_ of GV3101 strains was adjusted to 0.2. The florescence images were captured with a confocal laser-scanning microscope. The parameters were the same as those in the previous report (Wan et al. 2020).

### Validation of transcription regulation

#### Dual-luciferase transactivation assay

Promoter sequences were integrated into the pGreen0800-LUC vector to form reporter constructs (reporter constructs). The recombinant constructs were transformed into GV3101 containing pSoup assistant plasmid. The full-length CDS of *CsESE3* was homologously recombined into the pK7WG2d Gateway vector to generate pK7WG2d-CsESE3 construct, and the pK7WG2d-empty vector was used as the negative control. Both constructs were separately transformed in GV3101. The effector *Agrobacterium* (CsESE3 or EV) and reporter *Agrobacterium* were mixed at a ratio of 5:1, and then used for infiltration of *N. benthamiana* leaves. The luciferase assay was conducted following the instructions Dual-Luciferase Reporter kit (Promega). The activities of firefly luciferase (LUC) and renilla luciferase (REN) were measured after 2.5–3 d of infection. The resulting value was calculated by LUC/REN ratio. Each group contained at least six replicates. Primers used for amplification of promoters are listed in Table S4.

#### Yeast one hybrid (Y1H) analysis

Y1H screening was performed according to the Matchmaker Gold Yeast One-Hybrid Library Screening System (Clotech). The promoter of *CsPLIP1* was cloned into the pAbAi vector to generate the bait construct pAbAi-CsPLIP1. The construct was then digested by BstBI and integrated into the Y1H Gold yeast strain (*Saccharomyces cerevisiae*) genome to generate reporter strains. Auto-activation of reporter strains was tested on SD/-Ura plates with different concentrations of AbA (aureobasidin A) at 30 °C for 72 h. No auto-activation was observed. The full-length CDS of *CsESE3* was fused to the pGADT7 vector to generate a prey construct (AD_CsESE3). The prey vector and the relative empty vector (AD_EV) were transformed into yeast cells with construct pAbAi-CsPLIP1 mentioned above. The transformed yeast cells were grown on the SD-Leu plates at 30 °C for more than 3 days. The positive colonies were diluted into different concentrations and dotted on SD-Leu plates supplemented with AbA. Yeast cells containing AD_EV were used as negative controls. Primers used are listed in Table S4.

#### Purification of *CsESE3* protein and electrophoretic mobility shift assay (EMSA)

The CDS of *CsESE3* was merged into the pETDuet vector to generate pETDuet- CsESE3 vector with an N-terminal His-Tag. The construct was then introduced into the *Escherichia coli* strain BL21 and Rosetta (DE3), and protein expression was induced by 1 mM IPTG. The CsESE3 protein was purified using Ni–NTA His Bind Resin following the manufacturer’s instructions (Sangon Biotech, China). About 33-bp promoter fragments containing the predicted binding sites from gene *CsPLIP1* and *CsPLIP_Like* were 5’ FAM-labeled (GenScript, China). 100 μM of unlabeled probes (the same or mutated oligonucleotides) were excessively supplied as cold competitors or mutated competitors. Detailed oligonucleotide information is listed in Table S4. The binding reaction of CsESE3 protein and probes was performed according to the method of Gong et al. (2021b). Cold competitor probes were added at a maximum of 28-fold excess.

### Metabolite profiling

#### Measurement of tomato wax

Measurement of wax was conducted according to previous reports with modifications (He et al. 2018; Wang et al. 2014). Briefly, a group of at least five intact fruits were immersed into chloroform with 100 μL (1 μg/μL) n-tetracosane (internal standard) for 2 min twice. After drying by nitrogen (N_2_), the extracts were derivatized by pyridine and bis-*N*, *N*-(trimethylsilyl) trifluoroacetamide (BSTFA) containing 1% trimethylchlorosilane (TMCS) (Sigma) for 30 min at 50 °C and 40 min at 60 °C, respectively. After derivation, the mixture was dried again by gentle N_2_ and re-dissolved into chloroform. Qualification and quantification of wax were conducted by GC–MS (Thermo Fisher, ISQII, USA) with the Agilent DB-1 column (30 m × 25 μm i.d. × 0.1 μm). The instrument parameter settings followed previous reports (Wang et al. 2014). The content of each wax component was calculated according to the content of internal standard.

#### Measurement of JA

The content of JA was determined with the previously reported method (Wan et al. 2020). Briefly, 30 mg freeze-dried sample was dissolved into 800 μL of solvent mixture containing methanol/H_2_O/acetic acid (80:19:1, v/v/v) and internal standard for JA (DHJA; Olomouc, Czech Republic). The extract was then analyzed by an Agilent 1100 HPLC system coupled to an Agilent API 3000 mass spectrometer. The parameters were the same as previous report (Liu et al. 2012). Three biological replicates were set for each experiment.

#### Scanning electron microscopy (SEM) analysis

About 3 mm^2^ of leaf tissues were excised from leaves of WT and OE2 lines and used for SEM analysis. Sample preparation and observation parameter setting were performed as described by Wang et al. (2016). Three replicates were analyzed for each genotype.

#### Western blotting

Western blotting was carried out as previously reported (Li et al. 2019b). Briefly, 100 mg sample was dissolved into 300 μL 2 × SDS buffer and the mixture was then briefly centrifuged. Twenty microliter supernatant was extracted for electrophoresis at 80 V for 20 min, then at 120 V for 1 h in SDS–polyacrylamide gel with different concentrations (upper-layer gel 5%, lower-layer gel 10%). A semidry blotting method was then used to transfer proteins from Gels onto polyvinylidene fluoride (PVDF). After incubation of the membrane for 2 h, the primary antibody and secondary antibody were subsequently added. The primary antibody was Anti-FLAG Mouse Monoclonal Antibody (DI101-02, TransGen Biotech), and the concentration is 1: 10,000. The secondary antibody was HRP-Goat Anti-Mouse IgG (H + L) (00,001–1, TransGen Biotech), and the concentration is 1: 10,000. Finally, the photographic developer was added onto the surface of the membrane to display the image.

### Accession numbers

The species and gene numbers are: *Arabidopsis* (*A. thaliana*), *AtSHN1* (*AT1G15360*), *AtSHN2* (*AT5g11190*), *AtSHN3* (AT5g25390), *AtESE3* (AT5g25190); tomato (*Solanum lycopersicum*), *SlSHN3* (Solyc06g065820), *SlSHN1* (Solyc03g116610), *SlERF52* (*BAO18577*), *LeERF1* (*AAL75809*); grape (*Vitis vinifera*), *VvERF045* (*ANT73695*); barley (*Hordeum vulgare* L.), *HvNud* (*BAG12386*); wheat (*Triticum aestivum* L.), *TdSHN1* (*ANY98960*); rice (*Oryza sativa*), *OsWR1* (Os02g0202000), *OsWR2* (Os6g0604000); oilseed rape (*Brassica napus*), *BnWIN1*; soybean (*Glycine max*), *GmSHN1* (Glyma07g03500), *GmSHN9* (Glyma07g03500); phalaenopsis (*Phalaenopsis orchids*), *PeERF1* (*MG948436*); Gene sequences of Eucalyptus grandis (*Eucalyptus grandis*) were downloaded from phytozome (http://www.phytozome.net/): *EgrSHN1* (Eucgr. C04221.1), *EgrSHN2* (Eucgr.C01178.1), *Egr33m* (Eucgr.C02719.1), and *Egr40m* (Eucgr.C03947.1). SHN family gene sequences in sweet orange were downloaded from the Citrus Genome Database of Huazhong Agricultural University (http://citrus.hzau.edu.cn/orange/index.php).

## Supplementary Information

Below is the link to the electronic supplementary material.

Supplementary file1 (PDF 80 KB)

Supplementary file2 (PDF 847 KB)

Supplementary file3 (XLSX 21 KB)

Supplementary file4 (DOC 7297 KB)

## Data Availability

The datasets generated and/or analyzed during the current study are available from the corresponding author on request.
