# Supplementary material for: Transcription factor CsESE3 positively modulates both jasmonic acid and wax biosynthesis in citrus
Source: aBIOTECH. 2022 Nov 22;3(4):250–66. doi: 10.1007/s42994-022-00085-2 (PMC9755798; doi:10.1007/s42994-022-00085-2)
Supplement: Supplementary file 1 — Supplementary file1 (PDF 80 KB) [file 42994_2022_85_MOESM1_ESM.pdf]

Supplementary Table S1. Blue module genes that were significantly correlated with CsESE3 in WT and DEV.

Gene TD	Gene Name	DEV.cor.ESE3	WT. Cor. ESE3	Annotation	Pathway	KO.Number	BestHit in Arabidopsis
Cs7g06120	ESE3	1	1	Ethylene-responsive transcription factor	Wax	-	AT5G25190
**Wax biosynthesis**							
Cs1g02760	CER1	0.94	0.90	Protein WAX2	Wax	K15404	AT1G02205
Cs4g02580	CER3	0.93	0.92	Long-chain-alcohol O-fatty-acyltransferase family	Wax		
Cs4g09520	AT5	0.97	0.94	Oleoyl-acyl carrier protein thioesteras	Wax	-	AT5G55340
Cs5g27720	FATA	0.93	0.72	Fatty acyl-CoA reductase 2	Wax	K10782	AT3G25110
Cs6g12890	FAR	1.00	0.73		Wax	K13356	AT3G11980
**Jasminic acid related genes**							
Cs1g17210	JAZ1(b)	1.00	0.61	Linoleate 13S-lipoxygenase 3-1	ic Acid	K13464	AT1G19180
Cs1g17220	JAZ1(a)	1.00	0.49	Alpha/beta-hydrolase domain-containing protein	ic Acid	K13464	AT1G19180
Cs1g17380	LOX3	0.96	0.37	Alpha/beta-hydrolase domain-containing protein	ic Acid	K13464	AT1G17420
Cs2g03240	JAZ8	1.00	0.60	Allene oxide	ic Acid	-	AT1G30135

Cs2g28830	PLIP1	0.98	0.56	Jasmonate O-methyltransferase	ic Acid	-	AT3G61680
orange1.1t0165	PLIP2	0.99	0.37	12-oxophytodienoate reductase 2	ic Acid	-	AT1G02660
Cs3g24230	AOS	0.98	0.47	Putative 12-oxophytodienoate reductase	ic Acid	K01723	AT5G42650
Cs3g25140	JMT	0.99	0.43	12-oxophytodienoate reductase 2	ic Acid	-	AT1G19640
Cs5g17900	OPR	0.95	0.36	Protein TIFY 10A	ic Acid	K05894	AT1G76690
Cs5g17920	OPR	0.97	0.36	Protein TIFY 10A	ic Acid	K05894	AT1G76690
Cs5g30350	DAD1-like	0.87	0.52	Protein TIFY 5A	ic Acid	-	AT1G06800
Cs7g02820	JAZ10	0.97	0.36	Protein TIFY 9	ic Acid	K13464	AT5G13220
**Phospholipid metabolism**							
Cs3g14580	Lipase	0.93	0.26	Alpha/beta-hydrolase domain-containing protein	Lipase	-	AT3G05600
Cs3g23440	Lipase	1.00	0.60	Lipase	Lipase	-	AT5G42930
Cs7g09100	Lipase	0.98	0.55	Alpha/beta-hydrolase domain-containing protein	Lipase	-	AT1G10740
Cs1g12430	FAD5	0.93	0.60	Palmitoyl-monogalactosyldiacylglycerol delta-7 desaturase	Lipid	K20416	AT3G15850

Cs2g13110	LPP3	0.99	0.87	Lipid phosphate phosphatase 3,	Lipid	K18693	AT3G02600
Cs2g13440	PSS1	0.98	0.27	Phosphatidylserine synthase 2	Lipid	K08730	AT1G15110
Cs5g11570	DGK	0.93	0.31	Diacylglycerol kinase	Lipid	K00901	AT5G07920
**Ethylene biosynthesis**							
Cs1g21210	ACS8	0.97	0.75	1-aminocyclopropane-1-carboxylate synthase 8	lene_re	K01762	AT4G37770
Cs4g13490	ACS7	0.95	0.64	1-aminocyclopropane-1-carboxylate synthase 1	lene_re	-	AT4G26200
**Others**							
Cs6g21350	GALT1	0.98	0.85		Other	K14413	AT1G26810
Cs6g22150	UGT	-0.87	0.00		Other		AT3G02100
Cs7g25890	UDP-glycosyltransferase 89B1;	0.97	0.41		Other		AT1G73880
Cs2g25450	Peroxidase 55	1.00	0.61	Phenylpropanoid b		K00430	AT5G14130
Cs4g03740	Peroxidase 16	0.98	0.52	Phenylpropanoid b		K00430	AT2G18980
Cs5g18010	Flavone 3'-O-methyltransferase 1	0.94	0.45	Phenylpropanoid b		K13066	AT5G54160
Cs5g18050	Flavone 3'-O-methyltransferase 1	0.90	-0.25	Phenylpropanoid b		K13066	AT5G54160
Cs7g08070	Peroxidase 6	0.99	0.40	Phenylpropanoid b		-	AT1G24110
Cs1g18240	INV	0.87	0.52	Starch and sucrose		K01193	AT3G13790
Cs2g07720	GPT2	1.00	0.38	Starch and sucrose		K05350	AT1G61800
Cs4g02730	ATTPS11	0.93	0.34	Starch and sucrose		K16055	AT2G18700
Cs5g19060	SPS4	1.00	1.00	Starch and sucrose		K00696	AT4G10120

Cs1g20580	TPS	0.94	0.52	Terpenoi	K23232	AT4G37 990
Cs2g08460	LUP2	0.99	0.68	Terpenoi	K15813	AT1G78 960
orange1.1t0563:	TPS	0.89	0.93	Terpenoi	-	AT5G23 960
Cs1g21000	Transcription factor bHLH25	1.00	0.56	TF bHLH	-	AT4G37 850
Cs9g08460	bHLH92	1.00	0.46	TF_bHL	-	AT5G43 650
Cs4g09770	ERF7	0.77	0.80	TF_ERI	K09286	AT3G20 310
Cs5g02340	ERG061	0.92	0.61	TF_ERI	-	AT1G64 380
Cs5g33540	ERFRap2-3	0.63	0.95	TF_ERI	K09286	AT3G16 770
Cs8g05910	ERF109	0.99	0.56	TF_ERI	-	AT4G34 410
Cs9g16820	ERF026	0.90	0.56	TF_ERI	-	AT5G52 020
orange1.1t0529	ERF114	0.71	0.54	TF_ERI	-	AT5G61 890
Cs3g21660	ERF112	1.00	0.40	TF_ERF1	-	AT2G33 710
Cs3g23270	RAP2-1	0.93	0.72	TF_ERF1	K09286	AT1G46 768
Cs3g23070	myb domainproteinr	0.81	0.97	TF_Myl	K09422	AT5G67 300
Cs4g12760	Myb63	0.96	0.52	TF_Myl	K09422	AT1G79 180
orange1.1t0033	MYB39	-0.96	-0.86	TF_Myl	K09422	AT3G61 250
Cs1g08440	NAC	0.97	0.41	TF_NA(	-	AT1G01 720
Cs5g26150	NAC100	0.77	0.45	TF_NA(	-	AT5G18 270
Cs2g02790	WRKY42	1.00	0.60	TF_WRK	-	AT4G04 450
Cs2g06950	Myb102	0.99	0.34	TF_WRK	K09422	AT4G21 440
Cs2g07040	Mb4	0.98	0.63	TF_WRK	K09422	AT2G31 180
Cs5g02440	WRKY30	0.98	0.42	TF_WRK	-	AT4G11 070
Cs5g24240	wrk19	1.00	0.79	TF_WRK	-	AT4G12 010
Cs9g02040	WRKY 48	0.99	0.66	TF_WRK	-	AT5G49 520

Cs6g09960	LTP	0.99	0.87	Transport	-	AT2G38540
Cs5g09390	LTP	0.91	0.48	Transport	-	AT1G62790
Cs6g15290	ABG39	0.99	0.41	Transport	-	AT1G66950
Cs6g18090	ABC tranporter	0.84	0.80	Transport	-	AT3G03280
orange1.1t0176[illegible]	ABCB	0.99	0.65	Transport	K05658	AT2G47000
Cs1g20510	UGT	0.98	0.55	UGT	-	AT2G22590
Cs3g18090	UGT85A2	0.89	0.27	UGT	-	AT1G22360
Cs8g05320	UGT73B4	0.75	0.37	UGT	K14595	AT2G15490
Cs8g19260	UGT76E2	0.98	0.83	UGT	-	AT5G59590
Cs9g16640	UGT76B1	1.00	0.84	UGT	-	AT3G11340
Cs2g03270	NCED5	0.92	0.54	ABA_rela	K09840	AT1G30100
Cs6g19380	Abscisic acid 8'-hydroxylase	1.00	0.92	ABA_rela	K09843	AT4G19230
Cs2g28730	CSLC4	0.99	0.92	Cell Wa	K20887	AT3G28180
Cs3g09000	CSLD3	0.97	0.49	Cell Wa	K20924	AT3G03050
Cs4g03050	XTH23(a)	1.00	0.98	Cell Wa	K14504	AT4G25810
Cs4g03130	XTH23(b)	1.00	1.00	Cell Wa	K14504	AT4G25810
Cs4g03140	XTH22	1.00	0.97	Cell Wa	K14504	AT5G57560
Cs1g22140	GH3.1	1.00	0.91	IAA_signal	K14487	AT2G14960
Cs8g04610	GH3.1	0.76	0.24	IAA_signal	K14487	AT2G14960
