# Supplementary material for: Transcription factor CsESE3 positively modulates both jasmonic acid and wax biosynthesis in citrus
Source: aBIOTECH. 2022 Nov 22;3(4):250–66. doi: 10.1007/s42994-022-00085-2 (PMC9755798; doi:10.1007/s42994-022-00085-2)
Supplement: Supplementary file 2 — Supplementary file2 (PDF 847 KB) [file 42994_2022_85_MOESM2_ESM.pdf]

Supplementary Table S2. Transcriptome of WT and MT. Only genes with Coefficient of Variation (CV) value over 0.3 were selected. Expression of genes was expressed as FPKM.

Gene	MT.90D AA	WT.90D AA	MT.150D AA	WT.150D AA	MT.180D AA	WT.180D AA	MT.210D AA	WT.210D AA	MT.P90	WT.P90
Cs1g01060	17.03647	13.95712	16.31042	14.0149	19.76095	18.6049	15.72146	13.38323	30.99407	13.93
Cs1g01110	3.161729	2.20404	0.428787	1.035566	0.115697	0.744661	10.96922	1.472595	24.26059	3.688122
Cs1g01120	0.309867	0.359412	0.040732	0.15533	0.051522	0.077113	1.483726	0.108775	3.091836	0.433768
Cs1g01130	0.602477	0.51025	0.063617	0.107184	0	0.239691	2.284159	0.161221	4.381914	0.573776
Cs1g01150	1.725575	1.610519	2.586173	2.378654	1.427298	1.31785	0.629021	1.953327	0.282147	1.45047
Cs1g01160	2.248244	1.20472	1.935159	1.493467	1.709827	1.747352	1.29659	1.531962	2.136125	0.732453
Cs1g01180	0.259074	0.31174	1.067264	0.775648	0.350242	0.473079	0.584237	1.762556	3.776351	5.823988
Cs1g01220	209.4677	155.8473	35.32962	39.47546	9.94774	16.72283	6.718559	11.53648	0.451284	0.532173
Cs1g01260	0.549185	0.494389	0.998116	0.538644	1.103067	0.980326	0.44355	1.254497	0.39868	0.519656
Cs1g01320	144.4669	84.85921	71.97787	54.99693	40.80255	40.48437	26.57527	25.14288	13.76845	6.023385
Cs1g01410	1.568858	2.072632	4.89826	4.476973	7.038968	7.821191	8.043802	6.30853	14.29258	8.093135
Cs1g01460	27.67929	19.25192	17.6133	13.39674	5.350512	8.193665	4.812095	9.955185	4.359533	2.144743
Cs1g01600	12.61971	15.49328	16.61641	18.57592	10.64641	23.93688	7.571107	17.44376	62.31417	65.0733
Cs1g01630	20.26981	18.19616	31.63976	14.92929	30.03656	25.94312	28.24742	23.40672	25.50393	23.88937
Cs1g01700	729.3693	1038.606	820.4955	729.9439	429.544	207.0539	334.7884	100.636	34.4251	34.1124
Cs1g01800	7.686894	8.529973	30.49831	40.51598	1.961711	17.27074	15.40733	47.96772	13.26297	54.23865
Cs1g01860	1.29232	0.795282	4.080103	3.73523	5.057995	7.114516	5.232588	9.989096	0.929323	0.354808
Cs1g01880	2.352388	2.259169	2.299984	2.249143	0.767711	1.003117	1.050132	1.661159	0.525931	0.461425
Cs1g01920	10.2733	7.922845	5.542256	5.759128	3.456749	4.099746	3.231331	3.969175	2.173963	1.144068
Cs1g01990	22.77016	25.70903	35.47533	36.15957	32.37448	49.09381	14.32973	42.56765	54.53587	72.5422
Cs1g02070	21.44221	33.95292	19.1221	20.22693	3.835325	7.327936	7.452118	3.009664	11.47758	4.397416
Cs1g02150	2.238691	6.468454	0.076572	1.329707	0.371526	0.251865	0.077889	0.295455	0.235966	0
Cs1g02160	19.05884	27.07686	14.8213	16.03508	1.503338	3.771602	3.203688	1.26529	9.827124	3.935711
Cs1g02240	0.513795	0.566036	2.041899	1.830904	2.753144	2.143202	2.037593	3.334631	1.005672	0.94122
Cs1g02250	0.491942	0.545598	1.92723	1.739856	1.81801	1.600319	1.479694	2.830338	0.822458	0.977371
Cs1g02270	1.017639	1.307912	4.291776	3.687804	15.44179	9.488326	7.900404	7.46622	2.540017	3.212724
Cs1g02280	61.25521	46.54041	40.05577	30.51284	55.92602	70.55958	21.08799	117.1627	61.94276	53.28151

Cs1g02300	75.54743	64.74781	71.4221	66.37926	38.34256	94.66795	37.92179	41.08935	33.39904	39.43993
Cs1g02370	0.433102	0.560321	1.001368	0.631862	0.747671	0.696361	1.660026	4.082887	6.138226	14.89587
Cs1g02670	10.08939	10.55366	8.527899	9.825042	3.980825	4.242776	2.602502	3.580014	0.68792	1.84408
Cs1g02675	37.67537	43.46623	39.96235	39.62266	35.99474	43.44229	20.48443	52.32363	32.47763	58.20724
Cs1g02740	2.121847	2.038802	3.651856	3.640383	8.422179	7.611433	24.54738	17.61186	8.45888	10.65918
Cs1g02750	0	0.062871	0.008779	0.00606	0.009586	0.047453	2.065875	0.245815	1.111417	1.725159
Cs1g02760	16.2112	14.60124	28.45235	22.67408	51.89519	82.16751	186.7115	205.5103	185.8852	256.1424
Cs1g02790	14.86642	15.18066	17.93025	17.84263	11.82543	18.41566	3.258355	10.79957	5.932572	6.965263
Cs1g02990	0.091998	0.072071	0.35497	0.228675	0.637973	0.648937	0.952167	0.351553	0.11653	0.122115
Cs1g03020	0.097562	0	0.377621	0.111346	0.746839	0.301582	0.750965	0.516573	0.273019	0.955621
Cs1g03040	3.402823	4.733142	2.96889	4.422319	1.874647	3.528057	2.053686	4.03149	2.843026	5.899468
Cs1g03070	5.704059	6.138009	5.18503	3.865882	3.040572	3.13589	0.701582	5.101474	0.302057	0.095449
Cs1g03090	8.709883	10.52158	10.52768	9.740466	11.43423	11.29416	5.962557	14.0579	7.042811	7.952455
Cs1g03190	0.201244	0.108038	0.045209	0.11561	0.061723	0.175388	1.627266	0.453613	4.839595	0.331896
Cs1g03210	18.09283	22.52533	19.61179	19.21512	8.596592	9.021569	6.4192	7.444446	0.763149	1.092369
Cs1g03250	0.847095	0.769533	0.88537	1.470392	0.146823	0.283182	0.196445	1.571536	0.037154	0.39535
Cs1g03280	30.66121	42.73893	50.62904	59.86321	17.96713	38.07853	34.50174	109.691	4.61509	17.54669
Cs1g03290	10.98121	8.7449	12.33488	14.2237	4.739155	6.588316	1.21242	1.007685	1.294348	0.235395
Cs1g03320	2.463759	2.712111	3.999043	3.747856	2.529015	3.055702	1.728136	3.488239	1.182317	1.446524
Cs1g03330	4.685707	4.700214	4.959155	5.052029	5.111713	5.473503	3.019307	8.176357	1.840028	3.060982
Cs1g03390	12.17571	14.92131	7.365411	7.094349	4.554621	5.774984	1.894865	2.910878	0.073122	0.332121
Cs1g03570	5.985884	6.824923	3.418986	6.011666	1.828663	2.287184	1.146849	2.120496	1.410859	2.315696
Cs1g03680	1.192698	1.429554	1.132806	0.959772	0.522933	0.671956	0.263076	0.887009	0	0
Cs1g03700	47.3815	44.96616	54.95873	33.04942	23.4016	21.43069	1.556157	4.527576	0.211516	0.305673
Cs1g03730	83.68988	57.00977	194.6017	116.8467	182.4204	124.9154	128.0377	171.5601	14.40042	19.90663
Cs1g03780	43.24886	45.21413	109.7301	67.99946	135.3193	117.4481	139.6493	248.1243	15.08685	16.01956
Cs1g03790	10.73643	12.50279	5.270299	5.742049	2.56072	3.912771	1.817586	4.901552	0.06091	0.067493
Cs1g03800	66.70248	69.30821	147.5434	98.29547	161.7574	168.1971	216.915	399.8044	23.3065	28.92077
Cs1g03820	31.61679	36.92024	16.83272	17.44839	9.129603	14.22423	6.412334	18.20216	0.124966	0.217075
Cs1g03960	1.882595	1.551777	5.610577	7.484094	0.789968	8.775736	1.299054	2.921849	1.031205	5.406171
Cs1g03980	29.49359	48.13025	15.49396	29.30008	4.29308	5.494793	2.028254	2.380871	0.418458	0.36167
Cs1g04080	3.029421	3.585198	2.491291	2.557019	3.426276	1.762661	5.920882	3.498718	1.107446	1.172418

Cs1g04200	0.20091	0.160819	0.144051	0.166308	0.058047	0.232607	0.698038	1.824056	1.398441	1.332027
Cs1g04220	1.53333	2.645011	1.686561	3.396637	0.388986	1.754368	0.037783	0.112025	0	0.019844
Cs1g04340	21.70094	24.6903	14.67338	14.58947	8.353387	13.01788	12.99604	12.15065	3.829232	3.595434
Cs1g04350	11.84069	10.29348	15.1071	14.64637	18.79775	18.82287	29.80285	27.95058	38.40231	24.29528
Cs1g04370	0.863532	0.978416	1.005733	0.769547	1.447017	1.354971	1.839208	1.993071	1.155079	1.983721
Cs1g04485	2.159927	2.054272	2.468109	2.370562	1.894564	1.970188	0.461317	2.135432	1.52188	1.383077
Cs1g04540	48.89662	43.86636	36.59979	44.37849	30.5875	31.11796	17.11235	20.16637	22.13634	15.40702
Cs1g04570	76.72743	60.83712	34.55653	37.81158	9.746284	17.18433	16.56152	33.3241	33.06894	77.16475
Cs1g04580	0.26075	0.231122	0.558315	0.63898	0.357566	1.407117	0.417092	1.696879	0.584344	2.321439
Cs1g04650	1.900409	0.218842	0.272388	0.214101	0.362744	2.742357	0.099643	0.203698	4.446624	0.872318
Cs1g04750	5.983378	9.049209	4.375954	6.273024	3.080015	2.7291	2.652983	6.744977	0.497178	0.837548
Cs1g04840	0.30513	0.489152	1.81787	3.50898	0.486581	2.061022	0.166886	0.14562	0.01188	0
Cs1g04850	0.706305	0.486454	1.95326	1.125779	5.422897	4.306573	8.159349	2.151097	2.497306	1.610978
Cs1g04910	137.0189	86.6794	59.84109	52.52429	73.78112	50.16999	276.277	109.3416	3.122257	3.997872
Cs1g04920	9.90405	6.033195	2.601055	1.761655	3.952554	2.138407	5.837633	6.343372	0.271771	0.072158
Cs1g05030	23.44587	6.568508	15.28929	10.71821	14.67264	20.32363	2.667653	3.554807	4.775749	2.74148
Cs1g05070	17.54215	15.10987	16.66696	16.06904	7.269345	12.13309	6.931836	7.831461	13.99568	6.480132
Cs1g05090	3.555417	3.179461	1.533321	1.004739	1.565506	1.882868	0.70323	1.676088	0.026826	0.091315
Cs1g05110	3.156585	2.930641	3.852018	3.254614	2.191506	2.871207	1.433707	3.340444	1.046495	1.181127
Cs1g05170	7.81407	7.89358	8.759551	7.366405	7.797144	8.525429	2.057717	7.032671	4.99593	4.81163
Cs1g05180	3.147665	3.434419	2.979979	2.735406	0.64556	1.710451	0.122105	0.696191	0.077193	0.054637
Cs1g05370	0.140489	0.222142	0.196111	0.282385	0.375365	1.048945	0.395719	0.916302	0.390663	0.297153
Cs1g05460	0.143902	0.189591	0.269269	0.239639	0.147401	0.632456	0.097728	0.251589	1.059025	0.411153
Cs1g05470	1.020621	0.985925	1.191697	1.162547	1.248993	2.571418	0.773723	1.839282	2.188476	1.573308
Cs1g05510	2.326687	0.95088	0.847214	0.64977	1.39441	1.241667	1.633834	0.853552	0.499797	0.17251
Cs1g05620	0.655202	0.837632	1.242883	1.236595	0.343676	1.328349	0.118272	0.208913	0.148415	0.046026
Cs1g05770	264.7778	279.9173	218.7033	143.6147	239.8258	70.4494	97.5722	121.2801	320.5415	64.97596
Cs1g05780	28.84058	25.99244	22.14888	19.22498	24.6803	18.07484	32.02851	19.86346	16.58426	6.73329
Cs1g05950	1.359037	2.732774	2.993628	2.884489	2.352549	3.404671	0.48825	2.941479	0.911287	2.392932
Cs1g05970	2.937731	2.044259	4.548124	4.30729	2.86028	6.514069	3.184347	7.542262	3.80985	5.603239
Cs1g06040	7.716578	22.4362	2.771145	7.001985	2.399307	2.258291	8.084488	11.34715	7.823381	10.78773
Cs1g06090	1.600844	1.971104	3.193252	2.385767	3.552829	3.524865	1.735393	4.422043	2.240514	2.739463

Cs1g06095	0.711981	0.774033	1.469626	0.66277	1.996369	1.537978	1.17489	2.413489	0.874941	0.882309
Cs1g06140	0.231167	0.234884	0.503928	0.602388	0.810949	1.728543	0.563031	0.827568	0.104436	1.017806
Cs1g06210	12.39794	16.25175	18.18767	18.98617	10.53959	13.29333	9.789052	29.40352	12.00379	31.55875
Cs1g06220	21.09819	21.08536	52.36613	49.77534	13.36028	18.81702	8.936432	51.44345	7.03189	6.399455
Cs1g06240	3.041927	2.03467	4.548599	3.493007	8.884783	9.594959	13.34054	121.8043	121.1281	99.37425
Cs1g06270	0.747258	0.748699	0.529564	0.550256	0.422242	0.809308	0.396183	0.663282	0.645996	0.887067
Cs1g06320	26.09031	55.46367	23.02655	25.10152	9.668848	12.75139	7.664677	12.7937	0.442719	0.320557
Cs1g06360	3.701541	14.26596	1.054107	1.378526	2.007925	2.832921	1.540742	3.74215	2.732419	1.850391
Cs1g06410	0.502466	0.331346	0.048406	0.28704	0.328201	0.509141	0.77933	0.156707	3.680126	0.162844
Cs1g06440	4.515133	4.538847	7.604658	5.909848	27.44386	10.56781	27.50708	12.97219	3.915779	5.048187
Cs1g06480	7.844874	9.106019	6.276274	8.627315	6.143765	5.9071	2.409612	5.644895	4.59942	5.491001
Cs1g06560	0.019573	0.061507	0.02687	0.052968	0.031167	0.061728	0.019825	0.545944	0.107535	0.956275
Cs1g06650	1.915121	1.727469	0.63693	0.958888	0.150325	0.997862	0.875266	4.010428	1.483211	3.239395
Cs1g06740	74.8394	121.8575	56.2075	71.62368	40.52369	48.57957	28.02002	42.92637	26.25557	38.45118
Cs1g06750	10.54375	10.59516	33.24861	27.83926	51.85413	69.38397	81.95295	77.95525	37.37835	65.13201
Cs1g06760	493.6834	196.6461	195.6738	151.0526	244.8091	92.4397	304.2053	130.1155	305.4784	176.9991
Cs1g06770	0.069428	0	0.231966	0.120251	0.376684	1.278604	0.531466	1.212153	0	0.020602
Cs1g06790	23.84705	32.71951	25.04856	27.32898	11.23705	15.93352	4.496356	24.21145	12.5749	35.25063
Cs1g06830	75.42041	83.29772	28.51564	34.09654	8.154084	25.77989	6.583103	29.02938	33.301	65.93318
Cs1g06850	56.49234	82.28572	33.5257	32.7614	18.42274	19.63563	5.801015	20.15704	2.226925	2.587478
Cs1g06880	115.2525	117.5807	63.52175	54.62858	33.35752	35.89486	20.64747	121.2505	26.4888	91.65804
Cs1g06910	6.135475	4.965219	0.832095	1.152828	1.283492	6.226514	5.821762	10.74698	8.850506	26.7332
Cs1g06970	5.436907	8.501612	8.793872	8.169445	5.409759	7.800377	1.68835	10.65358	0.935015	0.691153
Cs1g06980	10.09157	12.69058	9.121061	9.773835	10.63116	19.04378	4.670294	12.23531	3.414411	4.630751
Cs1g07000	8.07933	10.28978	15.74499	12.67274	14.55446	17.46681	9.252992	21.50315	6.62869	6.432834
Cs1g07020	8.147433	12.53864	12.39379	12.53856	7.352338	8.720667	2.233281	10.61928	2.19653	5.195234
Cs1g07110	20.79588	11.04466	8.289177	7.113188	15.02263	16.01022	5.89667	9.172545	4.15364	8.766809
Cs1g07210	0.919572	1.342832	3.721212	2.021245	5.120709	4.394269	7.026418	4.932839	1.578107	1.074776
Cs1g07260	2.767883	3.110699	5.193328	7.594688	2.038908	2.577507	1.068996	3.043906	0.221332	0.373375
Cs1g07380	14.91483	13.55348	18.62985	17.50314	14.34	17.98216	11.58973	30.8895	0.610221	1.470601
Cs1g07420	35.94867	46.64069	17.88597	23.47991	16.45248	6.628239	5.28511	6.751415	1.993998	5.739245
Cs1g07460	3.976166	3.563644	5.434091	4.468925	10.53695	5.201178	13.58812	13.18249	7.606965	1.971194

Cs1g07510	278.7829	247.7931	113.4427	122.9317	62.87363	45.85312	36.93288	29.5292	25.01038	11.70896
Cs1g07550	17.68903	27.91445	8.380556	9.657259	10.62488	6.928987	7.752103	18.70171	1.853311	1.860318
Cs1g07940	20.21097	23.03576	18.45672	22.0203	14.76601	17.5473	8.940319	22.54072	13.84667	17.7572
Cs1g07950	516.6384	527.2483	317.9803	397.4913	162.9564	186.2481	210.9164	176.1799	387.1695	259.879
Cs1g08010	39.68445	53.71756	65.66338	52.80626	69.8065	57.73672	134.0523	206.6865	53.42153	105.2169
Cs1g08020	3.478991	4.195748	2.824075	2.776867	4.598874	4.543101	3.934954	9.151225	1.475866	3.837841
Cs1g08030	9.995306	7.436399	18.7313	12.84588	13.03587	21.12484	1.636565	3.415571	0.01512	0.058274
Cs1g08110	12.99584	13.64849	15.67845	11.87641	14.41608	19.80917	17.14054	17.68213	0.224749	1.673199
Cs1g08130	0.434556	0.253555	0.47881	0.378741	0.09639	0.22282	0.443662	4.128873	0.959218	4.133506
Cs1g08150	4.69153	5.788911	5.280697	4.190914	2.804436	2.925081	1.969832	2.769262	0.125174	0.225683
Cs1g08205	0.157204	0.200867	0.501217	0.490945	1.92406	1.670445	1.561501	2.113473	2.677372	2.484588
Cs1g08270	20.96827	17.1415	40.11194	33.57498	67.69009	62.18787	25.50573	25.86335	19.00632	17.91894
Cs1g08380	19.38083	8.415448	8.130445	7.238956	5.976479	18.46665	4.012552	16.27182	3.351692	1.730123
Cs1g08440	110.2451	75.04623	90.70991	126.9391	14.10997	47.66817	72.15516	82.10422	107.8823	275.2793
Cs1g08450	25.71212	19.99557	21.14834	29.37486	4.117343	12.27256	11.29815	20.30077	21.3328	52.44247
Cs1g08480	41.79901	45.99411	45.52548	49.23936	27.67521	36.76509	21.06658	49.38661	18.7411	21.89612
Cs1g08510	0.042071	0.063011	0.16499	0.035554	0.17427	0.307886	1.061051	3.433055	0.189063	0.205923
Cs1g08535	0.503161	0.470077	0.370593	0.428693	0.389165	0.575525	0.139155	0.894747	0.043446	0.119938
Cs1g08550	28.63236	48.16259	39.27268	43.83158	84.46549	106.2912	235.6805	255.5657	139.6769	273.7987
Cs1g08720	3.078229	3.372484	7.384006	8.33114	7.583967	8.431652	2.426081	7.227544	0.221964	0.157455
Cs1g08810	47.28399	82.75765	19.71619	40.35679	19.01246	20.81712	40.41975	52.6537	11.34945	15.07733
Cs1g08890	11.65838	12.78082	8.946443	13.71863	1.341393	2.208829	0.826234	1.713373	0.187892	0.28566
Cs1g08950	3.953766	4.495142	4.740005	4.364845	4.598717	5.581092	3.495205	6.123069	1.333105	3.916998
Cs1g09010	4.49116	3.424185	5.211266	3.627567	7.465211	5.304409	9.922629	8.140113	14.8944	6.675884
Cs1g09020	12.1353	7.463304	7.44802	8.958839	7.001835	8.930373	16.72416	15.17932	7.14539	25.54113
Cs1g09130	5.225284	9.071216	3.241948	5.064664	1.38079	2.350357	2.976783	3.752387	18.51362	35.43815
Cs1g09140	42.05833	108.5929	10.97404	23.47243	4.914669	5.786438	1.316726	12.1185	1.66327	2.136075
Cs1g09220	1.224196	1.253464	2.23322	2.593071	0.66888	1.382128	0.400214	1.86521	0.272392	1.116036
Cs1g09230	3.345905	3.579129	3.253301	2.220922	2.412121	2.11532	0.60509	2.016462	0.196354	0.273771
Cs1g09250	9.306335	8.33568	14.42556	13.64786	9.329801	7.734882	5.609956	6.232848	3.765331	5.582153
Cs1g09256	1.126109	1.272634	1.076599	1.063832	1.441511	1.503984	0.439482	1.165811	0.3192	0.151318
Cs1g09366	3.334036	2.629083	3.51359	3.492289	2.603809	2.940023	1.306478	3.463035	0.993961	3.692079

Cs1g09380	8.78696	7.728053	6.342165	4.748183	2.832927	3.137815	1.610117	2.334882	0.608006	0.877818
Cs1g09406	2.025415	1.506132	1.641298	1.875406	1.610703	1.781137	0.412561	2.212308	1.961282	0.703197
Cs1g09420	1203.539	1669.06	989.0686	1483.966	606.4318	545.6209	222.9635	327.3862	343.0116	505.1219
Cs1g09460	0.831094	0.683774	1.183885	1.249505	0.636599	1.370487	0.780458	1.37291	1.0728	1.410895
Cs1g09500	35.11257	43.21547	23.5429	28.72131	13.11908	11.53455	8.369991	17.82586	15.12495	30.45852
Cs1g09530	0.430934	3.858673	0.048233	0.131728	0.237213	0.154985	0.296834	0.551864	0.043486	0.047653
Cs1g09550	9.151327	7.294354	21.83393	15.63673	41.74521	34.8458	38.61037	34.38064	8.877924	4.628368
Cs1g09580	2.85039	6.639513	4.475859	5.324488	1.510005	1.700825	1.691262	4.283171	0.42705	0.44482
Cs1g09590	0.69812	0.842054	1.331229	1.269866	1.180876	1.104513	0.300175	1.473509	0.71539	0.445031
Cs1g09600	0.96242	1.395869	2.641695	4.148128	0.945137	7.082017	0.88366	11.46526	0.861979	1.743908
Cs1g09610	2.34285	4.710472	2.493137	2.707971	0.977652	1.214562	0.813962	2.516212	0.011077	0.103333
Cs1g09630	15.18899	16.37485	34.7668	30.34102	22.05768	17.70983	17.26727	42.80387	5.816257	6.849077
Cs1g09635	6.728311	9.148093	14.37259	20.50608	4.205006	26.59469	2.672797	32.87688	7.622928	8.888736
Cs1g09640	2.49824	3.165241	7.88952	8.030649	2.061625	3.905834	1.623959	4.806381	1.906087	1.308397
Cs1g09660	6.733041	8.750652	11.52484	12.87703	8.855812	11.43503	3.798109	14.38496	5.650075	7.036595
Cs1g09665	1.007127	1.416351	1.503558	2.039644	0.655379	1.440257	0.22327	4.371812	0.627519	0.989306
Cs1g09670	3.360327	4.624843	3.872314	4.137743	1.393538	1.519258	1.489076	3.388085	1.690969	2.987002
Cs1g09830	2.156213	1.102541	0.857967	0.946188	0.533984	1.524429	0.670322	1.176789	1.087436	0.70775
Cs1g09840	72.74662	44.96959	40.86319	41.04133	24.04132	50.13721	26.97687	31.26052	100.5311	50.81921
Cs1g10010	10.31056	14.24262	8.857519	7.907069	4.980095	3.907696	3.260942	3.386044	2.111869	1.959218
Cs1g10115	0.758253	0.733684	0.751927	0.962393	0.685184	2.039958	0.849158	0.850025	0.945589	1.958015
Cs1g10160	0.785234	0.480678	1.027155	0.773359	0.383477	0.945791	0.452568	0.38181	0.615693	0.666214
Cs1g10260	3.921813	3.848289	5.745821	4.753004	7.335864	13.30754	7.053963	20.07053	1.749255	3.77097
Cs1g10350	1.091481	0.353541	1.016495	0.521612	1.030514	0.392221	0.593766	0.132286	1.154762	0.744539
Cs1g10430	0.901031	0.693256	0.612749	0.736248	0.670122	0.731766	0.12821	1.061705	0.408371	0.468865
Cs1g10480	2.037331	1.940817	1.726174	2.139006	1.465014	2.161398	1.691195	3.822702	2.125246	4.182701
Cs1g10500	9.985559	5.937905	13.70058	13.81347	7.24834	11.74976	5.302097	3.044386	4.403333	0.949805
Cs1g10540	333.8131	302.8333	248.932	243.7592	74.43938	99.35969	31.10508	21.2556	16.80822	6.268542
Cs1g10610	9.375998	32.18375	3.349138	8.701606	3.485353	3.569878	8.451536	18.08943	15.14781	14.29894
Cs1g10620	1.880987	1.939095	3.632127	2.534944	2.244745	1.391558	1.184538	2.836619	2.138823	2.109839
Cs1g10680	1.912355	2.267163	1.754935	1.088344	3.244539	0.673647	2.200948	1.405945	1.38077	2.116076
Cs1g10700	10.69461	12.3317	11.21279	11.13066	8.626247	8.511402	4.557266	10.39782	5.724392	7.446446

Cs1g10850	0.360843	0.59038	0.511429	0.428971	0.322382	1.087794	0.099317	0.186694	0	0.02673
Cs1g10900	4.262387	4.225424	3.904229	3.915548	1.607132	2.293971	0.697556	3.427066	0.181203	0.257315
Cs1g10925	14.52719	13.91067	9.096007	12.20925	2.622551	5.013537	0.943772	5.047271	1.200194	1.516851
Cs1g11020	9.485115	8.909199	10.55902	9.292883	5.018603	6.873383	3.441321	8.362967	12.00798	20.44057
Cs1g11200	4.75854	3.718729	10.83863	7.841427	17.09766	16.9412	12.38768	14.97797	12.90306	11.61692
Cs1g11350	22.13204	22.2783	24.12514	19.40936	29.24207	16.72667	22.61913	13.39887	34.64174	15.32088
Cs1g11380	23.02848	19.76808	11.96039	7.246449	5.80418	4.059566	1.866246	4.160025	3.142897	1.412933
Cs1g11430	4.362242	3.541547	4.481488	3.860109	3.636088	6.383564	2.828229	6.939522	8.640138	10.48539
Cs1g11740	6.444956	6.910162	4.245611	4.481487	2.51054	2.074332	1.600165	3.579019	1.228028	4.381712
Cs1g11750	6.342025	5.108536	9.571636	8.581055	11.57769	11.23287	4.248144	10.28412	9.697856	7.691202
Cs1g11790	1.352943	1.081477	2.257043	0.895642	2.087656	1.891946	1.048827	2.218934	1.143168	0.9553
Cs1g11800	13.01993	11.30358	24.52459	23.24503	32.33853	51.50544	44.18104	92.69553	18.15626	29.22808
Cs1g11840	1.811457	2.095721	3.863189	3.498443	0.871064	1.63963	0.562389	4.119441	0.315159	1.123511
Cs1g11850	2.354611	2.871915	4.072371	4.221995	1.254621	1.702685	1.021693	3.857431	0.331448	1.229669
Cs1g11860	7.597234	11.24101	8.091751	8.197256	8.65877	5.809306	4.522921	15.58655	6.173834	4.729165
Cs1g11880	0.133512	0.209143	0.534415	0.438487	1.195395	1.086744	1.612311	3.725755	0.849813	2.501978
Cs1g11900	0.067504	0.066107	0.518007	0.535601	0.111032	0.447524	0.096154	0.551743	0.366485	1.912782
Cs1g11930	0.527989	0.640014	2.124925	2.393535	2.805988	3.436764	0.568704	1.690625	0.867585	1.608431
Cs1g11960	0.095086	0.150877	0.569662	1.012476	0.425529	1.521518	0.042668	0.804843	0.060745	0.295673
Cs1g11980	0.595138	0.778113	2.582064	2.704937	3.233806	4.235593	0.924982	2.36237	0.926242	2.382619
Cs1g12200	1.356054	1.602118	1.639632	1.344728	1.253598	1.433357	0.680777	1.659986	0.68557	1.246657
Cs1g12210	3.189702	3.200909	3.152666	2.300625	1.852242	1.464012	1.253996	1.514123	0.701118	0.38356
Cs1g12245	8.022914	6.787899	6.915058	6.747599	9.818664	9.354407	19.63371	6.32095	8.329736	8.107174
Cs1g12270	6.116047	6.381694	7.28132	7.386681	4.22807	4.560544	1.115849	3.496079	1.906396	4.42893
Cs1g12290	2.3704	2.038581	3.409318	3.103021	7.4157	11.96511	11.99004	5.117423	22.34961	16.00677
Cs1g12300	6.253626	6.907385	21.22364	7.964171	9.866697	13.64053	2.023453	1.206856	0.038298	0.036232
Cs1g12310	5.921773	1.387093	0.484031	0.612458	3.484395	1.882076	0.623393	0.136721	0.7526	0
Cs1g12410	79.37749	57.44833	27.74818	47.33634	13.07307	18.85937	33.15082	43.23981	84.61824	171.8292
Cs1g12430	0.280141	0.352753	0.570508	0.933048	4.494333	7.396842	14.40155	6.491436	77.57495	13.64509
Cs1g12440	2.484561	2.458578	4.335225	4.616851	2.897978	9.28732	1.018721	4.600287	31.46904	23.98071
Cs1g12450	5.278666	5.155893	3.260294	3.329239	1.8921	1.412402	2.06196	2.361839	1.02178	0.676361
Cs1g12460	5.355562	3.129298	2.316363	1.89009	1.623114	2.113936	1.546867	1.038172	0.557205	0.176932

Cs1g12480	0.80076	1.167273	1.20973	1.282067	1.192269	1.625689	3.537661	2.873607	0.035688	0
Cs1g12510	0.831354	0.831134	1.831343	1.284561	2.14259	2.00563	1.53844	1.450383	0.018658	0.023267
Cs1g12520	7.043389	6.244712	13.80146	9.657501	18.3558	17.48547	11.99576	9.576114	0.374764	0.458771
Cs1g12560	5.543178	6.893246	5.794958	5.027873	7.133358	10.14736	7.133679	23.65713	55.2061	58.39726
Cs1g12580	103.5886	106.6274	91.38077	85.95762	57.08592	59.77034	10.21619	40.22627	21.38025	22.8128
Cs1g12610	2.308539	1.257316	5.001892	2.409637	2.675969	1.106166	2.594984	0.549157	5.464171	2.271616
Cs1g12660	13.62142	24.81204	9.822737	18.04634	16.54162	8.809747	31.96633	43.66907	3.293404	4.797667
Cs1g12670	5.436259	6.252917	2.723905	3.393826	5.247921	2.738943	6.596696	7.083829	3.754134	5.778106
Cs1g12820	0.310575	0.231403	1.935799	1.73263	6.782703	7.407681	3.559637	6.852871	2.090139	2.038481
Cs1g12880	5.116608	4.120554	4.638475	5.381151	6.781335	11.23034	10.49085	8.381919	15.71008	6.675026
Cs1g12915	0.858193	1.078652	0.86526	0.690172	1.766767	1.835115	0.615231	1.756209	0.537704	0.505671
Cs1g12960	1.037952	0.963787	1.206894	0.960657	1.318055	1.584535	0.369076	1.752816	1.581904	1.715285
Cs1g13080	2.244811	1.959933	6.359634	6.685983	5.621458	9.333869	13.33093	18.49261	17.00932	6.589788
Cs1g13220	1.508613	1.47506	3.491705	3.280088	4.40384	3.703236	5.805633	5.659964	2.619079	3.54378
Cs1g13290	0.794438	0.793652	0.198262	0.268721	0.26041	1.242068	5.067208	5.723814	0.689745	0.837517
Cs1g13300	51.30875	28.62256	14.60629	18.66524	2.595058	3.440215	1.228292	2.070616	0.169711	0.206662
Cs1g13430	4.078187	3.695942	4.813415	4.53362	5.489777	7.178663	9.706559	8.712052	6.602557	7.882093
Cs1g13485	1.331693	1.350916	1.536993	1.577464	1.037657	1.472001	1.214542	2.795998	0.728748	0.872468
Cs1g13530	41.39483	25.59194	91.92236	103.4711	94.16229	142.4275	109.0934	71.25311	455.7088	437.1541
Cs1g13540	2.744575	2.271264	2.728803	2.861639	2.557693	1.877886	5.04859	1.282084	0.879023	0.392803
Cs1g13620	22.25089	12.58876	12.47176	11.80812	48.20426	37.07679	208.2652	68.33977	129.4372	113.795
Cs1g13640	12.36235	10.09597	4.532003	4.054466	5.269377	8.550882	0.998557	1.395593	0.174646	0.279922
Cs1g13710	19.26711	19.41248	15.9913	14.40532	6.15811	5.473828	4.124444	3.67494	1.057216	0.964155
Cs1g13850	22.64109	22.77737	25.00134	21.52894	56.52351	39.06274	144.8166	89.77766	136.6042	97.32422
Cs1g13870	9.862923	9.376649	11.6033	15.73131	19.9164	24.33901	35.91979	12.63498	19.09663	17.62098
Cs1g13900	0.740208	0.7049	1.374715	1.029241	0.932506	0.595461	0.257147	0.761804	0.466946	0.194982
Cs1g13960	64.15206	59.59537	127.3995	96.5567	87.13219	120.4431	151.229	134.6721	37.71528	32.7938
Cs1g13970	14.06319	13.13731	14.54523	18.88494	1.929346	4.541091	2.499935	8.322096	1.029827	0.569342
Cs1g14060	22.35663	20.2823	13.37739	10.26765	13.02224	10.74536	9.034344	5.620401	2.959052	2.952436
Cs1g14160	1.304402	1.337236	1.502239	1.467088	2.282294	2.506923	3.138747	0.951923	1.967987	1.614426
Cs1g14280	2.61581	3.91855	9.321077	8.753689	6.664532	9.570681	0.778004	1.492492	0.055326	0.031199
Cs1g14360	1.751253	2.067683	1.932192	1.693622	0.835537	0.809587	0.207068	0.852428	0.055023	0.057309

Cs1g14500	3.668412	3.256134	6.813256	4.55647	7.16287	7.148589	3.096595	2.962757	0.54521	0.248004
Cs1g14540	5.94941	6.096799	6.931133	5.656014	9.008958	9.985672	3.700082	9.732907	12.2607	7.921701
Cs1g14810	10.87998	4.090809	11.30285	6.290981	2.443833	2.597518	1.542023	2.387998	1.601982	1.461688
Cs1g14830	28.42682	26.11751	30.75919	36.58117	15.75068	27.98822	16.69018	47.9247	29.18692	54.05089
Cs1g14850	5.555014	5.781717	6.974438	6.60204	0.824314	1.949914	0.243078	1.891291	0.467137	0.440539
Cs1g14870	0.953317	1.56065	1.427539	0.877853	0.961457	1.263885	0.273819	1.076338	1.575816	1.12475
Cs1g14880	3.135532	2.923451	5.09236	4.543914	2.85495	6.259076	2.288113	8.628763	0.952914	2.270213
Cs1g14893	7.792325	7.597506	9.898874	11.81501	2.869389	8.303106	2.779913	11.7119	7.345507	15.27484
Cs1g14896	10.9389	7.503127	11.6289	10.85694	3.118595	8.698907	3.749532	8.833447	7.452977	11.33321
Cs1g14920	0.16935	0.220417	0.784033	0.530909	1.348171	1.640639	2.229591	1.621532	6.459831	6.311745
Cs1g14970	0.788135	0.360352	0.881357	0.739427	0.80208	0.902283	0.182913	1.715005	3.190221	1.474913
Cs1g14980	18.39174	13.18969	10.16829	9.814761	10.96754	13.67365	23.7384	24.10417	26.18028	11.07876
Cs1g15060	8.223966	9.572652	6.87645	6.722568	11.27725	8.031496	17.82675	7.668691	7.675039	6.311837
Cs1g15120	50.75493	42.50649	34.16531	32.74086	16.8572	22.99172	6.871959	6.814587	1.883015	1.654179
Cs1g15160	0.108865	0.084364	0.098798	0.142306	0.107697	0.231932	0.07628	0.119847	0.448581	4.63712
Cs1g15170	232.4497	407.9795	93.97109	146.7808	32.69455	30.25152	9.838817	50.0719	4.703533	9.496621
Cs1g15200	16.07989	14.78038	26.21159	23.22354	20.09386	28.50755	23.37734	44.11987	23.51531	55.80556
Cs1g15390	60.15398	39.38013	59.88933	76.23044	23.2986	22.3552	15.59634	14.05587	2.30001	2.399094
Cs1g15440	2.34714	8.478671	0.067556	1.157759	0.041102	0.723782	0.079218	0.889308	0.343345	0.256681
Cs1g15660	0.571999	0.845164	0.327512	0.50668	0.722974	0.897985	1.838074	0.635636	0.48965	0.170537
Cs1g15670	9.272763	1.217528	18.69265	23.78513	32.24726	68.14128	24.08654	17.74883	17.61276	6.431345
Cs1g15710	15.44286	12.1577	3.355907	3.946256	1.761737	3.769742	4.12053	3.489143	2.824978	0.776869
Cs1g15820	8.752318	6.740316	5.82433	5.79337	2.979703	5.83027	2.28812	5.690303	2.073786	2.321992
Cs1g15830	69.69665	50.6483	65.7792	52.21298	40.92003	42.3346	13.97376	23.03662	1.682439	1.858739
Cs1g15900	10.1913	12.60455	13.80723	12.81291	12.11379	12.59929	8.198598	16.83722	4.046772	7.411322
Cs1g15970	5.378541	6.015308	16.32601	19.73846	10.79879	35.32296	18.58253	12.63729	41.26549	71.91659
Cs1g15990	43.67786	41.93184	30.72928	33.60448	5.419885	8.836134	2.261151	9.691099	0.163272	0.316081
Cs1g16000	123.8817	94.55682	122.4639	112.7932	68.7764	159.6953	27.07084	143.9958	0.672619	0.391713
Cs1g16010	5.546786	3.215608	16.07389	7.944308	11.92318	19.49595	1.081498	3.664192	0.201625	0.161457
Cs1g16020	0.378499	0.842146	1.704309	2.214569	3.192048	2.612609	0.428382	1.943797	0.260676	0.25935
Cs1g16023	0.336747	0.374114	1.240053	2.923251	1.63152	0.933593	0.369138	1.270618	0.214104	0.195897
Cs1g16026	0.814023	1.1299	2.206389	1.870612	3.580465	1.858087	8.611795	9.088406	0.450999	0.428458

Cs1g16030	10.92269	26.58375	1.010337	1.012928	5.11852	5.478584	1.27584	1.264088	0.300106	0.429264
Cs1g16170	33.96854	36.85578	23.30732	24.0224	12.9783	12.08563	6.661218	10.63451	8.136143	10.14727
Cs1g16180	6.972396	1.605908	0.262819	0.31159	0.120842	0.446453	0.052738	0.193682	0.807894	0.782492
Cs1g16183	3.31151	3.073752	2.608575	2.626401	1.434319	1.969656	0.523951	1.324001	0.47512	0.201736
Cs1g16220	28.62746	25.89215	109.1934	123.5165	24.98078	35.65462	40.6277	73.46953	27.43414	135.5874
Cs1g16276	0.899074	1.371303	1.422056	1.758562	1.036417	1.657015	0.507773	1.544314	0.259761	0.469061
Cs1g16325	0.943572	1.392814	1.666284	1.308581	1.650965	1.553578	0.778604	2.624711	1.478146	1.455955
Cs1g16390	15.4657	63.70259	0.951493	6.89599	31.42	22.44188	33.24445	69.13514	30.5475	37.55762
Cs1g16425	2.30607	2.101226	2.223194	1.890531	1.953092	2.46571	1.244472	2.902227	3.043798	2.545953
Cs1g16430	609.5846	307.3377	281.1349	237.8326	341.6892	565.2762	644.4067	351.1591	451.7206	242.8794
Cs1g16440	45.6646	42.94761	21.91835	21.24066	13.92897	10.28988	6.13741	9.909922	2.595842	4.095924
Cs1g16460	7.384454	6.009768	10.07847	10.37549	9.698054	21.20921	8.588606	15.19357	9.347353	7.591941
Cs1g16470	28.3683	23.89357	15.92211	14.89297	9.453719	9.708799	4.804706	5.811023	8.752598	3.761515
Cs1g16560	43.59195	64.40822	42.09803	47.97091	15.75479	22.54715	23.64809	59.20189	15.09498	33.48767
Cs1g16590	18.19931	17.90934	32.11109	20.8369	26.7724	30.2789	13.51028	38.85668	1.334155	4.140046
Cs1g16650	23.44137	41.7969	47.93065	88.16796	1.718365	8.89637	3.688575	29.69081	16.63974	20.07386
Cs1g16670	8.162222	7.257763	9.151878	9.488901	3.782637	4.418501	1.854066	2.482012	0.224803	0.20901
Cs1g16730	0.448044	0.432356	1.55236	1.280829	4.869201	2.952361	4.010689	12.49843	1.312668	2.106929
Cs1g16790	1.422158	1.218966	1.238193	1.95296	0.471221	1.204096	0.740429	9.394852	3.026735	0.768346
Cs1g16820	5.92356	5.088202	6.994056	3.621472	18.91182	25.13233	7.964696	15.21274	9.96792	16.67113
Cs1g16830	4.507082	4.966697	6.866986	8.106078	9.501887	14.80996	1.354493	2.436386	0.475658	8.528025
Cs1g17210	39.9168	44.72642	108.6679	160.4469	13.00573	46.63915	64.63547	194.1107	321.3726	603.2708
Cs1g17220	63.51666	97.26103	169.8219	350.9506	30.72857	123.8223	117.4308	255.8338	513.7047	1016.238
Cs1g17250	23.47558	22.21176	17.33077	20.28164	8.447845	12.29918	6.626487	18.62652	12.06034	17.18479
Cs1g17280	6.856203	7.035528	6.075073	6.107805	4.068988	4.188042	2.091373	6.032303	2.941682	3.548091
Cs1g17290	105.526	96.90394	84.35161	66.77828	52.54856	35.53527	13.55881	19.39094	6.033384	5.132057
Cs1g17350	7.151233	4.830049	9.894913	7.888669	16.30839	11.96224	12.20787	3.914809	4.239623	2.139177
Cs1g17360	0.20834	0.073063	0.502406	0.108125	4.140465	2.030713	7.590453	7.718569	0	0.134411
Cs1g17380	65.23275	66.12555	95.50643	124.8648	38.558	67.66881	83.27895	70.83995	347.6261	338.1411
Cs1g17390	17.82001	15.98794	38.60339	40.27068	100.1789	128.3409	31.84166	10.92027	15.39041	26.65884
Cs1g17410	35.18907	25.18794	83.99522	72.96585	95.01232	95.49858	33.77081	21.36962	8.743474	10.51251
Cs1g17420	8.524929	9.001647	24.80724	22.88909	238.4546	263.4336	420.2204	401.0402	95.44692	172.3215

Cs1g17500	0.431712	0.248031	0.145935	0.237598	0.182594	0.347903	0.7304	1.67616	0.767838	0.356922
Cs1g17510	14.98963	13.31449	24.06508	20.57688	9.948524	16.3792	7.221441	18.55341	2.440872	2.364019
Cs1g17530	0.415004	0.319622	2.180848	1.694535	2.58391	2.273162	2.366216	1.842154	1.574798	0.987077
Cs1g17550	8.498051	11.64524	2.383075	2.992595	7.635204	11.40259	1.604861	7.696825	3.564699	1.681849
Cs1g17650	2.541823	3.759122	2.786773	2.968959	4.293221	7.136004	5.401611	14.91747	9.824922	22.58665
Cs1g17660	7.560133	9.329106	6.062788	7.84112	2.803727	3.702809	2.446925	5.098211	1.507653	2.076144
Cs1g17690	0.754116	0.629902	2.299488	1.694404	2.021597	2.360351	4.58429	2.058029	1.544253	1.680584
Cs1g17770	0.733559	0.543256	2.078149	1.770493	2.852093	3.911054	0.368159	0.77382	0.717575	0.487682
Cs1g17820	1.201952	0.536519	1.346961	0.313144	0.71101	0.505252	1.063755	0.246519	0.47956	0.067679
Cs1g17840	5.458822	3.76329	9.771706	8.420609	16.64695	19.84814	32.59782	14.72245	26.42972	16.75303
Cs1g17850	8.320805	6.47772	19.33541	21.24529	28.57354	42.25027	21.87017	35.5648	24.89441	33.2339
Cs1g17860	4.298397	4.311624	7.775797	10.83453	2.482234	4.635782	11.90373	26.82554	28.69521	56.84576
Cs1g17980	0.938044	1.395807	3.121806	2.542108	2.328731	3.222735	1.055259	4.152536	0.21469	0.655832
Cs1g18010	0.591613	1.226433	1.353363	1.410685	4.2692	4.24468	5.289245	11.91909	2.322077	4.276113
Cs1g18120	13.97039	11.29442	8.219013	8.350793	2.56398	4.510946	4.253778	5.416765	3.832838	5.146934
Cs1g18130	22.85765	36.5239	19.93531	21.23248	6.976707	6.241843	15.92478	23.05064	4.845424	18.91169
Cs1g18160	0.56292	0.620691	0.497276	0.465983	0.691519	0.351205	0.6169	0.521343	0.271267	2.71836
Cs1g18220	11.13556	9.303594	29.73072	28.78886	67.52739	179.663	56.65153	46.88773	356.8525	94.92228
Cs1g18240	0.479575	0.345408	0.644375	0.98135	0.312805	1.003766	0.143442	1.081089	1.197	3.920379
Cs1g18300	5.675787	6.186067	1.75169	2.172167	1.170388	1.696147	1.317657	2.535335	0.220773	0.318721
Cs1g18360	3.881851	7.835452	3.269244	2.856873	8.908968	9.084163	11.26949	22.70209	21.34613	25.86058
Cs1g18380	7.096726	4.275805	4.112344	4.155591	3.032323	5.487625	4.368131	8.620161	3.754215	6.352031
Cs1g18400	16.02064	43.21963	13.6709	26.7746	4.234364	10.79015	1.2783	18.78959	2.227502	9.320619
Cs1g18540	3.788162	19.8371	45.41343	92.71283	31.64562	379.9742	33.01123	128.2423	3.06158	10.85263
Cs1g18560	4.030003	4.292303	1.383267	3.15454	1.140695	1.091044	0.872362	2.723172	9.495122	6.015808
Cs1g18580	15.18627	23.50438	36.4065	45.00442	9.113586	9.164625	17.20003	33.71053	9.545129	23.91996
Cs1g18680	4.019354	5.536204	4.838165	5.882119	2.217044	5.220081	1.199456	4.500318	3.129633	5.699603
Cs1g18820	2.167866	2.143721	2.955012	2.015082	3.027556	2.99456	1.576764	4.074511	2.193631	2.020003
Cs1g19130	25.38072	23.21815	21.22568	26.68844	9.919975	14.32017	12.15622	10.96604	16.92502	28.42648
Cs1g19190	0.546641	0.80035	4.105061	4.421235	6.573798	6.957119	3.750679	12.5082	0.988413	0.543071
Cs1g19290	8.564538	2.756437	7.245633	6.933264	6.030694	19.44979	27.86599	21.4188	61.19649	65.4591
Cs1g19320	26.66746	26.60056	47.74612	26.90917	64.31171	46.3992	33.9943	18.35164	4.801281	3.327887

Cs1g19425	12.53367	13.88946	24.0545	19.26617	19.63385	16.68691	4.387741	13.53139	3.638251	4.83009
Cs1g19470	6.451826	9.500606	6.050974	8.801442	7.668326	9.114833	5.890418	14.04071	14.07514	34.12082
Cs1g19550	4.658789	5.777761	6.331475	5.480497	6.276094	6.915882	2.669387	8.131943	2.810908	1.616424
Cs1g19590	9.567569	7.34246	13.11284	15.4791	18.4035	21.5418	23.23093	22.60758	81.78594	66.33983
Cs1g19730	27.55717	30.63895	9.69118	9.48625	3.281872	2.980054	1.10606	3.264326	0.223437	0.68236
Cs1g19780	19.01988	25.87881	11.53057	18.61934	6.061605	7.681528	6.393876	16.55226	8.949189	16.28435
Cs1g19805	5.514712	7.067491	7.406743	7.888682	5.792632	10.71401	3.082355	12.06286	3.895057	5.635526
Cs1g19830	29.56579	43.97756	39.81099	52.55338	90.92088	158.6553	102.7035	40.07268	60.23289	74.09206
Cs1g19840	355.8782	358.7225	409.9954	368.3549	158.5489	195.5762	57.25872	156.6668	75.50545	115.6206
Cs1g19860	15.88453	15.17411	20.15614	17.91866	9.013788	10.18008	2.544927	2.847726	0.57816	0.529428
Cs1g19890	6.173031	13.01315	1.134979	2.229279	0.431137	0.23029	0.180936	0.313683	0.25817	0.167952
Cs1g19920	0.069474	0.022781	0.103124	0.050213	1.013324	0.395785	0.55575	0.414793	1.28694	27.02671
Cs1g19930	10.1705	6.470918	21.54283	14.12058	41.86854	25.77254	43.33606	16.323	9.415239	8.141052
Cs1g19940	0.642657	0.609946	1.325817	0.766334	1.083973	1.029178	1.330398	0.880923	1.016076	0.95445
Cs1g19970	0.15589	0.186611	0.460465	0.533352	0.721916	0.769758	0.556048	1.536824	0.378807	1.062223
Cs1g20000	50.63651	56.44002	68.03737	71.08213	77.87143	56.84419	36.25336	98.01979	118.3608	57.53787
Cs1g20070	0.686532	0.326141	0.493744	0.246224	0.395549	0.611537	0.278058	0.274068	0.036122	0.0895
Cs1g20120	0.075493	0.103394	0.18668	0.088903	0.041864	0.02147	0.312935	2.46484	7.178401	13.519
Cs1g20130	2.224725	1.79412	0.504707	0.56698	0.076913	0.041657	1.344555	1.066326	0.341984	0.211998
Cs1g20150	67.14203	78.12192	78.14012	94.52175	18.83262	18.77026	74.19444	173.2524	147.1461	398.7188
Cs1g20230	70.50405	69.6568	39.76615	35.832	13.58197	14.85366	7.5601	30.90908	0.512758	0.404621
Cs1g20260	2.271358	2.364007	3.796427	3.016572	1.476529	2.710022	0.763404	2.275393	2.534128	2.068158
Cs1g20360	1.243467	1.294173	1.995694	1.44151	0.734217	1.470771	0.411122	1.712669	1.924307	1.995004
Cs1g20380	48.25421	44.89977	25.80053	30.11363	16.33033	16.62782	15.18863	19.4546	7.38389	14.57576
Cs1g20390	11.60302	14.92598	11.42795	10.00991	16.44435	15.48386	25.62849	28.81924	24.65549	26.31363
Cs1g20400	6.710737	9.180643	7.241927	8.027818	5.380645	6.118094	1.861188	8.211847	1.487275	3.78869
Cs1g20420	11.55663	25.75349	10.71026	11.52342	23.87747	9.119138	15.46597	16.37652	1.26898	1.023675
Cs1g20460	5.332759	6.599215	4.433346	5.923569	0.883925	1.505286	0.896279	2.107155	0.151661	0.24917
Cs1g20490	0.686016	0.592032	1.452631	1.431641	1.570567	3.370658	1.595035	1.815466	0.217602	1.880608
Cs1g20510	0.012619	0.036949	0.141146	0.117795	0.759803	0.443731	1.061525	0.34613	2.555607	1.199848
Cs1g20530	131.1949	121.0938	315.9692	328.1164	247.1219	286.8529	247.0307	104.5121	374.0839	249.0407
Cs1g20580	237.0152	209.9535	456.7146	465.2022	1348.05	1280.045	1789.418	787.5114	2879.826	1853.582

Cs1g20610	10.60607	11.17206	40.57879	32.42497	38.77528	33.60175	14.81461	19.22472	3.676097	7.178091
Cs1g20620	0.129624	0.161654	0.186199	0.255836	0.142664	0.161488	0.040537	0.111974	0.167183	1.011924
Cs1g20690	22.45435	36.25748	6.915847	20.41475	1.136214	1.352725	1.336389	1.643874	0.541674	0.314407
Cs1g20705	26.90472	45.04379	8.216172	22.48208	1.06795	0.961668	1.776707	2.064182	0.434901	0.358553
Cs1g20710	54.30277	90.37865	16.11481	56.67677	2.586434	2.909128	3.136215	3.725424	1.612382	0.920702
Cs1g20760	1.116982	0.9456	11.39447	11.09915	13.39908	18.21219	9.373772	15.14994	9.654598	7.97744
Cs1g20810	0.3122	0.353334	0.160326	0.215234	0.162426	0.266219	0.137615	0.217538	0.744396	0.257532
Cs1g20820	31.0287	39.9328	120.5381	130.3406	109.3861	162.4281	37.46364	173.1229	128.3689	138.9387
Cs1g20870	6.716118	4.198646	1.858264	1.57534	3.298802	10.17394	4.48748	10.68718	3.911054	1.126569
Cs1g20880	107.5566	85.5445	151.5211	123.3509	59.69221	54.56621	54.018	58.23043	22.31637	27.79742
Cs1g20920	29.42682	37.06916	51.3333	81.31404	31.73253	46.4003	18.31772	53.90545	33.68865	75.55386
Cs1g21000	11.51436	7.45868	23.4121	19.45928	61.26453	35.66792	108.348	70.35714	460.9185	363.9148
Cs1g21020	15.7298	12.83934	6.403969	6.275144	3.435367	3.699143	5.941742	3.131795	3.480026	0.782066
Cs1g21060	7.280181	6.873449	1.86678	2.441283	2.700698	3.147893	3.406678	1.536715	1.159513	0.151412
Cs1g21110	63.64373	54.36853	56.28907	46.29305	72.2327	46.44834	86.18018	39.55114	51.90361	31.56272
Cs1g21130	842.1479	1313.692	111.2137	121.3593	31.73136	50.41207	34.71779	75.58794	5.402567	6.065095
Cs1g21150	7.652254	9.418853	9.384218	7.520824	10.99362	11.38902	5.966939	14.32379	5.604301	6.220975
Cs1g21160	82.71468	83.05898	69.69732	65.21797	63.15004	58.4478	46.6637	49.51501	65.41408	22.8518
Cs1g21210	1.896374	0.653292	0.708729	0.700751	2.381074	3.055771	14.3504	3.50363	9.893436	4.640199
Cs1g21270	0.263811	0.228248	0.562474	0.449731	0.5074	0.599611	1.032131	0.384247	0.856166	0.783618
Cs1g21280	15.95185	15.26368	11.36125	15.63998	5.594881	10.08876	9.455708	13.88419	11.23095	17.73673
Cs1g21350	0.701847	0.693054	0.61674	0.671501	0.405909	0.662727	0.233009	0.2018	1.544494	0.533053
Cs1g21370	4.26161	2.948306	4.101434	3.996286	1.598626	1.217356	0.704281	1.495559	2.807029	2.545447
Cs1g21400	8.768137	4.099634	7.766136	8.377779	5.76597	8.647355	3.691855	2.639911	3.002725	2.199074
Cs1g21600	32.3249	26.41895	20.56491	22.60105	18.8233	39.62013	56.66549	49.06427	3.006287	1.014474
Cs1g21610	0.348059	0.35023	0.509863	0.233144	0.214636	1.138385	0.88102	3.092648	0.287919	0.776536
Cs1g21640	10.24878	7.493854	18.0866	12.73823	29.68854	24.67907	46.38678	17.35829	14.21174	10.27582
Cs1g21660	24.81738	26.96634	20.81768	23.58046	9.912605	11.41867	7.242256	9.979168	5.958944	6.002981
Cs1g21680	11.66967	9.369434	24.39245	22.49025	27.61579	31.01421	23.48331	38.13309	7.668088	23.94607
Cs1g21720	0.86108	0.964952	0.948756	0.933806	0.763211	0.561474	0.625687	1.867477	0.071112	0.06135
Cs1g21740	3.833708	1.678308	6.043608	3.667731	13.46163	25.72059	15.91373	31.14897	21.11896	59.0797
Cs1g21750	15.47684	8.530203	7.908947	6.775751	7.747496	10.38598	5.944676	5.877413	4.430706	1.148123

Cs1g21760	0.947597	0.28492	0.317371	0.228188	0.121688	0.216899	0.097022	0.098367	0.111608	0.027275
Cs1g21810	2.727869	2.610565	2.522971	2.766052	2.179773	6.225451	1.40491	2.296855	0.661896	0.575168
Cs1g21820	14.0709	20.79925	25.95104	28.34318	10.57086	20.5973	5.61778	21.64173	2.959162	7.893733
Cs1g21830	16.39159	30.98433	8.686445	9.417126	5.679439	14.27882	4.318922	12.69526	19.38868	20.72536
Cs1g21870	6.236773	6.921078	2.391324	2.843324	0.810327	1.569196	0.020865	0.525608	0.172562	0.06002
Cs1g21920	4.597914	4.365589	3.039251	3.371864	0.96096	2.814353	0.309472	0.818343	0.036433	0.034662
Cs1g21950	14.28904	23.99167	12.00538	16.90062	6.517165	6.163409	5.579256	8.839023	2.339284	4.744922
Cs1g22010	3.969142	4.287105	3.573436	2.929657	2.713133	2.137979	0.954048	1.569626	0.367524	0.610017
Cs1g22050	28.32444	30.10108	13.19649	21.9705	3.378649	6.561863	8.634081	17.29216	4.157719	16.76659
Cs1g22140	0.040838	0.108466	0.063569	0.105591	1.13743	1.702235	28.07085	32.45556	10.27889	46.33253
Cs1g22210	1.163521	1.640151	2.435426	6.767419	0.056896	0.424037	0.399251	0.692284	0.867313	0.146461
Cs1g22230	17.74308	22.96869	11.94053	16.54742	8.911101	9.413359	8.286485	14.86591	15.02132	16.08785
Cs1g22240	26.25932	17.63027	29.03452	26.3227	57.47079	62.74812	68.94167	137.7285	145.2589	165.3739
Cs1g22270	15.98252	15.30869	19.94505	20.02212	14.71661	20.02569	9.045344	20.7281	15.83918	22.90723
Cs1g22290	37.93788	41.67026	29.52001	25.6073	35.79613	21.84688	44.95275	17.78021	18.62147	14.13636
Cs1g22320	0.443253	0.794581	0.543724	0.657138	0.802536	0.5339	0.227517	0.787426	0.324946	0.749585
Cs1g22330	51.75504	55.01594	75.47861	53.80641	49.34598	45.44907	21.49745	19.71205	5.525386	9.268665
Cs1g22350	4.107214	2.127796	6.534861	4.433624	1.172744	1.709148	0.610569	1.769286	0.998039	1.291997
Cs1g22360	15.53228	10.35317	16.30259	17.01335	4.765682	7.634798	3.222817	7.995326	5.761819	7.182817
Cs1g22380	0.568215	0.316408	0.485596	0.557691	0.252541	1.365331	1.090559	3.357162	6.958368	10.0952
Cs1g22410	19.55575	9.93567	25.09375	14.44672	6.251142	8.044959	2.905916	6.910229	3.460223	5.490411
Cs1g22430	6.817793	6.801081	9.194921	9.135448	17.30852	14.57853	37.4244	30.21483	2.841823	3.559361
Cs1g22470	1.047552	0.961055	7.313019	5.367005	6.300018	7.690915	2.671796	5.906845	1.481009	5.20213
Cs1g22490	2.925874	2.08365	3.605234	3.251893	2.147019	3.258266	2.087063	1.245426	4.190576	1.805594
Cs1g22500	9.594862	7.451037	9.292674	8.487699	14.02816	29.47109	16.38962	21.0969	3.661563	4.369582
Cs1g22540	9.309937	9.2442	45.19462	53.99819	7.337734	10.10821	54.8726	46.14116	38.53592	66.11531
Cs1g22550	4.919602	5.089763	3.308912	2.313522	0.435273	0.793544	0.467632	3.524515	0.107839	0.121008
Cs1g22570	3.233509	3.212813	16.52045	28.83804	0.535869	4.10519	2.949668	15.24535	1.854466	3.28599
Cs1g22610	6.061463	6.254264	6.356283	6.419282	4.142981	5.784116	5.669639	6.307541	0.568434	1.648042
Cs1g22620	35.18507	59.21121	17.63904	24.58592	37.26253	41.73637	66.26522	99.42104	48.09352	60.38253
Cs1g22630	8.475332	6.52603	8.780814	6.954565	5.977155	5.512389	2.928842	1.962647	1.017946	0.960387
Cs1g22660	23.76696	26.26129	46.86026	39.1777	35.35613	35.3033	80.43462	69.00605	79.66545	63.47409

Cs1g22680	2.809237	3.154656	24.52855	18.40098	105.8176	97.78336	176.2429	174.8746	262.6236	231.5987
Cs1g22750	0.007428	0	0.372712	0.78402	0.62935	0.943722	1.858858	4.784013	0.149875	1.23633
Cs1g22770	1.999113	2.013768	5.357884	5.104201	9.028095	9.881837	6.972052	12.90704	22.43844	40.56912
Cs1g22780	29.66791	34.23555	27.79985	28.46085	14.20749	13.73999	9.559516	32.62489	7.652442	12.3701
Cs1g22910	1.463082	2.060589	1.63044	1.019859	3.323289	2.462311	3.697839	3.000039	1.089904	4.573948
Cs1g22965	0.862896	1.273178	2.48234	2.242577	2.031478	1.143906	0.465272	1.8555	0.260719	0.507367
Cs1g22970	12.79228	15.94503	10.76508	13.40886	11.88335	11.40468	6.510508	13.82936	5.226104	6.449296
Cs1g23040	1.570589	0.368033	0.015957	0.083373	0.115199	0.238278	0.023013	0.013879	0.218673	0
Cs1g23060	45.61263	49.95593	38.34328	36.26505	17.74408	17.67357	5.408855	8.339668	1.342608	1.979615
Cs1g23090	6.754252	3.12137	1.644135	2.046282	0.550964	2.357756	1.027631	3.371459	0.683981	0.564239
Cs1g23130	7.804097	8.768546	6.410863	8.269524	4.877794	2.766921	2.383057	2.65957	1.322241	1.212752
Cs1g23150	9.414323	4.901474	2.004181	1.673309	1.81444	2.448554	0.839995	1.238792	1.813801	1.465508
Cs1g23160	410.577	244.8435	52.92771	42.04048	64.1774	52.48981	79.36556	113.0928	7.281598	37.50593
Cs1g23170	458.7567	277.6827	60.17262	49.11629	70.43274	58.89712	89.01097	123.0513	7.342768	41.84666
Cs1g23190	6.303175	3.574147	1.510635	1.576124	0.940552	1.437801	0.908436	0.610545	1.825908	1.965928
Cs1g23200	716.0819	495.1264	86.93238	65.25443	105.6724	86.40614	143.2332	212.9055	10.79351	53.80506
Cs1g23230	24.83941	17.85848	25.06876	16.05255	7.658613	7.484518	1.089997	2.149362	0.944869	0.149239
Cs1g23370	1.058231	0.57868	0.503809	0.419826	1.766135	0.791852	2.053404	21.63045	31.79336	25.71765
Cs1g23430	2.939504	2.463184	1.893247	2.123047	0.984967	2.676785	3.041417	3.494892	1.003302	0.467146
Cs1g23450	170.1257	244.95	77.45797	117.5769	28.19024	36.27636	12.70338	47.79839	6.628447	18.21389
Cs1g23460	1.713613	2.630257	3.44361	4.167221	3.533825	3.94566	2.312032	9.93893	2.283198	4.286564
Cs1g23470	0.326082	0.28597	0.936134	0.820103	1.260917	1.536992	0.829825	1.262395	1.302915	1.382386
Cs1g23480	0.281585	0.360265	0.598209	0.728615	0.040294	1.052795	0.435436	19.4859	0.041551	0.282052
Cs1g23520	9.709381	10.51863	7.72154	6.653984	5.004024	3.822047	1.975692	1.044624	0.432521	0.845165
Cs1g23535	6.525364	8.808148	8.506253	6.758611	8.756109	7.466232	6.601272	8.421311	12.91057	2.597806
Cs1g23570	8.602676	10.29407	9.559775	10.05384	4.990685	7.51276	2.515328	8.962556	3.405662	4.803904
Cs1g23590	12.078	18.05901	28.02088	30.17607	1.766509	5.849319	2.211265	17.74359	1.43714	10.25737
Cs1g23600	0.287383	0.622936	0.07263	0.168488	0.175767	0.537689	0.212669	0.894164	3.574585	15.09438
Cs1g23610	10.54432	10.5797	4.233649	4.767986	2.382988	3.041953	1.767709	0.99202	0.148006	0.035177
Cs1g23630	100.2443	81.61053	89.87752	112.3491	64.46902	48.1808	51.13597	21.80045	33.6665	23.33768
Cs1g23670	145.3216	120.1634	222.1265	212.9639	183.7438	296.1825	218.5179	94.16278	441.8466	237.183
Cs1g23680	17.71546	24.62836	16.42949	19.2875	12.47294	14.3725	7.261786	20.00539	7.744639	16.27379

Cs1g23720	28.68187	24.31912	55.64682	37.01845	48.26061	49.4075	13.36661	9.461671	0.939152	0.878331
Cs1g23770	18.03514	20.54968	11.07739	11.01397	3.748218	4.137471	2.904981	3.327485	0	0.047179
Cs1g23790	0.944173	1.815942	0.379161	0.333423	0.515697	0.329315	0.649548	0.510887	0.124672	0.128403
Cs1g23800	0.313142	0.396426	0.331551	0.218172	0.721463	0.303159	0.451028	0.558137	0.615056	0.842579
Cs1g23960	2.857745	4.336576	8.591209	8.188412	4.581885	4.404064	5.453622	11.7313	5.158239	9.430513
Cs1g24010	1.406431	1.265736	6.676533	4.175149	6.16522	4.625305	1.384262	2.428346	0.976404	1.061996
Cs1g24030	3.544933	2.30574	3.042672	2.443141	3.485249	4.680422	7.744744	4.632273	1.557347	0.288819
Cs1g24040	31.58081	31.90611	38.47838	34.19464	42.52543	40.49928	15.86033	38.43179	49.2315	56.30741
Cs1g24080	3.064121	3.69737	3.287884	3.679525	2.895087	3.348239	2.437663	4.352923	2.32332	4.907137
Cs1g24090	4.950898	5.582975	6.938767	6.341879	8.412891	7.236389	5.898278	13.02209	5.049414	6.772912
Cs1g24120	7.495909	8.933826	8.913044	9.223208	7.405417	8.233553	3.908448	7.065454	2.025966	5.956739
Cs1g24170	15.50319	15.61158	23.60661	21.57699	14.58728	16.84371	10.624	13.55128	0.897844	3.982819
Cs1g24240	7.971	9.877956	0.793924	1.633878	0.104294	0.113873	0	0.372246	0.193585	0.760691
Cs1g24330	93.56973	106.2488	74.67626	96.05528	38.94424	43.65648	32.6434	53.96149	25.85713	44.18828
Cs1g24370	26.85645	23.50954	37.64688	36.94607	36.43077	35.75522	46.61842	18.62578	48.10165	60.34501
Cs1g24390	24.39634	24.65786	19.36242	15.69763	9.774817	14.00156	5.28378	6.317785	1.375535	1.291651
Cs1g24440	258.9277	299.2131	810.856	504.968	1199.048	696.1729	493.8435	105.2789	223.9515	159.3262
Cs1g24490	6.861199	7.473077	23.22227	21.71034	11.42308	15.45972	6.043712	14.39473	4.493425	6.024593
Cs1g24610	1.348621	0.439273	1.72051	0.482908	1.186263	1.471622	2.983868	0.706225	1.622846	0.138109
Cs1g24710	1.476665	1.714319	0.542001	1.381082	0.250765	1.112853	0.281028	2.806427	0.056882	0.227859
Cs1g24730	111.5478	75.21636	39.57676	26.11176	20.80064	12.32994	13.5075	8.96399	2.346635	1.015433
Cs1g24810	2.240807	2.75918	2.916274	2.242144	0.945898	1.11594	0.734359	1.274239	0.022466	0.041703
Cs1g24840	5.838833	8.691108	8.338898	12.30209	2.191581	5.749062	2.968443	12.67416	9.316671	34.03204
Cs1g24850	17.3058	20.75527	9.681009	7.700477	10.04536	3.522262	1.46612	3.206593	0.525117	1.027702
Cs1g24860	0.720247	0.684859	1.515018	1.482107	2.714408	2.895087	3.40948	4.298638	1.731794	4.092435
Cs1g24880	8.241292	7.230186	4.002083	5.684049	1.283049	1.418298	2.569836	2.906479	0.720709	0.951465
Cs1g24930	4.867966	4.437892	5.079435	5.416584	2.314255	3.958265	2.005563	3.036324	1.013967	2.73071
Cs1g24950	24.58876	23.76961	30.38958	26.14402	51.93573	51.95622	36.09415	31.3156	21.83171	3.683206
Cs1g25030	7.410479	8.777619	2.55414	2.551779	1.010893	2.637566	2.63739	9.654995	0.124321	0.011607
Cs1g25070	0.981308	0.575866	0.567749	0.353851	0.314099	0.630306	0.899008	0.326375	0.07113	0.021687
Cs1g25200	15.35131	15.13839	13.68937	15.51284	10.22078	11.80218	7.730611	15.8641	12.81033	13.29724
Cs1g25480	25.60214	21.69209	35.90137	32.3061	36.51648	42.04488	16.94459	16.80932	3.11132	2.044932

Cs1g25500	9.506836	13.84857	8.638658	9.707148	5.461409	5.872774	10.77082	14.27588	2.422795	5.702864
Cs1g25510	155.1241	181.3447	97.97092	165.2123	41.90517	31.81845	34.07973	48.15557	6.857452	11.20268
Cs1g25530	0.165607	0.120521	0.052493	0.102876	0.346821	0.696976	5.880062	1.556002	0.630068	0.31169
Cs1g25540	9.72995	2.704431	5.006026	4.952504	4.62778	3.022834	8.150512	4.290124	1.175935	1.339993
Cs1g25560	0.624325	0.459623	0.924073	1.475585	1.497004	1.718914	3.77973	2.094043	10.70161	11.22885
Cs1g25590	18.59858	20.87325	18.45889	15.41998	11.2816	11.85297	5.169122	10.86232	1.965876	2.895956
Cs1g25650	0.539388	0.677803	1.149115	1.087747	1.119936	2.26086	2.19121	3.782683	0.59022	1.673596
Cs1g25670	1.862212	2.306189	14.91577	12.32675	4.557623	11.19472	2.40802	4.525451	0.235713	1.919045
Cs1g25740	0.394753	0.312827	0.3677	0.204796	0.392776	0.144968	0.938656	0.100096	0.177766	0.051223
Cs1g25830	6.525911	2.365163	47.57383	31.33256	50.70571	37.10147	51.31454	43.9075	0.63507	3.482349
Cs1g25850	23.48878	25.74235	10.16268	11.47503	3.409491	3.733294	1.986754	7.127805	0.453455	1.068396
Cs1g25920	20.5434	24.09504	19.3815	23.28604	12.09861	17.38721	4.255647	17.71808	8.788388	14.21615
Cs1g26060	0.22176	0.513008	0.879391	0.772352	0.390305	0.967608	0.123819	0.247689	0.166844	0.271551
Cs1g26070	52.26571	75.16255	65.39484	69.21624	11.08459	16.46664	21.31358	59.44561	14.67656	41.95453
Cs1g26150	6.213257	5.963415	7.007713	6.754168	11.43446	9.052713	13.39788	12.50312	5.231838	4.535875
Cs1g26260	12.45287	10.70077	8.351403	7.373491	4.099494	5.094419	4.900653	5.316972	1.563546	2.311069
Cs1g26280	35.63111	27.82137	24.65569	23.09744	67.96262	58.15927	57.13254	19.29322	31.55477	35.07236
Cs1g26340	2.499294	2.090286	6.074082	6.390187	7.2423	12.38983	7.550415	17.98209	10.74468	14.12585
Cs1g26360	6.050882	6.775848	4.558594	4.675583	1.366673	2.238582	2.023518	1.316334	0.5966	0.489977
Cs1g26380	6.752221	5.812106	12.21518	10.57955	17.95303	19.52795	18.29449	14.61582	24.73959	18.32932
Cs1g26520	11.34621	11.59501	10.14558	10.52206	9.186922	7.412766	3.117346	8.887739	1.27922	2.550328
Cs1g26550	60.20809	53.03508	49.24565	44.8701	56.24226	74.5719	38.36446	80.78775	112.0635	164.746
Cs1g26640	0.242189	0.25133	0.868803	1.354532	0.231148	0.865126	1.169247	1.250571	0.418959	0.751945
Cs1g26650	35.70965	29.86936	92.38308	134.2513	40.43527	73.14132	100.7076	98.30384	110.8165	110.6089
Cs1g26820	0.45452	1.170672	1.733845	1.331456	0.544297	0.611737	1.030297	4.910974	0.29248	0.887432
Cs2g01020	1.059488	1.381032	0.857605	1.04381	0.399318	1.455296	0	0.653558	0	0.128639
Cs2g01030	3.546327	3.766766	6.006699	4.633484	5.13299	4.447343	2.389683	7.883826	2.203875	2.202151
Cs2g01090	36.26782	26.34696	28.98205	44.28105	9.238423	15.25553	1.286841	3.106998	0.50272	0.816491
Cs2g01100	18.93464	12.75999	15.19324	17.4838	8.196857	10.6715	0.793592	2.271368	0.272926	0.362365
Cs2g01150	5.56204	14.56195	16.10541	23.60104	0.596604	1.439008	1.226681	9.752586	0.070645	0.70528
Cs2g01220	1.177163	2.232055	0.874881	0.899008	0.173597	0.1684	0.163775	0.411115	0.023876	0.042877
Cs2g01345	0.348774	0.423354	0.494042	0.651702	0.817573	0.834897	0.740936	1.5535	0.690373	0.773781

Cs2g01360	14.86439	8.290063	8.853212	8.351684	6.340528	8.245268	7.954463	4.448857	3.443798	0.333938
Cs2g01430	63.14354	68.2665	38.94313	42.25361	17.46901	19.29243	14.54779	33.68648	22.38962	59.7281
Cs2g01460	3.795914	2.721186	3.197912	2.068248	1.646962	1.988584	1.926152	1.240808	0.150754	0.334056
Cs2g01640	52.63148	53.97501	101.8717	86.79402	59.31157	65.54911	30.89113	72.38297	21.91512	100.5406
Cs2g01750	40.8199	65.25764	24.49019	43.04747	12.54011	14.14822	5.146705	21.85905	0.874307	1.098394
Cs2g01770	11.02629	13.13186	26.59125	23.59006	28.43254	25.01155	18.79163	56.72027	27.42929	54.1381
Cs2g01830	3.856724	8.2693	1.883601	3.537473	5.054379	2.910229	4.093297	4.974484	0.298753	0.19503
Cs2g01840	37.80252	63.75692	106.3201	115.6896	186.3478	244.5748	209.3491	401.1026	36.60791	82.47475
Cs2g01860	8.920066	12.5144	7.524217	9.625008	4.748808	4.627365	2.858883	5.554457	0.180833	0.389756
Cs2g01910	100.0975	86.2256	109.2808	109.8332	53.73852	54.2226	27.61438	34.00801	24.61143	10.35612
Cs2g01920	1.003971	1.122229	1.093695	1.17987	0.952094	1.138321	0.748856	2.36218	1.20072	2.21377
Cs2g01990	67.49945	96.47092	88.00833	97.04367	31.74392	25.28029	63.05705	91.77567	30.55548	96.86273
Cs2g02000	2.943659	4.132963	4.297854	4.072447	3.898329	4.722682	1.235653	7.094088	2.698678	5.351274
Cs2g02050	4.979635	4.49435	4.458896	3.964667	3.264766	5.605832	2.392379	5.577385	9.04904	8.566731
Cs2g02310	3.028926	2.850756	2.905771	3.992588	5.460339	6.683212	15.22425	8.039585	1.628631	3.347278
Cs2g02410	20.74182	20.21691	27.13853	24.58285	14.57753	18.67702	6.975477	17.36807	7.410015	8.381393
Cs2g02450	2.614229	5.474374	4.926498	4.638089	14.32454	9.055184	16.45188	27.84937	36.59209	21.94511
Cs2g02460	28.78387	27.51238	68.1875	51.6836	94.96401	97.3482	95.35177	119.2613	60.94757	60.50753
Cs2g02500	0.184806	0.130456	0.681447	0.403051	2.100592	1.019132	1.24142	0.868486	0.946063	0.958666
Cs2g02550	1.998239	1.253692	1.609043	1.50296	0.528129	1.156135	0.423947	0.652896	0.058414	0.034341
Cs2g02640	99.94756	83.9356	53.8142	65.1826	34.20887	63.4823	29.14381	26.96264	49.3739	13.27065
Cs2g02660	0.672756	0.956662	1.481529	0.943875	2.704134	4.022692	1.478994	0.989001	0.980913	1.700106
Cs2g02680	1.818175	1.088904	2.226911	1.939738	3.741939	7.914631	2.215701	6.264665	15.26992	15.7859
Cs2g02690	257.5927	136.0349	63.32917	73.3565	18.37308	48.53735	25.61635	47.18825	169.1518	229.6309
Cs2g02790	20.09824	18.61418	30.14135	35.78793	17.13165	27.46259	33.64522	44.30236	109.5781	124.0111
Cs2g02900	10.21682	12.56045	18.41912	15.81078	29.46807	22.83867	27.45034	35.38911	25.46494	25.10782
Cs2g02980	34.5348	58.77502	71.05005	115.7965	47.15194	96.64283	161.2728	73.37379	196.9354	258.165
Cs2g03000	47.65033	49.46127	21.6628	28.06668	13.09402	10.25896	6.740625	8.993069	2.297914	2.5928
Cs2g03010	4.436073	5.353596	3.805633	4.268378	3.152919	3.716069	2.483757	6.791216	3.908167	5.795522
Cs2g03020	4.398684	5.186589	7.230899	8.355883	4.022501	4.053802	6.001092	5.924219	3.222351	13.13683
Cs2g03050	8.520985	11.40906	9.519272	11.35923	6.296072	7.223031	4.236022	9.923693	3.374895	6.796976
Cs2g03080	74.74805	80.61075	48.70609	66.20684	9.147041	10.55772	2.361067	6.150094	0.925562	0.470608

Cs2g03110	2386.736	2293.547	1126.053	1518.607	351.9533	478.3456	292.7926	277.1564	9.332069	6.867794
Cs2g03140	9.988848	4.563932	6.938192	6.850715	11.73814	10.92092	9.538077	9.228856	0.817687	3.01114
Cs2g03160	3.007015	3.656658	4.906379	5.280737	2.657767	3.192286	2.815406	3.537877	4.10075	11.27852
Cs2g03180	0.120838	0.091103	0.289591	0.56633	0.001401	0.300278	0.84675	2.596183	0.134389	1.303835
Cs2g03230	52.29002	58.51633	111.3134	88.27804	151.1454	88.60896	345.2306	318.5678	307.8998	388.9585
Cs2g03240	42.14103	45.89517	55.99657	115.5258	7.702211	21.88689	47.61132	139.0693	423.2635	439.6752
Cs2g03270	4.521657	7.710601	1.526906	1.897378	0.508581	1.797478	21.17983	6.676277	40.9201	20.53532
Cs2g03280	0.566793	1.016304	0.149983	0.172135	0.061155	0.156184	0.120326	0.464397	0.298081	1.068447
Cs2g03330	12.59646	27.17332	13.70033	27.87878	9.026666	19.64875	12.51698	24.21946	0.25827	1.020676
Cs2g03380	0.079757	0.009938	0.2216	0.183242	0.414928	0.436109	0.309373	0.066851	0.896471	0.194883
Cs2g03430	0.597726	0.470832	1.082438	1.301304	2.304699	2.75883	5.93794	4.680777	3.261484	4.030733
Cs2g03480	39.43394	44.00629	53.61795	55.79896	50.66646	63.20247	25.84935	61.3481	49.03832	59.65985
Cs2g03490	11.10865	6.405249	1.45299	2.241372	0.171141	1.158281	0.253502	2.200918	0.327667	0.475955
Cs2g03610	2.969782	2.093111	5.651989	5.012725	0.811657	1.594619	0.958832	0.717168	0.150505	0.145211
Cs2g03620	14.30157	10.09188	22.60423	18.50731	28.97163	28.02331	51.5087	34.52751	33.55148	27.15621
Cs2g03780	11.50046	52.23889	1.843634	3.374643	127.8817	113.2077	29.14244	32.56654	11.30966	8.061123
Cs2g03820	0.643893	0.690707	1.947865	1.775357	4.058096	3.74826	5.642514	12.02512	3.186417	5.640775
Cs2g03830	8.965999	7.43696	8.120894	8.748996	9.767455	15.99156	3.78206	9.424096	17.16451	7.171772
Cs2g03840	25.54605	12.66011	13.83159	10.9294	37.80165	24.40188	53.54734	15.69424	36.69368	5.553162
Cs2g03900	4.08891	4.889338	6.311273	6.081333	6.871215	7.060597	4.803592	9.715477	9.823099	13.57344
Cs2g03910	31.45434	31.41746	48.9119	46.00762	67.52392	83.09437	42.07679	61.25943	82.61216	70.6465
Cs2g03950	0.209883	0.26782	0.3271	0.23045	1.985291	0.756098	1.299048	1.058032	0.144576	0.37463
Cs2g04085	2.688225	2.07685	1.035165	0.836377	1.292665	1.278989	0.575363	1.973526	0.464547	0.348323
Cs2g04090	14.66572	14.84191	9.664169	11.41678	4.096315	3.073601	2.912135	3.240458	0.270116	0.15721
Cs2g04140	1.514344	4.513937	0.593257	1.372354	0.395446	0.607585	0.338921	1.435924	0.097572	0.070845
Cs2g04190	16.00445	17.99274	6.127479	7.245927	2.871303	4.056182	1.814949	3.137588	0.224714	0.113017
Cs2g04220	28.13579	13.97653	19.23083	16.87619	9.569787	26.61028	14.88523	23.96195	2.288568	11.67355
Cs2g04370	47.61168	36.16709	45.67167	48.05003	59.0781	68.42992	125.664	49.76346	20.74647	8.135054
Cs2g04400	15.2707	18.46174	21.31467	25.61338	13.11458	16.25839	11.37032	17.01903	5.650321	13.9767
Cs2g04410	0.319291	0.370321	0.729525	0.788924	2.335943	1.693582	1.328779	1.413408	0.238672	1.097962
Cs2g04430	27.14621	38.31019	36.38659	35.89453	12.85222	9.92067	26.39499	40.78124	28.24982	40.81501
Cs2g04460	0.188449	0.13179	0.500855	0.396174	1.013699	2.624666	0.851656	1.321995	0.215194	0.091537

Cs2g04480	0.053941	0.055877	0.062562	0.060193	0.274223	0.17558	0.74777	1.551211	0.067195	0.023858
Cs2g04490	7.178047	4.607479	15.27749	13.33714	10.29009	12.25391	28.35453	42.29339	97.61368	59.41449
Cs2g04540	6.963386	12.46829	29.66065	28.71845	12.04634	11.05172	6.735626	20.71595	25.14885	20.90503
Cs2g04640	3.989352	4.130712	7.61527	5.455853	9.240088	8.267764	5.48185	15.45534	12.56828	7.571396
Cs2g04650	0.49026	0.566184	0.798495	0.80756	0.514151	1.154856	0.536073	1.170743	0.21787	0.189767
Cs2g04680	0.772064	0.525609	0.679412	0.746425	0.539829	0.917092	0.616636	1.589484	0.425805	2.258975
Cs2g04700	3.057504	1.49138	2.834726	1.891094	1.263985	1.271664	0.847028	0.580202	0.101312	0.157363
Cs2g04870	3.678559	3.46096	0.678761	0.262578	0.529563	0.197634	3.041329	5.049266	0.479785	2.389445
Cs2g04980	45.54135	47.34099	6.021252	10.78207	1.07519	6.871935	0.549658	8.234292	0.81523	0.63382
Cs2g05000	1.898256	2.36047	0.390869	0.862214	0.15806	0.28024	0.058139	0.105988	0.014287	0.019181
Cs2g05030	14.1363	17.09872	3.879971	5.458375	0.629656	1.532975	0.674427	7.72081	0.315855	0.468381
Cs2g05060	1.536678	1.033645	2.602404	1.896624	3.198697	2.634793	5.244014	2.117076	1.911181	1.712851
Cs2g05090	32.10984	19.19937	31.00418	33.3731	47.92077	47.70241	28.76514	9.940813	6.178176	7.491641
Cs2g05110	8.521121	7.034623	12.56842	13.59862	9.325281	17.83015	5.209936	8.042618	5.686826	10.70133
Cs2g05140	17.06675	18.21824	23.732	23.19949	30.62661	30.09787	18.97384	34.80017	12.92626	27.68695
Cs2g05170	4.341975	3.699778	13.05829	11.08677	3.895729	11.50831	10.43273	10.69175	1.417487	11.64347
Cs2g05210	5.946528	3.926235	11.89617	9.889503	17.65491	19.64322	15.75817	19.71097	14.79116	14.23155
Cs2g05350	51.54319	38.50905	59.2427	64.52512	70.68693	65.92363	225.7994	109.3649	131.3672	132.4859
Cs2g05400	6.886493	8.096485	4.741464	8.969639	4.198469	7.496051	2.416857	3.871187	3.772105	3.585312
Cs2g05490	1.568217	1.662459	1.429422	1.149092	0.557356	0.725449	0.303983	0.930147	2.175178	2.066899
Cs2g05530	121.8607	173.3836	53.01792	80.25272	20.90212	31.40504	6.746591	20.51811	11.16386	14.12601
Cs2g05550	3.817711	5.008038	3.791052	3.677636	4.190597	4.267137	1.463341	4.921636	3.560273	2.505089
Cs2g05560	81.19437	83.38499	88.03024	91.59781	25.94416	36.92991	47.93179	31.33776	1.458711	1.632909
Cs2g05600	5.561558	7.19008	4.988066	6.263965	2.783329	2.777212	1.017728	1.496894	0.537204	0.794765
Cs2g05640	16.46737	16.58637	11.15953	18.87114	4.134601	6.790127	10.25694	8.434518	11.93361	8.01669
Cs2g05800	2.586458	2.629476	3.394774	3.984566	5.544068	5.885351	4.095874	5.094461	4.367159	4.994101
Cs2g05810	3.643267	4.954909	4.259176	4.116667	3.51274	5.507478	1.911238	5.89314	3.451868	2.98096
Cs2g05960	45.03378	33.40076	42.26545	41.12528	52.3157	101.8713	14.21057	13.27629	25.18067	3.826697
Cs2g05990	6.356093	6.766402	6.403573	5.432874	5.044965	5.317493	6.295559	5.824052	20.63553	6.69361
Cs2g06010	8.425425	7.983956	5.146426	6.232325	3.18518	6.272002	4.640272	6.783719	14.89707	5.997435
Cs2g06020	4.080672	2.802344	5.239581	5.160708	10.95979	9.227972	12.1116	6.093636	6.558835	6.31458
Cs2g06030	11.20101	9.42661	25.99064	22.06512	14.07065	16.55647	13.43005	21.50291	18.75245	25.61112

Cs2g06115	17.2353	7.871572	23.92884	12.67675	35.46095	39.14064	18.11321	17.29533	18.55715	9.449623
Cs2g06200	23.0211	26.90076	116.821	88.33638	159.3077	174.3135	288.2713	406.4524	54.24477	79.65685
Cs2g06240	0.900178	0.673233	1.041899	1.259292	1.195796	1.388464	1.717761	3.879392	0.673967	1.831174
Cs2g06290	13.12156	16.35186	1.452169	1.804181	0.999162	2.02014	0.395482	5.558468	0.481868	7.081817
Cs2g06400	13.24266	3.545074	10.21803	9.439509	6.914747	7.144889	2.250748	2.12913	6.821479	2.239856
Cs2g06410	33.16494	36.02811	33.46537	34.15757	24.17925	26.62123	16.76772	48.72916	24.70918	35.81631
Cs2g06440	10.72001	9.47513	8.304173	11.71279	5.638756	8.825605	12.54216	24.39349	3.065981	6.033151
Cs2g06470	52.50883	63.28777	65.58962	63.55274	15.4786	17.64217	6.344992	8.063139	0.649841	0.670197
Cs2g06550	1.001623	1.468025	3.538911	2.982815	0.649243	2.211381	1.857799	2.370404	0.055101	0.067708
Cs2g06570	2.158645	2.345912	2.313992	2.142323	0.990971	1.366132	0.445703	1.440741	0.101034	0.418494
Cs2g06590	4.093829	4.324264	3.273276	3.479288	3.030326	5.817204	0.855635	1.869095	1.835085	4.991386
Cs2g06620	197.776	140.9916	364.0473	307.7456	706.4333	577.4504	1929.747	923.4486	445.6539	520.9471
Cs2g06630	6.169253	6.59733	5.327829	5.337738	4.221802	4.451515	6.8412	6.475326	2.4016	4.87903
Cs2g06640	26.05283	14.44563	18.72491	31.58045	6.536462	7.9365	20.42902	11.67401	9.735065	34.98049
Cs2g06650	21.74993	24.30138	56.82162	106.6904	23.97652	129.5253	61.31047	46.34447	132.9187	162.0401
Cs2g06710	40.85669	40.27225	50.1846	42.09408	39.21817	29.01833	12.16374	32.23682	19.92147	26.22248
Cs2g06730	31.31816	32.48233	24.44706	18.40913	16.10914	8.048315	5.416475	12.61162	7.379427	16.25422
Cs2g06820	15.45448	16.13977	31.69759	37.28862	33.92246	59.70846	39.12852	54.21665	34.80542	38.46632
Cs2g06840	43.05094	48.565	44.14931	44.82271	13.24321	22.78832	18.77969	55.26487	22.27522	40.42029
Cs2g06880	1.061736	1.391352	0.65853	0.618782	1.220917	0.717203	3.525493	2.441544	0.53582	0.714027
Cs2g06900	14.33934	7.680124	16.02742	14.7261	10.07508	25.97272	13.09265	19.37293	11.24527	19.15945
Cs2g06910	0	0.020186	0	0	0.010894	0	0.208	0	1.070619	3.293469
Cs2g06930	0.997643	1.767265	0.517119	1.024874	0	0.057019	0	0	0	0.027007
Cs2g06950	16.38644	6.96421	5.254735	2.872115	3.822231	2.851694	5.502302	3.705828	30.18307	13.81786
Cs2g06960	159.8858	144.858	51.37118	62.5702	17.51378	6.043196	16.627	21.37872	17.0046	16.99604
Cs2g06990	0.250651	0.302792	0.525205	0.381371	2.02239	1.681109	4.086379	2.511573	29.72242	8.493041
Cs2g07000	0.027013	0	0	0.059802	0.132005	0.124167	1.07296	0.31173	7.369456	1.941814
Cs2g07040	0.784434	0.800506	2.795953	3.504577	1.109897	1.659478	0.820114	3.697308	3.241224	8.421052
Cs2g07050	3.589492	5.0002	6.880085	7.828899	1.529033	2.349544	3.308159	9.600238	0.390808	3.237868
Cs2g07080	2.422413	3.405047	1.210178	1.9329	1.627692	2.423596	0.471932	3.426768	0.135044	0.410205
Cs2g07100	3.180675	2.04859	7.497194	9.749664	1.129087	6.718756	1.625143	3.202979	13.98329	14.60062
Cs2g07170	19.68343	21.84471	31.70917	28.63481	34.65729	14.22195	9.059942	24.4315	31.74279	103.13

Cs2g07220	8.118148	6.702581	14.48149	10.66174	20.23822	17.96638	50.06985	21.72617	13.80299	11.02383
Cs2g07240	155.3229	131.4985	252.753	232.3363	314.3649	322.3922	216.534	66.83301	579.281	335.1167
Cs2g07250	96.99205	74.17811	127.6759	126.3591	53.83205	100.1744	79.02907	15.81866	59.69351	35.38082
Cs2g07270	13.45216	19.57547	13.99648	18.12367	18.90253	13.70275	18.31434	11.10911	38.85998	14.73673
Cs2g07280	23.65729	17.02563	18.1401	7.110013	4.110692	2.116911	0.717095	2.03958	0.051316	0.142333
Cs2g07330	124.3557	211.9607	27.74486	47.74223	6.043601	10.62888	2.449094	23.67253	5.053708	20.30643
Cs2g07360	2.948171	4.241906	1.331409	1.954559	0.936684	1.403957	0.382296	1.608584	0.402349	0.453375
Cs2g07390	13.76739	16.8639	14.27513	21.40552	13.33509	23.15254	12.42968	45.26023	35.29627	76.27759
Cs2g07420	26.41537	32.73292	24.49684	29.79284	6.756835	16.65824	4.754482	9.282514	0.646286	2.522256
Cs2g07560	26.40368	26.26469	30.55584	31.89323	27.56497	36.04889	12.61092	30.61932	33.85036	31.76106
Cs2g07600	0.738409	0.537462	5.133387	2.022861	33.14592	25.66898	46.53811	10.2966	1.957879	1.753946
Cs2g07700	146.6398	102.4226	239.4063	422.9351	65.00125	161.4968	166.9946	42.67279	1213.108	597.744
Cs2g07720	36.86435	43.80327	74.77792	108.5331	23.53226	41.34091	45.30102	80.40862	93.68143	234.8806
Cs2g07790	4.15169	2.837397	3.136321	1.984211	3.47268	4.24806	2.070622	2.113594	10.67257	1.754613
Cs2g07800	9.515758	7.765412	7.912667	7.744059	7.944885	15.2815	6.164974	7.571257	34.05278	13.06633
Cs2g07920	6.170137	7.730567	3.69834	6.982825	6.952038	19.73032	20.17187	22.87862	7.242331	9.704067
Cs2g08030	8.051502	8.014012	9.190422	7.112115	5.41802	5.565788	0.83854	3.743809	1.492227	2.758269
Cs2g08110	64.46926	94.18809	59.53299	57.35506	51.74047	29.86188	23.36405	23.93783	1.672237	3.851769
Cs2g08180	4.072391	2.762213	2.480305	3.467701	1.036606	1.463826	1.379841	1.147163	0.130349	0.107486
Cs2g08190	162.9343	189.291	276.2031	313.8265	197.0167	201.7764	137.6203	342.2818	170.5828	314.6444
Cs2g08210	18.32344	28.6932	7.934172	7.510566	4.591354	5.14841	1.417955	4.35505	0.871746	0.982474
Cs2g08220	6.401934	7.421167	7.680632	8.172162	7.601384	8.767602	4.335151	9.355961	7.497754	8.148358
Cs2g08250	4.47513	5.304758	10.42025	5.905461	10.95194	4.436211	3.457578	4.459301	0.607953	11.54813
Cs2g08280	94.48265	68.88302	46.36398	45.58141	24.1487	33.99864	9.304907	14.63385	3.309125	2.883535
Cs2g08290	11.37136	11.31683	8.865541	8.761494	5.561483	8.31132	2.303384	4.785074	3.833587	3.407455
Cs2g08300	0.482303	0.419849	0.825006	0.89405	0.599678	0.904973	2.228578	0.845539	0.017494	0.042896
Cs2g08310	4.762375	8.566902	26.40631	29.38008	8.28135	17.28056	18.98151	25.89143	29.63655	27.94653
Cs2g08330	2.854819	2.970404	2.713127	3.18042	2.574909	3.133026	0.840622	3.225036	2.054851	1.53867
Cs2g08450	0.245946	0.263164	0.377752	0.453203	2.148835	3.449109	1.458702	1.73872	1.021528	0.192714
Cs2g08460	5.704093	4.312831	8.409203	4.159524	28.2777	20.30451	119.4798	85.6965	168.6698	258.7509
Cs2g08510	7.916531	5.666252	15.66286	6.692479	4.558792	2.654648	0.553991	2.461054	0.037814	0
Cs2g08520	2.771557	1.752671	4.553917	1.93912	1.405525	0.931888	0.156339	0.832036	0.02808	0.0411

Cs2g08540	38.9928	27.78615	65.64768	35.06077	20.09609	13.3793	1.330243	12.16774	0.142166	0.114551
Cs2g08585	2.225595	0.992561	1.757178	0.859759	1.490557	0.941568	1.931807	0.514996	1.183118	0.061851
Cs2g08650	1.993484	1.856107	0.966341	1.704755	0.275959	0.877758	0.692354	0.263381	0.298809	0.2076
Cs2g08765	3.286156	3.081421	4.849162	4.807348	3.271842	5.769799	1.473855	5.089792	0.774044	1.102533
Cs2g08780	1.394495	1.765652	2.616004	2.292221	1.611438	2.814297	0.872833	2.669381	0.261275	0.464446
Cs2g08830	5.941327	6.881351	7.933726	7.422525	5.453932	7.330285	3.209559	8.563042	2.089497	3.337576
Cs2g09030	8.139173	8.731821	12.44058	12.96121	9.630327	12.86652	4.491028	13.04578	2.580709	3.970626
Cs2g09440	66.62478	55.30259	48.27743	50.98056	40.10735	49.31122	41.17912	29.65157	44.12455	16.34042
Cs2g09450	36.54645	31.35109	35.32862	27.23421	30.15684	35.1004	11.29084	9.719617	86.66452	16.59294
Cs2g09460	7.686856	6.060082	9.04486	8.819222	13.20599	22.9981	12.35774	10.71104	30.89379	14.44505
Cs2g09520	38.46995	145.3132	9.07663	19.45456	7.730056	9.263976	1.928068	18.15179	2.188463	2.074853
Cs2g09560	1.024469	1.274544	0.646627	0.353794	0.498851	0.282	0.383168	0.088646	0.687395	0.31182
Cs2g09600	0.716585	0.495227	0.642805	0.425485	1.308071	1.514259	7.616312	3.420741	7.004656	6.140872
Cs2g09770	1.805576	2.180831	1.206075	1.382683	1.280397	0.8596	0.806851	1.043561	0.315047	0.752078
Cs2g09790	58.12084	55.64997	100.5322	84.99681	113.8263	95.85229	244.77	115.6698	92.49437	96.1675
Cs2g09820	5.528317	5.394794	4.29432	3.837836	1.809154	1.887515	0.49548	2.442912	0.216769	0.048433
Cs2g09840	2.254365	1.898509	0.137051	0.282646	0.030472	0.106295	4.120633	3.395075	3.961441	6.319835
Cs2g09850	4.667749	3.830117	2.452214	2.272251	1.750438	2.996957	14.22162	20.58669	70.93374	113.9309
Cs2g09880	21.63056	23.20804	23.96076	19.94264	22.37717	29.30228	8.197832	23.06957	33.86147	36.50951
Cs2g09900	3.369241	2.78236	3.133669	2.271719	1.570833	1.623042	1.318679	1.062503	0.346302	0.467259
Cs2g09910	39.15284	55.58505	21.9751	24.76613	11.64337	13.57849	7.715495	23.70635	12.73609	31.58928
Cs2g09980	57.52892	95.24148	131.266	136.1529	19.989	27.04172	46.71233	101.8435	49.55268	83.3012
Cs2g10010	4.412398	4.332564	2.635319	2.833114	0.842179	2.04661	0.413035	1.292244	0.108823	2.143185
Cs2g10035	4.96022	4.974794	2.949016	3.189061	0.958995	2.284252	0.469559	1.512672	0.124099	2.454469
Cs2g10040	1.965088	1.48831	1.470035	1.374557	0.461836	1.232288	0.34961	1.84972	0.043648	0.772059
Cs2g10070	2.903499	3.283585	12.98512	10.68023	2.504561	3.244006	5.028428	6.297638	0.143729	0.082284
Cs2g10080	62.93451	54.01035	70.68422	87.07271	40.44719	104.1561	98.37	99.37666	366.1228	279.8087
Cs2g10090	17.10796	8.198632	11.4493	9.278612	21.59313	42.62047	31.06239	23.39071	22.73215	5.615756
Cs2g10140	0.018186	0.064811	0.174398	0.674478	0.31416	0.131346	0.735912	1.805067	1.144885	2.211811
Cs2g10150	0.461992	1.242188	1.511079	1.949308	0.950865	0.950761	0.619834	0.711105	0.010205	0.112102
Cs2g10160	10.95011	13.19391	29.50812	31.48838	7.378379	18.7118	22.60419	23.9544	6.183017	21.90035
Cs2g10200	0.041201	0.165805	0.04968	0.067943	0.044142	0.024382	0.873215	2.733482	0	0.219893

Cs2g10290	16.86491	20.88136	12.13294	13.45695	5.940546	7.11791	1.187978	3.373492	1.294899	1.545208
Cs2g10320	3.141576	3.05492	3.535804	2.299725	4.95805	1.812016	3.24285	2.443801	0.376697	0.189885
Cs2g10360	29.7774	25.31998	39.68836	37.8233	62.0783	70.77716	48.61111	50.10065	94.36551	62.08725
Cs2g10390	0.365466	0.578019	0.567321	0.830547	0.320982	0.580848	0.274459	1.122227	0.598566	2.136624
Cs2g10440	25.68558	13.68549	21.62382	24.45804	27.88354	29.75322	16.73843	25.27361	25.77064	8.792605
Cs2g10480	9.132889	5.116802	1.675867	1.772946	0.353646	0.997463	0.172712	0.331061	0.153158	0.056552
Cs2g10520	18.20161	17.3807	20.91487	17.9279	27.91766	16.12863	17.25588	21.161	9.179053	4.445923
Cs2g10525	29.1467	21.46785	13.47795	15.5878	5.942043	5.73643	2.382958	4.390739	1.690101	2.706217
Cs2g10530	43.17219	40.05273	24.20616	34.85495	12.35623	15.00128	4.341682	11.09832	5.065329	9.27212
Cs2g10550	19.41037	14.74701	31.04488	27.14184	29.05727	34.83548	8.150572	13.77738	13.41195	12.9843
Cs2g10570	3.836464	3.640442	7.326724	7.154124	5.933597	12.19381	16.03553	7.319585	102.2107	30.04097
Cs2g10580	6.591544	5.232865	3.333593	4.487626	13.98185	13.18944	66.74964	26.19113	91.37054	59.85659
Cs2g10620	154.747	228.4373	83.19283	116.1752	40.1252	47.90751	27.1607	89.01689	22.32533	52.63375
Cs2g10630	8.245114	4.290917	55.13082	23.19464	98.66721	101.2061	34.30435	30.47287	57.34103	25.42754
Cs2g10690	286.472	352.5426	357.4547	395.2324	141.1307	202.2785	69.8904	137.3371	66.42284	97.84917
Cs2g10710	13.3884	18.31881	16.33836	16.42035	21.82332	42.1463	20.54038	38.11255	34.86681	16.41487
Cs2g10720	0	0.008869	0.052211	0.074618	0.529545	0.669672	3.030565	1.922451	14.93613	46.11809
Cs2g10740	0.941917	0.876309	1.809919	1.207261	1.259695	1.993626	1.967928	4.014254	1.960205	2.810761
Cs2g10760	12.93019	12.77387	10.87347	10.86508	3.780103	5.791556	9.205532	9.062047	12.82509	18.08432
Cs2g10970	6.2958	7.27368	11.67057	11.64213	7.507708	8.026052	5.34175	12.75561	7.273874	13.87049
Cs2g10990	3.09457	2.403241	5.211334	3.797393	14.62195	12.47297	39.55403	16.15123	6.016743	5.024239
Cs2g11110	1.572567	1.355526	0.874862	1.174795	0.371694	1.032484	0.679114	0.641811	0.03433	0.022529
Cs2g11130	0.148384	0.103865	0.583141	0.315754	1.610603	1.359396	2.553001	1.058376	0.700026	0.434881
Cs2g11140	0.1263	0.057172	0.504992	0.159391	3.828258	4.431266	3.205881	1.707307	1.071089	1.530361
Cs2g11160	33.17071	26.49902	69.45739	64.89524	126.8545	119.899	99.99994	107.7287	63.60835	56.63716
Cs2g11200	32.97937	39.50553	28.88946	41.32062	14.65865	16.33337	10.54232	27.14904	9.093488	23.29647
Cs2g11250	12.52424	21.02253	19.05077	30.07569	4.587797	14.59754	12.46822	28.06735	7.688907	19.53433
Cs2g11290	16.47473	19.55455	26.12687	29.28814	20.84238	40.04547	6.284691	17.73387	14.69191	19.60354
Cs2g11520	1.754766	1.975845	2.742671	2.916739	2.040756	5.713637	1.111391	2.097259	0.354915	0.161614
Cs2g11560	0.73264	0.567846	0.778288	0.278849	0.605451	0.467436	0.896065	1.941625	0.029858	0.037132
Cs2g11590	8.782024	10.84436	8.815132	11.117	14.62568	17.19766	11.46369	27.95614	25.75231	30.53389
Cs2g11620	0.294995	0.628861	4.891231	3.973649	62.58946	37.90457	86.54745	50.94598	0.110279	0.093585

Cs2g11670	10.46309	11.80103	14.65402	13.10033	20.38112	20.85373	8.613827	21.9019	17.20386	14.50408
Cs2g11750	11.17146	10.17578	11.82216	11.38766	14.54109	14.27465	15.1717	60.51056	169.5345	89.87989
Cs2g11760	4.596618	4.059333	8.499733	7.169585	6.599135	7.818365	3.682451	8.624374	16.10082	11.20033
Cs2g11770	6.36722	7.687897	16.89827	13.19312	13.75198	18.15436	13.24422	17.11317	2.282361	5.891653
Cs2g11810	0.871098	0.550504	1.441478	1.000987	1.04571	1.239561	2.41814	0.829416	1.242478	1.063495
Cs2g11840	17.69442	20.10939	31.54378	39.06757	40.6521	38.93015	18.02218	22.74495	44.27997	36.23443
Cs2g11930	0.175398	0.185784	0.136675	0.107402	0.089991	0.074998	0.127563	0.136622	0.106039	1.641561
Cs2g11980	7.916211	13.43402	4.72636	9.192393	7.211145	5.955635	5.79171	10.68268	2.282162	2.714849
Cs2g12010	50.19333	72.76068	79.43244	81.49404	45.00398	60.1212	43.36991	126.7409	64.60794	175.0427
Cs2g12080	122.4077	82.81915	41.25119	44.42822	32.3846	38.73002	6.334409	9.746499	200.1339	99.74876
Cs2g12100	20.16272	27.84738	16.69595	17.36154	41.90936	42.89703	67.7046	80.94641	37.13309	46.60385
Cs2g12130	60.66931	43.26136	46.34787	63.80697	23.23	32.50885	23.42228	13.50497	26.62455	25.53956
Cs2g12140	5.68601	1.85359	2.997856	3.153526	1.952063	7.192328	1.276654	0.599912	1.128583	0.408077
Cs2g12150	5.056473	5.42205	6.188376	6.112394	14.72563	11.85822	19.5275	12.08895	6.624964	6.356206
Cs2g12180	3.435439	2.612857	6.752145	7.388752	4.419651	3.937303	2.501093	4.187818	5.540471	11.62979
Cs2g12310	49.28259	45.8729	35.21497	39.29005	26.62609	27.89086	19.36218	21.39271	2.540042	3.675207
Cs2g12470	1.340935	0.955956	0.562865	0.642605	0.211335	0.383612	0.474531	1.123105	0.062028	0.048835
Cs2g12510	7.811739	7.877418	7.13591	5.650864	6.651678	9.229899	2.577796	3.662352	2.049104	1.318052
Cs2g12650	1.778403	1.095667	0.388477	0.956832	0.854257	3.571894	0.501912	0.869746	0.923208	0.218386
Cs2g12660	1.36807	2.128948	1.373776	2.025258	1.35638	2.25386	0.315714	1.914896	0.187142	0.762497
Cs2g12670	84.02838	120.0054	74.83538	90.15343	60.49053	53.85893	24.60425	88.16922	18.66055	31.89588
Cs2g12700	0.233005	0.207028	0.928457	0.545458	1.170907	1.038035	0.859964	2.91182	0.012042	0.023621
Cs2g12740	2.159963	1.892503	3.423732	2.670326	1.78404	4.949616	1.501645	2.783872	5.785488	6.09763
Cs2g12790	17.27398	10.51774	11.16542	9.529909	7.50846	12.60371	5.239046	4.45719	2.682457	2.241672
Cs2g12810	0.995102	0.88266	0.900151	0.994454	2.255674	2.093146	2.540596	2.141985	5.258816	10.18648
Cs2g12850	7.6598	8.150538	25.60958	30.25437	13.71832	27.66252	15.35106	29.11438	27.38178	37.81712
Cs2g12940	26.56593	36.8477	18.56661	24.4932	7.11643	11.97506	9.253775	21.31608	9.321831	19.40769
Cs2g13040	4.177309	4.163119	2.648229	4.348945	0.427064	0.748278	0.099776	1.22202	0.01953	0.256661
Cs2g13060	5.049953	6.423687	4.942016	4.908273	3.251074	3.962559	3.783412	5.708532	1.113487	0.33088
Cs2g13110	8.498171	8.626904	13.9872	15.0985	8.446822	12.47806	7.514256	20.24507	9.226591	23.89379
Cs2g13170	0	0.026936	0.240945	0.593544	0	0.278569	1.079108	1.809368	1.259332	4.473417
Cs2g13180	2.244903	2.380963	4.05774	4.968638	0.920095	0.73453	6.750616	6.934128	0.816255	3.694919

Cs2g13200	6.570799	7.032081	10.72453	12.71235	19.65756	15.08818	35.5732	17.35941	34.02211	17.84093
Cs2g13230	27.92877	23.24589	22.60404	25.38881	13.08378	8.221455	6.721746	8.607149	4.163786	12.38023
Cs2g13250	5.900612	4.820681	6.407679	5.242482	5.361373	5.972634	3.068891	6.765992	3.283463	3.121139
Cs2g13270	3.822926	4.719071	5.825998	7.366856	1.270792	3.543864	1.369044	3.408693	1.184433	1.018302
Cs2g13280	64.54716	46.18989	49.23912	53.12824	24.52173	65.29691	5.782223	15.39032	8.800714	4.268925
Cs2g13290	2.121824	2.684705	4.333218	5.763564	0.44336	2.288571	0.709168	1.161786	0.360239	0.284845
Cs2g13300	13.19516	10.80541	14.43724	17.26686	3.714422	13.51644	1.811911	5.131265	3.44838	1.802434
Cs2g13310	9.034052	7.482683	24.32182	25.92425	4.448457	13.20841	2.303659	6.217358	4.862303	2.881418
Cs2g13320	9.533096	6.143596	20.21487	22.77736	3.98448	15.92634	3.682122	7.942843	24.98478	17.55783
Cs2g13330	4.168075	3.837843	10.45425	12.58553	1.984143	3.406	1.198099	2.962857	0.014608	1.845617
Cs2g13340	4.113597	3.471212	9.042332	9.952717	1.925701	7.048284	1.394778	4.589034	6.524052	4.252598
Cs2g13360	5.465134	4.95585	13.20572	16.13967	2.586839	7.544211	1.57985	3.938505	3.141029	2.397843
Cs2g13370	0.818061	0.667739	1.651361	1.792319	0.339944	1.348272	0.356226	0.895773	1.13916	0.888161
Cs2g13380	2.136074	1.35953	2.954876	2.618943	0.408951	2.127529	0.392467	0.915915	1.904307	1.262064
Cs2g13390	72.05658	46.90269	56.85163	51.52875	31.68871	35.28634	30.83569	17.41928	25.31149	18.83798
Cs2g13440	2.035897	2.451188	2.720341	2.575099	3.03761	5.590807	2.170617	3.513263	15.97497	6.613473
Cs2g13450	33.49341	33.54757	16.6399	22.1924	1.635234	8.709283	4.866832	18.52005	0.472326	0.347405
Cs2g13500	3.402859	3.105008	5.565003	5.107133	6.924763	7.344739	10.3856	13.34454	2.683339	4.701158
Cs2g13610	18.53883	38.04292	39.62692	48.64364	5.242077	9.229167	16.68447	37.73775	10.43111	33.98846
Cs2g13620	3.542061	1.741842	1.689793	1.468603	0.674618	1.035955	0.97589	3.08212	0	0
Cs2g13720	1.273619	2.393085	0.662721	1.648178	0.070197	1.366523	0.416003	5.288996	2.509542	4.357874
Cs2g13730	31.62542	32.81109	21.49977	20.38195	14.03143	11.15296	12.20906	10.9552	3.712112	5.503632
Cs2g13790	73.27645	111.9922	13.96397	21.83788	30.07977	72.37368	29.31903	61.81686	25.09007	33.36318
Cs2g13910	40.49079	80.89671	139.9427	212.5951	22.52552	342.1691	37.69316	220.6238	18.09687	97.91656
Cs2g13920	12.35336	15.64698	19.70931	33.11585	5.07029	16.34572	8.478153	28.45044	160.2252	230.7494
Cs2g14110	56.15441	34.31079	123.074	89.66753	152.7739	165.9103	177.0921	155.0185	83.50492	71.83408
Cs2g14120	8.983659	5.355561	17.05772	12.81713	36.35727	30.61124	50.91618	44.86447	25.50519	27.23123
Cs2g14170	46.24474	34.57125	40.58358	51.664	15.00834	14.84024	15.81703	12.00989	44.52003	60.4446
Cs2g14240	35.72318	23.68666	15.0076	17.56414	7.519312	10.88	17.75992	16.71476	69.11428	73.35212
Cs2g14250	4.640499	4.453114	5.228296	2.713044	3.877647	2.181994	1.064648	1.994748	0.014139	0.07869
Cs2g14270	22.21169	15.35359	12.07525	9.385266	12.64982	13.9359	15.26494	7.419841	14.15412	5.984159
Cs2g14350	60.97452	45.31867	100.1692	94.76765	164.3641	185.455	220.8762	101.188	301.6374	194.5246

Cs2g14380	0.753044	0.41853	0.532431	0.504176	0.851219	0.238083	0.530709	0.619049	0	0.087342
Cs2g14490	2.503807	0.938865	9.314977	2.511914	60.11839	41.94189	3.101044	1.863935	0.67343	1.06872
Cs2g14523	33.36946	33.10274	29.2772	33.15065	16.04346	19.82953	7.439066	19.65614	8.257735	20.67408
Cs2g14526	28.0001	27.18568	25.44638	27.21675	12.96281	15.23963	8.827381	13.75085	5.124509	14.96639
Cs2g14530	21.27764	18.73586	17.83704	15.79324	9.022462	9.139937	5.52465	7.0175	3.753803	5.989378
Cs2g14710	6.295639	8.193376	7.266411	6.628784	3.183117	4.186687	2.100578	6.677413	0.894865	1.671688
Cs2g14715	2.954182	3.293986	2.911548	3.424057	4.073739	4.864778	1.337009	6.58338	2.932493	2.733194
Cs2g14720	77.32916	81.08521	39.44926	74.03418	15.01739	9.704475	13.71673	3.489911	1.172717	1.372749
Cs2g14730	10.09549	7.999907	7.549683	6.813005	3.555211	4.403421	2.887055	3.42617	1.389458	0.805674
Cs2g14810	0.790403	0.307447	3.634871	1.660323	7.386669	7.57724	5.436768	3.145394	2.330885	3.328953
Cs2g14850	0.946085	0.894029	1.62218	1.435615	4.475993	3.968217	5.221605	4.971892	3.710844	4.300679
Cs2g14910	22.47327	24.13533	26.16617	33.37343	10.23537	12.53377	7.436227	21.03246	15.20144	48.47275
Cs2g14920	2.367396	1.310424	5.297171	4.581495	3.708242	6.386706	8.275921	16.4605	1.110008	1.995576
Cs2g14940	167.0098	243.8302	65.80872	154.7572	17.00162	18.09042	7.368174	17.33677	2.350308	1.150453
Cs2g14980	0.00338	0.027838	0.007965	0.014234	0.094639	0.252428	1.459643	0.43791	3.5681	1.447707
Cs2g14990	3.291513	4.751649	0.903356	1.638245	0.507898	2.085738	3.152005	3.370083	1.468821	0.896509
Cs2g15220	3.723666	2.302337	6.558762	3.924595	7.0919	8.874163	5.008622	12.4244	1.826392	1.867683
Cs2g15230	20.33917	41.34615	14.38093	19.53805	22.69475	17.67321	23.18004	49.22295	17.11488	20.63075
Cs2g15310	0.606257	0.731999	0.267921	0.437054	0.147624	1.016644	1.619514	5.126474	0.120623	0.586616
Cs2g15360	140.9837	150.2432	97.00785	110.9279	44.2529	39.06399	46.55635	35.73539	32.30242	38.93273
Cs2g15390	0.225195	0.125215	0.73769	0.483782	0.872636	0.562137	0.364036	2.119715	0.839181	0.673478
Cs2g15430	2.291763	4.87215	0.529239	1.113738	0.326729	0.202738	0.250486	0.786546	0.238426	0.122262
Cs2g15520	11.98815	9.326109	18.28167	13.9579	34.35123	27.66894	53.6583	27.80548	57.25952	9.45487
Cs2g15600	0.916566	0.735401	0.888728	0.773696	0.349767	0.968072	0.334699	1.693627	0.034373	0.052232
Cs2g15660	7.022391	10.98187	4.623161	5.907789	4.92197	3.719828	3.756958	5.432108	1.825061	3.94434
Cs2g15700	0.022011	0.080711	0.43164	0.272903	1.128678	1.443715	1.395622	2.048356	0.030427	0.255297
Cs2g15710	37.81321	38.70635	40.90984	27.96231	43.40453	31.51207	42.83379	77.20465	7.89298	22.45245
Cs2g15735	7.438294	6.763055	10.88544	9.345625	16.97573	15.07206	14.86326	34.69275	12.21872	21.56872
Cs2g15850	3.436415	4.231631	4.30704	5.148978	1.879468	3.026426	0.585279	3.963711	2.210711	0.966257
Cs2g16070	9.170759	8.512305	5.047871	7.466904	2.958201	3.730828	1.51851	2.163699	0.439843	0.588018
Cs2g16140	1.975524	1.472754	0.962423	0.713509	0.471893	0.799383	0.304367	3.185623	0.412403	0.422343
Cs2g16150	19.61018	24.99901	30.52624	21.20259	21.2113	16.03801	13.68395	29.37752	13.45443	48.8815

Cs2g16240	1.032294	2.163355	13.16729	7.402341	41.90214	23.07599	44.60492	38.2	57.13594	84.70987
Cs2g16260	77.88871	55.33521	107.0011	112.745	237.1988	185.1941	243.0944	142.7415	163.1792	253.215
Cs2g16400	0.682781	0.687357	2.259299	2.408921	1.875761	3.612908	1.334062	2.264119	6.560755	11.73481
Cs2g16450	9.861313	10.38071	18.62882	16.13293	11.95302	15.96577	8.981375	8.83734	9.506176	3.8013
Cs2g16460	0.319352	0.812687	1.370216	0.91779	1.032685	1.518426	0.417801	0.651962	0.060594	0.129123
Cs2g16480	1.360705	1.615515	1.240362	1.575764	1.008935	1.456574	0.321759	1.433336	1.00027	1.222416
Cs2g16560	3.421342	1.276525	8.111369	4.295065	12.71593	12.49563	9.089597	14.64581	20.78453	21.18394
Cs2g16630	1.080533	1.185105	3.490417	2.854555	4.764886	8.814366	6.181338	7.876507	7.729044	6.330159
Cs2g16670	7.5261	9.626575	7.220038	7.345626	6.10573	5.568275	6.67811	15.65201	5.70084	10.036
Cs2g16770	2.391206	3.102964	2.699215	4.276263	1.601056	3.590784	2.192842	7.947878	13.4139	25.60799
Cs2g16800	8.591842	10.12471	16.46812	15.39511	7.51389	32.14474	5.152953	14.61612	3.70416	5.271476
Cs2g16810	2.432441	4.541996	1.515462	1.520685	0.183692	0.320481	0.34211	1.535382	0.086664	0.026916
Cs2g16840	3.676879	5.267458	0.487678	0.686159	1.330988	0.61956	2.382199	2.051623	0	0.02012
Cs2g16860	0.912862	2.039007	1.38758	1.468484	0.766627	0.753471	0.901122	0.521242	0.063449	0.061744
Cs2g16880	0.87306	0.413339	0.687075	0.391125	0.006903	0.020938	0.023932	0.028346	0.150682	0.031627
Cs2g16940	19.69836	19.67457	12.24601	12.04013	6.717024	7.270273	4.283115	5.303104	0.841305	1.474676
Cs2g16980	28.15648	25.98748	28.66704	26.28172	40.65938	52.15237	51.84854	23.8758	88.85744	41.05822
Cs2g17000	0.933988	0.605207	1.582098	1.067391	4.270927	4.56171	2.462915	2.852927	0.701341	1.942662
Cs2g17030	16.34705	13.29049	17.79633	21.08783	19.73102	47.39048	22.10943	26.65149	155.4425	101.1043
Cs2g17100	5.588011	3.595458	22.19892	9.077086	43.59773	61.40093	11.25889	8.576394	0.740238	0.342739
Cs2g17110	10.01441	7.051523	9.645723	7.608073	12.93234	13.11587	18.27803	16.60437	33.66424	33.37093
Cs2g17130	9.730184	5.389968	22.35912	17.21833	76.5617	46.39332	3.553924	8.623148	0.119315	0.072598
Cs2g17160	3.601083	2.869533	8.87478	8.447624	2.832347	10.42883	1.070039	4.862291	1.580175	8.895296
Cs2g17170	12.34576	7.954184	19.87131	20.69432	12.31546	21.57326	1.12349	4.520742	0.026155	0.064633
Cs2g17200	6.614611	7.36362	4.557859	4.439355	5.972182	4.652301	2.272645	4.901111	0.326238	1.045322
Cs2g17260	23.96494	28.06331	17.96903	19.21397	5.57604	6.610115	2.432735	4.662614	2.152047	2.074202
Cs2g17340	0.433657	0.772789	0.233853	0.292654	0.493929	0.390996	0.59043	1.730962	0.52431	0.848212
Cs2g17360	1.173441	1.046235	2.347853	2.121812	1.001497	2.311639	0.940281	1.139523	1.890168	2.848717
Cs2g17370	1.475303	3.478811	5.113713	6.285247	0.487669	1.460894	1.300643	12.32459	0.474605	7.709372
Cs2g17380	3.142886	4.145243	10.39742	11.39961	11.58599	10.53919	8.316258	9.110099	7.727551	11.83475
Cs2g17400	0.316906	0.620225	0.337365	0.418743	0.281087	0.420821	0.972082	0.103224	0.150491	0.33053
Cs2g17480	19.76838	18.1271	16.47587	13.9947	10.99227	12.13416	9.539133	12.28968	8.163956	3.236538

Cs2g17510	2.988348	1.206364	8.062813	3.564544	40.43805	29.11168	22.64091	9.966146	5.977639	5.066051
Cs2g17515	0.345555	0.308008	0.372072	0.390404	0.276359	0.267639	0.339878	1.046079	0	0
Cs2g17550	21.00838	21.32112	29.64939	31.33651	36.01473	39.74151	48.00409	81.23261	30.93589	56.96133
Cs2g17570	20.90235	22.30164	22.91803	21.27148	20.71948	14.13524	9.523256	9.674592	2.064738	3.666885
Cs2g17600	11.71912	13.94238	14.02951	15.97903	12.57794	10.7363	5.709587	7.495028	1.483197	3.691356
Cs2g17810	6.493612	10.59359	21.3164	20.01048	2.330053	7.819422	11.44888	23.45873	4.434786	19.6539
Cs2g17820	32.6908	32.56952	31.9132	35.57023	2.306444	10.93278	12.92853	41.94845	51.86914	106.5389
Cs2g17860	1.978622	1.115931	2.361852	1.189268	0.631685	0.339629	0.417566	0.458584	0.0469	0.026377
Cs2g17870	7.759178	9.537567	10.27955	10.42786	5.988615	5.569688	4.185347	6.264079	0.971969	2.421941
Cs2g17890	31.19516	32.67497	37.57853	44.00847	14.46709	16.01669	22.45751	24.65247	25.18679	67.35922
Cs2g17930	21.45443	16.66991	56.46388	44.18319	64.15381	62.64542	79.25788	79.39483	52.34741	48.52079
Cs2g17940	1.056396	1.444768	4.370421	5.146577	1.740248	6.678943	0.693313	1.834408	0.41098	1.781431
Cs2g17970	0.631113	0.830586	4.956761	6.007751	1.191969	8.62221	0.33799	1.026613	0.127734	1.455713
Cs2g17980	153.9035	218.7929	334.7408	408.3509	51.37107	132.3968	3.330494	22.0313	1.163586	1.461337
Cs2g18000	1.322369	1.819226	1.606112	1.619868	1.366608	1.996825	0.662477	2.450567	1.735716	1.522038
Cs2g18010	3.103418	8.460479	0.797736	1.185043	0.389417	0.53701	0.196534	0.565041	0.390279	0.371711
Cs2g18020	2.328867	2.566536	2.466466	2.490951	0.99109	2.136685	2.017624	1.988518	3.083086	2.927464
Cs2g18040	2.457329	1.634381	5.887876	3.916784	5.360343	7.342915	8.234127	5.173641	8.257627	5.857898
Cs2g18160	6.191933	8.483582	3.17908	5.111255	3.61472	4.842546	2.854792	6.425003	4.101567	6.906466
Cs2g18170	371.3867	632.5353	247.1893	280.8185	116.1131	116.708	50.85339	204.3724	54.08299	204.9072
Cs2g18260	1.060318	1.160445	2.363901	3.443173	2.320933	2.804293	3.829795	4.229677	46.48584	33.96254
Cs2g18270	0.518472	0.672461	1.150959	1.483308	3.922827	2.265453	5.818391	11.3809	62.40572	37.2667
Cs2g18290	1.41719	1.567113	3.199632	4.371984	3.725866	3.547321	5.920682	5.335095	52.1637	32.58579
Cs2g18330	6.430376	4.394804	2.669371	2.635574	1.934789	3.039806	1.738419	1.850816	0.724441	0.831533
Cs2g18455	1.613031	2.624277	2.883105	1.726717	3.606988	4.770874	0.940009	4.021337	2.930504	2.511736
Cs2g18470	2.147826	1.950394	2.12055	1.502544	1.686715	1.157473	2.023127	1.520955	0.932059	0.367528
Cs2g18570	6.942937	9.80872	46.07486	32.47049	104.8332	114.8106	106.9479	148.832	103.5886	146.178
Cs2g18600	1.619843	2.793024	2.640011	0.446914	0.175836	1.784388	0.114871	0.093522	0.379346	0.06939
Cs2g18706	0.170433	0.212719	0.433189	1.070707	0.120936	0.772681	0.019468	0.902091	0.037781	0.150173
Cs2g18720	3.065086	2.836072	2.304573	2.41699	1.197304	1.30209	0.980677	2.050383	1.118285	1.846887
Cs2g18960	3.82013	0.20043	1.687765	0.039751	4.53548	0.024657	11.41708	0.104137	4.866408	0.228962
Cs2g19010	98.90721	89.36272	104.6822	92.70794	66.49891	62.71313	27.94071	26.13464	14.70554	13.50761

Cs2g19200	16.78188	21.66554	27.26977	26.48319	42.35868	34.72427	56.32085	83.53852	56.90146	70.78655
Cs2g19230	1.928418	1.653172	2.359562	3.245829	3.202447	6.388745	4.196723	6.953378	12.64506	29.58185
Cs2g19300	15.35073	15.85728	31.12994	22.56099	36.20551	52.45119	20.80891	23.15419	3.481867	4.493882
Cs2g19320	24.03985	19.09445	48.45105	33.19274	57.89426	62.82469	37.64318	40.53597	13.08627	15.94936
Cs2g19350	17.94344	22.77288	6.57086	8.437136	1.356317	2.040781	1.641234	13.46848	0.297325	0.418183
Cs2g19360	32.32408	32.32377	20.30822	18.05302	20.15203	10.02512	10.98185	10.37734	3.129933	2.734563
Cs2g19370	12.40722	14.49725	10.12533	13.4228	5.742248	5.498536	3.684439	4.903919	2.028153	4.230173
Cs2g19380	77.35996	88.06705	56.01719	78.8187	16.49678	21.69763	2.381647	18.02661	7.240215	22.37355
Cs2g19430	39.17793	22.62847	30.34531	33.54035	38.81708	72.96659	15.00045	33.12288	4.23787	5.070086
Cs2g19440	25.10899	30.35382	14.45978	21.5187	9.999072	10.7236	9.065932	16.66893	6.657585	8.518509
Cs2g19470	6.266471	7.977938	3.858539	7.046435	2.550627	2.801035	2.402177	4.527134	1.855807	2.520351
Cs2g19510	2.056228	0.9393	9.451033	6.193121	7.076503	5.021614	5.381353	7.122272	0.964926	0.785399
Cs2g19590	34.61283	30.37796	31.45718	37.19846	20.89884	25.28897	11.33756	13.81048	13.62068	11.07835
Cs2g19600	1.046947	0.78274	2.336077	2.161019	1.732779	1.414154	1.066213	2.117928	2.909102	2.191918
Cs2g19630	2.117813	4.782565	0.556929	0.701816	0.355771	1.626304	0.67608	4.487531	0.19183	0.145714
Cs2g19670	5.407871	4.238066	2.110477	2.69988	0.680914	0.861816	0.938085	1.128879	0.559201	2.029871
Cs2g19680	24.83076	163.9035	3.912093	5.067101	6.131904	8.72308	4.705317	18.06213	7.350566	7.018682
Cs2g19790	4.160056	2.633629	5.840782	5.335767	12.11956	14.42475	29.55827	13.11585	2.123167	2.286551
Cs2g19810	18.02726	15.1289	18.05448	15.20644	22.29517	20.97471	34.75312	16.44215	29.90704	25.66595
Cs2g19820	6.15878	5.447709	7.528789	6.335045	13.60272	12.20455	22.51346	15.29875	44.60861	47.25244
Cs2g19850	1.215522	1.505676	0.224128	0.833943	0.008315	0.12038	0	0.025738	0.022827	0.012515
Cs2g19910	1.399814	1.38092	0.678729	0.740658	0.26702	1.166512	0.210752	1.471386	0.236992	0.05287
Cs2g19970	2.004655	1.829843	5.937838	5.79261	1.642071	9.8106	5.890751	7.961465	29.91974	42.87734
Cs2g20010	891.9685	807.5462	343.1252	667.2122	181.1456	131.56	141.9304	144.1726	12.63372	7.865949
Cs2g20020	6.34898	7.283797	8.001133	9.646112	4.78482	3.439793	4.272667	5.190152	1.961867	5.867554
Cs2g20140	658.3772	556.0814	267.2125	241.5089	286.6965	150.6959	48.5843	230.2227	0.581632	0.967337
Cs2g20200	96.75928	85.19215	102.9085	89.75885	133.3969	133.3601	44.06211	38.03416	8.512396	20.51167
Cs2g20210	5425.413	6580.359	1789.018	1898.19	1469.236	1804.365	1466.947	3145.092	192.678	523.3216
Cs2g20230	7286.762	8626.7	2364.048	2490.728	1897.056	2422.401	1926.75	4094.96	253.7816	688.4942
Cs2g20250	3.865145	7.696182	1.83327	2.674863	2.31296	3.002393	1.547483	6.763657	3.682792	2.610554
Cs2g20370	4.703895	5.30178	5.488167	5.126437	4.893304	5.445961	2.317892	6.384948	1.22762	2.18052
Cs2g20400	87.47124	71.79583	45.81204	44.07349	9.048461	16.66909	8.972631	19.80717	0.726494	0.401966

Cs2g20410	2.942917	3.837219	6.42411	5.913271	4.309616	4.313747	4.409301	8.875075	2.180886	7.984168
Cs2g20590	60.38518	84.85081	224.503	253.0707	307.6768	431.0402	127.4658	71.91667	258.6617	286.8214
Cs2g20600	0.610002	0.815016	1.33856	1.282998	0.637478	0.904868	0.802319	1.068718	0.444739	1.209199
Cs2g20620	3.620173	3.564124	3.457841	2.819572	2.465293	2.917884	1.629921	1.572852	1.557162	0.556263
Cs2g20635	3.47246	3.350638	4.963714	4.083522	4.994238	4.855018	1.384543	6.084463	3.531669	2.149802
Cs2g20710	2.965113	3.236503	3.129447	3.357136	2.223678	1.826266	1.116709	2.881283	1.927431	2.313049
Cs2g20860	66.36053	80.98682	72.8834	70.77014	43.41824	44.4842	18.16539	30.33463	19.38275	32.16469
Cs2g20890	9.897521	12.58429	9.760425	13.11341	8.157009	7.770525	4.973086	9.95525	2.018693	4.822923
Cs2g20940	13.67224	9.188449	4.717405	5.030601	5.340604	4.607794	5.356835	3.805645	3.424295	1.826839
Cs2g21000	257.0906	196.7531	344.1035	393.2056	219.4514	284.5175	588.0651	130.9321	417.4135	100.6129
Cs2g21085	5.093686	4.963192	4.53528	5.592837	4.581382	3.981784	2.120592	6.172961	2.397046	2.088834
Cs2g21100	78.99743	170.4128	62.42549	75.8379	17.0509	22.07977	20.84141	86.03835	21.62261	20.59786
Cs2g21150	75.33781	32.32938	33.41392	31.28981	7.384393	8.02552	30.06057	11.09229	49.46762	20.73102
Cs2g21160	6.166964	8.954603	4.043604	5.77098	2.549346	3.18845	1.257365	2.329417	2.55666	1.11378
Cs2g21170	0.916376	1.428596	1.439566	1.99676	3.392733	8.337107	3.709144	12.57589	10.884	18.60537
Cs2g21210	6.29156	1.993858	2.465406	2.067315	1.068016	3.167344	0.812143	0.363158	0.024212	0.041819
Cs2g21440	4.065775	6.212053	5.955233	3.784787	3.743251	6.043402	1.152533	8.698659	1.126846	1.714182
Cs2g21530	28.89836	34.05848	34.4835	29.20908	23.62863	25.56285	17.70286	47.71733	16.09647	27.27247
Cs2g21560	55.23395	52.47914	48.39019	41.82522	35.2542	31.75916	20.48737	16.70247	8.242731	10.96872
Cs2g21590	16.1423	17.07977	28.02399	25.56723	24.50648	27.7089	10.59996	32.89155	11.41406	18.17044
Cs2g21595	4.348463	5.612473	8.501213	7.777739	11.30905	8.536944	4.99304	12.4766	7.196198	8.714278
Cs2g21750	0.90374	0.523352	1.237977	0.91587	0.739734	1.782295	1.181829	2.31469	0.586639	0.204505
Cs2g21820	4.939114	4.759819	1.375839	1.386765	0.251585	0.635588	0.099046	1.494995	0.184938	0.080048
Cs2g21840	2.530532	1.776881	1.470613	1.761394	0.797327	2.335222	0.415769	1.3439	0.023654	0
Cs2g21900	7.682114	9.579092	6.128061	8.347121	6.580251	7.766331	2.243348	3.250411	3.140967	2.330276
Cs2g21940	28.82171	47.35544	4.444819	13.35484	5.989187	2.921231	15.50972	28.15358	0.930898	0.641557
Cs2g21960	5.275483	4.871333	6.904335	6.816958	10.86653	9.464547	11.4889	16.10278	9.504695	9.009151
Cs2g21965	4.12333	4.359934	5.090251	4.859063	4.148067	4.399527	1.972408	6.06866	1.787935	2.129406
Cs2g22010	4.874699	5.411373	3.893195	4.523436	14.63514	10.5263	8.922713	22.90921	2.788924	1.999151
Cs2g22040	116.9609	134.8603	75.91143	74.28797	29.64091	30.86541	6.533513	25.91029	1.206051	1.062557
Cs2g22050	40.38728	43.7073	53.39962	56.47647	26.77691	34.79046	7.685791	55.49546	2.572012	2.497283
Cs2g22100	245.0734	185.6379	389.2014	492.1705	79.64109	185.2063	36.54339	84.25888	6.064989	2.820349

Cs2g22140	2.1632	2.305299	1.288898	2.116304	0.162446	0.511425	0.308985	1.494374	0.035851	0.093631
Cs2g22185	0.902949	1.277882	0.831639	1.827839	2.140497	2.506086	1.600849	4.78239	0.148431	0.599644
Cs2g22190	1.26333	1.620697	1.3898	2.307557	2.118787	2.623616	2.250861	5.978707	0.380953	0.87617
Cs2g22200	16.08037	10.27935	5.306492	7.381593	0.296855	1.527251	0.128625	2.072224	0.35262	0.164755
Cs2g22210	0.506507	0.464199	0.923599	0.398836	0.534604	0.516229	0.415862	0.363261	0.039374	0.012852
Cs2g22220	0.506039	1.434441	0.549775	0.82098	0.217863	0.560437	0.662268	9.310403	0.246199	0.548184
Cs2g22280	0.655053	0.830519	1.016298	1.31509	0.153759	0.128823	0.288081	3.330837	0	0.401527
Cs2g22300	0.565857	0.530175	1.092262	1.139209	1.234614	1.218666	1.322374	2.578287	0.704528	1.419634
Cs2g22340	0.767215	0.736803	0.546805	0.431309	0.298707	0.285935	0.567786	0.229292	0.858083	0.1816
Cs2g22390	46.02102	57.85501	35.11142	40.80014	36.02221	35.37912	7.299578	17.3647	2.815826	2.244083
Cs2g22420	43.93898	25.56798	18.88036	23.55326	27.06966	34.78524	60.83228	26.91234	85.73832	95.7727
Cs2g22470	1.950077	1.14278	2.524303	3.53793	2.274058	1.946831	3.538222	3.215904	0.480327	3.917541
Cs2g22510	2.945846	2.238841	4.684825	5.521108	4.699052	4.662343	6.669273	6.521293	2.460288	6.147485
Cs2g22540	0.810288	1.589182	7.540304	7.976017	4.30778	6.384779	1.820354	9.025945	1.366185	2.354338
Cs2g22610	5.219611	4.851057	20.80105	22.60093	11.46872	40.9311	4.646193	3.937296	2.417921	0.256656
Cs2g22630	12.88646	11.22381	12.08402	21.99814	2.170143	6.391977	1.399632	3.586281	0.940251	0.552509
Cs2g22640	3.45272	3.471365	4.100668	3.867624	1.43417	1.760134	0.390581	1.50699	0.385428	0.111082
Cs2g22650	121.216	145.7856	62.14899	103.0451	17.16444	15.36055	8.187132	16.86632	5.140667	7.062169
Cs2g22670	5.166586	5.808156	6.866601	6.896157	5.448199	7.517538	3.110536	6.786425	7.11843	3.826035
Cs2g22680	2.291888	3.276273	3.634881	3.480696	1.942387	2.737638	1.693018	4.769377	0.672291	0.487321
Cs2g22690	0.081148	0.082635	0	0.006028	0	0	0.911982	0.282512	4.610138	0.878562
Cs2g22710	12.62188	14.88391	2.461538	1.844747	2.525436	1.567285	1.367245	1.4848	7.398982	3.422691
Cs2g22730	54.57747	66.2393	53.82246	58.54115	39.97095	50.10723	19.38978	42.13809	12.68736	19.5019
Cs2g22740	9.621449	6.207275	21.83385	5.538857	22.99086	21.82631	13.01779	11.6133	31.35311	36.84205
Cs2g22770	1.970052	1.107227	0.525527	0.15852	0.465122	0.480639	0.183727	3.172435	1.987112	1.243198
Cs2g22790	3.159262	2.43963	1.66174	1.389188	2.067553	2.305892	0.72611	9.541671	6.258819	3.48288
Cs2g22800	4.817366	3.330242	2.579162	2.362522	3.406065	3.643165	1.741577	15.7048	8.962514	4.210586
Cs2g22810	4.162508	2.988428	2.06336	1.718477	1.319411	2.407253	1.902142	12.36179	14.79522	9.586446
Cs2g22820	37.37743	31.7722	14.28339	13.80576	6.032607	11.98527	3.011966	14.77676	5.331782	4.232149
Cs2g22850	0.525546	0.4009	1.050076	0.756822	0.509523	0.688836	0.80744	1.686942	0.392453	0.437873
Cs2g23080	7.256004	6.871105	6.107761	6.490005	5.451757	6.330867	3.127338	7.069663	2.742641	3.21846
Cs2g23170	1.097841	0.967026	1.101512	1.161853	0.646574	1.084373	0.343738	0.994571	1.186391	1.985603

Cs2g23200	2.367964	2.22691	1.76549	2.411322	1.213068	2.491085	1.596603	1.431504	3.353891	2.765804
Cs2g23260	1.486245	1.893019	1.742889	1.951718	1.018107	3.612179	0.130774	1.779111	0	0.325803
Cs2g23280	77.04404	77.85531	190.8259	138.4566	506.2558	372.9485	55.32268	50.48887	6.824162	1.982702
Cs2g23350	10.66055	11.13302	11.38729	11.1455	28.2255	36.97313	26.8665	45.92246	8.496625	6.69174
Cs2g23360	10.02973	6.420517	19.83527	10.27276	51.20903	15.88916	24.51891	22.14785	2.850721	2.00135
Cs2g23550	6.782823	6.771845	5.198719	6.609653	4.992576	5.699217	2.937266	9.299568	3.484689	6.160653
Cs2g23590	54.99016	62.87387	61.64106	62.29823	45.73015	57.2823	19.81658	59.56338	44.65419	67.75663
Cs2g23630	1.340638	0.721829	1.18963	0.961777	1.188715	1.059473	1.218085	1.388207	0.662256	0.274493
Cs2g23660	134.5804	177.3214	125.8591	138.1481	35.22442	35.26952	53.60038	181.5906	60.70416	63.2083
Cs2g23750	30.06077	25.06117	21.97757	22.70148	53.69395	38.41949	60.47556	73.89587	420.7879	172.9212
Cs2g23760	1.083934	0.813221	1.208439	1.078505	1.199787	1.638078	2.353294	2.300384	1.783821	1.497518
Cs2g23970	3.989592	7.736929	1.008083	1.474148	0.575782	1.622736	4.316899	1.452564	0.012987	0.044427
Cs2g24020	0.649832	0.69377	0.79659	0.670076	0.539555	0.873147	0.313996	1.048641	1.271331	0.629774
Cs2g24030	13.64034	14.19529	10.16923	8.631438	5.342781	4.321311	2.604699	2.026825	0.105823	0.192914
Cs2g24110	2.346155	2.817642	2.237479	2.780571	1.40451	1.933372	0.47699	3.809879	0.668792	0.99132
Cs2g24220	6.374501	6.779582	11.0221	13.27023	18.78984	15.44886	35.72846	17.78002	33.36761	20.7661
Cs2g24300	0.335288	0.548909	1.411773	1.387074	1.087972	2.219333	2.128836	3.073065	4.41991	7.548504
Cs2g24350	8.98678	12.4986	8.397219	9.856789	8.540654	8.634178	26.68846	63.67371	12.17282	16.58048
Cs2g24360	26.7836	48.259	24.22419	29.26183	19.04449	27.32741	130.6042	282.566	42.68897	87.10682
Cs2g24370	3.794101	3.979396	3.579815	3.793216	3.577159	3.584568	1.426484	3.354171	1.911867	2.077231
Cs2g24410	5.30384	4.026613	3.585635	3.306307	2.051869	1.667763	1.749884	1.577278	2.634225	2.603321
Cs2g24530	3.792097	3.31725	3.055084	2.107539	0.590449	1.259894	0.185641	0.444779	0.008232	0.009735
Cs2g24640	0.730495	0.712189	1.666201	1.563609	0.978894	1.091733	0.750931	1.290526	0.31045	0.247427
Cs2g24730	19.00941	18.62095	24.19525	13.87026	28.95976	7.280662	19.36272	12.43009	7.342097	10.41762
Cs2g24740	1.821982	1.846137	1.867687	1.963438	2.274458	2.490343	1.448712	4.655892	6.417621	3.959428
Cs2g24784	3.1194	2.651939	4.898616	4.139568	4.320388	3.748751	1.739898	4.350408	1.58282	3.423373
Cs2g24960	10.24694	8.680223	7.218479	6.077702	6.473849	7.228051	3.131244	1.77508	0.602425	0.476455
Cs2g25040	8.800774	8.855374	5.896849	8.147927	3.489686	11.22466	3.956387	6.317187	1.169319	1.267686
Cs2g25130	1.219476	1.032557	3.800737	3.496721	5.904529	5.822722	2.449336	3.37251	22.03056	10.71796
Cs2g25150	61.3507	77.88131	42.23124	41.95574	20.55577	14.87294	15.44826	19.76382	48.74508	35.76523
Cs2g25285	2.065261	2.98261	2.656836	2.936867	1.753056	2.150105	0.724582	2.674658	3.629078	1.217797
Cs2g25290	1.547665	1.002023	2.901015	2.473481	4.089549	3.713788	6.024255	2.84633	3.133748	2.730439

Cs2g25300	3.493887	1.518367	3.190003	2.98199	2.924309	8.567303	0.923759	1.723053	3.482616	2.432282
Cs2g25360	1.791035	1.709565	0.710727	0.686591	0.944438	0.829654	0.614286	3.638339	0.037104	0.084564
Cs2g25380	0.350593	0.821625	0.376482	0.236927	0.552695	0.728809	0.508785	1.193327	0.065406	0.257418
Cs2g25440	2.340672	1.989123	3.190907	2.302583	5.714624	4.55293	15.80048	9.367168	10.936	9.56959
Cs2g25450	0.731708	0.602661	3.112526	3.016772	1.679369	1.441734	1.951662	7.482266	9.64371	28.93116
Cs2g25470	3.402214	3.328293	3.492831	3.587182	4.491728	4.193089	11.31575	7.4631	23.33534	29.08637
Cs2g25560	0.016258	0	0.632832	0.554628	0.404671	0.876931	0.02607	0.370582	0.480101	2.176776
Cs2g25580	14.05604	8.217523	14.10031	19.80824	31.55083	49.94419	45.22328	48.86987	41.24229	40.63655
Cs2g25640	19.18994	25.69889	15.25629	14.67923	10.87994	10.75008	7.762239	6.005132	3.193686	1.361817
Cs2g25650	31.69598	19.04795	42.60252	25.46952	47.67072	43.5384	22.55934	15.96138	15.47108	5.402076
Cs2g25790	36.95017	29.35204	30.7942	32.27817	34.56955	35.39711	48.12279	23.26185	76.28183	66.72914
Cs2g25850	5.303914	12.50485	5.093099	4.499109	3.409057	4.831627	1.617903	16.00935	6.193534	10.90106
Cs2g26075	2.781021	3.772321	2.846117	4.203911	1.328912	1.484459	0.473609	1.810468	0.168113	0.331946
Cs2g26110	4.250384	6.311111	5.171312	6.454461	2.726071	3.598044	1.123462	3.023383	0.760326	1.045041
Cs2g26210	7.246451	10.33752	7.465658	8.615878	5.435659	4.777484	0.905941	2.276957	0.396739	0.604009
Cs2g26220	1.592802	2.31901	1.447298	1.854808	0.891887	0.872619	0.334121	1.526066	0.234583	0.246946
Cs2g26240	2.900904	2.928697	3.398309	3.30577	2.871658	3.557292	1.315065	2.941564	0.850791	1.218076
Cs2g26250	1.166519	1.192912	2.598985	2.994431	2.423416	2.634366	1.287248	3.433599	1.429279	1.624417
Cs2g26590	10.68499	11.69922	46.49448	43.38227	20.58114	26.11332	2.487442	6.790943	2.162894	3.153579
Cs2g26610	5.305542	5.055058	17.0125	16.42462	12.96467	23.97205	1.36569	2.524365	1.173744	0.88181
Cs2g26640	3.769749	6.051282	2.257269	2.990193	1.320311	1.888251	0.942748	1.288859	0.0175	0.034621
Cs2g26650	3.640556	4.437404	9.634613	8.338075	1.253716	2.602281	3.719103	1.546659	0.113418	0.124739
Cs2g26680	0.427859	0.724538	1.064424	1.184002	0.803891	0.839635	0.431741	1.724838	0.046875	0.139911
Cs2g26760	24.40846	30.13999	22.08993	26.7901	12.68765	14.13552	5.536046	16.80706	7.735764	12.87749
Cs2g26765	2.154769	2.163637	2.390087	2.357798	1.279699	1.84776	0.853521	2.780074	1.424711	2.725351
Cs2g26780	2.784684	2.27716	4.236708	4.039492	8.284928	5.701167	15.47866	7.768703	3.834615	6.315058
Cs2g26790	4.588853	5.345812	3.977829	4.987964	3.472088	4.289865	3.554512	4.08944	0.551708	1.645667
Cs2g26940	1.123547	1.353428	1.849171	1.715562	1.286838	2.104511	0.509569	1.648353	0.428134	0.492746
Cs2g26950	3.710509	3.626528	5.050729	6.630728	2.655113	2.069685	1.577283	2.966494	0.146866	0.214545
Cs2g27040	63.06334	62.38245	83.8186	80.57467	44.44972	70.92608	19.5975	68.76351	8.632475	14.49696
Cs2g27070	1.150798	1.567025	1.330449	1.612421	2.830038	0.978754	0.930172	0.994345	2.209189	1.589956
Cs2g27090	93.11471	46.65011	19.91807	19.66197	15.6786	15.37897	6.199934	4.238691	0.273804	0.289808

Cs2g27100	163.5616	71.55715	39.03871	35.34421	39.70161	45.33603	24.22585	15.0955	1.429513	1.213056
Cs2g27110	2.434374	0.554133	0.104056	0.235255	0.346186	0.399416	0.09686	0.066177	0.046118	0
Cs2g27160	5.010164	5.727663	4.857144	4.595641	2.102462	3.71792	1.164103	2.724022	1.498871	1.64317
Cs2g27170	42.90593	53.22135	40.77865	45.92105	29.73996	26.89983	14.7554	22.28683	8.148612	11.97709
Cs2g27180	7.108894	9.016252	10.30632	11.10812	13.64834	13.81971	7.358958	31.05107	7.997663	8.325451
Cs2g27230	23.75412	19.86137	18.33186	22.36467	17.60475	42.50467	21.63645	22.31056	44.06356	28.04424
Cs2g27250	7.681903	10.46732	10.423	10.88449	10.08799	12.62298	4.840758	10.81965	8.091117	8.804314
Cs2g27460	6.519462	4.270059	3.732034	4.960203	0.697799	3.603598	1.323214	2.253382	9.652336	4.211616
Cs2g27470	12.51477	9.6438	19.68585	16.42834	26.97447	19.24689	26.52007	24.32838	34.45625	25.00507
Cs2g27610	274.792	157.11	56.13172	67.62289	37.93497	31.9318	29.82786	12.31318	93.74381	96.28101
Cs2g27640	25.10411	21.47188	19.81362	24.95537	7.98654	6.634752	4.39049	14.58375	0.907812	2.386156
Cs2g27650	18.30591	28.1923	25.14828	28.34363	45.66341	42.05731	63.48201	113.051	10.75214	24.36204
Cs2g27710	1.505321	1.027545	1.206177	1.278822	0.582551	0.946565	0.34009	0.826257	0.23463	0.276563
Cs2g27720	9.351429	8.387186	22.80098	18.04051	31.9141	29.75124	44.29888	35.16087	32.71694	32.60524
Cs2g27760	4.635295	3.98511	3.517818	3.55315	4.405708	6.50784	2.425191	5.193704	0.149782	0.141129
Cs2g28000	14.48383	20.96934	20.05902	28.54817	10.79301	19.29162	18.5882	27.28023	14.50539	58.19017
Cs2g28060	95.59477	120.2287	37.79262	66.15482	6.411306	5.222829	11.39214	12.92842	4.54488	5.429569
Cs2g28070	0.386572	0.382428	0.534447	0.572332	0.910027	0.707997	1.843255	2.362375	0.387632	0.534618
Cs2g28090	5.293242	5.494454	2.426685	3.057796	1.508184	1.318356	0.352364	1.003882	0.225779	0.871307
Cs2g28110	7.490938	6.760357	13.1667	12.11979	15.32756	11.08911	8.038957	6.937126	3.007513	1.565109
Cs2g28120	0.463245	0.982439	0.576445	0.610581	1.308388	0.746378	2.382434	0.986639	1.649996	2.377303
Cs2g28140	95.59301	98.41213	20.44798	37.73461	1.78257	5.466502	0.458229	1.358597	0.180383	0.240313
Cs2g28150	2.821846	4.321439	2.495566	2.94564	0.858827	1.952637	1.196405	5.672965	0.209701	1.245195
Cs2g28206	3.315848	3.722146	3.717871	3.977739	3.157264	3.443857	1.014859	4.523077	1.850782	2.541096
Cs2g28260	15.31301	16.92885	9.47431	11.11352	5.424269	5.970557	2.35157	6.606299	1.252464	2.107266
Cs2g28370	3.925321	1.932568	2.479785	1.448308	14.97421	5.89898	159.9331	34.70616	30.7398	41.32512
Cs2g28420	36.0177	71.95447	10.49195	18.38399	2.315492	6.151489	2.077501	8.154054	0.320457	0.543847
Cs2g28500	10.08058	12.34241	9.755251	8.746879	6.414582	5.422258	5.498167	7.524493	1.561312	6.266883
Cs2g28530	0.664149	0.99877	2.153953	2.580358	0.994843	0.806035	0.71158	1.795138	0.37898	0.814755
Cs2g28570	0.887828	1.555127	1.798093	1.510144	0.796077	0.591344	0.403639	1.111412	0.073975	0.070043
Cs2g28575	0.442586	0.604578	1.735797	1.117663	5.282371	3.117231	3.51875	4.918628	1.180933	1.531865
Cs2g28590	5.203398	3.682297	17.25001	10.65477	27.96782	31.69907	10.97655	13.99056	2.169362	6.019362

Cs2g28630	1.011703	1.044415	1.580602	2.107382	2.11497	2.505446	0.775989	1.614708	0.028536	0.151247
Cs2g28730	3.069037	3.544853	2.509852	3.208156	2.076148	2.581885	4.17529	12.17861	7.624458	15.84926
Cs2g28790	1.939176	2.029577	3.016676	3.312417	1.470428	1.384502	2.158068	2.532688	0.064382	1.200903
Cs2g28800	0.905625	0.786847	3.679669	5.932927	7.56257	12.53101	4.725245	8.750824	14.41497	11.97767
Cs2g28820	0	0.009127	0.046362	0	0.355442	0.857272	0.134253	0.840725	0.156412	0.632404
Cs2g28830	0.017514	0.097336	0.127294	0.123062	1.242631	2.963487	2.165841	4.010451	19.23272	19.9505
Cs2g29000	29.93069	33.4096	22.99698	23.61883	14.51001	15.70133	8.340937	13.24428	13.04008	15.99831
Cs2g29010	0.825666	0.94881	1.301031	0.897252	2.062502	3.956448	0.979623	2.638659	2.921968	0.990877
Cs2g29070	138.9855	143.1823	133.4733	115.8969	68.12503	73.9075	63.93641	84.39432	40.27284	49.51562
Cs2g29090	34.62061	31.816	1.709122	5.497138	0.329123	1.05227	0.106215	0.102893	0.42282	0.459709
Cs2g29100	58.72944	16.6802	96.74535	46.99823	51.922	114.4216	98.6604	13.48654	16.35233	80.44458
Cs2g29110	6.415199	5.94908	5.073677	4.5478	2.16576	3.532973	0.959394	2.52105	0.263158	0.423492
Cs2g29120	56.07957	86.67791	41.63264	51.63373	8.227102	10.09442	42.38939	90.64426	58.75123	178.0124
Cs2g29205	0.130273	0.095709	0.627742	0.646522	1.048248	1.398686	0.552134	1.662754	2.183233	3.571104
Cs2g29210	3.168828	3.155591	0.800242	1.727222	0.840394	1.040043	1.058138	1.310456	0.192249	0.110055
Cs2g29300	8.360466	7.589009	9.378626	9.097372	11.64846	14.81293	14.35715	12.81505	33.02694	12.09173
Cs2g29310	5.306838	4.740207	4.495216	4.332004	3.435614	4.455809	2.9219	3.681076	12.03139	3.729126
Cs2g29360	10.71372	4.374821	4.111421	3.179063	7.986694	22.98984	11.15198	23.29547	111.9244	69.69947
Cs2g29380	13.09197	14.00736	23.24171	20.36837	26.25633	35.20004	11.09641	29.2548	25.39378	18.82836
Cs2g29430	58.94679	44.20559	20.46115	20.61248	4.988532	8.339481	2.279242	2.606139	1.257044	2.418695
Cs2g29540	20.2775	17.93082	10.18354	9.774896	12.67664	9.45366	4.619881	3.402396	11.31511	4.919821
Cs2g29560	4.671727	5.566938	5.138894	6.6035	4.798558	6.375593	2.842617	6.465576	3.620684	4.810191
Cs2g29610	12.08508	16.89208	10.94918	11.90044	4.919748	7.238479	4.573858	7.743219	4.446089	7.019771
Cs2g29630	28.33405	26.15504	5.759394	11.10832	3.133585	8.88488	6.937944	6.69876	3.160804	1.376806
Cs2g29660	0.479111	0.907525	0.350952	0.421748	0.227924	0.259018	0.44899	0.658022	1.139322	1.676573
Cs2g29700	1.208973	1.300678	2.704305	2.77789	1.249254	0.994062	0.942163	1.700273	0.251637	0.509426
Cs2g29720	0.451113	0.221464	0.517415	0.367079	0.122262	0.895743	0.06546	0.29489	0.056203	0.28219
Cs2g29773	5.52662	7.762464	0.160055	1.088832	0	0.079623	0	0.033618	0	0
Cs2g29780	195.3816	256.7561	91.1477	204.2089	8.493439	16.6503	27.08481	17.60587	33.66187	15.03028
Cs2g29790	134.2093	116.1157	32.17684	70.07349	2.213177	8.578088	2.548991	3.829451	13.7531	5.834634
Cs2g29810	3.447438	4.186458	1.114849	2.918884	1.141888	0.010211	4.900841	0.688692	8.829753	7.717766
Cs2g29820	2.998149	3.657273	0.009419	1.679605	0.536845	0.008392	2.772811	0.225799	4.496459	7.562859

Cs2g29840	1.102686	1.252461	1.361818	2.789444	0.289691	0.500369	0.622994	2.431355	2.191838	3.269291
Cs2g29850	4.154021	3.570259	10.64167	11.22895	2.498123	4.661775	1.5716	2.332631	0.342171	0.22976
Cs2g29860	2.183119	2.224109	3.996991	4.432183	1.237767	1.980962	0.976984	2.949495	0.317585	0.980801
Cs2g29900	0.262399	0.31044	0.733264	1.072913	0.132267	0.821544	0.180077	1.463871	0.04393	0.103985
Cs2g29910	0.417586	0.537468	2.271423	3.541833	0.265639	1.186527	0.383827	3.835561	0.154101	0.33508
Cs2g29965	83.69254	89.52353	171.357	161.0371	167.9669	175.5358	49.10884	156.979	139.9258	188.2519
Cs2g29970	5.045318	2.721215	1.398026	1.604493	2.654696	3.384211	5.698369	2.125676	11.16159	8.368207
Cs2g30160	2.293758	3.003735	2.087504	2.005078	1.633609	1.870538	0.884916	2.56597	0.220751	0.556829
Cs2g30260	13.44711	24.19457	8.613359	11.92881	27.97166	18.64473	63.50854	41.14449	31.85316	20.9697
Cs2g30280	5.947247	11.7045	7.282452	8.54721	9.641203	7.160886	7.951396	8.963639	7.506695	7.419879
Cs2g30320	46.49703	61.54538	50.091	62.62598	125.2642	118.2593	97.83747	56.21772	197.5648	84.74191
Cs2g30400	87.23887	166.8006	23.46727	31.38854	5.629292	8.141921	5.334312	21.03845	1.269833	2.419061
Cs2g30460	12.09882	17.45657	118.6546	72.29218	348.8938	209.7342	389.9562	433.9665	318.4598	324.8878
Cs2g30470	4.84766	5.93741	23.63977	21.17683	17.60977	19.81553	37.14416	47.59738	35.3992	39.52241
Cs2g30480	7.049582	3.830182	22.38869	11.79984	19.10231	17.32263	55.11544	11.56011	72.50859	14.60151
Cs2g30590	66.18037	63.76709	48.25606	51.16204	19.57475	32.08544	9.390053	29.0876	22.02781	35.35696
Cs2g30600	10.27549	8.738365	4.461942	4.941078	1.306375	2.801322	0.168468	1.438192	0.363692	1.227456
Cs2g30620	1.780219	0.942727	1.016726	1.331648	1.479575	1.661674	0.131501	0.980632	1.493847	1.628498
Cs2g30660	6.349482	5.560026	4.627513	4.662436	4.430458	4.971508	10.552	16.3562	1.217007	2.0148
Cs2g30810	14.00339	19.20266	9.662552	14.14718	8.296692	7.969587	3.890933	17.03505	10.95769	10.37645
Cs2g30840	10.05617	15.3643	23.16518	22.93714	4.261842	11.56408	10.21206	36.32216	7.691274	20.99555
Cs2g30865	9.062146	6.7603	10.43059	11.04323	5.97057	15.40761	1.359826	6.067621	8.582347	9.004563
Cs2g30870	14.7435	21.69177	7.507102	13.33312	5.958267	5.308082	3.969166	5.180279	2.024231	2.200476
Cs2g30920	6.377038	5.850573	7.561527	6.654873	8.445223	11.6659	3.692322	10.89404	9.809823	2.828836
Cs2g31040	1.208706	1.096165	1.482991	0.694264	4.070037	4.470999	0.707363	2.297899	0.90482	1.209602
Cs2g31050	0.042611	0.267034	0.033895	0.407481	0.485224	0.33349	0.786248	1.382846	10.24898	739.3693
Cs2g31080	647.1957	584.5609	636.8664	598.4244	423.6959	447.3449	146.0101	491.4659	419.5195	372.8374
Cs2g31090	0.992187	1.318115	1.325331	1.912105	1.121019	1.230483	0.188913	1.728985	2.405259	2.20595
Cs2g31130	5.807018	3.297867	4.52197	4.615585	0.381346	1.12737	0.655339	2.893228	6.685835	8.931746
Cs2g31150	47.20025	29.84366	27.53772	43.84428	3.62272	14.8879	6.360204	9.183096	31.58212	16.4155
Cs2g31200	9.045549	6.40395	7.992624	7.989446	7.720088	10.27877	7.680314	16.75744	18.15551	21.89584
Cs2g31220	12.25634	25.16889	4.546396	8.949405	5.974178	6.394715	4.471214	11.70238	3.561532	6.295097

Cs2g31250	35.63401	85.51693	20.10971	18.66076	3.914907	6.875673	7.065174	26.22604	5.25678	19.61913
Cs2g31270	19.44696	22.29477	17.71586	19.05	11.27914	12.06928	4.682235	14.4214	13.71807	26.41347
Cs2g31340	0.896487	0.668598	1.504156	1.181425	1.02081	1.328268	1.727439	0.709883	1.38858	1.159803
Cs2g31350	44.13359	34.40078	7.509239	11.14365	0.70547	2.733453	1.359056	2.17338	5.474347	3.572415
Cs2g31360	226.3811	381.8389	78.2434	88.22893	16.72559	9.328435	3.04027	3.816179	0.469248	0.504673
Cs2g31390	187.266	194.8001	252.7328	221.4533	147.8832	104.5854	32.68096	50.73894	60.05722	89.86588
Cs2g31400	4.564926	5.280419	0.778729	1.682559	0.454881	0.156436	2.424561	0.20892	3.618496	2.758525
Cs2g31410	1.468391	2.346856	0.057552	0.197504	1.544115	0.066447	9.038896	0.904341	19.11698	11.77162
Cs2g31420	195.8766	154.0585	45.10323	89.5952	3.771831	10.89714	3.815768	5.508984	14.65758	4.744613
Cs2g31430	63.98926	49.15128	15.74979	28.31284	1.297649	3.183041	1.187053	1.696584	5.653208	1.755997
Cs2g31435	8.692477	7.102574	1.963163	2.83179	0.510996	1.098534	0.244421	0.457757	0.572163	1.100301
Cs2g31450	10.72083	7.192651	2.378888	3.121497	4.771442	10.27674	6.16967	15.1447	20.06074	24.94508
Cs3g01070	3.395045	3.975566	4.895815	5.193157	5.302959	5.527296	2.594213	7.561843	5.577055	4.537364
Cs3g01110	87.46529	81.26817	52.81278	58.94702	32.31848	40.81104	31.14658	36.42055	52.09327	38.50144
Cs3g01120	35.98992	111.5917	15.85051	44.51315	6.990215	4.996414	13.36697	10.3404	3.12317	2.045851
Cs3g01130	31.49585	35.44826	17.65471	20.39384	11.14834	9.969694	16.96051	6.084022	5.992045	2.599091
Cs3g01140	274.7873	343.6688	97.11257	170.2479	25.47033	13.68917	5.460492	3.376479	1.850668	1.16269
Cs3g01160	1.083835	1.564966	1.579067	1.481466	1.460711	1.851495	0.380886	2.10262	0.563951	1.187627
Cs3g01240	2.673952	2.551174	11.86675	9.382001	24.97528	15.53067	10.50591	25.98402	30.63359	35.339
Cs3g01350	1.383115	1.045177	0.887719	1.369326	1.222534	1.427953	2.336025	0.9065	2.637071	1.112479
Cs3g01470	4.672486	6.486956	4.204481	3.309109	2.776735	3.053807	2.090981	3.863048	3.466773	3.231282
Cs3g01480	108.9806	98.15886	104.4055	82.72601	40.79319	51.92283	13.48885	52.94918	0.882749	1.043482
Cs3g01485	9.623381	8.320875	7.617093	9.702935	4.365407	7.084944	4.176223	19.74025	0	0.373057
Cs3g01490	67.28253	67.05116	44.09527	45.77022	5.731277	10.71846	4.177219	14.22094	0.094735	0.162263
Cs3g01500	6.706724	6.958359	5.273693	4.718116	0.859414	1.442298	0.390619	2.822682	0	0
Cs3g01530	7.969646	4.878816	4.399084	4.051491	6.034195	7.108729	19.30903	6.961034	20.31298	8.354207
Cs3g01540	3.413387	3.658902	4.51001	4.838416	1.437279	1.711837	2.485802	3.695279	2.651294	3.833822
Cs3g01570	54.60108	43.34394	39.95277	31.99873	24.53083	29.24501	19.8037	15.04051	22.27511	17.39498
Cs3g01585	1.892534	15.38352	7.348247	8.429555	59.85069	4.883217	42.76574	220.266	3507.57	5268.41
Cs3g01630	20.03596	20.31692	24.62482	29.09926	24.1093	34.26348	48.70488	45.74384	109.5731	85.61111
Cs3g01720	0.79173	0.993816	1.115445	0.550881	0.869298	0.798662	0.938277	0.57052	0.117886	0
Cs3g01753	5.583775	5.727092	4.127166	3.968527	1.754916	2.67408	0.924976	2.697132	1.230065	2.070016

Cs3g01835	1.246962	1.409253	1.625584	1.578392	1.461341	1.252833	0.77438	2.456262	0.865208	0.617085
Cs3g01870	16.97265	18.29298	18.43303	19.66644	7.660012	11.80179	4.129333	6.69487	0.67577	1.228975
Cs3g01960	10.75594	9.164959	7.624243	6.912369	5.27249	4.825444	5.407621	4.723654	2.076837	2.76351
Cs3g01970	2.643699	2.814213	3.322742	2.929281	3.831674	3.344197	1.117521	3.800366	2.971206	2.323111
Cs3g02129	2.294583	2.728687	2.162816	3.563394	1.148787	1.497213	1.018227	2.767351	1.425051	2.53522
Cs3g02180	0.780865	0.643929	1.219498	1.136745	1.915343	1.993627	1.779407	2.437904	1.121026	1.47854
Cs3g02185	0.981217	1.462759	1.243047	2.101701	1.481343	1.901784	0.762508	3.185347	0.933506	1.285787
Cs3g02330	14.30719	17.65162	6.983207	9.008302	4.458152	3.197165	1.754401	4.698077	0.277139	0.506296
Cs3g02350	0.774934	0.355931	1.207389	0.944586	1.448444	1.885653	1.342263	0.573037	0.490123	0.032
Cs3g02440	7.6494	17.83323	1.142441	2.876362	0.900731	1.067739	0.285994	0.980454	0.204857	0.245911
Cs3g02510	20.23554	16.96351	41.7733	30.59046	83.80197	61.45037	109.3944	50.81454	30.63624	25.04068
Cs3g02580	12.70199	14.96708	2.018755	2.295321	0.949257	0.785825	0.058134	1.208153	1.141304	8.475695
Cs3g02660	8.844521	6.15256	14.36939	13.42828	24.55113	24.53396	26.47951	17.40038	24.8636	36.0784
Cs3g02670	1.198671	0.659982	0.97628	0.963497	1.132764	1.686278	0.523618	1.539344	3.915209	1.317109
Cs3g02720	20.4677	20.84051	24.12495	24.46288	12.09431	9.918044	6.300605	15.90011	1.66104	2.606975
Cs3g02870	6.931368	8.070598	4.878829	6.565663	0.777205	1.779243	0.750146	5.510312	1.046193	2.42913
Cs3g02920	0.474356	0.690986	0.982331	1.014455	0.490463	0.67028	0.2593	1.012657	0.230936	0.314093
Cs3g03045	54.48435	63.10673	102.1398	87.34813	44.06935	88.51829	8.67945	38.38195	0.151133	0.243122
Cs3g03110	24.13197	25.42467	21.37937	21.0868	13.91759	21.73363	20.7221	48.3155	35.30916	75.90197
Cs3g03130	1.285518	0.446089	0.824843	0.920891	0.551183	2.891878	2.826812	0.921555	2.41885	2.087265
Cs3g03135	1582.842	1520.513	633.2497	891.9234	124.0947	145.8878	10.83223	42.68592	1.318581	1.871639
Cs3g03145	24.60291	11.60566	83.59082	46.86327	123.762	91.09764	46.97736	132.3851	5.625799	4.737053
Cs3g03170	5.82339	3.684605	10.46084	7.005323	17.09948	10.18837	9.631564	7.819307	2.574124	2.44811
Cs3g03220	0.252189	0.289414	0.363004	0.261834	0.448574	0.354774	0.379945	1.001488	0.298496	0.727348
Cs3g03335	1.258176	3.102037	2.141838	1.561274	0.119537	0.183394	0.062872	0.044443	1.718358	0.418584
Cs3g03350	177.8867	327.7889	66.03215	210.0514	17.6765	21.39486	1.241219	7.476931	0.290601	0.637528
Cs3g03360	13.83954	18.96159	6.970636	9.903243	6.534114	2.86439	0.234236	1.984763	0.193757	0
Cs3g03390	11.28666	16.49697	5.798845	7.799957	5.163372	2.142721	0.056888	2.199322	0.193319	0
Cs3g03420	17.8473	20.22823	26.72754	32.54808	8.871412	14.92025	7.178485	21.14808	11.80564	44.36333
Cs3g03430	6.215779	4.364277	8.154104	6.98743	13.25773	12.53474	17.69505	7.083632	11.66952	9.310737
Cs3g03590	2.646653	2.669523	11.33977	8.256335	21.38272	21.53254	19.81634	32.50285	7.774759	21.78631
Cs3g03620	1.137431	1.127794	5.289394	3.427359	6.455405	5.953483	5.791756	12.86475	9.012101	25.22067

Cs3g03630	1.684861	1.608459	10.29849	6.861442	8.695251	10.37299	3.497874	11.6811	1.547568	2.303448
Cs3g03640	2.641062	2.29335	14.1431	9.520038	11.74581	14.3006	5.548118	15.27152	2.1807	3.168398
Cs3g03650	0.18582	0.195728	1.752679	1.653662	1.238172	2.618266	0.497904	1.467656	0.255671	0.296167
Cs3g03670	383.6441	720.2917	165.4695	384.4909	67.89075	47.64551	47.51579	129.648	9.892682	12.05581
Cs3g03680	56.55297	149.325	21.70832	53.35654	23.81587	11.70763	17.91379	24.47291	1.516509	1.375009
Cs3g03790	7.805835	11.73172	8.85301	11.34132	2.45679	2.603751	1.194007	1.612068	0.058955	0.064312
Cs3g03910	1.974526	1.950844	2.33758	2.287638	1.954761	1.923998	0.814311	2.511779	0.647888	0.978856
Cs3g03970	0.56698	0.539655	3.966959	5.727538	1.038954	14.30116	8.166095	2.814389	17.90535	51.61648
Cs3g04140	3.419515	4.162356	11.55235	18.09464	0.420491	1.744535	1.178978	2.814977	0.844637	3.26594
Cs3g04170	114.0065	86.56631	127.653	125.5824	68.49507	89.17293	28.07027	43.39472	54.56299	57.45853
Cs3g04190	16.52205	14.01387	20.35435	15.63125	9.330895	13.2348	4.240565	6.028535	7.290534	4.428883
Cs3g04270	0.884346	0.684449	1.692167	1.27211	2.008417	1.604643	0.921622	1.813915	0.900629	0.977671
Cs3g04340	0.51778	0.707212	0.856455	0.90461	0.546058	1.167619	0.083805	0.991186	0.622917	0.88341
Cs3g04360	224.9912	168.116	261.1433	259.6669	134.5497	183.8385	57.16747	84.00256	104.9705	109.8384
Cs3g04390	2.358982	2.945018	4.043172	3.921116	0.423831	1.661512	0.152256	0.210861	0	0
Cs3g04490	4.22774	5.005873	6.760743	6.852387	3.958629	4.698461	1.200841	3.147102	0.948247	1.682401
Cs3g04530	0.314208	0.127621	0.026034	0.049803	0.226488	0.211159	1.417161	0.627232	0.195886	0.236303
Cs3g04540	4.142948	4.992902	0.014143	0.024114	0	0.010127	0.119312	0.446988	6.164816	20.13996
Cs3g04550	23.25925	21.19735	30.30103	28.78817	45.84485	42.31023	47.38981	46.68983	42.74572	46.3471
Cs3g04570	1.748459	2.010784	2.282919	1.960545	2.27166	1.306136	0.919886	1.391171	0.94978	1.244894
Cs3g04755	0.315823	0.312526	0.683142	0.795456	1.452007	1.48218	0.991393	1.034774	1.630473	1.120801
Cs3g04800	1.74317	1.372481	6.617872	6.000968	6.325914	23.91318	3.544722	3.943097	7.065596	4.069276
Cs3g04900	3.814361	3.029956	5.258735	6.257211	1.894719	1.862807	1.406698	2.193495	0.26483	0.353882
Cs3g04930	14.45031	10.88495	12.08352	9.945795	5.622362	7.073216	2.923845	3.873188	4.252699	1.986591
Cs3g04960	3.402662	2.649074	10.42697	6.981113	19.2885	17.751	46.75196	12.66076	13.64893	12.66681
Cs3g05040	0.149115	0.256026	0.204739	0.210597	0.599265	0.307848	0.677968	0.92847	3.46327	0.443935
Cs3g05115	1.205687	1.40951	1.996988	1.423297	9.82405	7.906133	2.926578	19.56008	1.572428	2.004556
Cs3g05120	0.812163	0.901908	1.73102	1.341253	1.754924	2.219251	0.53105	1.709279	0.715214	0.613388
Cs3g05180	12.05803	10.97069	13.67324	10.79937	16.68022	17.1746	6.823129	10.47145	9.651972	3.932974
Cs3g05240	6.950559	12.22902	5.48908	11.61398	3.139356	8.582338	2.034788	10.24583	6.372599	11.30776
Cs3g05300	45.30362	83.14781	8.045473	13.09687	2.014139	1.769669	1.572761	3.860129	2.115722	5.786354
Cs3g05310	0.156186	0.144575	0.162827	0.060827	0.897506	0.185322	2.981453	0.922976	6.738266	8.012181

Cs3g05400	3.701349	4.002325	3.515431	3.56255	4.431475	5.452739	4.421746	4.084069	10.32678	3.84294
Cs3g05450	27.13202	21.35134	26.14412	24.61998	32.88643	35.41323	45.85972	20.27175	66.40424	46.49331
Cs3g05465	5.738211	5.680602	8.630039	8.864931	6.121818	7.467541	3.370978	9.63945	2.06496	3.283236
Cs3g05500	1.897146	1.486751	5.450456	3.324405	8.812334	10.59676	5.947437	9.481634	6.3774	5.83756
Cs3g05620	0.273353	0.797413	0.059787	0.085339	0	0.13543	0	0	0	0
Cs3g05660	7.403473	14.37207	2.125803	4.772283	3.597903	2.302124	3.743738	5.388938	1.256052	0.628074
Cs3g05690	0.744962	0.690561	0.604193	0.326467	0.548366	0.174558	0.50909	0.303576	3.045445	0.799954
Cs3g05760	2.044116	1.953154	1.484699	1.022498	1.024275	0.916554	1.182006	0.592728	12.13171	3.479754
Cs3g05770	5.273018	6.212804	3.489636	3.993911	2.763685	2.796202	1.548076	2.184225	11.25854	4.662358
Cs3g05800	334.4699	380.1444	274.8498	318.4977	50.27733	65.33031	38.91352	24.6131	2.077445	1.804642
Cs3g05820	0.600532	0.686814	1.064205	1.215543	0.617292	0.812401	0.346336	1.753492	1.088986	0.82019
Cs3g05850	5.110068	5.476776	3.351659	3.286376	1.698531	2.154143	1.218367	2.746016	0.024418	0.06281
Cs3g05870	2.141079	1.646137	1.836795	2.510597	0.181809	0.567898	0.178655	0.88442	0.090175	0.425529
Cs3g06080	9.21492	8.721884	12.72957	13.28463	6.25687	6.393246	4.400456	4.956012	6.684951	7.768175
Cs3g06180	13.15643	87.22524	0.527001	3.028045	9.497711	7.527564	6.272548	12.03936	2.400528	2.411111
Cs3g06220	5.965818	4.087717	3.379959	4.067978	2.385997	6.920453	2.550245	2.143115	6.563624	4.160703
Cs3g06370	18.35118	16.72166	40.69651	33.85572	10.11829	21.85121	1.189276	0.824967	0.111814	0.110906
Cs3g06450	6.248099	4.590051	7.048983	6.685174	5.904321	9.978636	1.723513	3.155074	1.830636	1.454621
Cs3g06480	6.029952	4.416609	5.351615	4.321748	1.432341	2.588896	0.901371	2.207603	0.378489	0.318143
Cs3g06580	0.397837	0.476894	1.716544	1.728474	2.29062	3.479546	5.974651	6.969289	7.804325	8.725846
Cs3g06590	3.131797	2.200985	3.513279	2.46326	1.66034	2.286089	0.389684	1.005553	0.223068	0.212586
Cs3g06595	0.351522	0.621838	0.783807	0.730744	1.024595	0.581761	0.624379	1.207363	0.548449	1.263838
Cs3g06630	5.532363	6.840978	3.003394	3.471718	1.705586	2.682511	0.835375	2.565995	0.076339	0
Cs3g06640	5.28704	5.886342	2.819657	2.967529	1.271142	1.808817	0.800473	1.885928	0.109366	0.030963
Cs3g06740	8.849501	10.91324	8.270908	8.687438	2.450449	4.321192	1.600626	4.035822	0.352368	0.361894
Cs3g06780	0.674555	0.78563	1.052845	0.965141	0.593789	1.104319	0.831652	1.963597	6.663229	5.411817
Cs3g06820	29.31253	17.34563	4.084574	5.797226	4.518758	6.034423	10.18588	5.496735	7.999475	0.39111
Cs3g06830	303.1182	211.4431	67.99574	109.866	174.5356	161.1528	434.6917	325.767	116.5375	11.15622
Cs3g06840	107.0207	135.2734	24.50285	42.26101	22.53574	26.8041	25.81407	36.86515	1.028064	0.324858
Cs3g06950	4.508796	5.94959	11.99672	9.750472	6.628807	12.46319	8.736535	14.25831	10.99801	19.93069
Cs3g07100	3.996399	1.855645	2.770043	2.268846	1.420778	3.855166	1.97976	3.195072	0.094321	1.341896
Cs3g07110	0.274578	0.279334	0.205208	0.246454	0.662243	0.503563	1.256448	1.940731	2.593066	4.258134

Cs3g07240	17.06043	23.44291	14.73828	17.30731	8.228282	7.973374	5.338311	14.32801	0.907024	3.415683
Cs3g07280	86.04483	54.76267	63.66669	64.24209	35.20973	73.05579	37.11509	48.73602	50.59299	34.51346
Cs3g07330	11.82172	8.077684	15.52666	16.78391	5.954803	13.43801	16.28773	22.67573	14.14172	6.794012
Cs3g07440	8.329716	10.69702	14.23906	26.66016	1.852632	4.76118	65.76265	48.19178	160.9264	132.8014
Cs3g07540	2.552614	2.374217	3.68426	2.447423	3.910892	3.453547	3.152023	2.709087	8.584721	3.424913
Cs3g07670	7.183203	5.554308	15.33223	13.28629	6.15188	6.664564	8.262816	15.31896	6.272937	14.13626
Cs3g07750	12.10991	23.28484	4.055549	8.757023	6.167371	6.423629	6.072165	9.608358	4.544946	5.035267
Cs3g07770	19.38139	16.15978	27.92058	33.11333	11.88167	25.66594	19.03272	13.51595	26.13615	22.72298
Cs3g07780	2.821082	1.904786	6.365172	4.656504	4.623882	4.464131	2.841833	4.807334	0.060214	0
Cs3g07820	166.1996	199.8095	137.747	156.0324	65.06971	62.82749	62.44731	105.7524	22.78344	35.79125
Cs3g07850	23.84706	19.83669	21.09674	17.82529	9.951169	15.33671	1.953218	5.984866	1.559287	1.323798
Cs3g07970	0.424475	0.383654	0.701026	0.465596	0.544505	0.596493	0.321612	0.389284	2.332388	0.69174
Cs3g08000	0.460407	0.482273	0.783172	0.799033	0.470477	0.886133	0.181599	0.456742	0.387068	0.28803
Cs3g08060	12.73275	17.73442	23.62078	27.84643	17.29515	33.20642	5.792561	13.42656	21.96001	10.21674
Cs3g08170	18.56283	17.96558	12.04975	10.38637	6.335873	7.287945	4.74985	5.452409	2.755576	3.660098
Cs3g08320	15.78269	11.87866	38.87665	29.35354	48.53217	41.72645	23.11892	39.02798	27.84599	24.79101
Cs3g08330	7.822674	5.515713	10.8645	10.48742	12.96303	22.72594	7.378828	22.69352	15.69803	19.45265
Cs3g08400	11.46639	18.76301	4.126329	7.743414	2.037231	1.829443	2.885766	6.807227	0.696536	0.951682
Cs3g08430	8.546149	10.62321	5.054125	5.581271	2.65107	2.321175	3.598282	2.963252	0.251656	0.118506
Cs3g08480	65.94601	109.9491	29.28298	65.37806	7.61687	5.440942	7.921155	12.90922	5.196932	5.569096
Cs3g08490	9.553546	10.85948	4.61888	7.212203	2.298486	1.296556	1.157486	1.597138	0.363536	0.760631
Cs3g08500	17.81549	22.12598	8.678505	12.63128	3.670292	2.158219	2.368988	2.496207	0.544792	0.945659
Cs3g08530	11.19697	10.88807	13.45243	10.30056	12.63316	7.31813	10.19371	9.724043	1.183279	0.895353
Cs3g08550	0.497694	0.392413	1.893093	1.745117	1.887647	2.121959	3.396494	4.576272	1.199443	1.946879
Cs3g08570	0.471857	0.23728	1.056889	1.08548	1.103777	1.524847	1.926576	2.998967	0.933303	1.563391
Cs3g08580	0.192045	0.143877	0.751704	0.662164	0.7227	0.802253	1.209771	1.794834	0.49584	0.763737
Cs3g08600	0.369436	0.284664	1.068981	1.078071	0.939046	1.006979	1.554195	2.094119	0.470494	0.945174
Cs3g08680	3.423258	2.889752	24.81954	17.14546	34.64029	34.09304	36.78307	28.68103	35.40035	13.15011
Cs3g08810	5.003182	8.915754	1.648343	2.976408	1.152144	1.277033	1.005593	2.347308	0.17565	0.367471
Cs3g08920	4.90907	3.320542	3.302439	3.713994	0.624003	1.86156	0.678637	0.555722	0.016751	0.039641
Cs3g09000	6.518886	10.7046	5.459453	7.557482	0.320323	1.168231	2.125095	10.65101	9.130311	60.90996
Cs3g09020	0.064055	0.077458	0.632595	0.597047	1.09251	1.246993	1.354491	3.264564	5.07266	5.863032

Cs3g09030	3.130574	2.527479	18.84028	15.38667	28.48104	33.00523	28.26967	23.87079	31.0234	13.26336
Cs3g09040	2.65964	2.536893	16.84892	12.98831	26.63648	28.71193	26.13334	20.44231	28.14514	10.57441
Cs3g09086	2.201573	2.881728	2.600491	2.922807	3.126832	4.5721	1.842202	4.112953	2.733748	2.602796
Cs3g09090	3.917575	3.355838	2.749389	2.317832	1.290678	0.95572	1.451476	1.470377	1.056924	0.499897
Cs3g09100	17.51796	23.25161	19.23789	23.93035	7.304118	8.759417	1.561471	4.213107	1.104839	5.107963
Cs3g09120	249.7838	167.3615	249.1055	176.7577	299.8876	373.8145	115.974	153.1342	2.777421	2.82004
Cs3g09140	18.36096	12.96139	20.95412	9.440895	16.64006	15.97806	2.857934	6.063798	0.657863	0.829134
Cs3g09175	1.207871	0.950363	1.161052	0.762196	0.543792	0.894478	0.27626	1.062698	0.009246	0
Cs3g09190	59.6096	65.62397	57.59432	60.25238	42.31098	33.32925	17.78542	31.48872	12.1178	23.83049
Cs3g09270	19.64385	20.74997	17.09785	14.5395	20.32841	28.11224	25.23994	16.31614	27.3731	6.507256
Cs3g09280	7.581282	7.460579	7.95529	8.68253	1.119522	3.712647	4.96065	6.146363	9.704582	27.72349
Cs3g09320	1.018943	0.843332	3.815992	2.149503	7.285682	5.030206	4.169157	3.613534	5.478263	6.436159
Cs3g09325	6.368315	7.240431	8.218734	6.148903	10.34267	9.485705	2.361783	18.28812	6.980392	5.245262
Cs3g09430	1.595851	1.641195	1.518943	1.812803	1.754885	2.387995	0.79617	2.386091	2.291564	3.011505
Cs3g09495	5.901199	6.30182	7.794539	8.249323	7.371105	8.546484	3.945741	9.809454	6.99449	9.36783
Cs3g09510	1.811721	1.572058	1.437639	1.496045	3.012081	1.762926	8.430643	1.927702	0.543557	1.239092
Cs3g09710	0.653776	0.289668	2.455342	2.245834	1.461906	3.632486	0.444722	1.041975	0.224872	0.626438
Cs3g09720	0.650976	0.868224	2.589102	1.739253	6.877006	6.836888	4.472707	4.505474	11.02182	12.73649
Cs3g09760	1.032327	0.996209	1.074955	1.196225	0.56346	1.217827	0	0.521833	0.060693	1.532927
Cs3g09790	3.101722	3.284746	4.866127	4.471003	6.94072	6.332039	3.03363	7.252379	3.262639	3.798221
Cs3g09810	3.958772	2.523779	2.492113	1.129241	3.424024	2.334511	2.437578	2.843724	0.263323	0.216982
Cs3g09900	39.35678	37.49854	19.71069	19.54296	5.637174	4.383416	0.675106	3.350572	0.65757	2.1872
Cs3g10010	2.89838	2.681749	6.96867	9.853785	2.636806	4.993716	0.592028	6.049942	2.142428	3.783197
Cs3g10020	1.179584	2.021701	1.554869	1.844335	1.593116	2.091763	0.378118	1.941536	0.845278	1.329893
Cs3g10030	2.384657	2.264007	3.426969	6.219843	2.644755	3.609467	0.110009	1.32092	0	0.147724
Cs3g10050	0.922015	0.960511	2.024954	2.646347	0.682188	1.451274	0.075442	1.420776	0.098084	0.184093
Cs3g10085	11.01601	9.566922	10.40067	7.882823	5.17117	6.327057	1.294224	3.484306	2.178105	2.395769
Cs3g10090	2.200229	2.275525	10.17205	12.53782	6.095452	9.252989	0.671622	9.729557	0.170109	0.284425
Cs3g10110	3.003167	2.389102	8.168701	12.84779	3.939818	5.930417	0.226447	2.43769	0.243647	0.259132
Cs3g10140	0.307385	0.381735	1.163446	2.240899	0.462344	1.02561	0.253297	2.039321	0.341691	0.329964
Cs3g10190	0.552607	0.373142	1.592036	1.548646	2.183971	4.705675	0.896572	1.332096	0.152768	0.193443
Cs3g10250	26.09063	35.1964	18.26629	21.28225	52.36506	47.29753	48.75285	35.47486	88.81363	41.63656

Cs3g10350	0.815167	0.722115	1.153039	0.448289	0	0	0	0	0	0
Cs3g10390	19.97682	20.34017	44.14244	34.68262	87.65664	52.17843	95.87436	148.0476	77.30595	77.58347
Cs3g10410	717.4227	789.5023	413.7523	431.8585	114.9971	229.1416	125.5865	406.8417	49.93794	107.8761
Cs3g10420	8.137189	8.991225	2.821443	2.113548	4.013532	3.677239	4.013498	4.735449	12.60827	6.949116
Cs3g10430	7.113911	7.65762	10.23557	12.26803	34.71548	30.94147	38.086	39.0903	127.8766	73.44669
Cs3g10440	36.13874	28.12621	34.59485	49.44972	9.55139	14.8094	12.7573	19.5463	35.89672	92.26838
Cs3g10520	3.513446	3.207162	8.448018	7.220007	9.212123	9.793647	2.710573	5.916692	2.46828	4.042776
Cs3g10550	11.32292	8.509381	12.50374	7.476803	48.84941	38.04342	98.26358	53.31729	10.59713	11.01443
Cs3g10580	39.56113	42.98243	63.52669	61.11316	44.48375	35.72414	23.55209	28.56909	8.549846	13.53351
Cs3g10620	0.047267	0.071631	0.063801	0.311233	10.47412	9.426603	3.15426	0.243083	98.62567	7.740564
Cs3g10660	0.035486	0.016911	0.41523	0.240995	1.900328	1.664815	0.647783	0.602402	10.20139	1.297651
Cs3g10690	2.769586	3.826722	8.315788	7.419623	7.876071	7.916159	7.772609	12.96764	7.62598	12.83906
Cs3g10710	0.127686	0.078855	0.323696	0.440478	1.905276	0.926571	1.04046	1.466966	0.039113	0.032712
Cs3g10720	1.832465	2.390776	5.078859	5.133042	11.24053	9.423619	16.19969	28.30128	7.001083	8.207518
Cs3g10740	1.605733	2.46893	2.293341	2.334168	2.79194	2.375351	2.017042	1.709909	0.195621	1.314566
Cs3g10860	5.386389	6.971756	3.061059	3.321367	2.658716	2.952176	2.943161	2.840559	1.554195	2.979843
Cs3g10930	79.91799	101.6062	70.54269	70.32981	25.96656	27.54375	18.31363	29.43308	2.05247	1.834678
Cs3g10950	5.008778	9.552553	4.975959	10.26513	2.296486	4.090033	2.86115	1.776877	1.294289	0.484412
Cs3g10980	35.37383	47.60467	65.02083	58.09796	87.35853	102.0486	21.20413	36.34672	4.882447	5.536451
Cs3g11020	6.538878	4.846289	11.81978	13.43279	8.045335	28.59467	5.677689	9.968164	13.13107	7.207034
Cs3g11030	2.541527	1.62201	4.387894	4.086783	3.154365	9.215512	2.10713	2.936607	4.871364	1.603851
Cs3g11050	3.234035	2.092778	5.265367	5.260293	3.877601	9.673204	2.462966	3.414884	8.487655	8.447222
Cs3g11080	2.409073	1.557429	1.823935	1.305431	1.702008	3.248181	0.368376	1.479576	0.985832	0.215813
Cs3g11110	20.65672	13.03535	16.62571	17.34025	16.87974	46.94962	4.703649	7.02163	17.51536	16.27895
Cs3g11120	3.151186	1.790514	3.708866	2.516371	2.150834	3.456024	2.166582	1.565085	2.676929	1.501918
Cs3g11160	4.705198	2.177219	4.392453	3.808523	3.708502	7.523124	3.049166	2.763126	6.900139	2.484708
Cs3g11170	15.72187	16.27273	26.17639	26.93959	33.85975	42.7846	68.71816	64.65891	49.81837	54.98132
Cs3g11180	31.60443	32.58225	51.92023	48.24827	73.20408	78.04693	156.7392	114.5602	92.93832	83.65891
Cs3g11270	7.646064	8.521217	15.45475	14.02849	28.12966	30.23188	28.57423	68.28719	37.59886	43.17643
Cs3g11280	2.0751	1.600093	1.819733	1.644915	2.397042	3.123764	1.315705	2.897326	6.008827	4.4231
Cs3g11290	5.882509	5.239904	4.657504	5.040202	1.130145	4.065092	2.180811	15.09113	16.26905	75.81747
Cs3g11300	0.162798	0.143766	0.023945	0.110987	0.021646	0.037216	0.254166	0.445068	0.447904	6.413054

Cs3g11330	2.244533	3.368859	2.697497	2.525525	1.346772	1.626586	0.589706	1.673369	0.284934	0.941847
Cs3g11400	4.810258	5.076937	3.715539	4.350101	2.77438	3.202813	0.569486	2.868128	1.683852	1.348884
Cs3g11420	27.14534	22.74772	25.41914	35.37529	18.3387	44.31173	11.15921	38.20958	46.81526	89.94991
Cs3g11590	1.134977	2.037785	4.896642	4.643647	2.70112	9.631891	1.888463	7.249989	1.714427	5.298903
Cs3g11610	5.031798	3.87848	1.866184	1.492688	2.315004	1.658343	1.838098	2.184376	3.354193	2.630695
Cs3g11620	2.285862	1.705149	4.132687	4.808481	0.52083	3.62357	1.966089	4.418468	6.846517	14.42746
Cs3g11700	0.433293	0.412584	2.928716	3.660981	1.683267	5.76761	2.036368	5.28484	1.40716	3.701038
Cs3g11710	0.176409	0.200475	1.442715	1.568871	2.27034	8.441633	7.193497	17.50972	4.782137	8.173665
Cs3g11715	1.949492	1.646191	2.397459	3.230226	2.251	2.913573	0.924041	3.880567	0.612643	1.15021
Cs3g11720	1.369621	1.310249	4.206972	3.679366	2.766373	4.442118	1.138081	2.143189	1.205879	2.69611
Cs3g11760	4.231582	3.758379	5.154687	5.918024	15.09428	9.320023	32.39107	15.83237	103.9047	61.15582
Cs3g11770	4.525511	3.901661	10.02704	9.513087	4.608842	7.123451	2.065593	5.777721	2.680536	3.934235
Cs3g11790	2.077161	1.982279	2.353044	2.193285	7.95678	4.930435	20.89683	12.65115	51.73992	37.20408
Cs3g11850	7.386173	5.838665	8.125807	7.934755	14.16774	16.11652	7.236665	6.744172	24.76503	8.677451
Cs3g11880	14.10598	13.01677	16.14718	12.88548	16.97929	12.80908	14.27078	13.49658	9.034737	3.819924
Cs3g12020	0	0.037564	0.011759	0.036894	0	0.025667	0	0.033268	21.36399	77.69074
Cs3g12130	2.968489	3.720792	5.091417	5.677108	4.509927	4.357992	7.642392	10.8257	22.8764	20.01383
Cs3g12160	1.097324	0.739257	0.9118	0.653017	0.342728	0.599047	0.109126	0.911885	0.301579	0.066577
Cs3g12170	6.779614	8.351744	4.460402	5.502346	2.040752	3.093788	2.652517	6.132367	2.552691	5.80154
Cs3g12200	0.667922	0.712734	0.431731	0.465832	0.306838	0.868015	0.12189	0.94014	0.440332	0.053637
Cs3g12220	22.06558	20.70144	23.64125	32.12912	8.545992	24.56596	12.74451	26.37729	66.8175	148.3102
Cs3g12230	1.461608	1.622562	12.22397	13.45647	10.75715	40.13162	8.041796	29.9708	22.57456	47.22354
Cs3g12285	4.868118	5.655186	7.201348	6.035087	5.926783	6.000454	1.466304	6.240323	3.104737	2.820092
Cs3g12370	1.930309	1.241072	4.026859	3.687436	5.547435	3.597699	6.210115	6.692933	8.218283	4.685497
Cs3g12380	2.667309	3.045511	3.309777	2.84915	1.244282	2.16427	0.369555	2.28324	0.555629	0.46021
Cs3g12420	0.668015	0.796379	1.007726	1.246198	1.340494	1.860121	0.746262	1.77079	0.450649	1.347964
Cs3g12460	0.26174	0.237513	1.44238	1.689909	0.390876	0.862154	0.178737	1.248086	0.711136	3.282411
Cs3g12510	44.92755	68.35699	24.47701	41.64125	7.856554	13.19538	3.226979	12.77071	0.98528	1.017992
Cs3g12520	9.055276	11.39118	11.26796	10.5673	7.163433	8.304823	3.155643	8.728953	1.070531	3.471828
Cs3g12560	13.01023	11.31481	4.742252	5.119559	1.698512	3.549254	0.408269	1.671503	0.313189	0.038358
Cs3g12570	18.94113	16.53808	7.993048	10.0002	9.775647	10.986	7.071663	8.546653	7.498518	8.445976
Cs3g12580	15.63186	14.91939	9.420406	10.0513	7.361167	9.824599	1.586303	3.914575	1.685064	0.80711

Cs3g12630	7.969866	14.32549	2.105855	4.428448	1.665767	2.124038	0.668364	3.033327	0.565774	0.984984
Cs3g12640	57.22995	107.7335	10.75857	31.64166	14.20284	7.952423	4.607102	14.21062	0.760829	1.070556
Cs3g12730	6.918018	7.723858	6.903772	8.699639	1.255732	2.775957	1.022192	5.6783	3.994325	7.241143
Cs3g12850	3.222371	2.933928	6.316762	5.321083	1.193339	1.853774	1.530643	3.640366	4.626829	9.006053
Cs3g12910	1.358902	0.930273	4.574902	2.816724	8.652623	8.565401	9.048096	4.739333	6.626021	7.623391
Cs3g12935	2.288503	1.824762	9.487011	5.118415	5.412803	9.601739	2.370566	18.56302	0.085886	0.371237
Cs3g13030	5.030238	3.10244	2.952142	2.76361	3.209891	6.543695	1.104377	2.853233	1.800644	0.615167
Cs3g13040	6.751556	7.206854	6.692507	9.570254	5.090239	9.836477	4.679638	7.654646	9.904156	15.48369
Cs3g13130	4.236249	5.159183	4.488308	3.917671	1.463583	1.947765	0.527656	1.531479	1.406288	2.084848
Cs3g13300	44.49907	59.79082	62.16088	77.87104	12.48749	18.55335	45.35403	51.05117	12.74557	36.09352
Cs3g13320	9.126848	18.37173	17.79091	21.72473	1.749594	4.391956	3.062502	9.448868	3.386845	8.099049
Cs3g13340	1.651587	1.513218	2.243782	1.872833	0.760281	1.128552	0.770317	1.810793	0.290826	0.971015
Cs3g13400	3.600292	4.741827	4.11244	4.947246	4.261812	5.308445	2.758885	9.300636	6.609935	11.05175
Cs3g13420	4.268134	4.980493	5.126983	5.736631	4.823034	5.188088	3.544052	7.829712	5.220156	5.720834
Cs3g13440	6.190969	5.72299	7.676261	6.752064	1.118718	2.406143	1.484999	1.466271	0.061404	0.053682
Cs3g13500	6.929035	6.891961	6.60873	8.065061	7.052619	9.933703	3.133505	7.773125	5.184055	7.984534
Cs3g13560	7.112324	7.755539	8.688127	10.0488	8.99336	14.21636	3.668847	12.28462	8.238164	10.90718
Cs3g13600	15.97969	16.95441	16.31449	15.72239	6.278548	7.177465	5.852152	9.170734	3.090638	4.365018
Cs3g13680	6.224611	7.82766	5.905585	8.646019	1.380056	2.92519	2.421223	7.387865	3.345716	14.21879
Cs3g13710	12.64236	13.74772	11.10443	12.80958	2.147818	3.54353	3.364883	11.41414	4.444103	12.84028
Cs3g13770	31.36988	30.90611	57.59381	69.40814	64.31952	84.07736	72.12252	74.09789	151.3901	175.4602
Cs3g13820	6.573498	8.705319	4.783056	7.457725	2.871796	2.762808	2.354351	5.31093	0.42804	0.430447
Cs3g13930	3.814288	3.107524	3.791415	3.833641	7.256155	7.213709	0.907003	1.312812	0.182819	0.55253
Cs3g13980	0.560282	0.649881	2.016831	1.733089	1.215714	2.744012	0.495234	1.78435	0.741364	1.614782
Cs3g14000	8.498361	13.05491	7.147609	9.073335	5.136967	4.579751	3.596926	8.757149	3.206243	1.964831
Cs3g14050	13.49027	15.66695	9.883321	12.29806	10.99949	11.40395	4.665987	9.542612	1.765764	2.006438
Cs3g14060	0.804177	0.585816	1.504333	1.469507	2.063984	3.161109	0.713757	2.292633	1.35233	1.474965
Cs3g14130	4.771676	12.82072	0.662146	3.278402	0.443952	0.52432	0.388966	1.443661	0.080958	0.476937
Cs3g14190	4.938366	6.088965	1.31116	3.560838	0.310629	0.380676	0.354565	0.296706	0.135972	0.230314
Cs3g14220	0.591036	0.861954	2.169584	1.978775	8.099629	3.529704	6.652132	5.596215	3.671862	4.085228
Cs3g14240	0.225979	0.042222	0.686115	0.362454	2.562452	1.052195	1.831047	3.966247	1.415003	3.572202
Cs3g14390	22.94572	13.65658	32.39672	25.06092	101.0533	150.6984	127.9896	59.23481	230.9757	164.8849

Cs3g14500	3.570878	4.884932	6.635568	4.318643	5.860591	5.064158	4.643515	10.35483	10.12225	10.69187
Cs3g14550	59.04972	48.12482	23.66465	24.4826	84.39862	25.79764	152.4827	53.79757	24.1408	15.25961
Cs3g14560	13.09893	12.56261	20.93176	18.44287	30.17893	27.69335	30.11868	31.56139	19.30348	16.7638
Cs3g14580	0.039609	0.108009	0.203144	0.336689	1.720884	9.491683	3.927526	1.610985	5.495681	15.0504
Cs3g14610	0.838518	0.813493	3.678815	2.688805	6.164289	16.52116	29.21031	17.86295	44.34472	28.84997
Cs3g14620	23.70539	26.25258	19.01329	23.50231	4.113483	5.012133	3.23331	1.576961	0.273928	0.212152
Cs3g14660	4.655511	4.40097	1.432141	0.83444	1.276084	1.329253	0.824278	1.787943	1.802757	1.158424
Cs3g14680	0.060086	0.323906	0.295806	0.899683	28.02215	41.48427	43.0659	42.16769	1.528382	1.107377
Cs3g14750	0.813807	0.799739	0.943308	0.895921	0.746973	0.513777	1.50564	1.660942	0.823625	1.390873
Cs3g14760	0.081797	0.061695	0.295154	0.371681	0.260756	2.807361	0.321097	0.22018	0.062931	0.110475
Cs3g14790	35.23628	29.90071	27.13306	27.23046	15.10112	17.51148	14.9048	13.6822	23.4264	28.5292
Cs3g14880	140.1912	184.1416	148.9228	147.0388	153.7377	144.6999	50.19931	79.02527	76.80538	86.1735
Cs3g14900	5.804444	6.141035	7.644172	8.446903	11.49827	14.42927	16.42404	19.84245	16.30532	16.60864
Cs3g14930	16.55198	6.014191	51.86626	25.83964	134.7227	162.546	63.18694	110.2333	141.8642	172.8432
Cs3g14940	20.89435	13.80731	26.69969	41.75937	1.162389	8.901499	5.90962	8.903859	10.90381	22.75565
Cs3g14990	0.363959	0.386355	0.776886	0.353341	0.64919	0.868236	0.996578	0.462622	0.476126	0.969927
Cs3g15030	49.28724	44.44487	92.10586	76.3629	149.9931	112.235	103.4988	137.3582	88.27014	84.72739
Cs3g15140	1.502127	1.790037	4.596061	3.834708	4.463886	5.938726	5.029085	6.847898	0.327321	0.389989
Cs3g15200	6.233211	5.881293	4.399756	3.41793	2.788532	3.100797	1.628029	2.392387	0.54594	0.411548
Cs3g15290	4.834067	7.193302	3.093422	4.82351	2.288968	2.150571	2.327585	2.990022	1.151757	2.027936
Cs3g15390	0.124149	0.027136	6.959627	0.21386	8.265836	0	15.62887	0.045717	0	0
Cs3g15420	11.09613	13.97753	5.648091	8.414011	2.411783	3.315165	1.196756	1.830922	0.679856	1.965568
Cs3g15450	62.34961	86.05477	27.31522	53.48446	9.649781	12.17105	6.684958	16.798	6.825206	12.27481
Cs3g15460	1003.374	593.6437	156.3103	210.7899	68.59393	79.93588	72.17025	30.01075	98.71797	58.71279
Cs3g15530	0.242444	0.147299	2.216551	1.258392	2.301132	3.214199	3.521515	3.748621	4.853794	6.198003
Cs3g15540	16.94244	15.27558	49.58681	32.06363	92.53498	57.56544	248.4323	179.2909	125.5917	135.1584
Cs3g15630	6.182513	4.453579	8.004586	7.47694	29.79568	29.6796	32.51919	25.21764	51.38081	24.01515
Cs3g15640	3.718894	3.404024	3.86282	4.100438	13.77003	12.93059	15.61462	12.97911	19.562	9.524548
Cs3g15670	0.106919	0.164335	1.286459	0.860972	1.355202	1.910189	1.800314	2.795876	4.448432	12.54009
Cs3g15690	9.187303	11.08992	7.85186	8.890435	4.48432	8.955674	4.966686	54.99101	6.138696	13.9028
Cs3g15740	8.355194	7.364948	9.761538	6.135376	57.07325	26.31091	104.1705	34.38738	14.48464	3.64154
Cs3g15770	1.533334	1.764441	0.842637	1.272427	0.249152	0.491495	1.009568	2.322154	3.146928	10.77864

Cs3g15780	21.42218	22.29732	18.49233	22.30057	6.654746	10.16471	12.65982	17.94884	18.04839	52.13863
Cs3g15800	134.3576	214.2552	31.20986	57.28711	26.24255	25.87274	67.89025	71.02675	1.254952	1.816483
Cs3g15830	19.56735	18.92162	29.27376	25.55134	63.01619	85.9494	116.3904	236.2905	68.37539	107.9691
Cs3g15900	82.24343	93.28049	83.18157	118.3438	21.4432	32.44033	102.3388	108.9024	137.2662	183.8009
Cs3g16000	20.05368	21.40374	29.85582	28.019	58.43086	64.60448	81.44137	92.34265	63.74548	50.77642
Cs3g16040	54.97773	77.67025	94.69597	119.4435	45.09161	35.39061	53.02859	69.3139	53.85223	84.54245
Cs3g16070	30.27006	31.19063	38.34454	41.47907	36.6701	40.84816	20.41075	50.63969	28.26043	64.04222
Cs3g16100	2.169781	2.246621	5.092049	4.946293	5.47742	12.88372	2.879213	7.253866	3.71605	1.806071
Cs3g16110	3.913325	4.533542	6.000932	5.723025	10.09785	9.364531	9.559537	5.601673	6.344896	5.175756
Cs3g16130	0.080345	0.263504	1.010587	0.444507	1.977565	0.920373	3.497573	5.083343	1.901725	2.792114
Cs3g16140	1.577269	1.413779	0.741832	0.478561	1.035961	0.326768	1.007226	0.550982	0.092243	0.029953
Cs3g16190	1.157657	0.606864	0.802509	1.559808	2.694416	2.489268	1.984095	3.536579	3.564204	4.203104
Cs3g16240	58.13293	24.24	15.87052	21.22059	4.702406	11.5543	7.974668	10.2754	27.27802	34.95289
Cs3g16250	18.23967	21.0439	4.923126	10.69769	10.66732	8.725138	17.36227	9.856637	0.636945	0.399728
Cs3g16290	8.481516	12.46705	12.01976	10.00593	3.615326	2.36199	3.366557	7.961931	0	0
Cs3g16310	0.96198	1.700921	2.297661	1.742602	1.996371	0.671199	1.825856	4.032266	0.063933	0.20229
Cs3g16330	4.354613	6.159919	5.831992	5.669697	3.28845	3.744388	3.167635	9.774955	0.261661	0.827334
Cs3g16350	6.360177	5.652521	4.304302	5.298184	2.450715	2.767485	1.447278	1.932913	3.127797	6.368747
Cs3g16370	0.819701	0.949292	0.325053	0.806092	0.147794	0.337048	0.247764	0.574398	0.279808	0.826315
Cs3g16380	13.76342	26.42688	11.36629	15.09226	1.803441	3.023266	8.361229	36.06101	4.518673	24.71018
Cs3g16400	0.009602	0.018069	0.37802	0.207842	1.333765	5.117535	16.73589	16.23397	19.72452	21.6509
Cs3g16410	7.094908	5.51906	6.422747	6.412976	4.279046	4.058822	3.186092	7.074429	3.419833	6.217276
Cs3g16430	0.145055	0.381659	0.595442	0.34719	0.203787	0.244555	1.420427	2.883127	10.80426	49.82514
Cs3g16440	42.56276	32.70727	44.64183	53.9064	31.47207	74.80813	30.57021	46.36474	24.89815	68.25757
Cs3g16460	0.080614	0.063653	1.085944	1.013061	1.17563	1.657213	1.004005	1.544183	0.699451	2.462796
Cs3g16480	14.03684	13.04111	21.37775	25.57691	10.00008	23.62369	13.39106	15.62388	66.34564	90.83714
Cs3g16490	2.142352	2.681055	2.918528	2.771435	1.196849	2.250877	1.486412	3.330661	8.601203	22.74882
Cs3g16520	8.840063	11.76721	12.28814	11.06787	7.033792	7.930871	3.26585	5.716665	0.294081	0.763458
Cs3g16525	7.719985	7.798152	9.422205	9.77429	9.387477	9.124055	3.548113	9.728635	0.377283	1.332242
Cs3g16530	20.09708	20.69281	18.20172	19.69341	7.831192	7.943368	8.728368	13.59243	0.972304	3.270244
Cs3g16570	17.49509	5.41358	1.894641	2.58904	6.351771	5.703568	12.03241	3.931356	34.6144	12.39999
Cs3g16590	22.00528	23.23582	19.62256	16.53513	11.6623	11.48528	8.281611	13.02839	2.281455	5.364891

Cs3g16600	5.681916	6.043846	2.901921	3.28325	1.782012	2.543566	2.080592	1.97218	1.094656	1.142863
Cs3g16620	6.261762	0.743746	0.540144	1.017559	1.02989	3.110208	0.463348	0.440221	12.89938	0.833896
Cs3g16630	0.995084	0.426583	0.572217	0.587911	1.176087	0.794358	0.520252	0.394956	0.233081	0.118544
Cs3g16640	18.00327	17.86654	30.35477	25.7589	41.68549	36.22067	42.41419	42.30397	43.30752	34.72497
Cs3g16650	0.235639	0.107799	0.468182	0.426174	2.245894	2.010864	2.398696	1.615977	5.51898	1.110595
Cs3g16660	8.383463	5.668793	8.627166	7.234229	11.88411	8.47878	7.336543	16.41357	16.12028	16.49422
Cs3g16700	11.40953	12.61618	32.15759	22.4823	136.6333	159.108	397.4988	439.2014	398.1478	641.7514
Cs3g16740	15.03271	19.63653	7.191939	10.77151	1.574294	2.467877	5.628847	7.847572	2.713227	7.795009
Cs3g16850	0.211081	0.319489	0.084935	0.11887	0.301191	0.224	1.51493	0.130339	0.023694	0.006618
Cs3g16860	10.29521	9.947085	17.24358	15.49691	29.50137	30.51403	38.88339	26.82946	21.39393	22.54757
Cs3g16892	0.921014	0.491394	1.056188	0.444183	1.24063	1.03195	0.231659	0.408711	0.133635	0.325781
Cs3g16896	1.967281	0.881928	1.780314	0.837583	3.29529	2.71313	0.301545	0.74938	0.907827	1.170581
Cs3g16940	12.7496	12.30493	35.81779	29.67216	44.94839	50.84677	52.99548	93.44334	40.85418	90.55996
Cs3g16960	4.394999	3.420747	10.76213	8.142304	13.14152	6.357025	7.181839	8.632775	1.759324	5.840144
Cs3g17010	16.58821	17.60989	31.24023	30.16937	47.98295	39.06168	69.41528	59.83104	168.096	212.0184
Cs3g17035	2.310249	2.205262	2.579901	1.344473	1.760601	0.917597	2.538338	0.640725	0.979686	0.236726
Cs3g17120	24.8277	25.19618	29.90524	16.64373	15.2572	21.67147	19.70266	18.47334	12.12363	12.49844
Cs3g17290	4.279385	5.284081	3.574867	4.095546	2.32845	2.486556	0.908622	2.75216	0.191316	0.28878
Cs3g17310	36.51241	55.79053	24.95348	33.4418	8.334296	10.67138	6.616665	16.09093	3.017371	7.678888
Cs3g17390	36.04892	94.45814	107.5316	148.4888	8.386303	16.84285	67.09828	115.1234	55.56115	317.918
Cs3g17500	13.01257	27.32486	11.86992	10.05524	11.43668	6.909683	10.4982	17.45861	6.350352	22.54066
Cs3g17520	7.917385	8.241979	14.83153	9.977928	25.81495	28.07239	17.26934	35.92096	27.91886	56.56309
Cs3g17620	39.06209	45.10122	49.1848	49.80815	83.56641	66.2104	175.4812	202.5075	81.70943	86.8619
Cs3g17630	5.290545	7.785018	6.666563	6.979186	12.15018	10.75644	21.58755	24.79073	15.39717	11.49162
Cs3g17650	16.66589	13.89525	22.37134	17.37639	39.43641	31.24074	93.58784	34.71576	12.45145	10.17874
Cs3g17660	0.825667	1.072365	1.459504	1.075868	0.934067	1.295958	0.367693	1.428001	0.06788	0.050344
Cs3g17760	20.38718	45.07299	2.709082	7.023423	1.451731	2.915591	0.179531	1.067311	0.693878	0.419336
Cs3g17830	2.458257	2.116473	0.98556	1.177848	1.620253	1.329551	3.640022	2.499282	0.941183	1.723907
Cs3g17870	23.30593	20.17831	10.89714	13.12679	12.76254	36.14376	23.05754	31.10327	55.35374	11.18627
Cs3g17890	8.079299	20.93449	35.07679	29.53873	3.031831	5.801966	4.123437	22.76809	1.154602	2.914536
Cs3g17900	52.1253	51.62773	45.60781	38.98663	25.82358	27.74544	8.63872	14.01593	10.98846	7.041643
Cs3g17940	8.763117	9.817615	29.45686	19.68888	36.83295	30.19267	16.80095	16.95569	6.105724	8.923301

Cs3g17970	32.12257	38.97064	28.06233	32.06818	12.14166	10.74555	2.535336	3.482965	0.934388	0.945627
Cs3g17990	20.23164	17.4801	20.7247	21.2551	29.19115	38.40174	48.87511	51.0927	88.07209	92.5551
Cs3g18030	121.8789	105.4629	195.0437	203.9212	208.7266	284.9039	157.1872	74.76229	239.8556	317.4588
Cs3g18050	7.807831	10.02527	5.489805	10.64652	0.608438	1.64379	1.768982	10.90938	0.805001	9.774547
Cs3g18060	32.54334	44.13309	19.01901	39.01892	1.675388	7.443245	27.53525	132.7636	12.26097	96.70655
Cs3g18070	19.6715	56.06797	18.63434	42.79391	0.870052	8.258149	23.1562	222.3011	14.97858	201.5355
Cs3g18080	13.55328	9.701827	8.889334	8.779926	3.12475	7.647031	7.478247	4.534896	6.545461	7.008746
Cs3g18090	10.11935	3.904886	15.05367	15.07001	12.61678	44.61511	31.09075	16.06848	148.8325	65.051
Cs3g18100	11.52431	4.406402	17.59432	15.74412	15.23909	49.79888	34.50885	17.33233	160.5541	61.22629
Cs3g18110	0.56725	0.585706	1.347752	0.711262	0.731165	2.077388	1.726046	1.582374	0.737622	0.548465
Cs3g18240	30.28382	44.67055	15.17011	23.86298	12.2318	12.27602	13.0944	14.40722	2.332173	2.70259
Cs3g18260	4.205818	3.750018	5.706864	3.798621	3.633423	3.455849	0.953514	3.695	0.382348	0.20671
Cs3g18280	4.513095	5.330215	3.071135	3.150903	4.697276	4.643146	9.536058	8.057654	26.79045	41.0944
Cs3g18330	2.24161	3.210371	3.178521	3.788478	5.452014	10.87425	17.69891	10.83228	310.9633	160.058
Cs3g18370	2.848929	2.651356	9.055466	6.044079	9.31454	4.578027	6.264111	4.39381	1.375135	1.800858
Cs3g18400	7.861555	6.624527	8.829535	7.04025	9.199222	8.046445	7.321461	4.778379	10.6636	4.458758
Cs3g18410	4.757405	3.584733	6.804218	6.118001	8.751416	7.391935	5.559808	6.905114	33.60203	12.84471
Cs3g18450	7.950855	8.50378	5.465045	6.757365	0.56201	0.940097	1.792125	2.943917	4.162097	4.843745
Cs3g18490	77.65869	55.68189	31.68589	32.54376	10.4619	21.08828	6.351399	13.5766	1.048144	1.190506
Cs3g18500	0.991769	0.97177	0.268803	0.350497	0.238502	0.253414	0.282533	0.722718	0.041456	0.097664
Cs3g18540	8.24258	6.749632	27.96628	27.68497	38.40648	108.3989	29.1659	22.08674	48.81257	65.96326
Cs3g18580	1.584445	1.710799	4.420088	3.623225	0.259743	1.158014	0.828195	1.697443	0.342545	0.355221
Cs3g18590	4.586706	5.28448	7.382522	6.889412	12.89324	11.14752	12.84604	18.67455	14.4852	16.36846
Cs3g18610	2.787527	3.641616	6.991619	5.044993	3.034698	3.339008	2.084959	1.940233	0.698871	0.582272
Cs3g18650	17.77229	18.03939	30.46634	25.2078	48.53056	51.86165	45.73136	37.03357	49.5185	47.95928
Cs3g18660	3.495011	3.301671	3.048413	3.393917	1.836122	1.565892	0.997295	1.256756	0.170947	0.113311
Cs3g18690	365.0627	488.1216	232.7813	165.628	113.4111	20.95064	14.00969	18.04782	3.391263	1.022499
Cs3g18700	27.5094	34.86691	16.96427	18.05783	12.89364	6.377696	6.927241	9.912269	0.682478	1.762519
Cs3g18760	5.219293	8.047695	23.25146	35.2648	7.740761	12.24902	20.37407	44.24786	19.79959	51.50479
Cs3g18770	0.259074	0.698136	1.728636	1.696424	6.829578	6.356436	0.578869	1.716138	2.717842	2.847678
Cs3g18830	26.16752	22.93923	50.65947	40.12489	65.60549	59.78022	111.8022	87.87755	63.23727	71.03328
Cs3g18920	4.132874	4.001828	4.657015	3.745094	5.840082	6.493766	2.251391	7.47205	6.011948	2.968459

Cs3g18980	1.626397	1.057905	4.258215	2.305988	5.668198	4.657161	0.647192	1.411659	0.682153	0.388138
Cs3g19040	7.935003	7.329605	10.3034	9.264705	21.55496	22.09999	42.42886	24.75061	24.22266	17.19908
Cs3g19060	2.122116	2.27063	15.0292	10.93223	23.04919	20.85373	18.74599	22.51373	71.84451	17.43546
Cs3g19080	23.36186	22.61245	9.299977	10.03817	3.539005	3.139784	1.759641	4.284914	0.407187	1.028049
Cs3g19090	68.70656	73.70581	60.48795	70.99248	28.7775	27.36096	24.20697	51.86037	25.56787	59.8926
Cs3g19220	0.467122	0.272439	0.644432	0.435385	0.314896	0.827015	0.293273	0.965556	0.288751	0.307205
Cs3g19230	67.04558	69.14094	126.2068	109.2113	201.2814	172.2907	269.6745	268.0467	133.8849	164.502
Cs3g19320	16.61997	7.719745	8.541114	6.212525	7.294784	6.124722	9.536766	5.526418	27.74375	6.206361
Cs3g19340	12.58662	14.06818	16.56917	16.40876	15.43427	19.78828	7.602878	21.58057	13.83348	16.07002
Cs3g19350	6.058069	8.090278	4.677435	4.548971	0.915937	1.050329	0.110486	1.806423	0.032892	0.490591
Cs3g19360	234.2989	347.7296	239.5278	249.3037	57.74112	70.25262	19.6956	72.72802	57.27259	211.5205
Cs3g19380	67.69379	50.39817	110.1812	97.71102	153.7186	144.1053	235.6973	176.7476	158.1517	158.616
Cs3g19420	2.76219	5.722872	10.62112	13.28795	3.61572	13.03309	13.29435	87.74993	14.75408	43.62046
Cs3g19470	11.72608	12.26158	16.52198	18.02902	14.47296	18.9948	10.638	24.62934	16.09302	21.06452
Cs3g19540	1.106534	0.549762	0.07305	0.059934	0.016667	0	0	0.06633	0.015757	0
Cs3g19610	1.529285	1.537559	1.5157	1.507649	2.175974	2.212151	4.845249	6.785772	0.770732	1.806176
Cs3g19650	137.6453	227.6933	25.57221	76.04151	10.7206	3.994172	16.92679	16.4293	3.961865	1.790489
Cs3g19690	10.74086	31.0778	0.869896	3.743041	4.027549	3.434593	4.765084	8.085071	2.486412	1.957141
Cs3g19760	11.29214	10.04061	8.926793	11.8216	6.483129	15.00639	14.8671	10.27007	225.8786	89.92211
Cs3g19780	0.667598	0.479356	0.334924	0.443417	0.142136	0.198052	0.115615	1.443512	0.02909	0
Cs3g19840	31.03527	33.38281	33.49351	36.21173	24.26167	34.4776	6.232761	23.89476	5.664352	15.55111
Cs3g19930	1.49129	1.135185	1.845547	1.946119	1.229225	1.688281	0.464769	1.069813	0.878079	0.346386
Cs3g19970	0.359533	0.210536	0.829652	0.451393	0.64808	0.780307	0.851256	0.361577	0.884515	0.617508
Cs3g19990	0.765186	0.866341	1.421509	1.155613	4.28438	4.771127	12.28647	7.935722	8.754222	11.08409
Cs3g20040	62.33175	62.28174	58.01507	50.48651	60.37684	36.66996	48.41472	25.04198	4.54614	5.108164
Cs3g20050	1.063576	0.89983	1.570862	1.455097	1.14611	1.226319	1.581276	0.99109	0.966295	2.146656
Cs3g20065	6.095271	9.308187	11.8631	16.70548	0.254871	1.602767	2.041467	8.338453	0.522667	4.297794
Cs3g20070	9.73802	13.8767	18.16162	27.48404	0.836646	2.275494	4.539263	13.11171	0.97345	7.427164
Cs3g20250	11.13201	14.95026	9.968604	14.50052	0.945433	1.416131	0.898225	1.653112	0.139652	0.27769
Cs3g20290	9.284099	21.27994	12.6523	15.78111	7.899143	7.996955	4.275678	8.876969	4.795138	3.604887
Cs3g20300	76.7112	105.0377	19.404	12.84867	6.693306	3.86965	1.997702	3.136449	0.677198	0.36474
Cs3g20340	0.903253	0.930339	3.234154	2.087474	7.192168	5.418996	9.804378	4.675651	0.157062	0.431005

Cs3g20390	0.631944	0.721953	0.590255	0.542718	0.708905	0.652397	0.318928	0.867234	0.530684	0.340919
Cs3g20410	6.661353	8.449332	14.70861	12.90795	10.60061	7.950198	3.502393	5.453418	1.849055	2.212253
Cs3g20450	1.76476	4.456776	0.404213	1.04539	0.387247	0.30457	0.264117	0.786796	0.233626	0.311826
Cs3g20570	26.91928	27.91637	70.43714	65.41361	52.21898	76.77174	22.50453	45.48414	62.29989	134.2133
Cs3g20630	29.17564	59.06286	6.227365	19.10189	1.474674	1.929058	0.262292	1.490341	0.800966	0.3888
Cs3g20720	7.290822	6.780141	1.25212	2.10478	0.886317	0.96829	3.818489	3.421654	0.71186	0.747694
Cs3g20840	0.216485	0.092665	0.233446	0.182236	1.365727	0.430453	1.035911	12.37783	3.67903	4.21116
Cs3g20880	6.98564	7.32522	5.248603	5.678929	4.119244	5.753727	2.696806	7.207278	0.912643	0.805718
Cs3g20890	6.038717	4.937989	4.022144	5.054151	1.802589	4.804754	1.52718	4.388335	2.984268	5.643097
Cs3g20930	0.117248	0.193715	0.357992	0.332737	0.338187	0.887203	0	0.587907	0.187936	0.152724
Cs3g20970	0.176482	0.433193	0.468645	0.641037	11.03507	3.164838	10.15096	12.53002	9.182247	13.81231
Cs3g21010	1.718646	2.357153	2.040505	1.712195	1.576425	2.243843	0.710568	2.666598	0.130927	0.366262
Cs3g21070	33.12666	14.73995	17.33132	22.93359	10.98291	42.44646	42.63942	28.70304	204.43	135.3569
Cs3g21075	155.7561	93.65368	549.0606	414.1788	1690.271	837.2334	247.7337	665.2446	425.3006	314.8002
Cs3g21080	0.248939	0.26113	1.611746	1.660988	4.798084	3.974652	1.014657	5.736835	3.002513	1.213637
Cs3g21105	29.74789	12.77572	14.84798	14.95633	18.10691	39.24508	33.26866	29.49608	214.8219	106.8829
Cs3g21130	20.93896	26.37494	35.58228	34.28033	15.2675	25.72198	28.6899	65.69058	42.778	93.26355
Cs3g21160	315.6528	251.4144	39.249	110.8644	36.04452	153.6029	64.85909	76.68676	30.19301	28.62177
Cs3g21380	107.5921	141.9093	72.47096	65.74464	18.36998	18.42753	36.77088	61.34718	19.45556	73.62866
Cs3g21530	233.3975	199.494	92.23027	88.11848	37.64188	34.35358	25.09256	23.78247	5.505205	1.689208
Cs3g21650	0.481746	0.525294	1.564162	1.427441	1.943433	2.337023	2.631949	2.141424	1.86078	2.269669
Cs3g21660	6.114277	1.8482	2.372356	2.434954	1.148795	5.077603	3.783975	1.530057	123.4308	55.56656
Cs3g21730	36.18231	13.58369	90.2528	78.55955	84.78593	122.3491	25.10815	43.13928	12.38335	23.06065
Cs3g21760	16.79111	21.93459	11.3949	15.92313	9.596102	9.534347	4.980868	14.66614	2.064124	5.580929
Cs3g21810	11.89392	9.634315	12.91133	11.24113	10.05228	17.70462	7.797975	18.60864	28.23424	16.72068
Cs3g21870	0.233269	0.229406	1.06759	0.580856	1.733841	2.173102	2.821139	4.090853	3.642657	1.33742
Cs3g21910	18.34642	34.48683	6.720768	15.98875	12.3435	10.37098	13.0896	26.91538	1.400005	1.462026
Cs3g22010	0.862423	0.505183	0.594905	0.671034	0.652128	0.653994	0.605344	0.663328	0.4509	0.284551
Cs3g22080	18.45	15.37596	11.76369	14.03925	19.57978	22.97344	27.33884	31.14138	62.95801	54.01193
Cs3g22170	0.004324	0.031338	0.119709	0.209859	0.694017	4.647159	5.141856	12.17172	3.638235	8.801778
Cs3g22180	19.18254	44.31086	8.630793	26.01314	12.68215	14.16095	19.65996	49.56553	7.100927	7.897191
Cs3g22190	0.973444	2.022891	0.956246	1.268422	0.084258	0.266505	0.04118	0.053473	0	0

Cs3g22210	4.169847	4.60115	4.7639	4.366438	3.798167	3.808419	2.456111	5.98355	1.867575	3.428415
Cs3g22260	10.43535	12.22662	8.423493	6.794101	7.754523	9.047965	3.694659	5.785726	0.16764	0.077703
Cs3g22330	15.73569	18.29528	14.02079	21.40836	6.959776	17.26694	19.86032	24.16087	4.74853	4.089736
Cs3g22440	5.858056	5.796728	5.947726	4.777234	5.111195	6.671108	3.713083	8.07979	6.514601	5.615892
Cs3g22560	42.8064	32.07872	8.866692	12.31181	15.00882	7.491809	17.74418	6.038128	0.448015	0.427067
Cs3g22600	0.047167	0.095134	0.026361	0.087907	0.086117	0.300931	0.943222	0.260019	3.263792	0.249844
Cs3g22710	3.586844	4.409372	3.166444	4.036242	1.353672	2.361323	0.293412	2.072633	0.963555	14.00679
Cs3g22760	1.259901	1.074983	1.450627	1.359167	2.948294	2.488247	1.937443	1.83617	1.957136	1.812042
Cs3g22800	22.95776	23.14836	40.96082	32.90701	46.91977	51.23552	22.26977	32.9157	5.011695	6.245501
Cs3g22850	30.62659	35.07477	32.19419	28.21351	21.65282	14.7186	30.2521	58.09939	15.23591	36.0548
Cs3g22870	7.531131	5.77126	4.554433	5.881181	5.181989	7.129204	2.143277	6.372889	0.809799	0.845367
Cs3g22900	0.800207	0.565948	1.267478	1.058394	1.096118	1.282314	0.406174	1.851422	0.97005	1.474416
Cs3g23010	15.02467	21.12626	21.99769	18.1906	26.06178	11.62142	15.04514	11.99948	2.463983	3.295722
Cs3g23020	23.96849	25.56219	30.69409	26.89006	40.11358	22.21933	27.00935	19.46456	21.71733	16.94657
Cs3g23050	0.067256	0.170094	0.88026	1.451852	0.287646	1.341129	2.58518	4.542716	1.200758	8.308843
Cs3g23070	46.48752	72.53718	86.66599	93.82714	47.02423	33.97061	80.41534	236.5935	54.60066	199.7122
Cs3g23080	1.154286	1.17933	1.897024	1.21993	2.238743	2.760594	5.637254	8.611754	4.908257	5.173252
Cs3g23100	3.397856	4.799909	5.669572	3.173771	17.21615	8.235997	5.567057	5.416333	3.876833	2.418097
Cs3g23110	1.819903	1.751039	13.19436	7.388695	78.72065	58.40142	218.9219	97.89001	41.71042	39.70268
Cs3g23170	2.150812	1.508518	2.813784	6.940249	0.276336	1.823149	0.075396	0.850551	0.168214	0.143041
Cs3g23220	2.238996	4.304717	1.230595	1.571988	1.615024	1.498613	2.04059	3.196403	1.103932	0.675301
Cs3g23270	17.93642	13.77924	19.75199	18.28759	12.17432	15.77467	14.16587	20.26545	62.89018	23.70909
Cs3g23310	18.12854	14.56207	16.14217	18.5516	14.05716	29.59414	24.57995	18.28668	41.47152	40.80801
Cs3g23320	0.743368	0.749289	0.34104	0.701082	0.054986	0.261384	0.070788	0.13522	0.207215	0.270112
Cs3g23360	16.07205	10.48671	1.029556	1.078342	0.166122	0.639258	0.308558	4.835571	0.096437	0.160929
Cs3g23380	10.22042	9.334375	9.043109	10.03	7.562248	17.0961	18.6754	64.19716	27.54866	72.81543
Cs3g23440	0.341835	0.567501	1.074016	1.421138	9.33504	9.804236	15.37921	18.27096	86.75589	75.4039
Cs3g23480	80.46183	54.45529	55.54091	45.61213	56.70346	31.29491	37.73166	28.07046	44.56365	34.9421
Cs3g23510	4.856312	2.024198	11.43248	8.05222	19.23042	18.59245	7.280041	4.721778	5.397488	10.56144
Cs3g23530	10.62875	11.32618	5.759723	2.49702	1.166524	0.9254	0.287235	1.118493	0.112675	0.068839
Cs3g23540	8.218104	9.479598	5.637843	6.431995	3.042098	3.775341	1.160587	4.733612	0.089058	0.168053
Cs3g23560	4.663526	4.679952	3.584408	4.44568	7.849948	10.38384	4.891011	11.53749	9.31324	14.09018

Cs3g23620	12.59861	11.79137	17.06385	14.94837	21.97317	20.95823	28.53768	31.15972	27.17678	20.43496
Cs3g23680	8.903586	5.790773	5.765868	6.375218	2.220114	7.253507	3.18772	10.62708	15.16645	28.90761
Cs3g23730	11.01143	11.68249	9.821587	9.585125	5.695344	6.771202	3.095356	7.850516	6.822777	7.860108
Cs3g23740	16.09869	11.77473	10.49202	12.56309	6.9283	16.50884	6.528104	19.93461	5.026692	5.101444
Cs3g23850	27.13486	19.44424	38.91783	39.86707	26.96718	66.94426	27.22934	20.0706	106.5839	90.80241
Cs3g23890	0.456048	1.329788	0.599109	0.690349	1.69425	1.179261	1.498996	1.829989	0.32887	0.289449
Cs3g23920	9.254739	11.95564	26.44596	27.9508	10.42218	15.08412	23.74585	26.11921	7.160847	18.98538
Cs3g23950	4.461328	10.31255	6.883122	14.09776	0.30861	0.904595	1.430451	21.2	0.406149	5.617285
Cs3g23960	9.904147	10.60273	11.77484	11.20779	7.98061	9.09137	5.72023	12.47683	10.18239	9.292145
Cs3g24120	8.082784	2.347408	3.693835	3.013663	7.157328	6.491272	8.23722	4.615774	9.523046	1.096475
Cs3g24190	9.390631	7.73705	9.732979	7.43605	9.079339	5.74092	8.299905	8.823492	1.401069	4.34376
Cs3g24200	0.742466	0.432682	0.894697	0.304037	0.200576	0.073026	0.565586	0.340614	0.143439	1.919672
Cs3g24210	13.42448	3.729891	2.435784	2.023842	2.258436	1.444944	2.557537	1.690009	2.173555	0.636679
Cs3g24220	10.81273	10.18073	11.09921	8.932046	9.928346	9.700256	6.731099	4.678915	4.935078	1.806712
Cs3g24230	13.08037	11.50118	26.87925	27.52868	25.3786	47.1638	85.77131	45.97343	657.328	451.3897
Cs3g24260	0.708088	0.915887	1.983627	2.719844	44.63651	49.2208	44.44287	33.49562	8.419937	6.495108
Cs3g24280	0.411081	0.240837	0.127557	0.193258	0.333888	0.594788	0.434043	0.096897	0.944029	4.146474
Cs3g24285	56.96415	38.3423	25.98227	40.94919	30.04289	55.00135	17.33544	40.30007	68.1208	180.6674
Cs3g24360	7.77888	6.188125	5.969886	6.644125	3.639531	4.661418	0.823539	2.035297	4.16841	2.49158
Cs3g24380	37.06336	26.89164	10.75271	13.0179	5.53777	16.18579	16.63275	20.15959	30.26206	19.24751
Cs3g24460	9.261301	10.45755	8.117794	8.988394	6.554612	9.587238	3.122821	8.955931	4.123811	4.833295
Cs3g24510	3.079381	2.912838	3.045484	3.076807	1.861391	2.44563	1.39266	2.987276	3.529601	3.718223
Cs3g24530	3.916728	8.642663	1.632877	2.903921	0.387134	0.607195	0.161888	0.388199	0.153411	0.065024
Cs3g24580	5.107251	5.347928	5.39759	4.407984	3.979614	3.211217	1.800486	2.828417	1.502304	0.702145
Cs3g24680	18.20597	16.17321	23.60166	30.94301	4.893249	6.245359	4.363188	5.107431	2.896715	2.542128
Cs3g24750	23.65243	49.89673	6.950137	14.62347	8.210634	5.209362	8.123217	15.73819	4.225038	4.465758
Cs3g24760	1.202136	2.082552	2.123211	2.741655	1.58465	1.865133	1.35772	4.099726	0.857664	2.754688
Cs3g24835	1.756473	1.646849	1.611747	1.228807	2.343946	1.497293	0.91025	2.378488	1.789352	0.645015
Cs3g24860	19.24702	16.53336	14.25648	12.58843	8.014572	11.38075	3.087357	2.934129	5.18158	2.904621
Cs3g24970	0.315554	0.235079	0.62109	0.597327	1.001239	0.476185	1.182592	0.358952	1.023515	1.714844
Cs3g25000	66.04809	79.54884	42.22559	38.94256	23.19461	28.29283	6.59624	24.44501	4.072617	10.16705
Cs3g25100	46.39	61.77635	21.01748	33.88259	17.15252	24.60578	39.90494	46.26304	12.98027	26.54192

Cs3g25110	26.5356	26.48322	30.69087	28.14149	43.63025	35.40948	56.89623	61.57006	32.66854	32.8283
Cs3g25130	2.202667	0.964542	0.657118	0.455128	3.857127	3.04266	4.003426	1.123391	0.74565	0.616302
Cs3g25140	0.040911	0.094556	0.0194	0.104143	0.069359	0.085531	1.171027	0.116332	34.38272	29.40807
Cs3g25165	2.081339	1.807005	1.705274	1.791774	0.788116	0.855567	0.312183	1.689632	1.075299	1.88947
Cs3g25370	2.954975	2.220577	3.967716	4.12937	10.76137	15.17396	33.40949	20.02744	95.48397	77.80878
Cs3g25380	0.04013	0.031194	0.289684	0.226904	0.330321	1.243978	2.84906	2.351979	5.548872	8.463362
Cs3g25410	33.26248	23.31283	7.080021	8.559172	8.819923	9.85935	0.688565	9.759025	18.19261	17.61094
Cs3g25420	26.28228	17.30227	22.46939	29.90574	69.46754	59.52464	74.04965	59.78158	300.1086	334.3111
Cs3g25470	1.224573	1.463306	4.683937	4.946527	2.879325	4.310489	3.991648	4.148806	0.937363	1.8421
Cs3g25540	0.443294	0.499125	0.761297	0.762271	0.559489	1.635119	1.216444	1.873859	10.78334	7.493174
Cs3g25560	0.35997	0.157639	0.474526	0.492114	0.850868	0.962147	1.082002	1.292287	7.108512	4.009926
Cs3g25630	1.058249	0.914524	1.575607	1.598236	2.637428	3.326376	1.539218	1.991819	3.812709	5.754396
Cs3g25645	2.423639	3.121112	2.919234	3.274718	3.141947	3.682214	1.304305	3.518877	1.2074	1.779119
Cs3g25660	0.438728	0.340405	1.021298	0.738919	0.934125	1.006667	1.748323	0.671753	1.098255	0.940109
Cs3g25780	84.77097	85.62593	166.1389	137.7882	198.4432	120.9312	78.85307	187.3155	22.85503	23.79723
Cs3g25820	27.91079	26.73342	52.26833	49.41707	43.13773	48.06324	25.06865	27.03644	13.70968	17.81789
Cs3g25830	30.36664	28.91353	49.08376	35.71088	60.07033	28.34259	41.601	39.48126	15.79004	20.65843
Cs3g25890	21.9638	20.41771	9.742358	10.68053	4.121213	6.564538	6.349116	9.346713	6.142195	4.635817
Cs3g25900	0.738791	0.5562	1.328667	0.628404	0.706521	1.338498	1.969762	2.07382	0.041668	0.019953
Cs3g25940	12.1137	7.483579	5.614124	4.468944	6.671805	7.125946	2.725487	2.452798	0.956731	0.27776
Cs3g25980	9.5748	24.40137	2.663278	6.265173	2.082412	1.299485	0.619886	2.820145	0.22906	0.176487
Cs3g26060	9.308838	10.01067	14.35273	13.77392	17.05327	20.59276	9.173922	21.99965	25.68652	25.63552
Cs3g26100	80.56158	101.0163	75.53171	63.57194	89.5362	117.924	46.48995	298.698	10.19276	8.988053
Cs3g26110	29.14357	25.02103	30.36698	25.72863	30.95153	31.66588	18.36807	53.05711	45.58485	43.26458
Cs3g26160	11.53455	13.0288	7.179707	5.561642	5.681655	4.789532	2.508618	3.40252	1.303448	0.805
Cs3g26210	5.993252	4.318371	13.87429	17.02219	8.909942	27.13961	10.41975	10.37621	56.77143	58.71567
Cs3g26220	417.214	243.6277	44.50309	45.65708	15.3658	37.95896	21.39503	37.54981	20.63695	54.46808
Cs3g26240	2.94637	2.228407	4.590459	6.158482	1.97652	5.616031	9.012421	5.93082	5.922275	6.998699
Cs3g26470	22.61422	29.65787	18.61351	19.67375	4.706241	11.47574	2.58843	13.37441	0.842722	3.013878
Cs3g26480	72.0879	104.0578	60.40969	74.7748	40.75491	51.54747	55.26001	119.2778	22.43534	54.37197
Cs3g26570	2.33008	5.420931	1.17738	2.073469	0.471062	0.337223	0.410543	1.516219	0.271809	0.222839
Cs3g26640	125.801	120.4126	165.9472	121.3416	194.725	93.03653	222.0303	181.4132	110.9113	144.6008

Cs3g26680	6.052666	5.224374	9.951388	7.496817	6.128517	4.048327	6.090116	4.676683	0.676577	1.411115
Cs3g26690	63.49123	104.4942	37.64427	58.01983	17.07034	16.04832	6.629082	17.68994	1.095212	0.884627
Cs3g26730	204.1698	363.3808	35.04985	81.08644	7.084816	8.324997	4.21109	10.82659	4.433378	2.777806
Cs3g26750	14.31647	19.10178	17.89792	17.76455	11.32901	12.52226	7.034771	14.12313	3.056937	7.866131
Cs3g26760	34.94613	37.13098	24.49698	33.18304	5.260417	6.773932	1.972955	8.731737	0.293172	0.197366
Cs3g26900	18.73573	12.60945	3.782934	5.753642	0.587385	1.61088	0.593099	0.756247	0.321797	0.394006
Cs3g27020	0.140521	0.674137	0.612959	0.286327	0.242315	0.326926	0.102037	0.492449	6.809747	22.76067
Cs3g27090	29.39859	76.52804	2.682113	3.917477	2.241681	2.647635	1.763669	3.671595	2.311607	6.495823
Cs3g27160	19.37639	18.8815	27.84148	28.94753	32.01323	54.78254	16.64829	41.13559	42.98677	33.36203
Cs3g27170	0.521403	0.599314	3.566824	2.411464	10.67119	14.90516	7.306336	19.05282	12.27657	7.774628
Cs3g27240	27.97255	50.03075	14.18868	17.89476	11.62849	10.41786	7.487283	22.09523	10.16599	14.35161
Cs3g27250	3.698535	6.15768	3.223513	4.083533	3.425266	4.191396	1.803153	4.116849	1.041717	1.504765
Cs3g27280	193.0349	199.8384	171.081	169.8792	126.6557	175.571	60.41661	73.30893	33.35012	44.63368
Cs3g27300	5.149589	6.452814	5.024408	4.811534	3.519394	4.097169	2.176393	2.57399	1.230728	0.746389
Cs3g27320	13.82301	8.223279	5.24508	7.205196	3.637724	11.97248	8.368851	7.722086	23.89998	4.556572
Cs3g27330	16.04813	11.45989	11.07931	12.14647	8.08384	20.40096	8.357573	21.70358	59.65937	78.10108
Cs3g27430	14.09528	13.27006	19.02465	16.72099	15.25907	17.9801	9.599891	21.51913	8.634418	14.27371
Cs3g27520	60.6335	128.6641	17.36088	53.39734	4.755121	10.42051	10.65185	19.70042	2.635949	2.988013
Cs3g27530	41.1486	37.01694	39.11452	52.34698	37.77424	42.92483	32.17093	69.31812	47.26489	58.73686
Cs3g27550	1.148807	1.055751	1.358293	1.475306	2.188134	2.389059	2.96453	2.332673	1.276281	1.984496
Cs3g27570	4.623765	4.824305	4.759079	5.691076	4.122071	5.830573	3.257707	9.740308	3.537812	5.812989
Cs3g27620	5.116818	9.786188	2.937119	4.845328	1.985431	1.603262	3.040641	2.883476	1.102063	1.12902
Cs3g27650	0.284037	0.549583	1.376565	2.242854	0.223534	1.073825	0.398114	0.958479	0.071154	0.203616
Cs3g27660	4.036586	3.86338	9.42324	12.83442	5.558445	7.619839	1.919054	4.828034	0.202527	0.753964
Cs3g27760	25.50538	30.94908	44.85213	39.88095	83.31865	69.99053	90.43231	53.78452	71.14805	52.94466
Cs3g27810	6.459795	5.933433	6.604956	13.16591	1.170839	15.12382	6.553817	18.69696	90.87397	234.465
Cs3g27870	18.70691	16.52918	17.94894	15.76275	21.00232	16.67829	13.85815	10.28263	0.752155	1.630961
Cs4g01090	4.689891	5.00273	3.273086	2.659783	1.222043	1.205468	0.322914	0.888471	0.532218	0.61626
Cs4g01120	10.74201	10.69004	6.861935	5.736087	2.388845	2.832234	0.73608	1.886371	0.794403	1.709114
Cs4g01140	1.404906	1.518071	1.498859	1.025397	3.009434	2.045046	2.308786	1.151517	0.623289	0.390731
Cs4g01190	3.639525	7.044186	3.72492	5.133907	3.221352	2.03091	0.910485	2.861624	0.178656	0.068811
Cs4g01210	16.96487	14.08619	12.5672	11.75456	6.80041	7.412321	4.986981	4.355841	9.085935	8.4546

Cs4g01270	10.2671	13.2044	13.12544	12.57441	37.6873	13.78066	32.93384	39.49488	12.08095	14.02908
Cs4g01360	1.562516	1.523295	2.008276	2.247924	1.649264	2.401001	0.885726	2.134413	1.0248	0.941552
Cs4g01410	3.052069	2.983998	6.859145	4.981807	14.30009	13.4961	10.77475	18.172	22.30518	19.69787
Cs4g01430	16.25998	9.489156	12.35512	11.53008	12.89676	13.58606	12.99181	14.29127	125.6021	48.2497
Cs4g01490	31.73421	84.33248	2.929961	9.043831	3.125645	2.534667	1.682273	1.044928	0.515065	0.482525
Cs4g01600	268.3863	430.7072	219.3891	298.6728	139.7571	85.51016	93.97243	182.8762	30.46725	58.94161
Cs4g01630	27.96133	25.49575	31.11518	45.39132	9.52194	23.40732	24.32933	55.97618	64.85265	189.9214
Cs4g01650	26.69108	31.3561	29.79365	25.04068	14.83408	13.50832	8.242169	8.049652	5.973188	6.203801
Cs4g01780	13.30602	15.67711	15.61953	15.77529	15.06565	16.62401	9.040084	20.90707	10.33796	11.09969
Cs4g01870	33.70059	43.59605	51.52318	61.38945	18.28181	31.98353	16.19828	37.58614	27.60671	51.22289
Cs4g01920	2.684793	3.316449	2.496799	3.107152	3.162941	2.696878	5.558564	5.920692	11.47768	9.382261
Cs4g02040	13.89314	14.56693	21.64322	21.03378	19.27662	25.16086	13.36539	29.71519	14.56088	21.00465
Cs4g02090	3.75643	3.147169	2.307049	2.455303	1.517456	1.327554	1.027849	1.271621	0.663438	0.301477
Cs4g02160	18.03819	20.94583	13.93025	17.44984	11.22006	10.9267	7.18431	25.42454	6.595681	13.80374
Cs4g02180	3.17564	5.108599	0.844737	2.024377	0.186331	0.064014	0.047445	0.2051	0	0
Cs4g02190	7.234429	13.33089	2.454795	3.370359	1.719511	0.940008	0.69736	1.500111	0.243806	0.129356
Cs4g02210	2.590637	2.146783	2.434277	1.990558	1.584935	1.340157	0.494871	1.149609	0.085479	0.266303
Cs4g02290	8.65015	8.096828	6.379495	6.027357	6.490668	8.545822	5.289078	13.59926	3.553074	2.237025
Cs4g02410	16.69099	15.47607	49.07598	34.27216	114.6254	60.04945	75.57747	65.48907	26.33225	40.75094
Cs4g02450	186.0366	227.9171	123.3227	137.1553	31.4966	76.03563	87.13259	197.7804	106.839	177.6971
Cs4g02500	2.478209	3.289606	3.915721	4.248167	5.465198	6.946857	3.741698	8.529954	7.979355	8.02759
Cs4g02530	3.712754	6.522634	4.743896	5.13967	3.483198	3.429668	5.256927	12.4	46.12103	45.43634
Cs4g02570	0.048819	0.040584	0.004728	0.049073	0	0.035045	0.235717	1.411268	0.093866	4.726199
Cs4g02580	24.4665	93.80262	14.29209	77.71709	43.12248	238.4916	89.57021	429.7763	95.56272	385.3329
Cs4g02590	2.680437	2.247305	6.883591	4.601114	13.25761	32.03335	11.55751	43.09896	9.328989	14.72302
Cs4g02610	5.818089	5.403172	5.919718	5.495024	6.967307	6.710652	4.403847	9.318483	2.586929	2.959501
Cs4g02640	14.33344	15.48337	8.270391	10.44497	5.850723	7.071495	5.485724	8.419329	7.333663	5.774918
Cs4g02670	4.382006	4.415073	12.62994	18.97642	13.91862	9.31337	10.7671	47.23717	6.081928	16.76658
Cs4g02680	0	0.281966	0.84798	0.847746	0.208687	0.554264	0.376321	2.807654	0.096024	0.878826
Cs4g02690	0.220375	0.161865	1.083993	1.86773	0.295442	0.199653	0.069823	2.575053	0	0.141266
Cs4g02710	5.856738	5.188846	7.537768	6.218417	23.07663	17.08663	22.84347	14.70695	22.67899	20.48471
Cs4g02720	6.792276	9.342362	5.860059	7.541527	6.219835	5.621311	3.291872	9.919954	3.235886	7.609051

Cs4g02730	54.07833	54.90892	19.33734	22.6549	12.79951	15.01672	16.44887	28.29786	61.28576	98.84493
Cs4g02820	10.4847	5.772081	6.355151	5.315766	9.871489	10.39328	3.404648	5.631116	1.160325	1.205103
Cs4g02910	30.93797	31.39997	2.127037	8.0614	18.81749	27.21047	33.08103	44.59631	85.66351	62.24204
Cs4g02940	24.64615	39.64377	6.018508	11.65081	1.009209	0.921556	0.458622	1.857408	0.114607	0.200944
Cs4g02970	18.02109	17.10211	7.977292	8.301585	4.256978	2.086288	12.56517	14.58839	1.147221	3.688656
Cs4g02980	1.195589	0.974623	1.216958	1.135787	0.326034	0.726989	0.482566	3.141149	0.049791	0.065737
Cs4g03050	1.734102	4.721915	2.000772	3.550455	0.295978	0.83683	6.647214	40.36915	1.639683	23.61032
Cs4g03060	1.015311	2.26527	0.720235	1.212742	0	0.472903	2.425691	26.66194	0.535306	6.675088
Cs4g03130	2.083687	5.91254	2.479785	4.595902	0.434621	0.84477	8.617867	57.6275	2.524811	39.5477
Cs4g03140	2.121864	5.570378	1.335794	2.238172	0.024447	0.608702	4.463507	50.43292	1.817993	25.64123
Cs4g03145	0.297646	0.390585	0.063467	0.108221	0.011434	0.146582	0	4.499589	0.188887	1.644655
Cs4g03200	22.35049	41.32298	14.28225	18.91887	5.285016	7.663904	15.00353	108.65	12.66354	48.77477
Cs4g03210	0.251678	0.46048	0.007631	0.187277	0.017286	0.07753	0.133222	1.734393	0	0.164854
Cs4g03220	0.648962	2.061458	0.350648	0.744165	0.050591	0.405318	0.68438	11.83345	0.258189	2.595782
Cs4g03320	7.611148	8.787652	6.444536	7.120811	4.256169	4.19134	2.055315	4.501084	0.418677	0.079212
Cs4g03330	26.50331	46.39359	15.71236	15.85858	22.29145	12.69008	74.77704	65.42868	17.14418	20.8798
Cs4g03370	0.127362	0.405057	0.402754	0.451667	0.1911	0.361526	0.335889	2.695337	0.980328	2.49299
Cs4g03470	5.240346	5.604368	6.626815	7.0518	1.909096	2.567451	5.096646	3.257141	1.28334	2.443069
Cs4g03490	7.677476	5.809079	6.789848	5.079414	2.983071	2.196287	1.987205	2.422559	0.086761	0.054963
Cs4g03550	8.705014	6.930943	7.832886	6.714351	6.98379	6.704664	2.645138	4.513151	8.250756	3.488943
Cs4g03560	34.24299	27.5996	18.07328	19.6659	15.48844	16.31593	11.53708	10.41249	14.91649	8.715279
Cs4g03580	6.894055	7.130851	21.76568	13.3638	25.32474	31.16481	34.66923	82.411	51.03663	59.55054
Cs4g03700	12.21999	10.35714	10.83654	8.051441	15.46359	40.74055	52.21614	25.43658	335.0187	105.5821
Cs4g03720	14.27503	24.1055	6.203705	11.81241	5.112284	4.744129	1.723321	5.935625	0.586354	0.676568
Cs4g03730	1.333905	1.624582	2.740824	1.870396	8.185566	11.97254	9.9508	16.19935	9.074623	4.767455
Cs4g03740	0.283458	0.256677	0.190392	0.202732	1.191486	1.309791	1.664503	4.181727	26.73867	37.91103
Cs4g03830	9.184958	9.007526	10.9891	8.513044	6.425461	4.835272	3.792379	2.851419	0.471328	0.522386
Cs4g03940	1.018744	2.633415	1.976172	3.22018	1.234564	1.594931	2.622484	13.27539	5.729755	7.206142
Cs4g04000	11.95384	6.234544	11.90266	16.92363	18.9407	36.31513	8.878783	5.232985	46.76195	13.32036
Cs4g04150	2.435521	3.961137	1.410413	2.027391	2.507983	2.342622	1.05518	1.216004	0.17342	0.193959
Cs4g04170	4.178975	6.056493	3.461667	3.792717	2.776579	3.532846	1.599639	5.085162	0.596989	1.409146
Cs4g04210	28.91699	25.58399	53.20672	58.07248	45.0927	64.91361	18.53802	8.461009	44.70579	28.63532

Cs4g04270	18.91233	18.4358	16.95742	16.10908	4.974204	4.348295	1.225824	9.223097	1.278279	5.38161
Cs4g04300	153.4614	53.69254	52.30425	48.11917	63.4355	79.68213	58.56396	31.68421	333.4465	188.7182
Cs4g04310	4.206337	4.573129	9.436866	8.134254	3.487913	8.248433	0.903083	7.287007	3.585097	5.560612
Cs4g04350	16.31521	19.7169	13.01548	14.79845	5.706897	6.50428	5.223834	14.87494	5.46583	11.27579
Cs4g04420	10.61334	17.56853	43.57845	67.24561	41.18194	42.42895	44.5287	127.3699	19.85564	70.01351
Cs4g04530	0.663212	0.610171	0.502407	0.809059	2.036613	6.869073	2.787203	2.746231	14.34703	6.960687
Cs4g04550	356.9815	479.862	447.2462	510.7461	202.411	202.1907	101.2619	145.0137	43.58427	34.5149
Cs4g04730	10.52193	18.69274	6.446545	7.83085	4.112972	9.002015	1.705112	6.449184	1.048942	6.217807
Cs4g04740	2.069811	4.053768	1.016032	1.539444	0.802662	2.041456	0.414402	1.676364	0.139376	1.631722
Cs4g04750	1.465399	1.64788	1.543314	1.73295	1.116694	2.734814	0.220777	0.437712	0.539161	1.14828
Cs4g04770	14.11145	17.73793	21.02625	21.07804	24.07807	28.20343	14.20697	31.83252	16.82169	23.54529
Cs4g04790	0.831632	0.514704	1.128125	1.092682	3.075326	1.632374	1.497286	2.384783	0	0.012641
Cs4g04930	12.20167	7.853365	10.86633	11.31851	18.10442	17.1478	20.88155	16.96238	27.96663	22.24722
Cs4g05160	8.376803	6.883424	1.902295	1.874761	0.624584	1.075938	0.228373	0.842724	0.1716	0.223018
Cs4g05190	9.600617	14.73611	18.58225	28.19415	29.13648	49.28474	28.65469	39.96379	19.07367	5.076716
Cs4g05200	2.423531	1.779576	13.6632	21.12648	5.587126	16.64598	13.58923	5.240653	10.89019	20.13022
Cs4g05210	36.00914	37.52148	25.43821	24.70728	15.48354	19.91892	19.89624	28.22476	28.2686	69.22452
Cs4g05240	7.081108	27.20004	11.21552	19.85082	4.462171	19.8179	11.76121	9.051479	13.00103	7.18774
Cs4g05250	1.375199	2.315698	1.720401	2.617712	0.914919	8.430693	0.875777	2.664756	0.049144	0.076163
Cs4g05260	5.885129	6.301918	8.142518	7.865184	9.012403	9.002602	4.262023	10.01008	6.49542	8.00958
Cs4g05300	38.15602	45.41838	60.40613	67.55497	27.22647	31.40603	54.92356	52.24753	62.40802	100.2265
Cs4g05490	198.7868	176.8972	124.3626	134.5092	98.96087	74.45305	117.2786	66.39908	310.4214	480.6498
Cs4g05500	21.32984	17.9045	16.17361	13.64019	13.62438	12.51587	28.93915	11.10386	16.64874	13.23264
Cs4g05510	63.61376	78.4467	36.81263	38.90997	10.32985	7.479949	6.773344	17.01532	4.266014	6.275783
Cs4g05580	104.8715	43.33479	109.2536	120.5116	123.2769	118.0216	136.3991	101.7638	119.0391	103.709
Cs4g05660	8.966631	4.759488	7.537284	10.68704	6.261238	13.79432	20.14213	11.09259	84.435	68.29022
Cs4g05760	14.48405	27.77573	116.2893	126.5732	32.80125	97.14497	10.37561	43.60825	4.69973	17.8993
Cs4g05890	2.145325	2.83529	9.190223	7.138413	2.265134	3.432645	3.571809	4.934312	0.339789	0.873703
Cs4g05900	19.43416	9.813096	12.87353	12.06717	11.77467	19.35107	1.083383	2.003797	0.145714	0.042921
Cs4g05980	8.30104	6.465181	15.83696	9.88298	4.439666	8.604011	1.253062	0.834659	0.065581	0.010498
Cs4g06010	3.264901	3.36026	5.898465	4.816868	2.309174	2.494135	1.411358	2.208704	0.60087	0.884425
Cs4g06030	8.356854	35.83041	10.43586	18.54984	5.316028	1.664004	1.049261	3.875857	1.028555	1.429723

Cs4g06060	1.28802	1.522729	0.626997	1.034411	0.201084	0.275611	0.183538	0.252181	0.472835	2.176814
Cs4g06090	3.069618	4.992667	3.258677	1.992496	10.64892	1.396874	4.499938	7.378432	0.449636	0.284634
Cs4g06140	23.89393	25.79819	22.36987	23.73223	9.43136	10.09229	3.476407	11.1029	1.583505	1.709524
Cs4g06170	27.07189	24.26428	8.452794	11.67147	3.169861	4.294835	2.617746	7.008885	0.82105	2.22968
Cs4g06180	18.59808	14.1033	4.772145	5.673288	1.745811	3.72753	1.230734	5.236111	1.726816	1.049451
Cs4g06230	23.18455	30.40103	16.78691	22.61585	3.935052	4.946991	3.784317	5.285875	1.655964	2.620773
Cs4g06260	57.9909	55.16658	28.00876	27.07222	20.66129	24.54043	12.26224	16.54448	15.27862	19.68908
Cs4g06290	16.08637	8.099762	21.66794	25.94108	13.26152	47.11613	14.70968	11.5166	128.7149	85.27765
Cs4g06310	9.892344	8.372396	6.434739	7.404606	3.15842	2.893102	7.459715	6.951249	0.258686	0.300573
Cs4g06320	0	0	0.015277	0	0.140106	0.256466	0.886562	1.739407	0.115182	0.142031
Cs4g06340	4.093198	4.129849	11.28987	12.27546	6.6421	8.564804	8.501681	20.13149	29.28395	74.68082
Cs4g06380	13.60209	13.9423	19.43997	14.4083	13.18223	9.106037	4.06582	6.585497	1.53508	3.923674
Cs4g06410	10.35038	9.182592	18.30668	18.19507	4.013216	4.875791	12.58838	27.83884	5.823889	10.24284
Cs4g06430	50.66241	43.74548	43.61996	39.66269	66.36342	62.41768	41.41458	66.62333	3.688734	13.38028
Cs4g06570	6.308656	5.216149	3.993329	4.028142	2.985336	3.215434	1.211818	3.381801	3.365213	2.344964
Cs4g06585	5.684659	7.254623	6.622945	7.225958	6.207728	9.216192	2.445862	9.468512	7.577723	9.615107
Cs4g06590	3.168814	2.851842	3.432655	2.846365	5.772585	6.08238	2.001895	5.804666	3.388423	2.562627
Cs4g06630	51.87133	30.86152	19.81836	24.22928	7.910343	9.590742	11.90226	17.05368	0.651433	0.447164
Cs4g06650	101.3225	52.1317	53.11362	48.03674	48.22958	77.86985	51.13824	115.304	27.04593	28.46919
Cs4g06670	37.82125	15.93445	12.47659	14.20859	8.746834	34.03851	3.993898	5.359808	2.389223	0.640703
Cs4g06690	8.823822	3.698671	6.566148	6.370408	9.718792	26.96597	4.919982	7.77272	3.11486	1.156177
Cs4g06710	216.1176	182.9389	26.5045	26.4679	5.364157	8.385677	3.140994	6.985599	1.262465	0.888226
Cs4g06720	1.564205	1.127949	1.206272	1.128933	1.076871	1.903274	1.260183	1.181852	3.280964	0.742851
Cs4g06760	4.48561	4.253539	12.43018	16.30805	5.429412	9.645136	7.752273	8.767598	2.852982	11.10523
Cs4g06880	4.409482	5.835347	4.851595	5.054104	4.469535	4.033258	8.837483	10.08813	8.170532	8.281838
Cs4g06910	1.282876	1.214493	1.471369	1.801379	1.606962	1.27347	0.670146	1.941935	0.260141	0.924295
Cs4g06950	0.07704	0.047555	0.389651	0.527193	1.468586	2.402112	1.064933	1.832003	1.657396	2.516987
Cs4g06970	0.372119	0.615648	3.21264	3.425133	4.274608	44.63856	8.891582	16.74557	4.6002	3.15617
Cs4g07040	116.3541	230.4879	191.4546	156.6459	17.746	19.55132	38.42327	148.4221	10.42055	24.384
Cs4g07060	5.636559	6.881801	10.26388	10.19124	8.500539	3.831823	0.094939	4.542221	5.307128	5.107646
Cs4g07080	0	0	0.01567	0.004075	0	0.016975	0.063408	0.022582	0.360609	9.062827
Cs4g07180	5.090538	13.11078	7.082683	18.66692	8.273829	20.56304	10.56046	28.52272	5.318434	19.51633

Cs4g07210	4.767138	8.09358	2.679541	3.690542	1.808911	2.043848	0.923099	1.073157	1.609867	1.691589
Cs4g07270	61.70691	68.83846	46.10859	70.26466	19.12898	21.07288	17.79732	15.84209	3.139778	3.483164
Cs4g07310	2.661553	3.670822	1.609566	1.663827	0.163831	0.212322	0.404649	2.403459	0.036722	0.107871
Cs4g07340	0.893271	0.605402	0.338839	0.602662	0.167635	0.962819	0.308988	2.171249	0.63493	0.721543
Cs4g07380	0.042601	0.187218	0.796585	1.694211	0.191218	1.361772	0.035162	0.164515	0.358778	0.044249
Cs4g07400	1.173653	1.413524	1.155647	1.353056	0.241282	0.325034	1.116919	3.38921	0.485056	1.334688
Cs4g07650	178.0674	306.6965	62.76844	97.79154	28.90519	29.99177	8.676928	22.91668	2.904959	4.776415
Cs4g07680	5.209345	5.761396	4.430468	5.041852	2.336525	4.01903	2.483963	12.02815	8.722673	17.46024
Cs4g07690	9.695213	10.96965	7.098136	16.84758	6.458503	9.458938	7.779011	12.54899	9.4825	36.80548
Cs4g07720	4.455027	8.389264	3.895873	3.545664	5.292927	2.450945	5.975035	6.918516	2.331697	3.463632
Cs4g07730	4.092533	1.29445	3.550982	1.983316	1.99774	1.902842	1.539316	1.078184	5.745846	3.186927
Cs4g07780	0.31761	0.377857	0.48643	0.409743	1.526625	0.896315	1.45096	1.279098	0.84226	0.855113
Cs4g07790	20.12112	20.41518	11.05626	10.48245	14.41793	4.919831	25.04025	15.95555	11.95155	9.956618
Cs4g07840	5.213396	2.480652	14.29716	8.462676	42.24408	41.64672	39.53584	12.44019	16.34243	13.76869
Cs4g07980	8.451995	8.747074	11.15157	10.85883	2.88003	6.569549	2.081032	5.17445	9.372183	8.529046
Cs4g08090	34.77536	30.85896	31.11925	24.98157	20.17735	17.35133	7.159938	9.346585	0.80934	0.478294
Cs4g08100	9.894988	8.037633	7.85985	7.385042	1.898437	3.098132	2.516773	4.859584	3.892427	7.038616
Cs4g08210	1.938725	3.000168	2.246778	2.821525	1.811686	1.907278	0.880994	2.864864	0.616227	0.740702
Cs4g08260	21.44782	21.96322	37.16292	35.69327	33.90348	34.74001	33.20103	71.51037	24.52149	29.72723
Cs4g08320	1.948555	2.602414	4.105104	3.000767	4.080063	7.84607	2.513543	6.207778	2.071289	3.291376
Cs4g08480	0.434441	0.466491	0.87283	0.736654	1.223425	1.174037	0.939024	1.633012	0.898904	0.610839
Cs4g08500	5.267989	7.407085	5.236144	6.304917	4.971326	4.855772	2.246575	7.38551	1.211314	3.365242
Cs4g08510	7.838941	10.25944	3.235654	3.695114	1.850735	1.517965	2.586475	2.827858	0.898259	0.47261
Cs4g08560	2.673748	3.231169	5.06431	4.654348	2.632193	3.130583	3.478165	6.301847	0.467131	2.007939
Cs4g08575	1.81064	2.015271	2.137636	1.924386	1.908766	2.458813	0.848773	2.633466	0.353923	0.994179
Cs4g08750	33.03398	23.84729	17.11108	20.07007	4.614216	14.08079	3.944337	13.54565	7.039972	20.37332
Cs4g08780	39.75247	31.24394	16.59976	17.85619	10.20344	9.432873	7.236633	6.855255	1.094641	1.321686
Cs4g08850	12.64123	11.02975	12.0002	9.652072	11.65281	9.952848	14.448	12.47732	0.256204	2.099769
Cs4g08990	18.12233	8.438266	13.5425	13.73585	18.88115	50.68067	16.23088	10.40993	67.90423	33.0307
Cs4g09010	0.07089	0.051154	0.353154	0.3185	0.504269	0.541694	0.860929	0.479	1.699097	0.425559
Cs4g09080	4.294742	2.327232	9.341064	6.848959	25.45861	18.77408	27.98108	14.48147	25.01849	14.57969
Cs4g09090	12.20055	5.318125	7.167754	7.9662	9.771189	27.43396	54.03571	18.86848	115.2099	73.0785

Cs4g09140	5.189739	5.50801	20.24538	17.58473	27.68194	36.33303	8.126538	8.420508	29.58587	17.28128
Cs4g09270	27.10154	20.38714	16.45329	19.508	29.77487	46.92311	49.5102	22.60574	44.59618	31.23174
Cs4g09320	7.563406	6.216403	7.078628	6.218157	7.202063	7.123341	8.802228	6.668901	8.941266	4.241127
Cs4g09370	130.655	95.86298	183.9903	151.4537	255.6846	180.7716	426.8665	177.3779	103.9787	80.13272
Cs4g09440	3.396814	4.726522	7.152635	8.955844	8.054089	9.97598	6.45533	6.658097	12.55215	26.04864
Cs4g09470	2.531464	4.087962	3.033158	4.679353	2.832497	3.251092	0.786436	3.825841	11.32963	18.85942
Cs4g09520	1.635569	1.929616	3.083971	3.777768	15.77415	14.68533	54.55646	38.36305	134.1552	66.95566
Cs4g09530	9.377437	11.74896	3.714928	3.75033	6.319592	3.25894	15.31827	10.23179	20.69845	8.335902
Cs4g09550	8600.633	7716.116	8497.31	8568.883	6188.95	6170.771	1784.015	5299.78	292.7574	950.6143
Cs4g09600	12.12015	11.50504	19.09649	16.63649	24.82046	20.68105	11.72569	23.63496	9.389825	8.201163
Cs4g09730	0.092133	0.22503	0.479382	0.414644	0.51973	1.377243	1.184563	1.945527	0.747243	1.402251
Cs4g09760	0.935069	0.879324	1.958872	1.629077	2.146168	1.440763	2.453007	4.021906	0.975657	1.9997
Cs4g09770	20.08212	28.72062	28.82959	37.53063	12.82463	19.99574	24.16523	50.41809	34.64568	67.29639
Cs4g09780	218.249	63.2194	46.13263	48.54934	86.66044	110.1202	114.6436	55.23521	785.1165	272.9743
Cs4g09810	18.88915	21.91041	19.36978	19.80565	23.9781	16.43996	7.934448	28.04515	42.75684	71.89772
Cs4g09830	1.896152	2.19459	1.686081	2.105417	1.627469	1.586674	0.459264	2.216524	1.458615	1.087368
Cs4g09870	324.3043	357.6336	711.2361	812.6687	506.2703	434.5329	1378.226	439.1248	80.45509	67.83109
Cs4g09900	2.94678	1.65435	1.333266	1.057555	0.129129	0.844763	0.032021	1.477609	0	0.11817
Cs4g09960	6.086043	7.588777	6.204598	6.576423	4.926612	5.084228	2.133044	6.388589	1.223387	2.240315
Cs4g10110	131.6446	80.76511	70.74029	52.68081	32.64302	40.82908	56.67788	97.58992	0.830301	2.036653
Cs4g10140	61.16262	50.24755	28.88097	38.02506	21.602	46.72458	13.1811	19.38036	43.88937	10.3176
Cs4g10160	1.401259	1.411132	5.320717	7.526874	6.208037	4.383416	11.20471	20.48705	34.88917	33.93453
Cs4g10220	2.643092	5.179185	1.192568	1.378101	0.631903	0.415235	0	0.53592	1.276356	0.526609
Cs4g10230	10.51467	11.03922	16.40845	14.86862	14.70722	19.05202	8.169249	18.19054	38.37616	20.8832
Cs4g10240	42.31849	38.22212	127.8991	116.9611	63.86289	139.5113	205.4319	35.90729	251.8422	103.9896
Cs4g10250	17.07669	15.05711	12.92074	26.85088	3.282463	8.335229	8.575594	15.37979	3.79813	4.770892
Cs4g10310	184.3576	326.5468	114.8267	169.1949	29.89391	47.76803	54.70232	190.481	231.4935	470.7218
Cs4g10320	21.38743	35.73787	10.90273	17.53432	3.313943	4.873157	4.900768	27.63571	22.2467	48.3191
Cs4g10370	0.303279	0.323199	0.617225	0.545892	0.344526	0.744897	0.041557	0.550404	0.244638	0.268087
Cs4g10410	37.17369	48.00728	44.32168	65.52928	12.05176	25.49027	14.48965	40.1507	38.81148	155.0722
Cs4g10460	2.682351	2.618213	3.126823	3.273119	0.762521	1.317287	0.739747	2.799622	0.568827	2.229899
Cs4g10500	17.11596	22.0117	20.05875	30.83696	6.502003	13.06939	7.266832	19.98295	19.95267	83.56171

Cs4g10520	3.494743	3.093291	4.011511	4.484146	1.027321	1.795589	0.943973	3.997072	0.878945	2.877055
Cs4g10560	2.388016	2.430314	2.502816	3.520021	2.169893	4.173339	1.174858	3.943374	2.229103	2.72863
Cs4g10570	3.816916	3.3851	4.353193	4.756035	3.091572	5.04741	1.515275	4.17634	2.668112	2.505384
Cs4g10580	7.225852	6.366521	7.337257	7.488799	4.146553	8.289486	1.190035	5.981314	3.451613	1.481876
Cs4g10590	8.101789	7.212497	8.216507	7.826703	7.42727	10.46719	3.451154	8.608984	7.153971	5.075825
Cs4g10630	0.323295	0.123291	0.362736	0.397347	0.231399	0.816204	0.017503	0.081634	0.05749	0.015586
Cs4g10710	2.964922	9.278932	1.595055	3.370915	14.59439	6.265875	12.62504	16.30498	1.746144	0.82487
Cs4g10930	2135.234	1415.89	760.1971	954.3372	456.1403	580.6062	356.1555	399.7377	1930.435	2242.891
Cs4g11010	4.502539	4.573127	5.816537	4.468374	2.003982	2.312766	0.79916	2.443835	0.458213	1.266369
Cs4g11040	0.879983	0.538094	1.759414	1.557574	1.396249	3.347571	0.22935	0.28928	0.427624	0.235065
Cs4g11160	125.0648	1.168505	29.6791	107.633	39.54743	80.88484	3.044868	8.24893	69.34439	68.9188
Cs4g11180	0.361352	0.380699	1.130218	0.798939	3.930298	1.202024	3.97853	2.785628	1.864256	0.570428
Cs4g11200	139.7221	112.9521	113.0944	123.022	35.80755	118.4991	17.13263	51.53958	97.93283	90.47664
Cs4g11330	1.308379	0.759793	1.913274	1.01203	16.43454	5.474192	4.444006	1.406512	0.144927	0.026068
Cs4g11350	4.834178	5.066646	5.917188	5.372364	3.344986	4.850684	2.110284	5.642016	3.979482	4.050597
Cs4g11380	3.848477	3.447179	5.322749	5.264185	2.904221	4.179452	4.30018	7.31777	3.528282	16.38835
Cs4g11440	0.735806	0.632624	1.805072	1.755556	2.88674	2.898139	4.702272	6.238792	21.51185	28.46116
Cs4g11510	0.223313	0.258613	0.281965	0.300388	0.387176	0.856499	0.461479	0.478208	0.648478	0.502036
Cs4g11530	1.9673	2.474025	5.356931	4.413635	2.930538	3.585534	1.093351	2.627381	0.900962	1.176821
Cs4g11560	2.79091	1.786504	7.515602	5.011773	7.89615	7.526314	8.054348	9.862451	12.37703	9.251789
Cs4g11600	3.811401	8.187489	3.537302	4.518439	6.354149	5.956048	6.10014	8.925488	4.34281	3.923762
Cs4g11610	19.49295	19.74142	22.63019	16.92654	9.771394	9.499574	2.750002	8.397015	0.205056	0.313337
Cs4g11660	1.701078	1.552715	1.939134	1.27591	1.093795	1.395068	0.265005	1.032028	0.175301	0.187874
Cs4g11690	17.56167	33.60344	42.80918	50.7853	6.561883	14.32366	11.45211	56.50397	4.158751	19.91973
Cs4g11700	19.02977	15.16242	13.45237	8.602942	6.319408	2.499198	0.9732	2.795715	0.57768	0.283904
Cs4g11730	1.069676	1.796382	1.792048	1.705564	0.722908	1.584335	0.170987	0.914464	0.035307	0.016623
Cs4g11740	2.716752	2.9972	2.752398	2.611971	2.247017	2.637686	1.159019	3.44786	1.270077	1.548025
Cs4g11860	46.7537	44.39067	70.2723	66.95936	96.40351	67.96255	107.3254	103.8331	78.57305	92.20585
Cs4g11980	6.358687	16.51773	3.560103	7.272929	3.147653	0.375927	0.465248	1.167544	0.036795	0
Cs4g12020	32.7278	37.68806	37.26555	28.94841	27.93929	16.08992	3.345019	4.078744	0.384579	1.01034
Cs4g12110	3.066523	8.063395	0.206615	0.834063	0	0.007472	0.009044	0.008504	0.164878	0.173272
Cs4g12130	191.7202	149.2243	160.6201	219.2512	22.44761	82.71971	144.9813	129.5298	594.1968	577.1142

Cs4g12160	27.29019	33.02884	16.95052	22.36106	9.570914	11.14997	10.10524	27.67531	10.38962	19.80426
Cs4g12170	22.09643	25.21648	20.13115	21.14171	7.574412	9.716411	5.944003	6.861862	13.38182	7.025242
Cs4g12180	1.055056	1.254628	1.292071	0.986247	1.847841	1.627674	4.160814	2.189099	0.756858	0.258553
Cs4g12220	2.368957	2.326856	2.747778	3.03405	0.656186	1.456545	0.128907	0.18412	0.330455	0.167828
Cs4g12280	16.91558	21.42199	12.9748	17.24336	2.15644	3.769702	1.432965	9.975314	1.249675	4.143542
Cs4g12290	8.583653	13.82795	3.22929	5.363305	0.137877	1.244053	0.04824	2.057279	1.135685	7.873384
Cs4g12320	2.752359	3.786692	1.920203	2.344362	1.813085	1.965556	1.96339	2.316176	0.465115	1.174864
Cs4g12360	28.9839	25.01063	37.7029	31.05858	41.78878	50.3258	64.25241	31.74722	41.30922	52.00319
Cs4g12450	0.059378	0.025235	0.063962	0.010503	0.431181	0.075778	2.913598	0.178309	0.743902	0.05112
Cs4g12460	1.283291	0.501534	2.098186	0.987526	16.92305	5.205924	3.947323	1.564423	0.151119	0.082369
Cs4g12480	0.229674	0.079297	0.15656	0.148597	1.066668	0.388453	0.32648	0.055297	0	0.007216
Cs4g12500	0.56081	0.384848	1.34285	0.728487	0.83921	1.576493	0.871703	1.551728	0.148939	0.436558
Cs4g12570	18.61066	22.47283	14.88328	17.60071	7.274978	15.39664	0.848483	4.40218	0	0.028943
Cs4g12600	5.774154	4.187894	5.936254	6.029305	8.722215	10.56265	13.32558	10.55538	32.63402	25.72585
Cs4g12660	9.211309	6.383188	21.15057	14.59102	30.24543	28.08063	67.02694	42.83204	31.27512	43.79278
Cs4g12710	0.093347	0.064911	0.148788	0.23164	0.604256	2.13114	1.75656	1.449146	4.956562	2.065087
Cs4g12750	12.41134	10.46511	24.74419	16.04436	34.90139	36.53927	32.61921	34.57417	52.92515	14.6035
Cs4g12760	0.070677	0.144766	0.420745	0.335089	1.420805	1.862979	6.583605	2.705125	63.41624	18.49273
Cs4g12870	1.348257	1.559224	0.884959	0.921665	2.373601	2.594808	0.581991	0.619853	1.140715	0.137923
Cs4g12920	3.137595	2.158382	5.111435	4.763801	7.423169	10.89551	1.549477	4.65901	8.050948	2.384798
Cs4g12930	2.166946	2.699745	1.597445	1.364132	1.218629	1.584016	0.603807	1.797653	1.259653	1.228813
Cs4g12990	0.323152	0.62349	0.275245	0.337467	0.054345	0.314101	0.244683	3.818676	0.246471	0.714399
Cs4g13080	11.69363	12.45661	17.08734	18.01533	17.6156	17.02307	9.542196	22.42276	12.98954	13.20101
Cs4g13120	0.135165	0.690604	0.006739	0.097777	0.006847	0.016963	0	0.011409	0.173201	3.547238
Cs4g13160	73.85014	69.47065	40.50521	30.46961	35.03068	34.57602	12.86499	11.37749	3.802089	2.315271
Cs4g13170	1.726338	2.32831	13.08989	9.053364	1.564469	6.072619	0.578394	3.634317	0.070032	0.224816
Cs4g13310	26.17645	27.53535	16.68618	17.72064	25.43436	15.40531	42.86158	28.34463	20.81676	7.531875
Cs4g13350	11.41297	6.692286	18.9312	12.54522	44.99299	30.47176	68.15637	70.8117	17.06159	14.6161
Cs4g13440	50.04164	57.68519	28.48069	40.87186	5.086792	11.6465	0.780978	7.210928	0.296243	0.420475
Cs4g13450	15.55797	19.11858	18.73428	18.76054	6.645189	14.3043	13.13423	12.46338	22.4259	32.89679
Cs4g13460	12.96023	11.72301	15.28304	14.28748	12.78334	17.5473	6.475722	15.58996	14.79406	16.68051
Cs4g13470	44.01429	39.05716	84.30097	78.29283	141.7186	126.2815	127.5757	136.4561	168.2966	157.4933

Cs4g13490	0.104542	0.127912	0.239032	0.080507	0.143872	0.119088	1.600859	0.913949	7.437521	3.317313
Cs4g13570	14.26815	14.94301	10.62666	9.344829	6.08363	5.941341	3.364601	4.351335	1.989199	1.735389
Cs4g13650	0.628671	0.790516	1.036758	0.736624	1.404042	2.13204	1.065135	2.93998	0.839109	0.767746
Cs4g13670	8.729351	8.744688	14.76651	13.10426	21.33482	17.14665	28.03285	30.39664	23.5078	23.34638
Cs4g13690	28.08321	46.494	9.871565	12.298	3.793417	3.8582	0.195446	0.94598	0.410142	0.465304
Cs4g13780	19.53269	21.75435	19.11019	28.74021	12.56655	11.30788	8.356373	2.7517	0.871868	0.46114
Cs4g13850	1.813006	1.560297	3.513376	6.244596	2.831571	7.062039	0.503687	0.676764	0.203943	0.148531
Cs4g13870	0.321788	0.269292	0.265887	0.425744	0.186572	0.109835	0.27414	2.313242	0.272479	4.116907
Cs4g13880	2.78355	3.337571	2.727975	3.255709	1.15773	1.357044	1.372551	1.904319	2.07837	5.360346
Cs4g14045	3.872592	8.241862	2.421416	5.340732	1.949964	3.319514	0.581721	3.094598	0.479545	1.449941
Cs4g14050	10.96246	20.9534	5.858237	10.99913	3.774022	5.213207	2.292737	7.744774	1.06493	3.873595
Cs4g14060	6.681942	13.22198	4.146464	5.597552	2.184802	3.151518	0.95649	3.367911	0.744138	1.432727
Cs4g14070	1.210136	0.784819	2.563185	2.112295	2.907544	3.381566	3.12898	4.199265	2.631488	2.524384
Cs4g14080	5.133296	10.21343	1.698795	2.445163	2.804037	2.300418	1.260347	1.142968	0.433652	0.161312
Cs4g14090	14.27267	15.04301	9.000493	7.791765	19.94722	18.37165	32.02394	26.05186	49.93032	42.63352
Cs4g14130	6.008025	5.558119	7.468935	7.216008	21.48533	16.64595	27.80589	21.09904	35.35502	30.20278
Cs4g14140	7.177631	5.025773	16.67026	14.14364	17.93621	18.80618	18.92639	32.70377	11.638	12.03218
Cs4g14150	9.216154	5.154007	5.522064	4.75625	5.643869	7.924079	3.001164	3.289659	0.301914	0.08901
Cs4g14510	0.999733	1.253258	2.145038	3.62454	1.152601	7.259433	0.966466	2.267926	1.019513	2.663371
Cs4g14590	0.156604	0.315975	0.753597	0.494348	2.111966	1.164142	1.791382	1.052805	0.06863	0.070923
Cs4g14610	6.652791	10.24513	3.642666	4.486464	2.231426	3.634288	1.794792	8.7713	2.013871	4.911599
Cs4g14630	0.072477	0.037552	0.139646	0.209691	0.193807	0.224417	0.134566	0.165797	0.2048	0.797105
Cs4g14890	75.33297	212.5538	112.9765	146.7116	9.174739	17.55519	46.02822	122.7904	53.13256	138.5023
Cs4g14895	41.64037	44.35538	53.30104	50.53749	54.80872	44.24544	21.19661	86.70333	30.18388	37.56265
Cs4g14960	7.186835	4.679895	4.67916	3.575645	5.251909	7.110973	3.347185	1.294583	3.092712	2.62584
Cs4g15040	4.697839	4.848512	10.27031	11.08417	6.604225	8.876329	3.976927	10.28391	5.711298	6.737465
Cs4g15250	0.127675	0.151485	0.738021	0.672792	0.534097	0.923861	1.772434	1.676756	0.197287	0.136508
Cs4g15340	21.7111	15.49613	20.76244	15.14591	25.22644	22.31277	18.36841	9.106147	8.925648	3.31806
Cs4g15360	95.71995	82.80284	82.65779	61.78017	69.42328	47.54782	45.01261	36.50545	61.92789	22.15958
Cs4g15440	2.229585	2.363269	2.276715	1.456469	0.370186	0.692723	1.249946	4.908113	0.070282	0.203124
Cs4g15530	0.462379	0.598109	0.516283	0.34649	0.768772	1.242062	0.304952	0.772946	1.087976	0.988658
Cs4g15550	1.426183	1.715438	1.492241	1.16821	2.213788	3.107713	6.155752	7.468285	4.069485	3.700315

Cs4g15560	0.008428	0.053208	0.020176	0.021982	0.139644	0.196934	1.08574	1.631175	0.115198	0.327529
Cs4g15590	192.285	242.5145	208.3482	194.7351	171.5564	164.5496	59.35159	191.3212	25.94084	61.11606
Cs4g15710	153.0771	153.446	132.4257	128.9717	102.8552	40.6312	23.17668	46.90319	6.153336	18.1921
Cs4g15735	56.63003	124.8311	166.9767	227.4535	13.6678	32.11434	57.61598	189.0886	36.69481	191.9129
Cs4g15770	4.628043	5.00617	13.79946	18.37798	7.915923	10.05504	9.491468	10.52382	2.362796	8.585811
Cs4g15890	25.79942	20.20018	20.47928	19.7593	30.23814	20.20603	48.15313	63.02449	65.12141	100.2381
Cs4g15910	4.192996	4.711982	3.298629	4.494699	1.083157	1.716659	3.247319	5.573321	6.836063	27.06684
Cs4g15920	0.671237	0.820402	0.619164	0.614992	0.325695	0.364271	0.545593	0.582273	0.779619	2.597494
Cs4g15925	3.063517	2.324663	3.241071	2.503836	3.140078	3.485447	1.241198	4.491926	3.894288	2.793988
Cs4g15930	25.13299	29.94847	18.79546	26.72919	6.890002	10.05102	19.84421	28.64706	41.71195	147.7912
Cs4g15980	10.84638	21.5761	2.518269	5.739124	5.854924	3.932701	2.962558	4.199309	0.609329	0.610144
Cs4g16000	6.4522	5.551844	9.908335	8.767922	10.66575	8.428324	15.98871	6.609042	3.510164	3.594987
Cs4g16020	13.99515	21.26703	20.12578	16.61662	7.814587	4.648843	6.22946	22.34467	0.567187	4.699441
Cs4g16030	4.803718	3.241115	2.57056	3.147052	2.024097	5.805139	2.761496	4.43993	16.21802	6.701299
Cs4g16050	16.93885	15.55215	36.78587	28.64461	64.79256	40.12022	67.91428	59.75142	76.09824	66.25085
Cs4g16075	0.429827	0.188166	0.870971	0.467493	0.991252	1.860618	0.72777	1.14019	0.199011	0.444552
Cs4g16130	8.185192	7.626757	5.178836	5.262245	1.787443	2.934738	0.785968	4.036219	0.087471	0.319815
Cs4g16160	0.208503	0.055368	0.24868	0.160701	1.045694	0.535364	1.30152	0.786371	0.00884	0
Cs4g16180	7.091416	4.761357	13.41874	15.5203	5.78012	15.43909	5.49663	8.789654	3.755962	21.39072
Cs4g16200	0.202521	0.316335	1.937543	1.88723	1.200057	3.404589	1.464492	2.203323	0.385987	1.81126
Cs4g16260	11.22408	12.66456	9.259484	9.925628	6.160155	5.196578	6.065246	6.278567	1.957325	2.689693
Cs4g16270	16.24074	16.91803	13.58789	11.91261	8.811136	6.67943	8.134405	7.931126	2.635274	2.637166
Cs4g16360	12.14642	11.782	13.03279	13.4082	21.55809	28.83214	12.71327	19.01365	43.56367	32.50838
Cs4g16650	26.78298	27.11285	79.77398	70.26594	100.4035	53.66795	54.63839	23.92185	16.35897	10.53018
Cs4g16680	3.681306	2.869435	6.496951	5.961615	2.332575	4.759032	0.785175	1.097347	0.428595	0.134575
Cs4g16750	15.34805	21.31005	30.11084	30.64317	6.74152	7.888126	14.74706	17.4485	5.895292	10.38624
Cs4g16770	0.042271	0.071116	3.16512	13.057	5.725396	21.4784	0.555612	32.18668	4.75401	66.21921
Cs4g16860	78.85931	16.55666	75.78304	57.56189	201.1604	67.26011	153.0678	11.90508	18.40714	2.031918
Cs4g16920	17.18849	26.54019	14.6063	18.4737	10.54591	10.23953	4.29997	10.76495	7.939133	11.60418
Cs4g17050	4.233077	1.565533	3.929399	3.800232	5.570319	1.274797	2.963859	1.439337	1.354893	1.453113
Cs4g17095	24.76052	20.6427	8.684724	7.694803	3.365418	2.934429	2.180189	3.480857	0.189976	0.136437
Cs4g17100	81.58319	78.99306	38.90093	45.15985	15.07238	20.47165	8.709674	19.71168	1.995858	2.439747

Cs4g17210	1.005992	0.629053	2.114713	1.403551	2.581512	2.583317	3.589305	1.465134	2.573128	2.332336
Cs4g17260	46.54977	67.33225	45.80125	61.99567	11.48233	14.10093	39.43698	57.25599	9.85035	41.05204
Cs4g17300	6.631833	5.001916	14.60279	16.41522	6.498183	15.45077	2.657907	8.135617	5.540788	12.47142
Cs4g17350	4.581605	4.7224	9.866853	8.238957	12.53156	13.74426	3.244631	17.12467	26.38877	23.61392
Cs4g17380	3.226948	4.426935	3.309117	2.978918	2.77228	2.463224	1.21336	1.848315	0.821088	0.827868
Cs4g17400	1.31056	0.916938	6.38078	5.228823	1.167113	2.514081	1.56865	2.63957	0.23345	0.793108
Cs4g17500	1.090592	0.82235	2.399231	1.485078	2.541784	2.598461	6.413133	1.804475	1.82958	2.368538
Cs4g17520	2.570963	1.82697	5.767938	3.087707	6.602052	7.175736	5.643189	4.956112	1.212671	3.570098
Cs4g17540	4.965141	2.895466	11.42949	6.988876	13.40257	17.8687	20.49705	15.57082	6.856828	16.07067
Cs4g17630	9.083762	7.239689	4.81725	4.252067	13.64532	28.571	27.7584	17.68738	63.57574	18.87802
Cs4g17690	0.390088	0.462135	1.021248	1.140781	1.525844	1.302833	1.618763	2.095982	0.756035	1.148203
Cs4g17780	0.787915	1.122287	2.608409	3.033316	6.506734	3.18431	9.635002	7.036514	3.301405	2.254561
Cs4g17785	3.097568	1.882289	3.257975	2.137995	2.832022	2.190615	3.324786	1.603889	1.175137	0.401314
Cs4g17810	14.5162	17.53268	11.98027	16.97249	6.555136	8.756837	3.076817	12.68293	1.643215	4.155396
Cs4g17820	60.20725	83.6432	74.82723	82.90852	54.1935	57.2618	27.13027	109.2362	46.2979	65.60886
Cs4g17830	33.78456	39.68824	21.77819	29.34915	19.82154	15.02842	11.02339	14.83246	2.48178	3.544387
Cs4g17850	27.12653	29.84576	39.12395	33.09676	59.75488	46.60228	82.6292	82.71684	41.07205	38.72624
Cs4g17870	8.43689	12.32803	61.38476	74.50576	50.37969	224.0592	45.55568	133.7373	10.12687	17.06758
Cs4g17890	2.16287	2.407228	2.502116	2.952871	2.561722	2.564158	0.764565	2.545582	0.725822	1.353386
Cs4g17900	0.794721	1.537509	1.43705	1.236907	5.086065	2.348272	3.114337	2.894798	0.279094	0.46493
Cs4g17930	21.61621	27.58186	25.14439	26.11164	21.35972	27.40292	10.28211	22.86044	10.48132	9.985599
Cs4g17960	26.42919	8.111144	9.095307	12.93392	6.413804	12.62727	10.52742	4.219573	21.08317	36.28596
Cs4g18000	6.039458	3.605198	11.59626	12.7833	23.11065	45.98877	52.14234	25.40397	21.11468	18.1539
Cs4g18090	2.638976	6.705205	10.51609	14.06661	26.31058	23.34228	79.72757	30.65769	140.0408	136.3915
Cs4g18120	16.19263	18.05283	76.43965	72.70808	49.83466	88.95271	64.19167	45.71361	56.62903	110.6489
Cs4g18220	2.919658	2.65913	2.125798	1.842233	4.713082	2.277834	3.757185	3.11557	0.426027	0.849146
Cs4g18230	0.552438	0.49472	0.777992	1.020717	1.117428	1.12923	0.627783	1.633584	0.909486	1.060178
Cs4g18240	60.36507	62.86809	122.7715	92.33224	152.682	66.36148	106.5109	106.2967	51.02238	167.1329
Cs4g18280	21.91272	31.00711	52.10772	62.11841	5.030168	12.02177	11.60794	23.28881	2.71163	7.871085
Cs4g18290	0.806609	0.839769	0.404678	0.90877	0.381694	0.381254	0.471899	1.02501	0.561824	0.586971
Cs4g18300	0.491158	0.414306	0.589749	0.531083	0.448389	1.07124	0.42234	1.02602	0.308738	0.65836
Cs4g18320	0.806081	0.760957	0.957689	0.861838	3.009148	0.614892	5.392508	2.955994	0.553822	1.320797

Cs4g18350	12.02696	11.94113	9.984447	7.236008	6.746342	9.983729	5.15683	5.498005	9.555499	8.464521
Cs4g18370	1.53574	1.783668	2.476529	2.451845	1.856503	3.085494	1.736203	5.746894	13.92609	20.86885
Cs4g18380	5.069551	4.79272	5.741574	5.48578	2.939207	4.926118	1.757529	4.217812	12.20421	23.39946
Cs4g18420	1.385634	1.544695	0.816002	1.08178	0.223449	0.466265	0.22541	1.188451	0.110915	0.125264
Cs4g18430	17.55493	37.42179	14.40167	19.4423	1.833612	5.670336	6.444761	35.95354	5.528841	24.09341
Cs4g18450	9.243481	15.18122	9.398809	11.96665	26.78707	33.69869	83.89683	62.93059	46.45126	30.82417
Cs4g18470	3.746624	4.066279	4.280527	6.894882	0.873614	1.79197	0.755506	1.702303	0.859578	0.90337
Cs4g18540	273.4466	295.4831	10.03246	57.61011	0.814716	1.101974	0.294045	0.190923	0.470066	0.491506
Cs4g18570	54.57255	23.08809	24.10776	31.15246	22.84389	27.00315	32.55958	9.122547	38.08072	38.24919
Cs4g18590	28.74872	31.59395	34.36741	39.65343	37.35586	55.35987	10.76123	39.3291	51.11095	47.62874
Cs4g18620	10.68437	13.10568	6.932353	7.619873	5.008721	7.456924	2.645011	4.812228	5.249142	6.054424
Cs4g18650	0.701585	0.591173	0.441121	0.50111	0.341284	1.017466	0.224548	0.420898	0.075976	0.101076
Cs4g18660	59.83452	47.92458	25.51251	28.4941	10.77639	28.34462	7.173707	13.47751	58.42168	45.43326
Cs4g18720	51.05259	56.81842	63.35696	68.50992	60.31014	56.69917	27.74556	65.2011	27.43739	49.67386
Cs4g18740	11.6453	5.12935	8.896179	7.264158	7.969587	9.814044	5.594313	4.51597	18.65461	20.63932
Cs4g18790	44.27951	41.75318	346.9742	190.2613	636.5798	209.6966	1166.302	952.4158	168.8224	441.6411
Cs4g18820	69.84541	136.4224	136.97	134.672	167.4909	98.40918	205.0383	530.8148	170.8493	474.8895
Cs4g18910	1.071372	0.864485	2.185441	1.769457	1.525183	2.362275	0.937471	4.765934	1.041098	1.079383
Cs4g18970	0.83226	0.616187	0.457297	0.56865	1.111279	1.278098	1.341404	1.346233	1.885524	2.423956
Cs4g18980	1.342382	1.05617	1.689793	0.888554	2.005243	1.134557	4.259697	10.50431	4.683544	4.219162
Cs4g19000	23.17536	19.27782	37.82495	41.20977	79.60595	86.67283	51.30276	80.28107	100.5335	120.7947
Cs4g19060	7.213628	8.37608	12.11485	12.5987	5.964166	8.30659	2.050665	2.46286	0.549989	1.188526
Cs4g19080	0.417963	0.665044	0.844623	0.635964	0.371267	0.339776	0.200197	0.830362	0.055563	0.085141
Cs4g19230	10.65715	8.286495	6.246947	5.077269	26.07644	18.91236	14.39599	14.03919	0.060532	0.227267
Cs4g19270	65.56955	65.04377	29.51536	38.08748	52.60376	34.68913	95.73564	38.39728	62.65716	26.79524
Cs4g19300	0.007773	0	0	0.00876	0.022939	0.169463	0.195455	1.050718	1.447572	0.493266
Cs4g19310	0.350137	0.279249	1.090232	1.952105	0.786279	2.208171	0.803749	1.294383	9.439079	14.227
Cs4g19320	7.239157	7.07792	8.256519	6.789814	7.553047	9.678044	3.739657	9.515632	7.933928	7.437651
Cs4g19400	30.53206	31.25707	25.51378	26.93165	30.91785	23.42099	31.16465	60.65929	11.30227	36.77959
Cs4g19430	0.452364	0.212096	0.509983	0.441422	0.632868	0.398597	0.900823	0.918355	1.51021	1.901888
Cs4g19480	47.72388	39.3635	28.26689	16.30738	27.85883	13.19133	5.009275	3.183019	1.323728	0.194433
Cs4g19540	4.506119	4.359363	8.754063	7.913495	2.059101	3.423014	3.139541	4.091494	0.942063	2.941478

Cs4g19560	12.52529	15.23425	16.15023	17.31784	3.530617	3.41417	3.901336	6.094154	0.878128	2.17618
Cs4g19605	9.242215	10.05748	10.34959	9.946669	9.327642	11.46185	4.561962	10.29085	10.03505	12.56658
Cs4g19660	4.48411	1.17876	18.44434	7.885833	11.92546	19.74015	4.210975	3.231275	0.670476	1.71786
Cs4g19740	7.742773	8.466266	0.906382	0.463219	0.596326	1.148457	2.119318	3.849583	2.929343	4.74894
Cs4g19860	49.25939	57.9776	64.77535	63.61195	47.55016	66.862	44.18978	86.52226	38.41685	81.7249
Cs4g19890	0.282165	0.223054	0.502849	0.653332	1.330567	1.109121	0.95547	1.359666	0.702854	0.66891
Cs4g19900	8.724292	8.445088	5.451354	5.212288	3.562106	4.037219	2.317868	3.135195	0.692901	0.449445
Cs4g20090	1.489659	0.466885	0.508276	0.303847	0.626098	1.269991	0.657452	1.250982	1.052429	1.481078
Cs4g20100	4.676506	4.360873	6.787211	6.932199	7.202538	8.7066	4.470937	10.46878	7.228698	5.731278
Cs4g20110	30.27039	20.40719	12.31007	11.54775	12.46513	8.35927	11.80001	8.599068	2.019088	1.047034
Cs4g20130	149.186	61.71288	78.37205	93.73267	171.0383	188.4518	78.65818	58.38077	111.0632	111.9132
Cs4g20160	0.972796	0.861393	5.782052	4.014878	4.824701	5.510148	1.910738	6.221251	0.88873	1.562859
Cs4g20170	0.932744	1.539475	0.595032	0.726033	1.068856	1.224914	1.412168	0.573632	0.2758	0.066143
Cs4g20300	8.382417	8.992661	4.998661	6.428262	1.360645	1.153237	0.42926	3.004958	0.252563	0.333272
Cs4g20340	4.063759	3.492415	3.811809	3.723097	5.109753	4.359708	3.003824	5.130123	11.06201	3.81382
Cs4g20350	0.042405	0	0.014148	0.045578	0.156514	0.065865	0.954689	0.271447	3.477778	0.262086
Cs4g20420	22.48798	21.54145	104.1616	86.63982	246.1213	173.8577	334.8032	255.4821	479.0606	322.01
Cs4g20430	7.6681	6.742757	10.11404	10.36301	14.83349	14.76422	25.02448	16.61679	61.99361	88.88163
Cs4g20560	130.0725	105.5643	145.7492	153.1317	72.75608	100.4686	32.46634	41.25081	20.54782	13.1539
Cs4g20590	186.9375	149.6022	201.0587	237.8543	105.8759	142.2892	45.17337	56.05765	28.57761	18.48877
Cs5g01020	23.45075	22.71132	20.479	26.93749	4.290257	7.576401	4.499377	7.784547	4.36997	3.180737
Cs5g01220	9.17106	12.35057	16.72323	13.52123	17.68179	7.362169	13.74312	14.50422	3.473597	4.737498
Cs5g01250	2.425588	2.106371	1.687568	2.545173	0.454977	1.090298	0.473239	1.134746	0.707754	0.475883
Cs5g01260	97.99796	114.6233	105.8943	159.5235	25.34686	68.93939	60.64107	61.63785	621.7093	606.3718
Cs5g01380	0.953037	1.943284	0.232206	0.447909	0.055249	0.053271	0.081894	0.30942	0.017228	0.025797
Cs5g01410	8.370354	19.24836	1.818344	6.800348	0.790236	0.579315	0.802577	2.959517	0.181265	0.154053
Cs5g01440	0.71308	0.754134	1.57493	0.727738	1.132948	1.02712	1.702883	5.980045	0.246528	1.510045
Cs5g01460	0.835115	0.738562	0.316502	0.305414	0.219956	0.391049	0.191883	3.51839	0.058995	0.019202
Cs5g01480	82.58749	58.91231	80.47927	61.14587	35.36464	14.24689	2.558386	2.755426	0.388955	0.158856
Cs5g01500	1.330916	0.690755	0.145724	0.126207	0	0	0	0.007122	0	0.007029
Cs5g01560	0.068229	0.231405	0.37057	0.344126	0.22279	0.496985	1.00155	1.149925	0.275069	0.528969
Cs5g01570	3.960213	4.084417	5.379638	5.754748	3.883678	5.771832	5.811715	17.86381	47.21206	35.22913

Cs5g01660	0.10763	0.015674	0.458419	0.064042	1.070232	0.469515	0.130561	0.056382	0.007307	0.025506
Cs5g01720	14.41608	16.08068	20.12165	19.32508	12.32874	17.18033	13.85983	24.80457	7.116496	16.5228
Cs5g01770	3.473076	4.936422	2.091244	1.909973	0.759762	0.301407	0.899599	0.917131	0.130517	0.149775
Cs5g01775	3.038706	2.846174	4.279643	4.287381	4.428237	8.154188	0.928231	2.504705	1.495594	1.217939
Cs5g01840	58.57955	43.09107	106.0961	74.61192	157.6724	160.8665	178.9901	112.0945	317.0346	232.2064
Cs5g01950	10.83686	9.959094	10.70397	11.30138	7.333919	10.40062	9.76392	12.1664	0.861202	3.096121
Cs5g01960	0.380895	0.757435	1.076376	1.595183	1.505104	0.794538	0.477244	1.353843	0.391451	1.261768
Cs5g01970	6.117597	9.171514	17.08221	25.19505	17.81374	13.21212	25.49935	28.23703	18.3577	45.319
Cs5g01990	26.43331	18.9243	6.212298	8.529945	2.457618	3.249654	8.633394	8.50694	0.667336	0.387158
Cs5g02010	44.77243	95.38134	8.757017	22.14624	7.510948	5.138394	13.42537	16.7516	4.318251	15.43016
Cs5g02110	1.622758	1.513273	1.179091	1.118405	0.42614	0.820984	0.781806	1.172198	0.597547	7.320287
Cs5g02120	8.902708	10.29778	7.234733	6.431464	5.624485	6.827869	3.376405	4.304774	2.561038	2.737973
Cs5g02190	29.53877	27.8033	29.32106	32.10493	25.57288	37.9871	11.08382	34.87699	34.19592	39.54728
Cs5g02200	39.11314	48.0583	36.7084	47.77474	23.00606	18.61696	13.80615	35.67421	5.703761	16.96349
Cs5g02210	10.85424	10.29504	23.01682	20.91704	45.71689	36.09162	26.12773	43.66097	11.45508	19.39787
Cs5g02220	14.02701	16.63257	16.09545	16.69062	18.95567	34.70863	15.84917	20.2284	1.546094	5.780281
Cs5g02230	8.766508	8.963532	13.43657	8.722339	5.305289	5.147222	3.500454	10.34918	0.077608	0.515319
Cs5g02250	0.188964	0.192831	0.661325	0.336611	0.877225	0.750072	1.340738	0.817585	0.545748	0.859356
Cs5g02260	2.718914	2.630771	6.18969	4.924272	5.987195	5.282487	7.984911	9.515702	6.077082	9.306951
Cs5g02310	0.548624	0.728348	0.626953	0.303858	0.659066	0.295702	0.247575	0.597431	1.025959	2.688847
Cs5g02340	44.20902	22.49837	32.49573	20.14259	118.5462	102.7689	168.8001	110.6912	785.4413	296.8469
Cs5g02380	4.509295	7.242122	2.174085	2.819722	0.525449	0.706132	1.047289	3.705923	2.523144	4.949256
Cs5g02440	30.24362	41.56628	65.32602	76.61024	6.02568	32.64194	22.41051	54.67792	20.13244	148.8648
Cs5g02450	8.56872	12.39491	18.25255	22.21843	2.156419	9.963714	5.854731	11.6458	4.953526	35.23296
Cs5g02480	0.96367	1.060693	1.340661	1.379024	3.687603	4.090237	10.27963	12.69221	8.372463	15.29611
Cs5g02490	0.881959	1.222867	0.418156	0.442339	0.158867	0.348529	0.378686	1.004997	1.761622	0.775018
Cs5g02510	0	0	0	0	0	0.007351	0	0	0.197428	0.966266
Cs5g02570	10.80458	9.77506	7.541599	7.0767	4.994549	4.985823	2.283575	3.767536	2.207881	1.463551
Cs5g02590	1.28827	1.405416	2.793483	2.635121	1.712008	2.014421	1.588806	3.255757	3.900169	9.069924
Cs5g02660	1.074281	1.392625	0.944799	0.985896	1.552024	1.799321	1.315741	1.297114	24.86503	58.12178
Cs5g02720	24.68433	19.48033	33.88651	25.04978	52.91513	41.32458	134.6361	46.73804	61.48475	42.43749
Cs5g02760	2.225533	2.116011	3.966183	4.25303	3.052841	4.731932	1.401887	3.423735	6.67149	8.714679

Cs5g02790	0.028983	0.143545	0.724375	0.457452	0.792676	8.385915	4.657308	74.17026	0.133368	1.226719
Cs5g02810	9.200103	10.32278	6.678463	7.609708	3.076421	4.671674	1.04404	6.444088	1.202746	2.038961
Cs5g02820	11.58482	10.77316	12.6946	4.795687	7.827694	8.360483	6.618547	11.84797	32.63091	5.291155
Cs5g02940	3.378105	1.726277	2.08623	3.24384	5.453264	7.938896	12.661	9.520851	51.2346	25.36493
Cs5g03010	0.417322	0.405593	0.240603	0.565827	0.061401	0.969003	1.037114	1.045324	0.016838	0.307352
Cs5g03060	23.72299	42.18704	60.41765	64.81962	3.024285	4.4879	9.499664	22.94038	1.218052	3.77258
Cs5g03070	18.15347	27.55941	32.52198	31.5891	16.81382	23.44926	2.765342	9.401184	1.748588	1.247619
Cs5g03100	1.718605	2.122241	1.410142	1.296744	0.827412	1.012128	0.567938	1.279561	0.641621	2.245824
Cs5g03136	1.658801	2.191457	2.186918	2.545434	1.889941	1.634758	0.341542	1.458032	0.07597	0.22147
Cs5g03240	23.99548	25.1906	14.43763	15.45773	5.575671	6.086873	2.727648	4.858337	1.623172	0.932391
Cs5g03270	4.181214	3.994082	2.795234	2.764469	3.208985	4.020932	4.383625	2.82785	5.257879	2.204865
Cs5g03280	8.875729	8.902221	20.71202	17.91722	41.78116	41.29841	40.82364	36.71657	17.81784	27.34603
Cs5g03330	56.27443	63.25295	53.0864	72.29808	20.24899	37.02001	16.15731	56.56925	80.7829	306.0423
Cs5g03340	10.79482	13.82468	7.351879	7.327394	2.770665	5.958423	1.519268	10.86211	0.971797	2.098581
Cs5g03360	3.656624	3.498904	4.071044	3.493996	3.943193	3.530036	1.819964	2.165395	2.189495	4.636413
Cs5g03420	51.85834	51.02385	104.4385	113.0292	16.93262	32.03075	39.94342	60.68854	14.75824	45.02172
Cs5g03460	28.04437	46.25027	18.38065	24.11318	9.190945	12.47425	35.25793	51.95357	1.963328	4.435199
Cs5g03490	4.470856	2.909717	6.335562	4.587481	10.16969	8.726029	13.0772	8.165273	12.85573	7.984567
Cs5g03500	2.749079	2.318966	2.69441	2.239841	3.128147	3.215378	7.015134	6.921839	4.937005	5.910744
Cs5g03510	6.506396	3.98499	17.20253	10.91051	22.50432	22.47171	27.00133	9.459707	12.56589	8.835334
Cs5g03560	3.606281	3.206772	3.955716	4.039461	5.300934	7.546972	7.7493	7.937843	25.37352	13.06364
Cs5g03570	6.099107	7.107752	6.837944	9.822525	1.688784	4.776295	2.720778	3.887812	3.619629	8.325078
Cs5g03580	6.972811	6.283304	5.939085	6.164676	2.639672	3.397038	1.328438	2.777197	0.11985	0.434373
Cs5g03620	2.485611	2.743009	1.963113	2.681997	1.572382	3.041536	1.302811	7.262964	2.228419	3.030124
Cs5g03630	13.02507	17.37874	9.493374	8.812183	4.877235	5.303727	1.199035	6.521083	3.020369	2.83078
Cs5g03680	0.099315	0.131055	0.266823	0.413418	0.256291	0.792812	0.573529	0.361005	0.465408	0.988684
Cs5g03690	1.338753	0.982308	1.882171	2.275304	1.852209	2.368411	0.243234	1.244902	1.72618	0.952869
Cs5g03730	1.705071	1.041596	2.387245	1.686674	3.371224	3.549539	1.447683	3.78949	0.390037	0.592079
Cs5g03780	0.194407	0.077651	0.279897	0.202029	0.516137	0.287728	0.854886	0.193367	0.258649	0.231901
Cs5g03790	1.704799	1.282437	2.346541	1.794622	4.59638	3.303694	7.988933	3.649751	2.590624	2.111309
Cs5g03980	12.04646	13.03376	9.008888	6.586988	6.85193	4.48838	1.959583	1.340283	0.166575	0.147872
Cs5g04020	2.867802	2.995633	4.507814	3.590589	5.55017	7.099964	15.38082	18.40786	9.866534	12.83457

Cs5g04030	33.77745	33.52313	47.06374	42.53233	46.69455	55.17629	23.8594	50.96951	25.65887	24.82189
Cs5g04080	8.709765	5.700455	11.06244	8.744518	9.846182	23.18224	4.256017	3.706024	39.35493	14.61458
Cs5g04210	0.912708	1.987532	0.227594	0.275211	0.118312	0.125034	0.093298	0.222764	0.044929	0.072541
Cs5g04270	9.885676	25.77748	4.245977	6.680579	6.075781	6.71051	14.79578	22.11323	5.720208	7.3308
Cs5g04280	9.978086	11.96552	3.149487	2.989993	1.178278	2.72596	3.6122	9.590962	0.045384	0.118197
Cs5g04290	299.4472	308.1709	129.5918	102.5027	114.964	56.58985	39.34145	44.68651	4.00051	2.907801
Cs5g04320	0.670798	0.564722	1.298282	2.003161	1.747494	2.2304	2.796314	3.295677	12.80723	9.714137
Cs5g04330	47.62272	24.98186	118.9861	113.1866	539.3368	468.0812	264.338	247.7772	406.2092	144.7774
Cs5g04340	29.49496	15.73995	25.97029	20.34041	43.01845	33.99278	53.91765	51.37596	107.8196	85.33273
Cs5g04350	25.88183	15.91451	20.61154	19.76597	51.60595	42.25413	58.9432	64.44637	131.0156	99.94693
Cs5g04430	56.66055	70.6286	40.36109	40.58064	48.35465	20.25882	57.41388	37.09081	10.56756	6.262731
Cs5g04570	0.814597	0.588383	0.596593	0.548992	0.295553	0.650723	0.358885	0.986595	0.13637	0.877551
Cs5g04580	11.37752	11.46917	14.80454	15.27355	8.836978	13.38296	3.726179	8.359375	4.898829	3.91239
Cs5g04620	29.8918	41.05803	28.73411	37.3456	8.944079	11.58118	6.282158	13.07153	2.076807	4.197342
Cs5g04670	1.9054	2.392762	2.60795	2.509193	4.080934	2.353566	1.033989	3.212428	0.671184	0.655199
Cs5g04770	39.44994	22.95907	17.74433	14.52615	12.51778	18.463	11.58382	9.083599	11.82178	2.922222
Cs5g04790	17.18437	14.40883	39.71183	36.67505	32.67555	43.77428	29.51826	37.50445	29.24129	61.30387
Cs5g04810	4.100524	3.936898	3.873497	2.699048	1.353279	2.931017	0.691003	1.826192	1.557078	2.086909
Cs5g04870	42.42587	33.76609	27.35864	27.94837	13.1937	24.95077	8.835875	22.34848	16.5214	22.08248
Cs5g04890	86.07284	111.1809	109.5276	130.7622	29.42608	47.52449	38.11354	102.6577	37.83749	96.14566
Cs5g04910	0.067932	0.048551	0.295132	0.220033	1.20799	1.459827	1.734034	0.935777	0.063301	0.150266
Cs5g05040	2.254707	6.938305	0.655095	1.823364	0.241941	1.032305	0.1494	1.554313	0.145287	0.100357
Cs5g05180	10.82554	14.17813	13.35577	9.207613	8.53137	4.799931	5.693047	11.74708	1.258595	2.754837
Cs5g05200	7.058304	6.605813	4.23156	2.926703	1.637756	2.217029	0.430939	1.56443	1.507964	3.895828
Cs5g05240	110.0105	146.5255	38.65137	62.96686	11.9288	7.019115	3.644942	3.062836	2.921901	4.773066
Cs5g05260	259.5685	182.3428	97.71827	81.06641	30.9329	22.51176	46.85126	62.44602	32.73402	43.61302
Cs5g05300	42.99544	41.74138	41.46964	51.35522	4.258081	25.10997	4.34792	31.76606	16.00418	59.1444
Cs5g05310	8.284093	8.59087	6.526108	8.497622	2.77577	5.76825	2.406958	3.848425	2.203873	4.648039
Cs5g05410	5.493443	15.10132	24.4163	23.36502	34.49889	18.88809	14.23984	5.747196	10.27565	6.142935
Cs5g05450	1.774768	3.580541	1.602867	2.077012	1.701389	1.600975	0.516762	1.767127	0.826742	0.827891
Cs5g05460	6.669038	8.972585	5.949212	4.922368	2.652061	3.67235	1.969905	4.866843	6.136122	7.195781
Cs5g05540	7.83232	8.498068	7.122455	7.632477	2.235674	2.710162	4.028829	6.396241	0.904242	2.947844

Cs5g05570	22.84292	15.24376	18.63988	20.7283	41.80204	56.74152	82.6005	41.27119	128.2172	79.4868
Cs5g05590	1.177756	3.715226	0.708517	0.983506	4.874005	4.105231	3.686964	4.138019	1.72785	1.904929
Cs5g05640	0.150557	0.213176	0.159365	0.246901	0.689287	0.307454	0.879393	2.646712	0.490039	0.344352
Cs5g05670	15.89754	18.45681	5.122045	9.85424	0.944832	1.589191	0.158003	0.8001	0.157647	0.087866
Cs5g05720	7.487432	9.278457	8.328478	9.745021	5.469621	6.759878	3.468672	8.235426	2.916923	5.114153
Cs5g05870	4.208448	3.705615	11.62295	12.0848	7.773048	12.70414	1.261434	1.409842	0.51809	0.517697
Cs5g05975	2.374658	3.163991	2.553452	2.997364	2.298259	2.846838	1.025907	2.870683	1.67286	2.321133
Cs5g06050	17.08138	13.54056	8.610965	8.300742	10.0617	11.46261	16.39981	7.425293	22.18378	10.19911
Cs5g06080	3.479887	3.19715	4.62603	4.093861	6.430676	7.791735	7.42356	5.585903	16.04801	19.2505
Cs5g06130	1.236185	0.895115	2.314252	1.590299	2.114699	2.500348	5.00398	1.777435	2.395767	2.062661
Cs5g06150	10.41792	19.24173	5.722717	11.81586	0.926759	1.809322	0.305769	2.825733	0	0.033667
Cs5g06180	28.32142	33.78282	22.90461	27.46886	33.03582	18.05236	18.41824	27.5593	3.921126	8.662312
Cs5g06310	0.093164	0.067074	0.459977	0.299174	0.154125	0.245565	0.010039	0.320302	0.292535	0.795558
Cs5g06360	2.609894	3.583935	3.120045	2.883181	3.529574	4.038107	8.831399	9.55889	0.679657	3.897943
Cs5g06380	2.37849	2.24327	1.976642	1.954882	0.506179	1.021932	0.159797	0.902058	0.431493	0.291394
Cs5g06390	1.556816	3.70451	0.555117	1.037501	0.207461	0.263884	0.212514	0.402693	0	0
Cs5g06400	30.87246	30.87289	17.61299	22.0179	7.00788	12.8546	6.510452	11.93029	2.335995	4.590613
Cs5g06430	14.36602	16.35362	7.506441	6.588576	4.593415	4.430275	3.775521	4.281041	0.058168	0.022151
Cs5g06500	0.438131	0.398685	0.862378	0.569132	0.635995	1.390977	0.140063	0.337089	0.334022	0.255649
Cs5g06510	76.80595	60.22318	102.6076	102.1278	51.59294	102.5221	35.79128	50.37736	79.10988	115.9063
Cs5g06590	27.15525	23.33155	21.56571	29.12245	4.544302	9.795516	11.85607	15.03387	23.63512	47.33751
Cs5g06600	29.24147	22.97897	49.2073	36.83123	53.74883	62.16006	178.1855	141.6523	33.44556	33.15391
Cs5g06660	6.882459	8.331079	4.984035	8.066266	4.005323	4.560588	2.826026	7.415742	3.811453	5.12829
Cs5g06840	21.8643	24.58916	31.40353	32.80736	25.40124	30.34416	11.06329	28.23012	12.83278	20.20987
Cs5g06920	30.08157	25.89152	13.99595	5.385518	22.11667	4.494937	13.23646	5.739725	3.092082	2.356487
Cs5g06970	0.477507	0.643505	0.67261	0.56705	0.333064	0.367213	0.298426	0.730989	0.162865	0.434198
Cs5g06980	2.18786	1.695833	6.120926	5.529305	8.25776	14.15006	10.30152	8.913759	19.08994	28.05607
Cs5g07160	13.41232	27.13041	32.52831	31.97006	3.113909	3.094438	12.83641	35.04803	6.176963	21.99139
Cs5g07230	11.8319	7.206979	2.897735	6.597416	0.337827	2.094177	23.71952	6.474535	137.8318	48.27726
Cs5g07440	2.313393	2.459409	2.161205	2.504068	2.005203	2.341933	1.298473	3.087556	2.591399	2.147929
Cs5g07470	2.812217	3.019148	2.632227	3.062903	2.148977	2.612954	1.366544	3.797477	3.080075	2.981905
Cs5g07550	20.38036	18.42139	3.050912	4.440042	1.217975	1.499003	1.025687	1.606435	1.853457	3.280536

Cs5g07570	33.78964	49.98337	29.63113	40.83263	8.640228	14.4101	18.20159	32.25295	58.61388	163.5366
Cs5g07650	8.405206	3.592289	0.920325	1.317457	0.436367	0.706245	0.263715	0.154801	0.13772	0.146187
Cs5g07690	52.79774	36.53829	47.22067	52.75716	31.32601	77.31166	25.70775	50.70502	117.7999	106.5658
Cs5g07780	19.0003	17.2486	15.15228	17.30123	10.07254	10.86977	8.417799	13.69685	2.585368	5.842754
Cs5g07820	6.553543	7.972063	13.79889	13.02158	17.10261	15.26354	20.1846	18.59524	12.45475	13.75537
Cs5g07856	0.536918	1.145237	0.285317	0.179486	0.028968	0	0	0.015054	0	0.014689
Cs5g07990	5.838032	11.9518	2.166404	4.128652	3.466025	2.70943	2.401844	3.50929	1.533611	2.200335
Cs5g08000	8.517053	7.627159	9.925374	10.36986	5.845912	12.17219	5.165524	11.90052	15.63281	22.75146
Cs5g08030	12.95262	16.41506	18.8447	26.65696	1.660182	3.127548	4.032344	9.405061	12.56021	9.388135
Cs5g08200	1.085584	1.026771	2.224686	1.460534	0.537242	0.78342	0.374163	1.056009	1.78056	1.468876
Cs5g08290	20.61057	29.96493	41.78632	49.81202	13.84315	17.92992	17.68004	22.59731	25.97113	36.35079
Cs5g08340	0.036466	0.162798	0.329444	0.214278	1.216973	0.791003	7.247876	6.106536	32.57707	23.08498
Cs5g08350	5.315546	5.471621	8.031748	8.448106	15.27134	15.55764	17.61638	17.32123	15.62987	16.62978
Cs5g08360	28.85142	31.8667	39.82577	72.64076	1.83668	6.975885	19.59329	17.9163	4.980491	22.26671
Cs5g08380	3.815989	4.903909	3.524138	8.7527	0.992578	3.541344	0.882755	3.741608	0.862034	2.022375
Cs5g08400	0.948805	0.891848	1.999693	1.336395	2.892583	2.465538	0.975133	1.338666	0.38819	0.556736
Cs5g08450	0.506398	0.27178	0.456417	0.492428	0.568257	0.443493	0.900187	0.602969	0.983236	0.342552
Cs5g08460	28.22828	35.97635	38.9421	34.36879	21.96247	33.09108	8.758368	8.325787	2.000283	1.748947
Cs5g08490	21.84675	20.92639	27.76377	30.82434	24.77137	29.95397	15.07596	36.05458	19.79757	25.39196
Cs5g08500	133.984	143.154	122.4077	151.7529	67.59925	64.73093	65.50293	77.53944	77.92267	228.1616
Cs5g08530	0.16363	0.249926	0.616144	0.833792	0.113847	0.195225	0.442214	0.491261	3.846585	16.29879
Cs5g08640	4.489207	4.277015	7.300855	5.846114	33.36232	30.2738	37.90403	40.7474	104.8505	163.9085
Cs5g08660	15.93396	21.14567	16.1354	16.74251	10.89917	12.71926	6.699059	10.28533	6.459214	14.44256
Cs5g08670	3.948832	3.983184	4.84032	5.824322	3.033331	6.100182	1.546527	6.332442	0.351786	0.45415
Cs5g08720	3.088087	4.49609	3.638684	3.589575	0.607245	1.761306	0.324537	1.395856	0.081215	0.057606
Cs5g08740	2.057401	1.743942	2.098617	1.572779	2.434072	2.600494	3.124275	3.739623	1.121756	0.50264
Cs5g08850	0.907506	0.528685	0.43393	0.381357	0.992928	1.912775	2.947076	3.065184	1.373804	3.338287
Cs5g08860	4.752225	5.460096	6.024218	4.59831	1.7047	2.651814	0.499379	2.15158	0.098098	0.128672
Cs5g08900	4.962141	5.464538	6.66939	7.228906	6.191459	7.728408	3.398942	9.245311	3.944736	4.952442
Cs5g08910	13.51016	7.894	3.686753	4.343	1.230816	2.088601	1.254913	6.801023	0.435089	1.826542
Cs5g08940	0.139297	0.233685	0.14501	0.140114	0.225384	0.605417	0.291757	1.180006	0.508105	0.875994
Cs5g08980	3.132502	2.705252	5.028212	4.951385	6.323734	5.562655	11.31589	5.087126	2.421994	2.237467

Cs5g09050	51.75544	85.71485	94.49759	68.21104	45.48112	16.67844	20.41851	8.326623	1.710088	0.654679
Cs5g09080	5.142378	6.13623	0.720754	1.341523	1.792876	1.499453	0.681032	1.732941	0	0.248659
Cs5g09130	17.58247	20.08769	31.40442	26.27812	39.97608	30.06436	39.40525	54.75229	22.76772	34.1314
Cs5g09180	23.83062	18.69244	14.45473	13.09635	10.26855	12.84379	7.719781	7.5397	11.94797	9.750942
Cs5g09200	4.517197	5.153583	2.492715	2.868573	0.944426	1.181168	0.725009	2.177403	3.746939	4.834157
Cs5g09220	34.79837	84.15002	26.76789	33.7245	17.51919	11.77816	22.85346	26.72044	23.54309	25.79609
Cs5g09240	4.635985	6.054787	2.011657	2.209801	1.080178	0.904522	1.074015	0.972115	0.207158	0.15319
Cs5g09250	17.40653	24.88308	12.4628	15.93588	12.08728	11.6718	5.890828	14.74829	12.90101	17.10544
Cs5g09260	18.59934	33.30861	16.31793	22.88787	13.07018	13.20813	7.572632	20.56908	12.63433	21.74627
Cs5g09290	8.197647	6.43855	2.482001	2.272265	2.815198	3.882915	2.45035	1.903302	0.844933	1.121425
Cs5g09360	65.85501	112.4725	61.33859	66.46486	39.62732	31.81621	15.26338	49.54559	6.728765	9.723686
Cs5g09370	2.098854	3.565928	2.647273	2.164085	3.961826	2.063793	4.13743	11.05854	5.158088	18.72324
Cs5g09390	29.46024	17.43106	34.62849	28.80858	64.01666	82.52288	77.5958	64.60201	262.2661	287.8521
Cs5g09450	13.30579	10.8589	13.75259	12.80493	13.25266	16.04756	17.8914	13.85347	20.28296	6.911719
Cs5g09480	0.667063	1.540922	0.588145	0.397944	0.199381	0.282779	0.029543	0.122673	0	0.155363
Cs5g09595	5.358033	8.802258	4.166806	6.158253	3.2444	3.287399	1.801701	5.713138	1.42981	2.040196
Cs5g09640	2.275155	3.614149	1.918647	3.953618	0.028204	0.126807	0	0	0.013113	0.010081
Cs5g09690	29.83124	18.33759	19.94235	16.43847	10.95968	13.27789	17.02324	29.52507	26.43738	38.67228
Cs5g09800	1.727666	1.013083	1.158052	1.11395	0.456311	0.909906	0.651948	2.566404	3.064656	3.094297
Cs5g09840	22.32774	26.46497	25.20774	16.85697	15.84098	13.12541	6.711543	8.4306	1.706713	1.332164
Cs5g09950	3.420355	2.648936	2.425921	4.231176	2.047565	3.103343	2.358274	2.829417	8.009753	3.739274
Cs5g09980	90.81202	124.9055	37.2521	69.93176	10.38259	8.167557	4.085432	11.60142	2.20797	3.053446
Cs5g10090	12.70104	5.655239	14.1377	11.13026	12.45457	19.96745	9.142104	11.02675	4.495154	7.465158
Cs5g10170	16.29535	17.90857	4.51954	5.370077	0.493238	2.615066	1.619529	13.26877	0.512616	0.254004
Cs5g10180	8.560208	4.867248	10.19604	7.315313	12.83533	27.57997	12.85407	10.52666	106.0742	48.57279
Cs5g10220	3.662022	3.895354	6.941702	8.230937	11.85759	9.272986	11.4186	13.72958	38.74009	45.44715
Cs5g10240	68.71502	77.11456	31.24336	35.03074	1.816516	4.407761	38.63226	43.15126	78.3772	123.423
Cs5g10250	15.99996	8.942105	3.160662	5.39715	0.235747	1.548956	3.406699	2.537049	0.177971	0.343906
Cs5g10280	4.00517	1.072403	0.976309	1.329855	0.876585	5.972388	0.533707	4.492447	3.068616	3.280035
Cs5g10330	23.21977	30.30783	12.86269	15.29438	9.769881	10.61944	8.521289	24.70484	16.88814	25.56417
Cs5g10430	37.8639	34.65103	24.83078	25.66747	15.35928	17.28011	11.34781	31.16461	64.8265	70.20906
Cs5g10480	1.143359	1.917797	2.616166	4.076951	0.209865	0.337295	0.715026	2.030185	0.159572	1.082135

Cs5g10530	23.97313	17.35523	19.18568	18.34125	4.72529	13.25866	11.735	71.90138	20.40451	82.07498
Cs5g10550	24.5269	24.83429	32.54903	26.48159	153.3986	34.91591	280.948	99.81874	206.4155	187.4548
Cs5g10560	0.81157	0.917923	1.977977	1.886641	0.718854	1.159548	0.63158	2.829565	0.539114	1.63184
Cs5g10630	0.275296	0.438971	0.338944	0.334619	0.39275	0.579316	0.317741	1.850366	0.520593	0.617083
Cs5g10640	5.757999	6.715617	6.45287	4.979226	5.467619	5.660354	2.131217	8.274504	1.760189	3.255634
Cs5g10660	17.25002	15.91202	24.14414	21.74193	31.3283	27.27439	43.02533	52.94324	17.78832	23.06344
Cs5g10670	105.8204	73.91464	88.32089	62.37052	117.8939	138.1492	89.04774	81.52316	60.56899	6.488067
Cs5g10730	28.44052	25.78661	48.32701	46.88613	82.70477	73.28843	163.3956	65.18733	100.9882	61.97612
Cs5g10740	21.52573	48.12643	6.283419	10.51714	13.51574	9.669709	23.62596	14.89517	0.789379	0.713756
Cs5g10760	10.77049	8.417665	9.064572	11.69457	1.55887	2.125784	1.812239	4.188711	0.514253	0.345055
Cs5g10770	8.786951	25.28024	0.675554	3.398651	5.029184	3.491395	10.22058	10.16727	2.824255	2.10201
Cs5g10800	8.049681	26.78933	3.696262	3.984921	5.995418	7.204244	1.90444	5.267624	2.558075	1.722591
Cs5g10820	386.9705	195.0711	1663.096	1215.598	2305.227	1556.396	2442.995	1308.355	1537.364	411.8797
Cs5g10830	14.21199	12.80708	4.141474	3.461062	1.099106	2.739807	0.228265	2.82933	0.83968	1.883984
Cs5g10850	34.69324	37.94713	15.31938	22.32451	1.294293	1.362027	0.174343	2.351375	0.157004	0.206239
Cs5g10870	6.86861	7.581394	10.48312	14.1225	4.764337	6.322636	1.117331	5.90679	8.993412	14.02398
Cs5g10920	71.8102	84.24053	55.95655	76.76503	31.36731	37.21042	26.29313	41.46479	34.50518	48.84438
Cs5g11210	14.30633	18.79932	17.26705	14.59338	35.67202	16.45679	49.34052	41.69091	18.28209	18.90217
Cs5g11280	113.7109	105.0394	85.01801	64.17823	35.23272	21.39912	7.95312	5.481893	0.569352	0.28741
Cs5g11310	1.609988	1.605856	3.055075	3.335622	0.997998	1.618071	3.768586	3.056451	28.27606	30.59083
Cs5g11380	0.231301	1.457757	0.388955	0.398986	0.067232	0.312819	0.0128	0.602868	0	0.065585
Cs5g11430	34.4855	41.49819	38.69326	41.67108	21.66861	26.20064	12.55374	37.22288	2.922044	7.915138
Cs5g11440	30.90573	22.35278	21.84251	20.0698	16.29083	34.66495	15.31121	24.8421	39.1205	38.73548
Cs5g11510	0.439188	0.334449	0.82492	0.763928	0.765175	0.838904	1.044102	1.981958	0.54768	1.068454
Cs5g11560	0.279548	0.203638	0.355672	0.397596	0.471193	1.339392	2.913182	2.554296	4.57126	7.19873
Cs5g11570	87.92179	51.9932	34.92188	42.16219	10.29281	17.87214	19.9997	25.15362	94.13471	156.9876
Cs5g11610	6.298117	5.990795	13.21818	5.25992	23.85115	13.37849	25.14011	19.9175	8.492411	7.679866
Cs5g11620	3.223705	3.24495	5.869904	3.245161	19.17169	17.76063	15.84795	17.30702	13.74049	17.3308
Cs5g11630	0.145614	0.524626	0.863851	0.815924	9.723919	12.23272	33.84547	34.11265	76.24988	57.56306
Cs5g11710	1.583295	1.380412	2.659845	3.067774	2.773006	1.812056	1.064678	2.563197	1.571769	2.652698
Cs5g11730	26.51107	23.05265	20.98773	16.04942	35.18723	17.9923	90.99656	34.65707	36.81526	18.35893
Cs5g11760	5.079022	3.747521	5.600147	2.69051	4.755181	3.577289	4.5566	2.132869	3.399155	1.412984

Cs5g11770	93.67498	94.21732	17.05099	27.09459	2.301575	2.415184	1.45214	4.364399	0.252837	0.169383
Cs5g11900	8.380755	9.070042	11.13742	12.61535	12.17149	13.14757	22.05548	24.31668	12.85863	20.4556
Cs5g11940	29.89391	34.85861	64.03915	43.56735	57.6127	43.63052	86.48186	34.49559	111.6702	109.0993
Cs5g12040	50.79256	65.28706	30.85714	51.72513	8.389864	16.88661	8.51246	20.01281	3.495835	7.859025
Cs5g12070	1.856735	2.759006	1.970275	1.929056	1.024956	1.031102	0.789955	1.644938	0.024081	0.147835
Cs5g12260	11.34598	14.9995	6.00727	6.743858	5.152755	3.497491	2.493735	1.943089	0.725734	1.293532
Cs5g12270	15.92633	19.1722	6.551123	6.422653	2.306786	1.44688	1.641296	4.186935	0.544466	0.547663
Cs5g12300	39.76549	29.93791	20.99333	20.04466	14.82672	18.83373	33.72983	47.67599	1.325853	0.9758
Cs5g12350	15.32238	24.17357	7.505889	8.265658	0.381563	0.617451	2.294804	5.826902	1.448118	3.321189
Cs5g12420	71.65278	77.57405	6.038301	5.557135	2.508545	2.566741	0.555031	1.494663	0.161921	0.128792
Cs5g12590	6.139898	7.031379	7.080872	6.588764	16.60354	7.730942	11.10812	10.34969	0.549221	0.742242
Cs5g12710	0.797917	0.574784	1.347573	1.224105	2.751773	5.400158	2.898019	2.038184	1.002638	1.073358
Cs5g12720	2.185878	2.388561	1.168355	1.812767	1.008797	2.403098	0.881434	3.193531	2.127089	3.680785
Cs5g12830	9.000074	9.299117	9.435897	10.94973	12.57143	13.25612	22.34757	25.62276	14.00051	19.68465
Cs5g12880	0.06239	0.461735	1.059769	0.784585	30.70728	38.39432	119.272	113.3096	139.9116	148.3368
Cs5g12890	0.032083	0.599964	1.363577	1.138097	38.18229	45.89706	150.4491	129.7129	162.8386	143.2973
Cs5g12900	0.238807	1.344404	2.917107	2.441919	74.23858	96.7401	295.0762	286.0286	342.6985	404.0492
Cs5g12910	2.659378	3.500363	2.848101	3.334545	2.5075	3.818259	1.178039	4.783698	1.778437	1.569948
Cs5g12920	2.42297	3.191089	2.431734	2.889674	2.32323	2.638011	0.924031	3.134364	1.145652	1.388642
Cs5g12990	3.274487	3.143338	3.630666	3.197624	1.403796	2.191871	0.343079	0.967122	0.231006	0.320493
Cs5g13010	7.945294	10.35116	2.74266	8.338859	10.38804	7.041745	20.82056	40.09991	40.50113	13.53688
Cs5g13070	6.959585	1.721912	2.993193	2.503382	7.935138	5.830133	28.31392	5.618967	75.92288	18.17162
Cs5g13170	0.592309	0.380137	1.158593	0.810612	0.457383	1.128107	0.058587	0.453715	0.311662	0.34151
Cs5g13200	1.032495	0.616717	1.559265	1.211934	0.882568	1.67648	0.166382	1.094959	0.303372	0.444193
Cs5g13265	3.613666	4.025764	3.144891	3.332783	3.071811	3.797037	1.734438	4.223136	3.406679	4.24071
Cs5g13290	14.3684	13.24971	4.410036	5.730277	1.596706	1.502223	0.853684	1.925037	1.570116	1.726168
Cs5g13450	8.975249	9.946849	3.146906	3.926969	0.722287	1.811223	0.27425	0.584656	0.87618	1.704186
Cs5g13500	3.41699	2.650109	3.37951	2.59921	2.908226	3.698628	0.821195	2.796567	2.174832	0.780539
Cs5g13510	27.45424	29.68665	16.25406	22.00452	13.83371	13.81055	22.73443	45.1346	36.47012	29.34646
Cs5g13540	37.32049	13.57558	16.46889	18.2394	6.65396	23.63052	6.087372	8.507122	30.86959	20.57745
Cs5g13580	0.013955	0.157646	0.758003	1.062202	5.974848	1.846942	2.628849	4.304122	15.00337	40.18654
Cs5g13670	1521.915	1506.381	291.711	452.5301	17.29117	12.20216	1.001695	1.29403	0.571836	0.683731

Cs5g13710	1.135732	0.842964	3.353367	2.147352	6.36283	6.682135	5.72642	3.624731	2.157531	2.315288
Cs5g13770	2.521149	2.549129	5.631828	6.639285	4.823965	4.149397	2.280037	5.857872	0.654641	0.37015
Cs5g13810	108.9455	109.6159	72.04379	83.13419	31.53298	39.90009	31.01074	20.53923	47.73	39.85286
Cs5g13820	7.335317	6.378815	13.23942	15.91217	13.55223	25.73545	3.464437	8.7087	4.787221	6.733667
Cs5g13860	143.366	162.889	24.76963	56.95806	3.381676	9.935186	0.51007	1.08361	0.102701	0.078231
Cs5g13870	25.62459	29.5343	16.00333	17.37487	7.148564	7.464473	3.963074	6.830535	5.959323	7.711895
Cs5g13890	4642.961	5195.333	224.2266	642.1015	25.47975	43.3715	4.646332	5.427357	1.852383	2.151243
Cs5g13940	1.614972	1.3637	4.310059	2.046277	9.353137	3.559506	11.54317	7.386514	3.53337	2.329424
Cs5g13960	1187.62	1336.054	82.07394	207.1457	7.92807	14.10601	1.325584	1.692997	0.70138	0.902209
Cs5g13970	6.776762	5.612693	16.54698	8.690767	34.63482	14.47728	39.43195	32.3199	14.89403	13.89174
Cs5g13980	2.026039	2.152048	4.957798	2.250077	11.86893	3.92258	12.21119	8.716399	4.015566	2.708916
Cs5g14050	3.377254	2.996828	4.692016	4.348216	4.827185	6.342348	1.969166	3.678457	1.810281	2.200348
Cs5g14090	0.090387	0.069825	0.346116	0.694304	0.065873	0.071128	0.035241	0.919488	0.166262	1.729158
Cs5g14320	1.226695	0.631148	3.006709	1.607283	3.968987	4.038944	5.6951	2.684924	2.446182	2.044158
Cs5g14370	189.2988	96.42818	47.90854	59.05371	23.10151	18.14744	20.80058	6.467687	36.34462	14.55276
Cs5g14420	7.875457	9.482831	8.052086	5.35908	12.41161	6.722685	5.471444	5.65076	4.464533	2.870293
Cs5g14450	6.888405	7.731418	5.361091	5.388272	1.94107	1.837555	0.974178	1.578583	0.230622	0.079345
Cs5g14480	4.300371	1.027186	1.339391	1.284758	0.82045	1.740132	0.562708	0.20206	4.169057	2.052746
Cs5g14550	101.8823	140.8203	56.54766	103.0656	46.03191	32.13431	36.19515	62.51206	11.13062	14.19181
Cs5g14870	0.414526	0.522776	0.045061	0.290668	0.908289	0.313495	0.738751	1.737661	0.33059	0.351909
Cs5g14880	239.2884	354.1164	270.9234	422.0222	420.4901	276.5087	641.2135	715.7471	394.545	300.2526
Cs5g14960	1.250592	1.049525	0.995698	0.91999	1.143963	1.101967	0.649876	0.742752	0.774344	0.286255
Cs5g15000	139.6138	188.4835	49.02706	110.1117	4.703761	5.213191	2.373042	5.735033	3.247267	3.408559
Cs5g15080	1.71169	2.309859	2.695199	2.539396	1.684816	2.764628	1.041542	3.038425	0.694265	0.667124
Cs5g15090	1.879857	0.611705	1.47613	0.615799	0.833299	1.393969	1.746162	1.230929	2.256666	0.377254
Cs5g15130	27.77	17.87899	40.42803	33.20026	31.23348	50.79193	25.51016	56.34426	4.094171	7.537904
Cs5g15150	61.21453	60.05239	65.35555	62.86016	37.73757	33.88465	26.74468	57.57887	0.561255	1.718134
Cs5g15170	49.33355	54.70071	70.68637	78.77457	57.60862	49.77434	160.7314	143.6928	186.7358	190.5647
Cs5g15180	1.374849	1.159952	2.284271	1.088139	1.501244	1.10893	0.575905	1.799025	0.843003	1.040135
Cs5g15210	3.151154	2.396311	0.844204	0.94708	0.439631	1.077893	0.329298	0.721514	0.116796	0.261833
Cs5g15345	2.051932	2.166661	1.600906	0.816072	0.590814	0.652849	0.111801	1.393767	0	0
Cs5g15350	5.948417	3.807903	5.593921	9.293719	0.893872	3.557574	1.136693	2.535424	0.306114	0.131014

Cs5g15420	23.76989	34.27921	32.33418	40.64789	18.49005	18.22191	28.10686	49.10964	21.09683	66.05702
Cs5g15450	1086.152	1483.192	460.9648	755.8914	144.5669	211.1799	83.88186	268.9572	87.39157	171.5285
Cs5g15460	24.54932	13.90291	16.2188	19.63871	12.62046	13.21123	13.14508	24.50367	14.60467	17.94408
Cs5g15510	10.5916	9.404885	7.923274	7.926067	11.26322	15.62869	17.65327	12.2418	90.63536	38.33198
Cs5g15530	3.987913	5.035195	5.775289	8.874029	1.566185	5.127394	0.346456	1.554325	0.735094	0.282701
Cs5g15860	1.261088	1.600995	3.113059	3.723411	1.790954	4.056715	0.608322	2.931696	1.012551	0.899926
Cs5g15870	0.552966	0.412365	1.110539	0.955696	1.798808	2.871069	3.091147	2.896425	2.977957	2.549403
Cs5g15880	14.16033	11.57769	18.22007	11.97775	20.77297	28.46752	21.62655	22.62213	42.56474	15.20997
Cs5g15940	2.758501	3.124494	6.982582	8.092045	21.70191	11.45839	11.5361	15.51782	0.601439	0.429917
Cs5g16180	82.57933	106.936	86.36561	104.3729	13.00848	21.96956	33.28701	73.18024	29.62196	87.57184
Cs5g16285	5.824028	6.367337	7.281417	6.567264	7.237643	8.736832	2.918949	7.208349	7.128706	3.674915
Cs5g16310	196.7313	84.62576	114.4875	114.7049	159.0734	199.2729	64.68205	34.13853	6.885726	4.059165
Cs5g16340	33.6304	24.72721	15.83416	17.71852	21.07014	25.7975	20.02351	12.83178	24.30846	6.877076
Cs5g16360	3.124874	1.657117	1.715736	1.583946	22.99642	16.18191	10.07212	10.69693	10.49173	5.360963
Cs5g16540	35.66945	69.93158	8.640836	18.78002	7.262551	4.573506	13.31977	24.9434	5.371425	3.78246
Cs5g16580	0.404599	0.457972	2.051391	0.939838	1.163135	0.673043	0.447796	0.906385	0.965983	0.40199
Cs5g16700	1.341707	1.594297	1.129578	1.322972	1.152132	0.899623	0.542394	1.213291	0.448518	1.039957
Cs5g16770	3953.739	5450.129	643.9309	1971.583	100.9131	125.5398	12.56323	12.42355	4.920408	5.909956
Cs5g16780	2981.972	3881.815	599.5303	1773.847	124.2182	161.8111	13.61072	24.924	4.62846	5.380816
Cs5g16830	204.303	176.9705	306.1087	377.3051	98.44319	82.12259	55.84918	12.00233	10.00958	2.746573
Cs5g16840	6.140661	7.830451	6.080692	10.11144	1.799053	1.581421	0.891462	1.223505	3.303713	1.443859
Cs5g16850	11951.09	14555.23	3559.62	8317.964	680.2348	1073.388	148.0952	324.5608	82.79006	74.78998
Cs5g16860	185.6007	203.072	247.2489	410.7284	175.3442	163.6886	246.368	54.53576	167.3719	78.50339
Cs5g16890	0.2486	0.196897	0.504835	0.42386	0.446603	0.539702	0.974138	0.33689	0.688827	0.716073
Cs5g16920	1152.792	1696.982	44.93334	284.7821	4.39937	9.404403	1.505147	2.115677	0.585127	0.832116
Cs5g17000	68.00709	50.14424	52.30227	43.76583	89.97644	45.93676	72.51081	57.7707	31.06307	17.5021
Cs5g17070	16.66834	20.43866	29.50861	26.05731	34.22706	32.35555	23.65642	67.15682	22.79878	32.98049
Cs5g17130	0.172224	0.157791	0.581409	0.222312	0.77081	1.733675	0.897436	1.743067	0.186119	0.487053
Cs5g17210	0.257025	0.24523	1.279585	0.942081	2.311387	1.725436	4.027137	6.815241	0.601007	6.271708
Cs5g17260	23.05802	35.9146	7.715169	8.600693	3.903056	2.715434	0.710787	2.480425	1.822956	3.433014
Cs5g17275	3.164034	4.22492	3.823414	3.525015	2.247375	3.64613	0.855483	3.671819	3.07061	4.163764
Cs5g17320	6.198168	7.628431	9.882923	9.410321	12.1194	12.82558	7.877644	16.93178	10.36331	14.88327

Cs5g17380	0.044231	0.195535	0.38601	0.336288	1.458836	0.694026	0.257514	1.356816	0.907307	3.17893
Cs5g17425	1.281517	1.458982	1.574138	2.310724	1.460688	1.608721	1.033222	2.679967	1.2183	1.994093
Cs5g17510	2.090602	3.306781	3.434472	3.946776	0.382681	1.2453	1.159786	1.823786	0.051614	0.363011
Cs5g17520	9.239684	17.46183	2.215599	6.355758	1.843246	1.464121	0.673319	1.107065	0.34314	0.152115
Cs5g17530	13.49792	11.27157	14.33946	7.928673	8.569751	6.066642	3.313231	3.800505	0.709488	1.25291
Cs5g17880	24.86288	22.95111	40.62993	75.94475	25.47941	40.6929	48.34716	31.41772	304.3819	237.9589
Cs5g17900	17.11637	14.53567	31.0098	62.2166	22.72962	40.70259	44.0422	20.34975	399.2539	269.7088
Cs5g17920	38.75426	28.03888	58.99988	120.5529	38.65383	81.57122	88.54966	36.56417	722.2006	602.3232
Cs5g17930	3.806118	4.173771	4.514417	5.920472	3.778695	3.912284	4.983059	4.960731	4.114931	11.69967
Cs5g17935	1.063578	1.200795	1.179832	1.224418	1.428121	1.769855	0.755233	1.693645	1.806732	1.415234
Cs5g18010	1.335514	2.106093	0.819743	1.215209	12.82083	6.521275	8.26303	4.016099	9.419633	41.05475
Cs5g18050	1.701012	4.210015	0.990728	1.409773	2.025566	1.275295	1.287501	1.241745	1.647699	5.143735
Cs5g18130	0.715692	0.819593	0.691306	0.617789	0.752437	0.927626	0.293337	1.475571	0.622913	0.365604
Cs5g18140	7.802994	19.31314	1.221466	4.114108	0.082138	0.084723	0.172063	0.15553	0.123745	0.145573
Cs5g18190	0.002052	0	0.009098	0	0	0.000874	0	0.029726	0.21216	1.105368
Cs5g18210	28.87519	34.24326	21.92401	24.94031	15.51387	14.96459	11.6346	18.55716	5.812172	5.458823
Cs5g18230	35.30384	40.28463	71.86863	108.2972	14.29608	65.0156	29.42922	202.1074	91.01856	178.4621
Cs5g18280	8.771734	10.85276	10.63883	12.16033	3.988061	6.768684	2.268709	7.507046	0.368731	0.2383
Cs5g18300	2.101721	3.379294	6.002257	10.35123	0.613492	1.990085	0.236352	1.163496	0.034022	0.117405
Cs5g18320	2.630402	2.061271	3.648669	3.987924	1.100687	2.799869	0.985505	1.766508	1.260346	0.916215
Cs5g18330	3.340883	5.134326	14.78751	24.35956	1.237531	4.993874	0.28282	2.421695	0.107201	0.077202
Cs5g18410	5.222426	5.959344	5.859775	4.959907	6.082509	6.790034	3.172109	7.330513	3.991999	2.56814
Cs5g18420	0.7277	0.530621	1.535421	1.37288	1.637489	1.457428	1.501891	2.597623	5.728628	5.662247
Cs5g18450	0.569498	0.453397	1.502069	0.929561	1.274645	1.065393	1.238763	1.653542	1.215442	1.270097
Cs5g18500	2.170254	4.147553	3.892347	3.53448	3.92198	4.652527	14.40598	29.66325	2.039117	3.6981
Cs5g18570	7.472674	5.305204	16.15913	11.81167	26.40387	18.85581	15.74174	14.48874	1.807521	3.065055
Cs5g18580	8.820156	4.663365	30.99647	19.65064	40.37751	50.72254	18.4177	15.88938	9.202581	10.44324
Cs5g18620	24.86764	112.3162	3.33776	3.056218	4.743282	6.231371	1.693059	6.464056	2.320669	1.370651
Cs5g18660	17.81825	19.93911	16.51919	21.14716	12.54882	11.08264	6.951137	3.536889	0.728917	0.366264
Cs5g18890	6.042529	6.445191	10.24517	8.33374	9.342972	13.48018	5.456845	11.40103	12.77658	14.60454
Cs5g18910	1.05801	0.390778	0.900586	0.868781	1.144269	1.238819	0.345127	0.809541	1.013296	0.679072
Cs5g18930	192.018	138.7172	389.5633	247.5362	294.471	435.8181	7.173934	60.76256	0.391	0.514495

Cs5g19020	9.49318	9.48701	31.87978	25.30424	37.93629	21.35034	56.00386	61.27805	28.35158	42.52047
Cs5g19060	0.074161	0.304804	1.796171	1.885191	1.829574	0.945126	14.10229	32.62585	16.74534	26.57818
Cs5g19110	17.85167	16.22363	9.13591	9.070409	9.435073	7.060158	5.205168	4.741381	0.550316	0.209845
Cs5g19140	4.372362	2.171381	1.799903	1.151836	2.985795	1.445602	1.864294	1.819423	0.216228	0.055188
Cs5g19160	0.025274	0.165639	0.465544	0.385397	0.211343	0.08762	0.250486	1.718625	0.745248	1.316194
Cs5g19220	0.729199	0.795353	1.329311	1.310933	0.242502	0.485303	0.507028	1.57662	0.652698	0.813007
Cs5g19240	0.806071	1.247984	4.737282	6.275218	0.249266	1.071594	0.342174	3.304793	0.033763	0.304544
Cs5g19310	7.313803	9.020046	13.78923	15.84235	4.602551	6.004772	4.70494	12.60235	6.728758	13.73565
Cs5g19315	2.09427	2.423069	2.298517	2.568213	1.685489	1.522364	0.580691	2.209263	1.249173	1.630302
Cs5g19370	0.492579	0.338198	1.160258	0.876886	1.500464	2.919745	0.280399	1.001546	0.190436	0.05948
Cs5g19400	18.93391	19.08842	26.70229	28.9459	5.22965	12.34874	5.996834	16.17824	12.46417	14.03682
Cs5g19460	3.436826	2.587523	8.345502	5.38412	1.294705	2.238074	0.653052	2.01294	0.475727	0.763698
Cs5g19500	0.485659	0.548385	0.701442	1.011347	0.255553	0.702997	0.152671	0.963296	0.895797	1.774902
Cs5g19570	0.740554	0.716559	1.336471	1.658764	0.992506	1.653469	1.069441	2.462868	3.526664	3.820258
Cs5g19600	0.858491	0.552441	0.256683	0.630263	0.028285	0	0.255632	2.625516	0	0
Cs5g19640	73.24032	83.50692	168.3215	169.2808	259.8665	217.1277	127.8324	181.0643	275.8721	261.4963
Cs5g19695	3.409872	4.09737	1.644405	2.209589	1.11487	1.021501	0.809348	1.421919	0.261335	0.73231
Cs5g19705	2.199051	2.405862	4.866106	4.214398	5.324447	10.00794	4.023537	10.2587	15.34027	14.46897
Cs5g19820	7.317534	7.489557	7.003303	7.038948	6.828453	6.440777	3.822159	8.858211	4.972094	6.112551
Cs5g19840	1.776171	1.347672	3.541133	3.624831	1.342462	4.764606	1.029717	2.748093	0.942298	3.682909
Cs5g19880	2.273003	2.866365	3.096573	3.343851	1.141145	1.792497	1.721834	3.380299	5.707722	13.1799
Cs5g19920	1.410317	1.334148	1.59078	1.294806	1.406461	1.33454	0.668088	2.323364	1.19294	0.70042
Cs5g19930	3.352223	3.032616	5.313825	3.900052	3.756632	3.823748	0.659572	2.067556	1.128225	2.76167
Cs5g19990	27.06202	29.47527	70.52241	55.29936	22.9303	42.83867	7.690693	7.525772	0.433146	0.190952
Cs5g20100	0.621804	0.781604	0.753533	0.25823	0.109939	0.084497	0	0.128576	0.082047	0
Cs5g20340	1.416145	1.297467	2.32173	2.259922	0.593743	0.802143	0.31767	2.07771	0.136443	1.269153
Cs5g20410	0.690785	0.494292	0.73947	0.67718	0.779304	0.889165	1.263304	1.136469	2.331394	0.981057
Cs5g20420	60.11664	68.58385	77.26567	65.75127	145.2408	100.655	268.0234	200.7346	213.2388	168.9394
Cs5g20465	6.099276	5.645091	7.74532	6.88616	2.494853	6.394233	2.177932	6.876609	7.506157	9.756184
Cs5g20500	7.897061	7.434041	7.611798	7.694811	2.347424	2.667262	4.221445	3.980133	2.039413	4.125798
Cs5g20520	27.06389	19.29572	12.36654	23.3974	11.23734	19.68273	34.9799	22.11233	66.61969	122.7758
Cs5g20550	1.992832	2.445276	1.720281	1.966667	0.632129	0.914514	0.316954	1.139904	1.057603	1.39692

Cs5g20600	12.77456	5.940138	14.99821	15.83398	8.141787	12.61517	15.56748	19.30288	40.14041	42.06013
Cs5g20630	39.0122	23.83673	36.34494	18.98365	50.14818	44.45301	9.618821	8.586958	2.160515	1.174218
Cs5g20660	64.03373	49.62334	54.58177	53.69585	114.0437	129.8429	140.2707	58.40327	98.55199	65.7169
Cs5g20670	6.723998	5.574823	8.681763	8.367023	14.10512	16.81863	23.18026	12.78641	15.67627	14.0841
Cs5g20790	29.50864	34.42395	26.02317	32.34336	17.32683	20.56409	13.95968	44.29543	7.414256	18.67274
Cs5g20910	50.05938	94.46377	79.80651	114.429	166.9814	123.1641	174.2226	158.2825	277.8921	160.0694
Cs5g20940	5.472193	5.491847	5.902043	7.161325	2.256331	1.792595	1.512745	1.80143	0.811504	1.125865
Cs5g21025	2.45599	1.798566	2.385971	2.155702	0.726124	1.822054	1.426621	1.276018	0.953416	2.287113
Cs5g21050	2.667521	2.275148	2.58889	2.404324	1.629913	1.433156	0.931818	1.159546	0.823808	1.111917
Cs5g21160	34.92918	36.42358	23.99489	24.98901	15.416	22.16451	7.858146	14.64971	15.2061	15.63397
Cs5g21190	0.100616	0.144217	0.208208	0.190926	1.027879	0.692512	3.252855	6.387452	28.6973	16.18257
Cs5g21240	1.66583	0.86306	3.62652	2.856161	5.220287	5.233643	4.303821	3.159723	2.818509	3.304791
Cs5g21250	0.045229	0.085627	0.211686	0.215552	0.73004	0.719906	3.677523	5.75931	21.69683	11.95408
Cs5g21670	0	0.497471	0.543168	0.416857	1.009148	0.630554	1.947842	1.437881	0	0
Cs5g21690	1.74429	4.486144	0.715872	1.711433	70.59569	61.41313	31.44506	54.41955	73.82928	48.14403
Cs5g21700	1.868885	4.217885	0.880519	0.953676	65.29096	48.80263	29.82795	42.8206	60.32481	28.14468
Cs5g21760	4.815799	3.752838	7.967805	6.707697	1.891082	2.215685	2.066235	1.87285	0.311059	0.296488
Cs5g21775	2.793705	3.255206	2.62575	2.778316	2.256045	3.914206	1.445364	4.245207	2.816491	3.845666
Cs5g21780	2.758416	4.005743	12.70964	9.753976	7.978371	9.461897	4.605236	9.494053	3.133954	5.484169
Cs5g21790	16.16048	10.94075	11.89602	15.24759	31.21443	34.09189	32.75902	30.94531	16.76047	26.52097
Cs5g21860	9.419478	11.48021	31.10588	44.66812	30.8219	33.26504	30.53017	88.88782	8.478297	54.08566
Cs5g21890	2.847586	1.715269	2.73233	2.924135	1.992701	4.188267	0.609863	2.965149	0.852568	1.346482
Cs5g21910	1.354726	2.507082	4.398393	4.883716	0.615983	1.458879	2.513871	6.175966	0.337345	1.956072
Cs5g21920	1.349782	3.521989	7.794277	8.672826	0.79985	2.465865	2.397328	7.636292	0.367407	2.306408
Cs5g22020	3.300811	3.184477	4.320815	3.030122	1.679126	2.836304	1.003666	4.827831	1.16555	1.536516
Cs5g22060	0.036025	0.168815	0.644441	0.330658	1.010204	0.472837	1.562659	3.529739	8.079945	12.2764
Cs5g22080	8.55776	2.949274	2.510779	2.846299	7.682006	7.075565	11.66855	28.2053	81.88016	64.12127
Cs5g22170	69.15395	70.61052	89.036	92.79686	19.02565	37.07387	37.99122	74.41437	48.71421	169.7276
Cs5g22210	10.47284	9.628716	27.50461	27.39132	8.524524	14.57612	18.18978	52.33389	16.24542	54.21367
Cs5g22260	1.259561	2.001928	6.993752	6.036323	0.261119	0.668313	0.530044	2.134128	0.075117	0.457126
Cs5g22390	2.551171	1.343376	7.012448	5.338574	3.979753	6.756661	1.263975	2.063447	0.716906	0.936783
Cs5g22400	1.11978	0.443784	3.242828	2.043654	2.239914	2.978559	0.934348	0.974689	0.232859	0.285539

Cs5g22460	1.890745	1.784977	3.219818	3.258865	0.613311	1.062697	0.605849	1.758868	0.646008	3.134271
Cs5g22660	3.130663	9.616245	0.413853	0.883329	0.169785	0.175262	0.09942	0.651107	0.168964	0.132259
Cs5g22690	6.796154	6.515427	4.345369	4.381395	3.087743	4.744587	4.319478	3.125626	1.427169	0.481537
Cs5g22710	11.08177	9.915668	9.664367	7.266822	4.677252	6.079804	2.426153	3.549826	0.535678	0.269352
Cs5g22720	1.781826	3.185165	2.842085	3.265192	1.438727	2.254416	0.251748	3.195064	0.311188	0.37764
Cs5g22725	1.31218	1.300835	1.304821	1.60388	1.77327	1.592906	1.217724	3.461732	2.114139	1.587666
Cs5g22800	13.65244	11.5066	14.30366	11.3399	9.254519	11.74149	2.51224	4.115538	1.526982	0.586955
Cs5g22810	9.896027	11.19212	4.757473	6.13364	1.440637	1.677527	7.607872	19.48766	3.429543	16.30085
Cs5g22840	8.475563	9.900281	11.81219	11.89934	10.19311	11.48591	4.907441	11.7108	5.844139	5.840098
Cs5g22880	1.573428	1.8074	3.15912	3.382827	1.156375	2.378928	0.779251	2.924399	9.718776	23.63126
Cs5g22890	2.991084	2.679369	4.631828	4.60812	1.811374	4.221861	1.178805	5.143494	17.73715	41.94256
Cs5g22950	10.38177	28.92364	2.716592	7.829947	26.6827	16.81995	25.46552	40.40059	5.919417	14.18629
Cs5g22980	10.75041	51.62624	0.54328	0.214564	0.131608	0.068362	0.080136	0.660665	0.028217	0.117168
Cs5g23050	9.648677	9.47896	10.10561	13.20598	5.275683	8.386547	3.321708	8.136214	2.001541	4.250466
Cs5g23080	0.080057	0.038998	0.203085	0.090267	0.358021	0.56084	0.216255	0.547464	0.355456	1.53654
Cs5g23090	41.65079	32.81634	145.8282	93.18804	105.6775	101.2426	40.22863	115.2249	31.22806	50.98332
Cs5g23130	0.503776	0.342536	1.084966	0.9077	0.896721	3.429736	2.861367	2.575847	10.91336	9.378327
Cs5g23170	3.528068	3.252931	4.061985	3.328856	2.195011	4.490398	4.517836	5.244411	9.522132	5.669304
Cs5g23240	4.94467	5.641003	10.32343	10.25177	5.043993	8.059695	2.066687	8.078685	1.445414	4.027858
Cs5g23250	2.870169	3.369731	2.419463	2.827561	1.4856	2.049304	0.780384	2.426838	4.972334	8.342231
Cs5g23420	0.303905	0.371569	0.491706	0.499572	0.363435	0.631012	0.30163	0.79002	0.995697	0.889938
Cs5g23480	3.215365	4.198976	5.352582	4.474873	4.034594	4.365343	2.133376	9.654363	3.98936	3.731055
Cs5g23490	7.81262	7.684372	11.72644	10.75466	8.345899	9.827708	5.874699	20.90453	6.62291	11.09127
Cs5g23510	117.7112	109.8191	98.80279	127.3782	83.07731	105.4689	40.72029	58.94224	19.42395	4.858204
Cs5g23540	13.52868	12.99697	11.81156	16.54525	9.14618	13.82667	5.232754	6.833741	2.059053	0.843133
Cs5g23630	5.714806	7.357848	2.267218	3.32197	0.986047	1.418818	0.850691	2.797774	1.193455	2.2809
Cs5g23770	3.493163	3.683591	3.979376	3.791406	3.913783	5.250444	1.726147	4.724848	4.793989	2.864553
Cs5g23950	0.586632	0.692082	1.963812	2.581066	1.563127	2.210946	3.162756	9.16314	7.62374	16.81382
Cs5g23960	0.619542	0.689935	1.957166	2.84196	1.111317	1.661102	3.059624	6.010075	5.013693	11.74586
Cs5g24105	7.541085	3.779096	34.31589	23.86572	70.47891	39.62521	46.31184	82.41391	28.8547	30.89109
Cs5g24130	2.245938	1.283798	5.964083	4.28178	5.211651	4.248712	1.793336	2.618869	4.241578	1.542847
Cs5g24140	4.151688	2.721736	12.29689	10.70276	8.826166	10.13753	4.284454	6.67794	9.430273	5.119341

Cs5g24170	35.78545	24.27579	31.09222	29.03443	31.12432	38.00263	50.96701	34.87029	30.29976	11.20411
Cs5g24220	0.78422	1.009105	1.226919	1.31446	0.900556	1.58353	0.874345	2.248439	2.451992	1.786537
Cs5g24240	0.938086	0.681937	1.508684	1.30801	0.319046	0.646094	1.870147	4.498464	1.506943	8.28975
Cs5g24300	15.40581	13.03539	15.16413	13.3415	11.71943	19.0823	6.140758	13.16661	35.53247	18.89078
Cs5g24310	4.811261	7.370321	4.054159	4.435378	9.197159	8.48852	13.50538	5.559458	8.993904	13.01196
Cs5g24350	31.2598	32.84327	48.20409	47.70702	24.96716	41.1563	16.74679	35.37342	25.83785	50.64442
Cs5g24460	5.828967	3.976908	11.76191	11.86819	14.85318	14.0582	11.70392	8.079312	5.713514	1.621364
Cs5g24520	1.716335	1.289437	1.487952	1.815743	2.842115	3.847558	4.489073	9.590018	1.519724	1.9225
Cs5g24590	19.23738	20.62716	16.54395	17.04077	7.695102	12.56094	3.48831	4.964538	1.717565	2.257423
Cs5g24610	0.628363	0.401592	0.93094	0.778	3.709319	2.715034	1.883507	1.751522	1.333088	3.548818
Cs5g24640	0.051034	0.13789	0.533102	0.296647	1.937131	0.823455	0.56308	0.377676	0.05254	0.182645
Cs5g24680	406.0839	419.8889	190.708	233.8058	111.9866	133.449	89.76996	135.6994	56.89496	60.22945
Cs5g24700	0.691209	0.878102	0.308242	0.341239	0.895965	1.166189	1.292829	6.64728	1.042879	2.111391
Cs5g24925	0.225998	0.194988	0.458949	0.374163	0.23649	0.482864	0.05064	0.429361	0.197035	1.031467
Cs5g24930	1.018851	0.89581	3.889752	2.884505	3.608524	4.581778	5.073354	7.359864	3.300713	6.388751
Cs5g24940	0.948678	1.219804	7.778578	6.547088	10.42343	12.00141	13.01608	25.17755	14.66905	27.16334
Cs5g24950	11.81255	10.25326	33.1993	24.00071	32.12902	33.54593	33.47968	62.22356	11.28502	22.30614
Cs5g24960	0.550822	0.575175	1.443089	1.333528	1.771627	2.178362	2.571437	4.072777	4.228717	7.924872
Cs5g24980	5.261634	5.713554	1.360176	1.381421	0.995354	0.769972	0.207441	1.00289	2.014615	3.373341
Cs5g24990	22.14661	23.03015	6.660282	5.979688	3.626145	2.965665	0.765852	4.834572	8.023589	15.08902
Cs5g25000	1.705731	1.4799	5.418133	4.257758	7.581224	7.403594	10.04868	12.58016	5.364879	9.145666
Cs5g25020	3.483329	2.328845	9.109509	6.760161	16.43558	16.96531	29.47535	9.611895	14.9554	10.80831
Cs5g25090	29.91711	38.04398	22.07615	30.60894	5.155516	8.055541	7.505905	31.43745	9.516165	48.94665
Cs5g25140	12.4803	16.81413	12.25999	16.71067	7.079417	9.823418	7.304057	8.272731	1.178601	1.506222
Cs5g25320	6.422333	8.591839	5.085535	6.678887	2.818416	3.660885	1.601571	2.47443	1.856909	3.134069
Cs5g25350	4.610102	7.287458	5.154305	5.065993	4.53234	4.990986	2.214614	5.680196	3.207591	2.188905
Cs5g25370	1.12503	4.866619	0.39684	0.446293	0.194155	0.133659	0.29108	0.136889	0.141565	0.0425
Cs5g25380	9.763353	13.95603	7.423888	8.548953	7.027927	9.15929	3.160151	7.823478	2.320759	4.110435
Cs5g25390	5.372276	6.455808	3.191326	3.651106	2.246378	2.893473	0.797007	2.226342	0.467607	1.07547
Cs5g25420	5.407226	4.400728	4.803711	4.708977	1.558987	1.962238	0.693525	2.45025	0.013759	0.029906
Cs5g25500	3.212117	4.620655	2.972615	4.671235	1.710532	2.732735	1.227195	3.87069	1.70108	3.05733
Cs5g25590	425.8592	328.2979	67.65567	133.3394	1.06515	4.451932	0.247103	3.049499	2.911956	2.056255

Cs5g25680	14610.11	15737.88	4005.75	7076.141	1467.207	2798.698	429.0773	961.7348	33.05861	27.61673
Cs5g25690	13378.59	14218.04	3618.9	6276.177	1262.346	2400.108	406.2072	808.6178	16.02773	14.43913
Cs5g25740	3.311321	4.087205	1.558807	2.279046	1.278148	1.940852	4.423662	6.601122	287.1467	220.2554
Cs5g25760	2.612841	3.127133	0.991641	1.790983	1.332394	1.752059	1.007907	3.658045	39.02977	104.7499
Cs5g25790	1.904793	2.42364	6.92834	11.72124	9.39294	10.38931	7.122978	20.2672	6.915924	9.009907
Cs5g25820	1.117311	1.555952	5.41735	7.307211	7.060692	7.008925	6.402014	18.15018	2.721451	4.028017
Cs5g25840	31.8607	19.64001	22.49721	20.22351	33.05929	20.33985	20.25329	12.66968	22.43365	5.83215
Cs5g25860	3.912686	3.704782	7.679445	7.285345	3.756218	4.268073	3.099309	1.337047	1.078656	0.533938
Cs5g25880	0.282631	0.083118	0.694547	0.386561	2.633375	2.754879	4.036943	2.944161	2.673699	1.693539
Cs5g25900	3.201729	2.467609	6.935159	5.950645	24.70785	28.27896	50.54957	38.13563	62.50886	123.742
Cs5g25920	3.630301	2.119279	4.902665	3.688728	16.33152	15.9005	24.92939	15.31718	26.28447	19.83212
Cs5g25930	2.178666	1.805853	9.252398	5.85475	33.85979	36.70896	54.8523	37.61105	31.54777	42.76419
Cs5g25950	0.14131	0.139515	0.097946	0.067136	0.625329	0.761832	2.109712	1.949275	8.846686	17.60921
Cs5g25970	1.254645	3.020659	0.184531	0.220706	0.180956	0.064425	0.0762	0	0.120917	0.079863
Cs5g26150	5.627333	5.976319	4.174204	5.119327	1.431244	1.542261	2.992666	4.911097	5.197707	14.34295
Cs5g26220	303.7003	289.8477	220.7035	155.7807	204.3183	85.21563	99.84902	92.12901	72.61709	72.43877
Cs5g26270	1.078792	0.406213	0.325341	0.348537	0.174143	0.349808	0.143291	0.11839	1.012799	0.709853
Cs5g26290	0.17119	0.173409	0.191863	0.221439	0.264148	0.180715	0.251561	1.185322	0.227973	0.410547
Cs5g26450	41.24725	32.35827	27.22452	35.34656	6.565579	10.83402	12.17283	17.54933	28.71467	28.08767
Cs5g26470	60.67573	60.6553	34.64903	34.67933	15.74052	22.57062	15.67209	20.53105	5.362978	2.362532
Cs5g26480	5.251652	4.982116	4.057692	4.856284	2.731325	3.054098	1.708544	2.367847	0.560653	0.819899
Cs5g26530	0.469775	0.654016	0.583924	0.37527	0.839423	0.34078	0.139965	0.19516	0.280254	0.074991
Cs5g26570	0.660966	0.556767	0.415087	0.353054	0.439324	0.549302	1.003723	0.392139	0.328754	0.199678
Cs5g26580	10.4662	9.427331	14.41516	19.63073	5.392732	8.317967	9.144638	14.63372	4.511545	12.74482
Cs5g26670	4.610643	7.696181	5.770295	8.72251	1.625446	4.495813	3.051319	15.30074	3.755366	12.25208
Cs5g26740	208.1416	232.0998	251.2752	244.21	162.0859	137.518	83.36131	101.4945	57.58061	57.4093
Cs5g26750	20.66939	25.6733	34.19567	39.85182	29.57457	34.14025	17.93437	44.44545	25.24062	41.96671
Cs5g26820	3.84889	3.443361	4.472441	4.087952	3.249914	2.566361	1.687017	4.070926	0.780533	1.783014
Cs5g26850	16.0884	18.29497	19.35637	16.65945	14.5389	22.44014	6.364642	39.04925	42.35085	68.57569
Cs5g27130	5.679003	3.648057	5.668152	3.843792	7.755178	5.867683	14.27779	23.56138	25.47859	19.48966
Cs5g27140	9.071913	9.736635	6.437406	6.66316	3.786901	4.837369	2.897839	5.301702	0.34585	1.122895
Cs5g27180	11.06682	10.50271	6.955691	6.607917	4.746575	5.9489	3.894256	3.001101	3.284646	2.176589

Cs5g27290	17.23335	16.31839	11.28156	11.75198	6.248055	7.361138	3.808791	7.045203	0.445564	0.703778
Cs5g27310	6.665418	5.001543	7.513878	7.077266	12.41077	10.43307	19.61795	14.86134	11.5524	10.76932
Cs5g27370	111.4017	69.10684	29.91875	36.85236	19.55381	10.47392	5.258566	2.81168	0.582691	0.251426
Cs5g27440	7.879053	4.570846	5.482131	6.300969	3.390491	3.902946	6.247749	2.985095	0.107763	0.057808
Cs5g27460	0.102594	0.056474	0.159072	0.206174	1.094684	1.024187	1.181776	1.432384	0.762581	1.376278
Cs5g27560	71.64725	72.35054	36.84191	34.08812	15.04132	26.32607	4.664877	32.63391	36.2962	60.88603
Cs5g27570	493.5361	610.4603	1025.066	1027.286	1044.913	1427.505	1408.606	1031.03	2261.166	1669.433
Cs5g27580	59.95099	90.35724	114.4754	116.9544	391.1308	281.1401	486.1055	223.006	334.7474	278.6064
Cs5g27590	27.66815	42.76918	56.02251	59.67574	176.4019	137.5529	208.6483	113.2199	161.7231	152.6136
Cs5g27600	33.10582	50.01628	66.8489	70.66794	200.9938	160.8355	237.6413	130.8253	186.2786	176.745
Cs5g27610	2.883088	3.285933	8.296052	7.618001	6.546737	6.107404	1.74283	2.186662	2.523111	4.317883
Cs5g27620	12.62558	12.73661	7.663037	7.631608	8.770695	4.796096	3.252599	5.682761	8.742441	11.34624
Cs5g27650	108.0395	95.50943	64.96602	63.66194	21.7484	37.60091	29.26976	92.0042	58.78998	108.2639
Cs5g27670	17.59197	13.87877	11.61932	14.52825	10.80182	19.72156	8.215363	13.06701	9.131454	12.93172
Cs5g27720	27.6551	27.5948	36.57489	35.87687	40.79781	48.0058	71.21096	60.54317	85.63058	95.74672
Cs5g27740	12.84792	15.26845	22.11895	21.83459	13.55601	23.92805	9.553581	16.25372	2.431838	6.469217
Cs5g27880	0.865148	1.115373	4.383227	6.443611	4.227682	8.773656	7.613322	9.044226	5.551633	4.151964
Cs5g27895	4.681537	5.774865	7.264909	6.475723	4.029831	6.818479	1.622796	6.252131	2.989277	3.013825
Cs5g28000	6.439539	7.276412	11.34813	9.907451	10.8829	11.28802	5.767855	13.70263	5.212853	6.302622
Cs5g28050	64.57061	87.80999	95.68505	129.2233	31.96407	49.03607	61.15492	83.55991	100.8583	167.3849
Cs5g28220	0.134518	0.161002	0.294332	0.148174	0.60286	0.706813	0.977551	8.877087	4.600076	5.563122
Cs5g28250	8.574027	7.714361	18.23607	14.72025	14.02297	19.62816	4.30759	17.62971	4.26392	7.61111
Cs5g28260	13.21416	12.07602	19.64633	19.26146	11.01978	17.07337	8.064335	18.49054	9.713176	17.73028
Cs5g28330	65.42721	71.38584	28.81612	38.95968	3.371929	10.15523	12.89477	39.63629	0.341506	0.459227
Cs5g28400	4.297008	3.719259	6.389288	6.539911	6.357283	15.70953	8.513679	30.73811	61.42042	175.0814
Cs5g28450	4.101499	4.08973	7.603646	6.680752	4.845731	9.277726	2.049929	5.547427	3.01612	4.040689
Cs5g28460	11.12645	27.50178	1.699319	5.089611	1.696558	1.734954	0.372322	2.0638	0.351421	0.349054
Cs5g28540	0.107974	0.096505	0.325056	0.169869	0.754361	0.366667	0.77799	0.830471	0.879531	1.755375
Cs5g28550	0.828359	0.752818	1.774557	1.368556	4.158648	2.434735	3.268036	4.577941	6.855317	9.727053
Cs5g28560	77.38637	114.0768	49.34789	89.01334	9.934572	10.40401	6.080813	22.90644	1.997636	7.258175
Cs5g28610	2.984553	3.012426	6.811229	6.993075	5.591521	7.893664	3.625763	8.3215	1.696567	2.689625
Cs5g28640	4.23571	2.722358	5.178479	4.770117	3.106496	5.953202	2.122795	2.912366	2.31408	2.285944

Cs5g28650	1.028383	1.166676	1.265834	1.136742	1.112838	1.638312	2.18324	3.570359	2.805816	5.624999
Cs5g28655	8.213763	5.544596	13.10921	6.282547	12.03574	8.584683	5.382788	16.27876	7.322681	9.901897
Cs5g28680	8.091784	8.758537	9.313914	6.896099	7.787798	6.274509	1.281557	1.207114	0.024539	0.180967
Cs5g28710	15.14385	14.80563	40.11102	51.08295	69.94848	45.45298	72.92201	86.02132	123.7431	169.6367
Cs5g28780	11.15696	8.666468	11.98369	14.68218	24.14099	15.82594	30.94823	33.90636	15.54721	23.99376
Cs5g28840	5.406917	4.75956	2.90016	2.764143	2.135759	3.311381	0.331884	1.452409	0.233288	1.591835
Cs5g28950	9.184525	8.202226	13.33474	10.29837	40.8192	36.04355	28.6616	26.8818	43.65803	40.63827
Cs5g28990	6.978776	5.064659	4.863076	4.232672	7.814615	11.29792	7.821092	8.656904	86.84136	16.86025
Cs5g29020	39.08802	62.46732	45.04381	47.59096	16.52086	19.28204	30.38623	67.02598	30.2835	68.87622
Cs5g29040	17.62089	25.87917	8.128215	13.85998	8.866555	4.404413	6.835967	6.864424	0.532021	0.299195
Cs5g29100	10.58222	8.972081	15.08707	14.30674	22.28563	20.22198	27.06828	17.30411	36.96617	23.81588
Cs5g29180	1.72855	1.665563	1.669637	1.161211	0.737386	1.201921	0.589705	3.481899	0.446344	0.640063
Cs5g29210	1.106082	0.906814	4.064756	4.513585	0.858517	2.17466	1.221728	1.897675	0.451352	0.430471
Cs5g29340	16.9741	19.45294	18.12097	19.83989	8.287634	10.03227	4.4209	13.24662	5.20909	8.593311
Cs5g29360	3.744706	3.892326	5.370334	4.014579	9.306385	7.32953	6.338329	6.027218	4.420304	3.137699
Cs5g29580	17.18377	15.51573	18.32969	18.43272	18.19848	46.78853	14.04754	20.66584	32.30853	29.88539
Cs5g29600	11.63905	9.167492	11.59411	10.67573	23.11442	26.46908	24.61318	29.59684	46.1735	33.49048
Cs5g29670	78.00017	79.46639	38.30226	43.06865	23.10267	33.17968	30.35621	23.69021	34.87749	17.97847
Cs5g29830	16.29613	20.83028	38.04588	60.64596	10.53729	18.75889	25.02756	16.90957	64.27081	20.09584
Cs5g29860	25.65421	15.63223	54.34665	51.32021	51.19841	147.9125	117.9577	74.67923	646.5365	282.5483
Cs5g29870	35.91635	18.07845	18.3569	32.56742	3.297407	7.698364	14.60546	4.923651	2.484578	10.01727
Cs5g29900	0.597613	0.648388	0.132746	1.236877	0.020541	0.14065	0	0.434016	0.047939	0.161368
Cs5g29930	2.246988	5.272156	1.740023	1.322289	1.905577	3.130785	4.37095	10.4828	11.83683	44.24739
Cs5g29980	0.8584	0.379914	1.665361	1.174157	0.736896	1.405091	0.389749	0.393872	1.927707	1.930853
Cs5g30100	267.4082	269.7443	256.1197	285.2776	84.96832	99.53125	35.6137	66.33026	74.6624	83.76936
Cs5g30160	0.250504	0.233326	0.983301	1.232651	0.141562	0.388972	0.56728	1.715425	0.053323	0.179692
Cs5g30185	0.097636	0.072673	0.056111	0.026878	0.105312	0	0.301861	0.097754	5.29978	17.6147
Cs5g30290	6.395154	5.947176	7.459462	11.39467	6.170854	10.95172	4.417838	13.83818	2.340732	5.817443
Cs5g30320	101.2366	164.6566	24.13736	44.98593	6.109453	5.954052	5.527342	7.485264	4.009752	3.404466
Cs5g30350	0.146762	0.048808	0.205306	0.236504	0.150627	0.232918	0.739058	0.254487	6.461027	0.87344
Cs5g30390	10.0879	22.24237	5.553628	7.21395	3.139484	6.48834	2.691403	10.73943	0.484687	2.410856
Cs5g30500	90.9404	99.51834	90.12387	80.70235	59.72199	51.83981	30.87111	37.48445	17.51424	19.74097

Cs5g30520	4.712825	1.777244	2.020751	1.313329	7.125835	5.882189	7.644896	2.359249	6.90439	3.023565
Cs5g30530	18.38534	15.35768	17.29948	17.32773	9.885083	13.56692	4.729889	6.310016	5.571168	3.125525
Cs5g30590	30.96452	68.31427	28.51814	40.62228	40.71857	31.32214	65.74098	65.45847	35.41278	54.77342
Cs5g30610	1.011221	0.46949	1.934114	1.464381	0.421092	1.490512	0.214282	0.321966	0	0.048638
Cs5g30640	14.5287	14.59626	24.3954	22.09203	23.01134	22.90674	64.26194	31.08085	95.22659	42.95646
Cs5g30645	2.489979	2.656992	2.612151	3.336351	1.142214	1.058062	1.019553	2.357168	5.217956	3.119736
Cs5g30660	0.33774	0.358673	0.859163	0.55471	0.705092	0.77133	1.739563	0.669661	0.744693	0.879372
Cs5g30720	24.57464	27.56351	13.50166	16.83483	25.90046	26.60071	37.23964	48.86473	79.85172	106.1017
Cs5g30740	7.702171	8.22212	13.50312	12.12199	18.59137	22.19567	13.24973	38.40519	12.47798	17.609
Cs5g30790	27.22153	25.13	8.228428	10.58366	9.759717	14.24395	18.51336	16.73037	25.31822	37.14273
Cs5g30850	0.594654	0.879169	1.343694	1.174602	19.53875	15.36447	27.9856	18.97237	177.6253	160.3374
Cs5g30955	0.978739	1.545115	1.593881	1.208209	1.372969	1.961659	0.316648	1.35018	0.094972	0.317328
Cs5g31060	12.40912	12.73801	22.75187	19.73308	21.18276	19.5169	10.2545	35.20873	11.92403	40.79127
Cs5g31090	1.092025	1.240225	3.05446	2.782807	3.695611	1.016625	2.000361	1.537657	0.38852	0.810705
Cs5g31110	6.226193	5.437665	28.08952	21.45562	47.17164	50.84275	50.48881	57.45673	30.87414	40.71655
Cs5g31115	0.937475	0.424883	0.683531	0.716357	0.59089	0.981764	0.59187	1.581284	0	0.023258
Cs5g31180	37.07941	65.40396	12.53543	21.84989	10.01293	12.24231	1.003095	6.437434	1.473043	0.939164
Cs5g31280	11.50962	10.63847	4.991734	6.712995	4.054989	8.855724	1.380154	4.592617	0.698388	1.53504
Cs5g31320	12.91007	14.46552	10.16275	13.49441	6.196884	7.389398	3.91206	9.022598	4.824169	6.765189
Cs5g31420	1.454624	1.282186	4.055856	3.317384	2.50194	4.089489	1.966302	4.602125	0.956448	0.642322
Cs5g31500	65.82569	61.26428	137.0263	127.135	102.2805	84.72228	83.49307	64.58476	46.93229	38.94221
Cs5g31660	18.8749	15.6044	24.22992	23.63356	2.358932	5.48095	10.34805	15.2253	7.002199	20.01546
Cs5g31670	69.45215	14.80367	24.38362	12.0497	27.92099	8.049822	2.139301	2.494798	1.413312	2.062327
Cs5g31710	1.276173	3.645579	0.720574	0.768789	0.238061	0.051926	0.164769	0.2263	0.199046	0.326221
Cs5g31740	5.463258	5.273706	7.007002	6.090596	6.75473	8.735446	3.311377	7.039825	0.973253	1.388658
Cs5g31840	11.91766	14.90928	29.93607	27.37986	60.7809	45.30558	89.68628	46.65664	54.39763	53.99271
Cs5g31970	5.944385	5.025962	5.752399	5.76261	2.923805	1.98486	1.135338	3.238018	0.045361	0.222848
Cs5g32070	23.28144	24.94266	39.00321	37.53036	41.1906	51.23514	25.82134	60.41945	74.02799	67.79522
Cs5g32090	2.810422	2.838505	4.553895	4.193019	5.922938	7.343959	7.120438	8.587637	22.73696	14.14979
Cs5g32110	2.126807	1.517098	2.395987	4.214047	1.71606	5.834731	1.814469	0.682304	97.37519	42.4902
Cs5g32150	1.134681	2.136339	0.44668	1.11898	0.149954	0.244948	0.18871	0.94809	0.303703	0.461707
Cs5g32180	41.76013	40.66206	74.28318	60.70627	116.2664	88.60713	60.33815	73.6887	93.04091	24.91258

Cs5g32200	3.005943	3.384678	0.593427	1.725294	0	0.078441	0.032544	0.110285	0.04804	0.077762
Cs5g32270	48.02432	32.29391	29.13801	27.0361	9.978872	16.14016	3.181412	5.388495	0.802535	1.854371
Cs5g32290	5.216845	4.450905	2.424698	3.423073	3.290262	5.013728	8.945783	3.516083	12.35367	6.325827
Cs5g32300	2.941198	0.859967	2.025513	1.547552	3.201437	5.89787	9.990058	4.956723	75.43562	67.16264
Cs5g32310	1.913448	1.549125	1.773802	1.778712	1.120808	2.641047	1.734809	1.52393	4.004561	1.749826
Cs5g32360	1.635652	1.333526	3.499453	3.974354	1.38083	1.987335	1.812965	1.667123	0.353234	0.354358
Cs5g32370	8.159988	5.459045	17.44793	20.26119	4.275778	5.444354	2.710078	3.053928	1.204779	1.26467
Cs5g32400	1.087866	1.586523	2.827827	2.299632	4.717755	4.883784	6.54243	5.389631	13.74214	17.53589
Cs5g32420	28.25764	124.7184	5.316184	13.75019	2.275548	1.493062	2.504759	3.178823	1.384676	1.195483
Cs5g32440	14.72776	28.61725	12.74976	17.41508	11.24408	9.794158	18.10047	12.83709	11.66111	7.19874
Cs5g32450	6.208474	6.669564	10.35682	11.14175	12.88853	8.09218	3.257822	15.80867	3.875388	3.94744
Cs5g32510	13.84201	13.53205	22.92088	20.58236	30.45095	41.07151	18.95241	39.51571	42.2407	37.08991
Cs5g32675	9.463058	8.692219	7.807342	9.262297	6.025384	9.886646	4.171609	10.14462	17.75625	21.42647
Cs5g32740	6.638836	8.157152	13.24643	16.48456	14.8222	11.37142	40.49066	36.24402	160.7319	61.84578
Cs5g32840	7.958327	6.997541	13.99312	10.90798	13.24297	22.25286	6.518962	26.78233	1.654712	3.960832
Cs5g32870	0.389664	0.292557	3.758017	4.30396	10.8322	11.10425	8.112748	8.295409	12.67282	10.44468
Cs5g32910	17.8117	20.0918	19.77399	22.20933	20.11953	22.31215	8.031697	19.92499	14.9047	14.08733
Cs5g32930	1448.403	1315.085	783.1207	847.8151	1250.441	782.7573	720.3449	513.6152	935.0489	358.6983
Cs5g33055	8.155526	10.04768	7.221715	7.169579	4.64367	4.998085	1.037119	5.208832	1.968468	2.829055
Cs5g33170	37.09277	43.93418	67.80135	65.33376	86.97529	97.87264	78.31395	108.592	5.137471	4.439606
Cs5g33200	12.16998	18.4703	7.197041	7.123873	4.846313	3.972103	3.879052	9.341392	0.830777	7.119446
Cs5g33210	6.308677	7.159888	7.746928	8.250706	5.586753	7.09326	2.325464	7.140344	3.967795	6.050378
Cs5g33240	59.67785	52.14485	59.12804	60.14191	70.53771	72.54775	187.1033	76.62866	84.24382	98.4713
Cs5g33250	49.1947	50.86766	41.42238	36.37215	22.84383	18.28432	13.20533	40.90518	6.817955	12.47447
Cs5g33380	2.478168	4.470016	6.272009	5.833964	3.527064	4.983793	1.9398	6.105475	0.611048	0.904953
Cs5g33420	0.114062	0.189253	0.391488	0.578908	0.061975	0.526162	1.226455	7.675895	0.625109	1.81291
Cs5g33440	2.031271	1.758835	1.556775	1.300047	1.387186	1.385091	2.557256	3.539228	2.155251	6.690788
Cs5g33450	4.874488	2.605324	0.87671	1.259096	0.270278	0.327805	0.044587	0.18733	0.393732	0.352815
Cs5g33510	7.733512	7.895153	14.8331	14.75772	23.0437	25.8478	29.59891	32.64515	29.12828	40.38199
Cs5g33540	14.61801	14.63059	13.01764	14.99322	4.392618	9.306263	11.24373	27.90362	35.58087	24.05942
Cs5g33640	48.64081	73.62444	59.37231	76.81044	23.79797	34.25089	19.55442	60.82102	39.22805	105.3929
Cs5g33650	1.397099	1.41598	1.119973	1.476534	0.977668	1.58889	0.765379	1.705508	2.127694	2.115773

Cs5g33680	6.498903	5.955019	2.202354	3.144969	0.593049	0.833663	1.041546	2.93203	0.960164	2.45331
Cs5g33700	28.2072	20.98785	34.80852	31.18901	53.26309	66.62293	95.92694	127.4487	31.78731	27.67016
Cs5g33720	15.34656	19.91069	17.74706	16.28498	16.10066	8.529683	15.74312	26.49884	5.36881	12.51425
Cs5g33730	1.584037	2.442464	2.096298	2.122852	0.626868	0.541697	0.574673	1.656499	0.325409	1.008584
Cs5g33830	4.718527	5.222111	2.083808	2.659809	1.151084	1.321877	1.179618	1.6691	0.597214	0.601621
Cs5g33990	4.842592	3.774026	12.11268	9.034414	17.1515	11.6753	7.643692	10.58187	1.554962	1.893507
Cs5g34010	6.973534	8.154999	5.001354	4.721979	2.149492	1.655024	2.023406	1.991277	0.022535	0.06256
Cs5g34030	4.917494	4.946636	5.296442	5.92351	3.500863	5.439474	2.839964	5.912962	2.932063	4.08917
Cs5g34060	1.345193	1.678912	2.356636	2.038953	4.947047	3.953182	4.636494	3.348336	6.821577	3.830808
Cs5g34100	2.999845	3.480798	3.006327	2.934208	2.298604	4.07321	2.459499	5.967554	1.794107	5.101923
Cs5g34230	4.49516	4.602572	3.883148	3.753955	2.541968	3.688007	0.883871	1.338756	7.102005	6.770025
Cs5g34260	0.5484	0.649195	2.084564	1.805887	3.110605	4.084613	3.687934	2.349586	0.958657	1.904844
Cs5g34280	4.350666	4.349637	7.24008	8.060506	2.785065	2.626146	1.47215	1.455614	0.146791	0.238374
Cs5g34360	0.42219	0.18561	1.022216	0.986661	1.45392	1.378678	1.075856	0.975031	0.594727	1.463845
Cs5g34390	29.6719	20.1184	18.99422	14.782	16.16146	26.09037	3.70104	14.09639	1.645743	9.911317
Cs5g34410	13.16904	6.806322	24.64076	21.25823	46.21411	52.9528	67.11673	38.50444	37.75492	48.07493
Cs5g34430	4.495773	5.339501	7.495512	6.391118	2.828808	4.426325	5.254871	11.43522	3.021029	10.02019
Cs5g34450	48.36095	77.26273	21.122	21.79421	10.32901	11.43799	2.902435	10.09433	2.208411	4.58335
Cs5g34540	0.52628	0.167735	0.243969	0.139143	0.48471	0.296871	1.919741	0.746776	0.102289	0.086092
Cs5g34640	2.572142	2.909754	0.320785	1.07711	0	0.035253	0	0	0	0.035671
Cs5g34690	86.85211	113.8601	48.68031	76.72673	2.551991	4.534152	0.944002	2.285152	0.278396	0.532676
Cs5g34700	2.880934	2.632096	9.35308	7.184699	8.001448	10.1603	8.827194	9.512176	1.916395	0.898028
Cs5g34830	5.620844	9.292927	12.09089	18.01058	15.02736	14.56123	15.14201	14.52321	5.034002	20.52806
Cs5g35080	0.51703	0.933975	2.228275	2.461213	2.355081	2.07494	1.796543	1.373623	1.506022	0.370846
Cs5g35100	3.153605	4.122719	10.83343	12.48244	9.097905	10.7885	7.949848	3.88245	1.410393	1.612932
Cs5g35190	0.786613	1.055924	1.101036	1.056929	0.907696	1.634272	0.486454	1.205554	1.17918	2.779914
Cs5g35220	4.710833	4.531258	4.805481	3.992345	1.501421	2.596547	3.827628	5.943409	0.912968	3.644729
Cs5g35320	5.280488	3.365464	11.36428	9.616075	11.09903	11.3432	8.314447	12.63029	10.14465	17.62022
Cs6g01030	1.301481	0.97823	2.344941	1.182221	3.825606	2.74207	1.756382	4.185689	1.332543	1.54213
Cs6g01070	17.32127	28.40625	18.98843	22.54979	5.206369	6.352952	11.71369	17.24747	6.891603	14.58241
Cs6g01080	1.723721	2.564852	2.479488	2.64863	0.33751	0.552309	0.877487	1.513533	0.715898	1.546598
Cs6g01570	4.885951	5.823455	5.4597	5.154966	6.812726	7.225306	1.975558	11.35582	11.84494	6.041389

Cs6g01640	1.992875	2.40245	2.587669	2.846551	1.801449	2.214889	1.834248	6.271467	3.458409	1.496547
Cs6g01745	1.840773	2.087257	2.222777	2.010393	1.557881	1.943291	0.786384	2.641206	1.078243	1.572932
Cs6g01840	157.0751	114.8329	53.86037	56.54805	29.07903	30.21478	6.556357	31.09669	81.85928	74.47211
Cs6g01850	105.5005	91.8759	141.7085	102.9047	208.2177	162.5907	95.94321	319.6614	246.1494	341.8752
Cs6g01870	13.02123	12.89876	49.82664	32.96056	55.62586	61.1112	7.179789	19.80717	11.03245	10.81126
Cs6g01980	1.86238	3.049861	44.68204	33.82402	217.522	171.2426	293.7621	444.742	323.2491	444.6007
Cs6g02000	0.360133	0.340251	0.871432	0.769628	1.377866	2.201993	1.024441	1.544218	1.132913	1.083184
Cs6g02010	0.332092	1.4113	0.236996	0.231567	0.076747	0.03322	0	0.040308	0.076795	0.221037
Cs6g02180	21.79166	20.69593	16.8886	14.44687	15.19708	13.0035	7.183615	7.074198	1.661378	0.686619
Cs6g02190	3.159978	3.477879	4.820696	3.422308	3.448755	2.957471	1.01731	2.640534	0.215477	0.464097
Cs6g02200	4.509033	4.158046	2.54458	2.334574	0.952171	1.555106	1.097073	4.079033	0.711224	0.600677
Cs6g02260	4.033645	3.511542	12.80211	16.32573	8.638092	21.09525	13.69814	26.38245	19.97911	40.06199
Cs6g02270	5.044881	5.066391	5.189491	4.849041	5.476298	6.323733	2.15431	4.82586	4.967889	4.559256
Cs6g02325	1.875791	1.279105	3.142704	1.369199	6.72343	5.384356	3.982518	7.258293	1.744759	1.536445
Cs6g02360	0.975274	0.707583	3.80718	1.681126	74.28584	35.96081	141.7657	86.38786	34.58908	16.50313
Cs6g02380	5.714281	6.479914	5.868498	6.98859	6.236178	8.282588	2.089949	7.145717	4.511661	5.764563
Cs6g02470	0.37749	0.798048	0.67603	0.342423	0.622608	0.245815	0.6736	0.730358	0.457863	0.243126
Cs6g02500	0.410452	0.79155	3.691024	2.787311	3.111823	4.346457	3.440401	16.47319	2.376691	7.151827
Cs6g02580	9.131175	9.326786	10.42269	10.25741	5.701668	8.160127	3.09162	9.373974	7.641241	12.57728
Cs6g02700	0.747921	0.527372	2.026602	1.330146	3.555663	2.293288	3.935315	3.306445	6.160632	4.907631
Cs6g02760	7.731744	11.54237	4.613415	7.256979	2.984497	3.222868	1.289926	3.737469	0.468096	1.550992
Cs6g02770	8.523818	12.92519	5.393706	9.576901	3.730871	4.896378	2.483719	5.215023	0.753549	3.140247
Cs6g02830	18.10553	34.90096	18.15147	22.21633	44.27637	40.23579	47.51827	81.12362	17.90579	36.2351
Cs6g02940	2.872292	3.435624	2.081884	2.630522	1.458758	1.806068	0.316299	1.992547	1.151261	1.597407
Cs6g03090	1.343714	0.891632	2.863504	2.085706	1.327376	1.904218	0.208731	1.016545	0.024349	0.022373
Cs6g03210	43.46539	73.64679	14.92596	19.29956	1.011646	1.259935	0.188371	1.387782	0.461771	0.135248
Cs6g03220	16.17771	18.64036	17.16845	14.6505	0.794182	2.842059	1.302256	3.347364	0.088601	0.096365
Cs6g03280	197.5108	126.475	327.2915	261.9244	450.6763	584.3259	109.0822	105.3488	98.75898	75.73084
Cs6g03330	7.534198	9.314748	7.961713	7.096	15.20614	15.29408	11.27192	4.643259	5.540276	1.966409
Cs6g03340	8.57145	10.97726	14.64105	11.85393	18.59907	23.98717	22.62748	9.650772	9.994372	3.899965
Cs6g03350	11.5416	14.99829	25.98688	20.2171	28.14905	28.41089	19.59926	9.592418	1.69429	0.589067
Cs6g03640	11.64506	14.23421	18.43512	19.70491	10.81244	17.3264	7.058445	18.46415	14.87513	41.25452

Cs6g03700	7.581845	3.557506	2.436992	2.108117	1.219879	2.511706	1.236914	1.007527	2.451597	1.197299
Cs6g03770	7.990607	10.05482	3.286315	4.512092	2.646381	2.513422	1.138248	1.122535	0.1232	0.094398
Cs6g03830	1.713197	2.290896	3.977818	4.019612	4.997264	4.828896	3.512214	5.766452	24.11779	31.72868
Cs6g03850	2.911976	1.163352	8.114045	4.58642	6.001461	2.889352	2.06921	4.252616	0.172249	0.038837
Cs6g04000	15.48028	21.93334	14.14561	15.73656	23.48443	18.89529	43.18714	37.8587	31.28189	38.17956
Cs6g04020	3.843769	4.903965	1.976463	2.6047	1.850246	1.950607	1.405121	2.364717	0.779948	0.764506
Cs6g04050	5.656607	10.51726	2.454346	4.676414	2.237489	1.523019	1.817576	4.992214	0.98977	2.044591
Cs6g04060	19.02021	51.46289	5.774065	21.05255	6.723465	4.131415	21.25435	27.5371	11.39168	18.2675
Cs6g04070	31.08539	24.09602	42.76255	34.28063	49.47773	45.24626	104.7359	46.54212	73.49506	52.92336
Cs6g04090	72.91111	60.66532	107.135	85.75186	103.4738	100.0873	166.4131	75.46389	110.843	78.2058
Cs6g04120	6.478691	5.645944	10.32519	10.27899	6.247716	8.402824	2.221716	5.442867	5.9595	2.71572
Cs6g04140	10.32163	15.89313	25.55162	20.61004	24.04967	21.76917	22.8024	33.76878	44.76946	54.83462
Cs6g04150	4.215094	4.26323	15.09891	13.0473	18.11292	14.68461	5.624567	20.47155	6.485159	10.8604
Cs6g04210	0.997064	1.173868	0.961058	1.147722	0.907023	1.21493	0.424646	1.372958	0.809045	0.932806
Cs6g04230	14.54285	18.24771	18.65935	19.1121	14.63831	13.78843	17.21027	18.3203	1.101519	3.505659
Cs6g04240	1.195284	1.217581	2.811159	2.589915	4.687811	4.955083	3.746041	5.241627	11.12075	12.39339
Cs6g04290	3.423631	4.572592	3.163968	2.896918	2.140765	2.266539	1.084758	2.535865	1.171262	0.891715
Cs6g04360	8.433849	10.55295	2.829426	3.869066	1.440513	1.51185	0.930426	1.567303	0.190665	0.323735
Cs6g04450	116.5842	76.46304	68.95053	75.53465	75.19234	146.403	272.3535	65.51058	107.4937	189.4728
Cs6g04480	0.081701	0.1044	0.273825	0.403922	0.48013	0.699953	0.299798	0.812615	1.388221	2.307928
Cs6g04530	1.612575	1.564057	1.68605	1.518239	1.353778	1.479417	0.802848	1.751772	0.833175	0.957824
Cs6g04550	2.024555	5.08483	6.861597	7.544412	4.008089	5.018235	1.986502	9.023868	1.870799	2.203232
Cs6g04620	10.44009	9.581124	4.335889	3.537823	2.155932	1.363104	1.331172	1.613628	0.37778	0.186839
Cs6g04690	0.49241	0.292669	0.857117	0.649506	0.793045	0.639093	0.263839	1.035504	0.320923	0.431693
Cs6g04710	1.987118	1.216894	2.470381	1.70309	1.960019	2.911384	0.317357	0.854627	0.089395	0.120871
Cs6g04790	0.3008	0.439076	0.414936	0.470337	0.423227	0.858816	0.206929	1.024703	0.716886	0.408419
Cs6g04810	0.879219	0.610252	0.763804	0.683675	0.77376	0.782516	0.307896	0.757375	0.690368	0.568509
Cs6g04830	0.805176	0.752912	0.484185	1.390382	1.052213	1.307267	0.416253	1.443313	1.074957	0.965734
Cs6g05010	7.929985	5.214856	3.903984	7.678006	1.339319	5.273815	0.826455	1.767816	9.925299	17.51265
Cs6g05050	6.198117	4.472631	7.253126	5.698742	9.337771	7.687868	8.740932	7.602799	10.3661	4.642247
Cs6g05100	14.05835	14.96572	13.68421	14.83694	11.77552	14.52413	8.200661	17.81891	16.02889	15.06486
Cs6g05140	8.831953	8.392803	193.922	181.052	191.0569	281.4444	381.0291	663.3319	7.412352	33.61964

Cs6g05150	0.020339	0.060509	1.121049	0.769149	0.17237	0.60278	0.252082	1.554272	0	0
Cs6g05515	0.143925	0.100972	3.390885	2.604062	3.568945	5.330965	6.318253	9.851951	0.141042	0.742628
Cs6g05520	0.032566	0.024493	0.68239	0.786291	1.008765	1.556157	0.910075	2.429029	0	0.131511
Cs6g05570	9.096381	6.067244	20.24787	13.02096	28.28485	27.78877	58.33247	19.61236	22.76714	16.11127
Cs6g05580	9.067392	4.945253	14.89017	16.77005	37.43148	33.42235	66.28919	90.19427	4.787471	4.045939
Cs6g05630	2.691946	2.772113	2.785961	3.615072	0.987894	1.923993	0.54707	1.138757	0.271206	0.813483
Cs6g05660	4.526157	3.234384	6.163721	4.467393	0.432172	1.722847	1.215589	1.744761	0.321829	0.749626
Cs6g05740	71.62223	52.06391	144.5171	164.975	178.302	255.1333	154.0118	167.114	164.8789	153.9203
Cs6g05750	3.181865	1.093247	2.536387	1.386828	3.353814	1.170537	1.483102	1.092501	2.115535	1.527033
Cs6g05760	1.206386	0.340379	1.397627	0.532803	0.826922	0.868701	0.51869	0.457205	1.678951	1.150052
Cs6g05810	35.25283	40.48264	49.0119	51.15709	70.88291	79.01022	85.33818	79.08731	77.50306	112.2642
Cs6g05820	0.179737	0.182291	0.339533	0.155226	0.325359	0.240241	0.030689	0.133625	0.225277	0.959604
Cs6g05860	3.286281	3.895769	3.660223	3.609721	3.239315	4.232285	1.519936	6.72608	3.446376	2.975111
Cs6g05990	2.082092	1.793688	7.907618	8.119151	2.312067	5.663652	6.703647	4.111752	12.05071	14.16311
Cs6g06060	0.505284	0.453737	0.776088	0.483615	2.168739	3.604693	2.740208	3.524804	0.46685	1.026001
Cs6g06100	5.543994	5.146233	6.438324	6.570585	11.64497	11.60374	16.17031	7.531946	16.81405	12.38029
Cs6g06255	5.554739	5.438782	7.895214	7.489331	9.651898	8.492112	3.649713	11.14625	7.471445	7.494766
Cs6g06470	6.238052	6.766981	5.833975	6.063924	5.076819	7.039309	1.852763	5.502569	3.201576	4.497358
Cs6g06530	34.28245	23.70472	24.66293	23.42071	20.40172	55.63029	24.77143	38.76277	8.303028	3.342664
Cs6g06570	51.90503	41.4253	36.45754	32.21598	23.75473	21.84472	24.9085	13.81728	6.926226	4.47695
Cs6g06660	4.387269	12.51863	2.433799	4.0566	2.824318	2.110831	2.89988	4.8325	5.098679	5.891242
Cs6g06690	2.039173	2.508949	9.034054	8.791833	17.42324	17.46665	21.61104	33.26699	24.81734	52.11219
Cs6g06700	1.410789	1.060992	7.685413	5.805512	12.73427	11.56002	11.13513	10.22155	7.233172	5.440054
Cs6g06730	8.192507	12.32724	7.577803	9.731141	6.299909	7.120904	4.24568	12.70073	3.840436	7.350102
Cs6g06800	24.74653	27.59368	35.29754	35.48804	43.0656	36.11327	102.34	49.21553	33.97855	31.07123
Cs6g06850	45.57245	91.72194	12.90585	17.51274	3.590856	3.613675	2.441863	5.577066	2.210735	5.132593
Cs6g06900	13.92113	12.73571	9.797608	10.05065	9.551367	13.23437	4.854274	12.18104	14.78164	18.29584
Cs6g06910	30.49372	39.26178	17.67244	22.8833	11.5296	14.62143	10.88645	26.50752	6.424716	17.3369
Cs6g06960	1.331614	2.366139	0.952459	1.444372	2.007437	1.717288	8.306978	5.801098	3.352429	4.574858
Cs6g07000	8.974655	3.635808	7.374274	9.923838	1.952121	3.20101	2.638284	8.051754	3.719143	3.688313
Cs6g07010	2.514975	0.638037	2.119856	1.664786	2.898334	2.362365	2.167219	1.639109	1.096445	0.366191
Cs6g07030	1.84693	1.189097	1.135028	1.816338	0.056738	1.267102	0.177333	0.127672	0.047898	0.068932

Cs6g07040	3.071503	1.977085	9.546845	6.950635	11.83519	14.13474	16.26217	9.282767	6.881061	4.940484
Cs6g07130	41.14736	30.81641	16.04375	16.36512	4.490235	11.12818	8.767892	16.75039	4.331261	1.278898
Cs6g07140	0.44901	0.759991	4.188181	3.494856	6.337911	5.00352	7.337084	6.861018	0.607972	0.154361
Cs6g07190	67.75175	34.86561	40.82255	53.53783	40.09875	72.98128	37.81843	32.99878	43.32458	57.26047
Cs6g07200	232.4769	163.4171	167.5178	136.8543	79.54385	81.81544	12.16902	16.09106	33.89975	24.90501
Cs6g07240	1.376968	1.021034	3.969836	3.582924	5.807778	5.276041	29.6939	7.832963	9.360185	8.328376
Cs6g07320	7.749993	9.855993	10.06177	11.01784	9.748965	9.590443	36.28184	74.49349	36.17109	47.68856
Cs6g07360	122.9024	104.9713	113.092	153.3471	36.07526	76.54492	61.08832	79.47595	234.501	450.3922
Cs6g07370	10.65193	7.498018	12.3694	10.40786	10.33003	16.61718	9.653941	10.32132	56.19916	142.8159
Cs6g07400	2.80421	5.147994	3.525868	4.067454	3.290185	5.473763	8.477742	11.97261	212.5358	348.4869
Cs6g07420	4.190266	4.14109	8.207514	8.413035	9.25981	16.84332	26.9408	28.43168	71.05209	144.8585
Cs6g07430	25.70987	42.60688	7.37495	20.40092	1.225245	1.568586	0.973619	2.459624	0.847837	0.423556
Cs6g07470	5.280013	7.127236	1.908118	2.502715	0.842582	1.419001	0.954006	2.541961	0.151448	0.36746
Cs6g07510	5.471654	6.239546	6.833035	7.136672	0.862199	3.245615	5.978009	19.83861	3.112172	19.95228
Cs6g07540	12.4655	11.81894	7.195922	8.068394	6.669522	5.473881	8.950391	6.412904	9.961356	4.089601
Cs6g07690	552.7162	479.265	1057.905	517.8833	1102.496	289.4009	150.3478	274.8694	54.75265	30.60763
Cs6g07700	234.3411	188.1651	393.5752	228.6802	542.4341	224.1765	281.1743	284.2511	117.7897	56.8023
Cs6g07730	2.611893	2.788943	1.840394	1.528171	2.228088	0.909316	2.954485	1.740759	0.354171	0.30599
Cs6g07740	5.240503	7.486933	4.586083	10.52859	9.798754	8.575611	8.137004	10.25891	9.577402	4.547837
Cs6g07770	9.427263	8.523552	7.235069	7.580348	2.65626	3.507792	1.455853	2.33851	0.784812	0.949736
Cs6g07830	92.87972	48.21165	208.5476	268.1115	137.1935	185.6512	347.387	199.6304	157.2391	387.4527
Cs6g07850	1.937019	1.903928	0.68813	0.612806	0.582731	0.496227	0.274774	1.552886	0.0498	0.153688
Cs6g07970	326.5067	130.1054	73.91844	96.41556	271.2947	229.0572	1102.962	195.2793	484.2007	77.80316
Cs6g07990	6.215448	10.80109	5.562569	7.804492	1.979414	4.419466	2.139326	5.594872	0.195747	0.259095
Cs6g08050	0.103135	0.286774	1.423958	1.441226	2.856701	2.959065	1.825214	6.051966	4.50446	7.145433
Cs6g08060	0.697832	1.078757	6.018235	5.911527	5.694407	7.38755	10.3422	16.50736	3.135984	11.24268
Cs6g08100	10.73246	4.539333	10.40483	12.40903	3.463135	4.783455	6.29225	12.81654	6.311507	6.065396
Cs6g08150	0.574023	0.374346	0.537518	0.663359	0.669559	1.068256	0.320085	1.042337	0.485131	0.545446
Cs6g08175	0.209851	0.346788	0.546379	0.829273	0.594901	1.58519	1.58719	6.468372	15.41048	23.53313
Cs6g08240	2.693343	2.796206	2.926801	2.742822	3.416541	3.979763	2.999115	8.519915	3.42903	3.134978
Cs6g08260	34.79972	91.38615	3.20804	6.134848	16.95892	17.39228	3.609693	12.66327	2.602485	4.379843
Cs6g08280	7.213877	9.118005	7.102217	7.320562	10.79511	8.355043	4.772496	13.02109	2.876106	3.084561

Cs6g08320	44.75868	89.26989	21.36062	19.49161	12.67579	10.25243	21.51178	23.12346	3.175521	2.847641
Cs6g08360	8.039299	13.65912	1.347116	0.802091	0.458696	0.248657	1.008422	4.708066	0.465743	0.403499
Cs6g08365	4.913511	5.232017	5.395293	6.611246	4.514514	7.259646	4.335017	7.087069	6.834372	14.92978
Cs6g08390	4.932911	6.735981	10.84295	7.440444	7.795526	10.41676	7.330301	21.20662	50.03509	91.18741
Cs6g08440	3.903279	5.501597	2.385629	5.071664	1.200582	1.4729	0.843974	2.372362	0.189112	0.358026
Cs6g08490	6.717196	9.586238	9.755338	10.04761	2.483426	3.094928	0.794071	1.826232	1.702332	1.469668
Cs6g08500	2.800342	3.586687	8.814867	7.554537	7.548167	17.62969	49.14618	8.228973	281.4468	60.47667
Cs6g08550	2.194029	2.149017	2.117001	3.615789	0.479455	2.485546	2.544203	4.727534	12.87217	25.53004
Cs6g08560	28.57101	19.10093	19.10018	19.79377	10.0654	21.69365	12.65181	10.46493	28.3653	13.9667
Cs6g08590	3.274285	4.001342	3.096433	3.510082	2.12709	2.625165	0.624899	0.638263	1.721765	0.51924
Cs6g08600	147.3376	151.7036	281.2537	355.9608	139.504	338.8698	307.6869	202.9846	1689.308	1277.958
Cs6g08610	3.13997	3.02903	4.859681	9.516541	1.080582	4.669699	0.684758	1.419996	28.63298	32.88284
Cs6g08900	1.789358	3.150513	1.822485	1.329226	1.849692	0.677965	0.996727	4.405574	6.133118	11.77502
Cs6g08920	0.564589	0.399669	1.278588	0.934997	1.561638	2.716551	2.080878	1.454465	8.510469	6.61473
Cs6g08970	7.19316	11.64209	7.889347	12.29633	9.779048	7.575406	8.359854	23.88253	0.716672	2.519107
Cs6g09030	2.792715	4.103456	4.769097	6.279919	3.74294	6.125199	17.87202	13.30218	7.927324	9.602993
Cs6g09040	4.08714	7.967416	13.7496	11.58003	6.237231	6.415908	8.622656	12.71519	3.814493	4.313922
Cs6g09130	2.459066	0.531577	0.198924	0.216856	0.3432	0.102754	0.056683	0.060926	0.123034	0
Cs6g09150	959.4302	938.6745	498.6602	808.2724	579.891	305.843	154.7903	391.3015	86.51537	134.8824
Cs6g09160	0.45101	0.752781	8.721615	4.426913	67.60052	39.72794	155.7808	161.8047	39.4853	83.04135
Cs6g09330	6.367953	6.052052	6.738458	8.13262	2.516767	3.862308	3.006408	7.061531	4.028941	10.71661
Cs6g09340	3.377987	3.731933	2.225732	2.120829	0.412982	0.878828	0.429611	1.268019	0.057867	0.041161
Cs6g09400	6.270919	20.47113	16.84228	19.24144	1.942653	2.614823	17.10923	26.4777	5.71767	16.98957
Cs6g09420	10.85225	18.28507	22.26636	30.52432	5.854754	9.789176	8.538095	19.91027	1.048884	12.22397
Cs6g09440	3.556567	3.889302	10.15902	14.0125	10.67202	11.96787	9.342758	22.21578	5.471167	6.592525
Cs6g09480	0.436593	0.331597	0.924975	1.009161	0.895689	1.219	1.275529	3.755628	0.128496	0.263885
Cs6g09510	10.41056	12.23343	20.89893	19.73623	24.41443	19.82762	40.44395	34.24877	34.22791	70.26824
Cs6g09525	8.548397	22.91974	10.3306	14.32651	1.670274	5.862104	0.093924	0.750016	0.099036	0.049119
Cs6g09530	19.54268	15.6328	141.9611	79.97364	99.49414	139.2386	17.06559	17.36714	16.11159	19.59977
Cs6g09540	65.15015	42.21582	106.3544	103.6248	67.84123	125.7824	41.84979	62.55248	60.14897	219.7234
Cs6g09560	15.15776	26.2561	19.2827	22.4549	3.284923	4.076913	9.469457	24.50361	16.58155	45.87635
Cs6g09610	18.93606	27.08664	65.15109	63.46432	20.65119	28.70348	31.69596	70.96361	5.167707	45.22836

Cs6g09660	0.164043	0.29587	0.332541	0.342787	0.129071	0.156229	0.229822	1.20333	0	0.034593
Cs6g09670	7.173364	10.90346	6.734536	11.83221	6.667067	9.904149	1.303122	3.529695	4.324406	9.659355
Cs6g09690	0.267706	0.277103	0.311451	0.377772	0.393539	0.337389	0.29757	0.976349	0.711924	1.070904
Cs6g09705	4.069735	5.555708	6.086583	5.2389	6.046476	7.279087	2.799996	7.641214	4.495387	3.922068
Cs6g09730	102.0056	124.5565	171.1924	181.8506	58.89813	79.04072	85.34495	132.5363	31.07506	158.6537
Cs6g09780	5.643	8.715952	3.443099	3.443884	1.68255	1.788703	5.262588	8.544861	19.45632	32.41434
Cs6g09790	0.164115	0.207416	0.256288	0.226698	0.056828	0.397006	0.150752	1.542425	0.451571	1.690221
Cs6g09800	1.319536	0.980322	0.558818	0.444086	1.586812	0.848469	2.271005	1.04843	0.66457	0.454309
Cs6g09810	0.06588	0.046524	0.105177	0.02434	0.48828	0.38761	1.476382	1.439912	3.700418	2.897717
Cs6g09880	8.462684	7.018336	10.61743	10.91233	5.188282	9.521593	3.919927	5.011421	6.74657	4.233566
Cs6g09950	432.3289	276.5012	92.6128	54.97602	72.53299	49.37732	37.40482	57.42096	4.328211	9.814371
Cs6g09960	215.0267	180.0328	42.88914	34.55235	396.6555	62.34242	3322.093	304.2156	247.3894	283.9034
Cs6g10120	40.97527	40.62194	91.89676	62.77563	234.0148	243.0869	15.4167	17.0854	1.381156	0.861665
Cs6g10200	2.979822	2.6821	3.252643	2.594187	3.879722	3.215568	1.51238	4.770502	0.792633	0.934476
Cs6g10250	60.3601	71.41937	42.28947	51.08848	19.04563	23.4224	47.54528	61.41859	48.90692	137.345
Cs6g10270	341.6904	248.7968	272.7928	365.0394	134.811	135.9097	102.6493	188.2124	181.7442	299.1236
Cs6g10280	509.5505	594.4572	595.5665	664.7805	325.0803	373.1986	212.5229	279.2569	298.9119	517.7395
Cs6g10310	4.276789	3.416173	4.907607	3.902457	9.032958	8.161046	9.84064	5.881826	4.598119	3.619664
Cs6g10410	2.422958	2.362105	1.989446	2.014788	1.064825	1.146288	0.802805	1.572784	1.032916	0.310283
Cs6g10490	1.087718	2.461961	0.686766	1.364183	0.141784	0.33682	0.852964	3.40499	0.323619	1.27116
Cs6g10520	29.94532	35.87949	55.97943	64.35164	24.66937	35.40959	24.30273	47.58071	19.15727	72.29831
Cs6g10600	107.224	88.16211	166.9483	171.5895	307.5945	267.5525	441.0162	267.883	337.6896	228.1026
Cs6g10610	15.84097	12.9329	6.781576	7.544907	7.855485	12.27502	17.47683	11.81997	43.47882	17.16609
Cs6g10640	5.631885	13.86439	3.449978	7.340796	3.14713	2.758656	3.104274	7.176491	0.159211	0.545609
Cs6g10650	0.218465	0.417783	0.18487	0.207923	0.220711	0.692292	0.607629	1.679406	0.19812	1.282403
Cs6g10660	0.571753	0.859425	0.942331	0.802244	0.611618	1.624606	3.25907	8.294268	0.738084	6.539499
Cs6g10675	0.332861	0.710124	0.848951	0.592141	0.56768	0.623797	0.496408	0.885078	0.086666	0.348536
Cs6g10750	1.053497	1.191443	0.830587	1.233849	1.248928	1.152113	0.490708	1.544356	0.826551	1.19922
Cs6g10800	3.641697	3.75883	4.352574	3.757755	3.516801	4.92402	2.374004	5.161161	4.542802	4.36605
Cs6g10840	8.270651	8.023101	6.264378	7.272953	3.929601	3.744561	4.414839	3.403468	4.219689	5.123444
Cs6g10880	1.980158	3.629455	6.093391	5.261668	2.038602	2.14346	2.386781	8.924826	5.028123	5.226864
Cs6g10900	0.297236	0.421546	0.225469	0.20492	1.056871	0.620194	1.860419	1.161707	2.161532	3.904383

Cs6g10940	0.469909	1.194727	0.487813	0.378908	0.655496	0.367129	0.731982	0.801082	0.198725	0.219231
Cs6g10950	4.591759	5.792068	2.660004	2.701037	1.780659	1.402101	2.085659	4.900606	3.530022	2.728879
Cs6g11040	1.030051	0.979503	2.894535	3.33669	1.375398	2.484739	6.218192	7.090956	9.605634	5.292584
Cs6g11150	3.496741	4.159074	3.919578	3.56083	2.539962	2.240077	1.589553	2.606148	0.820977	0.282571
Cs6g11170	47.78796	43.50498	30.89676	32.76813	20.83684	22.61428	10.79745	31.4741	8.349114	9.439043
Cs6g11190	2.616924	3.056439	3.498877	2.493634	2.275965	2.130717	0.839506	1.163358	0.029759	0.010287
Cs6g11230	29.84312	31.76176	25.26134	12.27723	27.06709	12.0339	18.39786	15.3867	24.80808	25.38493
Cs6g11260	13.65303	10.67882	22.53773	15.98682	37.21334	33.43431	22.78425	19.48897	47.89096	20.74115
Cs6g11300	0.161564	0.410442	0.907695	1.09328	0.234672	0.196577	0.303842	0.892776	0.760032	2.082879
Cs6g11310	89.54564	100.5394	73.86899	99.76791	26.34444	47.21375	77.10473	170.2088	529.1267	1207.92
Cs6g11350	92.68441	33.34774	41.39767	42.58914	42.75798	45.72315	60.47129	40.58181	80.68716	38.14819
Cs6g11390	24.15482	22.144	11.74176	19.68407	14.41311	20.24215	8.487724	16.72532	14.64255	22.65614
Cs6g11460	1.499422	1.087931	7.234774	5.771442	39.46917	58.34392	29.13126	19.21211	4.812146	2.90862
Cs6g11470	5.9818	5.26926	24.02528	23.30434	59.31163	103.1667	59.9604	61.6457	45.8402	40.74391
Cs6g11480	16.93038	20.15197	8.901957	11.1381	13.77566	15.94741	30.98322	25.54015	30.15178	20.66861
Cs6g11570	62.2544	36.11005	10.64721	18.5912	3.223501	57.74438	14.64325	33.38356	0.301474	0.113582
Cs6g11600	68.07774	54.91708	30.93996	27.7699	17.68831	14.62765	17.86399	22.17416	5.549612	2.065731
Cs6g11610	41.47391	33.64595	18.7118	18.28918	10.28195	9.923496	11.82312	14.42392	3.128393	1.589931
Cs6g11660	42.3083	17.26345	3.153254	7.3969	11.28603	7.451605	6.512286	2.775736	7.379596	0.474939
Cs6g11690	239.772	112.6065	27.40672	51.52502	78.81669	57.951	47.96435	21.90193	30.86078	0.822424
Cs6g11700	128.5928	54.63682	8.128941	20.85858	30.88621	20.31034	17.76337	7.247251	21.89829	1.338251
Cs6g11730	6.130738	6.294503	7.765679	7.18648	8.320774	9.656349	3.751464	10.70935	7.732924	6.030179
Cs6g11750	0.803325	0.355024	0.561794	0.310928	0.481852	0.266185	0.130595	0.142668	1.05882	0.629142
Cs6g11800	7.720738	5.826866	5.40536	4.641636	5.805617	4.664968	1.993057	1.490833	8.636396	2.110891
Cs6g11920	0.730805	0.999262	1.710109	1.619222	4.732039	3.086903	4.365862	5.137347	2.039266	2.193654
Cs6g11940	25.26929	25.66245	48.92909	57.56718	72.15901	125.2734	74.73217	32.75099	14.91127	10.25378
Cs6g11950	2.155059	1.52393	7.264805	8.568869	6.513654	16.87544	18.64283	6.961843	10.17729	10.63129
Cs6g11990	61.67722	55.23224	101.4445	101.663	190.2773	175.5145	338.4815	99.81569	112.4764	109.1336
Cs6g12060	6.87034	7.328414	22.3835	31.91532	7.057644	15.86394	0.703221	5.364998	1.411447	7.784606
Cs6g12090	2.35989	2.381378	5.452062	7.736826	2.381353	4.50222	0.079061	0.524286	0.113629	0.335086
Cs6g12110	32.97834	34.30985	40.41402	56.49955	10.20106	20.47376	2.720669	16.25185	3.600475	29.58798
Cs6g12170	5.109419	5.519458	8.907902	8.474728	9.389923	12.51432	5.001818	18.68751	7.601585	11.12635

Cs6g12270	2.311842	2.415864	2.603076	2.73362	2.210865	3.153379	0.851132	3.61126	1.354325	1.780475
Cs6g12330	3.036792	2.388402	4.902402	4.37968	2.454732	5.119265	1.112383	2.3343	1.849443	0.587333
Cs6g12340	5.891953	5.638625	6.897051	7.357161	4.771363	5.8097	2.695933	6.733988	1.736521	3.245212
Cs6g12390	17.97703	41.41897	3.04686	2.954208	0.419274	0.745437	0.11616	1.211113	0.442868	0.273256
Cs6g12410	13.91853	14.75405	26.32361	22.19745	28.40857	22.3786	40.26852	37.66188	14.29038	23.6949
Cs6g12450	19.82785	22.90301	3.054501	7.066267	0.133926	0.201883	0.090899	0.614711	0.123198	0.701728
Cs6g12480	201.5852	396.3138	401.0447	557.8225	40.95093	78.05126	190.7507	390.6017	64.00554	438.6604
Cs6g12520	6.043609	3.669472	5.593883	5.990037	5.467184	9.9339	8.650459	5.4008	13.81902	7.269842
Cs6g12620	28.48131	27.0908	42.26461	43.57422	22.05607	33.54418	15.49935	36.61028	66.3994	56.96038
Cs6g12680	12.95824	13.16297	6.705625	6.848514	7.006747	5.038321	9.401168	6.330561	9.672191	2.001669
Cs6g12830	28.36734	21.06791	45.98933	32.3579	37.83427	43.07832	5.297525	19.67494	1.516222	0.941886
Cs6g12870	41.97177	20.88968	39.64978	22.54327	161.7209	93.93142	398.5697	222.788	130.715	132.525
Cs6g12880	0.691508	2.310048	1.630672	1.46984	104.548	132.3049	349.1986	367.4085	353.2523	381.0024
Cs6g12890	2.023903	1.274702	1.387738	1.876588	1.628487	3.72227	14.6286	14.06551	19.93844	35.58935
Cs6g12920	17.26918	12.39629	12.15196	9.050799	11.30486	26.09073	3.151794	2.53817	0.478564	0.73813
Cs6g13000	62.65054	47.2078	70.83542	58.36342	74.44305	65.62049	74.12999	29.83534	91.3439	62.06678
Cs6g13190	11.48559	12.7925	10.81063	12.20805	23.5794	12.12774	14.76934	31.10806	5.596732	10.45129
Cs6g13326	5.587973	7.741456	0.893913	1.299213	1.357567	0.571789	3.452487	1.564808	0.102202	0
Cs6g13330	9.445451	14.08212	2.133704	3.760747	2.6145	1.731144	5.595001	3.453019	0.304643	0.192693
Cs6g13360	7.355044	4.149179	8.83438	6.44068	20.12979	13.71603	8.351051	6.34356	20.82561	13.48826
Cs6g13410	15.8589	19.01012	9.09118	9.604773	5.613858	7.921767	7.029008	19.76526	5.056073	14.92969
Cs6g13420	35.35035	41.27168	23.5685	26.00561	7.726382	9.497152	3.146056	9.70217	14.21761	11.65068
Cs6g13530	0.436607	0.373624	1.082961	1.010243	1.696087	1.779839	1.471233	1.431183	0.868228	0.452922
Cs6g13560	13.03141	14.20814	25.7079	23.79594	21.30386	17.6528	7.248053	10.56749	2.711079	2.525957
Cs6g13590	44.65573	64.60179	79.23586	112.5592	16.7261	34.24391	41.81188	78.2878	37.64966	163.1082
Cs6g13640	52.39775	59.30925	37.96292	38.40458	6.559966	13.21268	47.3043	60.72883	38.37039	69.389
Cs6g13740	0.670271	0.699993	1.010036	1.483029	2.149323	3.520369	1.859566	2.543746	3.959766	3.887315
Cs6g13750	3.040708	4.122956	5.03632	4.083963	4.203549	3.713054	1.548075	4.854109	2.930172	6.021174
Cs6g13770	5.259217	8.50682	1.73044	3.254877	1.102751	0.950427	0.267829	0.644542	0.407263	0.685217
Cs6g13800	3.974151	5.686675	7.120119	6.766599	4.804233	4.428438	3.482381	4.474638	0.651545	2.017036
Cs6g13820	22.61783	44.36597	61.97936	61.46657	19.54213	19.84319	56.78511	114.3387	59.62822	118.0253
Cs6g13860	13.71589	9.094036	12.01087	9.352784	14.62233	20.00518	2.381361	5.448931	0.287611	0.370224

Cs6g13890	1.672118	1.155512	1.369908	1.360455	1.153575	3.367601	1.503826	1.509988	13.74793	8.453161
Cs6g13900	3.948226	8.750276	6.170584	15.26717	4.527234	8.139016	5.62169	9.351398	8.044888	16.82801
Cs6g13920	5.840962	6.948627	9.261536	9.178116	7.604963	8.486381	3.014578	8.846615	4.97754	4.871757
Cs6g13980	4.619394	4.33152	7.307394	7.017444	5.60993	9.191096	3.690061	10.0175	1.681039	2.304753
Cs6g14090	0.576272	0.961771	1.931775	1.795119	2.114312	2.270348	0.499094	2.409872	0.629376	2.799655
Cs6g14100	6.425299	3.343268	0.468591	0.710282	1.320828	1.475265	0.664238	0.170117	0.53664	0
Cs6g14140	37.84658	43.45588	30.22393	30.43346	16.35644	13.49514	10.11549	16.80864	9.828906	14.10657
Cs6g14160	5.31847	5.619	7.67409	8.300036	10.40603	13.50671	5.827846	15.50959	7.083996	9.469798
Cs6g14230	3.321002	4.880674	3.144906	5.426767	4.889618	5.441263	3.205962	4.680061	2.877708	3.513158
Cs6g14250	31.68933	29.88783	19.4352	21.32185	14.72431	9.273914	10.27079	7.257289	3.294393	3.639341
Cs6g14270	42.189	44.55985	30.09246	46.04953	5.763782	12.67209	3.170839	8.261863	3.197862	10.52645
Cs6g14290	3.441851	3.060978	2.308765	2.762945	0.752993	1.394072	0.667764	1.167697	0.793498	1.915637
Cs6g14360	0.712551	0.563694	0.251532	0.419913	0.727608	0.373082	0.831662	0.243409	0.508178	0.217744
Cs6g14430	0.408473	0.435199	0.927292	0.916046	2.987874	3.428762	4.25893	2.408438	5.008202	6.137798
Cs6g14440	0.478797	0.322281	0.693812	0.597818	0.675888	0.668414	1.717522	0.579894	1.174341	0.816634
Cs6g14460	41.73764	50.5695	29.92973	37.81359	17.7995	18.09938	8.393179	25.38167	14.75909	24.37521
Cs6g14530	155.2209	198.6907	135.927	161.863	63.15282	81.03453	109.6286	115.7259	174.6479	212.9303
Cs6g14570	0.118839	0.189151	0.202522	0.636294	0.965078	3.810055	2.469008	1.321308	18.34232	14.57899
Cs6g14680	2.868465	8.224109	0.569407	1.900367	2.163535	0.867075	3.877429	2.419419	0.146433	0.126298
Cs6g14820	3.196039	2.136417	2.810713	2.90652	2.354779	2.702694	0.73038	0.756825	5.873981	1.541254
Cs6g14840	24.85325	23.19927	20.7021	19.94842	8.356355	14.46465	1.238138	5.927479	1.955964	2.950848
Cs6g15050	1.079171	0.83867	0.468099	0.801494	0.270636	0.687123	0.561331	0.520153	0.585118	0.111735
Cs6g15070	0.736444	1.375538	0.716198	0.908448	0.356153	0.159953	0.038762	0.341652	0.017336	0.050353
Cs6g15080	5.368249	14.19912	1.53359	3.882765	1.207697	1.471881	1.850206	5.996961	0.572549	0.855535
Cs6g15100	7.941035	7.969295	15.07184	12.85512	26.83698	46.04154	26.18969	53.55586	50.36971	56.49498
Cs6g15130	353.1164	258.2363	76.83245	81.77399	51.75464	106.2236	77.89033	33.49981	128.193	178.3812
Cs6g15140	0.410818	1.010907	0.236859	0.246049	1.893611	1.754797	6.699614	1.314516	6.40425	5.373017
Cs6g15150	0.827906	1.305797	1.609345	1.805049	5.34144	6.228044	7.539886	5.396933	13.87889	12.29551
Cs6g15200	5.368823	6.249003	4.29714	5.149955	5.222795	4.885443	2.716073	6.096299	3.502178	9.747513
Cs6g15240	7.34665	6.163294	4.453726	3.37657	3.760158	2.972686	2.007113	1.659028	2.026791	1.398537
Cs6g15280	4.111414	2.247817	5.955407	5.16259	8.13816	9.906502	16.39322	9.910486	21.42329	13.85628
Cs6g15290	18.14047	14.18562	30.90874	39.95694	11.25644	43.23477	24.78538	27.10297	166.5259	268.2988

Cs6g15300	118.6466	90.69091	171.8926	178.692	75.78368	168.0496	61.17975	85.84858	80.32026	168.7913
Cs6g15380	1.913858	1.567489	5.773168	5.156924	1.353326	3.533627	6.132434	9.321446	0.155854	4.906909
Cs6g15450	3.458809	1.339995	2.783385	2.305754	6.994744	4.666409	9.228092	4.00722	22.9314	6.485603
Cs6g15460	98.96769	95.58852	172.9947	146.9652	232.3892	302.5896	92.60544	282.1488	241.2277	222.4894
Cs6g15540	176.1712	163.7982	265.6843	245.3239	514.0401	400.2426	943.8433	749.1284	559.0756	470.4812
Cs6g15560	22.89904	23.38245	21.85807	18.17263	8.598149	11.28209	4.616622	20.2872	0.210527	0.706602
Cs6g15590	2.930404	1.352833	1.026802	1.13734	1.91754	3.355056	2.32651	1.217299	18.59124	3.71625
Cs6g15610	36.6011	29.86981	24.82194	31.0666	20.30823	22.94041	11.60284	5.535089	0.741082	0.977173
Cs6g15710	12.03672	13.34563	8.793653	11.32819	3.143809	4.164729	1.957055	5.271383	2.756562	4.794597
Cs6g15730	3.530274	4.394373	10.33872	14.32192	4.000031	3.553282	6.362082	12.99952	12.1633	24.7618
Cs6g15740	36.53659	23.25852	56.0929	60.44344	76.65839	112.4428	98.64113	40.28783	295.6476	222.192
Cs6g15800	8.226169	12.1595	41.92741	35.05183	51.67263	47.52274	9.968941	26.48773	6.048636	12.9092
Cs6g15860	68.19265	80.68571	56.12498	66.40151	32.4774	29.97346	26.50599	50.27753	15.39751	24.88319
Cs6g15880	2.176971	2.338239	6.445445	7.23795	10.06033	15.97883	13.97401	7.290788	10.31777	9.912665
Cs6g15910	49.88305	64.60874	83.66942	73.84324	104.5027	99.36661	165.4837	253.0961	75.22637	146.5075
Cs6g15920	5.730842	5.233112	7.990889	6.4409	17.17007	22.81916	35.79167	41.91645	16.14028	17.73492
Cs6g15940	3.727878	3.818027	2.586623	3.386834	2.348342	2.93587	0.974503	3.127059	0.843216	1.274734
Cs6g16000	119.9403	175.3932	38.37322	76.646	24.81952	10.42974	45.16815	56.68461	29.68985	28.10551
Cs6g16020	0.1417	0.269973	0.868683	0.451657	0.808878	0.386027	0.403224	0.760007	0.213347	1.124536
Cs6g16030	16.74518	17.4267	7.155231	9.413837	3.926882	5.149749	4.569025	5.300015	0.988554	1.715832
Cs6g16080	0.481233	0.537859	0.731156	0.579418	0.809696	0.346071	0.260317	0.502921	0.544753	0.750355
Cs6g16160	2.515143	2.373911	6.655268	5.298377	16.32182	11.36464	4.496151	8.644223	0.511536	0.715462
Cs6g16200	75.68687	155.9889	24.75811	38.28609	17.73702	16.01349	12.47905	22.88077	3.498614	5.013628
Cs6g16340	0.805858	1.239675	0.110794	0.164738	0.023456	0.065934	0.090709	1.054525	0.013648	0.063878
Cs6g16350	134.2365	177.2647	89.77917	105.7806	39.82954	33.99607	51.53617	74.40313	39.06808	105.0384
Cs6g16400	0.274249	0.926649	0.113857	0.155454	0.06511	0.009397	0.058588	0.046977	0.0628	0.08757
Cs6g16430	31.75518	35.79949	25.51907	36.05423	12.48556	15.08678	7.931924	21.8241	5.612124	12.37071
Cs6g16440	11.78317	12.59629	8.671707	8.507148	9.497918	10.84775	3.144519	5.987003	1.161195	1.384743
Cs6g16450	7.285013	5.521536	3.691845	4.178552	1.83251	3.555916	0.610251	1.498572	0.297189	0.442035
Cs6g16460	8.604564	4.469124	4.002276	3.437426	3.086144	4.533189	0.627708	1.036429	0.531551	0.28412
Cs6g16590	2.412374	3.997868	3.734914	3.558388	2.079949	1.803224	1.809749	2.377994	0.079808	0.304787
Cs6g16660	1.498855	2.922817	0.563732	0.991419	0.103491	0.13356	0.053982	0.270199	0.199827	0.17229

Cs6g16685	9.681162	3.726849	9.900254	7.054652	7.235021	11.76409	5.199967	3.730495	14.62028	9.380258
Cs6g16690	9.407323	4.404078	10.47931	8.788363	6.878871	13.53652	4.188126	4.528374	21.63347	13.9832
Cs6g16700	12.45895	7.666611	26.17396	13.52953	31.32209	19.7726	15.83625	13.07148	4.606723	2.226283
Cs6g16720	5.696413	2.697806	5.23741	4.922479	3.681634	7.783817	2.211609	3.187302	11.17174	11.36702
Cs6g16730	71.31022	75.88126	73.36904	57.04342	83.51003	34.37285	87.38486	39.52314	16.93466	16.52509
Cs6g16780	41.88263	52.42178	21.44477	31.1721	7.022288	9.138609	2.669799	9.092452	4.756149	9.420025
Cs6g16820	20.80424	21.72285	14.58589	12.44081	21.8348	16.50861	14.04583	11.36675	8.790926	3.893112
Cs6g16890	10.68325	11.71341	11.75647	10.38007	4.039072	3.876561	2.342526	3.963708	1.282817	1.239467
Cs6g16980	9.265594	9.379588	18.55312	18.50879	36.10701	90.63806	34.74539	42.296	242.9491	122.2405
Cs6g17080	10.4108	7.14318	22.36264	15.20521	38.51581	31.10553	53.30327	18.72886	12.79913	10.34603
Cs6g17100	4.278073	4.78796	4.391096	3.724214	2.964263	2.204503	1.532345	1.94193	1.097056	0.386686
Cs6g17170	17.42413	19.24938	5.423899	8.732528	2.420677	4.624115	1.25489	2.411908	0.625405	0.284999
Cs6g17190	24.12708	14.73612	93.76899	55.50566	25.20718	44.67269	3.158318	9.059707	0.336977	0.248267
Cs6g17200	52.19317	123.6342	15.85841	35.91347	31.72932	24.48091	26.06698	45.05134	8.589239	8.551738
Cs6g17340	3.319758	4.219702	4.800854	6.218128	5.084378	7.252061	6.289066	21.37889	15.20882	6.020997
Cs6g17410	136.9956	181.5133	83.18322	114.4538	15.92975	37.80678	24.4873	94.57104	59.44951	211.1547
Cs6g17510	44.23144	33.99536	39.78701	46.09144	17.52802	29.41258	23.72115	10.59252	13.63008	3.106125
Cs6g17530	11.00441	11.52284	7.56043	5.514314	3.561124	4.011222	3.829915	3.06148	1.514806	0.768772
Cs6g17590	66.02747	97.02089	64.89025	80.17092	9.024545	18.52085	32.66603	74.86885	42.7088	128.561
Cs6g17610	1.38703	1.649438	0.539861	0.789733	0.47001	0.389429	0.383967	1.252019	0.467094	15.68534
Cs6g17620	12.57669	10.98246	10.21668	7.98482	9.797838	9.152956	5.023746	13.66298	1.243795	0.424066
Cs6g17680	59.44748	54.46081	42.48573	44.51924	49.20344	43.27167	32.9677	14.09464	29.75769	17.76684
Cs6g17740	101.0908	89.34592	60.78085	54.52006	75.76409	20.43707	14.67335	14.22408	4.759494	7.839956
Cs6g17750	26.06356	23.23521	25.9581	27.17002	28.08722	52.30361	7.736277	12.15426	2.934863	2.389641
Cs6g17760	92.56682	78.61499	64.13208	69.68672	48.60364	74.22863	25.43643	31.49563	62.63177	51.95518
Cs6g17885	0.225971	0.445013	0.485764	0.420639	0.760739	0.943131	0.356095	1.046155	1.050018	0.717635
Cs6g17950	11.08015	7.840821	3.346184	3.422523	0.890954	1.299892	1.19714	1.679306	2.646274	2.297693
Cs6g17960	5.424697	1.956133	1.259429	1.139984	1.015576	0.832153	0.545568	0.371526	4.68288	2.711108
Cs6g17970	1.012479	0.311232	1.149368	0.973215	2.854861	3.921565	2.796714	1.385478	2.435072	0.634769
Cs6g18060	6.499046	5.644436	7.225874	7.522399	3.101359	3.397036	2.02461	4.259899	1.024817	1.199668
Cs6g18090	26.65145	31.38905	25.86405	30.50307	4.614774	7.206264	9.413475	53.55261	32.85545	74.23881
Cs6g18110	1.971348	1.35227	0.968213	1.109033	0.560537	1.519177	0.652484	0.815076	1.173648	1.223161

Cs6g18200	9.16421	10.69085	5.269363	7.281453	11.75859	5.896126	0.184011	0.904641	2.014899	1.292662
Cs6g18240	9.43772	12.12561	27.77353	31.09313	17.88413	23.4993	15.92216	37.99419	12.94712	14.12389
Cs6g18250	16.36144	18.27639	65.88197	59.08769	86.98019	132.4693	76.41649	133.1119	102.1565	88.65447
Cs6g18260	1.652476	1.660915	2.45967	2.257416	4.149833	4.201497	5.402326	7.084077	9.755575	7.932261
Cs6g18320	1.765144	1.359754	1.027263	1.076333	0.395994	1.440314	0.526707	1.336235	0.060884	0.032693
Cs6g18330	1674.733	1538.563	1389.137	1108.051	836.4759	924.2068	325.814	724.519	12.02753	10.23283
Cs6g18340	1475.614	1297.764	1448.499	1485.515	2066.458	1507.188	1374.234	1804.184	83.86523	210.6433
Cs6g18360	3.648986	6.678659	16.86669	39.8667	1.595685	6.539489	3.458361	18.44488	33.08871	112.7151
Cs6g18370	8.276147	8.158512	9.001311	9.33525	6.020231	8.100052	1.849321	8.704978	8.733381	9.845873
Cs6g18380	48.9937	42.64919	57.7539	69.25686	14.89182	23.78877	32.53226	48.11812	117.8926	217.245
Cs6g18390	6.989609	7.217924	8.683232	9.088494	5.895363	6.196619	4.436928	7.297562	1.223871	4.195717
Cs6g18400	0.017046	0.030059	0.261033	0.322149	0.625079	2.091949	0.087077	1.071139	5.867358	8.942287
Cs6g18430	4.054788	4.079867	11.57599	11.45588	4.465439	7.390073	4.068963	7.655493	6.587682	15.98613
Cs6g18540	0.829227	0.645487	2.060442	2.368132	0.316335	0.674836	0.521575	1.911154	0.008109	0.222463
Cs6g18580	20.12066	30.64399	13.179	25.63104	10.63208	16.66225	21.72229	23.46016	7.635847	22.44066
Cs6g18640	38.19265	30.76842	39.43048	54.60914	21.86314	37.84634	52.71335	110.5973	22.66719	35.5137
Cs6g18650	7.185398	8.336793	12.72727	15.27195	35.19805	30.90358	41.79225	49.43263	34.54299	72.35662
Cs6g18660	54.21789	54.01077	76.57137	63.4409	53.60977	52.93151	31.28312	69.45891	56.41128	83.05866
Cs6g18680	1113.779	1602.183	288.8207	751.6919	25.91067	26.64137	16.23648	38.3324	15.22997	6.866916
Cs6g18760	1.80701	2.258402	2.344188	2.540515	1.365983	1.18454	1.150131	3.651552	1.726638	1.351174
Cs6g18850	17.40038	28.53218	7.756051	9.482025	4.09411	4.484332	1.614648	6.127727	0.903882	1.186563
Cs6g18890	10.24033	9.241725	8.032825	7.92391	7.918548	9.074911	3.73758	8.439174	8.968851	5.88847
Cs6g18900	124.6014	87.36309	200.9406	167.8335	288.509	239.0587	245.8594	171.9542	527.7605	317.2904
Cs6g18920	22.79497	29.40659	16.21366	17.59401	3.749366	4.243579	1.970758	4.167698	0.388324	0.365345
Cs6g18930	2.323167	0.748382	0.18543	0.186269	0.006805	0.053095	0.124401	0	0.065521	0.00758
Cs6g19060	3.001807	2.192741	1.764459	2.040074	1.251416	2.148607	1.068293	2.369202	1.920049	2.255181
Cs6g19130	8.474243	7.685388	12.65988	12.63087	30.79414	22.11035	51.97304	64.35069	59.46491	75.03723
Cs6g19180	4.140818	7.078147	2.286653	4.094093	2.108052	4.6298	12.76494	11.45399	4.21361	6.955269
Cs6g19240	0.146273	0.017671	0.250814	0.238474	1.099203	0.252741	0.372502	0.186934	0.832665	27.86449
Cs6g19280	11.82968	52.95813	0.984898	6.710932	1.302522	0.70342	3.034527	7.088323	0.48269	1.137967
Cs6g19330	0.216521	0.42438	0.504643	0.453577	0.825693	0.706957	0.255009	1.421232	1.083347	0.885087
Cs6g19340	3.30675	5.404269	21.79269	18.53568	2.012205	3.92807	1.29986	3.156311	0.107389	0.49132

Cs6g19380	70.44892	70.86004	59.39541	57.28866	47.64078	28.30867	36.23561	158.023	52.51994	173.0951
Cs6g19600	1.243728	2.544478	0.795128	1.596324	1.052653	1.750431	0.490859	1.770878	0.026435	0.034723
Cs6g19630	0.44814	0.414725	2.915443	1.963686	6.854245	4.846013	2.466147	8.543728	2.798217	2.271869
Cs6g19640	13.29523	14.9508	38.9201	24.12891	111.2121	63.58843	87.0015	64.23375	42.58297	65.9911
Cs6g19670	9.598094	11.18416	12.97858	15.78595	7.989279	8.783056	5.001472	8.965498	0.802794	2.424817
Cs6g19710	5.896364	8.018923	5.251833	5.627201	1.416176	2.693892	0.805596	1.511232	0.455334	0.89134
Cs6g19760	1.324275	1.869794	2.895348	1.577026	1.810895	3.685537	5.555551	16.20163	2.110191	6.012801
Cs6g19790	6.083479	6.054056	2.599657	2.662711	0.843709	1.613831	0.286349	1.430506	0.129727	0.086443
Cs6g19830	13.83668	12.10375	13.56523	16.78188	8.774868	14.35007	10.13509	14.2861	22.5414	52.45068
Cs6g19940	56.42966	83.58315	20.5258	25.33825	36.92746	17.24028	19.09824	16.64721	16.56997	9.298279
Cs6g19990	1.112244	1.24536	1.791838	2.06872	2.149973	2.94578	0.681524	1.562764	0.547399	0.958214
Cs6g20030	67.87636	61.87057	45.85224	38.0582	98.89003	78.33749	136.5189	118.1109	21.29731	9.245826
Cs6g20150	2.287767	2.948698	4.176129	5.139986	2.567916	3.305956	2.732546	7.154994	6.884014	17.92133
Cs6g20170	6.357973	5.71808	7.124626	6.175758	5.807359	8.824252	4.517079	23.92479	1.696417	6.502763
Cs6g20180	0.103252	0.012134	0.311822	0.260183	1.090816	0.704435	0.13518	2.359707	0.099238	0.279046
Cs6g20220	9.761492	5.509874	2.520694	2.361776	0.732648	2.908934	0.694191	2.760861	0.155239	0.179472
Cs6g20230	20.1924	22.86063	26.70901	22.73443	23.41085	21.87093	20.12687	45.41678	3.267797	5.820997
Cs6g20270	28.65898	22.51395	54.94305	32.53663	57.4467	27.15878	12.72981	7.148256	2.744288	1.039268
Cs6g20280	3.613877	4.655312	1.74896	1.871865	0.700037	0.377032	0.24086	0.166134	0.058879	0.009051
Cs6g20290	0.051487	0.070261	0.058851	0.008313	0.01945	0.034002	0.116464	0.008595	5.125323	1.558267
Cs6g20340	2.211508	1.97352	5.361215	4.441052	11.64452	14.18145	14.34342	9.771718	21.045	6.29523
Cs6g20470	5.263486	5.707769	22.4303	20.05553	25.02473	25.7727	12.36154	16.28278	13.05909	16.62663
Cs6g20490	57.45976	21.91288	40.13389	47.51116	89.81808	165.0148	118.3767	70.99942	976.4211	472.0031
Cs6g20550	5.42792	7.535003	7.289174	7.095293	6.398459	6.575483	4.7812	11.42593	2.130835	5.372106
Cs6g20620	0.363572	0.696683	5.469959	7.734803	5.88239	14.71207	17.05145	26.2557	8.442305	14.93231
Cs6g20830	7.742426	5.758957	3.924861	4.720825	8.427427	9.578106	16.45113	24.39242	49.67657	34.4645
Cs6g20910	30.92584	34.14343	47.84199	48.20754	31.83799	51.63308	23.61267	52.20795	23.89282	34.6576
Cs6g20920	2.509647	2.08318	7.853865	7.913766	1.349102	3.918619	0.499099	4.128197	0.549062	1.665304
Cs6g20930	2.421944	2.949107	7.976375	10.02514	1.813088	5.236949	0.407203	3.771675	0.853821	3.304991
Cs6g20940	0.314055	0.388818	0.638167	0.669469	0	0.120423	0.124738	0.895219	0.225021	2.309669
Cs6g21000	6.428804	6.581529	3.961631	5.501406	1.99721	2.990383	1.155989	2.284902	1.350908	1.647501
Cs6g21070	1.544435	0.625579	1.957677	1.466664	0.656837	0.632968	0.105259	0.132914	0.388829	1.153753

Cs6g21080	21.45578	11.37322	25.39077	10.94887	10.646	2.760019	2.665678	3.687758	6.492894	9.563954
Cs6g21110	5.143486	3.829144	6.874305	5.891651	7.739231	5.810817	11.62181	6.793125	9.472096	1.784342
Cs6g21120	5.570788	3.096102	6.353404	4.258984	1.01635	3.640747	0.2988	0.60103	0	0.054938
Cs6g21160	2.574331	2.783227	2.115379	2.67072	1.579324	2.121908	1.274758	3.212678	1.314256	2.285743
Cs6g21200	20.2509	13.78212	25.34209	20.60314	19.90416	51.51456	23.71	29.30034	132.6884	65.9146
Cs6g21210	21.45775	22.17009	42.74429	41.97078	75.22077	113.7029	91.82314	126.6814	139.4523	102.2826
Cs6g21230	18.48144	25.60139	15.24285	15.07939	10.66217	7.809481	9.017609	6.296264	4.625299	6.144802
Cs6g21290	2.466519	12.87627	1.939598	2.324957	0.605992	0.549018	0.188747	0.718375	0.486419	0.578503
Cs6g21320	17.71684	36.31045	77.2705	147.6231	3.566853	8.546764	21.83453	45.1292	79.61059	130.2085
Cs6g21330	0.762591	1.231136	2.600994	3.564694	0.363042	0.890835	0.33871	0.695747	1.514769	0.986822
Cs6g21340	1.295919	3.233815	1.475972	1.353749	1.375568	0.29852	3.095102	2.034977	0.247518	0.20111
Cs6g21350	0.387435	0.424953	0.928948	0.753916	0.914124	0.759151	0.878306	2.061479	1.592889	3.353983
Cs6g21390	10.54884	13.03017	19.0891	16.32056	30.55036	24.40951	29.97714	33.49434	26.87476	21.46786
Cs6g21400	11.23253	29.05099	3.114453	6.923411	10.53215	7.102511	18.00892	15.79379	9.011093	7.23203
Cs6g21410	0.608738	0.783305	1.048027	0.841389	0.770124	1.227396	4.797738	10.56395	1.98363	5.120277
Cs6g21420	146.6954	307.9196	576.039	741.8775	70.91301	134.8483	120.2762	438.21	18.81026	160.0275
Cs6g21430	0.725137	0.43292	0.992999	0.818406	1.315719	1.131868	2.512666	0.701024	1.068593	0.918787
Cs6g21460	21.14094	34.91247	53.58857	41.58604	61.21557	138.7744	106.197	164.361	3.162698	2.553478
Cs6g21530	8.702753	10.65536	6.770378	6.260142	2.714364	8.812107	1.901624	8.922669	1.331948	0.385414
Cs6g21560	1.277996	1.60131	1.069172	1.090791	0.335884	0.605212	0.168063	1.123417	0.270343	0.146832
Cs6g21570	13.38665	13.95997	16.08427	15.54061	15.44956	16.93114	11.58199	24.89393	7.96703	7.916176
Cs6g21600	5.390599	10.15001	4.429179	5.659286	4.199252	2.658166	2.32577	5.427274	0.301941	0.630579
Cs6g21610	23.80316	34.36351	28.96836	31.9441	31.17793	35.97434	21.28207	46.59293	22.89504	22.56437
Cs6g21690	0.887804	1.146088	1.168283	0.908044	1.046216	1.253285	0.642851	0.602086	1.581284	0.46645
Cs6g21790	8.210193	8.568301	7.910815	9.558109	5.53079	9.633191	4.727546	12.01685	7.977978	12.4295
Cs6g21820	21.83404	43.22211	8.050472	15.35241	9.524154	7.982872	12.56555	24.08747	3.932925	8.792025
Cs6g21840	0.97388	1.449102	0.140156	0.177437	0.222319	0.054855	1.001453	0.170325	0.513522	0.247784
Cs6g21850	16.58643	20.58886	5.743562	6.695027	32.26641	22.95226	115.6683	102.2679	15.78305	15.97156
Cs6g21860	1.773506	1.610936	2.530884	1.991527	2.787176	4.075426	0.942759	1.83558	0.596355	2.328672
Cs6g21900	9.69988	12.45187	12.68578	6.405666	16.81813	11.58328	21.61784	10.01906	15.37699	12.18672
Cs6g21920	7.86983	26.79173	1.49711	3.309124	1.627629	0.526275	0.73976	0.976505	0.36487	0.286549
Cs6g21990	28.64981	19.91146	13.19321	20.32333	15.62376	16.61911	39.5448	17.64357	9.311514	23.08663

Cs6g22000	0.03417	0.041818	0.030612	0.04032	0.121442	0.062684	0.108732	0.118058	0.050753	0.951908
Cs6g22010	8.295888	8.626923	4.320844	4.48788	3.995054	3.803674	2.842564	3.716219	1.340465	4.483278
Cs6g22050	124.1262	94.71187	109.8461	117.7695	89.41579	164.3173	126.392	112.7989	330.4821	276.6114
Cs6g22105	4.500932	3.054539	2.890462	1.852263	1.95244	1.82069	2.924108	1.122827	1.724356	0.547147
Cs6g22150	14.65609	24.43119	11.0138	16.93084	42.55142	35.17346	34.67164	40.23726	0.453455	0.388873
Cs6g22210	10.69939	13.1645	9.214124	11.26796	4.359793	5.629739	1.945681	4.767927	1.421201	2.66933
Cs6g22230	33.19241	34.50124	22.13487	26.59656	10.77182	10.39014	12.06737	13.42206	12.84789	30.5128
Cs6g22270	2.974906	5.835478	2.220158	3.434452	2.380467	1.578205	1.96368	4.090871	0.749335	1.670251
Cs6g22300	2.9677	4.130941	4.150861	4.2317	4.458439	4.814774	3.037955	8.51897	3.291839	9.572844
Cs6g22320	14.92186	12.92677	20.11591	21.08413	19.05374	25.42986	5.394291	10.2867	9.531337	11.76393
Cs6g22360	14.17039	13.88869	12.34093	10.02795	4.813952	6.215749	2.909227	8.297873	0.044817	0.286654
Cs6g22390	49.71197	55.46437	30.90482	33.55438	3.917618	9.449141	0.639033	9.727099	0.539791	0.270262
Cs6g22400	16.45589	18.08107	20.82453	22.15243	24.20776	27.84602	10.14029	23.74955	15.00332	16.94478
Cs7g01030	4.6256	3.6082	4.569643	4.064164	3.904055	3.477262	14.96607	4.984896	18.8557	13.91583
Cs7g01140	173.2904	189.3404	184.7078	171.8803	126.0446	83.61934	40.54952	79.5975	8.880599	25.04622
Cs7g01180	22.95957	24.06319	28.14457	25.58422	13.03647	16.2309	5.058908	16.36639	3.608281	7.307352
Cs7g01190	45.93984	58.18421	79.31709	76.13764	85.87841	59.4691	38.91002	91.8491	25.9068	49.41226
Cs7g01250	2.196554	2.601752	1.348921	1.276825	0.417731	0.672784	1.174903	1.372248	1.194147	1.623684
Cs7g01260	3.585213	3.441725	1.54978	1.662468	0.686242	1.265244	1.716225	1.970803	1.734297	2.069889
Cs7g01300	1.474636	1.37464	3.084258	2.46396	0.78202	1.456394	1.397344	2.231277	0.535336	2.666253
Cs7g01320	5.97803	6.311037	9.453818	9.09653	14.34812	9.675656	12.49398	4.531243	4.896489	5.38959
Cs7g01380	19.81331	19.22827	16.80058	15.82474	8.425397	5.981753	6.495164	10.24042	3.25552	4.158155
Cs7g01390	3.041266	6.696523	7.146606	8.441694	0.427663	1.591738	4.152476	15.79931	3.246319	17.01163
Cs7g01400	2.246369	2.633249	6.270885	6.101001	5.188608	15.04759	8.893191	12.96722	101.1748	50.34503
Cs7g01430	22.66242	24.48188	30.89851	27.00888	23.54687	28.43722	15.196	35.82031	13.55678	19.33892
Cs7g01530	5.738123	4.324387	3.89652	3.933347	2.988835	7.678499	1.537123	4.11405	0.344839	0.212042
Cs7g01700	13.5287	16.77606	7.475611	9.735183	1.455063	2.301069	0.735775	1.646625	0.227119	0.259985
Cs7g01840	70.54413	51.5543	101.7863	85.46767	188.6982	183.6127	215.6279	95.38097	287.5553	221.2065
Cs7g01855	6.34473	6.162834	6.93888	5.879555	6.59497	8.612537	2.198637	9.465767	7.828865	5.643941
Cs7g01870	6.802542	7.775186	5.932684	4.816016	8.466616	5.351359	3.435516	3.268743	0.377115	0.423439
Cs7g01900	5.467865	4.930223	5.788931	3.386948	9.284419	9.027682	12.12661	35.69224	19.47902	27.25324
Cs7g02010	62.48043	32.61089	45.79741	47.2622	31.43669	81.89794	38.83918	39.81186	275.9928	203.6642

Cs7g02040	70.47445	171.4042	35.0095	61.14028	28.68855	34.29272	45.49609	91.7502	20.93411	35.38138
Cs7g02050	9.028551	16.31293	19.44765	25.23993	4.077519	11.52446	7.192659	29.65927	34.45781	129.4584
Cs7g02060	7.907817	4.017432	6.441515	7.599755	1.984894	5.512275	3.933181	2.983047	11.95632	8.998733
Cs7g02090	32.52897	46.39	43.19208	43.78681	24.32453	23.96683	14.65284	43.85153	11.84586	21.8824
Cs7g02240	86.85703	69.54141	187.7121	140.4408	141.4178	204.3555	226.9893	239.9397	677.0963	585.2338
Cs7g02320	0.793087	0.261595	0.33684	0.245442	0.263275	0.376781	0.242602	0.202801	0.50362	0.347651
Cs7g02340	13.48609	29.4069	40.76407	13.4288	0.088452	4.555351	4.623551	32.61719	9.073125	12.13387
Cs7g02360	2.938626	3.118631	3.383636	2.962161	2.897968	4.86573	2.187787	3.220142	0.679879	2.24033
Cs7g02380	0.369405	0.277833	0.672912	0.645108	0.969078	1.858328	0.98279	1.304849	5.646052	3.661355
Cs7g02500	21.05804	14.84521	36.17141	18.93811	131.5323	68.53197	38.08164	47.03539	5.549979	4.61407
Cs7g02540	0.100714	0.108391	0.106012	0.036173	0.551898	0.37775	2.229469	2.621243	7.354159	7.39365
Cs7g02570	60.96487	38.76312	79.76529	56.20777	76.21143	58.23268	98.54342	53.60101	105.0512	34.89293
Cs7g02620	14.09362	25.80571	29.97608	31.98454	3.937454	5.752246	9.610088	27.63745	4.724749	41.24392
Cs7g02660	28.49931	37.18181	98.85949	80.49984	56.25103	71.78728	10.95962	19.41548	9.676307	16.74955
Cs7g02670	155.0202	189.1131	698.8874	528.1452	536.326	486.3075	73.2788	55.42347	28.42332	50.16498
Cs7g02680	17.85392	21.69997	75.74759	67.44745	50.88633	56.86714	8.227973	9.261216	3.68078	6.94498
Cs7g02690	2.126596	4.88714	0.464883	0.769871	0.122698	0.180835	0.012198	0.587237	0.039965	0.192559
Cs7g02740	3.104072	3.135434	0.480122	1.506825	0	0.112532	0	0.179663	0.119168	0.163831
Cs7g02820	371.9444	325.615	435.3311	509.4391	79.94397	241.5575	242.0195	271.8204	1296.249	1666.397
Cs7g02830	1.142033	1.418491	9.582983	7.864816	1.254075	3.28583	6.095782	14.46946	20.15268	51.99365
Cs7g02840	14.24498	14.13734	35.70799	24.40676	20.13289	14.54685	23.09604	27.91683	36.8942	49.46938
Cs7g02850	123.9876	108.6591	128.251	118.5986	64.27962	48.35776	71.56061	102.4315	178.9035	160.857
Cs7g02860	1.127754	1.502509	1.227566	1.67395	0.297047	0.531729	0.299411	1.132662	0.669696	1.065634
Cs7g02880	3.866872	1.336166	7.246912	4.002378	8.686966	7.935784	3.031692	1.850555	3.603878	1.09048
Cs7g02920	4.658155	12.36947	1.791861	1.682589	1.041276	0.178016	0.274291	0.597492	0.08326	0.134334
Cs7g02950	6.727944	8.275209	23.06699	26.09019	17.65496	67.13884	10.23028	33.83954	12.99284	29.01131
Cs7g03070	1.254594	0.915747	1.953839	1.437237	2.688757	3.47034	2.507566	2.663085	2.444796	2.052617
Cs7g03160	9.064747	9.592688	10.12332	9.946324	26.3359	28.19787	22.50275	30.09556	17.53815	14.44903
Cs7g03180	63.88578	74.33357	48.37532	65.79095	24.52351	20.66111	15.07617	38.41986	14.67511	25.45604
Cs7g03210	0.662848	0.589069	1.427184	0.861809	1.552829	1.113284	2.558572	0.773648	1.140305	0.941267
Cs7g03240	17.28825	23.82379	27.9776	24.62423	13.07482	18.00392	6.352728	26.44375	1.802355	2.238622
Cs7g03250	0.293582	0.474135	0.358988	0.586564	0.249374	0.60737	0.394695	1.166723	0.150695	0.062257

Cs7g03310	0.528193	0.211701	1.46916	0.462336	0.659856	0.880511	0.361309	0.85301	0.432964	6.381324
Cs7g03320	12.13308	12.17269	7.574208	8.851947	3.656483	6.080443	5.34677	6.309355	3.4679	3.198643
Cs7g03390	0.176129	0.018052	1.553278	0.257553	6.545813	5.632647	1.013597	0.758126	0.170836	0.087462
Cs7g03460	11.29771	12.0346	7.367411	8.177789	5.012682	5.197205	3.661066	5.480601	1.474361	2.803801
Cs7g03525	2.154195	2.968991	3.972762	3.755506	4.178555	4.517329	1.697074	4.490415	1.680604	1.854769
Cs7g03530	17.74985	17.89775	17.19013	17.2011	7.519139	7.949703	3.348835	5.344554	3.906041	6.919493
Cs7g03580	21.75965	29.5076	6.505127	10.8523	1.298855	1.104323	0.205651	1.805086	0.296841	0.494704
Cs7g03630	31.99925	29.62484	203.6247	57.23612	220.3823	281.4706	143.923	223.3404	4.085157	8.918626
Cs7g03650	4.594392	2.840465	4.892973	3.286948	7.082675	7.426601	14.96238	6.786119	7.939856	5.257968
Cs7g03660	0.400612	1.563895	2.761158	3.388765	1.256548	1.261873	1.710773	1.592652	1.118547	0.603347
Cs7g03670	0.822923	4.995504	0.195289	0.451108	0.717108	0.483691	2.340167	2.497234	0.233772	0.236299
Cs7g03710	0.312402	0.463881	0.444483	0.356919	0.971843	0.276702	1.603371	1.461885	3.06875	4.557487
Cs7g03770	54.85071	22.75219	18.46322	20.19733	17.78644	89.0784	14.37335	15.21672	89.56655	22.65266
Cs7g03800	19.79932	15.7148	18.21514	20.27453	4.132582	10.99741	5.523874	12.47057	18.01738	60.13877
Cs7g03810	2.163356	1.136049	0.857256	0.511629	2.393612	1.793505	1.019192	0.456902	0.686686	0.051906
Cs7g03820	5.469425	4.365005	6.230241	4.720979	6.893465	8.566564	5.679575	4.570926	16.39478	6.628055
Cs7g03830	10.58941	6.832351	19.52419	12.23651	29.93722	28.14599	15.02781	9.177001	17.68929	6.345026
Cs7g03900	0.98596	0.948556	0.984636	1.660177	0.564896	1.230625	0.391401	0.65508	0.761756	0.844105
Cs7g04110	1.179074	1.746734	2.003666	1.877066	0.447389	0.772411	0.106761	0.908757	0.095969	0.119991
Cs7g04120	10.42751	20.31072	3.408079	4.464329	3.040555	2.401481	2.675422	3.23683	0.199445	0.232935
Cs7g04150	11.84192	14.71828	21.24971	27.81616	8.045364	10.85808	11.14704	15.18899	23.07037	40.23178
Cs7g04160	0.233518	0.294194	0.263786	0.246829	0.265449	0.290196	0.839476	1.421887	0.673808	0.878017
Cs7g04210	9.722877	11.78164	10.94879	11.2239	9.299879	9.999026	5.439925	12.28925	3.642551	3.199098
Cs7g04240	10.46696	23.46706	12.57939	10.33367	22.2215	13.55952	12.58461	19.54568	5.869694	8.103964
Cs7g04380	0.022292	0.052299	0.061032	0.053805	0.209903	0.077609	0.877571	0.124477	0.979765	0.815635
Cs7g04390	6.444569	4.44755	21.8749	15.90497	54.1637	50.17659	78.50489	25.035	92.83522	55.52904
Cs7g04520	0.119449	0.152145	0.151831	0.257971	1.62423	0.918007	11.677	1.748695	2.029023	1.19125
Cs7g04540	33.91484	36.18731	69.83786	64.51549	45.46294	39.32305	63.58807	68.57416	42.78015	81.99444
Cs7g04580	34.43615	32.18324	43.36208	43.76384	50.64895	79.15201	126.987	52.79074	61.668	51.00956
Cs7g04600	261.2361	216.0429	373.0811	259.0429	429.1603	340.6744	1008.597	434.9418	188.3794	130.5558
Cs7g04680	0.651882	0.678905	1.572139	1.585386	0.964954	1.893413	0.869552	1.997603	1.438343	1.593522
Cs7g04700	2.219824	2.153957	2.402609	2.096421	1.261844	2.055093	3.000782	4.451557	9.224534	6.154027

Cs7g04820	70.28745	79.58391	73.63286	75.70716	52.73853	50.33031	30.2492	83.51142	19.86998	41.04561
Cs7g04860	2.600899	1.932814	2.463757	1.565597	2.153874	1.121231	6.006001	6.144603	1.978059	9.094715
Cs7g04900	16.73841	20.63369	12.72858	11.00814	1.458969	3.64358	1.828298	4.889258	0.333205	0.556069
Cs7g05090	18.5848	8.849172	9.987482	13.59452	14.26577	23.25552	12.74428	23.29264	5.538996	3.012652
Cs7g05120	4.263609	6.393314	4.520979	5.068011	4.117341	5.097693	2.751732	6.878563	3.119511	2.901931
Cs7g05130	0.04203	0.194554	0	0.057555	0.117582	0.42353	0.126941	1.068126	0.764539	0.903506
Cs7g05180	25.91779	12.11949	13.89386	19.47211	22.14661	35.00546	18.34929	36.40628	9.752147	4.234717
Cs7g05200	0.534567	0.484086	1.303478	0.680792	0.647762	0.806084	1.65107	0.908575	1.699991	1.47822
Cs7g05210	0.74602	0.709271	2.803411	1.646256	4.438849	3.1517	8.460114	5.402666	0.185804	0.299749
Cs7g05320	3.139234	3.510997	4.440015	4.617252	4.776758	4.436072	2.210191	5.306439	0.647701	0.855435
Cs7g05340	28.42298	26.45843	42.94869	50.05659	59.46336	98.94	56.1101	92.53704	38.21232	29.55414
Cs7g05360	80.27	52.99272	22.57859	21.83204	12.10259	18.305	59.6497	43.57299	2.5689	0.245889
Cs7g05410	17.12534	9.69272	47.25809	40.77289	68.32373	94.70152	101.0486	92.89437	79.82245	86.61895
Cs7g05420	6.036326	6.054344	9.494725	8.873134	11.90665	15.07148	15.06911	21.65686	15.86173	17.78677
Cs7g05440	4.425411	2.313861	4.986069	4.552337	6.754597	8.670954	8.990169	6.057331	17.70791	12.57063
Cs7g05460	24.26279	31.57235	24.59939	40.17063	1.626862	3.769568	17.37564	42.06344	28.2179	144.1361
Cs7g05470	1.609217	2.402334	2.027177	2.357048	1.155965	0.899589	0.490925	1.190023	0.354941	2.88096
Cs7g05500	21.53388	23.13258	13.2073	10.54307	8.929612	9.305465	13.62756	12.08046	25.17496	19.01449
Cs7g05510	12.68985	8.881112	45.66055	31.12754	58.58168	66.74015	17.0126	20.74861	13.99323	26.18643
Cs7g05520	1.928148	2.252098	2.508896	2.453806	2.478646	3.837383	1.461923	3.84884	1.629008	3.584003
Cs7g05540	1.380534	0.44461	1.277114	0.527459	1.087115	0.393141	3.201034	1.251129	0.033796	0.026229
Cs7g05570	53.72274	72.2702	32.27574	47.7859	25.83757	21.9476	28.43313	33.21961	5.2359	8.457282
Cs7g05590	28.29632	30.86387	22.21953	19.53188	14.44301	14.69345	9.342764	13.24646	5.768739	8.061389
Cs7g05600	4.684782	5.964458	5.302672	5.599728	2.570036	3.642535	1.335127	5.772977	1.768298	2.046436
Cs7g05920	127.3584	108.6959	28.05844	40.25945	3.311711	9.818736	12.78003	28.37106	26.38623	38.65703
Cs7g05970	20.38596	32.03792	24.93153	33.28141	8.498941	8.612219	6.874643	14.71618	6.605673	26.16852
Cs7g05990	11.22916	16.02012	5.315474	8.381273	3.775652	3.850528	4.618327	6.523922	1.421076	1.345798
Cs7g06030	48.14077	42.18546	135.9904	103.6965	230.5509	353.2597	83.43744	179.3876	13.07053	7.962136
Cs7g06080	3.470967	4.745667	18.10179	17.9893	59.21841	50.66386	112.5634	105.8415	43.93611	28.1676
Cs7g06120	2.767901	3.805436	3.566855	7.761752	1.398323	6.422174	25.10424	132.5716	6.856145	97.06526
Cs7g06130	3.967261	4.056926	5.774076	7.444397	1.077749	2.687873	5.911723	16.07766	8.810998	16.61938
Cs7g06270	27.90205	29.92785	40.87989	33.98624	50.57384	27.11405	34.92761	37.26017	18.80523	22.16034

Cs7g06285	1.80314	2.193099	2.302841	2.563016	1.568833	2.390691	0.955407	2.769571	2.374936	5.696354
Cs7g06310	16.58364	21.80565	36.40841	28.71049	18.93346	8.08768	7.913526	9.593878	13.01312	15.78887
Cs7g06330	44.47379	25.79209	51.46592	48.81047	93.07899	102.075	14.21181	29.18473	151.9988	276.7482
Cs7g06410	1.116649	1.219952	2.526068	2.318676	1.761221	2.655437	2.851056	3.911756	2.53718	3.179677
Cs7g06470	6.751032	7.483709	5.968118	7.375627	5.682242	7.72056	3.308496	7.786881	7.133833	7.078822
Cs7g06550	7.772918	4.758105	8.000477	2.761094	6.584663	5.819373	1.509568	9.104629	0.511368	0.972157
Cs7g06580	2.933072	1.717078	2.638516	3.770699	0.807683	2.551367	1.542058	1.473271	1.311288	1.221985
Cs7g06690	14.21616	29.54616	6.594829	13.7125	12.03141	8.117251	29.11698	23.36184	7.55725	7.41036
Cs7g06720	0.83423	1.045916	1.632308	1.330298	1.208668	2.401773	1.782304	4.74599	0.848253	0.417158
Cs7g06840	72.58842	97.28482	39.37606	54.61585	20.61762	23.63772	10.4319	17.76073	2.071936	3.775231
Cs7g06910	5.126429	5.502524	4.379858	5.19963	2.923314	4.19965	0.932977	3.064654	1.031093	2.092268
Cs7g07060	2.329672	2.232901	6.079061	6.33359	10.36522	13.84684	10.95485	12.95749	1.468059	4.452394
Cs7g07070	0.704389	0.675033	1.632915	1.238389	3.439417	3.070723	2.495866	3.213768	0.347612	0.652898
Cs7g07120	5.973903	5.30883	57.06267	21.20275	27.00111	12.42626	40.81977	13.16788	1.561319	2.539904
Cs7g07160	33.07766	49.73593	29.60392	37.9025	21.2374	20.58545	17.64353	35.45573	13.32741	55.842
Cs7g07240	0.746243	1.755962	0.822341	0.734942	0.075833	0.18853	0.470934	4.346591	0.337721	1.173074
Cs7g07300	9.991041	7.805048	8.802808	7.845777	17.76331	16.41067	28.73317	13.02624	29.37725	5.951501
Cs7g07320	5.882039	6.446242	18.68283	20.75332	2.921059	6.792941	2.815812	11.71251	1.181073	6.716688
Cs7g07340	2.228546	2.24964	7.315337	8.3426	1.208066	2.893037	1.218791	4.510983	1.196259	3.713213
Cs7g07420	7.745146	8.296997	7.257049	8.120425	3.516702	3.673372	2.901278	3.729539	1.849624	2.675087
Cs7g07450	6.489848	12.6093	4.443278	6.849848	8.008386	4.242316	9.496253	10.85425	1.083548	1.68882
Cs7g07640	183.5276	274.1382	192.8226	404.8083	53.59625	138.3901	107.9964	181.9748	535.3515	1363.118
Cs7g07730	4.614614	7.084949	5.228494	6.990161	3.083733	4.201162	2.238305	5.099711	2.12469	5.799998
Cs7g07800	10.75528	13.03579	9.796048	12.23783	7.606334	7.418686	3.401116	8.885579	2.93814	4.851705
Cs7g07860	4.748551	3.12688	5.541463	4.517208	2.62876	5.259053	2.581571	4.252792	1.426784	1.301433
Cs7g07900	22.10149	29.63592	19.55147	19.52437	7.225637	5.178355	5.760991	22.28192	5.886072	13.40333
Cs7g07930	0.139804	0.021026	0.076544	0.043681	0.154815	0.289656	0.376986	0.226094	2.048722	0.35425
Cs7g07980	1.05593	1.278096	2.69366	2.601881	1.098061	1.635443	1.50278	5.58679	13.29699	38.8066
Cs7g07990	27.89533	33.54341	39.83471	49.37658	5.21327	11.03862	24.1887	41.67051	19.96702	55.29847
Cs7g08070	0.000665	0.089979	0.450823	0.721197	0.038562	0.372233	0.201231	0.363782	0.558253	2.333536
Cs7g08080	12.18883	14.14613	30.80984	49.75051	7.112541	19.36263	8.943458	14.38322	41.4142	86.41673
Cs7g08100	0	0	0	0	0.024814	0.098461	0.15548	1.315867	0.060672	0.030297

Cs7g08110	8.474281	5.283333	4.372393	4.035753	4.836938	8.528834	0.623343	0.951199	3.336425	0.261257
Cs7g08120	3.411395	2.30569	10.14411	7.537564	16.24083	16.3942	23.23179	10.88961	9.746046	10.25674
Cs7g08180	17.29015	12.34445	20.72255	14.24121	8.463352	7.761875	3.402795	18.435	1.048771	0.537581
Cs7g08190	19.16672	18.86064	18.42087	23.5171	6.102429	7.591398	2.977983	4.408766	1.438667	1.97788
Cs7g08220	1.741594	2.63426	3.073324	4.747813	1.078402	2.295186	1.879478	6.911507	0.806285	0.564518
Cs7g08270	0.799439	1.212118	1.098668	0.984751	1.227784	1.135309	0.400115	1.244389	0.527783	0.868376
Cs7g08330	5.818916	7.99721	2.545136	2.746357	0.461491	1.20309	0.417702	1.159623	0.057928	0.018749
Cs7g08380	8.576516	9.942929	5.48524	6.380838	3.413144	4.324983	4.293338	5.097437	0.754039	0.92883
Cs7g08460	2.420324	1.742285	1.829182	1.397251	0.274039	0.609182	1.033498	9.297827	0.144331	0.310779
Cs7g08470	95.51313	42.14739	29.76582	17.26508	5.97055	4.115549	6.069165	9.208395	7.038729	3.516242
Cs7g08550	52.2421	50.66103	40.48897	58.69636	17.56573	26.34465	21.30303	17.97214	9.325053	7.925517
Cs7g08590	10.57734	11.01387	17.58671	16.26165	11.07821	11.85126	4.885224	13.79666	7.097597	12.95793
Cs7g08600	56.38481	52.35164	20.17815	23.02113	13.73052	20.64018	37.86909	54.38184	56.39127	62.25833
Cs7g08620	91.68015	62.70633	87.99004	100.2597	102.6363	123.3647	320.5746	96.80572	170.2946	49.55976
Cs7g08650	0.566176	0.542542	0.564904	2.084219	0.703938	0.662073	0.589097	0.46864	0.089756	0.161885
Cs7g08680	9.38408	33.65165	11.28082	12.96631	2.315453	5.62764	7.98994	24.30116	3.953006	9.91498
Cs7g08750	10.41825	10.59715	15.29395	12.35681	13.47853	14.33035	10.00627	22.05055	8.265391	9.38284
Cs7g08810	13.56299	15.48923	7.515145	10.32028	5.469742	5.984626	2.489499	6.891901	1.713758	4.03043
Cs7g08840	65.71566	84.15675	85.36687	96.08318	47.20864	62.08492	22.40492	78.8823	55.16509	140.8239
Cs7g08890	1.90724	1.456311	3.522686	3.604728	3.955913	4.335561	1.213151	1.777876	0.091142	0.240345
Cs7g08920	6.864295	6.465784	7.614757	5.390772	14.50702	12.98614	23.30398	10.12086	6.46934	5.384947
Cs7g08960	16.47791	11.12272	8.758583	8.213008	16.03886	25.50663	35.86445	10.57015	42.13716	11.18581
Cs7g09000	7.157304	8.946692	5.139461	7.049651	4.131046	4.194189	2.245218	7.272813	1.671217	3.211823
Cs7g09020	3.25712	2.836504	5.107909	4.407096	4.138194	5.312841	1.963878	4.466419	2.790108	4.66156
Cs7g09040	24.36978	15.64432	43.19793	38.6954	58.08482	71.986	86.65886	44.15017	20.24123	8.937128
Cs7g09050	1.647414	0.597754	0.720173	0.818645	2.058717	3.107211	2.281174	0.249304	1.044827	0.30316
Cs7g09060	60.69237	40.23323	69.09913	58.56454	134.7244	129.0655	129.6062	79.1768	105.4701	63.84446
Cs7g09070	0.509663	0.303297	0.504828	0.299573	0.590872	0.717152	0.235614	0.451686	1.759124	0.397121
Cs7g09080	14.62112	15.68755	16.09462	13.69737	10.09169	11.35523	10.64607	22.85144	0.427288	1.093893
Cs7g09100	66.51005	80.39443	73.70416	96.59356	40.29434	47.48695	93.26538	109.1669	170.108	313.6784
Cs7g09120	10.11016	22.249	4.422843	9.632347	6.180878	3.849144	5.921399	11.53939	2.580386	2.825854
Cs7g09200	37.13056	27.17717	68.85349	53.34615	37.47784	36.39979	13.41895	15.9126	1.783007	2.291318

Cs7g09260	5.339195	6.545053	11.26761	11.76988	11.68889	9.362993	5.901602	12.24473	3.850601	0.94595
Cs7g09380	5.759993	8.08446	5.727213	4.949806	1.256595	2.29585	0.647553	1.919463	0.07149	0.187045
Cs7g09430	0.043583	0	0.02081	0.053141	0.09135	0.201198	0.40576	0.24208	0.242714	0.962287
Cs7g09440	25.08598	23.61575	19.06156	18.78232	5.282223	8.637064	5.612192	11.44524	25.11933	19.64273
Cs7g09560	18.2129	10.49729	13.53314	14.69586	11.7867	22.2791	11.98514	11.61075	21.46385	13.03169
Cs7g09590	21.54525	21.74493	28.67106	33.45882	20.87744	23.45241	23.26716	18.80358	7.632213	21.76597
Cs7g09630	28.80752	34.83069	105.2422	81.10593	165.1698	159.5758	239.3482	199.8371	48.9224	73.06748
Cs7g09660	6.948543	5.944332	6.19945	6.6294	2.978937	6.405092	1.908362	3.414132	3.663718	4.424904
Cs7g09680	6.012527	5.047871	13.95823	9.094139	22.71645	8.488549	12.88098	9.941833	8.272916	12.86964
Cs7g09760	12.23256	14.17118	9.523784	8.191064	6.573712	8.624966	4.921894	8.059646	3.742262	3.578431
Cs7g09850	2.568083	2.848369	3.425944	2.98806	6.118531	6.514773	6.189433	8.13468	9.268473	8.449409
Cs7g09860	110.9292	59.94802	21.7772	14.21438	12.97164	35.84578	36.94091	45.2622	1.884001	1.241287
Cs7g09900	75.58252	119.855	25.67693	39.32651	6.98312	9.548086	1.737513	7.572832	0.98286	1.078278
Cs7g09963	3.787827	4.220838	1.226634	2.291619	0.787978	0.960264	0.60196	0.603574	1.1278	0.635785
Cs7g10000	10.49887	15.66744	4.277834	5.865564	0.760139	1.490194	0.596927	2.782542	0.113498	0.160923
Cs7g10010	3.350062	2.862561	3.890067	3.583565	5.830704	5.238588	2.867961	7.727054	3.716131	2.386742
Cs7g10040	5.458523	6.410034	3.610131	4.18999	2.628713	2.141976	10.13386	9.76405	49.20369	23.66451
Cs7g10050	16.81643	13.00735	20.05436	16.2878	28.90649	22.55682	51.97193	18.38607	83.3243	57.92669
Cs7g10080	2.633316	5.174735	5.623081	7.540526	4.69919	6.384994	4.718344	8.360485	11.70752	18.25665
Cs7g10110	34.98014	29.46889	70.54684	59.35093	74.46119	69.04873	15.10515	10.65872	22.4528	13.15567
Cs7g10200	0.198919	0.590115	3.013166	1.83718	4.221444	6.597993	13.79821	12.92711	0.087165	0.179526
Cs7g10220	48.15588	43.49518	77.53383	66.61192	142.2856	118.8345	231.1863	172.5584	131.5109	95.38751
Cs7g10340	0.80407	0.840418	2.053016	1.360813	2.712361	1.774908	1.648071	1.813674	1.36296	1.104796
Cs7g10350	2.530786	2.543	4.151494	4.413659	3.376584	3.583435	0.971293	1.818333	1.820111	2.49084
Cs7g10390	3.355462	3.086518	5.369684	5.401682	8.161586	9.938684	9.908919	13.85262	6.855035	8.217508
Cs7g10510	5.904251	4.39509	4.947016	3.089541	5.776899	3.784278	5.893058	1.736729	5.401495	1.826167
Cs7g10570	0.546385	0.621996	3.885846	5.639586	0.667415	2.035682	8.279687	9.364169	2.770726	23.1798
Cs7g10620	4.638815	3.59088	4.44421	3.917071	2.21192	1.949805	0.387618	1.414935	0.111032	0.703107
Cs7g10710	39.53872	93.43066	32.76151	48.43914	132.9334	76.36208	218.5904	243.9062	86.59254	110.5549
Cs7g10720	15.35419	15.07023	11.64617	10.53332	6.954763	7.514309	6.004645	3.917102	14.20702	3.975882
Cs7g10740	69.66918	161.5278	15.928	31.49371	5.935785	10.35229	3.833552	9.361882	3.337285	2.322803
Cs7g10770	5.493552	3.648162	6.594929	5.884849	7.377154	15.48055	5.48898	6.827592	15.24022	11.1184

Cs7g10790	11.55732	6.389033	1.658473	2.981484	1.948347	5.089889	1.305041	1.7303	2.197951	1.757591
Cs7g10880	12.48807	10.05466	6.247116	5.792731	4.774313	5.999487	1.899007	2.909067	4.881174	2.954815
Cs7g10940	3.310262	3.887363	1.356619	1.316666	0.443345	0.284831	0.246106	1.567209	0.338086	0.629981
Cs7g10950	30.65804	33.43193	20.30144	22.69745	11.3135	14.2116	5.802819	14.55772	11.3457	14.92802
Cs7g10980	921.4335	1080.382	2089.051	1941.587	1990.077	2269.225	3161.852	4503.314	2215	3533.358
Cs7g10990	10.57324	9.347861	9.627019	7.986509	6.492515	8.530829	6.485267	8.511245	0.35869	0.876289
Cs7g11030	25.33108	25.95397	41.85634	39.26692	60.81863	61.75413	40.42343	59.82579	53.53049	68.25731
Cs7g11170	10.78855	6.712936	10.85155	8.027764	12.33127	15.2804	34.85717	23.24845	56.43871	51.81796
Cs7g11190	0.483883	0.9011	0.868782	0.449614	0.973708	1.532103	0.819349	0.860457	0.865078	1.381011
Cs7g11210	14.67815	11.84064	2.181785	4.287189	1.106109	2.409585	1.308468	2.094016	9.117388	5.860572
Cs7g11310	0.485918	0.536904	0.927677	1.705112	1.413088	1.685888	2.042629	2.571257	10.06737	8.462054
Cs7g11320	2.176368	4.482264	2.254791	2.155861	1.68264	1.380626	1.754237	0.217939	1.189474	0.352416
Cs7g11380	1.460798	1.975584	1.676242	1.479521	1.930825	2.184241	1.121398	3.146114	0.865044	0.886822
Cs7g11415	0.994435	1.086615	0.649227	0.637037	0.863818	0.891508	0.269099	2.378788	0.637487	0.686765
Cs7g11560	14.53749	13.66641	19.90795	13.34079	17.54554	9.259729	11.45886	8.947326	11.18794	3.956445
Cs7g11570	2.020969	1.466318	2.259762	1.401756	2.565269	4.907772	1.165889	1.150093	14.50436	5.556612
Cs7g11620	296.1712	339.689	297.8342	361.1092	110.8674	169.5305	48.58491	167.7364	51.32786	119.6433
Cs7g11770	19.7532	21.87203	11.55763	11.85976	3.688611	3.432414	1.548019	1.592752	0.071174	0.115436
Cs7g11820	0.181782	0.306981	0.157185	0.34492	0.47861	0.976878	0.409894	2.036897	3.928816	5.113718
Cs7g11860	12.32362	11.63385	18.22316	17.01854	10.07788	17.64549	4.81185	19.06871	8.694707	17.36571
Cs7g11890	1.833393	2.092266	2.914429	5.931026	0.41597	1.095899	0.428857	2.280588	0.19066	1.349801
Cs7g11950	0.471285	0.665201	0.93733	1.03709	0.507005	0.519477	0.475502	1.308265	0.103248	0.290632
Cs7g11970	6.71999	6.261116	17.64162	13.43369	22.33104	24.28861	28.44372	36.49506	20.95142	21.84229
Cs7g12100	0.993721	1.064225	12.19857	13.42438	6.276946	14.78909	6.034959	6.556698	21.66517	24.09416
Cs7g12185	6.886474	7.448424	9.036913	8.516011	6.085371	10.04331	3.39174	10.4922	7.618517	8.135462
Cs7g12250	5.405447	5.849177	4.196575	4.37541	2.194939	2.583657	0.957653	2.137228	0.488711	1.290138
Cs7g12320	2.758796	2.562035	1.788848	2.223242	0.952415	0.963945	1.129387	1.152485	0.555578	1.065474
Cs7g12420	9.862483	7.587181	5.279279	4.776115	6.011126	3.917774	1.564078	3.22877	0.166144	0.040443
Cs7g12440	19.92892	13.03538	11.44889	7.310901	15.01609	5.751455	9.013514	9.525868	40.47633	8.935438
Cs7g12510	10.79418	8.455873	9.949829	8.488271	5.622468	5.199225	1.969594	7.594562	2.787184	4.697686
Cs7g12540	15.06088	13.04577	26.2279	28.4573	13.6906	17.20521	16.87638	25.27002	21.16565	69.94243
Cs7g12560	0.715833	0.656212	0.841152	0.945909	0.860035	1.270379	0.515658	1.287682	1.260192	1.419137

Cs7g12600	0.142418	0.465853	0.770953	0.607069	1.68563	1.087575	2.973424	2.59377	4.330691	8.182527
Cs7g12620	0.032395	0.062362	0	0.009119	0	0.010546	0.075776	0.052332	0.949546	2.277589
Cs7g12650	0.951394	1.196892	3.166696	1.827885	3.846725	1.574766	4.677989	2.649334	0.615489	0.703478
Cs7g12690	26.92254	24.83994	11.97339	13.69302	3.911837	4.554485	1.402972	1.801528	0.252054	0.069848
Cs7g12720	2.4051	1.602474	2.664036	2.909058	2.702232	3.722899	1.036673	2.673585	1.500075	0.976772
Cs7g12780	2.790986	3.079941	3.226843	3.309769	1.625916	2.484729	1.119	3.963581	2.306959	3.07888
Cs7g12810	11.23588	10.48206	11.97606	10.00349	7.653888	7.945574	4.318583	13.65972	4.545855	5.358809
Cs7g12820	3.068879	4.326683	2.347167	4.857032	0.568262	1.89318	0.080602	0.873255	0.048771	0
Cs7g12850	23.56801	55.96842	5.939407	18.3325	8.645861	6.73725	8.789809	13.786	2.080234	4.114743
Cs7g12870	114.5651	88.52417	45.17679	52.73444	19.88366	47.88948	19.38198	33.96824	18.52994	48.09895
Cs7g12890	191.2524	119.7593	339.4382	317.7221	335.9449	394.6731	430.6157	225.994	796.3486	677.0063
Cs7g12910	5.737435	8.379529	4.143716	4.279033	2.271528	6.885862	1.116548	4.090866	4.183714	8.090529
Cs7g13120	4.120781	4.021352	5.04505	6.144983	0.954695	2.727504	5.244587	11.07462	9.169282	22.47897
Cs7g13150	1.447317	1.156351	2.766679	3.100213	6.6785	7.157681	5.87918	11.81267	1.163568	2.770294
Cs7g13170	11.17621	8.922771	10.03052	10.2592	10.6707	15.11513	24.17984	20.08275	29.87044	23.01605
Cs7g13180	0.411286	0.659765	0.898239	0.900706	0.374531	0.693147	0.514829	0.803712	0.703343	2.017836
Cs7g13200	193.5944	136.2662	58.96599	69.54555	46.86251	95.3633	43.74221	52.5363	6.34236	0.730362
Cs7g13230	7.006692	11.15095	47.30354	31.2132	34.67181	47.60771	5.983739	3.160645	0.359673	0.156468
Cs7g13250	62.61782	31.69973	30.76158	25.6254	14.62908	18.6932	14.08291	7.543143	1.861318	1.053321
Cs7g13290	1.223358	0.607652	2.651097	3.058461	0.147969	1.300882	1.66466	1.961448	1.057918	1.823556
Cs7g13400	1.178205	0.158462	0.181738	0.213848	0.334318	0.610233	0.213797	0.329697	0.79219	0.271971
Cs7g13500	8.689559	9.829088	11.32159	19.73187	1.917231	8.403703	5.537211	12.76455	7.694773	19.69347
Cs7g13740	6.115216	5.876378	4.643037	4.883392	3.507398	2.736911	1.310455	1.405044	0.591543	0.409965
Cs7g13790	2.699388	2.865747	3.956877	4.604761	12.86523	10.94482	26.58663	20.33255	44.07572	63.53553
Cs7g13810	1.721946	0.900801	8.215507	2.589948	54.53404	60.08107	49.0614	37.69923	73.34698	99.45052
Cs7g13830	0.503468	0.791058	0.571293	0.457694	0.66671	0.410141	0.480982	1.142591	0.660826	0.631457
Cs7g13920	7.899391	7.817212	13.20776	11.51141	19.42957	14.99878	11.52748	18.24331	10.24327	16.28466
Cs7g13970	13.4101	13.7975	9.937513	11.31567	4.393789	4.855315	3.605342	5.157313	0.789438	1.023954
Cs7g14020	102.6122	98.81358	212.823	195.5924	225.846	220.3719	326.4596	328.8689	185.5304	271.5148
Cs7g14170	1.188604	0.743403	0.815363	0.440618	0.724191	0.319306	1.296667	1.597526	0.743407	0.332363
Cs7g14180	1.564399	1.947926	1.620631	1.905148	1.468279	1.681595	1.425981	1.497784	1.368394	3.5661
Cs7g14270	4.222255	2.955326	2.860845	1.709545	2.688716	1.460796	4.20169	4.396937	3.282448	1.045777

Cs7g14420	62.00988	66.75862	65.71451	67.37284	39.76612	45.23827	22.88151	32.64075	7.095752	12.52361
Cs7g14430	27.25015	34.63451	26.81222	37.70946	4.620222	13.88926	1.339912	4.160496	2.87402	3.139291
Cs7g14440	8.862719	12.01685	10.56465	10.84396	10.48213	11.44803	4.796906	13.91021	3.830276	4.67009
Cs7g14460	71.31965	76.13154	98.42649	86.82275	129.6143	130.7949	131.6787	77.46267	81.93015	36.00049
Cs7g14470	6.936235	7.955552	9.075998	8.994626	19.00191	17.09346	23.28122	17.66034	20.21754	22.08089
Cs7g14480	0.55094	0.738978	0.886841	0.390725	0.875622	0.590103	0.330776	0.501925	0.824218	0.618577
Cs7g14510	3.89978	3.524095	1.799998	1.392533	1.273588	1.755571	1.3392	2.469945	0.799718	1.102355
Cs7g14590	0.689787	0.396325	1.447983	0.90594	1.322055	2.139663	2.274279	2.231101	2.717323	2.158631
Cs7g14610	0.324249	0.254231	0.882855	0.505048	0.367011	0.799108	0.569662	0.645296	0.813111	0.878044
Cs7g14910	2.7892	3.161764	5.907384	4.751674	8.027578	8.534284	4.38096	11.7226	11.25311	14.19914
Cs7g14940	0.114038	0.223374	0.322638	0.328996	1.022971	1.558418	2.498167	1.162706	12.0832	2.689943
Cs7g15000	26.07376	28.06009	32.2135	39.45628	16.12109	18.48321	8.909767	24.17037	7.588386	7.552111
Cs7g15050	2.211399	1.319198	1.602457	1.805985	0.36785	1.44383	0.187277	0.65909	2.926564	4.853119
Cs7g15080	6.957165	8.075839	4.161525	7.275014	1.512525	1.275221	0.164975	1.117205	0.172949	0.087165
Cs7g15285	3.724834	2.38554	3.133749	2.09183	3.578934	2.669157	2.151634	2.426218	1.55836	0.649596
Cs7g15290	19.48059	30.26818	8.662571	14.98503	15.82816	11.86691	16.22437	17.94271	15.42369	22.68594
Cs7g15300	0.481874	0.930919	0.605181	0.816667	4.925731	4.535883	3.65453	11.43693	1.995048	3.285896
Cs7g15320	31.89726	27.6471	48.33092	54.16766	98.24642	191.3077	18.68533	8.559917	7.683064	7.987317
Cs7g15360	4.057861	7.218111	2.160836	2.93748	3.48035	2.406928	2.205677	3.950929	1.061155	1.498553
Cs7g15460	25.18213	15.94808	20.97715	17.17462	31.50374	30.6339	39.81336	17.35076	33.94432	11.73845
Cs7g15480	79.34383	59.74066	151.0071	91.12129	111.6691	138.7864	63.24998	76.24473	0.976242	1.791725
Cs7g15500	0.547769	0.575689	5.292986	3.707574	13.84944	9.648132	13.72059	15.1083	4.338372	11.63564
Cs7g15610	72.11012	53.17024	151.7956	212.0136	32.68571	82.02054	54.67353	30.70929	148.8532	77.31973
Cs7g15640	0.914028	0.518897	1.821496	1.111453	0.522975	1.937249	0.150713	0.569791	0.488736	0.557648
Cs7g15670	9.512312	11.19899	14.65538	13.83945	11.79862	15.40429	5.388967	13.17958	6.396064	10.52736
Cs7g15730	6.475221	8.782986	8.058122	9.969523	6.103799	8.094386	2.779436	9.619586	1.918028	4.836952
Cs7g15750	3.025651	2.716922	1.773762	1.551867	1.118915	0.941491	4.828696	2.00441	0.037409	0.013157
Cs7g15760	91.45783	91.98507	391.9683	347.1516	289.8708	318.6713	78.21299	198.4424	221.057	253.6845
Cs7g15770	14.29493	10.12874	28.13474	19.69695	16.228	18.17209	3.665283	7.306555	15.34805	12.74741
Cs7g15790	0.974508	0.734101	3.064722	2.569074	1.060996	1.403246	0.844315	4.277225	1.512246	1.882061
Cs7g15810	6.616779	5.23214	16.64046	9.088011	14.06151	17.03668	0.193344	0.317334	0.609814	0.271772
Cs7g15870	5.008801	3.065664	8.765231	6.184381	7.720577	8.720563	2.436887	5.40297	5.070987	2.730872

Cs7g15950	73.40576	61.54424	26.59834	27.70717	8.235656	18.91201	7.803923	15.40393	64.27804	88.56746
Cs7g15970	4.479778	7.918833	2.24776	3.494348	2.749654	3.385042	2.598688	3.225587	0.678118	0.438149
Cs7g16020	148.9959	164.9964	148.4181	206.6043	25.21929	94.22004	36.71818	123.9182	229.0986	657.5965
Cs7g16030	95.55718	145.5326	62.29089	92.16652	15.73002	24.63061	46.84564	69.85031	75.39488	193.5944
Cs7g16060	3.703455	4.614497	14.20967	11.56364	17.42038	14.18375	15.4859	13.67153	9.87655	12.38633
Cs7g16090	22.47813	22.33057	45.22782	37.36074	66.35846	52.76212	52.6368	45.29968	87.18472	54.02239
Cs7g16220	55.64149	46.52133	82.93997	72.75416	52.75356	42.42713	17.13352	13.82319	4.6126	4.155813
Cs7g16230	37.98792	33.61877	56.2734	49.4591	38.21765	31.85322	15.77473	10.02531	3.703206	3.885762
Cs7g16240	64.10558	59.75402	94.85622	96.08893	60.41963	60.14007	21.49051	20.64227	5.776523	7.138755
Cs7g16250	8.567953	5.558636	3.831023	4.859507	2.286232	1.904027	0.599708	1.7708	1.192773	0.167732
Cs7g16260	45.4194	41.15141	30.2635	43.13072	24.52846	20.65977	17.96289	29.386	28.804	23.16981
Cs7g16390	0.503127	0.523213	1.818492	1.149323	2.389937	2.283357	3.307788	2.358535	1.50624	2.57582
Cs7g16400	1.41139	1.833734	1.213633	1.399167	1.07915	1.250744	0.851196	1.81598	1.183615	0.496879
Cs7g16430	10.53739	16.47081	11.64298	17.44778	23.01399	10.3164	10.92187	10.62225	0.521349	0.680692
Cs7g16510	4.996887	5.07505	2.835349	2.546847	1.479718	1.199028	0.667252	3.507833	0.721665	0.705199
Cs7g16520	6.763453	6.44644	2.99042	3.034124	0.455534	1.009129	1.30204	2.317417	0.110304	0.415118
Cs7g16525	8.115021	10.44731	8.886581	7.831143	6.80855	9.419464	4.193125	11.5122	6.318202	5.820056
Cs7g16660	5.928223	3.49511	4.351636	3.915006	2.833941	7.575367	5.201973	11.40252	22.34804	49.67933
Cs7g16720	50.57005	57.17959	22.4476	27.87732	15.56905	17.0116	17.78932	27.52926	13.19015	38.18517
Cs7g16760	19.23679	20.02696	46.08861	41.08412	13.63033	17.47281	22.31307	29.666	18.42311	36.47519
Cs7g16830	5.923871	7.789628	8.034259	6.386756	10.06269	4.286754	10.27663	5.791295	0.334804	0.369215
Cs7g16840	10.09659	5.598198	11.65088	13.23547	8.009511	21.85459	10.69178	17.01609	8.45502	20.87037
Cs7g16870	0.266618	0.337239	0.771224	0.557351	0.451574	0.610948	0.1106	0.959497	0.035029	0.025194
Cs7g16890	2.681727	1.971133	1.393036	0.841514	0.235434	1.384632	0	1.461084	0.101609	0.054461
Cs7g16900	10.52436	11.53467	4.858509	8.444548	5.80492	3.084517	3.02022	2.091196	0.730516	2.218445
Cs7g16980	46.73677	32.58839	32.9572	27.77105	38.39762	41.99581	7.835874	10.3253	3.933176	1.731358
Cs7g17020	1.605632	1.988274	5.13438	3.605098	4.145749	3.839055	3.785083	4.423335	2.743745	2.760322
Cs7g17040	0.211367	0.163766	0.494211	0.471327	2.81086	1.841608	2.969021	1.825948	1.307551	1.22509
Cs7g17090	0.036037	0.030252	0.039307	0.043578	0.018022	0.030719	0.971751	0.213533	0.007743	0.022656
Cs7g17200	2.846098	3.713044	1.499325	2.489344	1.574432	1.942439	0.157106	1.504636	0.071416	0.109734
Cs7g17270	2.33202	2.32868	2.70036	3.147081	2.999582	5.132334	1.341519	3.825909	5.171879	5.857707
Cs7g17275	1.900546	1.532255	2.24699	2.311602	2.692427	3.696547	1.532443	4.045538	4.267513	2.739315

Cs7g17370	0.350356	0.92911	2.306987	2.618015	1.962891	3.168718	0.707446	3.314527	0.456592	0.542186
Cs7g17375	1.367524	1.145945	0.730533	0.746629	1.314994	1.114326	0.560334	1.527486	0.547068	0.369039
Cs7g17390	0.910395	1.574538	4.189696	6.403234	4.237693	2.984213	9.50539	13.34657	20.02622	24.36088
Cs7g17450	2.631588	3.240361	2.386067	2.467763	1.503444	2.410514	2.859851	6.868934	1.665293	2.358427
Cs7g17630	0.278542	0.603266	2.092408	2.808306	2.475442	2.994549	1.551775	2.911991	3.576394	1.533228
Cs7g17670	180.3725	146.1923	158.0221	162.9759	72.62603	124.3478	18.75382	40.03817	12.91363	12.33833
Cs7g17700	3.847576	3.916908	10.28207	7.461718	10.42418	9.885349	9.233897	12.38901	8.088271	11.14026
Cs7g17710	9.956865	8.633494	31.7161	26.62905	40.67684	29.7629	18.07756	44.64615	7.185405	14.43943
Cs7g17740	0.409476	0.514677	1.710208	1.239208	2.389797	4.046452	3.131878	2.935642	2.734422	3.327391
Cs7g17750	0.196407	0.295809	0.416956	0.637504	0.716991	0.754207	1.205957	2.378982	0.82132	1.045622
Cs7g17830	2.594128	2.402889	3.735499	2.858172	5.329942	4.973192	2.179658	5.179983	2.650379	2.717473
Cs7g17840	7.026746	6.572226	8.943645	8.917043	10.66131	12.38008	5.515406	13.14455	8.737191	8.21935
Cs7g17880	24.2921	17.11384	28.74136	35.05927	20.02738	36.51847	19.62438	20.66362	55.59961	106.2649
Cs7g17970	0.649205	0.559406	1.469605	1.048208	1.622182	1.639104	0.93131	1.640323	1.486421	1.368087
Cs7g17995	3.936336	6.966691	1.880779	3.869458	3.467834	2.86734	3.758289	6.287648	2.421007	3.205229
Cs7g18040	0.185555	0.174775	0.228847	0.289121	1.413266	0.8837	1.710051	1.158289	2.558093	1.45179
Cs7g18050	5.269292	3.715028	8.79482	8.406023	5.257273	9.102164	1.931888	6.040576	0.835383	1.746664
Cs7g18060	6.346501	3.465921	24.74558	13.13671	18.01701	11.34995	2.844231	12.56053	0.995517	2.775609
Cs7g18080	2.794646	1.782828	1.739699	2.461352	2.504142	7.623348	11.16725	7.975931	1.325886	0.475488
Cs7g18090	1.744894	1.051564	1.494469	2.23587	3.119504	6.421329	1.216359	1.727973	0.383777	1.154507
Cs7g18110	2.008507	2.747407	2.122387	3.228023	2.117269	3.729308	1.284874	3.598017	2.234656	3.233533
Cs7g18120	2.135933	3.723272	3.520833	3.555622	2.969853	5.496083	1.873588	5.019343	1.896307	3.096749
Cs7g18130	1.683532	2.38123	1.613128	2.30538	2.767645	8.215981	0.075388	0.652556	0	0.057764
Cs7g18140	1.833533	1.275883	1.752241	2.094776	1.814672	5.132624	0.342573	1.118686	0.378692	0.98303
Cs7g18150	2.879604	2.585836	2.315155	2.780531	4.899201	10.22459	0.218528	0.832863	0.067646	0.03519
Cs7g18190	0.655706	0.306877	0.583378	0.854003	1.14955	3.017585	0.151536	0.429166	0.091672	0.187307
Cs7g18230	19.48579	18.91881	22.94895	18.79815	12.22802	15.26358	5.027317	16.88186	2.739669	8.385136
Cs7g18240	6.258372	4.042653	16.40348	10.87514	11.25172	9.622603	2.247592	10.84573	1.136137	4.80023
Cs7g18270	2.86854	2.607073	2.931538	3.607805	5.687137	9.564625	0.328467	0.950604	0.015602	0.090887
Cs7g18280	14.34316	10.17823	10.6832	12.31116	11.45118	25.48132	2.745139	6.009085	1.095371	3.475087
Cs7g18290	0.12431	0.062639	0.446961	0.313064	0.539233	1.313111	0	0.257709	0	0.026714
Cs7g18330	6.605025	4.494417	5.278695	3.079413	31.23245	12.16138	74.25568	19.75691	29.00432	11.62424

Cs7g18430	8.869246	10.47481	14.60477	12.33692	27.98496	24.08016	21.84715	27.0101	34.68157	24.83207
Cs7g18460	29.7122	22.58398	19.87331	16.80597	8.872098	10.79948	7.126679	2.890454	1.375126	0.745176
Cs7g18580	1.150156	0.954474	2.46583	2.342547	1.171333	0.78563	1.524828	4.355276	1.050725	0.495132
Cs7g18675	2.029485	2.194606	2.505553	2.743881	3.629287	2.858869	1.993828	4.131088	3.263176	3.338641
Cs7g18780	151.7138	215.9102	64.54855	90.69744	24.69511	31.8199	14.91115	30.72511	11.11953	21.73084
Cs7g18930	0.311814	0.134456	0.236584	0.129101	0.827783	0.780882	1.877558	1.202933	0.690521	0.063099
Cs7g18940	3.911245	2.710815	4.084926	3.155853	12.49768	10.01503	25.92182	13.95188	10.97414	1.282955
Cs7g18960	5.728286	4.197278	3.588138	3.103846	3.495751	4.444807	3.393862	8.862955	5.288258	4.609619
Cs7g18970	9.995721	12.11108	16.2619	16.36196	40.56052	30.06746	67.47997	217.5699	27.25935	125.3971
Cs7g19050	2.581318	2.872339	3.173788	2.959973	3.007113	3.920696	1.276251	3.821431	5.159159	5.755525
Cs7g19120	0.51302	0.508501	1.254878	0.988205	2.206762	1.866363	0.992037	1.653585	2.001223	2.217611
Cs7g19160	8.724762	9.086224	21.72948	24.53838	20.23864	29.68301	21.02254	23.92773	23.86742	58.22026
Cs7g19180	0.037033	0	0.281011	0.244811	0.815207	1.303171	1.974978	2.71185	0.202517	5.264437
Cs7g19210	11.53294	7.138841	16.43057	13.3342	19.26843	31.75182	3.013002	9.461743	1.460771	0.718226
Cs7g19280	6.62867	5.627185	6.380663	5.659157	8.654476	7.037805	11.64933	5.083022	10.65331	5.335117
Cs7g19420	122.3348	136.8431	83.89242	76.30548	60.80127	28.26979	10.29879	38.47657	8.744356	13.3755
Cs7g19460	39.8867	38.90052	59.70036	52.60051	82.69279	57.88116	119.2805	87.14559	55.38325	84.51295
Cs7g19500	32.25671	26.91076	31.2008	27.79361	28.83774	41.47266	20.10231	69.48482	32.3563	32.43001
Cs7g19640	6.218141	4.356336	2.810892	3.745918	3.191077	5.647121	17.5149	11.44022	95.28842	55.96349
Cs7g19690	1.007244	1.041375	1.137035	1.03703	0.972493	1.013918	0.387109	0.617389	0.1967	0.90636
Cs7g19710	19.72547	20.4934	12.1498	14.16264	8.173416	10.65984	3.430673	5.040829	0.576831	1.242866
Cs7g19720	44.21081	42.4862	16.96845	27.95827	3.644969	9.389009	0.545235	3.353629	0.144931	0.215479
Cs7g19770	109.2485	102.6222	79.44842	80.15387	55.30402	57.00794	52.89396	33.30462	41.96557	32.10233
Cs7g19830	0.547437	0.775874	1.86518	2.760816	3.598459	6.341039	5.537683	5.93988	13.22035	17.39469
Cs7g19870	6.899668	4.19323	18.43507	15.66639	56.2716	74.27804	98.06084	137.1486	67.57042	39.8394
Cs7g19890	9.536335	11.27287	13.39709	9.505761	14.05218	10.79278	15.80428	9.458756	10.66149	4.344928
Cs7g19900	1.688079	0.861065	0.928237	1.426876	0.674958	0.473793	0.463485	0.428223	0.023912	0
Cs7g20000	4.164289	3.099825	9.265498	6.916922	5.562646	7.730992	2.786321	8.006847	3.830171	6.497872
Cs7g20060	2.547064	2.265135	3.885121	2.523458	3.078653	2.068887	1.062537	0.946616	0.077327	0.03622
Cs7g20270	0.143585	0.361395	0.645891	0.470278	1.056977	0.811667	1.20555	1.18241	1.723385	1.580365
Cs7g20300	36.38491	29.05441	39.40126	34.695	49.44432	44.38976	149.2645	76.3065	48.04758	59.3279
Cs7g20450	25.11944	33.9175	20.0634	25.11247	25.13303	23.90668	11.7404	28.07627	9.781582	9.937054

Cs7g20460	12.37321	16.67606	9.492473	8.824924	12.19241	9.709667	5.194945	11.07223	4.95244	2.705178
Cs7g20580	5.824615	6.286024	3.496837	3.374517	1.514816	1.938332	0.478814	2.0756	1.589803	0.828361
Cs7g20805	1.179243	1.137944	1.542856	1.747901	2.009296	2.967984	1.15351	3.17407	1.60667	1.482005
Cs7g20820	2.409606	1.9641	3.487085	3.598795	1.554554	2.547251	0.901583	1.602301	1.762632	0.882082
Cs7g20870	9.940538	8.452135	11.74517	13.29466	7.517618	12.52959	22.07888	20.44629	39.02276	20.30343
Cs7g21110	1.148431	1.277379	1.834981	1.327146	1.300035	1.351005	0.642318	2.144977	1.138336	0.865263
Cs7g21230	102.1743	140.0395	97.88325	116.9947	40.63012	56.34206	70.42613	113.9997	64.54525	147.7845
Cs7g21300	2.234941	2.804923	1.802966	2.24718	1.21177	1.549765	1.120571	2.964399	1.667727	1.758494
Cs7g21340	1.392944	1.242286	1.410534	1.958225	0.444351	1.352095	1.570766	5.994378	8.239889	36.58777
Cs7g21490	4.82148	4.90846	1.901433	3.479411	0.188601	0.458636	0.158279	0.879858	0.891991	4.08609
Cs7g21570	18.91314	12.82574	11.39834	12.74436	3.99344	12.47051	8.730983	8.383492	17.7087	19.22526
Cs7g21580	7.393132	4.201594	1.984329	2.290789	1.086738	2.223186	2.83392	1.532478	3.051753	1.999324
Cs7g21630	1.477949	1.274307	0.748804	1.210448	0.141939	0.447726	0.22814	1.146439	1.702885	10.13911
Cs7g21650	21.52285	22.46501	44.9903	42.1632	69.20281	69.47866	80.10199	120.2055	69.85606	89.67811
Cs7g21700	3.933318	5.352474	5.50807	5.018453	4.31415	4.645985	2.773282	7.557702	11.21631	14.01791
Cs7g21710	1.253527	0.608638	1.634581	1.254953	2.895461	3.69753	2.47954	2.060728	3.931881	3.430633
Cs7g21730	5.34254	5.641929	8.335578	7.989407	4.79355	5.025338	2.6709	6.07818	1.624843	3.217744
Cs7g21795	1.303335	1.31355	1.398667	1.412196	1.221733	1.804607	0.510208	1.540495	1.721814	2.074664
Cs7g21900	0.257495	0.263793	1.426509	0.979871	1.160843	1.01745	1.534482	1.582096	0.994608	1.857169
Cs7g21940	11.07194	11.17926	9.182682	11.75151	3.335183	10.29775	5.358417	7.702218	7.862477	3.764114
Cs7g21980	0.030576	0.010588	0.441393	0.622943	0.395886	0.695778	0.554645	0.524528	0.805923	0.528497
Cs7g22000	8.814279	6.118055	4.98389	3.326172	17.44747	18.00587	31.28177	9.570609	70.22206	14.12994
Cs7g22090	0.369583	0.76376	1.60603	0.855984	0.319552	0.261105	0.083015	0.138685	0	0.022162
Cs7g22120	0.139133	0.101672	0.561578	0.757494	0.691881	1.057901	0.155747	1.348442	0.101758	0.174977
Cs7g22250	6.660596	7.678859	15.18996	16.82852	32.27104	38.7932	29.68014	35.2554	88.82833	70.3212
Cs7g22270	1.094861	0.751897	0.571895	0.71684	0.58474	1.153579	2.553689	1.864807	14.0168	6.420784
Cs7g22350	0.464223	0.427923	0.836981	0.6379	0.270298	0.389964	0.06533	0.191783	0.298491	0.783151
Cs7g22390	36.61041	29.0328	13.47161	15.09541	8.483623	10.65849	5.892745	14.69702	10.59081	20.27162
Cs7g22420	78.0071	115.3423	92.70218	91.1191	20.52169	29.91822	15.03111	72.34081	5.780513	8.676951
Cs7g22430	12.9916	25.88661	25.29305	22.56847	9.645007	10.18688	7.81184	21.8999	4.855426	5.019321
Cs7g22450	14.2413	30.73956	28.9541	25.44466	10.82631	11.29102	8.482021	26.84936	4.354462	4.293951
Cs7g22460	27.34914	25.30738	23.9081	29.05916	13.7103	23.67729	5.171548	18.69682	17.53623	21.3303

Cs7g22470	1.612395	1.656396	1.735225	2.194392	1.213529	1.032471	0.384403	1.474642	0.52361	0.546542
Cs7g22555	51.90258	51.07195	41.46459	46.49406	24.89045	27.01542	8.29284	32.22277	10.07686	10.51168
Cs7g22560	3.136987	4.200248	3.655243	3.666458	3.409977	2.012326	2.660469	2.85947	1.093616	0.472653
Cs7g22600	146.7576	171.1099	81.73571	94.51218	33.3447	36.39172	23.77122	38.82816	3.961191	7.512822
Cs7g22610	40.88747	67.97488	14.863	21.17046	9.884457	9.832656	8.572124	25.76578	3.247833	5.100535
Cs7g22650	8.895995	10.66056	13.04766	14.63901	12.05583	16.09541	10.56421	22.66436	28.15692	69.61973
Cs7g22730	52.70504	50.16834	93.50979	104.1252	100.0652	181.5949	55.27659	38.85285	588.7538	516.2891
Cs7g22760	0.063498	0.249324	0.261594	0.305173	1.123323	1.651902	4.010832	2.100338	7.851039	3.644495
Cs7g22775	21.23515	25.1941	18.43411	25.29595	14.2961	17.56377	13.76174	31.02876	20.74652	21.9071
Cs7g22860	22.24974	35.11872	9.057808	21.72016	4.605213	4.095819	2.306835	6.114248	1.605809	2.380028
Cs7g22920	1.917973	1.163246	5.350705	7.130078	0.621137	1.550543	0.224268	1.016008	0.066902	0.088167
Cs7g22930	21.63791	25.24239	14.77416	21.20368	1.815582	2.238527	0.334964	2.052559	0.06375	0.052469
Cs7g22980	9.279383	9.538831	4.083184	6.19582	2.121484	2.456439	1.080865	3.086124	0.262337	0.236661
Cs7g22990	0.513045	0.945779	0.551321	1.000091	0.387882	1.002254	0.337701	1.709262	1.890895	3.717899
Cs7g23010	0.548495	0.601198	0.618444	0.675476	0.599077	0.813919	0.479233	1.07237	0.729971	0.309948
Cs7g23020	47.69976	46.67709	34.85259	37.95851	18.58025	17.27877	12.71582	30.39987	13.40223	28.47028
Cs7g23100	0.729581	0.330304	1.404984	1.024327	1.413031	1.814614	1.941727	1.03831	1.445688	1.390287
Cs7g23120	1.117067	0.933204	6.648012	5.952683	1.024019	2.463999	1.014546	3.179871	0.660132	2.763411
Cs7g23150	6.805457	6.664776	4.918011	5.146378	4.431997	3.644439	2.690208	3.500191	2.155313	2.506299
Cs7g23340	32.73481	31.80989	47.43208	35.02812	64.21935	46.76652	39.20555	110.8359	100.3534	152.1647
Cs7g23380	16.55521	18.10394	17.55814	19.50962	12.51847	15.96649	11.25565	23.12926	17.55111	25.28416
Cs7g23420	7.384736	9.095024	9.761532	10.27103	8.821704	11.13509	5.463889	13.00428	10.43152	14.84273
Cs7g23450	15.2934	9.071205	5.754015	5.680882	4.648552	6.582131	4.757778	3.897909	2.928014	1.907733
Cs7g23510	497.8974	594.2442	587.4839	576.6491	355.6907	389.9491	263.8675	287.535	391.3551	455.4185
Cs7g23520	0.049593	0.022804	0.024235	0	0.069401	0.100154	0.067121	0.318031	0.287138	1.144141
Cs7g23530	0.723767	0.711254	0.678463	0.685709	0.836451	0.768825	0.544083	1.186893	0.500925	0.783068
Cs7g23655	2.60776	3.648924	2.414311	3.118884	1.092855	1.639121	0.586816	2.843595	1.351727	2.209865
Cs7g23770	17.26505	25.25119	8.375259	15.40949	4.373778	5.893438	8.178431	11.09862	4.355535	6.554408
Cs7g23810	45.32998	33.4991	22.50013	20.88272	8.26964	11.04636	3.784132	3.69507	0.789351	0.297056
Cs7g23820	18.57108	28.40131	7.157537	11.37458	5.875146	10.0985	5.85417	49.21872	12.78459	6.431145
Cs7g24240	23.08808	17.63023	15.81551	16.1455	5.805631	8.490326	1.811786	3.455161	1.676539	0.897166
Cs7g24340	5.399532	2.731503	2.87577	1.042221	7.539897	3.612136	13.5514	17.19446	16.99531	19.49744

Cs7g24350	0.404582	0.299737	0.496632	0.249762	0.850666	0.699758	0.37592	1.162896	2.974272	5.245159
Cs7g24390	16.8735	27.13981	25.24065	29.0364	35.66462	45.98942	62.29414	50.28792	66.9259	78.32175
Cs7g24430	4.668666	5.340365	4.416427	4.673901	3.863556	5.337317	1.906539	5.138959	8.132996	5.447438
Cs7g24450	4.096245	4.566071	5.493191	5.321418	5.572641	8.472621	3.496875	7.998505	7.292335	6.509169
Cs7g24650	0.717096	0.588023	1.248257	1.592717	0.39495	2.106095	0.176772	1.04527	0.042801	0.055342
Cs7g24690	20.34789	24.48808	22.21869	20.88644	17.9668	15.89937	21.98043	23.36224	12.4439	28.30089
Cs7g24730	23.05992	26.85319	8.329766	10.7963	5.357795	6.748109	1.484345	6.106857	61.76717	74.82737
Cs7g24740	0.417635	1.204121	0.112623	0.138961	0.086033	0.025154	0.008025	0.116335	0.441539	0.396443
Cs7g24800	2.447108	2.220687	3.97343	3.716768	5.698759	6.538707	2.600004	7.388231	6.263927	3.901504
Cs7g24810	0.879942	0.371406	0.351924	1.385319	0.914298	1.261385	0.829195	1.323957	1.358123	1.495712
Cs7g24820	12.88297	7.263966	20.08029	20.38212	8.900784	17.0587	4.363678	9.200473	4.386119	1.771627
Cs7g24830	2.379586	1.082692	2.9912	2.777497	1.021542	2.041938	0.495824	0.718583	0.360473	0.149536
Cs7g24870	1.941847	1.544323	3.394561	1.850885	2.49334	3.635694	3.260241	6.208254	0.491656	0.61468
Cs7g24920	2.632921	1.317703	1.459851	0.749863	10.22612	1.867332	7.891374	2.475807	27.25493	8.931548
Cs7g24940	32.12479	31.82705	24.71702	28.42528	56.00831	69.08066	75.31221	34.11414	28.15052	5.619901
Cs7g24950	2.295293	2.943397	4.447001	5.413671	9.658331	10.32778	9.503698	16.60823	14.82708	11.41932
Cs7g25030	0.462624	0.488338	1.065057	1.633194	1.407308	3.022705	1.770687	1.066392	0.615508	1.075013
Cs7g25130	74.55687	57.88424	51.67164	47.9509	152.6017	136.2621	174.7157	66.96865	82.12426	75.03909
Cs7g25170	18.2232	20.77537	22.46338	18.85751	21.68062	17.88418	25.77748	47.06948	0.455593	2.897981
Cs7g25280	20.08206	25.35658	36.61431	34.46706	25.91103	27.96363	23.12856	51.8553	18.90693	53.10198
Cs7g25300	28.20259	21.18694	39.69661	33.18036	59.16481	56.80771	108.5771	35.82127	52.21328	48.87229
Cs7g25370	19.06745	19.19641	23.72252	21.25285	16.81684	21.43314	11.14482	24.88338	17.02507	21.10003
Cs7g25390	46.20949	30.69428	95.89423	79.7711	143.4691	128.1932	181.7037	108.3745	178.1675	153.0026
Cs7g25580	798.7993	699.3163	380.7918	417.6017	45.64231	83.33386	8.735735	30.80027	16.5343	27.97403
Cs7g25590	8.587992	8.851186	6.361575	5.830689	3.851389	3.745089	1.501713	3.306718	0.556006	0.501297
Cs7g25610	411.6141	255.8594	190.9026	243.9894	386.8755	299.4109	548.5151	323.9898	170.2159	50.19627
Cs7g25680	49.88879	73.21453	44.8229	53.02119	14.9563	22.88988	42.14529	72.63119	89.22735	229.5895
Cs7g25720	2.29286	8.368602	0.70905	2.049787	2.418736	2.168112	13.07081	11.56484	0.397813	0.370005
Cs7g25760	2.092601	1.798905	2.523454	2.675273	3.413108	1.759603	5.600016	2.340065	4.222283	3.468358
Cs7g25790	1.077295	1.712095	1.112981	0.895688	0.198228	0.582068	0.111791	0.997324	0.020562	0.017381
Cs7g25800	26.96286	24.18511	10.50543	11.06536	6.016861	9.246583	8.050172	10.61687	8.371495	2.272146
Cs7g25850	4.898403	5.431947	7.685737	7.811031	10.31271	10.97025	9.952387	12.98311	8.859097	15.94305

Cs7g25890	33.96107	22.64074	15.92565	16.97866	7.081331	9.041642	25.06923	15.96749	73.77401	72.46926
Cs7g26040	14.28593	11.73197	23.98721	19.68336	16.06372	23.19785	9.448556	21.25844	9.00568	15.58593
Cs7g26085	2.830139	2.80808	5.218297	4.330688	4.495091	8.161724	3.480465	7.64214	2.73458	3.973863
Cs7g26110	3.255371	3.171133	6.489569	4.358696	5.104649	6.282869	2.064046	8.811929	1.518877	2.052608
Cs7g26180	19.21481	15.06364	27.09821	22.18272	52.0956	50.2898	76.01869	41.25549	42.03898	35.53421
Cs7g26250	5.450355	5.49118	1.019674	1.648101	0.540474	1.450037	0.963294	1.639892	0.012765	0.113904
Cs7g26290	0.436748	0.865754	0.415114	0.675805	0.571022	0.690923	2.626877	2.273708	0.730063	2.053249
Cs7g26330	0.914712	1.143438	1.683122	1.836804	1.632387	2.94318	0.927566	2.845672	1.849698	3.112404
Cs7g26370	71.77071	39.06754	69.94714	34.37185	70.7498	39.15168	35.7812	20.4115	289.4018	63.91192
Cs7g26450	1.003249	0.684442	1.908594	1.298025	2.799709	2.147203	4.519857	1.592918	1.638128	2.083903
Cs7g26560	251.8821	181.904	165.3407	175.7221	399.2713	378.5482	481.9346	269.2575	467.8427	374.6354
Cs7g26620	88.33422	60.05725	92.37419	62.60256	164.9209	122.6413	100.6093	57.49052	159.1591	52.75383
Cs7g26640	0.343499	0.665915	1.454631	1.211398	4.446626	4.579613	1.508866	1.991767	0.130806	0.129074
Cs7g26690	14.49566	31.85814	5.797818	5.89483	1.232354	1.645783	0.516461	0.414376	0.188407	0.044843
Cs7g26710	48.76262	24.24203	12.01987	18.00637	16.78883	16.81866	46.44694	37.76444	128.4721	110.2894
Cs7g26730	19.56317	18.60552	11.90865	10.56291	4.443509	6.299204	4.005652	9.934961	7.14999	11.50797
Cs7g26750	22.41485	25.77009	10.54015	12.90743	4.5632	7.932642	4.357575	12.74052	11.02374	13.28026
Cs7g26860	1.371744	1.221349	2.299759	2.311147	1.168406	1.841249	1.236048	4.420066	3.443849	5.387575
Cs7g26960	0.48974	0.736616	0.764547	0.716024	1.031476	1.433358	0.925778	3.431771	2.48097	3.867326
Cs7g26970	0.368289	1.852217	0.170424	0.229754	0.085619	0.045703	0	0.104801	0	0.161215
Cs7g27010	1.528497	1.933111	7.052569	6.430409	32.40179	23.8652	38.37578	36.29331	31.54031	31.17778
Cs7g27030	0	0	0	0	0	0	0	0	2.104941	0.542655
Cs7g27040	2.00284	4.949743	0.108089	0.449279	0.008711	0.016161	0.009686	0	0.079363	0.028265
Cs7g27120	0.994326	1.792906	4.404094	3.311206	0.659723	0.842538	0.347173	1.200462	0.058868	0.144079
Cs7g27180	32.83727	31.53636	48.84997	45.91588	78.56214	102.0405	145.9523	66.53853	66.79792	68.73926
Cs7g27290	20.16117	59.32517	5.823081	6.575447	11.35165	16.23307	1.789016	9.106331	1.779526	1.834333
Cs7g27360	6.098632	4.977202	14.25177	11.4084	11.47322	29.3425	13.61794	27.20791	3.57286	4.90066
Cs7g27380	5.609407	5.26736	3.205939	3.488559	7.671532	4.536243	3.469702	9.103181	12.03844	8.731106
Cs7g27400	66.36604	67.64562	96.38741	96.46066	124.7134	126.863	193.9139	143.6504	224.9742	349.812
Cs7g27450	2.196705	2.766926	3.947439	2.865754	1.445295	2.315466	0.753399	4.425146	1.165102	2.084601
Cs7g27460	12.37526	8.923444	25.76067	19.83279	37.21479	36.3746	50.06288	27.26528	37.5514	29.49327
Cs7g27470	188.6345	191.3216	216.4825	186.0113	200.9709	156.7904	374.0936	174.282	128.9112	145.3812

Cs7g27480	3.781893	3.446941	6.339028	7.855881	3.644385	5.456277	2.092277	3.440023	0.528549	0.834092
Cs7g27510	1.821255	1.659762	1.005861	1.395101	0.794062	0.602325	0.908728	1.162463	0.889767	2.449275
Cs7g27540	61.15775	54.59523	40.06957	45.81799	14.81093	20.44868	4.98221	16.53913	15.14712	8.176366
Cs7g27560	3.296745	3.564881	3.575353	2.996139	3.9935	3.64681	1.402785	4.492451	3.485124	2.298774
Cs7g27640	1.440557	1.748912	3.435753	4.121885	2.667531	5.447764	6.782854	6.179163	20.19951	32.69235
Cs7g27685	124.1325	80.31279	48.86567	33.97351	116.9437	20.01321	39.42792	140.4445	27.59753	40.36597
Cs7g27700	0.854828	1.049786	1.116653	0.78432	0.999477	1.186884	0.56979	1.297347	0.772358	0.682834
Cs7g27850	4.620873	6.148259	4.625334	6.436999	5.564179	7.945536	2.53449	6.770658	7.33718	10.0637
Cs7g27875	2.066409	2.793455	2.692188	3.226111	2.81824	5.365723	1.693433	4.029178	5.193984	6.616997
Cs7g27910	0.568511	0.735959	0.46436	0.381556	0.824439	0.780534	1.716466	1.010998	3.056301	2.594197
Cs7g27960	11.13627	13.5382	10.63585	11.01924	4.873695	6.094523	2.196889	6.798582	3.098247	5.609371
Cs7g27980	2.700384	3.423397	2.27688	3.080696	1.3981	2.065412	0.840394	2.39458	0.537206	2.14534
Cs7g28055	9.923549	7.790262	17.51087	15.72335	31.2197	40.88499	29.60103	71.68357	64.25731	47.70841
Cs7g28090	12.00235	8.45644	13.63121	12.1582	16.72339	23.05711	33.28898	16.65672	19.22278	7.385087
Cs7g28110	20.77637	22.42471	39.7667	55.15375	3.652731	8.832107	18.33569	59.03218	14.48894	87.28546
Cs7g28340	181.3053	222.9819	212.0493	288.1888	61.24304	68.42985	18.2612	21.94845	4.780003	4.699345
Cs7g28370	0.665655	0.58918	0.69828	1.01932	6.038462	20.13202	11.80278	104.4302	55.36791	43.84482
Cs7g28460	1.376072	1.214474	2.524836	2.09082	4.146458	4.190235	5.308068	3.996482	3.812222	2.670896
Cs7g28520	4.001223	2.471797	7.197102	5.009946	2.6844	1.788917	2.049493	2.22408	0.450904	0.572243
Cs7g28620	8.92894	9.750874	3.427819	4.416284	2.964054	3.199828	2.541535	6.337969	3.270751	7.899538
Cs7g28650	158.0562	64.88858	42.72704	62.92495	103.5368	105.6643	79.72284	57.25645	72.81205	36.7116
Cs7g28690	0.537161	0.675644	1.945211	1.292657	2.206766	1.363391	1.472298	1.785254	13.41497	6.40567
Cs7g28700	3.437339	4.732458	3.859413	3.201004	6.084466	2.87663	10.92801	4.802485	64.69459	39.75136
Cs7g28860	7.495627	6.259854	6.833768	5.936572	5.320424	7.276962	4.206541	10.20439	12.37705	5.916935
Cs7g28915	8.496203	7.587463	10.64001	8.648195	21.24059	18.94268	5.579492	28.26699	13.72629	9.379579
Cs7g28940	17.2475	10.43348	20.12472	16.84646	39.02693	48.71577	50.02552	22.23625	169.4732	103.8579
Cs7g28970	7.869916	8.30261	6.996851	5.76058	5.243772	4.675977	1.467672	3.239255	0.062009	0.038407
Cs7g29010	266.8352	158.8453	276.1506	270.1771	518.3339	803.9795	733.0964	351.6514	2610.088	1772.549
Cs7g29020	10.10643	7.867939	8.011597	9.019486	1.480816	3.042689	7.577615	8.169682	3.372574	4.227043
Cs7g29030	0.853966	1.541913	4.154898	3.701908	0.19731	0.899279	0.41291	0.939089	0.141376	0.217639
Cs7g29040	0.830423	0.656828	2.233396	2.451371	0.616088	0.732649	0.942922	1.105132	0.038298	0.034501
Cs7g29200	3.448163	2.477537	7.535622	6.300538	9.173529	6.886439	12.09396	12.95215	7.939948	10.18609

Cs7g29230	0.066283	0.064749	0.27737	0.126874	0.754375	0.608146	0.782688	1.348845	0.812268	0.914903
Cs7g29300	23.87292	25.62006	16.04633	16.99504	7.491554	9.038492	4.385476	6.041781	1.642546	1.238789
Cs7g29330	2.778893	2.860739	2.584713	2.694548	1.380194	1.785433	1.119742	3.107508	1.329902	1.628778
Cs7g29470	2.573057	6.833025	27.90664	23.84504	34.58705	40.82914	23.12869	25.30535	5.547025	7.218538
Cs7g29530	9.605705	17.83141	24.73724	27.75091	28.74553	30.36369	18.07688	31.19728	22.77743	27.40942
Cs7g29570	9.532759	16.98021	58.55816	60.91538	29.20865	32.37299	9.279311	18.73059	0.498492	1.196105
Cs7g29685	3.264457	3.524336	3.418021	3.153487	4.569776	5.049106	2.199961	5.963451	3.370366	2.166751
Cs7g29690	14.80427	11.22805	23.85054	21.17842	33.08058	35.58691	12.3684	15.6519	18.04438	16.49633
Cs7g29730	7.034966	11.36284	16.55629	17.38241	2.157253	13.77821	63.62045	33.24295	53.15149	70.70578
Cs7g29740	0.819054	1.325612	4.454112	3.898892	0.745343	2.732142	2.622916	2.90687	0.317957	1.348582
Cs7g29750	0.80991	1.370143	3.669226	3.759894	0.810914	2.81191	2.775048	2.850108	0.362951	1.711563
Cs7g29760	1.831559	2.828839	10.65287	9.717432	1.782347	7.196666	6.581979	6.912574	0.598689	3.631522
Cs7g29770	1.561878	2.459692	8.317318	7.60129	1.223196	5.159923	5.015126	5.803787	0.498841	2.672631
Cs7g29830	64.73328	41.54114	290.878	172.8235	466.9181	567.877	855.092	390.8509	478.6013	230.9992
Cs7g29860	61.29603	85.0381	18.59854	27.02285	18.66409	19.07153	33.2179	41.49713	13.26557	15.90816
Cs7g29880	3.710975	6.93831	1.243182	1.773348	2.020832	1.771159	3.477149	4.134508	1.395819	0.717449
Cs7g29900	8.388914	9.515339	13.13436	11.31717	13.92329	8.948603	22.00931	24.62008	10.79309	7.117997
Cs7g29910	5.986206	10.71987	2.224982	2.934662	4.093636	3.71334	6.262786	10.48386	2.859933	1.984884
Cs7g29920	6.353035	10.8927	2.399679	3.88518	3.832303	3.221655	8.188704	11.82619	2.203631	2.569793
Cs7g29960	11.26531	13.21171	9.155693	11.05322	5.680149	9.418418	2.352373	7.447535	7.166844	11.91277
Cs7g29970	9.003261	9.368083	10.34238	10.49922	6.448708	8.137968	4.561811	8.095934	1.943052	4.700725
Cs7g29980	7.513728	15.51869	18.81001	24.92492	3.188896	7.90246	8.183398	42.966	4.824923	24.54137
Cs7g30000	2.625047	2.525813	1.129377	1.393729	1.663856	1.843032	6.448457	5.921331	2.257847	1.345609
Cs7g30010	9.357711	25.12907	2.24744	8.463737	3.503009	2.762832	4.592041	10.11471	1.282431	1.02587
Cs7g30020	3.002546	2.976231	5.22381	4.30794	1.121267	1.417441	1.272036	0.830842	0.14576	0.050889
Cs7g30060	7.911248	4.447721	14.32446	9.086911	22.18321	24.18938	26.74092	21.52444	9.121816	2.7442
Cs7g30090	23.33746	27.68832	41.92886	34.33715	52.25467	31.05817	64.87923	61.90392	46.98589	45.57287
Cs7g30170	3.980809	11.27107	7.429947	20.5964	8.285884	26.51946	12.91141	41.50683	0.766287	1.342524
Cs7g30280	671.3339	576.1198	245.0309	350.3924	43.86693	117.4364	103.0967	159.6588	125.7308	265.2786
Cs7g30290	9.078017	9.816437	8.191278	10.77577	4.976699	7.67739	2.217173	7.389917	5.430149	5.874742
Cs7g30320	3.160917	2.637539	3.216523	3.65643	5.719411	4.448205	7.953638	7.24153	0.61845	0.323572
Cs7g30350	2.876702	6.736869	4.038074	5.207047	3.549581	3.468635	3.152143	10.58057	1.528755	4.56549

Cs7g30485	41.81868	14.06411	29.20289	21.35314	15.76241	13.89763	20.32109	14.2074	22.06918	16.28893
Cs7g30500	1.285889	1.389276	2.747415	3.398372	5.594638	15.14237	9.062607	8.139855	7.125754	5.597737
Cs7g30530	1.05166	1.519523	2.060034	2.55855	1.921841	2.069518	1.293652	0.269363	0.869638	0.425743
Cs7g30580	37.72389	30.2758	88.81382	58.44741	91.89031	92.42309	21.8605	20.84453	11.59154	10.40786
Cs7g30610	20.25566	16.43063	43.49483	32.70704	33.97371	44.55369	9.176194	10.90759	5.28569	5.048119
Cs7g30650	12.14448	14.30575	5.88543	9.267378	2.039859	1.951896	1.83327	3.479196	0.213648	0.547514
Cs7g30670	12.96282	9.796595	8.239273	7.081358	8.226102	12.29406	4.843845	6.175679	11.11789	5.260399
Cs7g30680	3.77148	2.897228	3.17817	3.027705	1.539208	2.306577	1.390337	1.208847	0.370405	0.337114
Cs7g30800	0.601535	0.604122	1.697355	1.075419	2.6354	3.194873	2.446018	2.49334	1.227921	1.224344
Cs7g30820	6.751664	3.198237	2.653984	2.527064	4.575584	6.808944	15.25076	7.439482	9.272594	2.193576
Cs7g30910	4.641227	4.185427	1.206997	2.734452	0.270004	0.262003	0.478697	0.289141	0.224474	0.501698
Cs7g30960	0.793155	1.449874	0.365684	0.709033	0.08838	0.101168	0.171188	0.325184	0.018442	0.02045
Cs7g30970	3.046454	3.329637	1.312893	1.554277	0.766119	0.787116	2.652791	1.618148	2.414976	2.743939
Cs7g31000	109.7018	264.1813	228.5764	322.8186	23.66979	36.04146	88.17558	201.4494	22.89807	113.6488
Cs7g31010	11.63757	12.32548	20.62575	34.47497	2.931355	15.79086	27.91111	28.95595	218.3684	217.7076
Cs7g31060	4.699013	5.292152	7.750379	7.399087	2.31442	3.012246	8.032709	11.35095	8.622173	15.58864
Cs7g31090	10.20739	9.274692	5.422489	5.371516	3.627703	4.586551	4.596162	9.210059	7.01808	8.880131
Cs7g31130	8.853293	8.556238	6.431208	7.955921	5.554616	9.256716	6.556383	15.92802	3.312007	5.450084
Cs7g31170	224.1869	216.3733	123.4053	99.55231	142.7824	101.1896	71.46832	107.6319	25.83846	28.19227
Cs7g31190	113.7157	140.6788	72.55654	89.95217	22.0556	20.747	7.502796	41.80004	2.614754	6.487305
Cs7g31200	8.471062	9.596193	11.54318	14.3473	11.12089	16.37483	6.556436	20.29311	11.2166	21.70848
Cs7g31240	1.312184	0.944297	2.544327	2.836707	1.029852	3.408981	3.452269	6.599813	23.53884	31.48742
Cs7g31270	8.961365	8.179104	27.42502	18.74075	42.81592	35.16372	42.24325	51.21417	8.911833	21.77228
Cs7g31320	6.262061	6.379185	9.825876	9.298401	4.656478	6.679202	9.687753	14.32278	3.508749	9.452464
Cs7g31340	134.7033	146.843	247.0139	238.9892	331.3427	275.9386	503.6999	211.837	229.4192	253.9895
Cs7g31360	29.10228	46.95632	21.60813	27.4767	3.855544	7.856995	15.35778	40.73005	20.38136	74.37397
Cs7g31410	67.26037	54.43824	10.71502	23.85138	97.30384	46.78056	116.3737	33.44133	32.61088	2.387959
Cs7g31430	8.948191	10.03069	46.75581	33.50412	29.91143	45.34697	10.52449	11.12929	5.276273	11.36888
Cs7g31530	14.01671	11.58739	23.22731	19.96172	28.91945	28.16127	35.37552	32.43314	40.16598	43.51833
Cs7g31610	0.005402	0.161297	0.358166	0.340305	0.847421	0.328627	4.193168	5.746423	3.797389	5.092396
Cs7g31640	66.55984	94.78063	25.73012	60.98774	4.388047	4.137019	1.359265	2.13849	0.781387	0.310514
Cs7g31760	1.476536	1.94552	0.820075	1.19148	0.352311	0.958432	0.488214	1.267201	0.871622	1.74359

Cs7g31790	4.311704	3.959059	5.532754	7.514982	3.213956	2.771307	9.621542	10.25373	24.92691	14.01195
Cs7g31800	371.1512	762.3856	49.54554	238.875	23.5093	27.23288	21.27552	30.6557	24.60327	24.56555
Cs7g31930	0.535734	7.332508	0.199154	0.599353	3.968008	3.218096	7.414652	20.12379	10.01468	18.43871
Cs7g31960	90.8439	85.49522	61.67153	72.1257	27.45883	71.50389	32.82503	61.33706	40.9078	47.20221
Cs7g32070	2.968485	1.725752	3.32765	3.644249	2.296407	7.129691	3.170689	5.447384	2.210282	7.035208
Cs7g32150	0.367026	0.794908	0.380797	0.441008	2.094819	1.64402	5.388484	7.66953	46.11933	69.02467
Cs7g32160	0.850428	0.763013	0.991319	1.047604	0.614882	1.21321	0.460984	0.507271	0.416834	0.32779
Cs7g32200	9.070795	10.32327	13.55623	10.87255	4.123563	4.892704	2.850795	3.181428	0.194013	0.333386
Cs7g32230	66.40146	61.56047	38.37507	34.38292	25.12513	20.06915	26.33897	21.95353	15.20748	12.75412
Cs7g32240	56.71264	54.99668	54.77417	46.62528	71.71298	39.98256	102.7429	59.25235	29.11851	19.57757
Cs7g32260	13.04533	10.07429	9.107228	5.210879	12.50534	4.84938	13.31422	9.49225	1.830249	1.403991
Cs7g32270	26.78461	11.23006	8.281138	7.921152	4.977617	8.65411	2.260713	2.680908	0.583368	0.119525
Cs7g32280	5.835684	3.214373	7.863947	5.602214	14.637	13.15939	12.30966	5.383464	3.870619	2.376351
Cs7g32340	17.04842	12.85685	13.09877	13.62541	7.824069	14.51879	11.30094	10.60202	46.85423	45.96832
Cs7g32410	3.173409	4.755443	1.90125	1.811976	0.731378	1.654296	10.70583	3.475625	0.147039	0.208538
Cs7g32420	9.79824	10.0442	10.86974	9.564733	3.796007	5.861883	1.702784	5.325751	0.807925	2.143095
Cs7g32460	9.038451	10.8086	18.43974	19.06732	18.57792	26.28638	8.297218	27.22908	8.67245	13.0368
Cs7g32490	6.43587	6.72914	9.188291	10.32611	9.361147	13.10762	7.067766	13.0806	7.940573	19.15148
Cs7g32520	18.20984	24.61833	15.74474	18.97615	8.701841	13.93656	16.38518	24.18964	33.39355	132.6766
Cs8g01040	29.46358	52.99724	11.85893	24.41448	8.184206	6.394472	6.87277	11.18096	3.080572	4.162994
Cs8g01050	24.25512	25.10561	37.7947	35.75124	42.74434	58.23118	24.12262	63.42669	59.60551	49.45233
Cs8g01090	0.233902	0.30725	1.065526	1.121217	0.187327	1.052193	1.165262	2.038513	0.171195	0.604049
Cs8g01170	18.79303	18.83947	1.632862	4.363364	0.167912	0.451468	0.057717	0.594449	0.067843	0.178865
Cs8g01220	22.93098	22.97221	41.42839	34.01244	52.1781	30.13713	46.44288	46.95527	36.88598	20.69023
Cs8g01230	0.773325	0.945833	0.617317	0.670645	3.527397	0.866446	3.428165	1.539606	0.902797	0.976378
Cs8g01260	90.72983	61.91701	34.99727	38.55278	9.934796	16.92074	6.701385	24.43474	16.31236	16.82023
Cs8g01410	23.09431	15.84302	7.049993	6.355816	7.684019	13.02248	15.46226	27.48776	6.505888	12.89812
Cs8g01470	18.19049	19.38431	22.90343	25.06709	13.87475	22.12144	14.83158	26.18377	13.70043	33.26229
Cs8g01480	13.71321	13.51247	12.28156	12.75394	9.252987	11.56874	7.293488	16.92257	7.537241	9.705853
Cs8g01540	9.327819	9.550249	6.956035	8.201462	3.755813	6.19017	2.79345	7.0884	3.370692	5.276194
Cs8g01550	5.707071	2.484484	0.882274	0.877516	0.141988	0.326297	0.124949	0.324104	0.033127	0.099925
Cs8g01570	13.14581	15.85858	23.40004	19.82396	24.41784	24.07255	12.56777	29.83185	14.59181	13.29948

Cs8g01630	6.429143	4.879777	17.16769	10.64913	22.40916	14.66108	7.514037	19.84104	2.185515	3.612857
Cs8g01670	11.7439	9.915474	8.375609	11.59776	6.86243	26.96304	5.756826	23.63842	34.94902	48.19308
Cs8g01690	2.739042	2.66205	3.026459	2.797035	3.293764	4.624761	1.850351	5.457163	1.703405	2.103019
Cs8g01700	25.16442	32.05452	11.11944	17.82209	7.592805	7.787356	3.347349	7.397225	2.628009	4.781283
Cs8g01800	8.598672	10.53456	9.320874	8.915597	6.795342	8.466406	3.515208	9.7813	3.675587	3.53554
Cs8g01810	401.18	325.157	34.34731	104.2422	30.73407	57.96376	2.481743	13.6844	0.288145	1.028344
Cs8g01840	1132.109	914.5523	61.91163	176.8938	56.79725	108.7044	5.37759	26.20781	0.549686	1.170847
Cs8g01850	2149.588	1880.942	231.7329	686.0151	235.1535	375.1221	13.33836	85.32536	1.041424	1.383752
Cs8g01860	36.13305	29.78865	57.2462	66.9756	45.91009	118.3235	7.178554	24.48777	1.453903	2.031503
Cs8g01880	133.2917	140.481	61.7427	84.73502	6.409683	32.35144	26.51436	174.151	23.39784	156.0114
Cs8g01940	6.830666	6.783673	8.365058	6.931464	17.91206	23.50378	24.92323	36.96791	1.195766	1.275364
Cs8g01950	1.720603	2.332427	2.432992	2.128067	5.279133	7.909434	4.945957	10.84893	0.389408	0.653279
Cs8g01960	19.17044	20.85492	5.61291	9.116763	0.309706	3.45645	7.372296	22.36295	16.50607	89.94491
Cs8g01970	16.86711	41.28205	3.07993	7.557076	2.035643	1.404643	1.865834	6.73751	2.450433	7.148984
Cs8g01980	1.352037	1.604209	0.928634	2.193418	0.272158	0.839078	0.505054	2.018386	0.020275	0.668464
Cs8g02170	10.65979	9.391274	9.069737	14.03074	3.534989	7.681592	5.383187	6.735325	43.1838	108.9015
Cs8g02230	0	0.038054	0.035664	0	0.020082	0.050729	0.957953	0.990594	3.143644	13.2185
Cs8g02260	4.203714	4.172572	6.389974	6.332588	11.40469	11.87059	10.87379	9.564133	19.07841	12.51544
Cs8g02370	4.430796	4.702886	6.528062	5.853037	1.589661	1.935103	1.303175	3.611761	6.716665	5.153805
Cs8g02380	4.296023	3.848075	7.424545	6.223902	1.80461	1.98001	0.669385	1.714549	3.438245	2.384826
Cs8g02440	0.236983	0.257994	1.53751	0.776808	2.515066	2.274188	3.559873	4.288188	6.316025	10.86729
Cs8g02450	29.12532	30.94025	51.81942	61.00019	51.82708	56.87708	49.37734	126.6191	56.8708	126.3664
Cs8g02530	81.85208	113.6682	20.13236	45.09968	1.714638	4.584963	0.701942	2.141375	0.795187	0.669033
Cs8g02560	169.7286	245.3894	58.5379	132.7815	9.241536	6.933337	4.245714	13.73	2.232389	3.555303
Cs8g02580	0.863812	0.991739	0.939669	0.867833	1.275188	1.129999	0.27651	1.423113	0.68498	0.777806
Cs8g02590	1.565662	2.430336	4.584243	3.591758	2.865376	2.460611	1.048225	1.282657	0.396875	0.382273
Cs8g02620	93.54324	49.92368	104.6692	84.34513	224.2278	184.1746	111.1351	86.97235	120.5749	45.80572
Cs8g02650	1.060963	0.668378	0.68097	1.097956	0.182969	0.822384	0.094335	0.058646	0.488203	0.354211
Cs8g02660	1.413439	2.375434	3.622287	2.980008	2.451021	2.395348	1.711758	4.27708	2.114238	3.160192
Cs8g02760	4.460282	3.614727	15.60197	8.288903	23.89585	18.26143	4.892271	3.532956	1.460325	2.144684
Cs8g02780	67.53869	92.10682	47.77855	54.14592	34.46528	33.21231	26.20249	28.65174	3.872906	4.778433
Cs8g02840	6.853032	4.574822	3.59218	1.870625	9.798985	21.47384	16.33985	9.477287	169.5664	46.25351

Cs8g02850	0.983661	0.470598	0.077707	0.151871	0.034115	0.128667	0.023614	0.160802	0.079993	0.19466
Cs8g02880	8.223317	8.892706	4.55581	5.395061	2.685027	3.136908	3.142921	3.417695	3.069525	2.301629
Cs8g02890	2.02669	2.524949	1.954511	2.303067	1.677952	1.568336	0.527743	1.169684	0.044411	0.049023
Cs8g03040	73.42898	48.50587	96.25681	67.85045	158.8532	118.7786	123.3762	55.89276	57.28177	32.77769
Cs8g03045	20.9529	23.48829	15.8194	17.42111	10.11296	12.13338	5.691229	15.49687	2.218583	4.278568
Cs8g03110	15.71233	19.60507	5.669263	11.15862	3.3352	2.644064	0.930243	2.97131	1.599337	3.310068
Cs8g03150	2.321318	2.332499	2.1699	2.09528	2.073555	2.07551	0.839066	1.932563	0.386513	0.719029
Cs8g03230	22.75336	33.1616	13.70631	21.57714	1.98271	2.560964	1.400664	4.804091	0.515984	1.259823
Cs8g03270	2.552055	2.515754	3.422	2.477863	1.694193	3.086565	9.173424	1.402749	0.374364	2.860796
Cs8g03370	52.62746	51.17126	46.6155	57.33294	12.12295	16.87684	6.879554	17.08663	6.1286	8.820163
Cs8g03410	20.96405	17.03008	59.52992	69.24305	11.85243	25.65491	9.701401	33.74093	23.96457	70.21869
Cs8g03430	8.296406	20.16635	41.529	48.0947	22.52628	18.18828	8.630033	27.28102	1.129527	3.995404
Cs8g03480	10.61502	24.8712	43.22985	45.83536	25.64422	19.2469	10.96191	29.35386	1.159998	5.102098
Cs8g03560	1.216746	2.895604	0.765614	1.322223	0.444933	1.066999	4.898719	31.18894	5.501401	64.03203
Cs8g03680	59.02892	50.06276	25.74849	33.24491	11.535	33.16788	8.472655	13.63591	28.21808	17.89967
Cs8g03700	2.06382	1.762881	7.346355	6.803075	8.027539	14.1053	5.653292	8.195184	12.17519	19.80393
Cs8g03710	5.892471	13.39329	3.845462	3.582448	2.548044	2.302426	3.266805	10.86871	2.824077	2.724128
Cs8g03770	13.5397	16.57221	33.08407	34.70549	23.48908	44.91498	5.54007	15.62259	12.98911	10.77125
Cs8g03780	1.174003	2.585168	8.115737	7.578813	1.448603	2.77044	0.969226	4.21747	0.154788	0.205829
Cs8g03860	6.232049	5.981733	1.294783	1.255461	0.724848	0.493513	1.103428	0.98983	0.153897	0.527333
Cs8g03940	57.51661	47.73623	16.51	11.87395	4.098593	6.51964	0.494183	5.76707	0.025181	0.034198
Cs8g03960	7.052164	6.253537	3.067935	2.368049	2.021753	2.102909	3.420273	3.541205	0.076188	0.081135
Cs8g03980	41.14336	21.9503	11.60983	10.59955	11.6577	4.926552	6.367176	1.396462	2.01925	1.244188
Cs8g04000	14.56264	7.294071	4.464279	3.744497	4.964884	2.330356	2.309158	0.492442	0.922815	0.530882
Cs8g04130	7.916013	8.691583	4.997755	5.133377	5.076466	4.683645	2.223957	5.996179	1.369454	2.046106
Cs8g04150	6.651497	7.715993	12.70524	13.26753	10.04856	11.94074	13.32913	33.99126	21.97485	51.96643
Cs8g04280	68.76699	82.91784	69.79511	76.22487	31.95222	31.54412	62.97597	81.61157	61.69088	65.82118
Cs8g04530	8.258889	7.450202	8.118557	7.377782	2.27689	4.987808	2.351642	5.964384	2.886623	1.22638
Cs8g04550	47.70858	20.75585	35.46062	28.7378	14.84418	43.88231	3.621461	51.58116	0.280289	0.461732
Cs8g04610	2.903889	2.108843	10.07372	14.82547	0.731427	3.691633	5.256741	5.355805	20.31946	13.30683
Cs8g04660	9.702365	20.22978	2.558825	4.075353	0.980509	0.638129	0.903109	1.275579	0.332884	0.39526
Cs8g04700	15.42727	54.61276	0.406251	4.693009	1.421974	0.488369	5.972351	2.786088	0.449566	0.143665

Cs8g04720	5.151018	3.983848	1.124221	1.379595	0.679698	0.761344	0.556517	2.118482	0.407988	0.429662
Cs8g04770	1.591164	1.976559	2.477925	2.077641	1.439227	1.638776	0.645807	1.746006	0.092426	0.195782
Cs8g04830	4.208705	3.509727	5.803819	5.244825	6.83342	6.865558	3.795488	15.8574	5.882428	15.45286
Cs8g04840	1.844098	0.945104	2.898674	1.973702	3.701355	1.65575	1.23754	2.318933	0	0.395482
Cs8g04880	0.293331	1.075962	0.454564	1.402156	0.448741	1.643334	0.199794	1.038377	0.432625	0.374163
Cs8g04920	107.716	106.1801	137.3745	129.6593	193.3206	114.4283	539.424	360.3734	275.0719	188.3421
Cs8g04930	282.8528	268.8367	396.1832	332.2959	425.1486	342.8911	1406.428	1258.697	1661.897	1322.158
Cs8g05000	8.00268	14.07695	11.13309	13.27747	2.599889	2.609068	7.463797	22.35649	8.282751	16.44313
Cs8g05060	14.06345	9.980394	25.29708	23.11038	61.98577	86.62127	143.7608	30.80473	122.8648	71.66987
Cs8g05230	48.39805	40.36677	9.489948	12.16898	2.084858	5.958288	0.843651	3.92139	1.743289	1.06993
Cs8g05250	14.9211	11.46062	8.747338	9.686156	2.986046	4.409611	3.175657	0.957484	2.168532	0.946359
Cs8g05270	0.143748	0.180677	0.075328	0.069379	0.15707	0.25679	0.501739	0.386619	0.918832	0.338805
Cs8g05280	7.443205	10.21854	19.4307	17.38303	19.68336	18.48973	41.67622	34.00369	8.775438	5.225317
Cs8g05290	24.76608	52.51006	48.86795	72.60185	8.43506	10.05246	15.44861	14.3758	23.10966	28.18954
Cs8g05300	12.80407	23.50874	30.37587	37.45204	13.01525	10.49896	16.83697	16.08071	10.85848	8.790838
Cs8g05320	8.389816	8.212685	9.931993	13.22188	3.22696	3.763773	12.18514	7.508565	27.42197	22.78079
Cs8g05370	8.744352	11.68566	4.111768	6.207294	1.797244	2.674754	0.718843	3.352526	0.785737	1.043649
Cs8g05410	56.25885	52.04576	93.72263	98.57517	211.1401	236.1275	230.7924	133.4281	47.24793	73.00844
Cs8g05460	29.74547	36.00122	26.19169	21.80006	11.43079	12.36926	4.325518	11.28095	0.208935	0.313591
Cs8g05490	5.901287	4.899133	5.694399	6.100078	3.217137	4.746201	2.145735	4.224179	0.842868	2.80914
Cs8g05610	6.906186	7.293511	2.100859	2.289714	0.600309	1.56235	0.446562	3.954153	0.056475	0.139089
Cs8g05670	24.26162	34.45829	22.89525	22.07688	19.92243	21.48984	9.224079	16.73101	1.705917	1.557531
Cs8g05740	2.883795	1.688102	1.618347	1.816759	2.230339	6.148294	1.579057	5.630519	1.402125	0.735043
Cs8g05860	12.05946	8.930582	19.48273	14.21942	28.34103	20.43043	35.99174	13.53647	14.05038	10.47175
Cs8g05910	10.80584	28.03563	14.9034	16.38286	0.987182	1.322582	12.946	34.43757	8.090692	130.6321
Cs8g05940	106.5669	115.1524	66.90043	64.01209	14.09132	15.80761	7.3744	6.192124	0.769987	0.307354
Cs8g05990	1.919387	0.660634	2.526957	1.66523	8.037163	6.125696	3.061062	1.050901	4.097566	2.647421
Cs8g06035	0.926742	0.978502	0.729566	0.862453	1.454555	2.236202	0.868704	2.722998	0.620912	0.442015
Cs8g06090	10.98128	8.825174	7.306298	6.55687	1.943348	4.754251	1.182975	2.838401	0.086136	0.085924
Cs8g06160	0.01509	0.070886	1.265074	1.111165	5.027092	4.860041	1.438509	2.2215	1.174907	0.560995
Cs8g06170	0.01542	0.108285	0.704125	0.895813	4.175775	4.528242	1.974894	2.498832	0.996081	0.540719
Cs8g06360	297.5867	150.4504	820.7326	615.6178	507.8779	576.5846	272.2735	328.1464	301.0391	360.1009

Cs8g06470	300.96	246.3192	152.3524	168.1015	87.72931	103.5696	70.66185	75.46735	43.63716	41.83642
Cs8g06530	9.16913	6.482548	7.053555	6.74476	6.408739	7.587966	7.614118	4.583157	11.42309	3.508793
Cs8g06560	0.286376	0.280338	0.288893	1.030014	0.010031	0.286821	0.019799	1.82641	0	0
Cs8g06570	17.05006	26.59852	8.972174	15.10956	3.190816	7.93423	6.631064	17.24072	0.499148	0.621495
Cs8g06580	4.779798	5.175842	4.080957	4.273505	2.618797	4.594127	1.56376	4.382581	1.146812	0.572995
Cs8g06590	1.682859	3.024306	1.57511	3.808413	0.251106	0.66655	0.38121	0.498214	0.780553	0.357776
Cs8g06610	20.16993	29.85503	9.746026	17.85323	3.075339	8.292274	8.24578	18.3832	0.6186	0.753864
Cs8g06620	18.14674	33.29081	17.21952	16.5984	1.699959	5.757673	2.307493	8.132286	0.729628	0.407183
Cs8g06630	53.01071	58.02336	37.04669	40.32893	10.00014	12.41565	3.703289	10.54861	0.684176	0.777619
Cs8g06650	22.32003	34.58829	9.226323	16.51062	3.036959	5.888756	7.307743	13.52545	0.334543	0.819449
Cs8g06740	111.6694	58.48736	80.94107	82.94823	139.3348	182.9419	122.4143	115.7447	177.3954	119.4035
Cs8g06820	124.2323	67.19272	96.83856	100.5983	205.5786	206.6321	186.1789	67.61563	36.14364	8.806663
Cs8g06935	0.560381	0.625039	0.931218	0.346041	0.720506	0.872967	0.759742	0.805439	0.355697	0.546986
Cs8g06970	6.976018	7.429147	2.225816	2.564124	1.96121	1.204791	2.894609	3.672292	0.101271	0.284612
Cs8g07020	24.94867	25.5409	35.67233	31.21697	14.10481	21.12577	7.319626	7.812431	13.23637	15.06968
Cs8g07080	19.46398	20.12901	27.72494	25.75322	7.239823	14.80056	1.5575	4.575326	8.001722	11.58684
Cs8g07210	51.3476	95.29099	21.1662	41.37187	22.19093	16.74645	33.9071	42.94447	8.953658	12.81919
Cs8g07220	17.46408	29.12854	6.49282	14.70609	7.468324	6.692916	9.640594	16.35635	3.225587	3.776933
Cs8g07230	4.601994	6.816895	11.05189	9.116741	71.38258	86.43045	103.0593	50.16348	2.819308	4.39863
Cs8g07270	2.867456	2.14597	4.5502	5.474756	6.327187	6.15698	5.961917	3.361795	0.077404	0
Cs8g07360	1.235861	0.808276	1.373894	1.462058	1.431771	2.520406	0.849579	1.571579	2.022696	0.571898
Cs8g07470	22.20148	19.66837	12.25792	10.82442	8.415612	11.95712	5.2753	11.2884	21.63958	12.95521
Cs8g07630	20.64176	20.27307	13.27253	11.74308	7.074135	8.473434	5.271	5.971942	3.123123	4.452081
Cs8g07660	7.101861	7.301732	17.33751	3.556089	14.60845	12.85432	29.1311	11.39128	9.92275	2.664598
Cs8g07665	5.41806	4.708163	5.884103	5.585879	6.571919	7.068704	4.046478	10.47522	3.151193	5.510424
Cs8g07680	2.689769	2.323578	2.418133	2.180735	1.091111	1.345258	0.893431	1.176938	1.29266	0.603145
Cs8g07700	16.40224	10.42751	11.6022	8.596486	4.299403	6.157842	3.830084	2.883718	8.681157	3.327324
Cs8g08080	2.905946	5.373759	0.7338	1.619791	0.020422	0.18247	0	0.480159	0.030381	0.056249
Cs8g08100	0.798054	0.728019	0.987733	0.879349	0.893405	1.01125	0.479418	1.351328	0.565235	0.652888
Cs8g08250	8.007825	6.165991	20.66119	14.9444	73.11389	53.54101	62.69004	51.89909	58.11504	52.54169
Cs8g08270	4.958594	5.437943	11.69132	10.37669	4.8957	17.37514	5.576009	21.96377	12.26219	38.93582
Cs8g08300	16.20236	10.68575	34.91979	29.48645	27.17593	40.89225	10.10062	13.5136	7.492841	8.606136

Cs8g08420	5.606569	5.191082	6.274493	6.035276	2.511106	12.18386	2.154404	3.935926	2.731109	0.634575
Cs8g08580	7.747534	9.638422	5.892317	7.052729	3.537318	5.104348	2.047267	4.878191	5.199178	8.113131
Cs8g08590	13.04524	15.69653	13.15443	14.57001	8.64504	13.21728	5.252191	13.75772	1.59416	4.665941
Cs8g08600	8.726017	12.91592	21.01648	21.56136	4.33187	11.48919	8.120169	21.91546	10.87163	72.6833
Cs8g08710	171.8722	341.0812	47.47011	108.168	7.247815	6.348432	4.170607	5.213515	4.092751	1.440624
Cs8g08720	7.89308	26.7702	3.735889	6.345596	6.47923	5.434899	15.22071	28.96793	6.10556	8.661987
Cs8g08760	15.1328	15.74717	8.830152	15.44006	4.020204	6.151546	2.601787	4.95241	1.377513	2.458568
Cs8g08810	1.818795	1.524102	0.285973	0.654721	0.076963	0.155625	0.118873	1.314907	0.056105	0.122634
Cs8g08820	115.9391	111.175	86.57525	88.41255	44.10278	60.87605	33.66842	115.5219	0.842212	0.537267
Cs8g08880	3.389111	3.058514	3.83868	2.956944	10.16926	8.651779	10.17421	7.510835	5.386192	4.023118
Cs8g08890	1.825907	0.973593	2.276472	1.054096	2.03394	1.576085	2.1784	1.504867	1.237246	0.314525
Cs8g08920	0.728058	0.958002	0.645223	0.767076	3.179682	1.330909	1.462549	2.025041	0.225061	0.100583
Cs8g08990	17.2941	14.01193	10.2901	8.41203	19.88285	21.44914	5.692725	8.414907	1.363036	1.00317
Cs8g09070	168.069	106.5928	37.92426	51.02949	1.09227	9.145066	1.193666	12.1804	0.970102	0.645976
Cs8g09090	64.10699	61.19404	42.36651	73.21438	17.46984	75.39305	55.13388	111.5547	9.954747	12.50299
Cs8g09100	1.779446	2.393351	1.642841	1.764169	1.134888	1.570582	0.60673	2.448325	0.474259	0.543944
Cs8g09143	1.53005	2.073992	2.530538	3.51417	3.583618	3.85349	0.940738	4.375556	2.054244	1.873993
Cs8g09270	7.900758	10.54161	9.622651	10.56339	7.041042	8.343138	3.547477	9.758465	4.737216	6.207988
Cs8g09350	150.8781	156.2687	50.29614	78.19085	7.373034	12.57632	4.007858	1.565792	0.287294	0.367514
Cs8g09390	3.844595	3.896514	16.94671	15.94553	8.687375	5.518231	8.581505	14.86459	4.536094	7.779279
Cs8g09620	2.058243	1.301435	1.640739	1.616985	0.587943	1.639237	0.507033	2.758461	2.871896	4.572453
Cs8g09660	8.536727	8.723275	10.85706	9.051071	10.68318	12.48889	6.224079	14.44538	10.21491	9.429047
Cs8g09680	27.25514	29.44084	44.12689	47.21857	32.65689	47.62682	19.29875	57.79629	23.84239	42.4128
Cs8g09720	64.43971	81.36059	63.69321	99.60619	195.2164	162.6057	801.7558	236.0707	137.2814	36.67287
Cs8g09730	6.083334	4.250584	19.10898	13.77696	33.20895	21.98634	93.38359	45.38221	25.30622	10.55562
Cs8g09740	3.844583	3.34256	2.905282	2.469073	15.8328	8.580269	33.87129	10.43752	15.01623	10.42637
Cs8g09750	12.86144	0	17.2998	9.293255	6.887808	5.643426	8.769912	11.41827	7.462507	0
Cs8g09780	0.142203	0.581256	0.281033	0.345922	9.061228	3.567795	29.16562	36.06119	197.0247	200.8725
Cs8g09870	2.375362	2.590503	4.832133	4.04238	2.218067	2.851933	1.566725	5.007417	2.373554	4.101065
Cs8g10135	2.144932	2.014107	2.287731	2.494297	3.406955	2.479179	1.062939	3.422734	1.864934	1.501066
Cs8g10200	0.498584	0.469699	0.885121	1.549352	1.36173	1.614943	1.894417	2.439849	9.485133	8.245605
Cs8g10210	1.823787	3.730813	2.027728	1.914532	1.340886	1.19653	1.533413	0.171037	1.009822	0.288324

Cs8g10215	1.096859	1.454683	1.299829	0.996344	0.621981	1.094507	0.083916	0.989919	0.333236	0.437903
Cs8g10260	38.16721	38.04752	30.48617	24.07322	22.32485	16.13687	14.68699	11.77529	6.653487	6.393302
Cs8g10300	0.356405	0.463007	1.461776	2.090044	0.178702	0.209256	0.26049	0.754302	0.297658	0.464825
Cs8g10370	28.55418	17.13631	20.52715	18.86556	11.05606	22.49926	11.41671	13.10495	13.54718	8.278872
Cs8g10390	6.354374	6.241778	2.152227	3.368374	0.660546	1.113482	1.170818	2.259107	13.7968	23.33061
Cs8g10420	0.283806	0.27892	0.65223	0.466155	0.625334	0.92343	0.272209	1.013894	0.714481	1.395024
Cs8g10660	3.107253	3.21506	1.726264	1.859055	2.266529	3.67895	0.858592	5.283721	0.391372	2.229538
Cs8g10700	7.217321	6.153814	9.771644	10.72561	18.14679	16.89297	11.89682	16.90606	20.11193	13.81408
Cs8g10800	30.27929	35.90523	15.49094	24.14823	8.743887	7.766304	5.666251	10.6789	6.191621	7.63954
Cs8g10810	1.187442	1.462499	1.571226	1.909439	2.34971	1.220747	0.976872	1.172622	0.5944	1.751814
Cs8g10860	76.03857	101.7313	71.19243	33.31466	32.87045	47.07231	38.16712	39.90681	2.023255	9.104114
Cs8g10910	28.87702	31.9937	26.3336	26.30921	20.8712	17.14715	6.925661	16.98707	2.43137	3.961548
Cs8g10950	9.842051	4.806184	14.48681	10.96552	47.15738	96.15573	3.083284	12.84334	0.845816	4.33617
Cs8g11063	0.95751	1.092332	0.794561	0.72639	0.562469	0.915888	0.360089	1.115662	0.329959	0.469682
Cs8g11066	4.813821	5.988152	5.293399	4.895098	4.624093	5.077975	2.802114	6.960184	2.519453	4.489429
Cs8g11315	1.390359	1.953891	1.728268	2.156269	0.940833	1.475371	0.383599	1.838242	0.434826	1.449344
Cs8g11330	17.4276	19.20627	12.72748	12.69828	5.429419	12.50815	14.98122	23.53416	158.6895	61.15438
Cs8g11370	0.258469	0.323368	1.282929	1.283455	0.321304	0.69235	0.307881	1.518144	0.094746	0.75367
Cs8g11500	2.813341	2.664397	4.197164	3.148163	3.105728	2.648079	2.46392	3.680069	6.824355	14.67714
Cs8g11514	700.0072	956.3552	70.36222	151.2803	9.840507	17.19666	2.713015	11.62211	1.38187	6.657512
Cs8g11520	4.658833	4.725782	3.890918	4.622248	2.78136	4.610916	1.405885	4.677288	3.605252	2.851295
Cs8g11590	0.054572	0.182616	0.05321	0.015883	0.063469	0.12285	0.191083	0.845973	0.630648	0.588927
Cs8g11970	8.787192	9.721329	10.49831	12.46076	8.888515	12.82249	7.566553	17.78385	11.64246	16.06691
Cs8g12000	3.982738	5.619146	10.83935	11.74065	3.110119	10.1723	14.81249	20.93313	6.582653	30.6783
Cs8g12020	0.820771	0.550077	1.320081	1.524647	0.7418	8.994482	2.973396	23.1109	0.247375	0.657483
Cs8g12070	21.44465	15.27222	10.88957	8.010814	22.88739	15.808	8.847764	4.725547	15.64557	3.030119
Cs8g12085	8.931433	10.59004	12.29291	12.3901	7.807876	9.4422	2.125551	9.512978	2.363959	2.171629
Cs8g12370	1.878582	3.416425	12.34566	11.73122	1.049617	5.38555	2.867617	9.906597	2.49035	12.17167
Cs8g12450	43.16274	29.37728	31.12718	31.38786	43.29021	19.6379	42.50738	17.53185	11.10014	6.982656
Cs8g12480	38.01374	31.21199	78.70145	62.87107	116.2542	107.0367	199.3429	106.4553	95.88018	106.0428
Cs8g12530	2.471388	1.021161	2.812787	1.859731	0.701501	1.143194	0.534314	1.5733	1.189807	0.532546
Cs8g12640	4.706771	6.261372	5.306068	5.618471	16.69823	13.10972	36.66191	58.60894	36.87345	35.65384

Cs8g12650	37.03175	26.30461	33.7177	32.68618	40.05829	35.12033	44.02562	22.29126	40.97637	16.51464
Cs8g12690	28.14722	22.56236	27.17519	36.26865	33.66745	87.94288	35.37943	25.32834	56.27144	19.01113
Cs8g12710	5.535075	4.348037	7.039911	7.373331	0.677202	3.370575	0.807592	3.560414	2.242793	3.037609
Cs8g12715	1.568717	1.424838	1.539928	1.603455	1.701506	2.371651	0.519916	2.071445	0.251146	0.44139
Cs8g12730	2.857514	1.790596	3.105987	2.346869	4.257413	6.353371	1.959359	1.872567	0.190339	0.135289
Cs8g12830	3.446342	3.512156	3.058167	3.486514	4.017311	4.407284	0.827619	3.663372	2.536387	2.568064
Cs8g12880	4.133045	1.894503	6.066907	3.516788	16.61184	17.10815	10.12916	5.67611	10.2867	8.10152
Cs8g12910	8.80635	7.961798	8.751273	7.929678	9.523503	10.69921	5.782547	12.26314	7.69377	7.125969
Cs8g13170	11.56644	10.5194	3.665309	5.668977	3.144255	6.729092	5.003665	5.449083	6.646472	4.218488
Cs8g13190	11.33434	5.777989	8.482354	6.512435	8.720307	8.169969	9.58006	6.851226	27.15618	8.613523
Cs8g13283	2.689383	3.423278	3.287006	2.803553	3.186582	2.619916	0.903053	2.989894	0.5624	0.982972
Cs8g13286	9.44186	10.11198	9.944859	9.579601	7.194711	9.939594	2.441845	4.213781	3.865527	4.380075
Cs8g13530	2.803618	4.829789	6.58592	8.21861	5.278249	6.04601	1.144584	6.945349	1.335028	5.78599
Cs8g13600	0.468178	1.329277	3.422506	2.337683	1.81826	3.128843	0.607431	2.827886	0.187521	2.939065
Cs8g13660	323.101	542.4499	177.9865	312.0378	57.92046	70.22023	32.90703	170.8458	21.02733	54.29006
Cs8g13680	20.44976	19.18747	24.95343	16.40454	41.98875	15.92268	4.620473	9.473206	1.769838	3.026573
Cs8g13700	0.729156	0.764096	1.78012	1.781427	14.6863	8.067432	18.37888	17.36063	0.791889	1.005607
Cs8g13710	0.904714	1.180575	0.513997	0.802093	2.182122	3.048174	2.036681	2.801834	1.352664	8.492991
Cs8g13730	0.818559	0.954079	0.499456	0.597057	0.960409	1.615393	1.913696	0.962431	0.845039	3.142768
Cs8g13740	261.7904	227.277	186.4661	158.7128	167.5984	117.9577	68.96879	202.2302	114.3996	219.148
Cs8g13770	17.75557	17.93631	13.61776	13.53826	7.932377	7.94692	10.24678	8.837253	2.760455	4.252111
Cs8g13780	8.383668	7.271095	15.47221	12.13392	8.102644	8.467059	4.652161	5.589506	1.438003	2.218118
Cs8g13800	7.630131	9.113416	4.015337	5.1819	1.161841	2.419347	1.324824	2.351618	0.152097	0.197599
Cs8g13820	1.550718	1.103542	2.264957	2.036645	2.980002	4.332341	0.767799	1.264825	0.092595	0.171384
Cs8g13910	3.419686	2.494104	4.214345	3.388683	2.864568	1.830386	0.385226	0.999307	0.019305	0.007734
Cs8g13965	3.750567	3.59629	5.305989	3.599598	5.024259	5.288516	2.20418	5.580178	2.285046	1.119971
Cs8g14010	3.261364	3.633329	6.705185	5.489286	2.160951	3.018962	0.645268	1.839644	0.149564	0.290233
Cs8g14040	12.16647	14.62759	10.34405	14.02151	1.837275	2.269496	0.286149	2.120633	0.413489	0.563296
Cs8g14080	5.229588	7.623153	3.803052	4.94697	2.272146	1.729148	1.23241	3.556416	0.528427	1.155845
Cs8g14120	524.4674	380.2635	796.7386	699.7477	375.1333	456.1613	313.0423	100.8907	682.6646	436.2414
Cs8g14150	136.436	57.88866	74.10427	131.6808	40.98418	117.0743	422.2937	597.4333	125.2191	106.8466
Cs8g14190	2.657541	2.542162	0.438858	0.974497	0.211419	0.40776	0.06227	0.359257	0.627655	5.452274

Cs8g14280	4.937899	7.917024	19.69797	31.26174	56.97687	105.1041	70.32756	85.17837	406.0069	446.7368
Cs8g14360	51.03078	60.82433	95.13907	77.28661	175.8942	141.5245	219.7756	128.0669	297.0436	203.5553
Cs8g14510	2.725183	4.194996	3.42582	2.145294	0.816056	1.338441	0.692222	1.578686	0.11674	0.072141
Cs8g14600	6.089044	18.09216	14.57642	24.25652	7.337137	7.510096	22.8789	25.87637	57.65292	14.71094
Cs8g14690	1118.631	1017.617	797.6785	922.7549	262.7835	424.7649	174.5237	371.892	1.966708	1.972675
Cs8g14700	38.6555	76.72962	86.23979	129.0578	5.477193	14.58032	11.89313	66.7229	5.61205	69.85184
Cs8g14740	0.142177	0.027068	0.347769	0.875214	0.10687	0.381914	0.046874	0.356608	1.430075	19.98834
Cs8g14800	1.420812	1.289901	3.38649	4.176982	1.437754	2.498305	0.183338	0.880877	0.326925	1.030376
Cs8g14810	5.238867	4.09377	14.10118	18.98412	6.041073	13.45035	0.325051	5.245467	0.435875	3.073179
Cs8g14840	2.249154	1.821625	6.091937	12.16808	4.11714	8.496664	0.212952	1.353747	0.072725	0.439864
Cs8g14850	0.900466	0.650156	1.158086	1.504625	1.454663	2.539843	0.772487	2.533111	1.360409	2.355652
Cs8g14950	46.76741	19.74481	28.09524	48.83267	6.981776	51.63443	31.89872	15.50484	871.7214	632.4135
Cs8g14960	107.3498	118.0945	187.0007	204.0416	598.4069	582.0947	1318.034	1004.829	2278.206	2228.941
Cs8g14980	2.162569	4.563086	1.786414	2.481339	37.56152	15.98774	53.91491	37.8069	0.351384	0.151232
Cs8g15060	0.214262	0.265412	3.268634	3.351396	2.572185	11.2491	2.509931	9.737971	0.378926	0.701865
Cs8g15070	6.108115	6.115348	14.67875	10.87547	44.95634	50.19803	14.65758	32.28084	19.35304	25.68231
Cs8g15150	173.4282	237.4604	273.3987	314.3024	55.31117	94.8332	148.7786	269.3343	106.5706	444.9384
Cs8g15240	30.76736	17.472	33.53427	29.25066	9.914299	25.30987	7.109752	11.57195	4.498662	1.381241
Cs8g15250	9.088646	4.856152	9.873074	9.166485	2.783445	7.735555	1.752009	3.557171	0.858668	0.469775
Cs8g15290	24.23045	32.52691	19.67962	14.98101	6.554064	6.594388	0.842158	17.53151	0.081856	0.020932
Cs8g15340	2.519697	2.707804	11.43418	13.3128	19.04413	22.52595	17.95846	26.15288	10.74014	17.19673
Cs8g15350	1.527064	1.501983	3.865266	3.723007	2.777921	2.261671	2.556479	6.021858	1.511695	2.816735
Cs8g15390	1.585852	2.065491	7.477445	6.75523	9.558022	5.206348	6.962411	13.71316	3.577904	4.077088
Cs8g15410	2.032737	3.061309	1.95956	2.166766	2.425779	2.118583	0.881682	3.967222	0.469593	0.617337
Cs8g15500	31.88384	32.97339	34.0441	37.68868	21.23837	28.49784	15.74746	46.78782	15.08539	26.23659
Cs8g15610	6.08393	5.103704	1.221777	1.158085	0.545744	2.169192	0.443313	4.593393	2.069853	5.750254
Cs8g15670	14.96547	14.56449	10.1388	10.48985	2.783105	6.729195	1.196063	3.331999	0.418153	0.415335
Cs8g15710	16.3456	17.22118	12.19607	10.7377	14.6687	13.13765	10.47107	14.35377	3.300088	13.90526
Cs8g15780	0.842402	1.204034	0.90019	0.912547	0.449682	0.641526	0.280368	1.18343	0.416901	0.158538
Cs8g15890	243.292	176.4817	198.7984	187.2444	152.6175	169.6227	24.49269	57.26823	94.07599	108.9408
Cs8g15970	85.60832	63.34992	127.5093	97.99155	220.0777	172.9983	268.6422	118.3854	193.5845	135.3786
Cs8g16090	3.673547	4.123481	3.377839	3.840995	2.493854	3.229389	1.762961	5.749131	2.056585	4.731599

Cs8g16140	27.58547	14.66303	3.660984	3.952527	2.643899	5.112513	3.368456	3.735706	32.31826	9.847048
Cs8g16290	0.403414	0.597679	0.794794	0.704096	3.75067	2.780687	15.44345	10.16594	17.72884	9.813489
Cs8g16360	189.2455	134.9682	151.0507	168.9249	82.81854	164.6902	53.89516	149.8573	25.45047	20.86231
Cs8g16430	4.414835	3.423095	2.468617	2.02626	1.346396	1.580568	0.733244	1.393481	0.077201	0.05747
Cs8g16540	53.26689	68.8756	37.75222	39.88302	40.94903	38.1372	22.48346	63.20447	5.416408	14.06527
Cs8g16610	1.0608	0.590689	0.290304	0.379996	2.595315	1.789399	1.782643	0.552577	1.620804	16.68868
Cs8g16640	153.5702	288.4414	47.07201	70.50839	61.10803	33.02206	118.6758	93.33328	76.40121	17.15164
Cs8g16650	1.57927	2.055391	0.992309	2.083523	0.289823	0.713739	0.055679	0.080479	0.25542	0.115095
Cs8g16660	256.9879	225.7565	299.3086	303.9235	199.7197	187.1311	73.145	144.3059	120.2253	155.3293
Cs8g16700	179.4396	172.0126	336.7546	331.2997	588.7006	654.3237	747.0014	444.1579	702.9667	731.3318
Cs8g16750	15.75875	14.45583	22.90839	19.32219	41.3635	38.35148	56.85451	48.98881	73.85641	64.73519
Cs8g16770	6.332638	8.30737	9.45126	9.831488	17.07741	13.4462	27.55319	21.54682	15.06418	17.1922
Cs8g16800	45.29004	41.43544	17.94139	20.3008	8.252414	15.36884	9.989163	7.130848	0.731465	0.172099
Cs8g16810	5.713229	6.589816	7.222586	8.827722	5.556499	11.02052	2.061724	5.967953	5.736991	5.074343
Cs8g16850	42.55034	49.13462	30.89434	36.0751	25.88032	56.97356	25.53249	40.72399	27.73414	22.25631
Cs8g16930	40.8327	40.24136	40.2272	45.98311	21.35042	24.38451	8.735652	9.366828	1.865092	2.251774
Cs8g16985	7.61959	11.00454	10.36275	8.29538	4.04295	3.656966	0.944474	3.99381	0.570277	1.516118
Cs8g16990	268.2778	308.0389	421.3884	214.2176	475.7999	305.8931	590.4114	232.6162	414.5625	332.4838
Cs8g17000	239.6961	213.8374	315.237	281.206	348.5795	299.3636	535.8118	248.3352	341.4827	285.6242
Cs8g17010	209.2382	188.1601	274.8039	235.4042	325.0393	275.9273	597.8964	251.885	303.4093	250.535
Cs8g17090	2.066873	3.023005	8.397767	9.13347	1.517309	3.250929	1.853395	5.701345	0.534931	1.738109
Cs8g17160	5.092621	5.132536	6.204645	5.327811	12.909	4.569385	70.1498	17.8221	18.58971	26.92335
Cs8g17190	1.155778	0.948061	2.596815	1.641563	7.670894	6.45926	5.637091	4.716172	8.944978	7.949193
Cs8g17200	37.09652	29.06505	28.81664	34.46787	10.16015	24.64628	14.94335	31.11915	48.67104	76.37155
Cs8g17220	61.28412	45.489	94.94864	73.15858	113.8104	69.53061	36.39	44.59744	8.483999	30.7167
Cs8g17240	3.637325	1.763506	1.694628	1.450159	0.677171	1.071941	1.002862	3.068088	0	0
Cs8g17270	1.792232	1.817123	1.376561	1.594046	0.564632	1.115771	1.40287	2.28362	3.842619	2.19296
Cs8g17360	78.32561	110.4814	109.456	166.4896	23.91371	42.85387	47.77268	163.7777	80.98448	374.5351
Cs8g17373	0.974945	0.689307	0.938855	0.860066	1.285576	1.137771	1.143821	1.540955	0.384097	0.957785
Cs8g17390	45.87197	38.59588	44.11807	49.42811	39.56278	44.5433	8.889511	11.68129	28.41917	10.65152
Cs8g17420	17.07239	12.53901	5.110453	8.056704	2.243096	3.677692	1.14947	1.451439	0.442941	0.209346
Cs8g17430	18.07865	13.46797	6.611071	6.344534	2.327354	3.126497	1.111155	1.26004	0.543728	0.209162

Cs8g17520	1.500085	2.373644	2.461378	1.684	28.07158	34.47257	13.35319	23.32552	34.84759	41.04562
Cs8g17550	12.78792	14.22706	16.21496	14.82784	13.45874	12.31791	10.04377	9.932856	5.591177	2.323873
Cs8g17580	0.590765	0.586406	0.649659	0.633918	1.465444	1.331656	0.954828	1.276664	0.857022	0.850755
Cs8g17790	6.066931	5.458288	13.45305	10.98624	18.54552	21.03733	24.42077	23.45503	19.56961	22.64133
Cs8g17800	7.699183	5.69777	15.62312	8.127427	31.10229	46.97763	24.44842	70.53046	4.055326	3.732931
Cs8g17810	5.796629	14.98253	4.56011	7.473117	0.69573	1.002189	4.896879	10.44977	16.97283	32.82172
Cs8g17830	11.84425	9.866591	9.6007	13.54671	44.44741	40.79212	16.63139	93.34785	0.749072	0.645828
Cs8g17890	1.558854	0.887685	3.122913	2.258603	3.274494	5.027425	1.568541	3.41835	3.395768	3.797057
Cs8g17900	0.4207	0.107623	0.180875	0.319917	1.194345	0.59982	2.143625	0.093687	1.124412	0.125336
Cs8g17930	1.993315	1.652535	2.498072	2.469976	1.115601	3.451333	1.461897	1.254011	0.83307	2.748071
Cs8g17940	1.985677	2.339591	2.50384	2.338237	3.870718	3.464554	7.265414	6.404265	4.212308	4.475779
Cs8g17950	10.59693	9.850981	6.187028	9.572797	1.360321	4.640477	0.287602	1.764747	0.986155	0.447042
Cs8g17960	70.13599	139.4694	119.9844	140.62	8.663542	24.955	33.89684	121.1316	35.44096	127.1401
Cs8g18000	0.852714	0.617741	1.461469	1.296744	3.182371	3.204249	2.810917	3.174656	0.485328	0.470431
Cs8g18020	2.665622	2.910192	7.075338	5.466785	3.649464	3.052693	6.667835	13.83195	18.27155	23.13849
Cs8g18050	9.112992	10.7257	1.904024	5.032906	1.547499	0.887384	1.283438	1.125375	0.26031	0.262644
Cs8g18200	80.66188	48.67352	36.90159	32.81603	50.15804	58.18452	41.85445	10.95109	76.29218	29.39772
Cs8g18290	3.616402	7.686142	4.963598	7.318761	0.958388	2.157363	2.397579	16.69712	2.006746	9.888284
Cs8g18300	83.80501	86.38446	68.58794	82.0493	30.44533	32.70577	62.366	134.793	177.2707	199.4429
Cs8g18310	30.4044	28.35058	14.66643	17.356	3.74918	3.866456	1.235265	1.36741	1.457205	1.084484
Cs8g18330	7.677258	10.13958	9.671998	10.38424	7.450321	8.400852	8.472947	11.20945	6.532085	13.26874
Cs8g18350	6.183938	6.051195	7.240064	6.473302	7.159794	7.887479	2.266743	6.239788	3.700409	6.591685
Cs8g18360	52.0969	70.31251	119.5674	100.7682	98.76323	71.91797	109.8803	329.6584	141.1064	172.8157
Cs8g18380	4.780507	4.508344	6.93156	5.16574	7.452799	4.917857	2.550851	6.165594	0.314269	1.352636
Cs8g18400	11.1482	10.16594	8.68793	7.358438	17.77228	16.9843	21.63563	13.38637	15.77895	19.09123
Cs8g18430	14.64934	18.03855	16.23881	18.64076	7.037478	8.173413	6.983794	13.97445	11.45948	19.74005
Cs8g18450	7.126501	6.133511	7.825707	7.57402	5.424305	6.662255	3.172102	2.367809	5.318034	7.12705
Cs8g18710	10.4399	12.70393	11.91296	14.444	4.208662	5.043623	7.767454	11.76227	2.982555	8.348674
Cs8g18740	2.393429	2.873969	5.605567	5.964561	6.723575	7.787834	6.110199	10.71019	10.71709	15.96563
Cs8g18760	1.442567	0.957203	2.303451	4.278085	0.751592	3.63146	0.439621	1.845669	2.367169	3.620165
Cs8g18780	70.13695	44.65068	15.15111	19.13164	8.668324	16.19866	25.36166	5.078539	24.06978	3.3599
Cs8g18810	4.138907	3.465785	10.38154	9.651231	8.119811	8.787609	3.288899	3.861599	2.842932	3.073251

Cs8g18830	58.57827	106.4672	84.71491	85.40871	35.01325	35.08347	50.66426	99.85596	49.40411	77.59362
Cs8g18850	5.721805	7.527259	2.786393	3.761634	2.067835	2.1061	0.607589	2.700107	0.349191	0.925359
Cs8g18860	4.586704	1.372846	5.79838	2.944768	12.59526	15.20951	5.173826	3.223127	4.325229	3.299115
Cs8g18970	52.09415	51.31707	48.98295	52.341	33.66404	38.27628	11.42378	23.41266	13.14316	18.63867
Cs8g19010	37.48642	55.08126	18.53263	30.62818	3.862241	6.939059	2.403592	6.716695	2.382911	3.671584
Cs8g19110	0.420038	0.136509	0.154464	0.262153	0.161262	3.989739	0.922831	1.300824	2.137366	1.012618
Cs8g19140	95.82055	70.853	95.1669	55.67157	95.18399	41.97917	67.65964	47.7497	143.0937	58.96084
Cs8g19150	16.29894	15.96576	17.38041	17.76682	12.79476	12.43448	5.576158	16.60007	2.978852	4.322655
Cs8g19230	5.836743	5.655972	6.062354	8.39338	1.189857	4.107935	5.074924	8.987805	8.693701	19.01501
Cs8g19240	31.76422	30.55606	26.91451	29.81258	34.42992	26.65614	16.15212	17.58466	89.05941	26.73495
Cs8g19260	4.825899	2.643142	2.335282	1.58472	3.06261	3.019899	5.055226	8.434842	12.30068	13.6089
Cs8g19280	10.39494	9.02878	13.22757	13.42644	16.79295	19.86867	31.49202	55.49014	12.99723	15.15889
Cs8g19390	0.850587	0.678489	2.255216	2.863176	0.680426	3.896218	0.258085	0.444659	1.365605	1.605405
Cs8g19400	2.004487	1.512971	7.80988	8.130936	4.652634	11.59049	2.483626	6.030931	1.921818	1.900464
Cs8g19420	4.357806	3.729921	8.898379	5.297339	13.32942	6.894598	6.119746	11.54392	0.843373	3.326023
Cs8g19440	1.13439	2.157163	10.57808	12.8141	7.897497	9.96067	3.757487	15.7441	1.995763	4.155125
Cs8g19450	0.794497	1.019778	5.770092	6.021198	13.70354	18.91391	11.21385	23.31724	2.894406	1.547437
Cs8g19470	0.151641	0.19882	0.256133	2.546347	0.018795	0.222981	0.010379	0.196153	0	0
Cs8g19480	11.34903	9.021344	32.77026	27.33001	61.44735	58.09839	50.70238	60.87037	52.42015	55.09608
Cs8g19490	57.247	56.99677	78.74614	69.28536	82.35736	64.95334	207.4759	305.7528	86.27755	75.69674
Cs8g19500	11.14906	9.022563	31.91947	25.95295	58.42153	53.55871	49.77987	58.28408	49.66172	50.2847
Cs8g19520	8.348165	6.375274	5.03582	3.506392	6.303848	4.826952	15.20883	26.85239	12.97556	11.14957
Cs8g19540	1.593198	0.613736	1.244973	0.929911	2.802922	2.675089	6.840604	6.8146	4.055214	5.907212
Cs8g19650	8.288198	5.846563	4.737251	5.020406	5.092345	12.99979	11.93196	11.02562	12.64193	9.533975
Cs8g19720	56.63884	36.87615	0.621537	1.243982	0.943505	0.735517	2.265003	1.942828	0.398227	0.491291
Cs8g19730	4.848066	1.375593	12.86112	5.687602	40.72876	31.90857	6.48054	7.117415	1.763312	2.930084
Cs8g19740	0.343573	0.19341	0.524918	0.461544	0.8832	0.50704	1.114971	0.993991	1.710689	2.238889
Cs8g19800	19.09098	14.46088	45.75973	31.42208	92.40756	54.4129	107.6395	52.0059	17.63985	19.14659
Cs8g19840	18.59736	15.43858	25.90559	24.61966	48.68881	75.8919	54.94284	43.66056	195.9136	61.44416
Cs8g19950	0.857063	0.94767	0.568236	0.719608	0.463739	1.50887	0.544906	1.033525	0.051174	0.045061
Cs8g19960	22.86817	46.43524	13.30956	30.13814	3.674996	5.180192	5.142165	11.13392	2.7545	2.233019
Cs8g19990	30.47956	32.55867	9.155454	10.26509	3.226135	4.319995	0.902252	1.183682	0.109762	0.160404

Cs8g20020	3.618376	3.897107	11.37057	7.829229	22.42023	19.94805	15.70987	17.73695	29.2863	22.05915
Cs8g20070	5.400838	6.041823	4.205823	3.770513	2.170283	2.594058	2.577063	4.885104	1.806694	2.235985
Cs8g20100	11.93759	18.16976	9.966766	9.883921	12.91548	9.545294	8.037651	20.37563	5.279494	7.335093
Cs8g20110	0.335522	2.450901	0.252787	0.551317	0.23095	0.111727	0.037764	0.236905	0.203098	0.683338
Cs8g20130	0.141538	0.188173	0.047089	0.055568	0.01627	0.238473	0.087298	0.042191	5.743645	2.255357
Cs8g20240	43.93566	19.52038	31.05701	39.12251	8.770543	37.2658	5.222618	17.99328	41.1714	52.05757
Cs8g20280	4.608881	3.830629	3.35089	3.511266	2.261617	2.559321	1.843803	1.308204	3.126009	1.543452
Cs8g20310	77.01114	54.71726	170.6819	130.9113	107.7425	101.7308	75.47129	90.02069	45.01856	8.88724
Cs8g20410	187.9564	222.0826	19.90898	30.25803	2.732301	4.141277	0.832748	2.59286	3.890533	21.78484
Cs8g20420	29.38928	55.58049	73.15426	75.05129	16.7177	31.21491	37.10506	96.85739	39.92745	86.69528
Cs8g20480	18.37995	22.21915	24.63637	18.52994	34.50007	19.57996	39.64858	29.37959	4.107743	4.648427
Cs8g20490	1.037162	2.148568	0.633944	0.422969	0.497623	0.673449	0.458174	1.2943	0.021569	0.023101
Cs8g20580	15.31975	18.03208	11.52693	10.7577	4.664562	7.977735	3.278036	9.034495	0.731793	0.847769
Cs8g20630	0.498401	1.255816	0.648098	0.627768	1.058051	0.326143	1.047325	3.474773	0.175455	0.105027
Cs8g20740	1.531277	0.358482	2.32118	0.972097	1.243065	1.974046	0.07874	0.043458	0.116654	0.010862
Cs8g20750	43.46874	44.06943	68.74438	56.27526	79.45805	72.25913	171.9627	148.5848	54.95226	85.49381
Cs8g20760	7.647188	4.912348	7.156036	7.886605	11.64926	17.60912	14.22518	13.58174	17.14212	6.886259
Cs8g20790	5.731526	8.343945	5.06295	7.24069	4.387747	5.07321	2.505684	6.770801	3.065293	4.863256
Cs8g21020	1.407606	1.501523	1.618269	1.445934	2.084124	1.485983	2.332556	3.666535	1.43262	3.135603
Cs8g21060	1.799884	1.554361	1.145584	1.037393	1.56759	1.313816	0.649463	1.194737	0.449093	1.369926
Cs8g21070	15.05079	21.9236	7.29431	11.72807	2.899622	2.939117	0.87956	2.975083	0.155792	0.319483
Cs9g01010	4.258913	5.293289	7.345908	5.578425	4.413084	2.346489	3.485237	5.284055	2.431388	1.860659
Cs9g01150	8.456242	11.23608	13.70786	13.4813	6.018219	11.3121	21.38814	27.86198	4.086855	14.61875
Cs9g01160	2.377585	2.493129	3.830521	3.805167	3.709069	3.40774	3.815179	4.159115	1.20147	4.000176
Cs9g01250	35.12274	42.15065	16.96443	14.81657	14.32346	3.726082	4.269767	1.848711	3.108131	2.158583
Cs9g01410	50.20735	53.84949	112.1691	157.6766	215.0582	287.2784	184.2184	191.2801	263.7994	434.9473
Cs9g01430	6.673867	7.673116	3.804031	4.521516	2.106568	3.686256	1.552573	5.388374	4.765296	10.45196
Cs9g01620	9.841127	4.335875	4.085422	5.09796	2.333243	8.620222	2.569054	10.0532	5.115808	10.27906
Cs9g01632	2.321264	1.708459	1.93778	0.594361	1.275381	0.832102	1.975939	0.988203	0.869736	0.09247
Cs9g01634	2.536639	1.811266	2.33867	0.746123	1.355626	0.829189	1.04863	0.499837	1.04635	0.546532
Cs9g01650	6.80284	8.507689	5.286636	6.822445	3.453212	4.571542	2.659135	7.312292	1.131153	1.463203
Cs9g01680	16.6598	19.70559	25.7608	21.37096	20.01886	27.26183	11.98958	33.34862	17.32435	35.06187

Cs9g01690	24.59139	22.79637	31.48301	33.30434	18.95037	21.54352	11.07467	27.57893	10.12983	15.13977
Cs9g01780	16.98904	29.30419	15.29593	22.56931	2.790189	2.865166	2.617853	11.53936	0.536337	1.584565
Cs9g01800	0.136433	0.275446	0.551173	0.509216	0.865615	7.04067	0.897523	4.502301	1.556314	1.247916
Cs9g01920	30.72916	19.68452	15.4833	15.95033	11.09114	18.63634	5.234131	18.11272	18.44166	7.655865
Cs9g01970	5.810776	8.384883	2.705036	3.271618	2.353086	1.658034	1.669571	3.705853	0.277304	0.79432
Cs9g01980	24.09465	24.70616	11.60477	12.57417	6.871579	12.24468	5.557845	11.26561	10.64619	6.171094
Cs9g02040	24.94021	26.43133	27.61278	34.29466	13.05675	19.25171	18.60948	51.81499	52.29799	118.1114
Cs9g02055	6.521773	7.292341	11.64233	9.450096	16.25424	17.23227	10.68296	20.38529	4.337637	9.832207
Cs9g02090	3.753808	22.0142	7.291345	7.857331	17.57569	41.19422	4.221553	12.82832	24.65875	17.34851
Cs9g02130	12.5771	10.61032	11.3909	12.5358	6.828011	13.01959	5.425747	7.698822	13.58058	10.89799
Cs9g02140	0.868127	0.202981	0.057058	0.1242	0.035682	0.028957	0	0.01591	0.287716	0.085999
Cs9g02190	315.9593	168.6869	299.6451	268.6797	472.4946	443.8539	615.2757	903.054	163.2154	241.202
Cs9g02270	0.143859	0.045632	1.505112	0.870724	1.817292	2.109637	0.80056	0.872726	0.44587	0.282674
Cs9g02280	13.51048	26.94917	3.868256	11.78077	10.25275	7.864253	7.196401	11.63605	2.817819	1.884197
Cs9g02390	7.250113	11.36657	8.810869	9.144197	6.235088	3.483894	6.180027	15.19851	18.46186	30.67549
Cs9g02530	25.2971	22.21066	42.4346	40.32453	76.15191	86.76785	86.97831	56.2538	226.1336	87.70176
Cs9g02540	15.13703	16.22821	25.59226	21.0994	12.45674	11.4679	4.818341	10.97536	1.544058	5.528283
Cs9g02570	0.74254	0.844613	0.406026	0.42451	0.82894	0.733098	1.231548	1.772781	4.32156	8.603438
Cs9g02580	11.03729	8.662152	6.659526	7.501871	4.313305	10.54081	2.568723	5.520067	1.665297	1.442965
Cs9g02710	24.55172	20.23976	8.611004	10.71974	3.509796	5.406631	3.222793	7.962824	0.850155	1.337725
Cs9g02720	11.77469	17.51637	10.87922	12.77013	5.778179	7.922987	15.76535	33.13664	17.26293	48.21195
Cs9g02740	1.726105	1.591427	1.485812	1.164768	1.64457	0.86984	0.850173	1.837649	0.404031	1.123866
Cs9g02790	0.435419	0.922034	0.755153	0.636572	0.123507	0.110489	0.194134	0.055578	0.546338	0.085108
Cs9g02820	0.365014	0.438681	0.02978	0.30527	1.058854	0.195545	1.471648	1.406763	0.033591	0
Cs9g02830	0.555332	0.57403	0.393454	0.354621	0.854858	0.393077	0.333163	0.218946	0.022511	0
Cs9g02840	0.711939	2.562324	2.876861	5.571227	11.14865	19.6291	1.581921	5.04337	0.806119	1.874653
Cs9g02930	0.940017	0.595617	4.889585	6.354812	12.76459	4.532202	3.203227	5.621375	5.474821	5.78726
Cs9g02990	38.90061	89.20328	5.295152	27.22422	10.75253	8.010934	9.295338	20.84271	2.714444	4.667843
Cs9g03000	109.8368	118.8773	160.9215	113.6312	91.37338	75.66222	21.54963	32.84425	2.07055	2.385231
Cs9g03050	6.003834	8.433517	6.909117	8.033024	5.499675	4.667741	2.865643	6.386602	2.867732	3.344414
Cs9g03060	0.534452	0.278594	1.245426	0.608421	0.81641	0.770754	0.562659	0.56831	0.51214	0.543204
Cs9g03190	48.84427	51.69348	28.59166	27.04606	19.83352	22.99317	7.459784	10.25705	1.576901	1.105937

Cs9g03280	14.93514	28.2701	8.66453	16.97219	9.673562	9.24757	7.277155	22.98377	11.37806	13.8409
Cs9g03320	23.32541	13.11423	14.66265	13.81262	19.45604	20.52329	17.78355	22.04677	38.05414	24.95595
Cs9g03360	0.708414	0.395551	0.553873	0.780549	0.231305	0.997705	0.038592	0.159966	0.058735	0.353543
Cs9g03570	70.18287	46.64506	86.65068	64.30635	215.0696	127.7548	809.2721	504.9676	39.2832	63.66978
Cs9g03590	0.705534	1.883379	0.769781	0.982816	0.239037	0.171228	0.197433	0.142124	0.613275	0.067183
Cs9g03610	48.9537	56.43154	150.122	63.45549	133.4378	79.16393	71.72286	69.84083	2.490446	4.509797
Cs9g03630	487.9496	474.8869	1295.041	1002.607	3196.823	2875.365	1989.292	2328.104	462.248	708.2471
Cs9g03660	14.35557	11.57916	31.46005	23.7647	73.37115	65.22869	55.76806	78.34469	68.44628	153.3439
Cs9g03670	0.328453	0.302071	0.454634	0.404388	0.930125	1.033166	0.679893	3.126559	0.772166	0.721536
Cs9g03730	16.86479	17.50811	14.26359	16.04111	8.098814	9.977654	6.650871	22.21886	11.30623	15.85127
Cs9g03770	7.17263	10.306	3.562313	3.971778	3.354243	2.264608	2.541886	4.352976	1.178478	1.432548
Cs9g04030	15.11447	26.87851	22.78118	25.40706	3.875375	4.267517	12.17513	24.60766	4.16639	16.23384
Cs9g04050	0.255721	0.449483	0.631748	1.223891	1.609886	1.0859	1.087112	2.101906	6.793212	10.73516
Cs9g04130	0.924714	1.756637	7.569823	4.902341	6.132079	6.678569	4.68212	33.04413	3.471202	12.72507
Cs9g04140	2.429287	3.346543	16.04962	13.29838	14.47391	16.02038	16.68003	67.13969	12.57794	35.5736
Cs9g04180	9.664951	14.32894	3.940482	6.083722	1.31057	2.613946	2.46813	3.435085	2.143098	3.731364
Cs9g04190	5.538257	5.725955	9.328033	7.549269	8.28029	11.3318	4.828464	6.744104	2.259254	0.852787
Cs9g04200	4.627451	2.606695	2.264887	2.223279	5.71079	3.462606	2.355802	1.616379	1.485348	0.56695
Cs9g04210	6196.582	7833.403	5622.197	6271.616	3648.253	3253.178	769.1135	3081.856	2350.033	6856.826
Cs9g04250	17.06596	17.01813	13.69823	12.65463	5.08419	4.500804	2.932424	5.804573	0.821144	1.603428
Cs9g04290	1.605744	2.02043	3.378203	2.897161	3.464537	2.652654	5.217873	4.30551	3.58773	4.850529
Cs9g04440	432.6292	429.3449	434.3658	474.1862	356.0663	331.3848	544.0564	808.4084	270.174	738.838
Cs9g04500	0	0	0.026817	0.01342	0.221144	0.070751	5.743633	5.201607	0	0.127526
Cs9g04520	3.286535	6.264564	9.132674	9.568639	0.765444	1.778767	1.401264	6.577899	1.121215	4.604602
Cs9g04570	0.648191	0.713374	1.337601	1.157144	1.670757	1.728348	1.360558	1.489363	1.239754	2.371454
Cs9g04610	0.597732	0.353987	1.128612	1.032691	0.863091	0.896304	0.256065	1.023493	0.086899	0.284328
Cs9g04630	3.449659	3.571702	3.637154	4.44104	5.963131	5.299885	8.332475	9.150829	0.344358	0.819745
Cs9g04690	0.281111	0.381708	0.212941	0.250385	0.675846	0.665073	1.391813	1.697631	4.041992	6.668234
Cs9g04780	16.57731	15.42415	11.06045	13.86401	7.8232	8.329885	9.219203	9.063883	2.525121	4.462607
Cs9g04890	3.571498	3.119018	11.14511	10.80513	17.84622	24.25628	23.74145	20.61531	53.54019	47.05519
Cs9g05010	2.261235	1.074109	1.539366	1.63378	1.087746	2.445157	1.261847	3.407686	1.511919	2.281294
Cs9g05030	228.9654	191.8327	327.9833	303.6342	445.001	380.9643	1146.149	863.7195	394.9856	426.2774

Cs9g05120	1.083316	0.725593	3.087629	2.13328	2.761247	3.118646	1.125374	2.78648	0.096244	0.197025
Cs9g05150	8.477027	16.00581	4.081865	4.480323	2.714382	2.349839	2.059326	3.354237	0.933602	1.32108
Cs9g05220	0.355719	0.470832	0.20776	0.103473	0.729841	1.287793	0.834352	3.243543	0.313117	2.681173
Cs9g05280	2.955492	2.900006	1.67926	4.112267	0.190778	1.023687	0.040185	0.206227	0.051017	0
Cs9g05310	33.53663	47.61175	28.55703	40.15575	18.73631	20.67733	16.09865	22.4889	15.37003	43.46492
Cs9g05320	243.3255	304.929	388.212	334.2176	163.7135	135.103	155.6732	234.2042	93.60947	149.5061
Cs9g05370	10.43577	17.12815	2.591409	5.291187	0.869472	0.874812	1.3443	2.513183	2.375581	5.104737
Cs9g05620	2.832678	3.256838	1.828648	2.04746	0.810548	0.952064	0.400459	2.608139	0.733247	1.138882
Cs9g05630	0.159476	0.208738	0.524409	0.73816	0.320039	0.492203	1.742924	2.437519	0.195489	1.514537
Cs9g05660	3.082866	3.451997	2.293362	3.245703	1.009739	1.739527	0.466526	1.53938	0.515414	1.096377
Cs9g05680	111.41	112.0571	46.81079	85.39758	3.226957	4.448312	4.7938	6.66238	1.908199	1.465054
Cs9g05750	34.61856	45.12194	24.83321	33.88835	12.89691	14.77988	12.88473	25.49392	28.94063	67.52765
Cs9g05800	0.248814	0.395143	0.207617	0.271347	0.196516	0.397121	0.13496	0.450925	1.267178	3.289281
Cs9g05820	1.331523	1.927297	1.934104	2.529178	3.39396	4.17885	4.062825	11.37268	16.88563	23.75933
Cs9g05840	33.3145	39.22943	21.17069	25.78788	6.735514	11.69506	2.891205	30.52528	7.448836	39.57037
Cs9g05910	2.931757	2.401857	12.23836	9.47354	8.201035	9.571102	5.33986	3.992488	0.036141	0.125509
Cs9g05940	2.575165	2.646009	5.206807	4.213683	4.959766	5.954056	5.393322	5.928209	1.283973	3.222054
Cs9g05950	1.660373	1.351479	2.463178	1.942321	2.81055	2.344115	3.647138	1.696003	1.899218	1.977475
Cs9g06010	0.092288	0.077883	0.050938	0.013775	0.068198	0	2.77822	0.091848	0.128554	0.594986
Cs9g06060	2.899291	1.951144	0.742249	0.796233	0.427451	1.468048	0.848191	1.674712	0.527938	2.348644
Cs9g06150	4.45524	2.747314	1.342607	1.162114	1.264516	2.93694	0.311026	0.587996	0.536635	0.863172
Cs9g06290	24.90444	31.39613	28.59604	28.23845	13.31784	15.94859	24.16022	38.76343	19.42599	47.10601
Cs9g06310	25.20253	49.18784	4.969866	11.32887	1.875151	3.4816	2.764709	6.956209	0.590989	2.550462
Cs9g06360	3.843843	6.605899	1.169353	1.249575	0.482222	0.346245	1.870643	1.229205	0.408055	0.18666
Cs9g06450	4.04657	2.431943	12.34676	8.632577	17.98771	21.95157	45.55683	31.92677	30.18906	38.26669
Cs9g06480	6.373203	6.644217	7.730899	7.324466	12.38861	12.97458	14.5938	15.10144	12.29209	11.39542
Cs9g06490	0	0.040408	0.031253	0.025593	0.036223	0.168454	1.605524	1.040359	45.70363	84.50574
Cs9g06500	0.464657	0.497376	1.365339	0.873401	7.357134	9.46079	16.5369	23.7212	19.49975	19.87842
Cs9g06520	0.494692	0.206694	0.33498	0.311208	0.364444	0.863478	0.30637	0.391053	0.394488	0.793768
Cs9g06530	1.814825	0.811992	0.143038	0.140779	0.016659	0.016397	0.057554	0	0	0
Cs9g06540	28.76773	21.51563	12.77636	10.09048	26.50694	13.68283	37.01362	14.39129	45.79263	25.55286
Cs9g06620	95.45237	71.05255	100.1932	95.56738	180.6506	133.5989	99.32578	57.42189	181.0761	47.63913

Cs9g06630	48.14809	35.18184	36.20868	34.74582	30.93712	32.88087	27.40769	16.91716	89.67677	31.44637
Cs9g06660	123.9009	183.9374	43.27192	61.2149	11.17438	10.54237	7.087597	18.14308	1.871373	3.436378
Cs9g06670	110.2276	135.2615	85.69281	102.5141	11.87091	13.66014	27.52563	39.10711	25.81661	109.291
Cs9g06700	6.958073	21.83263	17.00185	16.83645	7.597208	1.658329	0.208394	1.411344	0.165597	0.152048
Cs9g06710	6.874051	23.07563	18.7367	22.82866	7.91266	2.009545	0.150982	2.196831	0.208079	0.425676
Cs9g06740	0	0	0	0	0	0.005404	0.018847	0.016597	0.449045	1.128529
Cs9g06800	3.342525	4.762778	5.12141	5.927193	7.675664	6.422306	18.11236	20.54527	7.681275	8.097218
Cs9g06846	5.152658	5.581459	7.676411	6.672114	9.463968	10.96178	3.789266	10.28095	5.213377	4.336736
Cs9g06860	27.47504	23.62177	7.208142	7.878162	7.352185	5.049953	5.963315	6.090297	0.95292	0.61865
Cs9g06870	93.18698	92.34735	41.71252	12.84989	150.3988	35.03872	117.0217	78.38408	31.8267	33.57062
Cs9g07050	0.486769	0.254873	1.382585	1.283829	1.105167	2.341447	1.17975	1.232003	1.568826	2.489829
Cs9g07140	50.55961	68.60163	53.07605	46.06748	9.336909	17.28928	1.927601	18.51224	1.895536	3.631868
Cs9g07160	4.746279	6.816295	12.51199	10.48093	4.610751	6.686054	5.63168	10.52148	0.614781	1.970881
Cs9g07180	2.907213	4.862537	7.301026	7.425754	1.961877	2.337121	2.207703	5.599489	0.86311	1.835098
Cs9g07200	4.070801	6.064117	11.90029	10.78781	2.5139	3.644165	2.752104	7.450002	1.922935	2.749148
Cs9g07230	10.19108	18.39	24.00229	31.6401	2.860757	10.61168	4.092977	8.952963	1.197347	5.549825
Cs9g07445	5.361147	2.921675	3.74405	1.698435	2.526426	0.819998	3.187171	1.043015	0.249466	0
Cs9g07540	7.115424	10.16031	4.753667	3.605822	2.888797	2.163174	0.931548	1.141664	0.149398	0.168004
Cs9g07560	9.305595	11.26282	30.48032	35.78712	8.636922	17.78605	7.596656	11.07139	4.405234	11.60688
Cs9g07590	1.077074	1.421821	2.294237	3.674587	5.371291	5.548841	1.47192	4.86622	0.428956	0.688887
Cs9g07620	2.848224	3.666962	3.858357	4.351937	2.654279	3.004695	1.623823	3.324195	2.496149	4.365082
Cs9g07670	0.843198	4.033569	0.956817	1.180192	16.89974	3.604248	438.7687	98.35271	0.690572	0.765809
Cs9g07680	132.2148	362.2739	40.77264	127.0893	405.3699	126.7801	1242.811	1027.142	18.40252	7.254812
Cs9g07740	0.936452	0.938393	1.264659	1.14577	0.870156	0.642998	2.425921	1.707349	0.698181	0.60968
Cs9g07790	11.64145	5.230451	1.050097	1.405868	0.160964	0.849122	0.897268	1.550064	17.56661	18.4997
Cs9g07810	7.792254	8.762447	13.24321	17.76178	5.191314	12.74212	5.315769	24.48348	11.78447	19.56561
Cs9g07830	73.55001	65.34801	63.25593	58.41046	41.46127	18.86926	77.59267	27.08377	16.08267	14.11655
Cs9g07860	3.829771	3.356712	6.12583	7.633844	4.609817	4.714003	4.470155	6.99722	1.992431	6.054328
Cs9g07890	88.50644	138.753	61.28258	65.0781	29.20051	27.90851	14.03081	13.51572	14.38597	7.737179
Cs9g07970	46.23168	45.76067	73.28531	45.4049	36.38368	39.67219	29.16365	88.5486	0.606567	5.580151
Cs9g07980	3.025453	2.261325	5.341288	5.788007	1.69554	3.870766	1.316572	2.819965	2.828942	3.653375
Cs9g08000	14.54007	16.26167	41.13961	37.98908	44.96631	47.98534	73.87451	68.3402	48.42273	109.7938

Cs9g08020	25.07973	26.68166	16.6097	20.04231	9.283623	8.3884	8.822831	14.76716	5.989648	6.958278
Cs9g08030	40.02193	47.12162	13.45937	22.70927	1.276694	1.142967	0.348613	1.664913	0.417166	0.628277
Cs9g08070	40.90507	43.57028	40.5561	43.20588	12.03754	17.29678	7.419399	8.331789	7.204851	7.161106
Cs9g08100	56.66736	58.69993	79.54918	87.90177	36.01871	71.72931	30.45192	94.50801	13.64274	11.95852
Cs9g08250	2.493334	1.426215	1.056532	1.227332	1.176892	2.876682	0.770529	1.476896	1.273888	1.443604
Cs9g08260	3.25419	1.936626	0.970462	2.172018	1.434493	3.578045	0.666309	2.218023	1.62894	3.662926
Cs9g08340	1.308484	0.813042	0.38326	0.611191	0.585927	1.115185	0.366778	0.990812	0.632336	1.315323
Cs9g08380	2.018989	1.175839	8.491134	3.296964	8.711203	10.17925	0.198461	0.323728	0.047912	0.091763
Cs9g08420	2.159439	2.067711	1.766461	2.22741	1.9853	2.457012	0.538778	2.629857	1.047601	2.247668
Cs9g08440	1.221255	1.35658	1.525364	1.53275	1.602022	1.973982	0.57938	2.462281	1.326318	2.545753
Cs9g08460	8.769222	12.9764	24.67816	39.25507	3.377492	19.16847	9.733073	32.54923	112.5259	318.7857
Cs9g08480	10.33308	13.49881	10.17475	10.34934	9.149047	8.058389	9.30038	8.182002	2.74836	7.260988
Cs9g08490	11.89839	15.89935	15.35228	20.35487	7.676988	9.857489	9.173131	14.95329	10.97132	25.9416
Cs9g08500	10.81506	22.26108	4.492635	8.713246	5.216486	6.543401	5.056689	8.77025	5.88087	4.542476
Cs9g08530	16.76511	18.33487	7.898311	8.149706	3.131074	4.280991	1.792976	2.614594	0.47334	0.400871
Cs9g08590	32.80763	38.04413	33.29001	31.50305	18.22072	12.58305	10.39878	18.79419	3.235952	6.855486
Cs9g08610	0.420667	0.317644	0.531974	0.676239	0.544103	0.471247	0.447141	0.283049	0.802807	0.248727
Cs9g08700	0.18795	0.608702	0.79494	1.360231	0.200553	0.40242	0.435626	1.68639	0.184512	0.364554
Cs9g08770	1.680279	0.792082	1.783818	1.278208	1.964736	0.799089	0.579216	0.039054	0.02094	0.007769
Cs9g08870	5.450795	5.948828	5.616102	6.117811	7.071396	7.20368	4.384062	9.234747	8.29248	6.379452
Cs9g08990	4.285244	4.041419	12.83404	10.18548	7.150232	8.956091	2.647024	6.934959	2.185553	1.908096
Cs9g09060	0.613434	0.654262	0.603727	0.784771	0.973067	0.997506	0.379851	0.859113	0.525659	0.465696
Cs9g09080	63.38071	53.87635	244.655	145.7392	285.3735	199.157	138.6211	204.5441	14.49134	19.18427
Cs9g09090	0.41615	0.22502	1.681303	0.69696	1.587661	1.086274	0.415562	0.540026	0.200415	0.082602
Cs9g09100	3.543975	5.045405	3.931101	4.360022	2.848295	3.203641	1.225152	5.300062	0.436532	1.009573
Cs9g09120	1.53263	1.574811	3.729025	4.847856	2.209488	3.018444	0.843172	1.889307	0.146901	0.120366
Cs9g09280	11.69441	11.12805	9.887121	9.572779	6.269237	10.57512	2.801274	6.641951	5.517081	2.627889
Cs9g09290	0.769019	1.183548	2.772454	4.037741	18.29722	12.99877	24.63982	32.46589	15.32764	23.02271
Cs9g09320	3.736698	4.400792	8.446308	7.146745	11.34513	14.07346	15.75064	26.56743	16.78353	15.58478
Cs9g09370	0.583318	1.236345	0.787452	0.813297	1.204804	0.882839	1.200483	2.243014	0.252227	0.260423
Cs9g09430	0.256267	0.197535	0.203008	0.185304	0.321578	0.566024	0.496724	2.20684	0.573924	0.619225
Cs9g09450	8.019621	32.00927	0.389099	5.496968	4.797739	11.00294	10.94962	18.31924	10.46545	6.849507

Cs9g09475	4.134636	4.608194	6.066341	6.103851	5.94862	6.879448	2.248163	8.019103	4.834147	5.622699
Cs9g09530	27.07618	26.93303	17.62283	24.21264	5.741075	7.955853	2.36034	3.684673	0.692029	0.348413
Cs9g09540	0.46559	0.194719	0.413469	0.469937	0.519922	0.593177	1.814237	0.666183	0.489243	0.468592
Cs9g09570	11.1782	10.74727	10.21725	12.35849	3.641393	4.605749	1.050159	2.417179	1.630965	1.02907
Cs9g09640	8.012044	3.975169	6.183116	5.562083	15.37845	26.53224	27.90352	14.63259	29.51964	15.52663
Cs9g09660	32.36088	23.9339	47.26319	41.42859	71.78785	73.37292	145.8945	65.83896	90.39694	95.22683
Cs9g09690	0.283802	0.459385	0.692963	1.02607	0.475455	0.699956	0.091155	1.043895	0.053376	0.573692
Cs9g09710	0.795747	0.434466	0.671172	0.816346	0.093752	0.487887	0.14019	4.997235	0.009784	0.064199
Cs9g09740	11.67324	9.682991	58.57677	79.58502	30.73069	117.4079	20.41252	29.19933	37.12191	76.52049
Cs9g09750	27.06989	19.87329	34.51641	25.36382	113.0455	101.6767	154.4494	90.96977	4.946105	2.650155
Cs9g09790	4.56844	5.09999	4.112565	2.766504	2.925285	2.59659	3.306318	2.426751	0.304301	0.938261
Cs9g09800	6.562501	8.108381	6.873228	5.538323	5.772365	5.080249	6.895714	7.157139	0.721197	1.73508
Cs9g09810	2.065993	2.836103	2.076101	1.276831	3.952475	1.973422	2.820503	3.29637	0.012461	0.46906
Cs9g09990	0.374549	0.431174	1.239015	1.173855	1.16398	1.55681	0.473976	1.8069	0.318027	0.114657
Cs9g10150	0.551924	0.553102	1.041711	1.086083	4.815874	4.101903	2.464495	3.279128	3.854577	5.717404
Cs9g10360	1.02563	0.868351	0.996228	0.882243	1.283029	1.48368	1.429541	0.991263	3.094519	1.240457
Cs9g10400	63.15033	58.72877	116.2248	106.5424	127.3416	149.7684	56.29415	163.7599	46.89887	81.47803
Cs9g10410	47.41033	41.63011	92.90793	88.06008	88.77674	124.2571	39.27655	128.8935	34.38367	72.15833
Cs9g10430	5.220742	5.102682	25.93536	22.97121	27.43604	26.23833	26.08421	68.90719	15.37446	22.63927
Cs9g10440	11.49893	10.44294	48.80744	40.43233	130.8587	118.7395	82.94498	126.9237	40.57211	47.9582
Cs9g10480	11.32871	14.16374	36.48482	45.58773	5.180471	16.91864	12.74044	41.40738	7.244701	19.92418
Cs9g10520	22.25701	22.39348	20.29694	15.95564	47.02553	33.85682	24.39004	14.90091	0.846109	0.602367
Cs9g10530	22.10456	10.77199	29.88665	22.82186	49.4838	57.08998	21.06497	5.417441	0.560606	0.174041
Cs9g10630	220.8486	160.8126	67.7624	73.04519	41.9547	48.25737	235.9153	194.314	21.62344	20.54905
Cs9g10650	2.809349	3.18235	1.601156	3.575163	1.095029	2.121268	1.103919	1.183147	0.151334	0.322836
Cs9g10700	2.979984	4.222917	3.923948	5.034859	1.714602	4.507348	1.056932	2.813314	0.758616	0.338287
Cs9g10740	5.970555	5.356616	18.44985	15.351	14.24628	10.79428	5.825267	11.10786	0.930217	2.627168
Cs9g10780	0.292399	0.243007	1.370977	0.926559	0.65787	0.893222	0.30567	0.878005	0.020028	0.208146
Cs9g10830	103.6298	73.91108	166.6821	134.1931	161.918	199.5473	49.80784	59.85845	40.25264	5.292573
Cs9g10850	0.338012	0.136304	1.190718	1.007775	1.343483	1.256831	1.399961	1.166359	0.740596	0.938615
Cs9g10925	0.225408	0.375466	1.39825	0.950306	2.357619	3.187206	0.46506	1.73502	0.420683	0.254987
Cs9g10940	50.28293	47.22266	115.273	78.85343	135.4215	161.5305	52.84342	152.8755	27.25649	21.32594

Cs9g10950	28.93795	29.32802	48.47034	40.10012	48.9344	73.11681	26.60061	97.24326	14.55009	18.88491
Cs9g10960	0.955038	0.880185	1.267713	2.179164	2.792325	3.005991	2.186969	3.767584	0.894334	1.398367
Cs9g11050	17.06025	18.65432	19.16497	23.13616	13.71015	15.43278	14.08052	10.00901	7.682979	23.08628
Cs9g11190	0.242527	0.213775	0.295566	0.312058	0.495111	0.358104	0.913369	0.271256	0.168015	0.15701
Cs9g11200	4.757571	3.547628	8.454698	7.300365	12.78315	13.28386	14.41763	12.69364	18.00734	20.56952
Cs9g11390	2.999182	3.246404	2.521319	2.130534	1.798784	1.431758	0.357923	2.298871	0.101938	1.110527
Cs9g11480	287.1528	235.8894	292.6358	266.4051	205.6935	236.0619	74.94738	85.38567	29.60698	3.408989
Cs9g11510	3.038845	3.757148	3.315361	3.606057	2.249569	3.149486	1.701585	3.164614	1.62484	3.908162
Cs9g11610	19.23092	23.52543	33.55894	29.20109	33.48922	35.30621	19.01464	44.30659	12.57058	15.54455
Cs9g11690	28.15002	28.01507	33.33785	32.63824	15.32834	19.67879	9.592151	23.62685	2.936921	4.48871
Cs9g11730	0.175294	0.083463	0.937555	0.719536	0.8108	0.980742	1.231395	0.913678	0.454479	0.521813
Cs9g11750	29.95965	22.1561	28.68995	16.66003	29.08112	19.98781	9.269847	18.54673	0.319915	0.689796
Cs9g12040	2.0806	1.688864	7.989238	9.594438	3.731789	5.446789	0.292491	5.645124	0.910313	1.29894
Cs9g12070	0.517449	0.302736	1.848336	3.398699	0.086116	1.207623	0.145361	1.985402	0.17179	0.472736
Cs9g12160	7.923689	6.064831	9.857676	14.11971	2.272403	4.910017	0.314554	4.551341	0.326318	0.481797
Cs9g12180	1.829982	1.104647	1.905774	2.453123	0.722033	1.637394	0.212183	0.666393	0.322534	0.368416
Cs9g12190	1.270209	1.365365	1.464064	1.56449	1.016365	1.346489	0.540422	1.556141	0.989895	1.574182
Cs9g12200	0.70392	0.374983	0.954351	0.968973	1.39103	1.529244	1.489859	1.118825	0.655348	0.606867
Cs9g12220	31.59927	24.98862	14.22537	18.13215	3.505866	7.356898	0.556286	3.611096	0.254184	0.208496
Cs9g12240	35.92309	25.71531	37.4067	45.20825	11.44748	17.54642	5.113676	15.48846	16.36138	20.73754
Cs9g12253	0.973291	1.118048	0.928815	0.841619	0.728313	1.185445	0.334178	1.010135	0.670694	0.578314
Cs9g12256	4.27056	4.768597	5.939643	4.30028	5.358462	5.32055	2.343286	8.080959	3.910381	3.155101
Cs9g12285	10.33647	8.074567	7.829067	9.03709	6.108202	7.378532	2.390331	5.852321	7.248626	5.96918
Cs9g12370	68.24414	76.10448	50.19585	53.70827	44.15184	15.76507	28.41302	13.09745	4.123659	6.304407
Cs9g12400	0.604409	0.520922	0.925886	0.742361	1.170142	1.153336	2.079296	0.810691	0.980384	0.894191
Cs9g12450	31.4106	33.19147	36.33645	41.41083	15.12087	20.49812	10.78765	15.10177	5.659398	13.77274
Cs9g12460	166.0311	251.3378	89.30076	101.2859	55.23889	61.14194	35.12018	29.07054	24.29839	29.09495
Cs9g12710	6.007174	10.98895	11.49093	14.48057	1.908339	6.015903	7.392263	22.90084	2.476306	22.87204
Cs9g12780	12.75544	6.754385	10.75176	15.40589	8.602864	19.77303	29.65056	15.67264	122.282	98.74582
Cs9g12800	0.651009	0.67456	0.614603	0.871975	0.377587	1.265285	0.288391	0.53273	0.046743	0.155206
Cs9g12830	2.089673	2.968459	1.327043	1.2627	0.743887	1.168804	0.398689	0.833637	0.180116	0.031417
Cs9g12900	0.844371	1.44281	1.401672	1.300209	0.705643	1.124563	0.472339	0.902241	0.122123	0.172541

Cs9g12910	0.744403	1.402662	0.700122	1.029774	0.428219	0.985226	0.2796	0.767119	0.120438	0.070899
Cs9g13010	1.247873	0.592661	1.615348	1.180739	2.01985	2.068669	1.649706	1.546417	0.31086	0.1999
Cs9g13050	32.72978	23.17122	14.8493	18.04721	2.746672	4.508571	2.331364	3.996144	2.202015	1.567939
Cs9g13080	0.850991	1.223306	4.826436	3.871135	6.831613	7.325743	5.186017	8.879565	4.540457	8.824439
Cs9g13170	6.979783	8.4328	10.29324	9.698983	12.76787	14.64616	5.326611	13.81618	8.630982	8.279498
Cs9g13280	0.26971	0.122404	0.699328	0.463624	0.624057	0.675189	0.702292	1.435571	2.218513	2.097673
Cs9g13330	5.191509	6.18073	4.447224	4.970505	1.21316	3.080019	0.543785	3.290297	0.100941	0.518979
Cs9g13460	14.6663	61.839	1.167444	5.599656	5.377466	3.225364	9.082382	9.402199	1.60126	1.615259
Cs9g13470	10.71123	10.61815	6.22163	6.31492	4.434742	4.768928	6.015779	7.574588	6.658833	2.427927
Cs9g13540	0.522165	1.27043	0.789386	1.025946	0.472578	0.912261	0.333675	0.569117	0.036297	0.071374
Cs9g13580	6.654746	11.34321	19.98028	18.32491	43.08556	34.12507	13.42799	21.06853	0.675845	0.992646
Cs9g13610	73.92856	161.7077	182.689	233.6766	30.32469	39.67846	42.80918	122.985	2.247491	8.108215
Cs9g13620	81.62104	163.3403	295.9175	336.6042	70.87872	105.4673	95.28995	239.3204	115.2926	125.419
Cs9g13630	7.659641	8.769755	29.58225	18.95454	126.0253	85.81443	25.90398	19.17892	3.724467	2.174884
Cs9g13710	0.552846	0.650594	0.566323	0.688473	1.03974	1.353384	0.748634	1.660586	1.161598	0.536017
Cs9g13800	2.854374	1.689391	3.155685	2.181759	3.648154	3.558527	8.189517	4.452826	8.718313	2.614385
Cs9g13810	0.034836	0.077908	0.157975	0.179208	0.52497	0.632492	0.314027	0.962816	2.741611	3.806
Cs9g13980	5.753149	6.476785	8.545378	6.725915	4.951006	11.25842	8.112313	5.409143	19.2718	6.145998
Cs9g13990	9.396625	10.3977	7.413872	6.659874	7.520301	12.88816	6.380954	15.25341	15.0775	12.0301
Cs9g14040	51.53568	51.38407	29.90663	32.65925	11.79664	24.93695	8.596889	17.03534	2.802449	1.774863
Cs9g14100	0.390787	0.21388	0.16954	0.057678	0.503258	0.310614	0.570738	0.638158	0.981928	6.529971
Cs9g14120	46.09687	46.52812	32.74791	43.33355	15.87155	19.96263	40.66662	75.41624	112.4169	231.6264
Cs9g14130	5.17639	4.590586	4.994849	5.940458	2.352311	3.052933	2.452596	10.17853	7.533684	25.76733
Cs9g14140	0.341448	0.790229	0.521251	0.524556	0.513615	0.809785	1.516279	6.873786	12.58539	24.91816
Cs9g14150	13.9721	18.01142	14.18005	17.39658	5.029567	9.352255	14.86028	72.84848	223.1126	457.2149
Cs9g14210	10.0003	15.60835	5.362126	8.644985	4.77784	5.81213	3.231214	6.168699	3.609397	4.953843
Cs9g14230	5.373823	5.477294	4.342321	4.4311	1.46909	2.338725	0.67992	1.426708	0.675777	0.729944
Cs9g14250	16.87369	17.49073	29.27567	27.01148	21.25057	32.10338	10.56386	25.09063	25.77595	32.19146
Cs9g14260	1.643631	1.236918	3.38511	2.343271	3.61093	3.125215	5.605692	3.659583	3.139194	3.060488
Cs9g14300	0.814456	1.038005	0.462364	0.670717	0.358246	0.552716	0.209643	0.864653	0.142111	0.813191
Cs9g14320	496.0185	389.0895	416.8338	473.0539	298.0197	535.851	362.8538	282.0714	159.8147	65.30009
Cs9g14330	14.49663	13.70759	17.56794	19.29983	12.20165	15.78684	7.305317	12.26724	12.55283	4.643507

Cs9g14360	10.3402	6.649114	2.464659	2.77018	0.536155	9.921139	0.965392	1.103948	0.258503	0.199592
Cs9g14370	11.76573	9.642049	10.38158	11.60277	7.851546	23.83704	3.826529	10.14568	6.720925	5.576006
Cs9g14380	9.540658	6.429487	2.790096	3.374846	0.677263	6.25961	0.125244	0.63388	0.143525	0.095444
Cs9g14453	3.911004	3.334484	5.73599	4.561902	4.643643	6.739719	2.380585	5.356435	3.442567	4.886595
Cs9g14480	2.735762	3.514133	51.21086	10.94838	42.42166	26.30393	11.85015	12.4336	32.5905	46.07765
Cs9g14490	2.310754	3.442451	5.52703	7.08699	0.677744	2.164845	0.902645	3.583783	1.090035	2.415833
Cs9g14510	3.619313	4.800008	8.612135	9.833743	0.815092	2.922868	0.691984	3.953379	0.26963	1.255473
Cs9g14520	0.576826	0.696814	16.98404	3.374298	15.00668	6.834483	3.796009	4.64413	8.895594	8.078525
Cs9g14590	14.00452	24.4718	10.56687	16.26415	1.509105	1.808059	1.766233	1.235501	1.406645	2.019834
Cs9g14600	10.17541	10.32642	5.608132	9.502753	0.810416	1.91816	1.755416	3.044946	3.536493	12.42009
Cs9g14660	39.16633	88.61521	53.16608	78.13206	28.96573	87.80361	38.77499	69.34549	8.752119	7.793997
Cs9g14690	3.764446	3.810249	8.006244	4.806602	18.75789	13.85441	31.25187	29.80656	14.04046	13.8687
Cs9g14710	12.92197	15.05291	25.46336	28.90495	35.89565	36.35785	31.65299	53.79044	38.52675	75.92078
Cs9g14750	2.018508	2.261805	3.516585	3.134518	2.992809	3.166825	2.947849	4.793036	1.393811	3.962467
Cs9g14840	1.204421	2.438712	0.033532	0.109569	0.063728	0.067241	0	0.044777	0.023411	0
Cs9g14880	0.75875	1.339426	1.131868	1.288146	0.770853	0.951241	1.058243	2.848517	0.469806	0.672603
Cs9g14930	21.49164	23.13088	21.62843	20.0578	15.11917	14.85679	9.444191	9.828402	6.635577	7.312377
Cs9g14960	92.86453	73.55396	58.13215	59.80172	1.562644	9.264107	0.821459	5.891674	0.141001	0.039591
Cs9g15020	11.06481	13.38167	4.80052	6.708168	4.344662	4.019539	5.00745	3.325639	2.402329	2.285125
Cs9g15170	1.491332	1.808622	2.887612	2.614044	4.873571	3.337609	3.232031	4.806306	0.223201	0.246937
Cs9g15180	1.232333	1.110971	1.637191	1.451499	1.809735	2.644641	1.042478	2.818865	0.076815	0.175313
Cs9g15290	4.798313	5.605063	15.30368	11.6963	23.75555	21.71263	31.2393	27.74324	6.046376	15.92286
Cs9g15350	0.320322	0.313956	0.956656	1.462232	1.274123	1.084665	0.500394	2.529311	0.311149	0.214129
Cs9g15430	602.5333	1176.09	114.9476	247.8117	188.5894	243.8097	312.8114	642.041	143.6121	415.6831
Cs9g15560	3.338216	2.832473	3.963978	4.853711	3.615407	5.365917	1.023639	5.953504	3.054486	6.071786
Cs9g15565	1.796377	1.549882	2.055266	2.056017	1.699253	2.290557	0.781494	2.89846	1.158415	1.925309
Cs9g15580	8.665106	9.411502	14.70452	17.98961	3.654786	9.131886	2.278476	8.205672	10.51425	9.769931
Cs9g15590	4.104751	4.748766	2.424083	3.619624	0.700288	0.659391	0.316167	1.694685	0.254216	0.318255
Cs9g15620	1.616168	1.951952	7.669077	7.36648	7.355099	6.2585	4.291252	9.425283	8.375068	12.72841
Cs9g15630	0.184572	0.121556	0.397544	0.726998	0.135302	0.184874	0.166149	1.177308	0.073584	0.360018
Cs9g15695	1.015626	1.61259	1.226674	1.141263	1.914786	1.818361	1.036305	2.742099	0.316915	1.002603
Cs9g15720	0.505479	0.697197	1.10947	0.902954	0.563202	1.258506	0.588248	0.881338	1.18956	1.777663

Cs9g15730	0.804012	0.792692	2.062192	2.042443	1.561925	1.65273	1.722623	3.607219	1.170117	1.863582
Cs9g15800	12.20862	9.386445	7.771323	4.986728	33.66384	28.91711	28.67594	14.55649	0.783518	2.616255
Cs9g15830	10.85892	9.63808	22.15042	18.40682	37.95969	37.93205	11.2076	4.080831	0.386367	0.249522
Cs9g15860	5.513881	4.507514	3.845801	3.333272	25.43158	12.28369	50.65672	14.59484	20.85724	14.25644
Cs9g16020	20.71281	20.01254	20.21645	12.73039	10.53386	16.21828	26.25646	24.69465	12.79838	18.01274
Cs9g16030	20.62319	19.85166	28.62902	29.1768	44.44939	41.70092	57.71558	79.085	49.78233	51.6871
Cs9g16170	299.3458	314.2661	439.0719	398.136	699.7622	281.7407	874.3535	442.5486	390.053	502.6455
Cs9g16220	0.066395	0	0.286775	0.369587	0.479152	1.077794	0.226852	1.295213	0.187958	0.195647
Cs9g16230	4.708556	2.230977	2.23844	1.832155	0.888286	1.387981	1.317096	3.967039	0	0
Cs9g16240	2.48091	1.245654	1.173104	0.866444	0.424492	0.628269	0.645486	1.831833	0.029819	0
Cs9g16360	34.26252	22.29019	17.22358	18.41046	16.61858	6.448187	11.62358	6.38512	14.64553	12.65421
Cs9g16380	9.473538	6.951374	8.635549	6.97746	12.28773	11.04725	18.96715	7.780178	28.21898	5.897794
Cs9g16450	33.57071	20.15209	30.27212	27.19746	26.76696	22.61148	13.47016	10.26707	3.631838	0.745658
Cs9g16460	1.679938	0.689493	0.503015	0.528111	0.261284	0.533637	0.186346	0.211482	0.042619	0.023458
Cs9g16480	17.76103	24.95477	16.78868	17.87569	17.81644	16.18499	23.93208	26.37188	2.461279	6.893104
Cs9g16490	3.426878	4.207841	4.406756	0.963365	3.362266	3.171537	1.580856	1.815218	0.120818	0
Cs9g16510	0.38733	0.291288	0.603667	0.59988	0.4814	1.198848	0.378166	0.470289	0.091518	0.09303
Cs9g16560	4.24193	3.554594	4.87345	5.711527	5.13631	10.32874	1.976752	5.449661	4.698611	7.074571
Cs9g16610	71.1605	64.17663	87.76661	74.63903	81.9423	85.87765	81.79727	105.8569	12.28174	29.07905
Cs9g16640	0.203402	0.773194	0.438317	0.534224	19.50895	4.807313	99.06162	63.18509	288.4104	112.3404
Cs9g16670	131.8557	173.1553	95.93766	118.4722	47.80724	49.70968	25.17151	54.62434	39.78285	75.55184
Cs9g16680	83.58279	104.3448	64.35881	69.45808	31.67392	31.85145	15.75467	46.69927	17.24616	38.71772
Cs9g16690	43.47263	55.17994	20.28796	27.56397	8.543987	7.766345	5.332219	7.9886	5.330111	9.461592
Cs9g16750	3.720848	2.815818	3.805836	3.295403	4.61362	6.008123	7.501165	6.830146	3.535758	3.27005
Cs9g16770	9.349343	16.35489	5.130861	13.87273	0.687784	1.340294	0.278708	1.673492	0.109011	0.228996
Cs9g16810	64.8144	86.58283	30.68322	39.78009	2.054623	6.510067	37.80178	92.43148	38.23596	275.6634
Cs9g16820	100.5614	96.17058	53.9907	69.9767	6.354775	25.11551	87.10103	124.1819	179.5904	429.3948
Cs9g16830	8.469962	7.519197	4.707829	6.46249	1.733886	3.235878	2.396225	4.523948	2.852727	6.477919
Cs9g16870	5.941081	0.864178	2.003307	1.790672	0.869404	2.225281	1.372944	0.612632	9.695538	2.658377
Cs9g16880	36.30606	37.5758	29.7316	22.09602	10.54205	16.81559	5.615981	13.31733	6.001163	3.928321
Cs9g16890	15.36942	16.34717	5.373895	6.460699	1.547589	2.202732	0.845842	2.047196	3.378201	3.534976
Cs9g16920	9.169722	12.43199	10.75414	13.74501	7.115834	8.179526	2.203661	4.326747	3.543651	2.716326

Cs9g16940	1.535975	1.083974	0.43051	1.33521	0.068203	0.32408	0.167273	0.804699	1.074467	1.332311
Cs9g16960	115.8811	99.60861	75.57766	69.12194	57.90812	37.91073	17.7668	8.985436	1.652231	1.836647
Cs9g17050	0.050744	0.070761	0.558244	0.064446	10.62526	11.48196	5.604958	4.706191	6.113524	2.218758
Cs9g17100	0.771682	0.633026	0.813803	0.905577	0.9987	1.506511	0.664953	1.436104	1.541297	1.57298
Cs9g17160	0.449103	1.374086	0.052922	0.198728	0.064974	0.054132	0.073762	0.040125	0.097306	0.019869
Cs9g17210	142.2901	173.3715	144.2679	137.4016	78.36459	59.67263	53.12577	121.8039	28.42964	85.94009
Cs9g17220	216.897	219.3391	201.1087	196.2298	196.5022	101.9896	103.0595	187.5102	53.54255	98.43761
Cs9g17230	3.260164	4.570188	0.789145	0.566962	0.242108	0.468684	0.102804	0.919906	0.254191	0.247311
Cs9g17240	0.223437	0.129357	0.077521	0.034816	0.070412	0.108468	0.072613	0.010984	1.126163	0.289005
Cs9g17250	237.9387	172.8462	76.84516	67.64053	80.05093	46.23074	52.01672	43.71097	354.8964	457.7288
Cs9g17270	4.685067	7.633986	10.96934	8.650545	23.16691	18.0445	43.44011	75.80986	36.01077	99.34838
Cs9g17330	12.40874	12.25477	7.106024	9.305503	3.370028	6.343025	2.67482	3.787718	0.913566	0.589617
Cs9g17380	21.03301	23.93765	14.47613	12.35829	12.64043	10.24667	197.8904	26.49845	10.11548	42.83755
Cs9g17410	10.90464	10.62758	28.41613	23.47123	37.06589	45.21493	30.68637	39.71405	36.63899	42.68177
Cs9g17440	9.565629	12.25801	15.2358	16.30604	12.70928	11.8658	8.363658	21.47139	17.51171	18.42252
Cs9g17460	0.108271	0.083799	0.105252	0.172059	0.100623	0.253163	2.170147	0.52045	3.920282	0.865075
Cs9g17480	1.501741	2.733598	1.93157	2.050919	2.30134	3.246476	0.693412	5.050438	0.522281	1.140365
Cs9g17540	5.475951	5.756568	6.083493	5.919918	6.014853	6.374641	3.059486	6.962801	5.090761	3.386252
Cs9g17610	2.039992	7.376641	0.717844	1.839524	1.061928	0.639032	1.75364	2.34645	0.847903	0.79336
Cs9g17620	9.670216	21.36307	5.880768	8.994484	7.235208	7.348069	8.626513	11.22228	5.848926	5.350309
Cs9g17640	3.481898	4.16347	3.993201	4.054092	2.289842	2.628035	0.958312	2.024115	0.204733	0.623492
Cs9g17670	0.103557	0.312203	0.531039	0.464821	0.845713	0.590134	2.458987	1.01152	31.72575	53.28928
Cs9g17740	6.899444	6.263143	8.349928	9.377176	13.33046	19.14947	14.55714	19.88034	38.42063	37.20965
Cs9g17790	8.571141	5.751909	14.65158	7.997979	9.34945	7.628981	7.035923	6.708684	6.649933	4.069709
Cs9g17820	0.105324	0.058233	0.263008	0.203013	0.543984	0.356103	0.126244	0.233332	1.447143	0.688815
Cs9g17835	3.184928	3.191841	3.103973	2.715247	0.989628	2.047177	0.23845	2.428403	0.887739	1.263492
Cs9g17860	0.546793	0.473463	0.90865	0.658639	0.614458	1.079184	0.304385	1.538679	0.02985	0.598635
Cs9g17890	8.187622	7.05689	4.039218	4.096321	1.701705	3.311879	1.466244	2.990356	0.245911	0.351665
Cs9g17930	19.67129	18.01395	10.25837	12.59959	4.36424	9.212938	3.766746	7.401279	1.350946	2.332352
Cs9g17940	154.4853	119.2019	182.8561	142.1922	247.4665	201.1445	387.4938	147.1502	237.7912	158.8863
Cs9g17990	60.15627	59.29802	34.2257	31.08274	15.35631	19.27864	15.84009	22.45548	123.3541	47.14622
Cs9g18020	69.04419	63.15037	42.84386	29.33697	56.77001	22.65814	28.15299	37.76677	60.10939	57.81512

Cs9g18040	3.522525	4.186107	5.511163	4.702351	5.811705	5.49868	2.390865	4.083513	0.69647	1.841168
Cs9g18050	6.367086	9.878196	5.794137	6.707379	4.594408	4.530316	6.694667	14.69009	2.629335	7.253473
Cs9g18090	3.827849	5.429898	2.935541	3.420875	3.145035	2.561199	3.343285	7.074702	1.28973	2.107425
Cs9g18110	9.671052	14.57568	9.291961	10.7864	7.206794	7.829599	9.151289	22.46585	3.461493	9.025646
Cs9g18170	0.474919	1.633972	0.220412	5.234231	0.418401	0.762698	0	0.488003	0	0.005729
Cs9g18180	13.10117	17.25441	35.9464	36.70311	51.99604	20.42641	0.657006	0.709587	0.069326	0.021719
Cs9g18210	0.628345	0.518938	0.623714	10.52934	3.075144	2.260365	0.00922	0.276059	0	0.013451
Cs9g18230	0.062643	0.041087	1.436141	0.600135	1.066892	0.26313	0.125338	0.211048	0	0.026762
Cs9g18240	4.875491	4.644456	27.68904	18.25363	36.41148	17.60675	1.927948	2.871719	0.092312	0.287686
Cs9g18250	2.416098	1.822742	3.623361	3.471828	2.589999	3.963695	0.912096	2.098112	0.873754	1.946555
Cs9g18340	35.56655	35.21449	38.13195	36.46732	19.8247	20.07141	18.50306	25.17997	14.85669	35.67181
Cs9g18370	9.214146	8.648355	10.78345	10.58314	9.673829	13.88118	3.680459	8.532756	4.292261	5.293221
Cs9g18410	4.04609	3.443395	6.269686	5.332262	10.12581	22.9059	8.919286	10.49463	9.519358	3.383424
Cs9g18450	0.523409	0.176619	1.26986	0.482885	1.097906	0.875355	1.577015	0.969155	0.877148	0.725204
Cs9g18470	35.1956	74.14044	7.974739	20.5462	39.43261	22.9089	36.15197	35.81874	7.092068	4.893845
Cs9g18480	0.923629	0.775114	1.874046	1.698814	1.319194	1.651707	2.081576	1.449143	0.11081	0.22269
Cs9g18520	1.039682	1.445571	3.681747	1.592705	2.470954	0	2.750137	0.98957	0	0.291255
Cs9g18550	2.707046	2.371502	7.046996	5.505144	8.872242	10.7048	11.38834	8.166583	9.895481	8.044518
Cs9g18610	33.28866	33.60729	26.1268	31.76133	18.53398	29.77166	13.33729	36.81092	45.17956	69.86906
Cs9g18700	44.50213	41.38784	34.19321	43.95329	25.23922	48.47301	89.99373	73.85008	19.14183	49.89027
Cs9g18710	30.49872	22.14376	35.95349	27.89541	50.61736	57.05762	181.8989	72.14872	90.20817	57.91355
Cs9g18780	9.183289	30.34401	1.519327	4.328201	0.520086	0.4779	1.009904	3.210357	0.50091	0.276276
Cs9g18910	0.100699	0.171709	0.37866	0.251002	1.126098	2.625117	1.419543	2.519067	15.74859	5.491119
Cs9g18920	0.658264	0.92942	1.208449	0.822439	0.435888	0.958224	0.06837	0.041336	0.0479	0.039789
Cs9g18960	0.010944	0.015275	0.107981	0.130371	0.819285	1.028171	1.55962	4.592404	0.999839	2.51141
Cs9g19000	8.480097	4.687533	1.576601	1.324333	0.278243	0.965323	1.127077	7.294594	0.165956	0.145187
Cs9g19150	6.995872	5.520308	6.494984	6.080017	3.686928	4.651338	1.489155	6.784803	2.128978	4.483008
Cs9g19160	113.6552	95.10803	102.1964	121.9487	45.29027	52.70016	34.98043	66.18605	4.803702	8.592372
Cs9g19170	60.04306	65.53718	139.4597	184.0039	67.06202	87.12137	35.11374	100.1776	24.23016	45.62155
Cs9g19270	133.6925	159.5191	209.3229	215.122	411.525	515.9579	695.4124	571.0035	398.351	402.278
Cs9g19310	5.525624	4.715136	4.559268	4.892369	5.141172	7.611288	4.609265	12.45063	12.53217	12.07891
Cs9g19340	53.00864	42.59593	70.8525	58.02081	44.21582	50.25462	15.13535	31.33533	15.81326	16.6057

Cs9g19350	5.772002	8.719393	2.6369	3.996843	1.407649	1.525768	1.292309	1.841862	0.463967	0.443732
Cs9g19470	5.696809	6.895183	6.559721	5.792993	7.765669	7.577285	7.062169	12.60551	1.02165	3.262144
orange1.1t0(	12.59489	14.80134	7.658814	9.055819	4.097084	7.400211	3.644917	7.737162	0.877976	1.231532
orange1.1t0(	33.85548	28.26897	47.09812	36.47928	55.54769	42.42331	77.16771	36.8801	39.8113	24.9102
orange1.1t0(	4.430127	7.473304	6.32607	9.05967	7.864004	5.036687	17.57659	36.6143	7.308371	11.89851
orange1.1t0(	3.496271	1.674538	1.612055	1.452562	0.657293	1.000915	0.95353	2.98827	0	0
orange1.1t0(	0.029918	0	0.370133	0.332294	0.471872	0.950454	0.193539	1.215772	0	0.20034
orange1.1t0(	4.993768	8.178002	3.913093	5.069524	3.402375	4.420733	1.931434	3.732055	1.801862	1.743367
orange1.1t0(	3.563658	1.853878	3.969178	3.345461	3.792455	4.533216	4.325413	2.984451	2.948132	2.76769
orange1.1t0(	1.031621	0.343442	0.651812	0.45458	0.781909	0.693683	1.119821	0.471971	1.371755	0.293678
orange1.1t0(	76.83279	65.62354	57.04714	47.92541	71.04503	78.16677	93.21135	141.2724	28.04872	68.55123
orange1.1t0(	1.444916	1.291076	0.741642	0.500168	0.490677	0.686588	0.407916	0.917075	0.047108	0.078422
orange1.1t0(	4.660345	4.666895	2.611002	2.82284	1.066886	1.185258	0.846591	2.6303	0	0.222896
orange1.1t0(	0.866033	0.890293	1.051697	1.29258	2.314924	1.785254	2.157674	2.10606	0.529129	1.188481
orange1.1t0(	24.03788	36.80035	13.93679	26.08577	41.21846	22.52606	31.66572	41.10767	5.999642	6.596064
orange1.1t0(	634.6166	557.0355	304.9499	378.2497	105.2338	150.192	93.57269	196.8617	365.33	685.0887
orange1.1t0(	0.610205	0.884109	1.655972	0.721018	1.534698	1.798663	0.844264	1.520889	1.408727	1.535727
orange1.1t0(	11.84031	11.67422	12.06043	12.37158	8.034776	12.22308	6.253138	5.860294	8.463038	2.839495
orange1.1t0(	20.18119	19.18075	6.781476	7.269169	4.745588	7.210389	3.773769	4.479311	4.011379	1.780088
orange1.1t0(	14.68156	21.00803	12.47831	16.88145	7.264327	12.02578	7.6984	17.44355	15.40539	29.79546
orange1.1t0(	9.862095	12.46932	7.604416	7.946104	4.471476	5.829323	4.352197	7.03805	7.099278	10.82369
orange1.1t0(	3.655103	3.461666	2.068919	2.198558	1.567003	1.605236	2.042059	1.671096	2.184435	3.039257
orange1.1t0(	1.528878	0.979686	1.280276	1.29599	0.632878	0.569955	1.264644	0.832469	0.413612	1.635191
orange1.1t0(	2.885899	3.133384	2.657489	2.560467	2.267951	2.57393	3.188586	2.508945	0.810293	5.060408
orange1.1t0(	1.767773	1.872333	1.64223	1.752214	0.913393	0.967196	1.072923	1.175803	0.425483	0.494706
orange1.1t0(	7.636705	17.02007	7.407387	11.3885	2.137791	3.148881	0.839052	2.034574	0.155975	0.728279
orange1.1t0(	1.062144	1.070071	1.457012	1.504029	0.785998	0.883316	0.749924	2.647248	0.7395	1.519134
orange1.1t0(	20.42228	37.53472	7.560316	11.68615	4.889709	4.271507	1.585319	3.836439	0.836034	0.902188
orange1.1t0(	0.748734	2.918647	0.696171	0.587277	0.012224	0.373719	0.11454	0.041165	0.034329	0
orange1.1t0(	2.331326	2.417374	18.31857	21.4733	15.41622	93.31143	13.78464	31.98757	0.900776	0.924172
orange1.1t0(	3.743184	4.043021	6.084555	5.804691	8.324389	8.63166	7.763002	6.305672	4.584769	4.135917
orange1.1t0(	19.52289	24.9722	32.47733	29.01592	39.35084	33.04391	21.10902	55.7404	19.48318	34.10243

orange1.1t0	5.244189	7.451515	3.700674	5.072797	1.441989	1.753002	1.457417	2.688743	0.391687	0.43054
orange1.1t0	5.963669	7.684929	2.787189	3.916507	1.661693	1.219377	1.529644	1.248491	3.628894	0.328625
orange1.1t0	1.502816	2.29446	0.538393	1.059947	1.562735	1.842843	0.754673	0.72149	0.372313	0.2932
orange1.1t0	8.50706	10.15545	4.956233	4.855128	6.944379	6.299622	7.670091	7.26305	13.3755	5.31804
orange1.1t0	11.17599	12.45333	11.25227	13.04538	4.514818	4.827702	2.070616	3.287693	1.331581	0.9412
orange1.1t0	6.864837	8.348662	19.14248	35.31906	20.51236	58.6841	17.89667	30.59476	77.80202	98.93558
orange1.1t0	0.315783	0.342932	0.270402	0.326573	1.974233	1.845814	3.753966	18.96561	0.197429	0.237736
orange1.1t0	0.603597	1.137783	7.354294	11.27977	1.09339	2.98705	0.033218	0.204491	0.024841	0
orange1.1t0	195.5082	150.8698	72.72124	75.15475	136.6518	130.6009	269.0811	123.83	37.14042	15.23866
orange1.1t0	7.907045	9.925725	4.173034	4.15221	1.741662	1.406701	1.651378	2.495488	0.302346	0.414889
orange1.1t0	15.23833	20.87497	8.059246	8.109226	3.378351	4.228188	1.528076	3.985759	0.012211	0.051389
orange1.1t0	0.809736	0.793159	1.449925	1.174285	0.570404	0.761376	0.342046	1.127949	0.640505	0.800168
orange1.1t0	0.184113	0.18666	0.911688	0.777946	3.406907	3.614102	1.995761	1.478333	3.595194	6.898586
orange1.1t0	0.540668	0.239196	0.476023	0.680924	0.774815	0.768782	1.338035	1.16079	0.436609	1.484917
orange1.1t0	9.93177	10.22166	10.27042	9.738906	10.78744	10.52027	4.083862	9.346623	8.161988	6.825094
orange1.1t0	8.199837	10.55132	8.744733	10.4548	0.346424	0.631301	0.886102	4.005298	0.237303	1.030978
orange1.1t0	29.60921	34.28604	31.21764	26.2177	17.17333	15.42211	9.127763	28.21876	6.92559	12.82611
orange1.1t0	14.65785	19.22251	11.06169	9.131377	6.48166	5.415212	1.453458	4.83386	1.499614	2.365993
orange1.1t0	3.615572	5.081244	4.817072	5.668317	1.917994	2.833852	1.963691	4.637754	1.816779	7.344613
orange1.1t0	5.521861	5.917239	9.673125	10.33971	6.555076	7.106638	7.228833	7.839523	2.962112	7.040791
orange1.1t0	4.57643	7.862283	25.02957	26.89912	1.628157	5.591253	4.389865	10.03476	0.51392	2.976632
orange1.1t0	2.18631	3.293136	7.130455	8.65913	5.294303	7.03778	2.532144	7.746689	6.952692	13.71769
orange1.1t0	108.7272	256.2221	110.3581	222.3094	199.9228	150.0186	175.7183	397.9875	129.5524	148.3271
orange1.1t0	20.78344	44.48942	22.92514	54.33434	45.28585	34.61343	21.02167	81.60322	37.18965	19.75345
orange1.1t0	1.23327	1.68089	1.032697	1.212875	0.640278	1.347391	0.394488	1.160922	0.208971	0.158725
orange1.1t0	6.975957	7.064807	7.839502	7.173333	5.409332	9.817328	3.590819	3.571896	0.81778	1.0328
orange1.1t0	10.95942	12.38127	15.5856	17.60743	8.441801	10.84527	4.146687	8.802305	2.854141	7.083222
orange1.1t0	49.03436	70.36082	61.0324	67.82059	19.1509	22.72672	41.97725	39.19804	32.78307	70.45188
orange1.1t0	7.464069	8.277636	4.380494	4.323356	2.028809	2.456702	1.939381	1.923169	0.074046	0.147175
orange1.1t0	0.568638	0.612272	1.108723	1.227154	1.284593	1.545664	0.640123	1.529637	0.375078	0.398832
orange1.1t0	3.681535	4.220204	6.5474	6.703565	4.802041	9.906413	3.335257	9.936467	5.682459	8.642808
orange1.1t0	57.14517	44.55675	64.96854	55.00992	81.86963	88.52099	68.41906	48.41162	50.54628	17.50753

orange1.1t0(	141.622	140.7658	99.22934	92.36728	63.18098	69.12797	130.3974	154.651	13.30603	13.93911
orange1.1t0(	6.356344	10.49637	1.592383	4.393821	0.141349	0.382361	0.117619	0.210965	0.167663	0.120947
orange1.1t0(	9.082228	9.82299	12.25365	5.086662	6.731304	4.359066	4.76757	24.82645	24.59572	75.52754
orange1.1t0(	52.3924	44.1138	48.4648	45.85273	33.34549	37.83344	20.3747	39.61837	23.19043	56.75871
orange1.1t0(	31.09896	36.09334	16.56524	18.78479	5.540814	6.367495	4.921848	20.5415	6.854456	14.89333
orange1.1t0(	39.53383	45.42786	29.60605	37.03475	25.75897	25.31063	15.98643	50.45901	14.53866	26.99284
orange1.1t0(	32.99956	38.87476	15.07742	15.37107	13.7663	9.491097	15.17274	26.88254	70.16612	64.72149
orange1.1t0(	5.588742	9.075036	3.922147	2.982929	3.994452	4.970832	3.317765	0.563952	8.8502	2.385132
orange1.1t0(	5.267325	4.460833	4.418264	5.295827	3.153763	3.813536	6.414864	10.04781	14.74945	32.06723
orange1.1t0(	9.045237	6.066683	7.824798	4.921379	6.060437	6.631738	5.584998	4.272554	13.82521	3.914484
orange1.1t0(	12.89688	11.69565	14.12279	16.07681	29.31414	43.88492	54.896	50.25667	111.0855	86.89025
orange1.1t0(	2.831252	3.466549	1.845661	2.803546	10.00362	16.05556	11.10068	10.97338	20.86752	2.670569
orange1.1t0(	9.61908	12.80481	9.223975	12.87105	27.94699	31.72011	53.79209	96.16966	46.94814	105.3733
orange1.1t0(	13.6589	19.12296	19.37725	23.59845	8.990807	12.42721	12.56723	22.84465	17.51458	39.53673
orange1.1t0(	84.07827	119.0711	43.98692	54.83071	38.46971	41.02397	31.85216	60.06771	17.9732	32.19979
orange1.1t0(	4.639029	5.229795	2.409891	2.795112	0.826612	1.033718	0.415747	1.132117	0.103377	0.131248
orange1.1t0(	48.1839	47.29148	25.76828	27.10762	25.84255	20.04605	16.18094	14.82113	21.37567	46.78918
orange1.1t0(	12.92577	10.79283	9.132221	8.225325	7.521301	7.785628	2.904597	5.851323	3.439297	1.608605
orange1.1t0(	7.107761	8.379648	6.764901	6.811483	2.632197	2.853328	1.905029	11.72779	2.024204	6.126499
orange1.1t0(	2.234223	2.096253	3.737271	2.854648	3.073301	5.659701	1.672338	3.685078	8.075326	5.394937
orange1.1t0(	198.6438	290.759	270.1163	359.5637	30.09803	69.32511	41.54033	90.32101	44.20679	115.307
orange1.1t0(	5.420219	5.867652	5.71008	6.500481	2.033435	3.535733	0.749781	2.617601	0.146	0.349763
orange1.1t0(	52.37095	81.10587	22.35996	37.05251	21.2103	16.3405	20.08881	33.27277	12.64644	13.57787
orange1.1t0(	5.653382	3.634381	10.75442	7.092736	17.54477	15.41012	27.97119	9.843711	4.381593	3.416022
orange1.1t0(	88.22203	64.79136	47.71712	52.53279	42.9885	53.58282	24.06097	22.69072	39.12416	25.29996
orange1.1t0(	1.827736	1.577759	3.590303	2.726406	1.365216	1.791986	1.111628	0.196959	0.831141	0.048378
orange1.1t0(	42.56714	51.66731	39.7436	47.15142	55.72499	60.31022	85.3305	95.76851	141.67	80.89857
orange1.1t0(	14.80195	15.54855	5.534072	7.830196	1.926546	2.26157	1.300731	1.011612	0.541431	0.648109
orange1.1t0(	23.72965	25.59906	23.99314	29.08065	7.47402	8.739396	4.290652	10.44873	0.469712	1.030634
orange1.1t0(	0.923245	0.651175	1.795618	1.010022	2.394767	2.05237	1.928493	3.25847	0.094575	0.193783
orange1.1t0(	11.2584	9.550174	8.102944	8.003117	2.675686	3.649843	4.501522	7.511017	0.839077	2.017042
orange1.1t0(	9.365393	9.981511	5.125464	6.743486	1.418607	3.102937	2.155489	3.751461	2.17477	1.779421

orange1.1t0	35.66457	27.86906	25.49689	22.95398	26.72976	36.01628	67.96127	46.1896	226.741	198.396
orange1.1t0	19.75069	24.76029	21.70303	19.93287	23.9894	13.03744	30.45239	19.51336	39.85162	22.1984
orange1.1t0	155.8565	119.3298	120.5315	107.8627	46.35247	62.45953	36.53893	19.00838	0.44816	0.209053
orange1.1t0	7.614373	8.556099	4.729932	3.741308	3.799463	4.270903	1.088391	2.845795	1.783159	1.447831
orange1.1t0	1.979581	2.26427	18.3174	13.15439	24.65753	21.76351	5.820782	41.94833	3.137977	5.825791
orange1.1t0	57.31952	30.9833	32.65151	34.77089	26.21319	35.19062	14.75778	36.73578	114.115	147.7274
orange1.1t0	72.13879	66.57202	88.92272	93.13643	47.3021	49.80266	105.942	164.5449	26.25711	108.9053
orange1.1t0	38.03795	56.38326	77.94452	94.12708	315.7836	280.7109	625.9241	260.0953	86.60118	89.10013
orange1.1t0	0.329649	0.926505	0.401407	0.238245	13.73205	12.05368	3.206034	8.322563	4.465836	5.021693
orange1.1t0	21.89816	31.24049	6.403925	13.42253	0.541527	1.02347	0.09967	0.604769	0.721103	0.298538
orange1.1t0	5.532042	5.23695	25.68595	14.02283	31.38401	17.29308	11.86113	24.2667	2.216118	3.095272
orange1.1t0	0.452482	0.285606	1.534939	1.188538	3.308568	2.051699	1.215116	0.979388	0.302002	1.027639
orange1.1t0	49.17703	307.8655	2.095256	36.60039	11.85957	11.15513	42.13677	114.6779	69.37024	81.03898
orange1.1t0	11.0627	11.95389	20.04393	17.80303	24.89371	24.86398	27.29775	29.30898	22.17794	26.19895
orange1.1t0	1.969704	2.34529	2.506041	2.12118	2.40652	3.370902	1.144892	3.98107	2.087462	1.882175
orange1.1t0	1.778373	1.771823	4.069854	3.816717	4.333203	5.224951	1.578209	2.593989	0.519226	0.424115
orange1.1t0	20.64709	11.83617	8.898342	10.21733	7.035342	8.584557	9.071743	13.03955	11.71695	29.36377
orange1.1t0	3.946047	4.707846	3.587074	4.718626	1.905794	2.579153	0.634876	1.928891	2.109	2.375144
orange1.1t0	0.699892	0.911167	1.527093	1.969305	3.836284	2.668326	4.993327	3.887409	5.06061	7.351385
orange1.1t0	0.013643	0.021332	0.019481	0.014657	0.064847	0.308116	0.522714	0.196417	1.449368	0.23253
orange1.1t0	26.20029	28.45	17.61395	20.89381	3.950582	4.247086	1.594602	3.964898	0.171294	0.361757
orange1.1t0	13.12863	19.98123	8.973595	11.81097	7.725371	6.299074	5.339916	9.616436	2.258672	3.011701
orange1.1t0	0.611603	1.295997	0.217128	0.319127	0.137812	0.166271	0.047396	0.067424	1.583284	0.403469
orange1.1t0	2.031889	1.557865	4.529482	3.818986	9.99088	5.272161	5.879621	5.05667	11.04403	9.592697
orange1.1t0	24.64477	26.86259	59.30258	49.65034	92.73815	52.31336	49.26744	57.35806	70.30589	89.9689
orange1.1t0	4.956465	5.512348	5.285005	4.958276	4.67703	5.947918	1.884291	4.683247	5.512738	4.717079
orange1.1t0	1.870458	1.460534	1.300052	1.405067	1.302322	3.319884	1.335079	3.599097	0.155455	0.229791
orange1.1t0	0.080129	0.371518	0.323499	0.666363	3.419796	12.74201	23.83183	39.95756	155.2044	163.3223
orange1.1t0	5.713649	2.962107	4.038978	5.106863	3.274958	1.211543	2.157077	1.249411	2.7671	1.665799
orange1.1t0	67.21329	52.22335	62.19863	59.09536	64.07691	107.4809	80.4602	217.3653	8.790944	22.4665
orange1.1t0	20.0265	23.69786	50.03949	61.20623	47.63712	86.93723	23.32109	174.2061	20.29124	40.7334
orange1.1t0	3.662447	3.085627	1.848933	1.959849	9.452701	5.834873	24.90835	8.180667	9.102721	5.808643

orange1.1t0(	8.002926	10.1011	11.46491	15.09948	3.020357	5.741951	1.730663	7.029432	1.577189	3.05053
orange1.1t0(	19.01412	19.17956	26.66034	24.32341	7.74036	11.20826	4.493872	14.05005	3.079469	5.055586
orange1.1t0(	1.26072	1.643724	2.394351	2.115773	1.527829	2.144057	2.406969	3.746572	3.497299	1.462943
orange1.1t0(	2.857279	3.688985	3.357723	3.819099	3.501328	3.731486	1.405903	4.211142	2.484234	2.753776
orange1.1t0(	0.066179	0.304623	0.328195	0.265831	1.012293	0.842745	1.146418	0.443316	1.80413	0.660621
orange1.1t0(	7.304797	6.782502	11.33613	9.51456	9.914144	4.860833	2.890193	7.973532	9.132343	38.09635
orange1.1t0(	0.010127	0	0.584271	0.206015	0.305251	0.528442	0.335508	2.684113	0.42632	0.325316
orange1.1t0(	1.041858	1.153759	1.781475	1.425359	1.682167	2.38594	1.863727	1.284039	0.384936	1.091842
orange1.1t0(	5.013754	6.04201	4.838876	3.233281	2.655902	4.425141	1.547971	3.68625	0.727331	2.237786
orange1.1t0(	5.450181	4.422247	4.397527	4.055817	4.40243	5.615827	3.889212	3.594391	9.049449	1.987255
orange1.1t0(	8.494684	7.205773	7.422887	7.632503	7.750937	10.99188	4.991744	6.296505	17.00571	5.416715
orange1.1t0(	1.212087	1.108502	5.113517	5.5703	12.78573	10.49336	1.72743	6.985607	2.874388	12.14392
orange1.1t0(	0.521364	0.831472	2.492773	2.545399	7.393443	5.217006	0.604448	3.826731	1.522	7.264538
orange1.1t0(	1.888731	1.169145	2.164622	1.01317	2.211874	1.319872	2.739526	1.260464	1.406882	0.382251
orange1.1t0(	66.91366	82.30992	47.21359	52.31995	22.85246	23.07795	13.0145	26.58337	17.73853	29.76806
orange1.1t0(	11.83393	16.77602	9.431777	12.05339	4.672263	5.369259	2.488461	5.963819	2.725598	6.962101
orange1.1t0(	2.061726	3.481661	6.154221	6.883017	7.612474	7.038621	6.104183	11.51569	1.522144	4.692959
orange1.1t0(	262.7376	297.3642	141.0546	144.1573	84.23937	68.98597	26.09446	71.73619	39.46531	44.37692
orange1.1t0(	0.907377	0.643314	0.568101	0.271833	1.321188	0.98322	2.671591	4.016602	12.68385	14.95377
orange1.1t0(	1.997295	1.074222	1.340176	0.482968	1.155965	1.919384	3.43928	5.541254	0.718471	0.652711
orange1.1t0(	10.13601	14.57092	4.837063	5.703241	0.540961	1.370229	0.344157	0.444121	0.098215	0.036446
orange1.1t0(	8.371893	8.934159	2.795523	3.695793	2.398155	5.240991	10.44064	5.852064	5.739157	6.189241
orange1.1t0(	5.599152	9.50069	1.638734	3.693127	2.81948	2.674313	1.068398	2.169025	0.271735	0.091625
orange1.1t0(	16.36589	16.02829	19.44006	15.48265	14.18546	17.5461	5.576361	16.23036	9.186773	7.351636
orange1.1t0(	2.074563	1.572263	1.454285	1.166144	1.441535	1.72171	0.356086	1.787462	0.44731	0.333671
orange1.1t0(	40.5171	7.036618	10.74173	9.605164	8.065007	9.887375	11.65758	8.364791	107.0583	14.88486
orange1.1t0(	7.615474	13.11746	4.469139	6.656869	5.910745	8.826323	2.833695	11.64458	4.452115	4.772076
orange1.1t0(	10.9131	14.40122	3.135497	5.931519	0.136993	0.130059	0	0.038062	0.113905	0.019726
orange1.1t0(	1.623838	1.787613	2.719204	1.929193	1.285884	1.622293	0.5393	4.152981	0.424187	0.339954
orange1.1t0(	20.40986	17.52138	9.448325	10.29002	2.17747	2.23755	1.259203	2.701976	0.15212	0.10058
orange1.1t0(	11.7769	15.10503	13.26543	14.26004	5.626103	6.641623	5.688249	8.155885	8.347848	11.09292
orange1.1t0(	47.58699	39.6737	56.95421	67.94266	14.05594	20.50452	18.32562	27.97916	13.41279	29.01291

orange1.1t0	13.30846	14.89054	16.421	15.46966	13.20623	12.5092	7.04109	16.69458	7.981243	8.381725
orange1.1t0	61.81136	72.2498	76.71766	94.85323	18.304	16.70548	36.28977	37.01184	19.72732	35.46901
orange1.1t0	4.099449	4.667283	3.334271	2.970068	7.288726	9.731173	4.904051	7.613306	2.132091	2.483063
orange1.1t0	59.31671	94.00166	43.40017	55.54577	45.37549	44.433	23.977	48.36532	26.16738	37.68173
orange1.1t0	3.389931	3.343517	4.040624	4.342723	1.565893	1.723455	0.963839	1.962366	0.665999	0.700873
orange1.1t0	1.144581	1.529794	0.841668	0.928176	0.645761	0.629078	1.45125	0.871279	0.526979	0.612254
orange1.1t0	8.959536	8.134109	5.484309	4.934714	4.534775	4.170917	4.214505	3.709495	6.109745	4.303733
orange1.1t0	290.3784	245.2872	203.033	225.1357	147.62	138.3434	123.2574	71.63808	109.8183	75.43437
orange1.1t0	30.12318	49.35907	17.90561	63.27119	0.036571	14.12521	16.39647	39.57925	0	49.3204
orange1.1t0	23.58489	11.20625	6.8249	2.999508	6.381036	2.01618	0.326044	0.685426	2.70228	4.272883
orange1.1t0	5.652732	9.050212	3.896347	6.476312	3.751981	2.624312	2.349419	3.937747	0.181669	0.127285
orange1.1t0	4.343975	12.73384	0.384021	2.388934	1.22559	1.051871	2.163118	3.988601	0.154985	0.161113
orange1.1t0	3.942363	1.837597	3.097383	3.936455	3.691156	5.937897	5.915868	5.243117	1.878518	2.644257
orange1.1t0	1.838384	5.26799	2.019903	2.986293	1.885868	1.1002	2.659975	3.840695	0.180894	0.904471
orange1.1t0	0.004349	0.013512	0.032565	0.010821	0.25919	0.079741	2.213226	0.563991	2.965949	0.846426
orange1.1t0	13.1334	16.31717	18.861	20.1079	11.69977	17.24244	5.215179	10.37141	4.036802	9.051379
orange1.1t0	9.604695	9.121948	16.5723	12.68927	3.630602	10.57051	3.290767	12.13773	0.056109	0.048059
orange1.1t0	13.95999	21.73667	6.389753	7.557133	0.332675	0.487701	2.292424	5.115986	1.331598	3.078537
orange1.1t0	4.458805	6.490453	5.640661	5.996206	6.854768	6.468721	4.120089	9.208774	4.237438	5.493586
orange1.1t0	0.139209	0.48278	2.557245	2.638231	14.77566	9.850109	18.18288	8.225482	175.716	87.15835
orange1.1t0	2.177541	2.042555	3.390656	2.832331	0.670184	1.143085	0.610171	1.296298	0.377817	0.381809
orange1.1t0	17.30189	18.18155	17.09646	16.87649	9.135586	10.95409	7.36261	9.62113	7.403713	11.64615
orange1.1t0	1.480843	1.603683	6.129778	4.198912	3.73612	4.030671	7.766732	9.607342	2.208706	4.958196
orange1.1t0	14.35683	27.56006	8.68439	14.3766	12.30728	21.61743	5.800308	26.32874	8.764625	9.6279
orange1.1t0	3.921361	5.191409	7.89624	7.806312	12.04335	11.51532	6.059391	13.44713	6.470628	8.54765
orange1.1t0	4.340843	5.695619	3.044413	4.539861	0.995865	0.765757	0.311621	2.161449	0.043354	0.077059
orange1.1t0	6.61939	8.027594	5.767584	6.425442	4.990357	5.441916	2.24322	10.2002	2.819033	4.971427
orange1.1t0	7.658564	7.822134	17.92507	11.47971	19.32136	16.48064	5.865429	6.173498	7.705757	13.57888
orange1.1t0	0.280731	0.275256	0.551671	0.63855	1.920964	0.802238	1.780503	2.079149	7.129183	5.76895
orange1.1t0	29.40669	36.50639	17.172	27.87643	6.508381	6.696316	4.007414	6.309649	1.241915	2.144005
orange1.1t0	8.69566	10.95094	11.48745	12.80538	7.722842	8.58194	2.619971	8.691748	4.227285	2.986061
orange1.1t0	2.144749	7.472518	5.213145	6.4434	3.342699	2.047295	1.008278	7.621364	2.528146	2.538969

orange1.1t0	8.54576	7.576669	7.214992	8.160116	0.761756	2.357693	0.109535	1.09687	0.047536	0.209505
orange1.1t0	0.899713	0.75017	1.054824	1.502238	0.269232	0.871868	0.033138	0.35071	0.017635	0.136477
orange1.1t0	0.559964	0.476871	0.737552	0.627912	0.429933	0.412626	0.284339	0.903625	0.365264	0.651128
orange1.1t0	5.236641	2.902518	0.657556	0.422436	0.167925	0.238411	2.37535	2.510616	0.57231	0.453522
orange1.1t0	57.14676	42.86886	36.72996	36.27958	106.4973	96.29759	89.77908	31.25845	41.78323	51.57806
orange1.1t0	0.068198	0.079977	0.236093	0.134454	1.230809	0.66888	1.347937	0.21272	0.52411	0.367148
orange1.1t0	2.213021	2.566658	1.739657	3.405452	0.351008	1.114617	0.593885	0.54875	0.053397	0.198149
orange1.1t0	24.35024	19.91949	18.23191	22.08621	16.0343	18.07068	7.368763	2.336102	3.460106	0.269122
orange1.1t0	108.1826	97.39212	89.11543	76.6155	51.16016	56.32616	26.89627	30.51135	9.865031	7.120122
orange1.1t0	45.88685	40.94233	44.15728	45.89198	20.91379	36.3218	7.974311	9.760487	2.567156	0.554993
orange1.1t0	35.87556	43.95272	37.74988	35.34151	47.99601	45.43551	40.19511	78.99314	8.566664	18.40712
orange1.1t0	68.31196	50.5874	55.49414	57.21181	36.96445	44.59403	9.465258	9.350624	10.59054	5.057088
orange1.1t0	0.278309	0.260137	0.990209	0.692399	0.434125	0.962303	0.689552	1.171198	0.337989	0.209308
orange1.1t0	3.535128	10.16948	1.277696	2.686132	1.548211	2.135007	1.708645	2.244998	1.839345	1.83285
orange1.1t0	6.554499	5.165092	12.11296	9.863107	4.171185	9.724812	0.586633	2.185112	0.159433	0.27487
orange1.1t0	13.67496	10.3639	23.35254	15.74007	9.566691	17.46118	0.855709	4.453286	0.122009	0.113307
orange1.1t0	1.13253	0.847721	1.693622	2.017433	0.745402	1.605343	0.116934	0.568139	0.023364	0.033786
orange1.1t0	2.200795	1.989811	3.998941	3.438468	1.939914	3.045143	0.311338	1.236082	0.060173	0.046747
orange1.1t0	30.46464	29.08617	74.75275	52.03815	32.06895	31.61276	2.102961	12.79252	0.299613	0.446304
orange1.1t0	44.29244	40.59424	78.5203	66.18733	43.91233	47.63671	18.57542	20.0191	5.496494	7.846865
orange1.1t0	1.110855	0.575246	1.787082	0.952685	1.239873	1.53996	0.380149	2.404072	0.645795	0.842269
orange1.1t0	3.21543	3.037982	5.581314	4.160983	5.087408	6.494144	0.481587	2.227946	0.154105	0.074027
orange1.1t0	0.519604	0.506116	1.243178	0.624761	0.627609	0.920856	0.285161	1.859526	0.317763	0.081109
orange1.1t0	1.58594	1.070347	5.249089	3.661388	2.544313	3.476094	0.512953	2.177725	0.163338	0.214397
orange1.1t0	1.213567	1.165851	3.003964	2.881273	1.837562	2.882922	0.43668	1.540842	0.032721	0.117015
orange1.1t0	1.603881	1.300484	3.773115	3.247166	2.102084	2.726807	0.342938	1.581887	0.130235	0.30159
orange1.1t0	9.64539	3.959009	13.15016	8.492592	7.632947	15.35936	3.007861	6.247275	0.77312	0.622168
orange1.1t0	1.271799	0.956382	2.900936	2.006607	1.052171	1.969147	0.245675	1.066923	0	0
orange1.1t0	4.410535	2.810135	7.075339	5.238747	6.935575	9.718111	1.556706	5.644933	0.35867	0.400856
orange1.1t0	4.451383	4.372914	5.792799	5.010429	9.485583	9.442897	4.249954	3.382941	9.34255	4.199468
orange1.1t0	4.556654	5.835475	3.714931	5.407148	2.842804	3.267439	1.469693	3.696949	1.670868	1.841036
orange1.1t0	18.41913	16.55503	28.94144	20.90584	10.48989	17.08585	5.23228	7.875373	0.044506	0.05521

orange1.1t0	13.59647	8.311863	7.214877	6.624563	9.63283	11.979	7.738963	10.36852	7.423124	1.637514
orange1.1t0	96.3818	80.18577	36.56089	37.60127	22.38675	23.99281	74.74996	52.74884	5.892922	1.974186
orange1.1t0	8.662102	11.37409	22.10413	17.93767	27.85651	18.07596	24.69209	46.99536	1.182897	6.835621
orange1.1t0	1.682714	1.767153	1.902831	2.040356	2.396485	2.818995	1.24437	3.191589	3.334638	2.382108
orange1.1t0	6.702936	3.789334	3.868089	3.598638	3.062627	5.934903	1.241703	3.843941	1.819293	1.897171
orange1.1t0	6.986659	6.184977	5.62882	4.338821	6.987224	7.704901	1.195792	5.683961	0.027523	0.061251
orange1.1t0	4.165349	4.408711	5.418367	4.887404	3.210663	3.898288	2.311666	6.511156	0.268594	0.6549
orange1.1t0	5.008863	6.936721	6.814722	6.150974	5.638658	5.891707	5.68129	14.10844	2.626836	9.564066
orange1.1t0	0.822645	0.713795	1.141353	0.52708	2.721981	1.274496	1.161639	2.840608	0.097086	0.397408
orange1.1t0	0.294453	0.243822	0.89789	0.547157	1.306731	1.194713	2.645433	1.855826	1.083994	1.408584
orange1.1t0	3.403712	4.208691	0.387251	0.662685	0.442727	0.337354	1.562007	1.347632	0.922426	0.424463
orange1.1t0	315.7521	322.6167	411.7902	365.4925	232.5906	171.4281	57.54999	86.90641	97.40937	150.7805
orange1.1t0	1.558088	1.767732	1.311029	0.998276	0.81928	0.739199	0.567127	1.765724	0.413882	1.508355
orange1.1t0	13.61933	30.24582	6.64552	9.022732	1.857874	2.539452	2.529876	4.61486	0.824151	2.441904
orange1.1t0	24.6824	10.01629	5.166656	6.006069	0.81006	3.208876	0.565998	1.82025	0.63439	0.370427
orange1.1t0	0.540782	0.458807	0.259151	0.240244	0.714768	0.391526	0.573254	0.921386	0.14074	0.877053
orange1.1t0	11.1783	8.785181	7.559703	5.909217	3.916977	5.338463	1.603119	2.628635	1.955353	1.29932
orange1.1t0	2.056039	1.66071	1.050285	1.193157	0.414658	0.601447	0.742509	1.259387	0.329385	0.695171
orange1.1t0	239.0784	252.8748	257.2018	330.6117	72.25873	153.681	155.1228	145.0116	250.829	362.8422
orange1.1t0	7.099722	9.514978	14.32787	11.9924	5.22214	5.235441	4.951218	13.83916	0.364764	0.77527
orange1.1t0	4.21608	4.140571	4.433875	4.938196	2.649256	3.050289	1.354176	1.570141	1.900357	1.332302
orange1.1t0	36.15713	32.48039	21.98235	26.1006	11.62203	14.66621	15.46447	17.16091	1.907353	3.473709
orange1.1t0	6.948518	5.763271	3.368072	3.885088	1.318272	2.321997	0.924373	2.236344	0.392939	0.58451
orange1.1t0	0.013076	0.007281	0	0.004955	0.024674	0.037606	0.305536	0.585766	2.82341	12.75451
orange1.1t0	22.1356	16.83357	16.95483	21.78956	7.710945	9.970502	18.89	15.94117	33.18503	33.79625
orange1.1t0	0.589623	0.726062	0.060401	0.085376	0.005594	0.071637	0.106484	0.775877	0.439497	3.149997
orange1.1t0	3.286387	1.374745	0.997197	1.517276	1.784258	2.823043	1.727332	0.333105	1.401217	0.311991
orange1.1t0	56.50806	74.62743	46.64686	55.19312	11.61116	16.66002	19.7822	60.47933	65.46258	71.1947
orange1.1t0	8.679329	8.58776	17.34	16.35228	20.88755	23.41031	13.58092	23.0792	11.35029	13.87661
orange1.1t0	2.553537	0.587858	11.39811	7.087593	28.49402	54.41459	9.938416	10.46768	1.551323	3.600903
orange1.1t0	2.370514	3.202306	3.706486	4.53384	2.444413	4.094776	3.5555	8.6148	8.372924	43.1085
orange1.1t0	42.72285	59.97019	44.14474	51.55029	21.15647	29.31454	26.24743	64.74749	37.35525	82.22047

orange1.1t0	1.664776	1.648695	1.660819	1.672756	1.079169	1.098685	0.571938	1.449279	0.115325	0.26957
orange1.1t0	14.97146	16.87059	11.73809	11.15781	10.92905	7.175181	3.23847	3.778125	1.510472	0.930419
orange1.1t0	0.409543	1.060802	1.225321	2.748741	2.039595	57.39248	2.86555	11.49719	0.161466	0.095449
orange1.1t0	0.041084	0.019367	0.040768	0.012774	0.175535	0.122289	1.694377	0.967158	7.605906	9.314626
orange1.1t0	50.97459	38.53195	10.8658	16.20591	5.031174	8.984418	6.873501	7.130983	199.5205	275.4619
orange1.1t0	3.398701	3.725933	2.289167	2.583707	2.040906	1.764774	0.542177	1.700334	0.989093	1.682221
orange1.1t0	1.784324	2.039248	1.198674	1.322752	1.411664	1.309432	0.411165	1.054972	0.856343	0.942632
orange1.1t0	0.718265	2.486056	0.452998	0.831009	0.404349	0.366405	2.021044	3.524454	0.10261	0.904068
orange1.1t0	109.4243	115.1925	102.7804	102.044	56.22651	83.65656	33.26922	80.71534	8.704651	22.13276
orange1.1t0	0.463788	0.399279	2.37143	1.833648	10.63176	9.706987	0.293304	1.183844	0	0.146676
orange1.1t0	0.446136	2.331035	3.056647	2.626114	1.318694	0.348595	0.527509	0.837959	0.18113	0.34688
orange1.1t0	14.86923	22.30601	45.27199	46.64531	9.371098	18.99267	0.570378	2.26372	0.207213	0.188424
orange1.1t0	5.053836	2.953921	12.0405	10.26492	11.59893	16.33868	4.00644	6.543759	11.89343	13.81185
orange1.1t0	29.50721	26.38259	18.90669	18.38132	19.14936	13.87104	52.69744	39.95407	242.6915	179.7694
orange1.1t0	507.2373	460.1259	441.5075	449.3423	341.3021	471.8695	579.3612	211.357	351.2791	239.1497
orange1.1t0	24.52986	42.57148	15.98941	21.83641	7.044807	9.958548	4.905922	15.95925	1.463959	1.350549
orange1.1t0	0.15894	0.15704	0.82345	4.735515	0.077707	1.436804	0.083247	0.529015	0	0.06347
orange1.1t0	3.739159	4.937302	2.194433	2.728897	3.833502	7.39339	1.741976	6.890969	0.724696	0.8627
orange1.1t0	2.62806	8.115108	0.494523	1.664268	0.514809	0.805719	0.667227	1.640431	0.042161	0.117811
orange1.1t0	2.984286	3.877948	7.685873	7.564022	9.504565	11.45908	15.86839	19.88059	52.24461	53.32543
orange1.1t0	0.293322	0.26692	0.173366	0.150542	1.421723	0.715948	1.567357	1.063135	0.661359	0.927336
orange1.1t0	16.59563	17.42495	31.83639	29.40916	49.2809	48.50878	67.57336	39.39815	6.20263	10.59523
orange1.1t0	1.192006	0.775514	2.395407	2.473361	4.681102	7.045502	5.178282	7.490824	0.62008	5.022374
orange1.1t0	0.824113	1.033395	1.361337	1.383723	1.888047	2.641645	2.029409	2.365228	3.539131	3.724332
orange1.1t0	67.73032	70.83453	58.37592	51.73779	32.11407	20.69226	27.05864	31.99743	10.10068	13.55421
orange1.1t0	0.800006	0.575976	2.157545	1.199743	1.882322	1.705174	1.674624	3.005015	3.579448	5.272518
orange1.1t0	0.3362	0.355529	0.663422	0.30526	0.753121	0.567075	2.32914	0.657374	0.773385	0.406399
orange1.1t0	0.697057	0.662002	2.127649	1.801231	1.380158	1.565615	1.202965	2.416518	0.722728	1.777109
orange1.1t0	5.4546	4.525325	8.715743	10.00255	1.002199	3.341642	3.926819	19.5595	7.564871	13.21574
orange1.1t0	1.916665	1.319746	5.952365	3.618928	4.246138	3.275526	3.237688	4.199762	4.130575	5.400471
orange1.1t0	0.672976	0.613749	2.042518	1.52822	2.035891	1.746529	1.912445	3.509675	11.45159	11.00263
orange1.1t0	1.068907	1.514145	0.656139	0.732698	0.357602	0.452809	0.848255	1.170141	0.045744	0.023152

orange1.1t0	5.383756	5.905845	4.33145	4.25179	2.096941	3.134834	3.749097	13.03841	4.666408	17.81354
orange1.1t0	2.499699	1.722639	0.398313	1.680703	0.179673	0.231591	0.201871	0.252645	0.06457	0.038233
orange1.1t0	2.10508	2.390534	2.739039	2.991254	1.476248	2.250237	1.057539	1.190393	0.093316	0.357287
orange1.1t0	0.409458	0.527423	0.714425	0.706012	1.298203	0.479083	0.560209	0.415474	0.15117	0.380649
orange1.1t0	3.776127	4.639628	1.674256	1.875099	2.255786	1.710717	2.658089	1.234197	0.259327	0.241756
orange1.1t0	58.88456	43.28236	61.12177	53.19011	113.3583	120.3222	158.7917	86.24291	104.9367	71.81856
orange1.1t0	2.962547	3.000051	1.389385	1.357219	0.590211	1.034492	1.469457	1.704347	1.39095	1.901633
orange1.1t0	213.915	161.8826	309.732	247.5056	1004.455	845.6714	1273.185	397.7849	558.8454	374.6168
orange1.1t0	2.153503	3.326421	0.554124	1.188795	1.50831	0.823321	1.322184	0.780421	0.208283	0.126474
orange1.1t0	21.64043	11.29129	44.98778	36.69014	256.5323	312.2403	38.85217	33.48371	22.31231	7.579549
orange1.1t0	7.891625	7.500185	9.939643	7.983457	27.86561	34.40196	6.292696	7.039718	2.390458	0.979815
orange1.1t0	2.263777	3.436755	3.329655	2.424646	2.201242	1.984975	1.315598	1.768949	1.17641	1.208432
orange1.1t0	8.826549	12.11651	17.66128	15.00965	10.43986	13.11415	4.385762	16.43533	6.688485	13.41202
orange1.1t0	7.933649	8.681699	8.155583	8.698467	1.542363	2.698676	2.263824	3.715788	0.905151	2.613972
orange1.1t0	0.535555	0.475431	2.690794	2.581895	0.09453	0.30706	0.320643	0.581134	0.385675	0.986024
orange1.1t0	3.67737	2.422185	9.109773	7.108063	4.280682	7.278114	2.487014	2.223925	3.177495	2.278209
orange1.1t0	4.886407	3.764328	8.090668	6.686962	6.718184	15.80062	4.412363	6.979351	4.372504	8.04442
orange1.1t0	50.38354	36.63357	56.56361	50.77644	127.0279	83.75487	138.5356	80.62827	130.1091	105.4779
orange1.1t0	5.7712	5.574544	18.42356	10.92439	24.06218	15.21489	27.16444	37.03138	1.793799	2.040778
orange1.1t0	6.046983	4.792246	20.78376	10.72613	24.71156	12.99207	12.76283	19.31414	1.216879	1.154904
orange1.1t0	18.0419	18.35176	55.1019	37.2743	74.58481	52.02423	102.1214	145.6492	8.655967	11.91536
orange1.1t0	3.090167	3.773094	7.531338	6.539334	10.92305	7.89291	18.87199	23.62825	3.703901	15.04808
orange1.1t0	2.574365	4.528522	5.661151	7.734231	15.09462	10.40734	27.37788	45.03232	10.52832	29.97697
orange1.1t0	89.30847	88.93076	66.02642	72.76288	46.85207	72.20516	39.12362	101.6322	12.80984	38.4685
orange1.1t0	24.27283	24.54919	51.9566	41.52872	89.56688	78.68894	70.3844	112.3364	75.81499	66.48742
orange1.1t0	18.71827	29.8546	14.26793	17.95003	2.697184	3.419508	15.01974	68.46794	14.41891	90.50721
orange1.1t0	0.129029	0.421468	0.402146	0.376665	0.031758	0.274432	0.546439	1.077051	0.408533	2.758017
orange1.1t0	89.67559	116.3706	152.5216	163.5272	52.05013	81.63827	92.57067	126.4908	257.567	563.5889
orange1.1t0	9.356847	10.93651	10.17526	10.62258	8.025218	14.21219	3.929319	9.275779	11.93601	8.279575
orange1.1t0	9.481017	3.069008	4.976869	2.490123	9.506465	8.063609	7.02002	11.87543	14.76556	6.218352
orange1.1t0	8.421004	15.65791	4.477331	8.822692	2.074644	2.176026	0.621784	2.730629	0.437004	0.967523
orange1.1t0	259.4882	362.0846	209.0673	256.1157	372.9296	335.1352	521.4768	412.2022	1037.66	358.8901

orange1.1t0	21.83416	17.82524	37.61813	27.96486	48.47748	40.02953	90.95724	31.00989	23.14186	25.46715
orange1.1t0	89.95683	61.24412	13.97485	9.65613	15.26335	27.04865	17.30317	43.73191	7.976273	3.270027
orange1.1t0	35.04226	37.04963	46.81552	41.76562	166.4128	114.6748	280.1322	145.1418	560.8237	255.8838
orange1.1t0	37.5351	32.24999	40.27139	37.80663	54.69496	50.43888	101.8643	98.09345	51.71047	61.1348
orange1.1t0	0.866103	0.290026	0.543898	0.597987	0.256107	0.235689	0.239217	0.425984	0.020186	0.383574
orange1.1t0	5.99967	3.10835	1.727668	1.778784	1.372323	1.535444	1.465239	1.329851	0.326342	0.245119
orange1.1t0	3.773394	7.294346	3.214846	6.169353	1.60163	2.081449	0.941051	2.799856	1.231186	1.962341
orange1.1t0	0.084446	0.086261	0.293462	0.100506	0.131032	0.117137	0.337981	1.150245	0.049894	0
orange1.1t0	26.47061	41.79167	25.64454	31.94332	34.54585	33.78624	94.51689	164.9409	79.23293	145.6378
orange1.1t0	17.47835	20.06767	11.25294	11.813	8.56769	8.803934	2.815924	11.23883	2.722294	0.742934
orange1.1t0	72.9026	101.7973	65.72135	106.8594	24.98444	36.27666	13.53554	33.24474	29.22241	11.81605
orange1.1t0	254.6734	372.9982	142.5211	214.8986	54.55046	98.25823	14.90441	47.2438	3.717441	2.324162
orange1.1t0	8.851506	9.782923	6.971972	7.596862	3.905102	4.416206	2.797183	4.336902	1.273704	2.580454
orange1.1t0	7.428172	7.564589	4.66231	4.902047	2.822275	3.025093	1.591584	2.017232	0.697646	0.56256
orange1.1t0	13.9529	15.45595	19.77273	15.39851	26.31648	13.83499	9.862026	12.11489	2.676492	2.215602
orange1.1t0	5.07165	2.939082	5.849906	4.683049	3.746791	7.53983	0.502772	1.523071	0.268302	0.03564
orange1.1t0	5.150076	7.152553	5.935743	1.585242	10.38766	1.314405	1.513732	1.838398	0.751555	0.189189
orange1.1t0	9.573196	6.513887	10.27549	7.797157	0.860004	2.329916	0.113709	3.310125	0.057202	0.141342
orange1.1t0	39.15985	61.02855	73.78088	81.41488	52.12861	75.03532	16.17726	22.70045	4.650085	2.467561
orange1.1t0	7.402871	9.953836	10.63533	14.74741	8.986488	11.89323	4.744255	16.26413	7.97326	4.493337
orange1.1t0	26.08412	25.41975	14.60342	14.47669	4.191621	7.014484	3.942904	10.41714	6.456478	6.956683
orange1.1t0	0.456665	0.735833	2.154054	2.461784	1.581781	2.750811	0.693672	3.010105	2.656102	4.180722
orange1.1t0	0.369217	0.496252	0.637848	0.997862	1.868419	3.327473	0.37398	1.316627	0.221807	0.410274
orange1.1t0	9.626156	6.995024	9.26874	6.723395	20.60226	11.83882	18.92343	8.120253	2.699304	1.829403
orange1.1t0	0.156275	0.172961	0.106433	0.15443	0.190458	0.194574	0.079641	0.104738	5.972213	1.969874
orange1.1t0	9.477367	6.14232	3.264468	3.02132	2.512013	2.475546	1.487286	1.236103	0.114088	0.097684
orange1.1t0	4.2032	2.520047	3.18485	3.433259	1.554602	3.373945	0.846116	1.823684	0.491592	0.463315
orange1.1t0	8.692133	7.687586	7.982834	7.62803	3.210983	5.336725	2.36882	7.133451	5.949403	6.314423
orange1.1t0	17.17772	15.18942	12.26511	9.739137	7.318732	8.563137	4.291379	5.346803	6.398516	3.318984
orange1.1t0	10.0505	9.639017	20.23593	15.67651	12.6551	11.72264	3.84927	10.50329	4.456971	3.200258
orange1.1t0	1.830728	1.536691	1.911702	1.95182	4.056039	4.30037	2.510013	1.864304	3.03589	2.530249
orange1.1t0	9.178091	9.621326	8.243519	8.95562	5.708326	6.204614	2.973544	10.67338	4.506034	6.575534

orange1.1t0	2.329063	2.64067	4.42135	4.770857	1.773989	2.079014	2.46582	4.089513	7.334503	12.1117
orange1.1t0	3.649064	12.54509	0.244014	0.631939	0.059828	0.2905	0.067718	0.381846	0.103136	0.118723
orange1.1t0	2.727003	0.998135	6.977222	4.698562	13.79954	16.54954	4.599022	7.72702	2.961022	8.598833
orange1.1t0	54.20962	44.21104	33.67208	28.89416	23.75957	46.85425	19.25352	16.76072	33.26589	9.75197
orange1.1t0	0.486205	0.250502	0.2611	0.31668	0.213091	1.257811	0.105294	0.126645	0.163479	0.126887
orange1.1t0	1.902348	1.633776	1.673517	1.786709	3.852698	4.382166	8.102222	6.350295	5.811598	7.025143
orange1.1t0	114.9733	74.97175	217.8773	140.8178	309.4578	209.3759	338.8357	98.62516	434.7325	301.3679
orange1.1t0	17.03102	9.39783	26.98806	26.99371	34.65241	46.28798	13.70864	32.95946	25.75941	10.74038
orange1.1t0	2.540623	2.664809	2.94236	3.581698	3.233078	3.669709	1.538123	3.662278	2.242638	2.571944
orange1.1t0	14.80071	20.09354	16.64181	19.42008	13.0484	15.23022	12.41739	28.76331	18.38784	38.82308
orange1.1t0	18.98575	24.89221	7.648806	13.59023	2.149112	3.044573	2.037263	1.602644	0.023044	0.065125
orange1.1t0	20.06332	12.68865	18.16882	15.93067	49.54949	41.86392	71.0363	37.71442	133.297	87.52957
orange1.1t0	102.5028	116.0952	127.7785	132.0293	76.33337	134.0284	18.14616	60.78261	2.05487	6.163951
orange1.1t0	0.291186	0.762919	2.300194	2.989132	3.503248	6.294707	0.755323	2.249787	0.322667	0.63078
orange1.1t0	6.212103	12.33791	13.24163	16.99512	15.92839	10.10211	23.7487	46.5627	7.918294	31.96178
orange1.1t0	10.47027	13.22194	15.13843	17.28896	5.902028	9.317636	7.867419	20.17736	4.612976	8.437422
orange1.1t0	4.165025	12.0097	1.034032	2.949407	0.126451	1.073079	0.592452	14.35069	0.195148	3.693318
orange1.1t0	23.37501	27.47374	30.12428	26.54708	64.05633	41.83924	157.3188	107.66	107.1336	66.6821
orange1.1t0	8.505711	9.40101	38.25418	48.58661	88.83971	232.5294	58.03502	135.2218	486.0195	441.2484
orange1.1t0	0.420749	1.955151	1.34654	4.570635	0.046335	0.11191	1.114603	0.154188	0.069607	0.094552
orange1.1t0	25.44244	20.87876	28.54182	21.77525	81.95794	34.22856	303.0796	270.137	93.56523	61.10114
orange1.1t0	0.134983	0.134756	1.691902	1.326285	3.632335	7.646767	7.40856	8.90919	11.15626	5.877266
orange1.1t0	15.10346	14.90626	10.00038	10.74834	8.566903	7.502552	6.80727	6.654562	1.879702	2.962622
orange1.1t0	1.622095	2.156865	1.393207	1.529523	0.583483	1.032046	0.733419	1.994793	0.049504	0.077985
orange1.1t0	1.406927	2.217522	1.175401	1.459096	0.361602	2.411948	0.113559	0.620373	0.056203	0.058984
orange1.1t0	3.610582	1.665033	3.253877	3.108897	1.211271	3.073718	0.570672	0.313694	2.502795	0.631516
orange1.1t0	4.283846	4.166406	5.074937	5.037286	4.956802	4.521084	1.303664	4.492039	0.474625	0.962978
orange1.1t0	20.46724	21.21287	11.64233	12.27351	9.961749	9.006918	6.295743	8.495641	4.651428	7.310586
orange1.1t0	6.356112	5.373522	16.061	13.89401	15.04543	20.98575	4.670957	6.577225	2.940244	4.41622
orange1.1t0	4.404278	4.368262	8.976562	8.800888	5.307254	7.236883	2.111081	5.997257	0.577675	0.58325
orange1.1t0	25.41638	30.34706	50.76919	52.69953	60.30727	48.09288	12.91211	47.38781	78.94134	185.0947
orange1.1t0	1.300615	1.493834	1.557492	1.785584	1.026367	1.38136	0.801111	1.951758	2.392755	2.471735

orange1.1t0	2.913056	2.677487	6.302886	4.55023	5.879006	6.56397	4.292584	4.394201	0.90097	2.366871
orange1.1t0	1.029469	0.839231	1.41813	0.933009	0.940726	1.676093	0.913251	1.159272	1.330673	1.220523
orange1.1t0	9.134044	9.526095	10.35398	10.72792	12.78564	10.64177	7.065886	16.05501	8.120549	8.026992
orange1.1t0	1.258108	1.171193	1.891775	1.815195	1.345006	1.249639	0.626111	2.494549	0.282988	0.568499
orange1.1t0	44.45488	23.59725	29.7209	25.05731	45.75197	33.13687	53.75093	21.43644	53.91577	16.93434
orange1.1t0	0.347083	0.234671	0.957299	1.189958	0.694444	2.443016	0.994381	0.882947	1.189525	0.981454
orange1.1t0	0.539198	2.175951	0.314151	0.499416	0.109733	0.416463	2.395364	0.466865	0	0
orange1.1t0	12.54626	9.475141	11.32199	11.06921	10.66889	17.08328	12.22538	9.042177	8.842327	4.889725
orange1.1t0	6.839774	5.995936	9.943142	5.282063	39.55554	14.13279	27.60511	8.925467	14.92859	14.42708
orange1.1t0	5.84261	12.58299	18.53223	20.88396	8.867904	12.14968	10.7803	32.40759	14.33415	28.72532
orange1.1t0	39.04974	46.63866	47.89231	48.26649	50.87656	41.86242	114.0298	231.6652	55.12463	78.01188
orange1.1t0	3.156844	4.061799	4.302619	4.666178	2.346931	5.618664	1.804443	11.85005	5.763187	10.02532
orange1.1t0	0.544499	0.896013	1.277302	0.754393	2.067978	1.347373	3.117772	1.859939	0.392872	0.416382
orange1.1t0	14.0756	16.20365	11.66449	11.17872	10.04693	9.947767	3.418745	7.12716	9.363932	5.991335
orange1.1t0	5.689178	4.541836	1.89422	2.02732	1.659918	1.444823	1.860787	2.486493	1.552186	1.858509
orange1.1t0	7.431369	5.664851	2.645923	2.806151	0.812457	1.463398	0.975615	1.337622	0.257371	0.126755
orange1.1t0	3.479287	3.750428	5.023298	4.125406	1.79421	3.071903	1.474885	5.357952	0.048736	0.078927
orange1.1t0	0.326416	0.569522	0.793423	0.31847	0.083281	0.171134	0	0.067444	0	0
orange1.1t0	63.66888	52.22308	19.66629	24.01821	6.397157	7.614283	3.31522	3.432038	1.447354	0.459807
orange1.1t0	20.16832	29.14588	9.544288	8.921972	3.331411	4.265859	4.873334	8.273722	3.25695	6.68037
orange1.1t0	107.7224	86.28396	66.73887	54.74854	114.9963	30.06053	27.5846	31.58559	4.672104	3.753896
orange1.1t0	0	0.060602	0.674039	1.900676	0.233544	5.249247	0.453551	2.507811	0	0
orange1.1t0	0	0.0319	0.750387	0.810952	0.361116	3.236317	0.529818	1.779843	0	0
orange1.1t0	3.354808	3.19167	4.526343	4.82378	3.125016	5.62286	1.298461	1.555183	1.3317	0.680643
orange1.1t0	17.40635	15.83728	22.75573	21.70584	13.50026	25.1851	3.878398	5.483411	1.372202	0.938152
orange1.1t0	18.196	13.9232	18.35574	18.24292	12.9375	25.71958	6.458267	6.605882	10.55925	5.455707
orange1.1t0	137.2281	134.0496	30.19134	37.47348	10.91736	23.54737	13.31448	30.78884	6.098534	3.406056
orange1.1t0	16.42677	23.74442	16.46986	19.56331	17.22111	10.02017	17.12247	21.59964	14.05044	15.45179
orange1.1t0	0.788591	0.727428	0.771528	1.101688	0.347967	1.970398	0.47968	2.881222	3.396838	7.04509
orange1.1t0	22.56032	27.92723	22.82307	23.84302	13.22506	15.63991	6.882302	11.47203	10.61452	13.84692
orange1.1t0	63.82882	59.24333	43.71137	40.77347	14.19598	13.48512	5.807184	7.145387	13.92163	7.539969
orange1.1t0	22.18445	28.25195	15.58855	26.96744	5.14286	12.26805	6.905955	10.20131	50.39479	89.64309

orange1.1t0	3.015828	3.461127	3.77363	3.657576	4.869603	4.167311	2.742441	6.596511	3.661954	4.609595
orange1.1t0	4.935723	7.991725	16.85038	17.9207	50.34064	45.394	134.1779	109.8869	104.3394	104.1246
orange1.1t0	3.966819	4.769656	13.80883	15.99791	29.80496	42.43876	88.92595	42.14551	12.14846	8.182382
orange1.1t0	6.519856	5.503759	35.00414	38.06885	49.89538	30.03185	51.27707	111.2914	30.1299	69.24692
orange1.1t0	30.45392	17.64991	27.55167	16.66506	45.62241	20.21934	14.43106	11.96188	10.10865	2.678713
orange1.1t0	64.74214	23.00169	30.8493	44.82502	7.515028	19.97759	8.392791	12.75936	17.42509	26.98565
orange1.1t0	11.89169	13.3418	10.61356	10.97078	7.190544	6.411447	3.182481	4.475079	2.085682	3.589264
orange1.1t0	2.684051	3.559558	1.106732	1.643289	1.166985	1.303958	1.281131	1.30769	1.459424	2.571119
orange1.1t0	8.305767	12.93994	3.574697	5.924997	2.66642	3.210599	2.62411	4.42692	4.751573	6.627495
orange1.1t0	20.68018	21.04608	16.12901	32.96169	5.365993	14.61152	16.05421	20.53104	11.56557	15.58685
orange1.1t0	18.46884	17.53517	14.2275	28.62583	4.740237	12.35242	15.51665	16.1397	9.959428	12.64962
orange1.1t0	41.65038	30.34404	23.49365	22.74516	13.44654	15.51442	10.78262	9.90305	6.6341	4.647903
orange1.1t0	25.62064	30.74202	18.55895	12.78861	5.754264	5.413954	0.9548	3.76602	0.284918	1.529095
orange1.1t0	76.97748	84.26127	76.29725	77.56256	50.52175	49.67242	24.59165	65.00423	30.04707	40.6166
orange1.1t0	0.780605	0.887008	1.368044	0.709395	1.083871	0.981672	1.163932	0.843151	1.767675	0.773108
orange1.1t0	0.798176	0.611674	16.61556	12.11857	75.61502	56.43609	8.919545	8.921525	7.139944	15.66634
orange1.1t0	0.232061	0.247177	0.399466	0.443339	0.314362	0.693779	0.233895	0.371004	3.044429	0.617392
orange1.1t0	6.363543	6.703905	6.0035	4.585244	2.230228	2.866992	1.867375	1.569355	0.579213	0.167022
orange1.1t0	9.72157	4.566169	15.01838	9.054013	13.73811	12.90467	3.48957	2.715544	0.603542	0.366125
orange1.1t0	2.101271	2.134635	2.841729	4.044535	7.409039	4.739423	0.9098	3.848261	1.658354	2.058022
orange1.1t0	4.280396	3.363814	3.720727	2.887969	3.279447	2.978655	2.842622	4.71063	0.926319	3.498095
orange1.1t0	0.911915	0.55284	0.696999	0.360501	0.835257	0.45406	0.626617	0.45729	0.192375	0.329385
orange1.1t0	6.814269	8.964903	7.019684	8.637231	6.706672	6.189702	2.576996	5.99917	4.449829	5.011418
orange1.1t0	61.17727	37.09921	35.72105	28.06346	40.32217	57.21321	14.28423	8.528034	24.35502	3.624917
orange1.1t0	10.55016	10.9219	11.68412	11.81878	8.671188	11.51733	6.484095	15.33598	22.42011	24.75991
orange1.1t0	2.030851	1.470144	0.841252	0.643329	0.053166	0.258927	0.084192	1.690507	0.131153	0.185944
orange1.1t0	19.32804	20.62905	12.80397	12.67624	10.10318	10.89479	5.267762	9.524766	3.882143	5.232435
orange1.1t0	3.912922	1.109014	5.029742	8.060064	2.794271	15.9359	0.1946	0.054415	0.825219	0.208757
orange1.1t0	20.18301	19.66684	26.90403	37.05055	23.56836	31.97739	53.63292	31.70221	16.44796	80.13995
orange1.1t0	1.7699	2.506728	2.164763	2.294783	2.362992	2.815863	1.722679	5.11928	2.107445	2.040469
orange1.1t0	5.509553	6.414277	3.238615	3.66216	3.212987	3.260543	2.864277	3.54868	1.379114	2.582101
orange1.1t0	0.331367	0.370522	0.628104	0.441551	0.742129	1.128337	1.056782	1.113079	1.054133	1.61268

orange1.1t0	15.31011	13.51449	5.895402	6.34649	1.557525	2.271234	4.420319	5.340012	0.442287	0.701307
orange1.1t0	9.293029	11.19265	16.62037	15.26331	16.79706	18.06547	9.658911	22.2418	8.900256	15.20346
orange1.1t0	0.488806	0.570639	1.316153	1.13681	0.774602	0.61165	0.236887	0.849426	0.225699	0.4552
orange1.1t0	4.432493	6.436208	3.398853	2.901596	0.852096	1.144672	0.408425	1.031355	0.101711	0.103442
orange1.1t0	2.457146	0.894785	1.251536	1.104683	1.588767	1.704568	0.727177	0.405681	0.198329	0.0527
orange1.1t0	0.400051	0.361241	1.647853	1.206713	0.883517	0.598233	0.426996	1.844748	4.053955	2.480093
orange1.1t0	7.528835	9.51801	1.684968	1.323307	3.282098	7.074815	23.317	19.61029	43.93071	83.207
orange1.1t0	0.568366	0.786926	1.017798	0.967603	0.744832	0.644896	0.503008	1.076029	0.592204	1.312088
orange1.1t0	0.393404	1.108715	5.726445	5.987215	31.05381	23.09388	42.03278	23.48315	469.6059	236.5479
orange1.1t0	0.315756	0.843027	3.694963	4.250331	21.17885	16.42674	27.32605	16.7259	334.2785	181.1999
orange1.1t0	255.5239	408.1132	122.1889	142.0297	54.58741	60.0773	56.57077	143.8589	26.68955	69.04114
orange1.1t0	7.886184	7.64948	9.692071	11.77705	27.00194	15.50915	41.2778	55.78396	252.8301	584.1019
orange1.1t0	8.723592	13.85656	8.913348	12.03897	3.26253	2.536771	1.428357	2.33287	0.426668	0.663398
orange1.1t0	14.60868	20.5807	14.76035	20.02736	4.731782	3.79312	1.948343	3.474901	0.578276	0.898807
orange1.1t0	1.318365	1.794668	3.546514	3.369247	12.70797	11.0429	28.33909	23.69528	20.38818	26.06888
orange1.1t0	1.755625	1.937439	0.860276	0.655065	1.157116	0.921663	2.433004	2.240292	1.6339	2.179716
orange1.1t0	1.071884	1.351424	1.676329	1.555026	1.02587	1.860776	2.346097	4.339208	3.43985	7.521222
orange1.1t0	18.56682	21.49585	20.06034	13.69926	15.19059	8.39422	1.681193	2.029642	0	0
orange1.1t0	29.88531	32.11367	35.89588	46.75783	28.45752	48.03765	94.44453	212.3303	195.0654	383.477
orange1.1t0	4.803695	8.250908	5.712955	6.062488	14.30514	28.48514	26.99342	41.42748	132.897	203.5819
orange1.1t0	1.378718	5.168085	0.323812	1.035124	7.316211	7.729713	7.562484	19.08224	34.14042	51.14308
orange1.1t0	37.96813	39.42976	8.227957	14.2744	10.01532	13.55512	9.020811	19.95157	4.678106	5.830119
orange1.1t0	29.61917	34.73213	16.35678	27.2713	2.205579	3.305607	0.465873	1.505938	0.299964	0.126909
orange1.1t0	7.163457	5.509391	28.3383	17.11175	47.48867	45.18569	100.5969	44.34584	23.7577	23.95419
orange1.1t0	0.162819	0.165919	0.640242	0.562215	0.535567	1.076498	0.130454	0.725742	0.064348	0.103928
orange1.1t0	0.736063	0.714167	0.479116	0.583971	0.340299	0.814561	0.066296	0.209245	0	0
orange1.1t0	17.07902	17.81399	31.39741	41.90091	5.593361	12.32	8.198743	29.09501	31.11606	85.62212
orange1.1t0	0.793615	1.19263	1.037549	1.509572	1.427164	1.128742	0.663757	2.489845	1.495261	1.793
orange1.1t0	2.134876	1.158126	1.213614	1.280847	1.75647	6.770475	0.433654	0.892849	1.356262	0.436822
orange1.1t0	0.511968	0.308666	0.255237	0.210826	0.193685	1.370987	0	0.031908	0.145945	0.050592
orange1.1t0	0.619711	0.481541	0.758075	0.911118	0.877477	2.887855	0.069221	0.545325	0.417306	0.052123
orange1.1t0	0.651461	0.494064	0.62636	1.140293	0.579323	2.578796	0.180418	0.627936	0.657555	0.325844

orange1.1t0:	1.12029	0.687869	1.348726	1.636756	1.388405	4.309436	0.286736	1.39842	0.919547	0.30035
orange1.1t0:	2.379454	1.793852	3.642015	4.096888	1.424391	2.581723	0.412716	2.164328	1.085328	1.157051
orange1.1t0:	0.357688	0.408254	0.447701	0.480711	0.590145	0.595398	0.21404	0.9624	0.339183	0.518238
orange1.1t0:	1.722158	0.887107	1.253582	1.638361	2.659941	3.896763	3.646475	1.496792	5.371934	1.818906
orange1.1t0:	1.445927	2.054741	4.617821	6.47337	4.335854	5.683318	5.678408	22.83553	1.520643	5.330506
orange1.1t0:	47.39516	61.30497	175.8218	147.5872	176.1051	94.35815	105.9735	197.5253	10.85581	40.53358
orange1.1t0:	12.17174	19.43114	9.818308	15.26673	13.44781	10.78592	8.16914	21.27219	5.310312	9.385168
orange1.1t0:	2.29936	3.805728	7.864207	7.134543	13.97456	10.58777	3.721138	7.575123	0.174442	0.360453
orange1.1t0:	21.48679	21.05487	13.87042	12.25035	7.371465	8.791185	5.464003	6.252024	3.246706	4.604216
orange1.1t0:	1.319128	1.192303	1.848295	1.478103	1.187359	1.267986	0.674235	0.883455	1.015646	0.336254
orange1.1t0:	3.050913	3.492506	12.87727	6.742033	19.43486	16.68628	1.207683	1.200435	0.011631	0.022057
orange1.1t0:	1.553534	0.690392	4.269348	2.234819	10.26619	9.371932	4.475541	3.878484	3.763769	4.996254
orange1.1t0:	1.036961	0.785647	2.775526	2.912466	3.826929	4.18942	2.242592	4.869991	0.717913	0.658243
orange1.1t0:	0.567179	0.267837	1.639699	1.221064	2.66728	3.201173	3.172528	3.209211	0.787167	0.439219
orange1.1t0:	1.835349	0.707117	6.460311	3.609508	16.48858	11.33144	13.17181	9.055607	0.409488	0.449484
orange1.1t0:	30.07758	51.21957	93.99029	130.1367	6.990226	14.78057	17.08225	79.80404	2.274814	26.94468
orange1.1t0:	42.85972	36.93041	62.13014	42.87008	60.52806	31.57145	44.18773	39.34298	26.38064	21.34243
orange1.1t0:	5.557054	5.12389	14.76917	8.342681	26.6736	38.24775	38.39312	24.16314	0.907021	0.574011
orange1.1t0:	5.799834	6.773561	58.51933	40.52696	176.2558	236.6764	403.077	465.8363	127.1023	118.9113
orange1.1t0:	39.7483	36.89141	33.35126	31.15671	30.29038	31.55626	46.64244	20.67235	47.45442	42.92154
orange1.1t0:	0.446532	0.495113	0.21956	0.151201	0.143481	0.231289	0.317507	0.192016	0.133865	3.489535
orange1.1t0:	11.90625	15.62247	19.14098	14.65084	31.78676	17.99083	26.87902	16.42833	25.14711	6.839479
orange1.1t0:	5.582231	4.035759	0.103089	0.100115	0	0.085107	0.135355	1.460667	0.104876	0.529172
orange1.1t0:	25.36144	28.15689	4.305658	5.046945	1.86771	3.06723	2.259437	8.608479	0.203053	0.126618
orange1.1t0:	4.702146	8.305822	2.928118	2.939404	1.145274	1.807283	0.154427	1.525812	0.362771	0.486062
orange1.1t0:	6.405876	6.770646	3.455497	4.334572	3.402874	2.826646	1.465975	1.633551	1.341517	0.727484
orange1.1t0:	10.38865	12.35181	8.279147	8.195149	6.483527	6.490528	2.488739	4.178234	1.705728	1.66773
orange1.1t0:	43.21631	45.90951	77.09181	68.31566	110.3405	103.2563	118.8321	144.5095	110.5621	134.3945
orange1.1t0:	14.79781	12.27141	11.73549	9.481075	20.35591	12.79466	17.26775	6.486589	1.511875	0.654003
orange1.1t0:	2.329328	1.206856	0.072375	0.377956	0	0.001739	0.004607	0	0.008819	0.008251
orange1.1t0:	20.58604	17.28872	24.50745	29.35454	55.62999	79.98118	115.5564	109.8065	84.36584	54.59719
orange1.1t0:	0.638436	0.385047	2.045465	3.798017	2.534637	2.991307	10.91966	3.745266	25.67127	11.59138

orange1.1t0.	65.05277	67.4424	126.8634	122.1442	213.0785	172.3952	397.4478	167.8601	216.9416	144.2765
orange1.1t0.	0.626784	1.012294	1.188384	0.927597	3.969553	6.183078	6.27338	3.205833	0	0
orange1.1t0.	59.23461	92.55081	29.75574	48.50724	18.3235	17.13921	23.84037	25.84619	10.69615	11.32965
orange1.1t0.	125.0533	105.8473	202.1355	177.6832	354.839	273.9045	434.6371	288.7878	255.8311	175.6432
orange1.1t0.	16.35091	12.77392	16.17407	15.41488	20.32145	20.96316	22.06974	57.31359	1.079235	4.40816
orange1.1t0.	10.8069	13.13503	4.420505	6.488813	2.023571	2.512716	1.516356	1.774628	0.354293	0.186793
orange1.1t0.	4.892638	3.59956	3.877724	3.188002	4.986557	4.110128	4.822537	4.121994	1.486758	3.189925
orange1.1t0.	1.053189	0.997493	0.71705	0.651232	5.306106	1.543826	1.416306	0.319385	0.061277	0.833903
orange1.1t0.	2.38406	1.23102	1.860258	2.081208	0.739342	1.839308	0.292357	0.207776	0.598456	0.180059
orange1.1t0.	46.42123	29.39935	57.56427	40.46468	35.01776	31.62148	3.008811	9.664381	0.724249	0.172015
orange1.1t0.	20.57639	24.80373	15.9795	18.21959	8.22112	9.688109	4.955999	11.93779	5.809449	7.838906
orange1.1t0.	3.425605	2.093711	2.207786	2.122438	5.46535	5.405804	4.973937	2.005188	2.283591	2.44735
orange1.1t0.	5.315552	3.838556	3.476922	3.326579	8.546454	8.156777	9.975296	2.76691	3.858391	4.286121
orange1.1t0.	3.06926	3.481287	2.48474	2.696341	1.912316	2.131239	2.104299	4.838259	0.725323	1.818298
orange1.1t0.	4.891024	2.89685	0.482172	0.480018	0.176862	0.173993	1.469606	1.264307	0.882226	0.505722
orange1.1t0.	0.004362	0.006718	0.026033	0.019934	1.50425	1.806503	3.635015	1.439389	2.042595	1.949612
orange1.1t0.	4.570613	6.045693	3.101519	3.683756	1.632864	2.677303	1.368927	3.65967	1.028101	1.786653
orange1.1t0.	3.357264	3.458523	3.43623	4.127956	1.830516	2.506584	0.409897	2.222416	0.040584	0
orange1.1t0.	1.89105	5.476238	4.038005	4.701505	9.297192	5.356748	18.55405	8.183421	2.024395	2.632929
orange1.1t0.	10.61896	7.874948	18.50688	19.4234	6.800161	16.46182	3.384816	7.045993	4.522321	8.387813
orange1.1t0.	17.36158	16.48906	35.56062	33.04459	20.06129	42.4876	7.94549	20.43679	22.30422	41.89083
orange1.1t0.	43.59157	82.65254	20.3569	36.52517	22.45708	18.60084	27.59333	43.93561	15.47729	16.28812
orange1.1t0.	5.60259	5.749381	5.245726	5.659409	3.547639	4.290095	3.693399	3.372335	4.057339	1.546553
orange1.1t0.	14.35686	12.32185	59.71113	46.24953	57.76684	44.03015	26.3222	49.10406	28.40197	27.41012
orange1.1t0.	8.328824	11.06277	6.972301	8.113972	24.42375	23.92138	48.9827	27.12912	16.33523	17.71071
orange1.1t0.	3.964385	8.513738	2.334814	3.586438	11.90608	4.587866	8.706632	18.59693	25.06135	13.30792
orange1.1t0.	1.703418	2.593114	10.63268	15.73676	12.42805	13.39404	5.091145	30.95098	7.325811	14.66458
orange1.1t0.	19.11394	43.24708	31.99136	25.21157	25.32233	29.2272	38.46469	16.34181	19.78498	22.76357
orange1.1t0.	10.00573	13.44358	37.2162	24.78614	95.83499	112.6569	52.92674	81.76042	10.0323	3.639913
orange1.1t0.	0.047534	0.018149	0.213487	0.240109	1.035376	0.527469	1.830758	0.817012	3.089105	1.062935
orange1.1t0.	42.98745	34.56296	93.47317	76.05293	205.2614	180.6828	330.8445	176.1298	194.5775	151.7615
orange1.1t0.	18.0388	15.68177	23.36557	18.35421	39.54832	30.14338	58.62598	23.45529	27.5033	27.1631

orange1.1t0:	14.36994	11.4823	27.53796	21.15032	31.4798	42.87812	74.20424	67.25074	24.10841	37.22042
orange1.1t0:	13.15313	10.24117	19.9328	21.9648	23.8582	48.61995	31.68725	30.52387	29.22894	24.06705
orange1.1t0:	0.710683	1.283723	0.496566	0.455275	0.011302	0.10733	0.017631	0.059595	0.665885	0.887178
orange1.1t0:	557.492	612.293	186.2591	283.5783	91.37904	104.1948	224.7486	235.0661	402.1305	452.2498
orange1.1t0:	24.51821	30.95574	26.47861	27.7028	21.14873	9.883	21.492	29.81111	4.987842	11.01159
orange1.1t0:	0.267037	0.492809	2.073521	1.913839	2.884458	3.224819	4.105134	6.104748	6.473663	13.06277
orange1.1t0:	33.8021	101.7748	15.40185	13.05697	11.25511	15.14418	3.832842	16.95357	2.46169	3.329043
orange1.1t0:	0.116184	0.200123	0.136243	0.184984	2.435949	0.881964	1.092148	0.348929	0.316526	0.107558
orange1.1t0:	0.573806	0.690943	1.056504	0.915153	0.159613	0.234729	0.235351	0.683797	0.317299	1.089669
orange1.1t0:	0.145177	0.079963	1.583603	0.74943	0.477212	1.018326	0.193005	0.218883	0.090595	0.098639
orange1.1t0:	0.649766	0.468263	1.746413	1.167506	2.338552	2.204513	2.333017	2.206026	2.591542	2.067181
orange1.1t0:	0.163557	0.152651	0.895322	0.674837	0.878711	1.225773	0.663234	1.524702	0.831879	1.120442
orange1.1t0:	0.274743	0.209646	0.525727	1.236572	1.957367	1.830937	1.638453	2.254622	2.530208	3.094194
orange1.1t0:	10.71698	8.835314	16.0846	13.97534	22.78387	25.14265	33.80297	25.57909	48.5453	34.67946
orange1.1t0:	1.956819	2.519494	2.00606	1.775686	0.815187	0.823291	0.756013	1.686964	0.270268	0.693558
orange1.1t0:	33.41694	34.71052	8.305166	12.11246	4.473468	9.151176	0.581724	1.292145	0.207677	0.230646
orange1.1t0:	4.39534	5.020793	6.179932	5.959179	6.354106	6.681555	3.291786	8.354672	3.600781	4.196616
orange1.1t0:	3.981558	4.316103	5.338307	4.661573	5.632611	6.071863	2.737042	7.155114	4.858123	6.910485
orange1.1t0:	0.046406	0.123316	1.63295	1.459311	2.845588	4.320584	1.608412	2.179758	1.596772	1.382991
orange1.1t0:	9.669273	14.59565	33.25186	27.79143	115.5528	105.0785	28.83182	59.42739	41.39199	40.23082
orange1.1t0:	0.24868	0.476838	4.460493	3.810417	9.391922	12.08435	3.260292	5.025618	3.512793	2.193128
orange1.1t0:	3.741914	5.711821	12.08264	10.93388	38.18127	38.62964	12.43291	17.81642	12.97679	15.88386
orange1.1t0:	11.92071	18.47653	41.94104	33.06642	122.4556	107.0999	37.40338	50.4172	39.96457	39.54464
orange1.1t0:	47.73903	61.77653	131.601	103.4253	113.3661	119.8614	19.75464	45.88069	26.4307	17.38289
orange1.1t0:	13.22831	19.75614	38.52656	33.43455	30.2813	39.27366	3.981947	11.17613	10.64271	4.209037
orange1.1t0:	15.1596	14.46117	29.29465	21.19646	17.98047	20.4809	3.944492	9.305992	6.92064	4.389246
orange1.1t0:	13.63456	15.65879	11.2346	8.502575	4.656439	6.279538	0.921844	1.973922	1.226843	0.619917
orange1.1t0:	1.927113	1.671606	3.519558	6.571908	0.465097	1.356187	0.029534	0.569744	0.023878	0.06381
orange1.1t0:	5.555967	6.941608	9.218955	10.78295	32.33922	30.25924	33.4828	35.36633	120.067	70.39376
orange1.1t0:	9.404034	10.56868	12.92747	14.49361	27.72869	28.43892	48.40203	36.42333	102.8246	74.82133
orange1.1t0:	2.064151	2.185418	3.717818	4.141035	1.521586	2.818124	1.063128	2.906758	2.352656	2.602529
orange1.1t0:	0.279544	1.077422	1.217843	1.326014	1.021398	0.800519	1.50278	4.22033	67.02547	53.26974

orange1.1t0.	1.093735	1.070938	1.662537	1.661265	2.271876	2.191201	0.99067	3.20604	1.814787	1.914719
orange1.1t0.	0.578202	0.71956	2.440131	1.31751	2.007489	2.090407	1.615298	4.485662	1.989156	2.470384
orange1.1t0.	1.887843	2.291442	3.676514	2.895691	4.426836	2.308552	6.94356	10.10492	27.98812	23.36771
orange1.1t0.	3.468202	4.444516	5.044613	4.787922	1.629118	1.947902	3.366617	3.386721	1.626331	2.912334
orange1.1t0.	0.673411	0.712363	0.852002	0.954419	0.451716	0.814965	0.276029	0.466695	0.722977	0.691236
orange1.1t0.	2.156308	2.003071	3.512026	2.348419	0.868813	0.712484	2.058642	1.623646	1.247549	0.844727
orange1.1t0.	1.716028	1.934266	1.534955	1.738604	1.33466	1.749844	0.75582	1.764044	1.849857	1.443865
orange1.1t0.	2.837023	4.088397	1.246116	2.189129	1.766862	1.751361	1.764941	2.061137	1.160203	1.630501
orange1.1t0.	385.689	632.6166	79.16624	148.3092	47.61358	42.26665	34.47236	59.77545	100.1219	70.66987
orange1.1t0.	77.65854	79.0776	40.71896	78.83789	46.14732	34.4223	31.25957	50.79264	113.3229	192.0671
orange1.1t0.	12.46293	10.03173	6.856667	5.41406	5.697309	3.707361	6.103181	9.842267	0.998523	0.246894
orange1.1t0.	4.991005	2.406675	9.85722	7.905331	14.01188	12.87139	11.49196	9.550528	9.833502	5.772575
orange1.1t0.	6.966203	9.777663	6.301721	8.038571	7.053762	6.410959	3.072492	8.23078	3.062541	3.78101
orange1.1t0.	0.507486	0.330206	0.822952	0.715914	0.097043	0.219627	1.107247	1.855499	0.650659	2.081132
orange1.1t0.	18.20709	22.86659	30.98292	28.01856	24.45183	35.0214	8.66072	13.48572	26.73298	5.799791
orange1.1t0.	4.156548	4.701728	4.743024	4.682987	4.862091	6.462625	1.997014	5.37923	4.810623	6.00614
orange1.1t0.	6.328777	5.081077	3.954586	4.419802	18.92111	9.359644	58.50089	20.71342	24.14745	20.8569
orange1.1t0.	2.462153	4.328567	6.197142	5.905485	12.17394	9.25999	3.533507	5.983832	0.137481	0.299714
orange1.1t0.	5.889303	6.923358	11.40688	13.72904	3.895163	10.63297	3.633863	17.7878	9.445058	13.02796
orange1.1t0.	0.575246	0.917573	0.355695	0.818177	0.578256	0.200239	0	0.126646	0	0
orange1.1t0.	0.694116	0.779202	3.795269	3.348059	2.489068	1.021117	2.381817	4.273851	8.05117	11.04197
orange1.1t0.	0.710941	1.294121	1.160919	2.06352	3.013989	1.002595	1.470693	3.97711	0.904528	2.519887
orange1.1t0.	0.754358	0.316776	0.622918	0.420196	0.355441	0.42704	0.148546	0.146958	0.178271	0.090483
orange1.1t0.	22.33733	32.50266	14.38874	25.41845	10.2411	11.6138	10.938	21.82972	3.300204	6.413764
orange1.1t0.	4.430348	4.127587	4.537505	5.687669	3.857658	4.884819	2.877902	6.481614	2.296174	4.91259
orange1.1t0.	0.57396	1.283485	0.377694	0.51679	0.17556	0.477965	0.111553	0.161696	0.161843	0.208781
orange1.1t0.	9.426024	10.81486	10.44387	15.06055	5.003787	7.735332	9.554564	11.23392	2.737961	7.348158
orange1.1t0.	323.786	411.8608	174.6795	806.7277	236.0472	333.3831	70.58369	72.90022	2.714749	6.305424
orange1.1t0.	39.45938	40.23254	90.29353	128.3749	101.311	68.88207	62.07499	42.4687	7.607716	2.667216
orange1.1t0.	22.33301	18.9218	28.36628	26.664	18.56872	20.37185	19.44964	19.82272	3.601215	15.60823
orange1.1t0.	0.409356	0.51489	0.77733	0.814117	0.494198	0.569161	0.726506	1.889765	0.692695	2.00187
orange1.1t0.	4.360698	3.723716	10.35287	13.79437	16.78729	30.21495	11.86161	20.2154	34.94108	44.47141

orange1.1t0	22.92731	18.10608	118.2838	34.57174	133.4243	88.31966	4.508323	2.834791	0.44795	0.250226
orange1.1t0	62.69155	36.1269	17.70684	16.32388	5.066477	9.689766	8.665541	6.647363	7.017274	2.197907
orange1.1t0	26.20226	20.78371	12.54409	11.42246	2.853919	3.22824	2.236605	2.831171	0.049197	0.033088
orange1.1t0	5.172386	8.292678	0.997263	2.608924	1.013792	1.022519	0.702917	1.600717	0.119183	0.317646
orange1.1t0	8.154554	5.94531	22.35628	15.60289	12.8783	17.79981	0.98199	2.724869	0.198824	0.142224
orange1.1t0	9.935652	7.200459	18.62289	15.26435	26.76341	21.65605	42.05016	17.39976	15.01785	19.38207
orange1.1t0	12.54182	12.73163	15.40382	14.37478	13.94955	19.44519	7.39374	17.94687	14.24035	12.40772
orange1.1t0	3.539851	4.120689	3.352926	3.989592	0.703649	0.967847	0.802347	2.164681	1.397046	1.953371
orange1.1t0	0.858179	2.440065	2.150296	2.729515	0.184214	2.495236	0.036572	2.89838	0	0
orange1.1t0	2.136488	1.872512	3.233161	4.131422	0.185573	1.063532	0.137906	0.150373	0.056026	0.034411
orange1.1t0	1.633893	1.834984	1.9719	3.157846	3.314553	3.069886	1.397211	3.647164	1.78985	2.427206
orange1.1t0	25.52574	43.11179	28.64094	32.61041	21.58412	16.95458	10.80815	24.84996	14.6707	22.20352
orange1.1t0	38.30626	22.85175	11.73881	17.1641	9.907161	9.457905	10.51992	26.68865	4.859212	14.72858
orange1.1t0	0.901664	0.695669	1.455051	1.39729	1.358446	1.948742	0.248957	1.42852	0.671957	0.210153
orange1.1t0	3.893497	4.014424	3.26333	3.465283	2.417232	3.963909	1.642647	4.704367	0.517171	0.647682
orange1.1t0	0.733437	0.634013	0.788942	0.804879	0.413545	0.703289	0.36642	0.739986	0.320549	0.792261
orange1.1t0	153.5553	408.0587	69.17825	118.4791	76.58863	60.69602	79.35349	150.5973	9.52887	21.69448
orange1.1t0	0.653854	0.35674	2.027235	2.884802	1.813307	5.672958	2.1974	2.039365	3.455135	3.212289
orange1.1t0	0.583588	0.589128	0.400521	0.394536	0.149005	0.194944	0.009464	0.107332	1.540483	0.490468
orange1.1t0	0.082075	0.073207	0.06	0.044095	0.027878	0.031387	0.030531	0.025546	1.783887	0.36654
orange1.1t0	0.156909	0.139485	0.195272	0.219952	0.272532	0.604198	0.03938	0.134137	1.00797	0.181329
orange1.1t0	0.732359	1.720203	0.683723	0.853297	0.777454	1.052009	1.480725	2.423868	0.681991	1.299802
orange1.1t0	80.59241	70.26458	41.69023	42.2181	4.279425	7.9108	0.524552	2.111157	1.644734	3.394094
orange1.1t0	2.187206	0.954383	2.485897	1.255548	2.276719	2.036699	2.489053	1.640288	2.055038	1.009322
orange1.1t0	21.75541	14.83463	19.04012	17.29792	26.03419	25.32092	89.30883	37.49326	45.69512	20.29348
orange1.1t0	20.15797	14.1168	16.6095	16.03595	24.56513	24.85417	88.32578	40.43351	53.57767	22.52606
orange1.1t0	6.835246	6.786629	9.720641	9.930642	5.647234	9.3914	4.966105	11.11677	4.633446	3.807514
orange1.1t0	0.258205	0.451021	0.315967	0.322915	0.345899	0.152589	0.314586	0.799843	0.936136	0.626543
orange1.1t0	0.786847	0.787252	1.177308	1.39957	3.329626	4.52642	3.351951	4.541685	7.393475	11.23246
orange1.1t0	23.67083	24.66938	15.20995	22.24927	7.859815	10.85789	18.64656	28.94973	16.40143	52.95851
orange1.1t0	3.865899	3.352911	1.980002	1.785536	1.212593	1.370721	0.537227	2.239401	0.280521	0.083813
orange1.1t0	20.10705	35.61785	15.73676	14.88304	3.279891	5.017593	6.690659	13.58378	5.928339	9.132338

orange1.1t0	11.03847	13.54429	12.34193	13.2657	19.01191	10.11144	17.2036	14.44084	1.5384	2.865507
orange1.1t0	17.14185	10.86641	6.280928	5.726066	2.473758	4.626817	1.750165	3.541861	7.522154	6.497654
orange1.1t0	7.695065	8.129241	10.68546	10.33133	9.093955	12.23049	3.80102	8.937258	5.512066	5.647352
orange1.1t0	25.78546	13.6305	21.20792	14.45613	30.11867	23.44813	14.92001	13.25635	43.62269	14.89376
orange1.1t0	3.904303	9.533678	2.784773	4.700181	2.192617	2.305661	5.949666	7.713589	0.584144	1.029383
orange1.1t0	20.59429	26.14623	24.09826	25.22106	17.25965	20.1816	9.131856	21.94282	12.47825	16.98424
orange1.1t0	13.68954	10.48754	15.93173	14.5075	21.62836	7.924947	24.29185	12.01389	2.650248	3.524463
orange1.1t0	13.99107	28.56957	4.323651	11.27818	7.023514	5.374261	6.933243	13.43563	2.884046	2.29272
orange1.1t0	0.343688	1.010319	0.350305	3.203169	0.713725	1.433702	0.120797	0.306026	0	0.022512
orange1.1t0	0.252159	0.612505	0.206426	1.892214	0.527751	0.755527	0.064229	0.198609	0	0
orange1.1t0	6.896267	5.689095	4.834758	4.324232	1.854386	2.286455	1.187159	1.216629	0.431271	0.235034
orange1.1t0	8.76024	11.31883	6.764418	11.01198	10.03882	22.1734	43.91006	25.92571	26.30493	7.155899
orange1.1t0	5.140802	6.457553	3.128358	6.057737	4.432828	9.766729	20.94913	13.85858	11.1839	2.47637
orange1.1t0	30.22817	60.89571	27.91275	75.67472	91.29159	127.9461	267.7315	210.9585	226.4299	60.28579
orange1.1t0	7.780944	8.246388	7.069249	7.790847	3.889404	2.751091	1.315811	2.52639	2.455226	4.551891
orange1.1t0	2.042218	2.747721	3.629479	3.773674	2.159887	2.042284	0.556587	1.886986	0.331493	0.615268
orange1.1t0	3.56473	4.746163	6.76007	7.591661	4.077086	5.052469	0.987033	3.521138	0.935643	1.329559
orange1.1t0	0.19269	0.282509	0.389464	1.48415	0.911056	2.745801	0	0.019623	0	0.02313
orange1.1t0	8.044852	6.751537	37.44951	25.19489	48.02817	45.88694	26.71893	67.37374	8.396263	32.31781
orange1.1t0	5.842497	6.861311	8.752677	10.83267	2.868004	5.429202	0.386197	1.420273	0.072957	0.059078
orange1.1t0	11.98398	18.8321	56.84338	52.44867	66.9755	109.8362	9.697737	14.08517	4.449502	4.374912
orange1.1t0	184.9092	132.8107	197.5812	292.7702	576.8125	552.159	1537.697	796.3817	1370.665	1161.109
orange1.1t0	343.9357	295.8225	162.6441	179.4628	16.75546	34.88474	1.944047	5.388053	3.208483	7.193536
orange1.1t0	584.5106	454.2873	287.1657	269.5416	33.22667	66.26712	3.26178	6.184736	3.006391	4.557698
orange1.1t0	2.247097	2.762616	3.542952	4.430721	1.034841	1.094647	1.820119	2.941134	0.931979	3.008751
orange1.1t0	0.369727	0.223817	1.031466	1.464642	0.6598	0.864847	0.446492	1.941771	0.081948	0.152314
orange1.1t0	2.449887	3.051514	3.257431	2.852172	1.967029	2.579761	0.71739	2.422422	0.637425	0.33966
orange1.1t0	1.926323	1.436445	3.03085	1.783407	0.822416	3.804211	0.20431	2.249176	0.046309	0.107122
orange1.1t0	5.177717	4.554433	3.216251	2.843312	0.959961	4.073956	0.119429	1.418198	0.049704	0.130397
orange1.1t0	4.413079	4.309509	7.753765	6.853648	11.59742	13.57441	9.373556	14.67217	8.760341	7.7916
orange1.1t0	13.1811	15.8588	7.132831	12.79454	7.675545	9.714858	7.152792	14.09454	3.159371	4.272337
orange1.1t0	7.512815	4.975434	6.393177	7.398777	7.725389	15.48093	4.185369	8.998057	7.576348	7.715559

orange1.1t0	2.125113	1.499782	2.848037	2.243532	3.003238	3.206276	6.744268	2.841684	3.016954	2.51876
orange1.1t0	0.564804	1.171999	0.451786	0.83613	0.237209	0.482036	0.380752	0.734771	0.232015	0.386124
orange1.1t0	3.639225	2.36947	8.418191	5.844836	6.876723	6.80218	7.937543	11.10372	12.90051	19.27631
orange1.1t0	0.789343	0.752646	3.263776	2.48883	2.88575	3.251427	2.644287	3.417056	2.421593	1.667621
orange1.1t0	0.25434	0.942232	1.653735	0.593714	2.214881	1.862188	0.060798	0.069375	0.022223	0
orange1.1t0	31.92037	15.4065	16.31153	18.97008	7.653265	26.58613	6.383915	7.674667	124.1423	76.48805
orange1.1t0	48.13154	64.11197	76.865	77.67513	52.87153	49.40258	23.79634	59.05413	13.52588	32.76748
orange1.1t0	0.806146	1.018671	3.281914	2.793877	8.15223	10.04753	8.864277	8.792351	4.851764	6.438727
orange1.1t0	13.11379	11.02138	29.0359	17.19806	47.78372	27.70329	14.47834	17.07094	17.52955	5.457118
orange1.1t0	10.2672	6.883395	19.59197	9.142415	31.85388	17.65237	11.06078	10.13092	11.08558	2.322764
orange1.1t0	54.21368	131.54	33.12227	65.27528	32.22257	20.48323	10.22056	23.63429	0.31813	0.167569
orange1.1t0	5.368632	4.645028	13.53551	7.730913	23.51376	15.91493	4.362625	6.579271	4.847307	1.367672
orange1.1t0	9.618877	8.445958	13.57702	10.8856	5.336396	7.025786	2.176642	2.695289	3.272689	3.003792
orange1.1t0	1.677773	1.641277	2.707144	2.116735	0.92742	1.827627	0.419969	1.120257	0.917774	1.057758
orange1.1t0	0.731657	0.864106	0.782683	0.739972	1.345296	1.889501	1.820806	2.148583	0.177943	0.254482
orange1.1t0	1550.075	1548.633	2932.706	3344.514	1890.566	1779.8	1472.846	501.8755	1737.522	1200.987
orange1.1t0	1.090261	1.791883	2.542636	2.462757	2.212086	2.133007	1.027298	3.022917	0.595551	1.229007
orange1.1t0	76.44732	151.1712	23.52697	57.23447	37.31774	24.97985	65.96434	67.93951	35.16186	32.42465
orange1.1t0	1.754582	2.400121	2.044058	1.73031	2.213195	1.718747	1.867062	4.347749	2.75438	4.126293
orange1.1t0	0.822927	0.723059	0.809351	0.75371	0.286508	0.617734	0.176317	0.384122	2.872421	0.951549
orange1.1t0	3.485867	2.419708	6.574336	4.706062	8.042663	10.06443	0.775755	1.862072	0.430337	0.234711
orange1.1t0	5.457764	4.813597	9.636755	10.62409	4.499399	7.781672	6.63657	8.935078	11.80004	20.52271
orange1.1t0	3.754525	3.551553	6.571975	7.222309	27.18362	27.71036	27.97026	19.30295	13.84166	4.896926
orange1.1t0	6.828865	6.651803	2.469736	2.753078	0.800653	2.091851	0.311053	0.668512	0.204142	0.152535
orange1.1t0	111.7265	95.49836	144.709	97.94441	38.67196	51.81524	8.106357	13.27894	2.716661	2.136655
orange1.1t0	193.435	166.4302	270.097	222.601	63.62643	107.2289	13.27386	26.693	4.252758	4.932666
orange1.1t0	3.863863	4.369083	4.565579	7.111366	11.7315	13.39068	9.70739	2.683389	6.961157	1.155683
orange1.1t0	3.767192	6.711595	2.30933	4.101238	5.606092	4.590186	24.73428	16.38857	8.761892	12.08596
orange1.1t0	1.461501	1.41912	1.422966	1.469274	1.862765	2.047856	1.27963	2.372069	1.648394	0.677368
orange1.1t0	2.514652	2.956627	2.809051	2.159042	1.382067	1.561676	1.18726	1.607625	0.650664	0.722816
orange1.1t0	0.596426	0.821447	0.693686	0.627042	1.320896	0.725122	1.107024	0.582967	0.501955	0.527072
orange1.1t0	191.9758	118.3045	63.02442	73.17972	40.53596	36.37156	62.2394	46.62996	228.1852	199.7787

orange1.1t0	1.6117	1.884677	1.619326	1.427003	2.636321	3.175305	3.484777	4.848853	5.06157	4.291673
orange1.1t0	0.304755	0	0.083444	0.913081	0.697768	0.602763	0.632796	2.164824	0.227617	0.041184
orange1.1t0	168.0605	211.5586	150.7455	150.67	88.35774	72.9351	72.73623	83.31907	28.90845	40.03778
orange1.1t0	0.282894	0.311448	0.712639	0.330029	0.455402	0.599453	0.144788	0.231506	0.242524	0.218498
orange1.1t0	10.38167	10.57715	13.55669	13.66925	15.71436	16.84083	7.850392	19.01496	13.41508	16.11145
orange1.1t0	20.09969	24.28394	16.39129	20.21345	0.330675	0.385645	0.307979	1.209855	0.894086	0.410006
orange1.1t0	4.037916	3.391345	4.703763	3.59084	2.50056	2.340022	1.359343	1.897424	0.886226	2.239509
orange1.1t0	5.216429	6.066751	6.016352	6.381923	2.175934	3.940003	1.395216	4.679242	4.979949	7.310432
orange1.1t0	11.47867	16.01537	17.44378	19.56996	6.192363	8.7244	7.536485	19.16997	2.018578	8.844741
orange1.1t0	11.04415	10.1216	8.413766	7.824325	3.61619	4.566303	3.499375	4.116297	1.246965	1.000147
orange1.1t0	5.311421	3.744536	2.926229	2.179105	1.377278	1.581533	1.205915	1.864299	2.534141	1.583652
orange1.1t0	3.426629	3.429462	11.74753	11.79829	2.04653	4.696631	10.36628	18.14856	8.2403	20.50652
orange1.1t0	43.54861	31.08556	90.51483	59.21107	119.3142	139.9872	91.88841	61.81386	24.468	27.533
orange1.1t0	14.59057	10.65443	16.61699	13.37735	8.843705	7.795274	5.51176	4.231393	3.272911	1.147402
orange1.1t0	4.930518	23.72014	0.715586	1.265974	22.91028	24.3067	21.64298	32.67237	56.62333	48.01414
orange1.1t0	6.806849	9.767213	3.453072	4.104324	2.203398	2.039664	1.827087	3.119176	0.358392	0.459365
orange1.1t0	103.6086	93.05268	152.5125	199.0507	49.37978	43.64872	27.31585	6.580946	5.524994	2.150008
orange1.1t0	1.525144	1.950437	1.827592	2.62561	2.110327	2.573226	0.641648	1.519417	0.774487	0.490451
orange1.1t0	3.122954	4.163921	3.690092	5.555366	2.84364	3.773284	0.973045	1.964433	0.98173	0.792362
orange1.1t0	54.38826	56.4147	55.87276	57.28832	34.24793	37.53327	20.1875	28.76062	5.943449	11.41568
orange1.1t0	1.159697	1.069911	1.087369	1.104239	0.399884	0.764569	0.294987	0.922995	0.359518	0.388664
orange1.1t0	2.390346	1.737455	5.175683	4.950426	4.140277	4.129959	3.978453	6.144509	7.098715	15.81836
orange1.1t0	0.223042	0	0.802107	0.539741	0	0.525525	0.943834	1.304476	0.547258	0
orange1.1t0	1.521343	2.128751	1.577648	1.71108	1.39877	1.91076	1.00634	4.422518	1.562026	2.106663
orange1.1t0	10.63737	13.8002	7.185863	9.511405	4.387144	5.246292	3.268737	5.986066	3.068696	3.385664
orange1.1t0	3.34285	4.991354	3.468632	4.142714	2.175007	3.450499	1.374976	2.180504	0.93318	2.109563
orange1.1t0	1.60043	0.976868	0.860627	0.435	0.025606	0.009245	0	0	0	0
orange1.1t0	0.73842	0.220268	0.688939	0.490145	0.923513	0.163901	0.595007	0.137787	0.792015	0.573257
orange1.1t0	78.65954	62.26289	60.87255	67.016	40.78442	42.94518	50.18663	25.00748	18.29197	3.472473
orange1.1t0	0.538733	0.521805	1.60667	1.218169	1.167501	1.437111	0.642929	1.581136	2.034964	4.935969
orange1.1t0	1.425066	0.813317	2.575364	2.296502	2.536201	2.790459	4.174203	2.778057	2.612336	2.834539
orange1.1t0	3.250003	1.979993	3.507131	2.960366	3.955072	4.697611	2.818796	4.798631	1.767193	1.881832

orange1.1t0	0	0	0.026246	0	0.040876	0	0.107115	0.096926	0.606813	2.070408
orange1.1t0	7.360531	10.39553	8.050987	9.063293	9.428948	8.883292	5.471497	14.71839	7.621222	11.54245
orange1.1t0	35.68893	31.66337	38.44957	45.56848	21.30462	30.42614	7.216717	10.64468	4.380573	3.218366
orange1.1t0	13.36545	15.2753	19.0029	21.80681	18.04581	35.43935	7.370778	20.66501	61.52276	51.43217
orange1.1t0	1.914766	2.443694	2.779669	3.668307	1.519457	2.955629	0.84977	2.599056	3.449352	3.544256
orange1.1t0	0.886847	0.786032	0.841995	1.110912	0.6049	1.021418	0.34004	1.525353	0.97602	1.558325
orange1.1t0	21.42738	18.6021	24.65862	22.85825	12.01117	13.37809	0.926563	2.543568	0.257144	0
orange1.1t0	19.79189	13.45189	20.45225	14.78561	9.556505	8.002106	0.981477	1.798347	0.205679	0.011042
orange1.1t0	15.28059	11.32771	18.26874	15.36828	9.439412	10.60287	1.012169	2.271716	0.312189	0.077891
orange1.1t0	2.177624	1.758315	3.373121	3.097685	0.368453	1.418629	0.263801	0.32281	0.178928	0.119169
orange1.1t0	29.2255	54.5496	19.53106	33.99179	11.00822	8.001425	9.585759	20.7663	0.832439	1.181732
orange1.1t0	20.56568	14.68233	6.229874	5.839426	5.546065	9.050227	11.56235	10.64102	1.540073	2.601519
orange1.1t0	0.808206	0.300866	1.752376	0.989115	0.410804	0.423343	0.063252	0.414682	0.017838	0.016326
orange1.1t0	3.178535	3.733288	10.05526	12.67335	0.362062	2.296632	1.158009	3.279696	0.961089	5.621095
orange1.1t0	1.883542	2.803769	2.942492	3.115185	3.133052	2.794874	0.871854	2.7255	0.7955	0.360555
orange1.1t0	5.400056	4.080349	5.407364	3.591972	2.000424	2.561266	0.718728	1.685675	0.463329	0.43912
orange1.1t0	1.30595	2.023192	2.089734	2.957818	0.88293	1.701562	0.267341	0.894236	0.112285	0.202209
orange1.1t0	0.586917	0.404894	0.344907	0.321252	1.815906	1.041978	4.151719	1.821894	1.957987	1.63311
orange1.1t0	3.926019	2.978315	2.81046	2.007274	16.1084	6.209688	37.23644	9.23261	13.2598	7.249016
orange1.1t0	3.544855	2.846209	4.280645	3.107346	1.030757	1.88841	0.658875	1.602408	0.562928	0.364258
orange1.1t0	0.999749	0.824947	1.212741	0.811811	0.219624	0.816333	0.267002	0.443339	0.551684	0.217266
orange1.1t0	3.109669	4.359451	1.675763	2.772527	1.06131	1.844485	0.30487	2.372286	0.111124	0
orange1.1t0	18.71066	11.53085	4.927525	4.25634	2.298293	2.733334	1.12858	0.891703	0.048642	0.031038
orange1.1t0	50.27409	48.94055	38.58788	56.33198	35.369	27.20531	21.36505	31.71402	26.95657	26.02526
orange1.1t0	42.84513	35.03919	64.35004	50.81778	44.44962	33.07579	16.09919	12.48171	4.072692	4.186673
orange1.1t0	72.74326	62.12352	108.1538	100.4381	69.48507	61.4595	25.03203	21.69907	6.920394	7.635891
orange1.1t0	33.0645	27.79291	23.83881	32.41337	19.2828	14.67639	13.89809	20.42526	19.316	15.54979
orange1.1t0	81.89899	73.81273	62.40834	80.8892	54.70383	39.79935	38.91939	51.48915	50.72001	40.98669
orange1.1t0	9.266376	8.235673	27.4785	24.66389	34.78562	27.4512	14.65303	36.10814	4.401416	10.07393
orange1.1t0	3.768226	5.058872	7.016632	5.554445	9.509548	9.736875	7.188956	16.76487	18.94784	17.48862
orange1.1t0	20.10785	15.29823	25.47334	32.64169	7.869294	14.76604	0.414349	7.815009	0.307799	0.897645
orange1.1t0	1.917302	2.709505	2.829067	2.865749	1.847859	2.387803	0.991866	2.851169	1.002897	2.901527

orange1.1t0	1.332905	1.404401	2.10948	2.001836	1.139962	1.502867	1.211577	2.147742	2.369495	5.969009
orange1.1t0	1.982095	0.667078	1.44086	1.252544	0.418096	2.036938	0.721194	1.479255	8.650779	11.14625
orange1.1t0	0.50495	0.624437	0.677279	0.772692	0.615775	0.678616	0.379682	0.852166	0.388307	0.666929
orange1.1t0	5.266819	4.463467	6.66083	6.39192	2.686458	4.610108	2.57201	1.96943	9.819435	3.511368
orange1.1t0	19.02912	12.97379	19.81909	22.63189	5.851686	9.548808	0.387628	4.575477	0.247188	0.471274
orange1.1t0	1.167883	0.765936	2.526721	2.015149	1.37729	2.434106	0.128999	1.442953	0.537998	0.478269
orange1.1t0	34.0335	26.78925	54.76258	27.44216	29.61125	9.449299	13.35083	7.971539	0.52598	0.15448
orange1.1t0	0.55855	1.079527	9.203285	6.646754	6.002346	9.707083	1.721554	8.81968	0.970707	0.866467
orange1.1t0	1.294051	1.182444	7.471818	4.990617	6.247847	7.522607	2.621437	9.033193	1.20415	1.819919
orange1.1t0	5.426953	5.200325	7.206151	8.424971	3.951515	6.250234	2.199256	16.66486	0.189793	0.271381
orange1.1t0	11.72943	11.3224	21.01128	20.00926	8.859344	19.44948	7.785752	36.18794	1.133686	1.873321
orange1.1t0	14.15433	17.51785	44.45271	52.4437	9.17374	28.85052	29.19776	43.33182	10.84386	59.272
orange1.1t0	1.119995	0.675384	3.529802	1.591135	3.667507	3.745014	0.183822	0.226565	0.069807	0.098438
orange1.1t0	4.810137	4.496376	11.27465	11.47473	11.09287	16.43359	6.27386	15.5161	11.88205	15.45955
orange1.1t0	2.247331	1.493998	4.348836	3.392381	2.99571	4.308234	0.5548	2.635965	0.176638	0.174631
orange1.1t0	4.505309	3.159336	8.84533	6.814435	6.578257	10.12847	1.718403	5.572699	0.306712	0.326842
orange1.1t0	1.281572	1.771904	1.610161	2.140213	1.119767	1.663529	0.865174	2.479253	0.280774	0.754439
orange1.1t0	38.65058	45.09845	43.70961	30.50311	16.87522	14.23179	15.38959	12.08851	0.19556	0.879477
orange1.1t0	0.015414	0.089141	0	0.002325	0.038235	0.012178	0.93881	1.669668	0.036861	0.358138
orange1.1t0	31.18218	19.02874	24.88841	19.3857	39.46746	28.98352	140.3097	46.6741	64.7044	22.71011
orange1.1t0	0.648968	0.830879	2.540626	2.80396	0.087676	0.400589	0.351783	0.630523	0.139458	1.022146
orange1.1t0	4.045525	4.28816	14.04335	19.38066	0.580488	3.07057	0.771986	3.768463	0.817771	6.371468
orange1.1t0	2.286973	2.039948	4.862063	4.539813	1.041266	2.7822	0.213186	1.210792	0.03636	0.103696
orange1.1t0	5.042751	5.282432	12.81196	12.90902	3.109432	7.282843	2.805265	2.937956	8.337983	12.59411
orange1.1t0	34.83701	39.44251	18.25878	19.20581	10.35493	15.94946	7.268461	19.93425	0.133607	0.262905
orange1.1t0	36.13095	40.49779	65.89035	40.50555	68.90927	92.04367	117.9614	258.1896	24.6671	36.33089
orange1.1t0	12.49374	12.4045	57.26976	42.57411	16.50337	19.23533	4.323577	7.898339	3.502434	5.03318
orange1.1t0	0.586809	0.426952	0.951252	0.712097	1.173923	1.362423	0.396117	1.215397	1.603919	1.834742
orange1.1t0	4.396752	9.273875	3.059606	4.412772	3.031367	3.145937	2.880406	4.585795	1.810462	2.553979
orange1.1t0	5.484456	3.534476	5.121876	4.051875	1.675656	2.153797	1.128718	0.884117	1.910697	1.039814
orange1.1t0	0.95363	0.30679	1.239917	0.745074	0.237087	0.225439	0.136933	0.19133	0.217177	0.136917
orange1.1t0	0.587793	1.18782	0.516018	0.936276	0.188614	0.389776	0.126684	0.79184	0.305174	0.600724

orange1.1t0	3.23722	4.812699	5.528593	5.15494	6.334273	6.820328	1.982841	6.839054	3.185851	3.977892
orange1.1t0	98.47347	82.27426	202.1557	104.6569	206.2833	66.03132	40.71217	49.23769	16.71023	8.562807
orange1.1t0	85.07849	70.81447	259.1058	124.5931	260.0315	116.8746	61.97273	60.95543	43.31967	17.57041
orange1.1t0	4.257291	5.431468	6.739965	7.342637	5.273433	6.076525	2.614862	6.899395	3.300058	3.807427
orange1.1t0	5.28326	8.78022	16.31013	15.14869	37.08072	26.36269	10.5228	17.26843	0.510677	0.969741
orange1.1t0	25.33767	32.45394	81.50975	95.03996	16.52172	52.06332	51.16706	75.56638	19.37546	94.9308
orange1.1t0	4.72008	5.160228	5.18998	4.653219	2.284647	2.736859	0.369941	1.657394	0	0.206615
orange1.1t0	3.996541	2.920988	7.441955	6.942526	8.354592	14.98035	5.733223	17.6944	27.27889	24.02602
orange1.1t0	76.08234	53.64396	21.3562	27.00237	11.71008	17.59185	8.185004	9.23636	2.35265	0.403853
orange1.1t0	416.6415	334.3232	35.42697	110.0137	31.84576	58.70057	2.396927	13.50784	0.285596	1.21339
orange1.1t0	4891.8	5540.956	121.1973	735.6864	46.25187	81.25376	5.558696	45.36957	14.11051	27.77764
orange1.1t0	9.357442	6.256643	5.577405	8.5579	1.732676	4.234953	1.621195	1.375101	3.082101	1.917687
orange1.1t0	0.398432	0.328587	0.926676	0.464848	1.133173	1.389956	1.232935	0.81273	0.336505	0.185325
orange1.1t0	1.030428	2.088125	0.729863	4.470149	2.734522	3.616859	0.136159	0.440952	0	0
orange1.1t0	2.269693	2.20962	5.198988	6.382176	5.604666	9.254299	2.140554	7.595703	3.034818	4.383476
orange1.1t0	4.052745	5.37873	2.290682	2.803837	0.781258	1.421875	0.813162	1.466743	2.081613	2.791484
orange1.1t0	1.20981	1.003859	3.622688	4.583687	0.098377	1.065106	0.249632	1.858531	0.241225	0.813632
orange1.1t0	6.47492	5.290656	4.357586	4.023571	1.154125	2.77135	0.627255	1.574135	0.049097	0.032362
orange1.1t0	1.893305	3.185573	1.050679	1.238416	0.139357	0.333321	0	0.274555	0.317946	1.374059
orange1.1t0	3.588787	2.557767	2.705633	2.062768	14.41058	7.998383	28.20569	11.11306	14.27343	9.604493
orange1.1t0	3.606108	4.070553	18.33752	17.04868	23.17382	15.10172	13.80188	32.44501	8.01258	11.16588
orange1.1t0	259.8228	217.4636	108.4843	120.9705	11.11743	20.90935	1.043371	3.040103	3.188097	9.446481
orange1.1t0	4.331727	8.687553	4.496676	4.563998	3.126231	2.825109	3.525	0.470179	2.224558	0.810117
orange1.1t0	14.88248	23.7058	12.64336	14.53841	2.053614	4.679044	4.389297	1.721292	7.845933	3.230069
orange1.1t0	70.03574	42.94231	19.7238	20.02472	9.591065	13.56169	5.210718	5.630362	0.658776	0.161132
orange1.1t0	9.94495	9.103344	8.050253	8.114656	7.657403	10.87698	6.541613	7.532192	16.95589	3.964642
orange1.1t0	2.437296	5.567765	4.698285	2.926739	2.974448	4.978507	1.122878	3.305803	4.191282	3.282212
orange1.1t0	10.19631	9.718865	7.06916	8.505047	4.735317	4.943146	5.207783	4.176561	5.22436	5.503414
orange1.1t0	1.998456	1.239354	0.904811	0.776239	0.109946	0.112279	0.594363	0.342616	0.06658	0.360787
orange1.1t0	61.08921	48.12025	67.05034	66.34042	32.34732	45.23208	13.69032	17.99428	6.753013	3.461494
orange1.1t0	1.229744	0.953102	0.666064	0.933163	0.64533	1.400003	2.835392	2.080465	16.37229	7.259735
orange1.1t0	3.069236	5.421386	8.025845	7.525263	15.89814	11.78732	4.554757	7.774368	0.168754	0.382055

orange1.1t0:	2.393586	1.274902	3.272434	2.1619	3.526718	1.186956	3.462839	1.625325	5.745876	1.429705
orange1.1t0:	3.301082	3.905733	2.405556	2.877386	2.023583	1.934489	0.266779	1.782411	0.277554	0.424017
orange1.1t0:	0.49036	0.249641	0.622232	0.789382	0.415115	0.574308	0.284841	0.752804	0.666536	1.127771
orange1.1t0:	0.391351	0.49866	1.234483	0.852811	2.260122	1.515039	2.033017	2.604644	4.177827	3.827873
orange1.1t0:	60.82075	42.14538	140.6269	84.49893	133.2668	89.97288	92.61782	122.0259	11.10671	14.60272
orange1.1t0:	1.67814	1.132358	2.014624	1.741012	2.774692	2.502629	2.992027	1.452077	3.196567	1.342432
orange1.1t0:	14.8221	18.07783	7.918236	9.495596	4.694896	6.8546	7.429756	15.7361	4.242711	12.27801
orange1.1t0:	1.785502	1.974595	6.251963	8.475759	0.115159	1.356018	0.370761	1.693995	0.282784	2.856656
orange1.1t0:	15.69278	10.82482	9.690698	10.62021	20.64638	19.47851	23.0169	6.281142	2.866474	1.164257
orange1.1t0:	18.92322	19.33924	7.08781	9.863979	2.617442	3.038208	1.781152	1.310233	0.770632	0.756482
orange1.1t0:	4.193406	3.597571	9.062974	6.360682	2.502338	3.582888	1.006674	1.931073	0.229399	0.318636
orange1.1t0:	9.142187	11.74197	26.69334	27.67038	5.879365	15.29145	22.51989	21.64063	5.052188	18.70392
orange1.1t0:	4.133448	3.132262	3.501807	3.673638	3.812856	2.864523	6.71054	2.332848	0.70403	2.566327
orange1.1t0:	0.09729	0.361925	0.630007	0.877976	2.429263	1.155529	1.553255	2.434786	7.207873	18.78204
orange1.1t0:	1.632483	2.076487	1.7343	1.110415	1.845008	0.670522	0.663438	1.241394	0.420833	1.093752
orange1.1t0:	3.252475	4.202559	12.12879	10.84612	15.01853	13.38333	12.41783	13.7178	8.929486	12.01757
orange1.1t0:	1.153435	1.075263	3.085068	4.076576	0.103024	0.88766	0.217093	1.642816	0.241225	0.643348
orange1.1t0:	1.662186	1.880143	1.561947	1.810845	1.718439	3.374426	0.9525	2.234106	2.868571	1.614339
orange1.1t0:	3.100621	2.794539	5.501082	4.262363	3.510922	4.868755	2.624381	5.502295	3.177071	5.203444
orange1.1t0:	1.22215	0.844049	2.837717	1.891866	4.485943	3.199967	1.211459	0.993193	0.82395	0.579451
orange1.1t0:	2.505933	1.776971	2.210938	1.098975	1.213272	1.326424	1.764313	0.722972	0.897549	0.713114
orange1.1t0:	1.719237	2.209859	1.876026	2.288953	2.324036	5.00092	1.591991	3.593016	12.42248	6.365845
orange1.1t0:	46.61994	68.85559	80.2138	79.94495	116.9311	146.1139	20.90167	61.7461	4.400122	6.702806
orange1.1t0:	17.69098	21.87275	25.86451	24.11137	19.87445	18.81817	7.806269	8.150359	2.332999	3.171011
orange1.1t0:	12.02273	7.187458	7.825701	6.717177	5.009462	8.596141	3.631011	3.239183	1.956728	1.542916
orange1.1t0:	5.34989	8.367443	1.570895	4.440053	0.352933	0.27355	0.383598	0.421706	0.145268	0.11799
orange1.1t0:	4.612152	5.579283	1.897081	1.853591	0.292503	0.794148	0	0.363646	0.122142	0.391332
orange1.1t0:	18.17512	15.01828	11.81709	10.86881	4.071347	10.14745	1.934862	6.314006	0.107327	0.244554
orange1.1t0:	5.716115	6.600649	4.579595	4.992571	4.116925	6.222447	1.443698	5.421524	4.267484	3.624256
orange1.1t0:	1.640054	1.675745	1.445967	1.874245	1.159169	1.517638	0.767069	2.088853	1.686142	1.537963
orange1.1t0:	0.511729	0.720147	1.779077	1.861949	0.190005	0.716987	0.450399	1.065801	0.409668	0.55671
orange1.1t0:	39.27987	15.63682	65.89509	61.75747	51.60472	194.9194	126.7041	69.42108	612.9795	295.0643

orange1.1t0:	2.162196	1.512483	1.900885	1.165262	3.334542	2.311869	7.370918	1.292978	0.997775	0.780363
orange1.1t0:	0.697657	1.066246	0.949211	0.769127	0.699267	0.59462	1.579773	1.160587	0.891364	0.343212
orange1.1t0:	4.527251	5.887877	4.821266	4.762715	3.287737	2.641681	2.319629	4.477552	2.186677	1.783891
orange1.1t0:	0.864763	0.989216	0.71528	0.608531	0.693581	1.180583	2.068814	0.442809	1.516671	0.866753
orange1.1t0:	2.564162	0.861065	1.720064	1.209089	0.847492	1.967399	0.749933	1.957239	0.120261	0.681264
orange1.1t0:	7461.547	8508.069	1241.347	3265.133	162.2237	473.9404	25.75522	40.2878	4.360161	5.89426
orange1.1t0:	4.24231	6.337373	3.417932	5.05568	2.135801	3.36253	2.384774	5.228669	3.73581	10.03744
orange1.1t0:	3.449336	4.197892	2.997318	2.751806	0.489203	1.83702	0.322988	2.082907	0	0.138472
orange1.1t0:	0.192337	0.201425	1.053815	0.794431	2.251197	3.350457	0.610421	1.726285	4.564513	4.624085
orange1.1t0:	0.174217	0.051601	0.183264	0.335924	1.643702	1.387798	2.382661	1.650133	19.62619	6.055815
orange1.1t0:	0.478582	0.143915	0.410474	0.907346	1.604277	4.649259	2.547245	6.549622	17.0901	30.66483
orange1.1t0:	0	0.121216	0.11381	0.064873	0.401403	0.526661	0.592237	0.826008	8.945797	1.837709
orange1.1t0:	1.762746	1.53003	3.913981	3.093547	0.836824	0.991538	0.801773	1.562481	0.908012	1.412031
orange1.1t0:	26.81522	35.34738	45.01119	36.88624	62.56583	64.46922	15.5034	25.09758	2.279534	2.97331
orange1.1t0:	13.45491	18.05452	16.80423	26.2372	4.320937	8.874421	5.222171	17.75655	13.11275	66.09777
orange1.1t0:	0.88845	0.494422	0.728747	0.234066	0.209923	0.179065	0.3344	0.211148	0.171352	0.198316
orange1.1t0:	8.040956	9.503557	26.03953	20.92883	39.68305	39.45062	44.11762	55.13841	10.43514	27.80745
orange1.1t0:	0.15158	0.073212	0.258243	0.332873	0.560205	0.499278	1.222929	1.045591	0.991909	1.611733
orange1.1t0:	12.17389	13.35811	13.43355	12.37696	16.15672	18.72792	6.033022	21.67387	16.68901	16.40418
orange1.1t0:	0.534684	0.654013	0.692455	0.651396	0.43365	1.085549	0.314414	1.080197	0.196791	0.918958
orange1.1t0:	342.8642	361.8416	238.0546	329.5619	196.9924	415.5906	109.5464	201.0263	236.5108	343.2022
orange1.1t0:	1.356321	1.466866	0.892605	0.909183	0.449946	0.96098	0.405807	0.979958	0.374569	0.627396
orange1.1t0:	0.033019	0.299246	0.495907	0.382567	8.896266	13.37993	39.65877	41.15836	44.03461	77.55528
orange1.1t0:	1.78016	1.676492	2.564047	1.646346	3.375774	3.035437	5.359769	2.99655	3.406125	3.484496
orange1.1t0:	3.206277	3.519224	3.063247	2.958049	0.821771	1.412692	2.362713	2.355364	4.562109	14.66213
orange1.1t0:	2.049003	4.487427	1.907637	3.122926	2.043292	0.972442	2.290738	1.570335	0.365276	0.418178
orange1.1t0:	90.65877	130.4271	146.2408	185.1984	29.08917	49.35434	79.60213	144.1135	57.17237	281.2466
orange1.1t0:	1.145998	2.671716	3.420657	4.533703	10.13001	5.619942	14.06081	32.95914	179.1322	141.5885
orange1.1t0:	3.69588	3.238019	5.3203	4.991847	1.813827	3.758956	4.094008	3.155652	5.459924	5.15062
orange1.1t0:	2.956611	2.605258	10.19213	6.914932	10.81246	6.210421	5.65415	10.86178	2.762801	2.205669
orange1.1t0:	3.944436	1.976906	2.858763	5.554052	0.261867	2.367982	1.006886	2.046919	0.153636	0
orange1.1t0:	2.252658	3.552257	4.571665	4.78458	3.314133	5.881648	1.228392	2.267879	4.037816	2.030012

orange1.1t0:	2.603348	2.677433	1.14102	1.831835	0.353829	0.401005	0.126807	1.087353	0.525893	0.804746
orange1.1t0:	0.951483	0.726121	0.810207	0.980845	0.548343	0.729243	3.2939	0.937344	4.558373	3.399206
orange1.1t0:	0.712561	0.332796	3.549445	0.946293	25.72925	25.05812	23.03789	18.15182	31.5799	32.97554
orange1.1t0:	0.45365	0.819302	0.585521	0.448041	0.649828	0.422666	0.620652	0.895686	0.332395	0.592449
orange1.1t0:	0.261117	0.12764	1.229978	1.108063	1.534602	3.274091	2.082202	3.088987	1.517644	0.898996
orange1.1t0:	3.825131	3.410137	2.907802	3.346863	1.83155	3.368276	1.649082	3.424617	0.981602	0.308315
orange1.1t0:	96.05077	69.0016	80.62063	83.2136	168.7946	164.2277	140.7663	51.62942	28.33455	12.09387
orange1.1t0:	0.77642	0.292996	0.374673	0.213166	0.434575	0.351437	0	0.286136	0.176641	0.608587
orange1.1t0:	1.557682	2.209563	2.550681	2.955209	2.93674	2.788337	4.410707	3.911717	1.617631	3.558858
orange1.1t0:	4.101214	2.17918	0.594822	1.504	0.574101	0.712352	0.547361	0.410723	0.795136	0.700417
orange1.1t0:	0.659859	0.641841	1.25673	0.772829	1.447641	1.360859	0.353516	1.231658	0.101329	0.160461
orange1.1t0:	9.170999	9.725566	4.22692	7.906581	1.373325	1.967117	1.574301	4.997583	2.249681	9.221152
orange1.1t0:	115.6466	85.5844	88.02271	82.54964	29.57939	43.79577	9.076172	19.80755	10.24861	4.64854
orange1.1t0:	2.128383	2.670714	2.594576	1.328942	2.052324	2.102238	1.901014	1.795285	0.846302	0.786589
orange1.1t0:	1.875769	1.183682	1.704657	1.451425	2.08959	1.597118	0.505691	1.509349	0.424813	0.553153
orange1.1t0:	5.240801	5.797862	16.86646	22.34156	2.446566	5.741823	3.357621	10.73965	0.743545	5.938066
orange1.1t0:	1.25544	1.00331	2.099958	1.220263	3.58168	1.731126	5.379787	3.187747	1.916152	1.62049
orange1.1t0:	17.76073	36.67144	7.733849	11.64527	4.605519	4.161318	1.304827	4.793842	0.834983	1.358983
orange1.1t0:	38.42441	30.11153	38.9237	32.26141	53.68197	46.80625	101.0866	65.00125	53.88727	45.31067
orange1.1t0:	0.617124	0.468458	0.566796	0.578182	0.874642	0.666009	0.317912	0.986	0.672601	0.653415
orange1.1t0:	8.574711	8.453201	6.187973	6.040108	3.940085	4.154629	2.436044	4.847272	5.399215	4.563196
orange1.1t0:	0.293223	0.351465	0.797082	0.643209	0.849608	0.490853	0.395148	1.207116	0.992736	1.959877
orange1.1t0:	9.069545	10.74002	17.45987	17.50466	16.57648	18.13516	6.191119	17.16669	8.120275	10.7193
orange1.1t0:	4.262909	5.969107	6.965968	6.113075	6.986556	7.48334	2.89459	9.193261	5.947513	4.871628
orange1.1t0:	3.521292	5.753288	4.135028	4.663352	2.848747	3.811164	1.110325	5.939939	4.393414	4.821098
orange1.1t0:	3.069265	3.266673	4.669865	4.077563	5.103183	5.985596	1.518515	5.45466	4.880131	6.410106
orange1.1t0:	11.77737	12.35097	11.72801	14.85166	5.819061	7.233068	2.957969	9.912324	13.93616	18.09266
orange1.1t0:	10.23183	16.51105	10.22094	11.47545	9.612106	9.553249	6.342528	18.60465	7.814883	10.54215
orange1.1t0:	1.159767	1.172169	1.163304	1.071907	0.901475	1.100514	0.291899	0.920914	0.359588	0.313813
orange1.1t0:	0.435869	0.262984	0.561126	0.798495	0.810041	0.98694	0.595245	1.072477	0.300201	0.98064
orange1.1t0:	1.02024	1.126226	1.483503	1.215301	2.281651	2.181571	0.521823	2.87721	0.987868	0.758259
orange1.1t0:	1.170486	0.842298	4.221368	1.879314	3.165243	2.104207	1.274556	3.440362	2.611587	2.446443

orange1.1t0	0.222328	0.134172	0.500964	0.348143	2.614419	0.676064	1.84936	3.31663	1.403488	1.162705
orange1.1t0	8.574052	11.03824	9.110452	11.78375	6.364499	9.874308	4.532307	20.68345	13.77677	16.40932
orange1.1t0	0.554696	0.505137	0.886163	0.953403	0.494247	1.044203	0.387917	0.844797	0.566375	0.652071
orange1.1t0	7.596415	7.066887	6.791512	5.960061	6.50584	7.033383	1.969996	4.925268	0.267208	0.256042
orange1.1t0	1.149454	1.794311	2.070544	2.167625	1.800246	2.049734	0.993801	2.768051	1.974437	2.630858
orange1.1t0	0.074013	0	0.016989	0	0.165504	0.123386	1.178299	0.169286	2.687504	1.719779
orange1.1t0	8.502891	10.50266	9.486408	10.44382	12.43414	14.31058	7.007106	19.59068	10.67358	16.44963
orange1.1t0	0.408626	0.432034	0.291597	0.380974	0.421449	0.713171	0.327166	0.977601	0.504967	0.545619
orange1.1t0	2.800919	2.705691	2.78947	3.072214	2.049758	2.308533	1.000196	3.426473	3.633242	3.29115
orange1.1t0	9.339423	9.842687	8.529394	9.058138	7.694514	10.62692	3.687007	10.63061	9.5592	9.504305
orange1.1t0	0.162856	0.254076	0.399055	0.496099	0.360661	0.556773	0.163384	0.885497	0.874808	1.194756
orange1.1t0	7.756592	6.21963	13.60317	13.40697	7.382662	7.26465	3.586397	12.15901	4.56446	13.10555
orange1.1t0	1.699795	1.588159	1.830086	1.449878	0.95109	0.869094	0.263171	1.095454	1.609374	1.50244
orange1.1t0	0.417757	0.674871	0.404599	0.471585	0.672956	0.59532	0.360054	0.800492	0.430787	0.281952
orange1.1t0	5.042318	5.377637	2.985313	3.031342	1.824272	1.878849	0.650677	1.743047	0.538073	0.429741
orange1.1t0	4.685416	5.99777	6.218648	5.746796	5.778891	5.646356	2.413386	6.181642	2.302854	2.528595
orange1.1t0	5.532637	7.331794	9.065712	8.912294	2.398402	2.425486	1.096413	5.73961	1.825463	4.058554
orange1.1t0	7.350588	7.668001	8.611309	6.502958	5.908918	6.381357	2.307943	3.202622	1.887313	2.259842
orange1.1t0	4.566757	5.533329	5.144259	6.093313	4.063046	6.737987	2.742769	8.029936	5.946848	6.02122
orange1.1t0	1.932867	1.969701	2.225115	1.918851	1.84106	2.846407	0.470759	3.360358	2.070013	0.994057
orange1.1t0	0.421319	0.230884	0.535612	0.578429	0.687293	1.015324	0.433182	1.258019	0.654961	1.088957
orange1.1t0	13.3128	17.87206	15.23486	20.42422	2.888343	6.88626	3.107896	10.72631	1.92584	9.029584
orange1.1t0	19.62522	21.82711	12.21838	12.90035	3.799833	4.115763	1.994726	3.701325	1.406833	1.396123
orange1.1t0	12.29569	12.44488	11.0774	12.67788	4.418394	5.325224	2.018144	7.031624	6.323401	17.55388
orange1.1t0	1.258045	1.813427	1.50383	1.621782	1.241218	1.188846	0.491939	1.90437	0.684585	0.84707
orange1.1t0	0.99706	1.191204	0.580182	0.920608	0.437355	0.753198	0.267766	0.831873	0.676638	0.61216
orange1.1t0	2.055848	2.362007	1.702986	2.324494	1.399469	2.134064	0.75472	2.313377	1.115725	1.156682
orange1.1t0	0.765087	1.346102	1.116707	1.714658	0.60043	1.623717	0.445088	1.209765	0.516017	0.59794
orange1.1t0	19.91091	23.52106	30.37721	29.25893	34.78644	37.82215	17.69115	41.98716	21.5046	20.33851
orange1.1t0	1.396131	1.269919	1.648159	2.648583	1.09507	1.562248	0.712744	1.323502	0.667332	0.729035
orange1.1t0	2.343944	3.330852	4.382846	3.700693	4.472957	4.863027	2.313778	7.225594	4.218058	3.864492
orange1.1t0	3.653913	4.26126	4.775004	4.356404	5.601719	4.225441	1.987919	5.989344	2.873885	3.639859

orange1.1t0	0.962895	0.749913	1.730782	1.420776	2.424054	2.655825	1.808473	2.885648	1.881639	1.772643
orange1.1t0	7951.216	7630.304	2383.1	3389.505	619.8999	2077.248	560.4013	3796.54	270.1231	1225.548
orange1.1t0	1.809303	2.458937	2.049638	1.856297	1.70499	2.66102	0.77305	3.327238	3.596281	2.600265
orange1.1t0	2.209051	2.884672	2.153236	1.649652	1.209578	1.114434	0.537883	1.674542	0.330849	1.16022
orange1.1t0	2.300068	2.887047	1.777702	2.035083	1.329104	1.87609	0.668473	2.318593	0.364223	0.406692
orange1.1t0	2.346344	2.292287	2.119854	1.966645	3.144909	3.322087	1.343185	4.461074	1.59255	1.124806
orange1.1t0	1.365561	1.199372	2.030225	1.960002	1.639406	2.205803	0.870811	2.702845	3.28051	6.694356
orange1.1t0	0.271228	0.933243	0.213284	0.346516	0.296897	0.736811	0.016571	0.88539	0.989807	1.648525
orange1.1t0	14.51487	16.78945	17.33382	20.11244	10.97932	19.7214	6.169953	20.71974	20.01758	24.38492
orange1.1t0	18.76654	19.66259	19.51744	27.64024	11.16996	14.00699	4.757097	6.830249	3.244484	1.460466
orange1.1t0	1.617119	1.82307	1.363612	1.48284	0.346538	1.189024	0.237622	1.349381	0.229113	0.628175
orange1.1t0	0.463151	0.29985	0.480839	0.863849	0.224484	1.007735	0.247042	1.566227	0.847292	1.140986
orange1.1t0	9.93443	10.02576	19.90521	15.78183	14.75479	20.36363	3.639193	12.27804	8.771627	6.270379
orange1.1t0	5.407951	5.528716	7.018815	6.916033	4.714873	6.132046	2.327021	7.2272	8.702338	10.43834
orange1.1t0	2.744123	2.819718	2.905913	2.72796	3.614973	3.885739	1.197735	3.627678	3.911142	2.955614
orange1.1t0	6.121638	8.052457	2.426127	2.888193	1.207594	1.271933	1.516459	1.709462	0.036293	0.449108
orange1.1t0	9.619769	8.116061	9.685508	11.10605	3.014341	5.248168	1.092569	1.585244	1.62349	2.030115
orange1.1t0	2.615092	3.064231	2.290373	2.896639	1.490071	0.84569	0.240447	0.77135	0.202045	0.185725
