# Supplementary figures and images for: Transcription factor CsESE3 positively modulates both jasmonic acid and wax biosynthesis in citrus
Source: aBIOTECH. 2022 Nov 22;3(4):250–66. doi: 10.1007/s42994-022-00085-2 (PMC9755798; doi:10.1007/s42994-022-00085-2)

**Supplementary Figures**

Fig.S1

Fig.S2


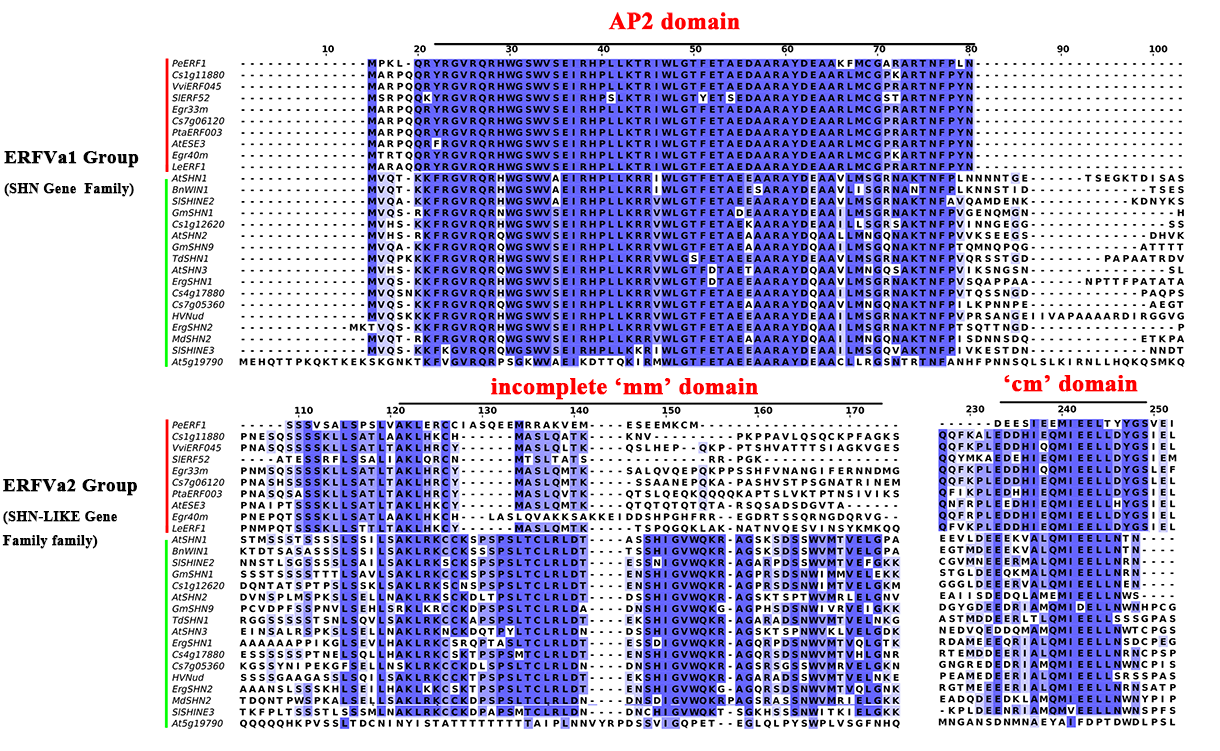


Fig. S3

**
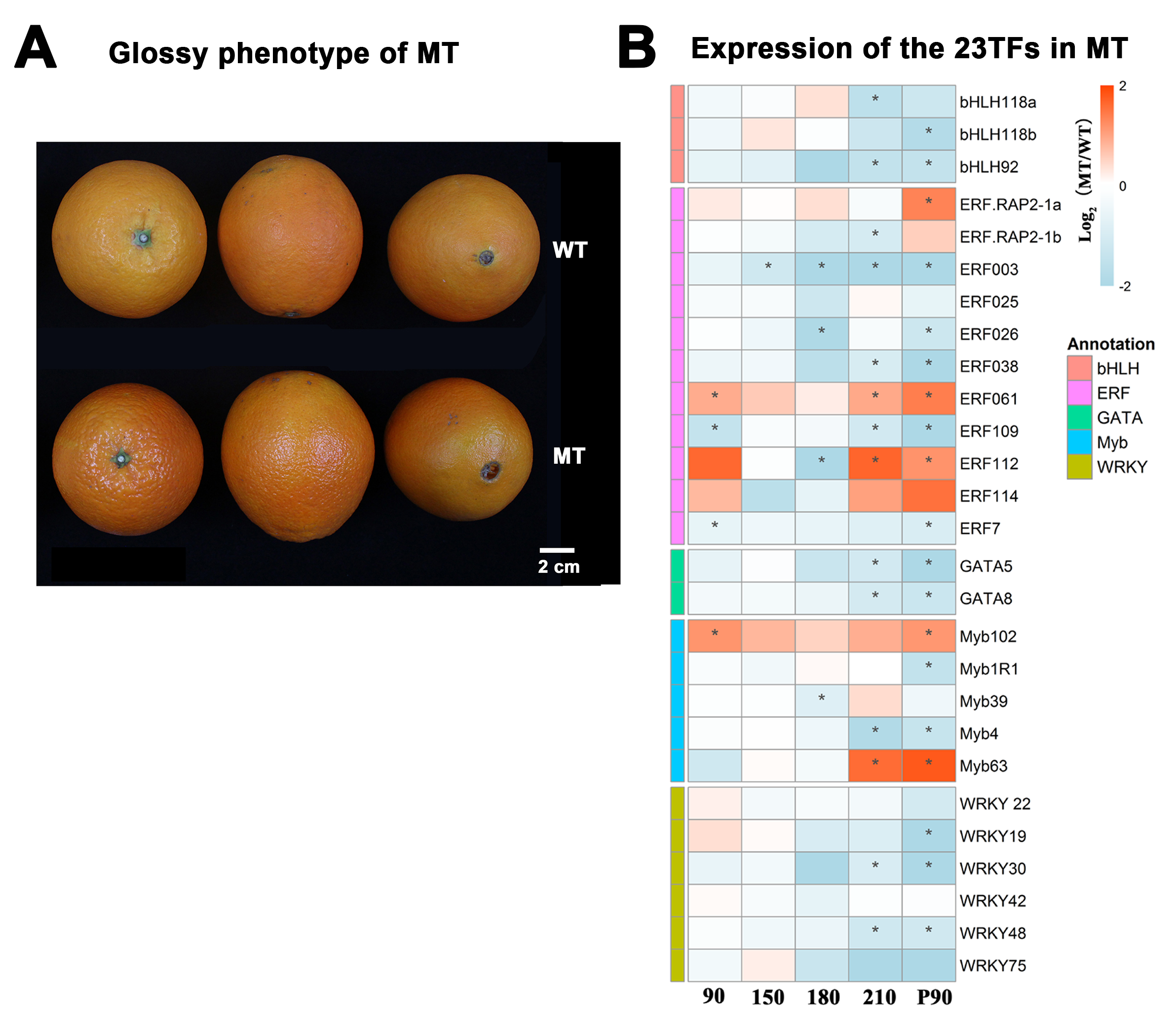
**

Fig. S4


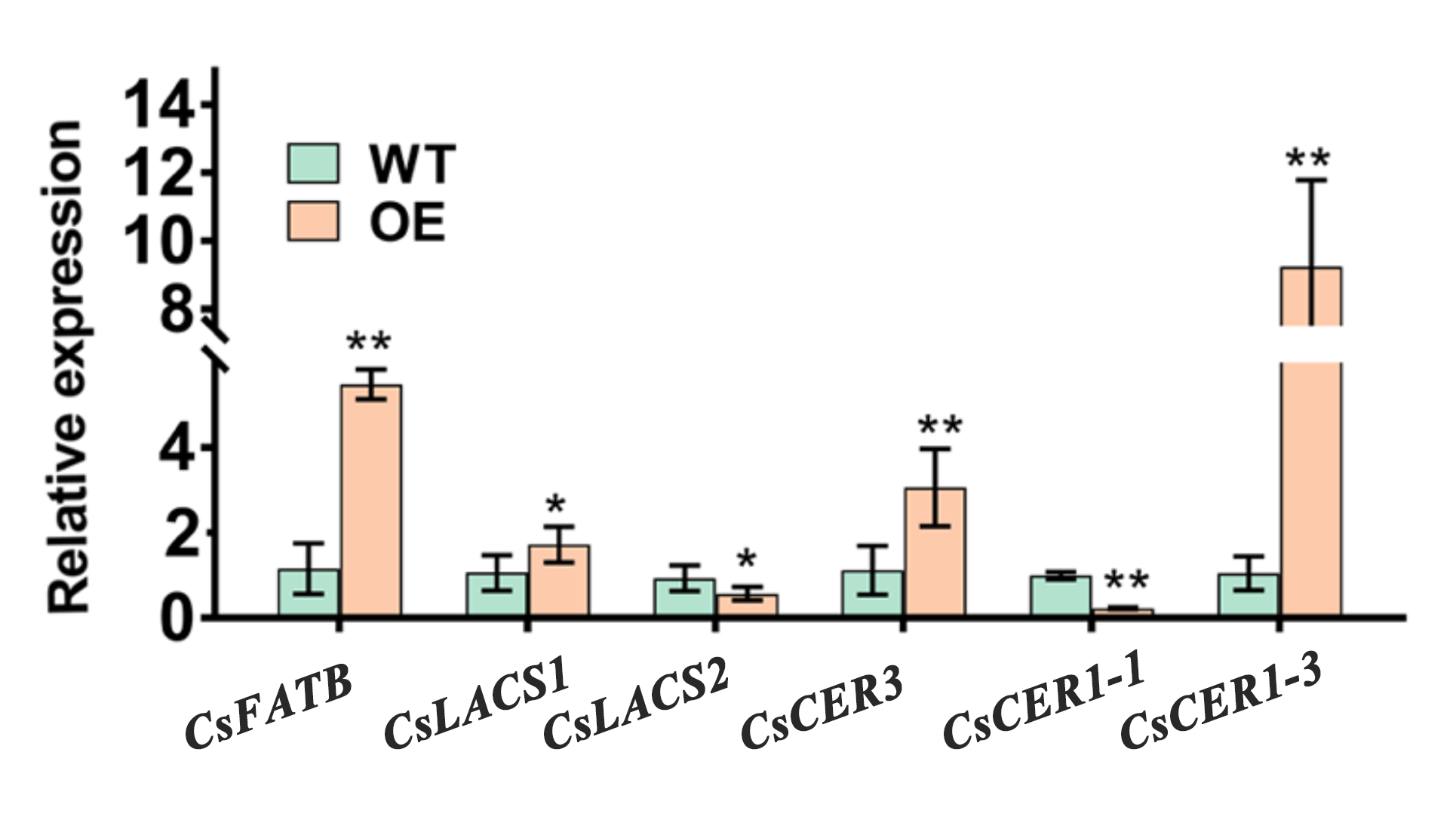


Fig.S5

**
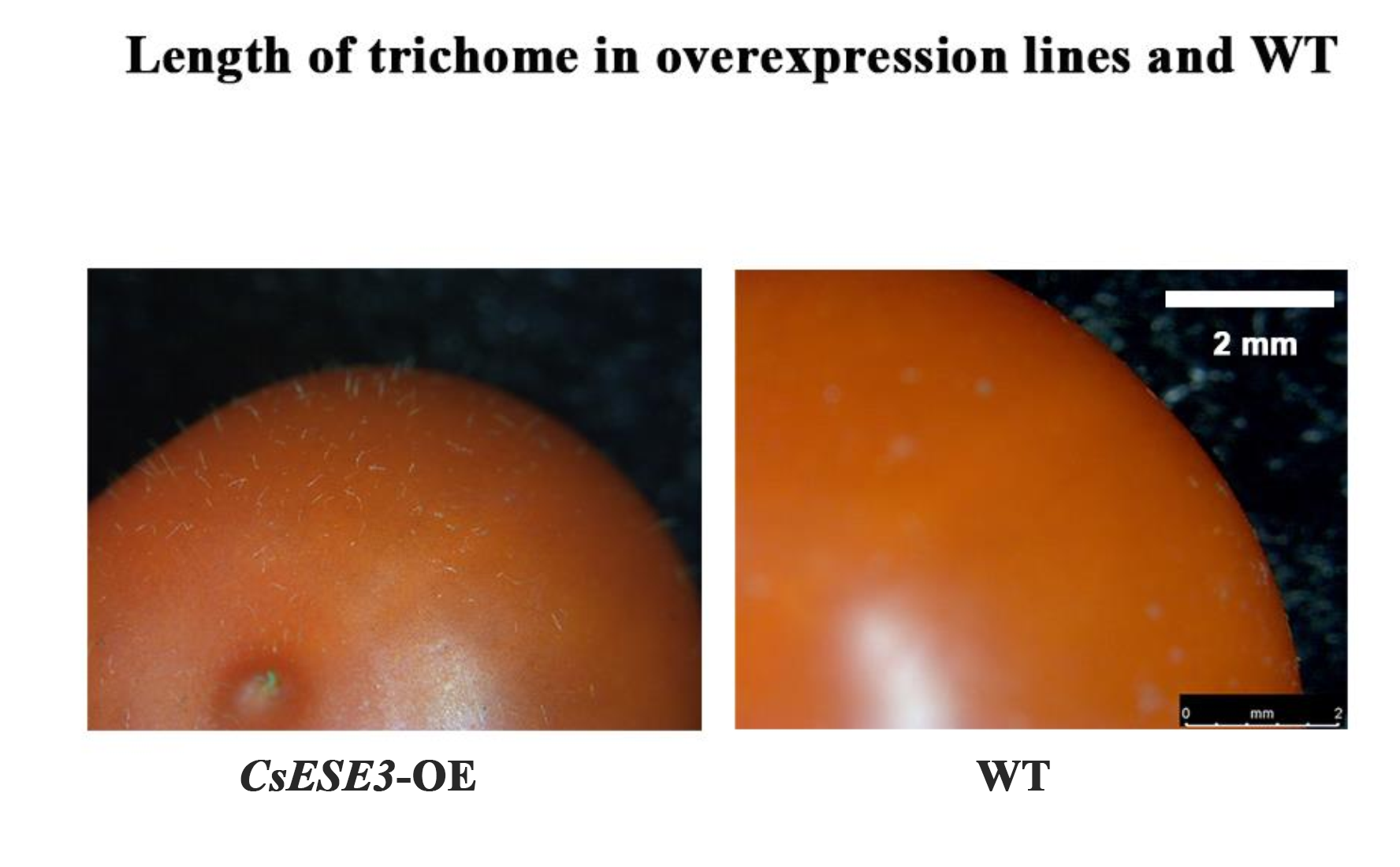
**

Fig.S6

Fig. S7


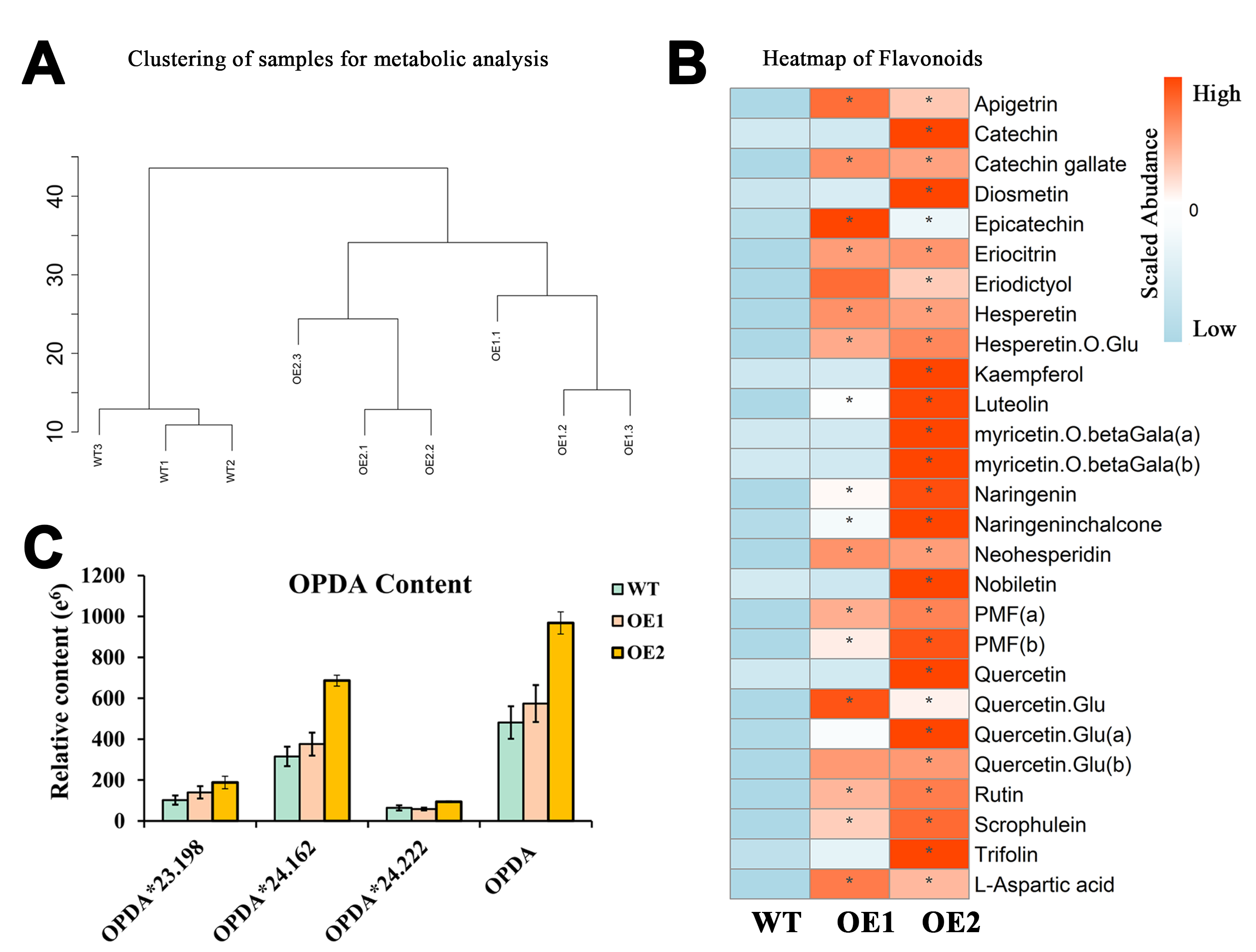


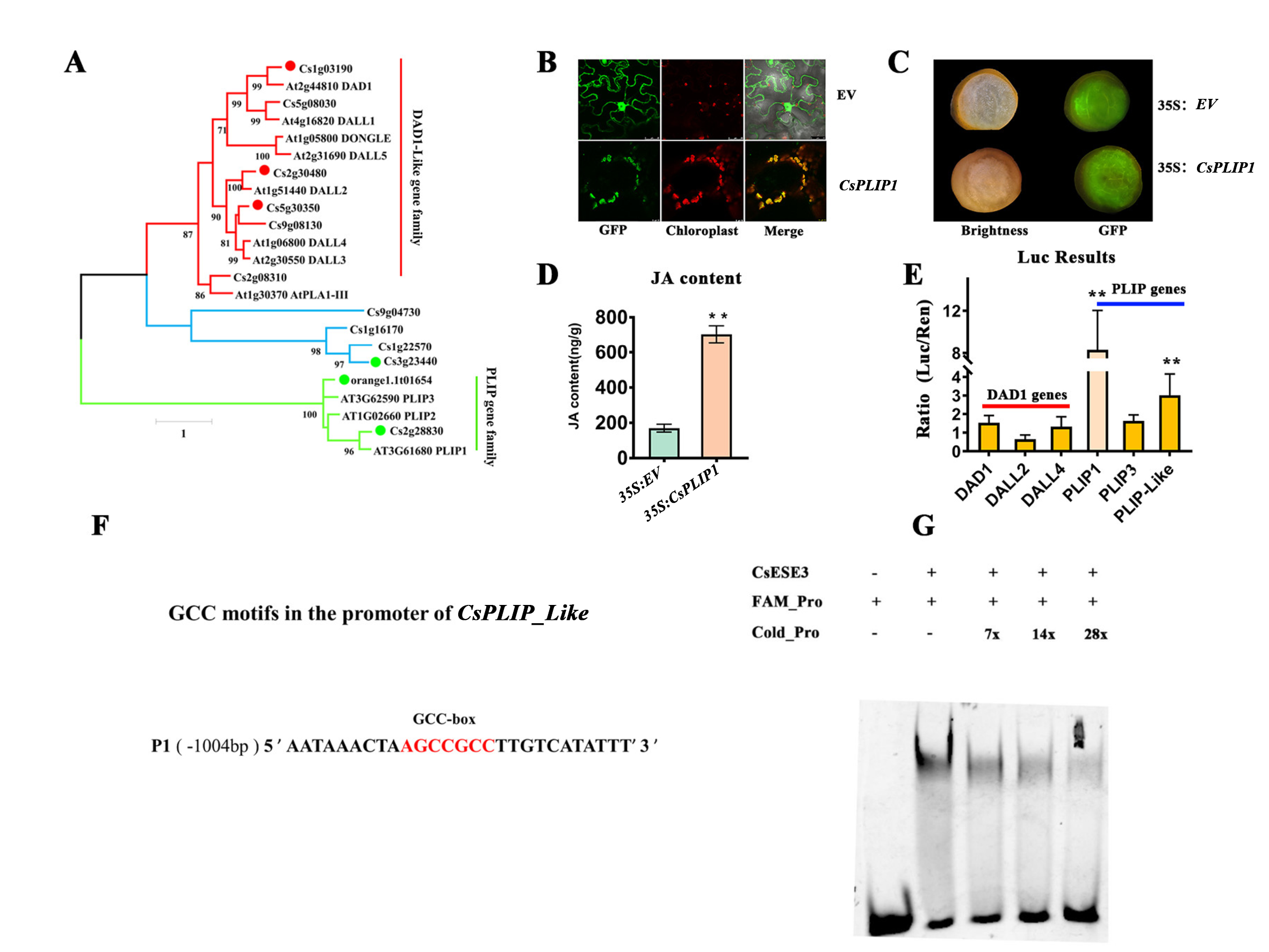
Fig.S8

Fig.S9


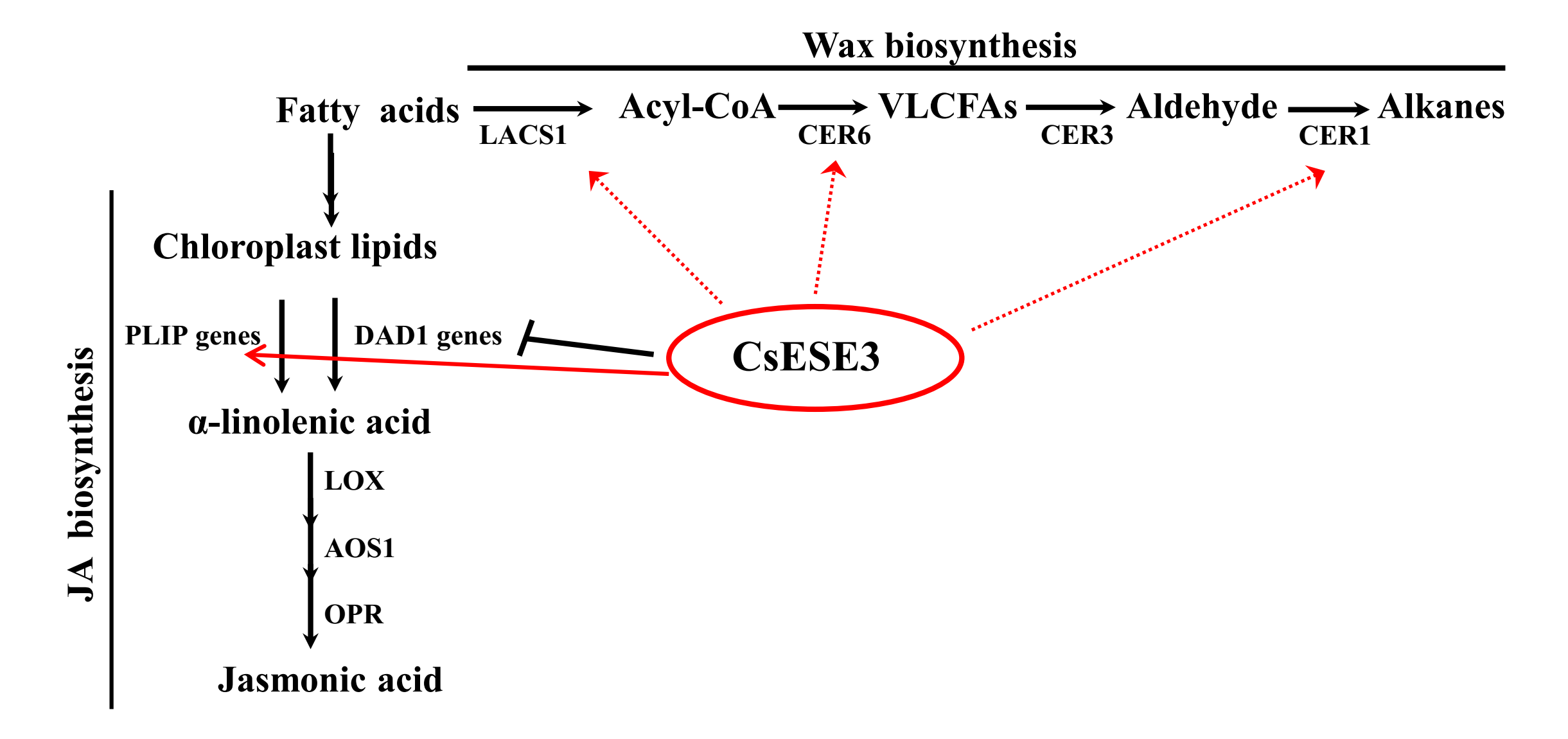

Supplement: Supplementary file 4 — Supplementary file4 (DOC 7297 KB) [file 42994_2022_85_MOESM4_ESM.doc]
